# Rethinking Transcriptional Activation in the *Arabidopsis* Circadian Clock

**DOI:** 10.1371/journal.pcbi.1003705

**Published:** 2014-07-17

**Authors:** Karl Fogelmark, Carl Troein

**Affiliations:** 1 Computational Biology and Biological Physics, Department of Astronomy and Theoretical Physics, Lund University, Lund, Sweden; North Carolina State University, United States of America

## Abstract

Circadian clocks are biological timekeepers that allow living cells to time their activity in anticipation of predictable daily changes in light and other environmental factors. The complexity of the circadian clock in higher plants makes it difficult to understand the role of individual genes or molecular interactions, and mathematical modelling has been useful in guiding clock research in model organisms such as *Arabidopsis thaliana*.

We present a model of the circadian clock in *Arabidopsis*, based on a large corpus of published time course data. It appears from experimental evidence in the literature that most interactions in the clock are repressive. Hence, we remove all transcriptional activation found in previous models of this system, and instead extend the system by including two new components, the morning-expressed activator RVE8 and the nightly repressor/activator NOX.

Our modelling results demonstrate that the clock does not need a large number of activators in order to reproduce the observed gene expression patterns. For example, the sequential expression of the *PRR* genes does not require the genes to be connected as a series of activators. In the presented model, transcriptional activation is exclusively the task of RVE8. Predictions of how strongly RVE8 affects its targets are found to agree with earlier interpretations of the experimental data, but generally we find that the many negative feedbacks in the system should discourage intuitive interpretations of mutant phenotypes. The dynamics of the clock are difficult to predict without mathematical modelling, and the clock is better viewed as a tangled web than as a series of loops.

## Author Summary

Like most living organisms, plants are dependent on sunlight, and evolution has endowed them with an internal clock by which they can predict sunrise and sunset. The clock consists of many genes that control each other in a complex network, leading to daily oscillations in protein levels. The interactions between genes can be positive or negative, causing target genes to be turned on or off. By constructing mathematical models that incorporate our knowledge of this network, we can interpret experimental data by comparing with results from the models. Any discrepancy between experimental data and model predictions will highlight where we are lacking in understanding. We compiled more than 800 sets of measured data from published articles about the clock in the model organism thale cress (*Arabidopsis thaliana*). Using these data, we constructed a mathematical model which compares favourably with previous models for simulating the clock. We used our model to investigate the role of positive interactions between genes, whether they are necessary for the function of the clock and if they can be identified in the model.

## Introduction

The task of the circadian clock is to synchronize a multitude of biological processes to the daily rhythms of the environment. In plants, the primary rhythmic input is sunlight, which acts through photoreceptive proteins to reset the phase of the clock to local time. The expression levels of the genes at the core of the circadian clock oscillate due to mutual transcriptional and post-translational feedbacks, and the complexity of the feedbacks makes it difficult to predict and understand the response of the system to mutations and other perturbations without the use of mathematical modelling [1].

Early modelling of the system by Locke *et al*. demonstrated the feasibility of gaining new biological insights into the clock through the use of model predictions [2]. The earliest model described the system as a negative feedback loop between the two homologous MYB-like transcription factors CIRCADIAN CLOCK ASSOCIATED 1 (CCA1) and LATE ELONGATED HYPOCOTYL (LHY) [3], [4] on one hand and TIMING OF CAB EXPRESSION 1 (TOC1/PRR1) [5] on the other. Over the past decade, models have progressed to describing the system in terms of multiple interacting loops, still centred around LHY/CCA1 (treated as one component) and TOC1. The latest published model by Pokhilko *et al*. (2013) describes transcriptional and post-translational interactions between more than dozen components. We refer to that model as P2012 [6], in keeping with the tradition of naming the *Arabidopsis* clock models after author and submission year (cf. L2005 [2], L2006 [7], P2010 [8] and P2011 [9]).

The clock depends on several genes in the PSEUDO RESPONSE REGULATOR (PRR) family: *PRR9*, *PRR7*, *PRR5*, *PRR3* and *TOC1/PRR1* are expressed in a clear temporal pattern, with *PRR9* mRNA peaking in the morning, *PRR7* and *PRR5* before and after noon, respectively, and *PRR3* and *TOC1* near dusk [10]. PRR9, PRR7 and PRR5 act to repress expression of *CCA1* and *LHY* during the day [11], but, until recently, TOC1 was thought to be a nightly activator of *CCA1* and *LHY*, acting through some unknown intermediate. However, TOC1 has firmly been shown to be a repressor of both *CCA1* and *LHY*, and it now takes its place in the models as the final repressor of the “PRR wave” [9], [12]–[14]. PRR3 has yet to be included in the clock models and the roles of the other PRRs are being reevaluated following the realization that TOC1 acts as a repressor [15].

The GIGANTEA (GI) protein has long been thought to form part of the clock [16], whereas EARLY FLOWERING 3 (ELF3) was known to affect clock function [17] but was only more recently found to be inside the clock, rather than upstream of it [18], [19]. GI and ELF3 interact with each other and with other clock-related proteins such as the E3 ubiquitin-ligase COP1 [20]. GI plays an important role in regulating the level and activity of ZEITLUPE (ZTL) [21], which in turn affects the degradation of TOC1 [22] and PRR5 [23] but not of the other PRRs [24]. The clock models by Pokhilko *et al*. include GI and ZTL; GI regulates the level of ZTL by sequestering it in a GI-ZTL complex during the day and releasing it at night [8].

Together with EARLY FLOWERING 4 (ELF4) and LUX ARRHYTHMO (LUX), ELF3 is necessary for maintaining rhythmicity in the clock [25]–[27]. The three proteins are localized to the nucleus, and ELF3 is both necessary and sufficient for binding ELF4 and LUX into a complex termed the evening complex (EC) [19]. In recent models, EC is a major repressor; it was introduced in P2011 to repress the transcription of *PRR9*, *LUX*, *TOC1*, *ELF4* and *GI* [9].

We here present a model (F2014) of the circadian clock in *Arabidopsis*, extending and revising the earlier models by Pokhilko *et al*. (P2010–P2012). To incorporate as much as possible of the available knowledge about the circadian clock into the framework of a mathematical model, we have compiled a large amount of published data to use for model fitting. These curated data are made available for download as described in Methods.

The aim of this work is to clarify the role of transcriptional activation in the *Arabidopsis* circadian clock. Specifically, we use modelling to test whether the available data are compatible with models with and without activation. There is no direct experimental evidence for any of the activators postulated in earlier models, and as a crucial step in remodelling the system we have removed all transcriptional activation from the equations. Instead, we have added a major clock component missing from earlier models: the transcription factor REVEILLE 8 (RVE8), which positively regulates the expression of a large fraction of the clock genes [28], [29]. A further addition is the nightly transcription factor NOX/BROTHER OF LUX ARRHYTHMO (NOX/BOA), which is similar to LUX but may also act as an activator of *CCA1* [30]. By examining transcriptional activation within the framework of our model, we have clarified the relative contributions of the activators to their different targets.

## Results

**Figure 1:**
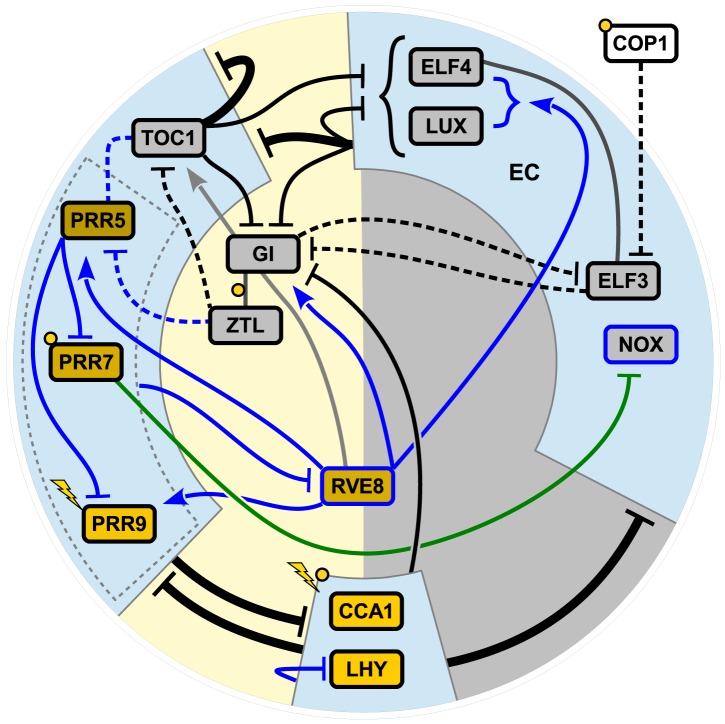
The F2014 model of the *Arabidopsis* circadian clock. Components of the clock are laid out according to approximate time of peak mRNA expression, clockwise with zeitgeber time 0 (lights on) at the bottom. Yellow and grey boxes indicate proteins that are active primarily during the day and night, respectively. Solid lines indicate transcriptional regulation and dashed lines indicate protein–protein interactions, with arrows for activation and bars for repression or degradation. Additions to the model relative to P2012 are shown in blue. The green line indicates a hypothetical interaction, and the light grey line indicates an interaction that the model predicts to be extremely weak. The light blue boxes show three main modules of the clock, and interactions between them are shown with thick black lines. EC is the evening complex between ELF3, ELF4 and LUX or NOX, and the dark grey line indicates the ELF3-ELF4 complex. Lightning and yellow circles symbolize light input at the transcriptional and post-transcriptional level, respectively. For an alternative version comparing F2014 with P2012 [6] (published 2013), see Figure S1.

Based on available experimental data and interpretations in the published literature, we have developed a revised model of the *Arabidopsis* circadian clock. The new model is presented in Figure 1, and a comparison with the most recently published model, P2012 [6], is shown in Figure S1. Five major alterations are discussed below: remodelling of EC, addition of the LUX homologue NOX, removal of sequential activation in the PRR wave, repression of the PRRs by CCA1, and addition of RVE8 as the main transcriptional activator. For brevity, we refer to Text S1 for further details and results concerning nuclear localization of TOC1 by PRR5, splitting of LHY/CCA1 and removal of unmotivated components and light inputs.

To increase the robustness of the conclusions drawn from the modelling, all our model simulations are presented as eight curves, derived from an ensemble of eight independent parameter sets as described in Methods.

### A remodelled evening complex

Overexpression of ELF3 rescues clock function in the otherwise arrythmic *elf4-1* mutant [27]. This suggests that the function of ELF4 is to amplify the effects of ELF3 through the ELF3-ELF4 complex, which led us to consider an evening complex (EC) where free ELF3 protein can play the role of ELF3-ELF4, albeit with highly reduced efficacy. This, together with our aim to add the NOX protein in parallel with LUX, as described in the next section, prompted us to rethink how to model this part of the clock.

EC is not given its own variable in the differential equations, unlike in the earlier models. Instead, EC activity is seen as rate-limited by LUX and NOX on one hand and by ELF3-ELF4 and free ELF3 on the other. In either pair, the first component is given higher importance, in accordance with previous knowledge. For details, see the equations in Text S1. This simplified description requires few parameters, which was desirable because the model had to be constrained using time course data for the individual components of EC, mainly at the mRNA level.

**Figure 2:**
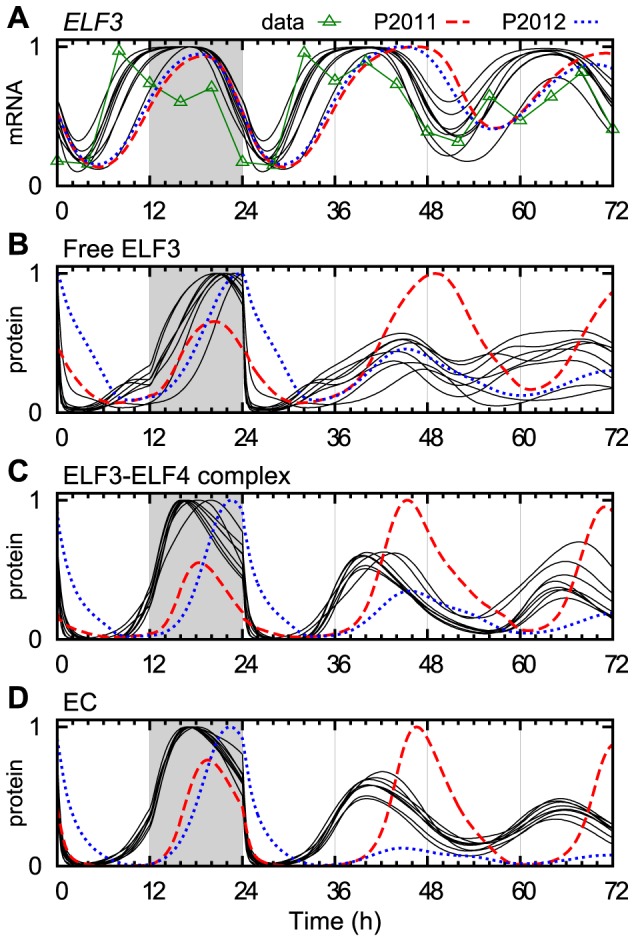
The evening complex and its components. Concentration levels of a selection of model components relevant to EC, in the transition from LD 12:12 (light/dark cycles) to LL (constant light), comparing our ensemble of models (eight parameter sets, black lines), to the previous models P2011 (dashed red line) and P2012 (dotted blue line). (A) *ELF3* mRNA in wild type (wt), compared with a typical experiment (green triangles, data from [51]). (B) ELF3 protein in the nucleus, not counting complexes. (C) The ELF3-ELF4 protein complex. (D) The resulting evening complex. Each curve was normalized to a peak level of 1. Grey background signifies the night of the last day of LD before the transition to LL at ZT 24.

The effects of our changes to EC are illustrated in Figure 2, which shows EC and related model components in the transition from cycles of 12 h light, 12 h dark (LD 12:12) to constant light (LL). ELF3, which is central to EC in our model, behaved quite differently at the mRNA level compared with the P2011 and P2012 models, and more closely resembled the available experimental data, with a broad nightly peak and a trough in the morning at zeitgeber time (ZT) 0–4 (Figure 2A).

The differences in the dynamics of the EC components between our eight parameter sets demonstrate an interesting and more general point: The components that are most reliably constrained are not always those that were fitted to measured data. In our case, the model was fitted to data for the amount of *ELF3* mRNA (Figure 2A) and total ELF3 protein (not shown), but the distribution between free ELF3 and ELF3 bound in the ELF3-ELF4 complex was not directly constrained by any data. As expected, the variation between parameter sets was indeed greater for the levels of free ELF3 protein and the ELF3-ELF4 complex, as shown in Figure 2B–C. However, the predicted level of EC (Figure 2D) showed less variation than even the experimentally constrained *ELF3* mRNA. This indicates that the shape and timing of EC were of such importance that the EC profile was, in effect, tightly constrained by data for the seven EC repression targets (*PRR9*, *PRR7*, *PRR5*, *TOC1*, *GI*, *LUX* and *ELF4*).

### NOX as a brother of LUX

NOX is a close homologue of LUX, with a highly similar DNA-binding domain and a similar expression pattern which peaks in the evening. Like LUX, NOX can form a complex with ELF3 and ELF4, but it is only partially redundant with LUX, which has a stronger clock phenotype [31]. The recruitment of ELF3 to the *PRR9* promoter is reduced in the *lux-4* mutant and abolished in the LUX/NOX double amiRNA line [32]. To explain these findings, we introduced NOX into the model as a component acting in parallel with LUX; we assumed that NOX and LUX play similar roles as transcriptional repressors in the evening complex.

There is evidence that NOX binds to the promoter of *CCA1* (and possibly *LHY*) *in vivo* and activates its transcription. Accordingly, the peak level of *CCA1* expression is higher when NOX is overexpressed, and the period of the clock is longer [30]. This possible role of NOX as an activator fits badly with its reported redundancy with LUX as a repressor. In an attempt to resolve this issue, we first modelled the system with NOX only acting as a repressor in EC, and then investigated the effects of adding the activation of *CCA1* expression.

**Figure 3:**
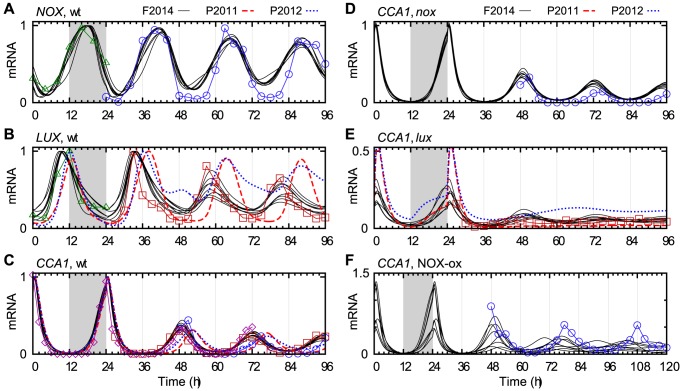
NOX and its interaction with CCA1. Comparison between the F2014 model (eight parameter sets, black lines) and experimental data (green triangles [31], blue circles [30], red squares [52] and purple diamonds [53]), and the earlier models P2011 (dashed red lines) and P2012 (dotted blue lines), where applicable, in the transition from LD to LL. (A) *NOX* mRNA in wt. (B) *LUX* mRNA in wt. (C–F) *CCA1* mRNA in (C) wt, (D) *nox* mutant (*boa-1*), (E) *lux* mutant (*pcl1-1*), and (F) NOX-ox. The peak mRNA levels for the models were normalized to 1 in wt, and the same normalization was kept for the mutants. Experimental data were scaled to match the model in panel C, and the same normalization was used in panels D–F. Note the different y scales.

Figure 3 illustrates the role of NOX in the model in comparison with LUX. The differences in their expression profiles (Figure 3A–B) reflect the differences in their transcriptional regulation (cf. Figure 1). *CCA1* expression is decreased only marginally in the *nox* mutant (Figure 3C–D) but more so in *lux* (Figure 3E). Because of the redundancy between NOX and LUX, the model predicted that the double mutant *lux*;*nox* has a stronger impact on circadian rhythms, with *CCA1* transcription cut at least in half compared with *lux* (Figure S2A). According to the model, the loss of LUX and NOX renders the evening complex completely ineffective, which in turn allows the *PRR* genes (including *TOC1*) to be expressed at high levels and thereby repress *LHY* and *CCA1*.

A comparison with the P2011 and P2012 models, which include LUX but not NOX, is shown in Figure 3B, C and E. Here, the most noticeable improvement in our model was the more accurate peak timing after entry into LL, where in the earlier models the clock phase was delayed during the first subjective night [33].

Period lengthening and increased *CCA1* expression was observed in NOX-ox only for some of the parameter sets (Figure 3F). The four parameter sets with increased *CCA1* all had a very weakly repressing NOX whose main effect was to counter LUX by taking its place in EC. Removing NOX from EC in the equations and reoptimizing a relevant subset of the parameters worsened the fit to the data (Figure S3). These results support the idea of NOX acting through EC in manner that makes it only partially redundant with LUX.

The possibility that NOX is a transcriptional activator of *CCA1* and *LHY* was probed by adding an activating term to the equations (see Text S1) and reoptimizing the parameters that control transcription of *CCA1* and *LHY*. The resulting activation was very weak in all parameter sets, and had negligible effect on the expression of *CCA1* in NOX-ox (Figure S2B–C). Accordingly, the addition of the activation term did not improve the fit to data as measured by the cost function described in Methods (Figure S3).

### Sequential PRR expression without activation

In earlier models that included the PRR genes, the PRRs were described as a series of activators; during the day, PRR9 activated the transcription of *PRR7*, which similarly activated *PRR5*. These interactions improved the clock's entrainability to different LD cycles [8]. However, this sequential activation disagrees with experimental data for *prr* knockout mutants, which indicate that loss of function of one PRR leaves the following PRR virtually unaffected. For instance, experiments have shown that the expression levels of *PRR5* and *TOC1* (as well as *LHY* and *CCA1*) are unaffected in both *prr9-1* and *prr7-3* knockout mutants [11], [34].

Instead, direct interactions between the PRRs have been found to be negative and directed from the later PRRs in the sequence to the earlier ones [15], [35]. A strong case has been made for TOC1 as a repressor of the PRR genes [9], [14]. As in P2012, we modelled transcription of *PRR9*, *PRR7* and *PRR5* as repressed by TOC1, but we also included negative auto-regulation of TOC1, as suggested by the ChIP-seq data that identified the TOC1 target genes [14]. Likewise, PRR5 directly represses expression of *PRR9* and *PRR7* [35], and we have added these interactions to the model.

**Figure 4:**
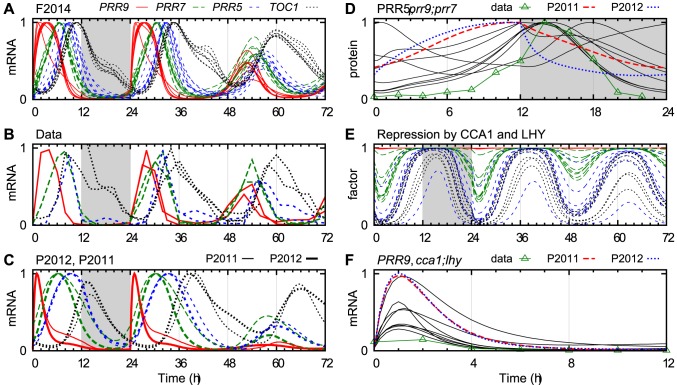
Expression and regulation of the PRR genes. (A–C) The mRNA levels of *PRR9* (solid red), *PRR7* (long dashed green), *PRR5* (short dashed blue) and *TOC1* (dotted black) in the transition from LD to LL. (A) The F2014 model with eight different parameter sets. (B) Experimental data: *PRR9* [35], [36], [54], *PRR7* [35], [54], [55], *PRR5* [29], [55], [56] and *TOC1* [53], [57], [58]. (C) The P2012 and P2011 models (thick and thin lines, respectively). (D) Total PRR5 protein level in *prr9;prr7* in LD in F2014 (solid black), P2011 (dashed red), P2012 (dotted blue) and experimental data (green triangles [54]). (E) The predicted repression of *PRR* transcription by CCA1 and LHY, as a multiplicative factor, with colours as in (A–C). (F) *PRR9* mRNA in *cca1-11*;*lhy-21* in LD, normalized to the corresponding wt curves in (A–C); colours as in (D) but data from [11]. The peak levels in (A), (C) and (D) were normalized to 1, whereas the levels in (B) were adjusted manually.

As illustrated in Figure 4A–C, this reformulation of the PRR wave is compatible with correct timing of the expression of the PRRs in the wild type, and the timing and shape of the expression curves were improved compared with the P2012 model. An earlier version of our model gave similar profiles despite missing the repression by PRR5, which suggests that such repression is not of great importance to the clock.

A nightly repressor appears to be acting on the *PRR7* promoter, as seen in the rhythmic expression of *PRR7* in LD in the *cca1-11*;*lhy-21*;*toc1-21* mutant [36]. An observed increase in *PRR7* expression at ZT 0 in the *lux-1* mutant relative to wild type [29] points to EC as a possible candidate. Although Helfer *et al*. report that LUX does not bind to the LUX binding site motif found in the *PRR7* promoter [31], we included EC among the repressors of *PRR7*. This interaction was confirmed by Mizuno *et al*. while this manuscript was in review [37], demonstrating the power of modelling and of timely publication of models.

We further let EC repress *PRR5*. We are not aware of any evidence for such a connection, but the parameter fitting consistently assigned a high value to the connection strength, as was also the case with *PRR7*. This result hints that nightly repression of *PRR5* is of importance, whether it is caused by EC or some related clock component.

The real test of the model came with knocking out members of the PRR wave. Here, the model generally outperformed the P2012 model, as judged by eye, but we are missing data for some important experiments such as PRR7 in *prr9*. As an example, Figure 4D shows the level of PRR5 protein in the *prr9*;*prr7* double mutant, where half of our parameter sets predict the correct profile and peak phase. In the earlier models, the only remaining inputs to PRR5 were 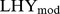 (a hypothetical delayed LHY/CCA1), TOC1 (in P2012 only) and light (which stabilized the protein), and these were unable to shape the PRR5 profile correctly. The crucial difference in our model was the repression of *PRR5* by CCA1 and LHY, as described in the next section.

### Regulation of the PRRs by CCA1 and LHY

CCA1 and LHY appear to work as transcriptional repressors in most contexts in the clock (see e.g. [38]), but knockdown and overexpression experiments seem to suggest that they act as activators of *PRR9* and *PRR7* [34]. Accordingly, previous models have used activation by LHY/CCA1, combined with an acute light response, to accomplish the rapid increase observed in *PRR9* mRNA in the morning. However, with the misinterpretation of TOC1 regulation of CCA1 [12] in mind, we were reluctant to assume that the activation is a direct effect.

To investigate this issue, we modelled the clock with CCA1 and LHY acting as repressors of all four PRRs. If repression was incompatible with the data for any of the PRRs, parameter fitting should reduce the strength of that repression term to near zero. As is shown in Figure 4E, the model consistently made CCA1 and LHY strongly repress *PRR5* and *TOC1*. *PRR7* was also repressed, but in a narrower time window that acted to modulate the phase of its expression peak. In contrast, *PRR9* was virtually unaffected; CCA1 and LHY do not directly repress *PRR9* in the model.

Even though CCA1 and LHY were not modelled as activators, the model reproduced the reduction in *PRR9* expression observed in the *cca1-11*;*lhy-21* double mutant (Figure 4F and Figure S4). *PRR7* behaved similarly to *PRR9* in both experiments and model. Conversely, in the P2011 and P2012 models, where LHY/CCA1 was supposed to activate *PRR9*, there was no reduction in the peak level of *PRR9* mRNA in *cca1*;*lhy* compared to wild type (Figure S5A).

To explore whether CCA1 and LHY may be activating *PRR9* transcription, we temporarily added an activation term to the equations (see Text S1) and reoptimized the relevant model parameters. The activation term came to increase *PRR9* expression around ZT 2 at least twofold in two of the eight parameter sets, and by a smaller amount in several (Figure S5B). This would seem to suggest that activation improved the fit between data and model. Surprisingly, there was no improvement as measured by the cost function (Figure S3). With the added activation, *PRR9* was reduced only marginally more in *cca1*;*lhy* than in the original model (Figure S5C). A likely explanation is that feedbacks through EC and TOC1, which repress *PRR9*, almost completely negate the removed activation of *PRR9* in the *cca1*;*lhy* mutant. Thus the model neither requires nor rules out activation of *PRR9* by CCA1 and LHY.

### Transcriptional activation by RVE8

Like CCA1 and LHY, RVE8 is a morning expressed MYB-domain transcription factor. However, unlike CCA1 and LHY, RVE8 functions as an activator of genes with the evening element motif, and its peak activity in the afternoon is strongly delayed in relation to its expression [28]. Based on experimentally identified targets, we introduced RVE8 into our model as an activator of the five evening expressed clock components *PRR5*, *TOC1*, *GI*, *LUX* and *ELF4*, as well as the morning expressed *PRR9* [29].

**Figure 5:**
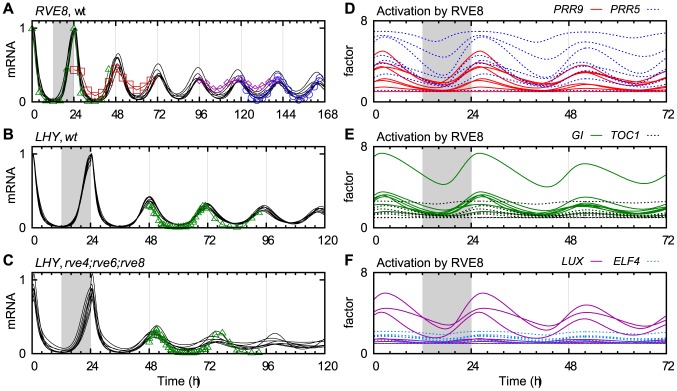
The effects of RVE8 in the model. (A–C) Expression levels in the transition from LD to LL, comparing the model (eight parameter sets, solid black lines) with experimental data (green triangles [29], red squares [59], blue circles [28] and purple diamonds [60]). (A) *RVE8* mRNA in wt, (B) *LHY* in wt, and (C) *LHY* in *rve4*;*rve6*;*rve8*. (D–F) The effect of RVE8 on each of its target genes, as a time-dependent multiplicative factor, in the eight parameter sets. (D) *PRR9* (solid red) and *PRR5* (dotted blue), (E) *GI* (solid green) and *TOC1* (dotted black), and (F) *LUX* (solid purple) and *ELF4* (dotted light blue).

PRR5 binds directly to the promoter of *RVE8* to repress its transcription [35], and it is likely that PRR7 and PRR9 share this function [28], [29]. Using only these three PRRs as repressors of *RVE8* was sufficient to capture the expression profile and timing of RVE8, both in LL and LD (Figure 5A).

RVE8 is partially redundant with RVE4 and RVE6 [28], which led us to model the *rve8* mutant as a 60% reduction in the production of RVE8. To clearly see the effects of RVE8 in the model, we instead compared with the *rve4*;*rve6*;*rve8* triple mutant, which we modelled as a total knockout of RVE8 function. The phase of the clock was delayed in LD, and the period lengthened by approximately two hours in LL in the simulated triple mutant, in agreement with with data for LHY (Figure 5B–C), though we note that CAB::LUC showed a greater period lengthening in experiments [29].

To investigate the significance of RVE8 as an activator in the model, we made a version of the model without RVE8. The model parameters were reoptimized against the time course data (excluding data for RVE8 and from *rve* mutants). As with NOX, we found that removing the activation had no clear effect on the costs of the parameter sets after refitting (Figure S3). It appears that activators such as RVE8 are not necessary for clock function. Still, the effects of the *rve* mutants can only be explained when RVE8 is present in the model, motivating its inclusion.

The model used RVE8 as an activator for four of its targets in a majority of the parameter sets (Figure 5D–F). The exceptions were *TOC1* and *ELF4*. Although *TOC1* is a binding target of RVE8 *in vivo*, *TOC1* expression is not strongly affected by *RVE8-ox* or *rve8-1* [28], [39]. This was confirmed by our model, where the parameter fitting disfavoured the activation of *TOC1* in most of the parameter sets (Figure 5E). The eight parameter sets may not represent an exhaustive exploration of the parameter space, but the results nevertheless support the notion that the effect of RVE8 on *TOC1* is of marginal importance.

## Methods

As with previous models of the *Arabidopsis* clock, our model consists of a set of ordinary differential equations (ODEs) with parameters that need to be fitted against experimental observations. The final F2014 model consists of equations for 35 variables, with a total of 119 parameters. The number of variables has increased compared with previous models (see Table 1), but the number of parameters has been reduced relative to P2012, due to the simplifications described in Results and Text S1.

**Table 1:** The number of parameters and variables in different *Arabidopsis* clock models.

Model	Parameters	Variables
L2006 [7]	60 (+8)	16
P2010 [8]	80 (+17)	19
P2011 [9]	107 (+6)	28
P2012 [6]	123 (+10)	28 (+4)
F2014	119 (−)	35

Parameter counts in parentheses refer to constant integer Hill coefficients, which are written explicitly into the F2014 equations. Variables in parentheses for P2012 refer to ABA related variables.

### Data collection

Constraining the many parameters in our model requires a cost function based on a large number of experiments. To this end, we compiled time course data from the published literature, mainly by digitizing data points from figures using the free software package g3data [40]. We extracted more than 11000 data points from 800 time courses in 150 different mutants or light conditions, from 59 different papers published between 1998 and 2013. The median time resolution was 3 hours. The list of time courses and publications can be found in Text S2, and the raw time course data and parameter values are available for download from http://cbbp.thep.lu.se/activities/clocksim.

Most of the compiled data refer to the mRNA level, from measurements using Northern blots or qPCR, but there are also data at the protein level (67 time courses) and measurements of gene expression using luciferase assays (12 time courses). About one third of the time courses can be considered as replicates, mainly from wild type plants in the most common light conditions. Many of these data are controls for different mutants. Where wild type and mutant data were plotted with the same normalization, we made note of this, as their relative levels provide crucial information that is lost if the curves are individually normalized.

### Model fitting and constraining

To find suitable values for the model parameters, we constructed a minimalistic cost function based on the mean squared error between simulations and time course data. This approach was chosen to allow the model to capture as many features of the gene expression profiles as possible, with a minimum of human input.

The cost function consists of two parts, corresponding to the profiles and levels of the time course data, respectively. For each time course 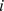 with 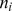 experimental data points 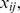 the corresponding simulated data 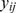 were obtained from the model. The simulations were performed with the mutant background represented in the model equations, with entrainment for up to 50 days in light/dark cycles followed by measurements, all in the experimental light conditions. The cost for the concentration profile was computed as
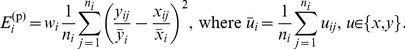
(1)


Since the profile levels are thus normalized, eq. (1) is independent of the units of measurements. The parameters 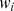 (see Text S2 for values) allowed us to weight time courses to reflect their relative importance, e.g. where less data was available to constrain some part of the model.

Where several experimental time courses had the same normalization, e.g. in comparisons between wild type and mutants, the model should reproduce the relative changes in expression levels between the time courses. For each group of time courses, 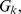 we could minimize the sum
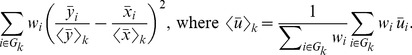
(2)


Unlike eq. (1), the nominators in this sum are guaranteed to be non-zero, which allows us to operate in log-space where fold changes up or down from the mean will be equally penalized. Replacing 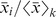 with 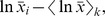 and likewise for 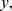 we write the final scaling cost for group 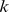 as
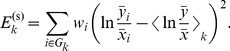
(3)


This cost term thus penalizes non-uniform scaling between experiment and data within the group.

The total cost to minimize was
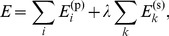
(4)


where 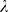 sets the balance between fitting the simulation to the profile or the level of the data. We used 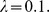


A downside to our approach is that period and phase differences between different data sets result in fitting to a mean behaviour that is more damped than any individual data set. To reduce this problem, we removed the most obvious outliers from the fitting procedure. We also considered distorting the time axis (e.g. dynamic time warping) to normalize the period of oscillations in constant conditions, in order to better capture the effects of mutants relative to the wild type. This process would be cumbersome and arbitrary, which is why it was deemed outside the scope of our efforts.

Compared to previous models by Pokhilko *et al.*, fewer parameters were manually constrained in our model. In the P2010–P2012 models, roughly 40% of the parameters were constrained based on the experimental data [6], [8], [9], and the remaining free parameters were fitted to mRNA profiles in LD and the free running period in LL and DD (constant dark) in wild type and mutants [9]. For the F2014 model, we completely constrained 16 parameters in order to obtain correct dynamics for parts of the system where we lacked sufficient time course data. Specifically, the parameters governing COP1 were taken from P2011 where they were introduced, whereas the parameters for the ZTL and GI proteins (except the GI production and transport rates) were fitted by hand to the figures in [41]. All other parameters were fitted to the collected time course data through the cost function.

The eight parameter sets presented here were selected from a group of 30, where each was independently seeded from the best of 1000 random points in parameter space, then optimized using parallel tempering for 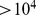 iterations at four different temperatures which were gradually lowered. The resulting parameter values, which are listed in Text S1, typically span at least an order of magnitude between the different parameter sets (Figure S6). The sensitivity of the cost function to parameter perturbations is presented in Figure S7 and further discussed in Text S1. Plots of the single best parameter set against all experimental data is shown in Figure S8.

To simulate the system and evaluate the cost function rapidly enough for parameter optimization to be feasible, we developed a C++ program that implements ODE integration and parameter optimization using the GNU Scientific Library [42]. Evaluating the cost function for a single point in parameter space, against the full set of experiments and data, took about 10 seconds on a 3 GHz Intel Core i7 processor. Our software is released under the GNU General Public License (GPL) [43] and is available from http://cbbp.thep.lu.se/activities/clocksim/.

## Discussion

### Modelling and data

Accurately modelling the circadian clock as a network of a dozen or more genes is challenging. Previous modelling work (e.g. P2010–P2012) [6], [8], [9] has drawn on existing data and knowledge to constrain the models, but as the amount of data increases it becomes ever more difficult to keep track of the effects of mutations and other perturbations. For a system as large as the plant circadian clock, it is desirable to automate the parameter search as much as possible, but encoding the uncertainties surrounding experimental data in a computer-evaluated cost function is not trivial.

Our modelling demonstrates the feasibility of fitting a model of an oscillating system against a large set of data without the construction of a complicated cost function based on qualitative aspects of the model output, such as entrainability, free-running period or amplitude. Instead, we relied on the large amount of compiled time course data to constrain the model, using a direct comparison between simulations and data. This minimalistic cost function had the additional advantage of allowing the use of time courses that span a transition in environmental conditions, e.g. from rhythmic to constant light, where the transient behaviour of the system may contain valuable information. Consequently, our model correctly reproduces the phase of the clock after such transitions (see e.g. Figure 3C).

Our approach makes it easy to add new data, at the price of ignoring previous knowledge (e.g., clock period) from reporters that are not represented in the model. Accordingly, our primary modelling goal was not to reproduce the correct periods of different clock mutants, but rather to capture the profiles of mRNA and protein curves, and the changes in amplitude and profile between mutants and different light conditions. Compiling a large amount of data from different sources has allowed us to see patterns in expression profiles that were not apparent without independent replication. For example, the *TOC1* mRNA profile shows a secondary peak during the night in many data sets (see examples in Figure 4B).

All collected time course data were used in fitting the parameters. To validate the model, we instead used independently obtained period data from clock period mutants. The results are shown in Text S1. In brief, most predictions in LL are in good agreement with experiments, with the exception of *elf4* where the period changes in the wrong direction.

To experimentally measure a specific parameter value, such as the nuclear translocation rate of a protein, is exceptionally challenging. Hence, constraining a model with measured parameters can introduce large uncertainties in the model predictions, especially when the understanding of the full system is incomplete. Fitting the model with free parameters can instead give a large spread in individual parameter values, but result in a set of models that make well constrained predictions. For this reason, we have based our results on an ensemble of independently optimized parameter sets, as recommended by Gutenkunst *et al.* [44]. At the cost of computational time, this approach gives a more accurate picture of the uncertainties in the model and its predictions, rather than focusing on individual parameter values.

Based on our experience of curation of time course data, we offer some suggestions for how data can be compiled and treated to be more useful to modellers. These points arose in the context of the circadian clock, but they apply to experiments that are to be used for modelling in a broader context.

- If the raw data contain information about the relative levels between experiments, for example between mutant and wild type, do not discard this information by normalizing the peak levels of the curves individually.
- If possible, provide data from both before and after treatment, preferably as one uninterrupted time course, so that changes in expression levels become clear. In clock experiments, this would entail including data from the last day of entrainment before a shift into constant light.
- Increase the time resolution of measurements where expression levels are expected to change rapidly, as this adds valuable information about timing. This is especially important around light/dark transitions to distinguish between acute light responses and circadian rhythms.
- Be clear about the conditions during entrainment, especially if they were varied between experiments.
- If possible, apply background correction so that the data reflect the true ratio between peak and trough levels. Alternatively, be clear about whether background correction has been applied.
- Use supplementary figures or files to present data that were not included in the figures and that would otherwise be lost to the research community.

Two of these suggestions concern the preservation of information about the relative expression levels between experiments. One example of the value of such information comes from the dramatic reduction in *PRR9* expression in *cca1*;*lhy* (Figure 4F). As implied in the section on *PRR9* activation in Results, clock models ought to be able to explain both shape and level of expression curves in such mutant experiments, but this is only possible if that information is present in the data.

### RVE8 as an activator

Based on the current knowledge of the clock, most clock components are exclusively or primarily repressive, and RVE8 sets itself apart by functioning mainly (or solely) as an activator. According to our model, RVE8 has only a marginal effect on the expression of *TOC1*, but activates *PRR5* and other genes more strongly, in agreement with earlier interpretations of the experimental data [29].

We note that all six targets of RVE8 in the model (*PRR9*, *PRR5*, *TOC1*, *GI*, *LUX* and *ELF4*) are also binding targets of TOC1 [14]. This may be a coincidence, because TOC1 is a repressor of a majority of the genes in the model. It is conceivable, however, that activation by RVE8 around noon is gated by TOC1 to confer sensitivity to the timing of RVE8 relative to TOC1 in a controlled fashion.

We were surprised by the ease with which we could remove RVE8 from the model. After reoptimization of the parameters, the cost was decreased in three of the eight parameter sets compared with the original model (Figure S3). Thus, the clock is not dependent on activation for its function (although it should be noted that the model without RVE8 lost the ability to explain any RVE8-related experiments). This result indicates that the model possesses a high degree of flexibility, whereby the remaining components and parameters are able to adjust and restore the behaviour of the system. Such flexibility challenges our ability to test hypotheses about individual interactions in the model, but we argue that predictions can also be made based on entropy.

Even if an alteration to the model, such as the addition of RVE8, does not result in a significant change in the cost function, it may open up new parts of the high-dimensional parameter space. If, following local optimization, most parameter sets indicate that a certain interaction is activating, we may conclude that the activation is likely to be true. The parameter space is sampled in accordance with the prior belief that the model should roughly minimize the cost function, and the same reasoning motivates the use an ensemble of parameter sets to explore the model. The conclusion about activation is indeed strengthened by the use of multiple parameter sets, because we learn whether it is valid in different areas of the parameter space.

### Problems and predictions

Our model agrees with a majority of the compiled data sets, but like earlier models it also fails to fit to data for some mutants. This indicates that important clock components or interactions may yet be unknown or misinterpreted. We here give a few examples.

*NOX* expression is rhythmic in the short period double mutant *cca1*;*lhy* [30], but our model predicts a constant high NOX level in constant light (Figure S4F). If NOX is repressed by PRR7 as assumed in the model (see Text S1), the rhythmicity can only be explained if PRR7 is also rhythmic and drives the NOX oscillations. Unfortunately, the model predicts that PRR7 oscillates only for a single cycle in *cca1*;*lhy*, before going to a constant low level (Figure S4B). This is a prediction shared with the P2012 model; we are not aware of any data that invalidate the prediction, but given that PRR7 is only slightly reduced in *cca1*;*lhy* in light/dark cycles [36], we believe that PRR7 may be rhythmic in constant light in this mutant.

The addition of NOX as a component partly redundant with LUX leads to an untested prediction regarding *CCA1* and *LHY*. Their peak expression levels are reduced only marginally in *nox* but roughly by half in *lux* compated with wt. In the *lux;nox* double mutant, the model predicts that their expression is cut by at least half again, to nearly zero even in light/dark cycles (see Figure 3 and Figure S2).

The modelling suggests that nightly repression of *PRR5* and *PRR7* is of importance. The evening complex (EC) is thought to repress *PRR9* and *TOC1*, and our prediction that EC also represses *PRR7* was experimentally confirmed while this manuscript was in review [37].

Several known clock components were not included in the model, partly due to a lack of suitable data. Examples of genes that could be included in future models are *CHE* [45] and *EBI* [46]. More experiments and data are also needed to clarify the differences between CCA1 and LHY, the role of NOX as a part of the evening complex, and how PRR5 affects the localization of TOC1.

Additional non-transcriptional interactions should also be considered in future work. This includes protein interactions such as the regulation of LHY degradation by DET1 [47], [48]. Most importantly, the recently discovered and highly conserved redox-related circadian oscillator is linked to the transcriptional clock [49], [50]. Understanding that link may help explain why some clock components more easily remain rhythmic in experiments than in simulations.

### The complexity of the clock

The insensitivity of *PRR9* to LHY/CCA1 in the P2011–P2012 models, as illustrated by its unchanged level in the *cca1*;*lhy* mutant (Figure S5A), shows one of the problems of constructing and fitting large models: The transcriptional activation of *PRR9* by LHY/CCA1 looks like an important term in the model equations, but the effects of this term are small. To reduce the prevalence of such “dead” terms and parameters in the equations, we recommend examining their effects in isolation, as was done with the corresponding repression terms in Figure 4E.

The ability of our model to reduce *PRR9* expression in *cca1*;*lhy* (Figure 4F) can only be explained by indirect effects. CCA1 and LHY repress *TOC1*, which in turn represses *PRR7* and *PRR9*, and the resulting indirect activation may be sufficient to counteract the direct repression by CCA1 and LHY. In general, in a highly interconnected system such as the circadian clock, it is perilous to draw conclusions about whether interactions are activating or repressing based only on altered expression levels in mutants.

Previous models (L2006–P2012) described the *Arabidopsis* circadian clock as primarily divided into two interacting feedback loops, the “morning loop” and the “evening loop”. In contrast, we describe the clock in terms of three main modules linked by transcriptional repression and many additional connections (Figure 1). Our results and experiences support an important point formulated by Hsu *et al.* [29]: The plant clock is best viewed as a highly interconnected, complex regulatory network, in which discrete feedback loops are virtually impossible to identify.

## Supporting Information

Figure S1 **Model comparison.** An alternative representation of the F2014 model (bottom), allowing easier comparison with the P2012 model (top), adapted from [6]. Symbols as in Figure 1.
(EPS)

Figure S2 **NOX interaction with CCA1.** (A) The predicted *CCA1* expression level in the *lux;nox* double mutant, in the transition from LD to LL in F2014. The peak levels were normalized to 1 in wt, as in Figure 3. (B) The activation of *CCA1* expression by NOX in a variant of the model, expressed as a multiplicative factor. (C) *CCA1* mRNA in NOX-ox in same model variant as (B), shown as in Figure 3F.
(EPS)

Figure S3 **Cost function values.** The value of the cost function for the eight best parameter sets in the six different model variants discussed in the text. Note that all parameters were reoptimized in the model without RVE8, whereas only a subset of the parameters were reoptimized in the variants with CCA1 activating *PRR9* or with different NOX function. Furthermore, the original model improved somewhat as it was optimized in parallel with the other variants.
(EPS)

Figure S4 ***PRR7*** **,** ***PPR9*** **and** ***NOX*** **mRNA in wt and** ***cca1;lhy*** **.** Comparison between our model (solid black lines), P2011 (dashed red lines), P2012 (dotted blue lines) and data (green triangles) between wt (left panels) and *cca1;lhy* (right panels), in the transition from LD to LL. (A–B) *PPR7*, (C–D) *PPR9*, and (E–F) *NOX*. Data from [54] (A–D) and [30] (E–F). Peak levels were normalized to 1 in wt.
(EPS)

Figure S5 **Effects of activation of** ***PRR9*** **by CCA1.** (A) *PRR9* mRNA in P2011 (dashed red) and P2012 (dotted blue) in wt (thin lines, higher) and *cca1;lhy* (thick lines, lower), in the transition from LD to LL. Activation by LHY/CCA1 affects the expression of *PRR9* in the afternoon, but the peak level is unaffected in the double mutant. (B) The activation of *PRR9* by CCA1, after refitting our model with such an activation term. The activation is shown as a multiplicative factor, whose peak is >1.2 for half of the eight parameter sets. (C) Expression of *PRR9* in *cca1;lhy* in the day (LD or first day of LL), in the model where CCA1 activates *PRR9* transcription, with peak levels normalized to 1 in wt. The difference between the model (black lines) and data (green triangles [54]) is comparable to the difference without the activation term (Figure 4F).
(EPS)

Figure S6 **Parameter variability between parameter sets.** A visual representation of the values of the model parameters in the eight best parameter sets (eight different symbols). These values are also presented as a table in Text S1.
(PDF)

Figure S7 **Parameter sensitivity analysis.** The relative change in cost function in each of the eight best parameter sets (eight different symbols) when each parameter is altered. Symbols above (below) the zero cost line refer to multiplication (division) of the parameter by 1.1.
(PDF)

Figure S8
**Model simulations compared with all data.** Simulations with the single best parameter set, plotted against all 800 time courses used for fitting the model. As described in Methods, simulations and data are normalized to the same mean. Time courses with identical normalization are shown on the same page (“Scale group 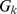), with the total scaling cost in the title. The profile and scaling costs (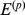 and 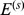) for each individual time course are shown in the legend. The time courses are named after the data files used; these are available for download as described in Methods. The naming convention is as follows: initial letters denote light condition, dd (constant dark), ll (constant light), rr (constant red light), bb (constant blue light), ld (light dark LD 12:12), lgd (long day LD 16:8), and shd (short day LD 8:16); followed by gene name, C (CCA1), L (LHY), T or P1 (TOC1), G (GI), P5,7,9 (PRR5,7,9), LUX (LUX), NOX (NOX), R8 (RVE8), E3 (ELF3), E4 (ELF4), Z (ZTL); suffixed by “_m” for mRNA data and an arbitrary number for uniqueness, or just the number for protein data. The last part of the filename is “-ox” for overexpression, and/or lower case gene names for mutants. A combination of LL and another light condition indicates entrainment in something other than LD 12:12, followed by LL. Where all data come from the same light conditions, the background is shaded for night; exceptions include scaling groups with data from different photoperiods.(PDF)

Text S1
**Additional results and equations.** Further information about the modelling, covering details about the evening complex, the regulation of NOX, the splitting of CCA1 and LHY into two variables, the localization of TOC1 and PRR5, and the removal of the ABA circuit, 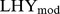 and some light inputs. This text also includes the differential equations of the model, a table of periods comparing model to experiments, and the parameter values of the eight best fitted parameter sets. The equations are presented in their wild type forms, which do not include modifications used when simulating the many different mutants.(PDF)

Text S2 **Overview of the compiled time course data.** A list of the roughly 800 experimental data sets that were compiled and used for fitting the model.
(PDF)
