# Supplementary figures and images for: Rethinking Transcriptional Activation in the Arabidopsis Circadian Clock
Source: PLoS Comput Biol. 2014 Jul 17;10(7):e1003705. doi: 10.1371/journal.pcbi.1003705 (PMC4102396; doi:10.1371/journal.pcbi.1003705)

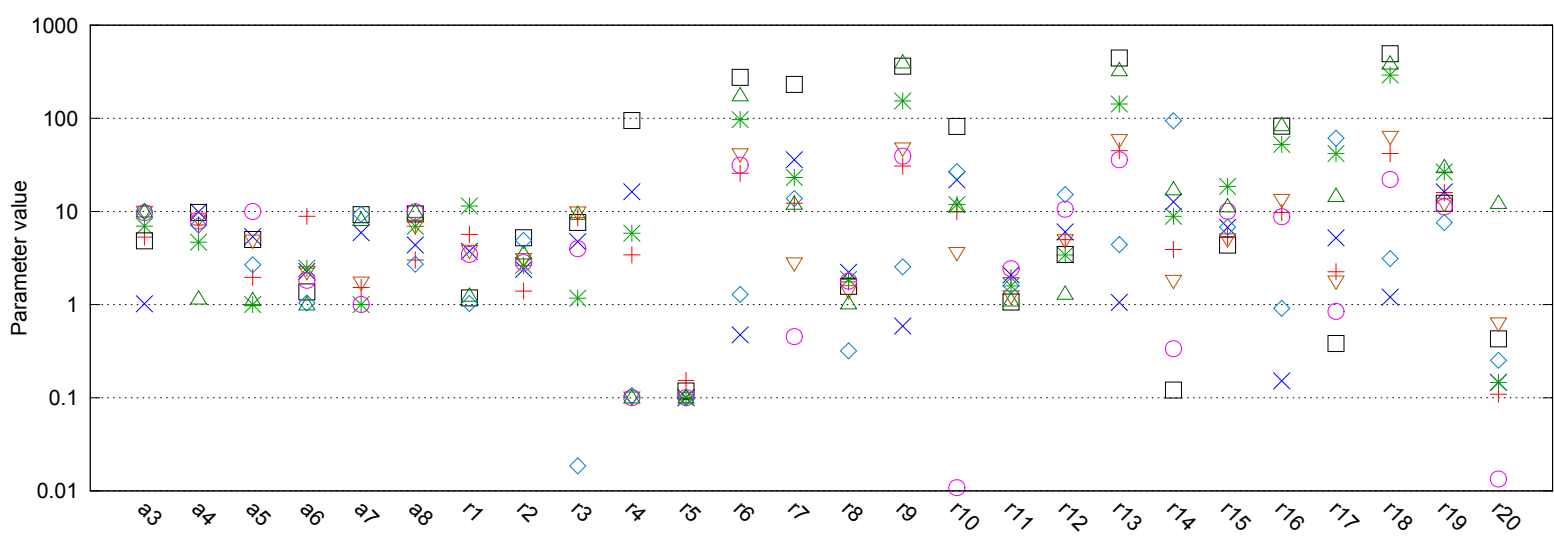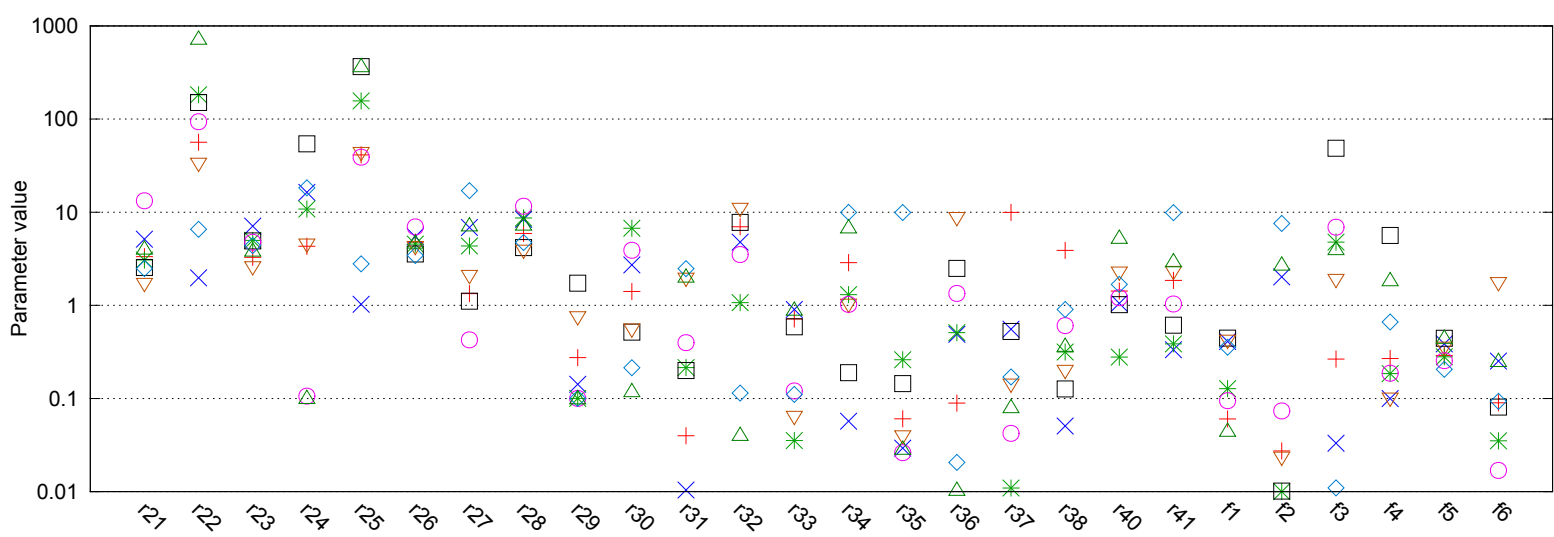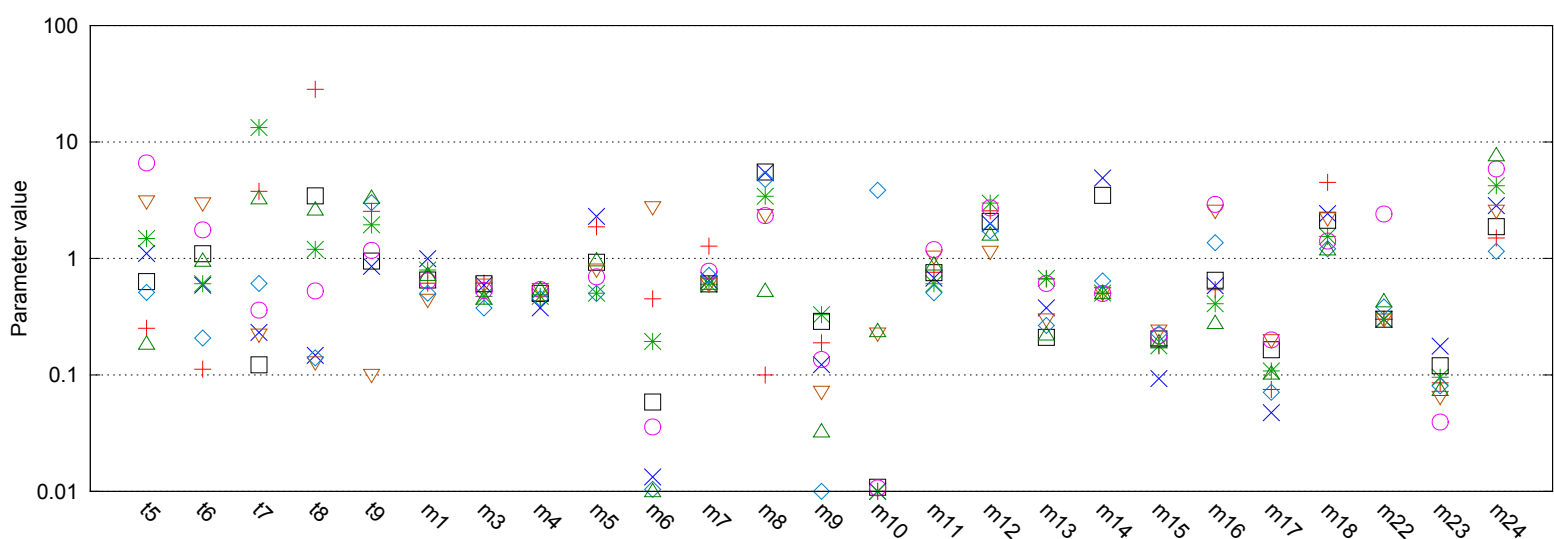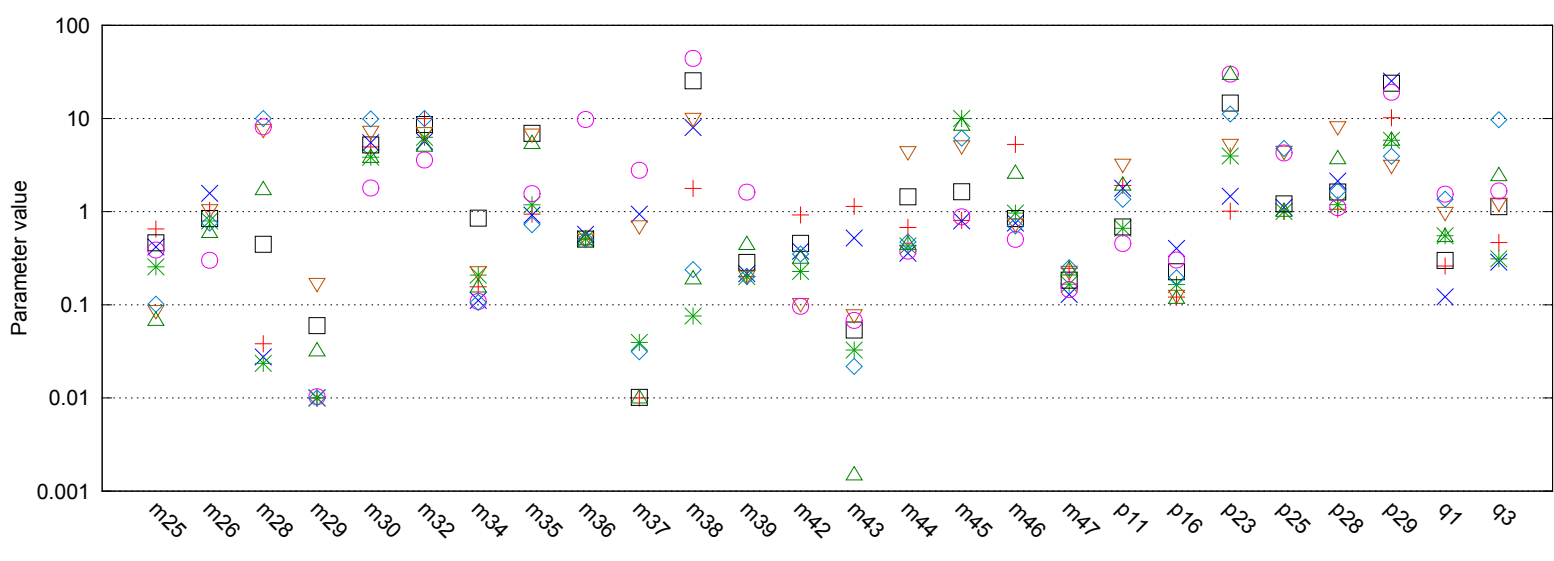

Supplement: Figure S6 — Parameter variability between parameter sets. A visual representation of the values of the model parameters in the eight best parameter sets (eight different symbols). These values are also presented as a table in Text S1. (PDF) [file pcbi.1003705.s006.pdf]

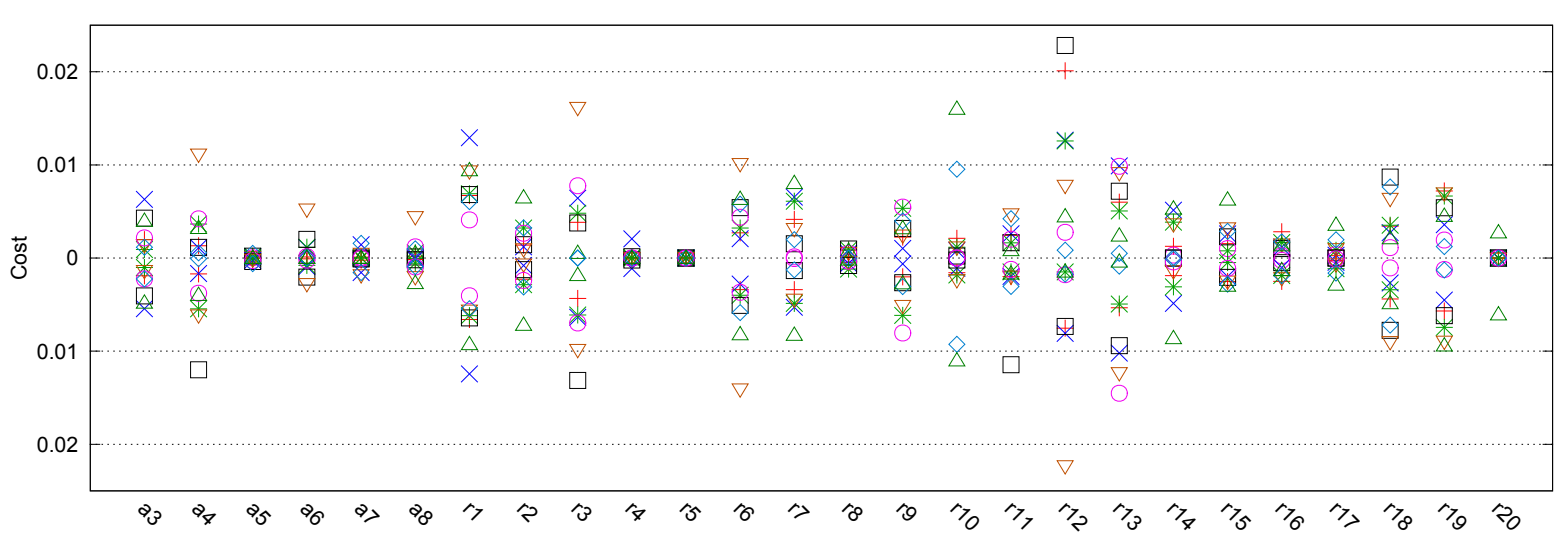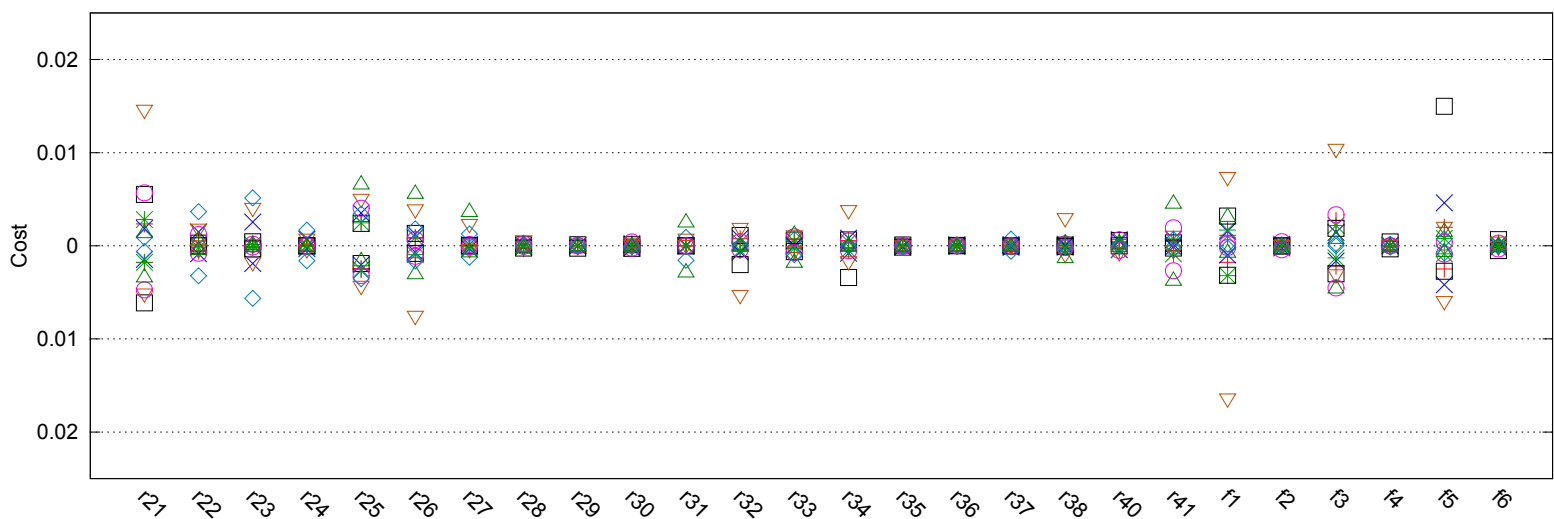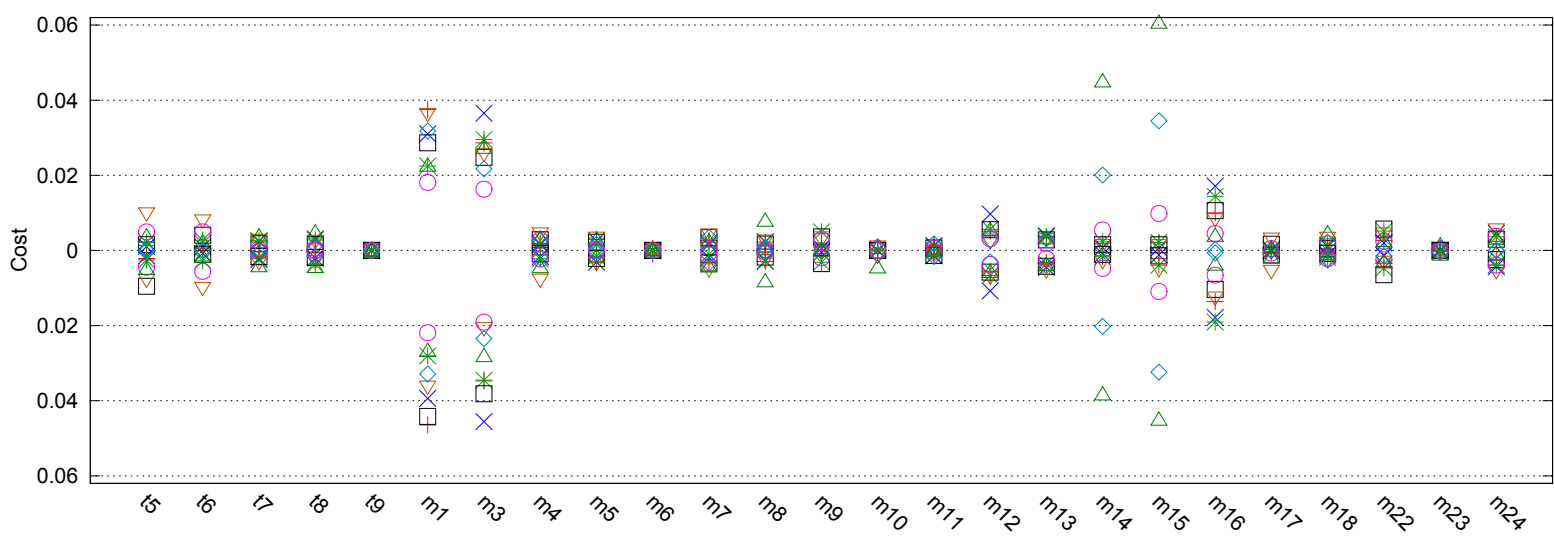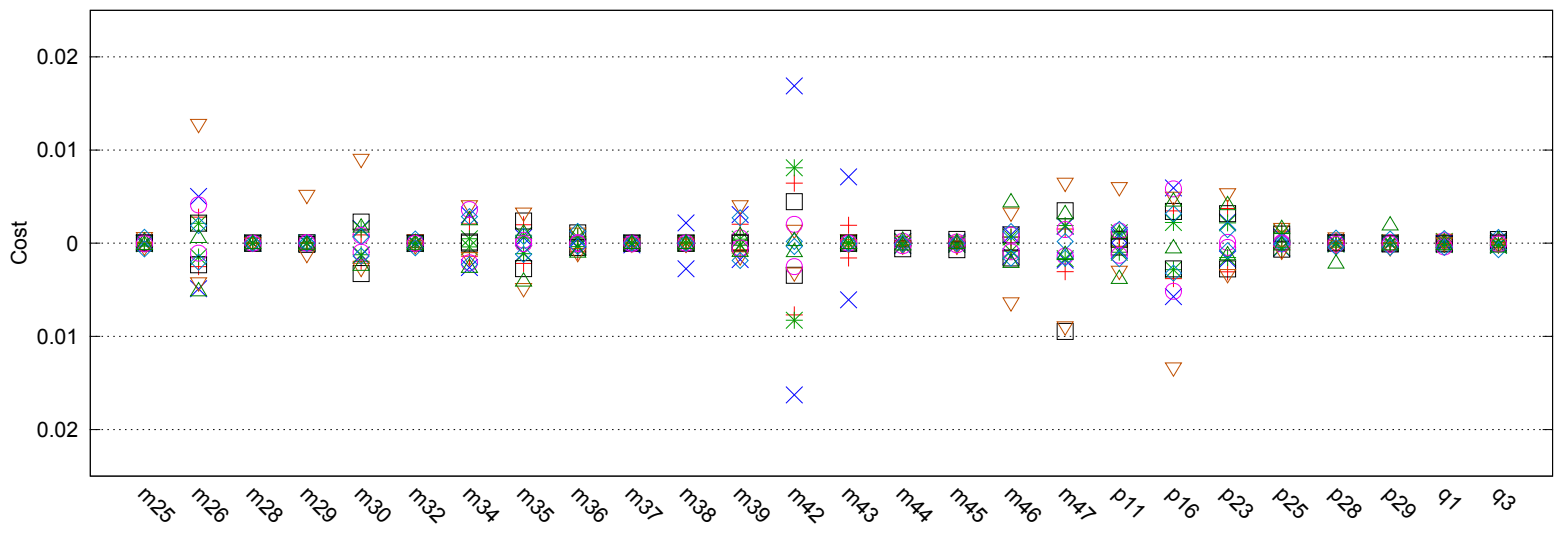

Supplement: Figure S7 — Parameter sensitivity analysis. The relative change in cost function in each of the eight best parameter sets (eight different symbols) when each parameter is altered. Symbols above (below) the zero cost line refer to multiplication (division) of the parameter by 1.1. (PDF) [file pcbi.1003705.s007.pdf]
