# Supplementary material for: Rethinking Transcriptional Activation in the Arabidopsis Circadian Clock
Source: PLoS Comput Biol. 2014 Jul 17;10(7):e1003705. doi: 10.1371/journal.pcbi.1003705 (PMC4102396; doi:10.1371/journal.pcbi.1003705)
Supplement: Figure S8 — Model simulations compared with all data. Simulations with the single best parameter set, plotted against all 800 time courses used for fitting the model. As described in Methods, simulations and data are normalized to the same mean. Time courses with identical normalization are shown on the same page (“Scale group ), with the total scaling cost in the title. The profile and scaling costs ( and ) for each individual time course are shown in the legend. The time courses are named after the data files used; these are available for download as described in Methods. The naming convention is as follows: initial letters denote light condition, dd (constant dark), ll (constant light), rr (constant red light), bb (constant blue light), ld (light dark LD 12:12), lgd (long day LD 16:8), and shd (short day LD 8:16); followed by gene name, C (CCA1), L (LHY), T or P1 (TOC1), G (GI), P5,7,9 (PRR5,7,9), LUX (LUX), NOX (NOX), R8 (RVE8), E3 (ELF3), E4 (ELF4), Z (ZTL); suffixed by “_m” for mRNA data and an arbitrary number for uniqueness, or just the number for protein data. The last part of the filename is “-ox” for overexpression, and/or lower case gene names for mutants. A combination of LL and another light condition indicates entrainment in something other than LD 12:12, followed by LL. Where all data come from the same light conditions, the background is shaded for night; exceptions include scaling groups with data from different photoperiods. (PDF) [file pcbi.1003705.s008.pdf]

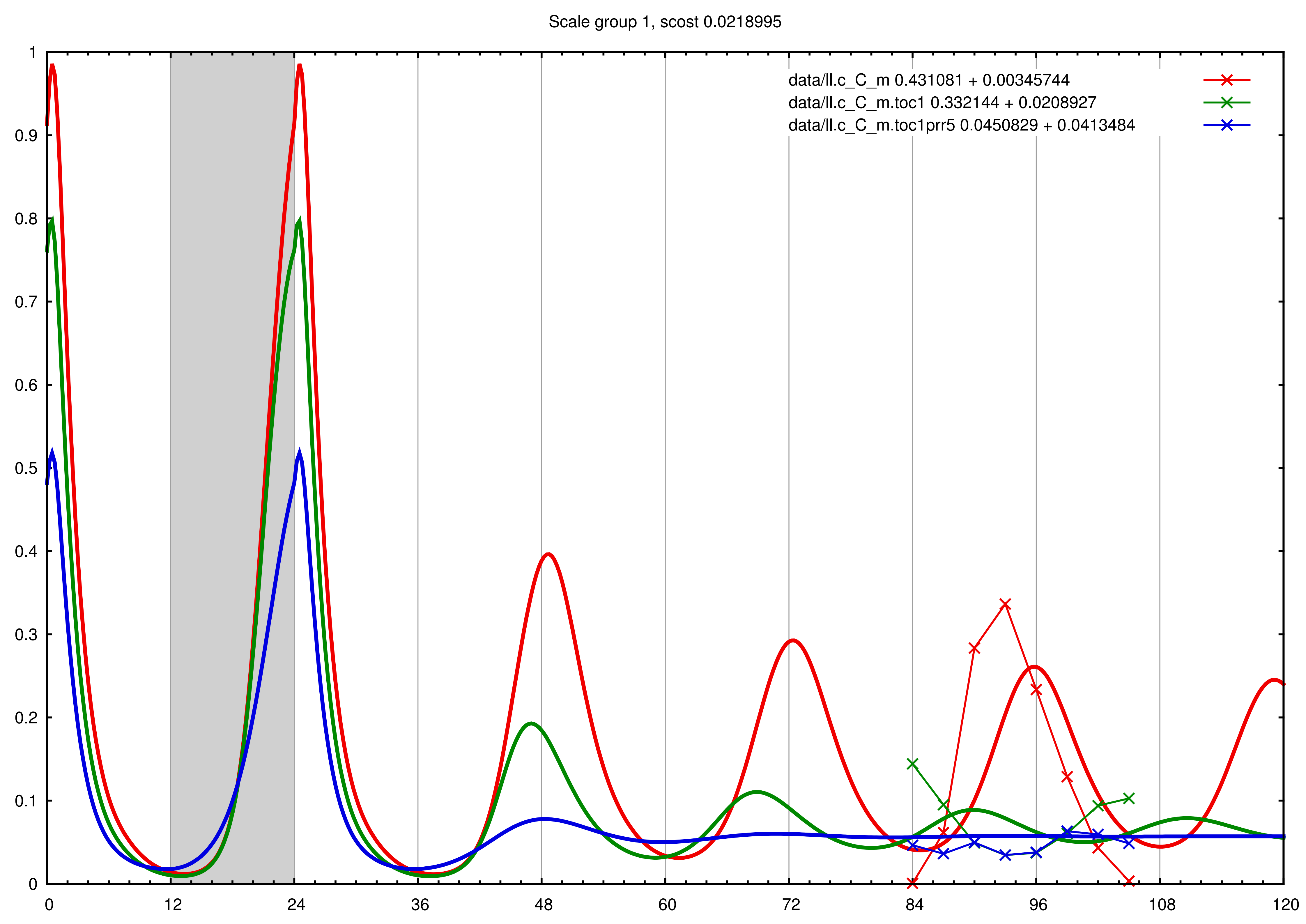
Scale group 1, scost 0.0218995
data/ll.c_C_m 0.431081 + 0.00345744
data/ll.c_C_m.toc1 0.332144 + 0.0208927
data/ll.c_C_m.toc1prr5 0.0450829 + 0.0413484
0
0.1
0.2
0.3
0.4
0.5
0.6
0.7
0.8
0.9
1
0
12
24
36
48
60
72
84
96
108
120

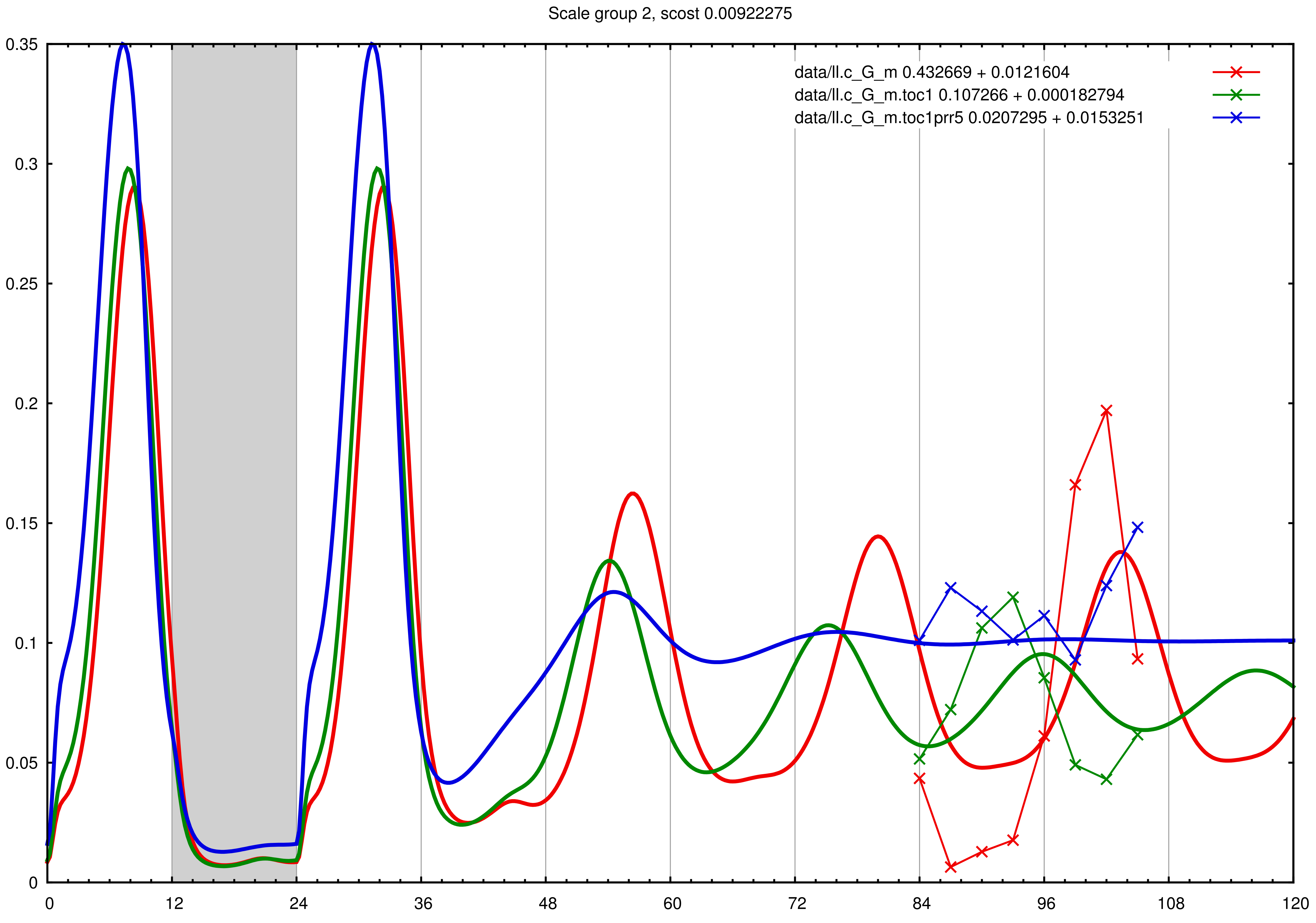

Scale group 2, scost 0.00922275
data/ll.c_G_m 0.432669 + 0.0121604
data/ll.c_G_m.toc1 0.107266 + 0.000182794
data/ll.c_G_m.toc1prr5 0.0207295 + 0.0153251
0.35
0.3
0.25
0.2
0.15
0.1
0.05
0
0
12
24
36
48
60
72
84
96
108
120

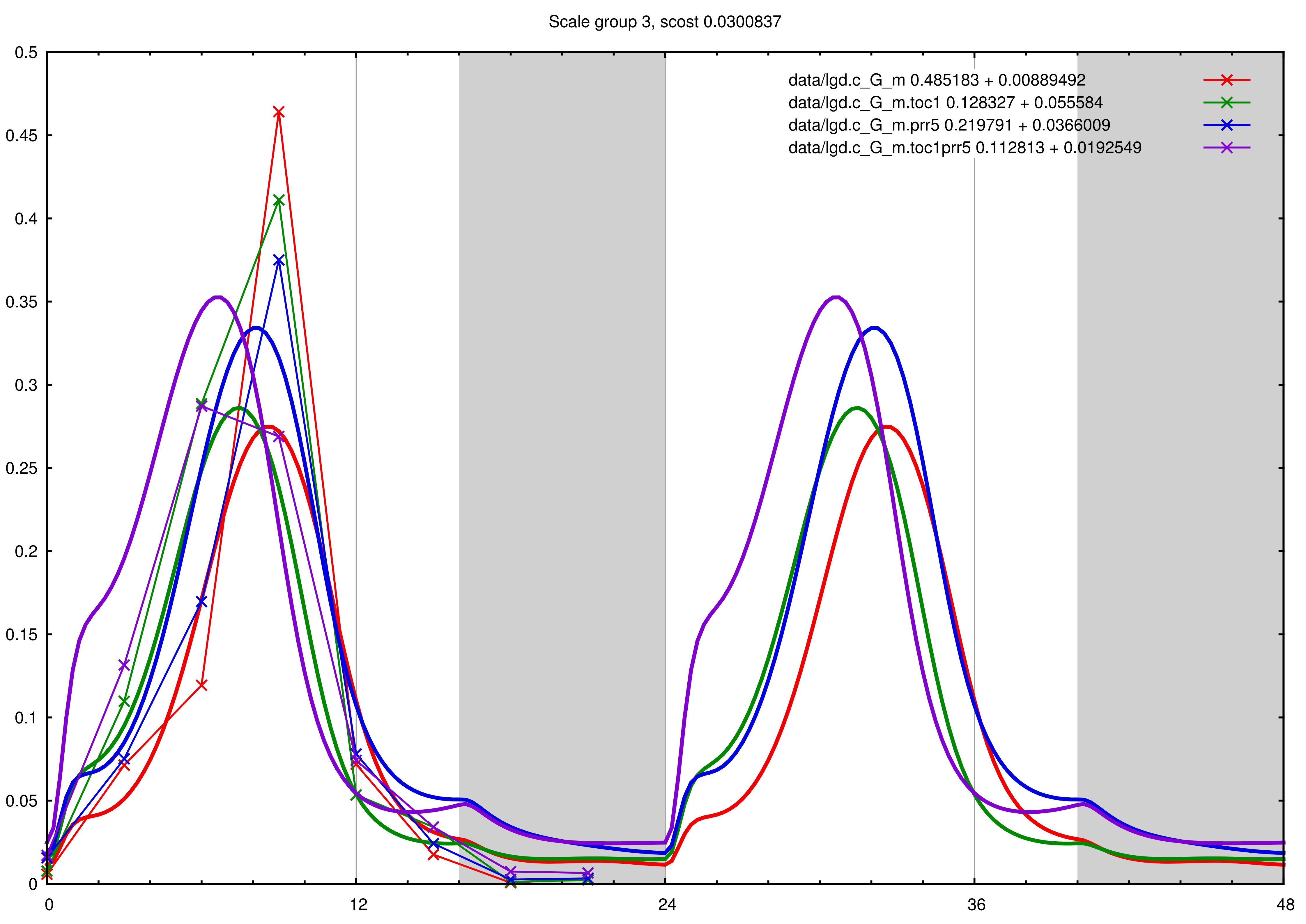

Scale group 3, scost 0.0300837
data/lgd.c_G_m 0.485183 + 0.00889492
data/lgd.c_G_m.toc1 0.128327 + 0.055584
data/lgd.c_G_m.prr5 0.219791 + 0.0366009
data/lgd.c_G_m.toc1prr5 0.112813 + 0.0192549
0.5
0.45
0.4
0.35
0.3
0.25
0.2
0.15
0.1
0.05
0
0
12
24
36
48

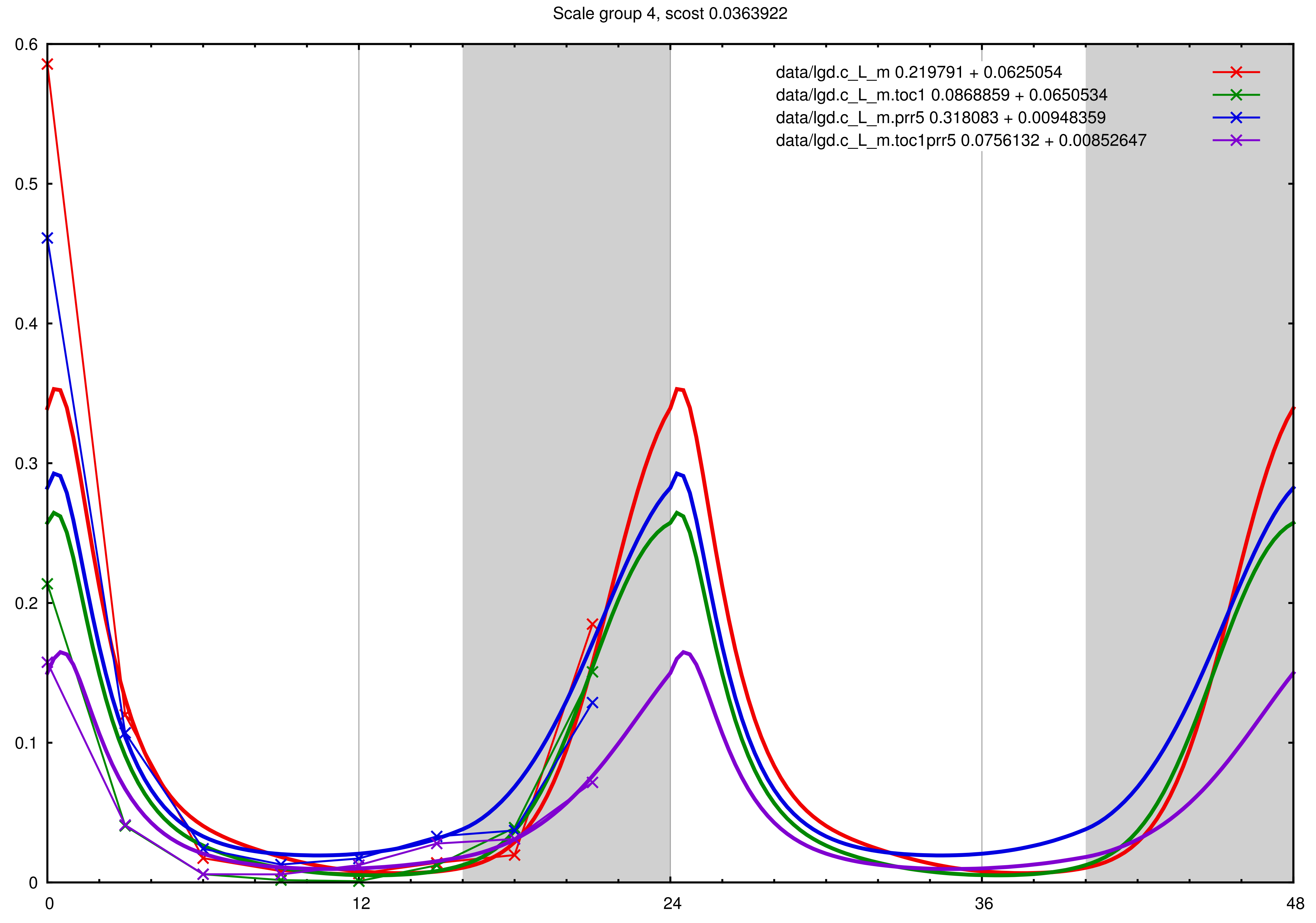
Scale group 4, scost 0.0363922
data/lgd.c_L_m 0.219791 + 0.0625054
data/lgd.c_L_m.toc1 0.0868859 + 0.0650534
data/lgd.c_L_m.prr5 0.318083 + 0.00948359
data/lgd.c_L_m.toc1prr5 0.0756132 + 0.00852647
0
0.1
0.2
0.3
0.4
0.5
0.6
0
12
24
36
48

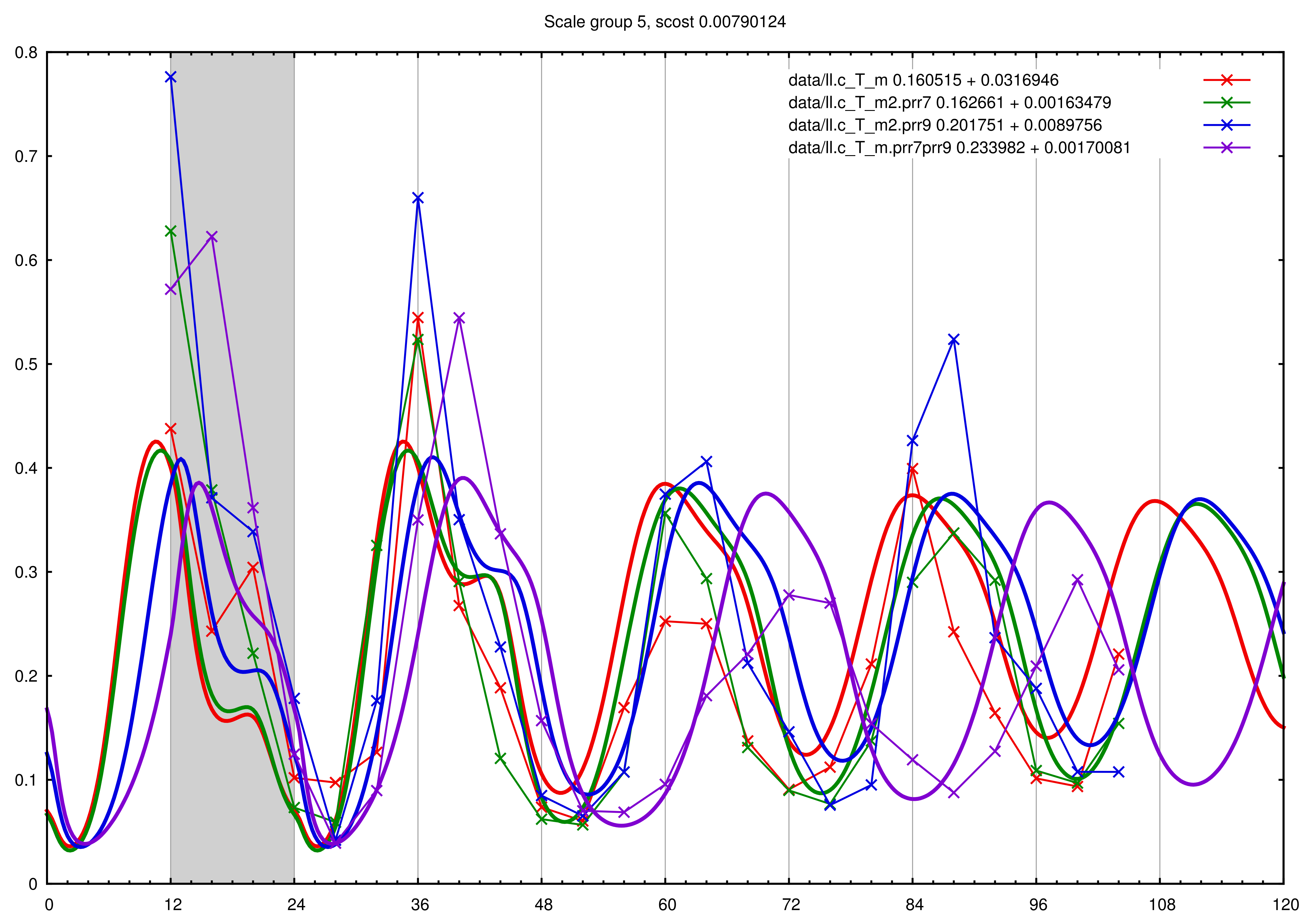

Scale group 5, scost 0.00790124
data/ll.c_T_m 0.160515 + 0.0316946
data/ll.c_T_m2.prr7 0.162661 + 0.00163479
data/ll.c_T_m2.prr9 0.201751 + 0.0089756
data/ll.c_T_m.prr7prr9 0.233982 + 0.00170081
0
0.1
0.2
0.3
0.4
0.5
0.6
0.7
0.8
0
12
24
36
48
60
72
84
96
108
120

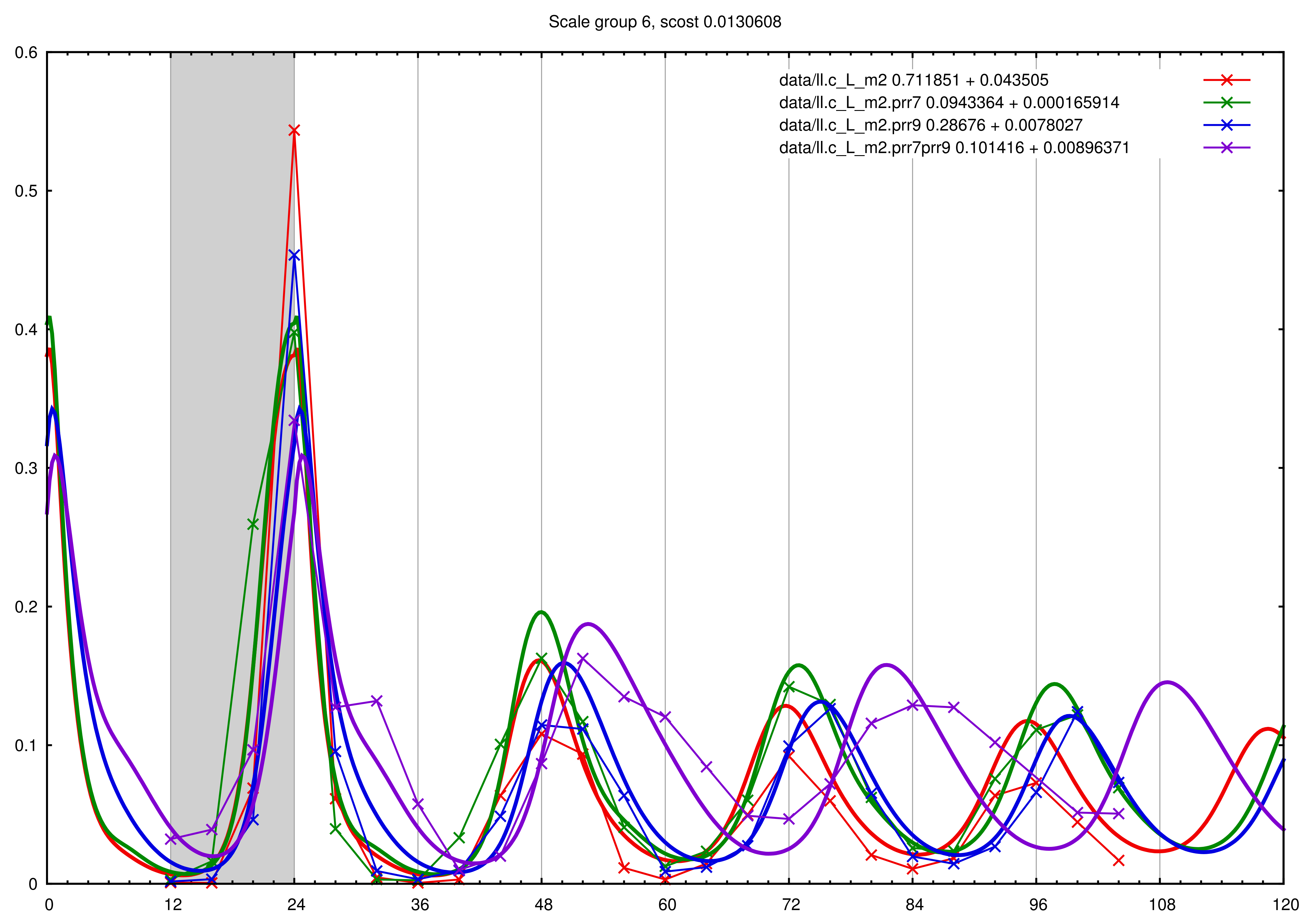
Scale group 6, scost 0.0130608
data/ll.c_L_m2 0.711851 + 0.043505
data/ll.c_L_m2.prr7 0.0943364 + 0.000165914
data/ll.c_L_m2.prr9 0.28676 + 0.0078027
data/ll.c_L_m2.prr7prr9 0.101416 + 0.00896371
0
0.1
0.2
0.3
0.4
0.5
0.6
0
12
24
36
48
60
72
84
96
108
120

Scale group 7, scost 0.0132883

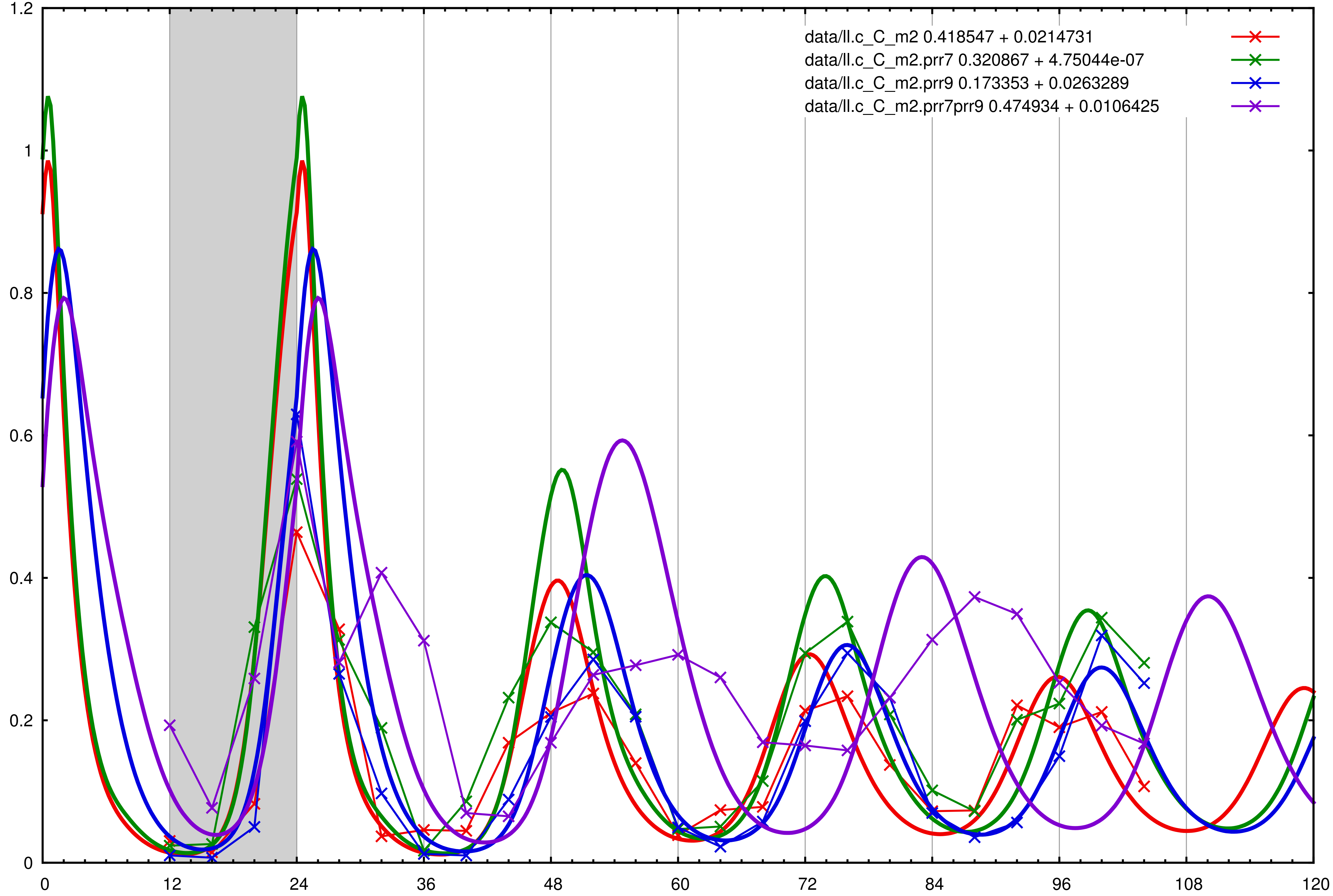

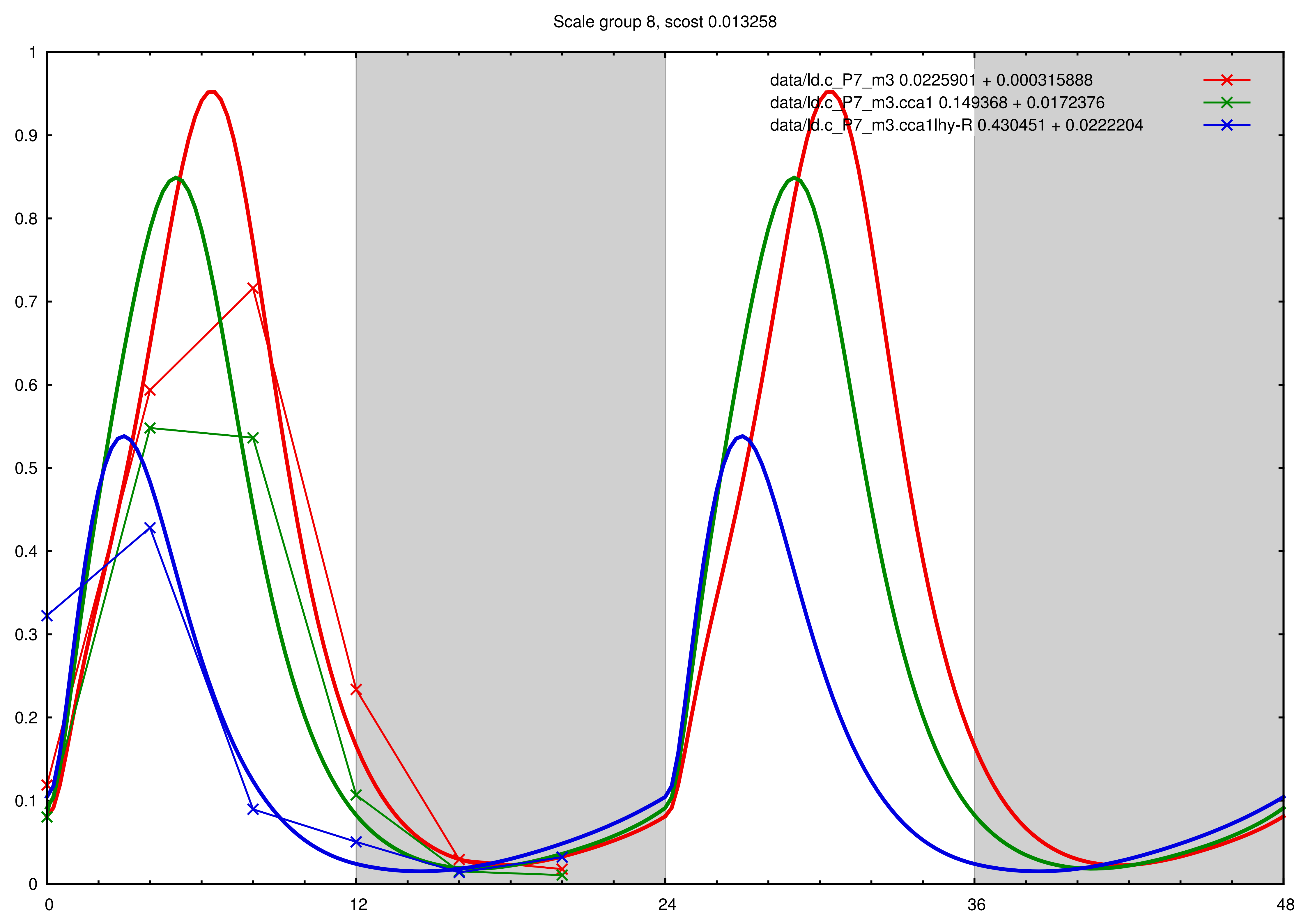

Scale group 8, scost 0.013258
data/ld.c_P7_m3 0.0225901 + 0.000315888
data/ld.c_P7_m3.cca1 0.149368 + 0.0172376
data/ld.c_P7_m3.cca1lhy-R 0.430451 + 0.0222204
1
0.9
0.8
0.7
0.6
0.5
0.4
0.3
0.2
0.1
0
0
12
24
36
48

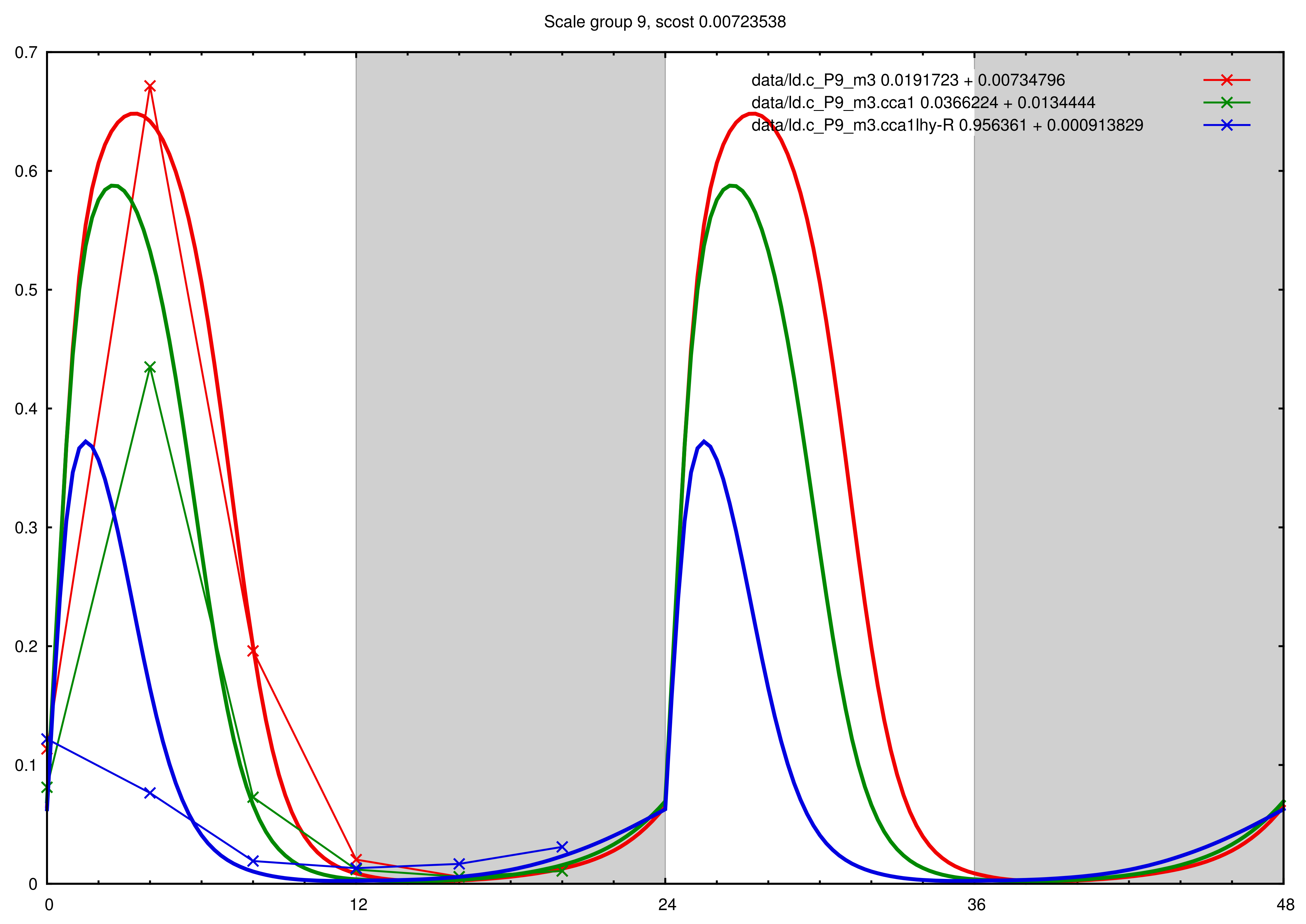

Scale group 9, scost 0.00723538
data/ld.c_P9_m3 0.0191723 + 0.00734796
data/ld.c_P9_m3.cca1 0.0366224 + 0.0134444
data/ld.c_P9_m3.cca1lhy-R 0.956361 + 0.000913829
0.7
0.6
0.5
0.4
0.3
0.2
0.1
0
0
12
24
36
48

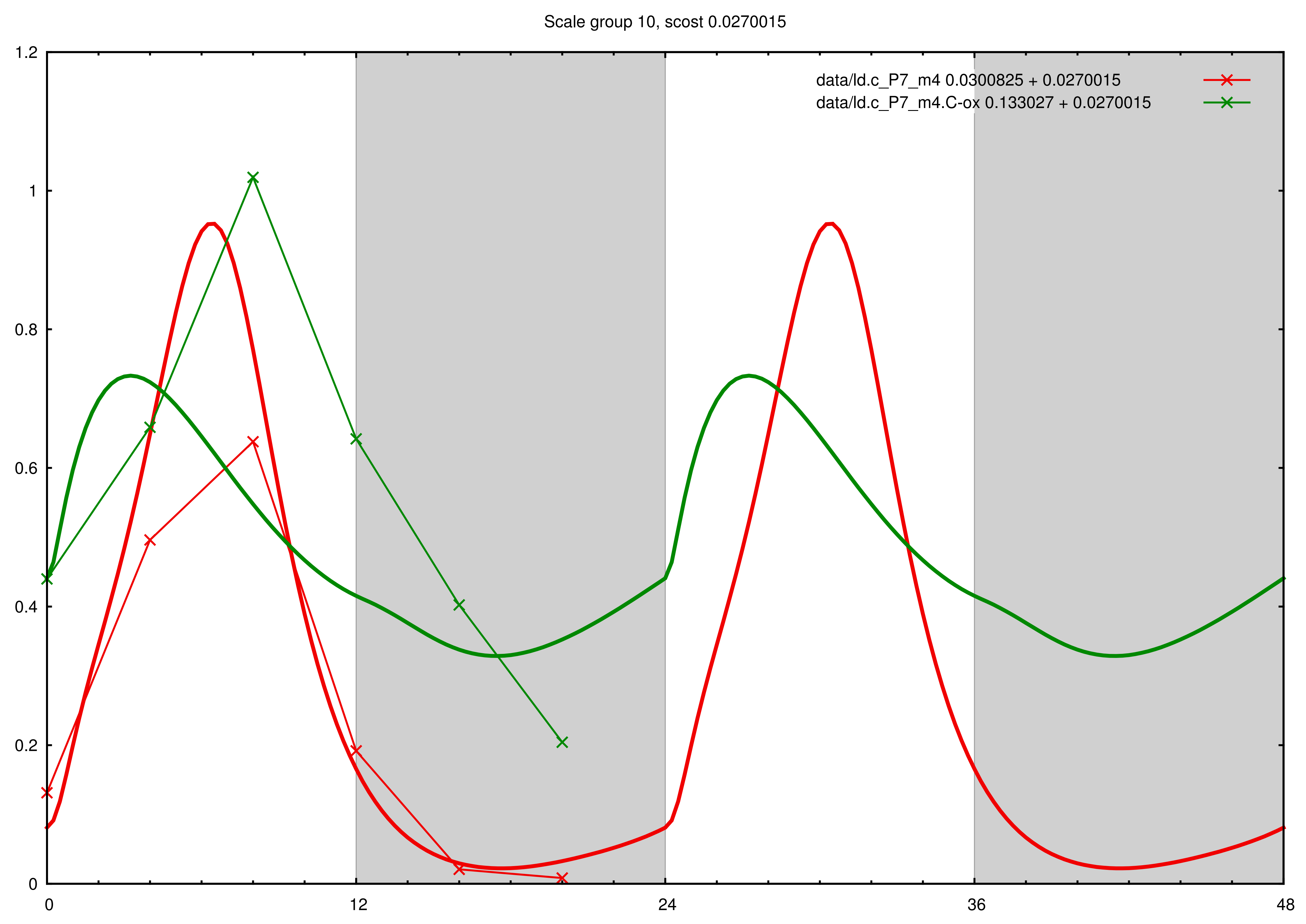

Scale group 10, scost 0.0270015
data/ld.c_P7_m4 0.0300825 + 0.0270015
data/ld.c_P7_m4.C-ox 0.133027 + 0.0270015
1.2
1
0.8
0.6
0.4
0.2
0
0
12
24
36
48

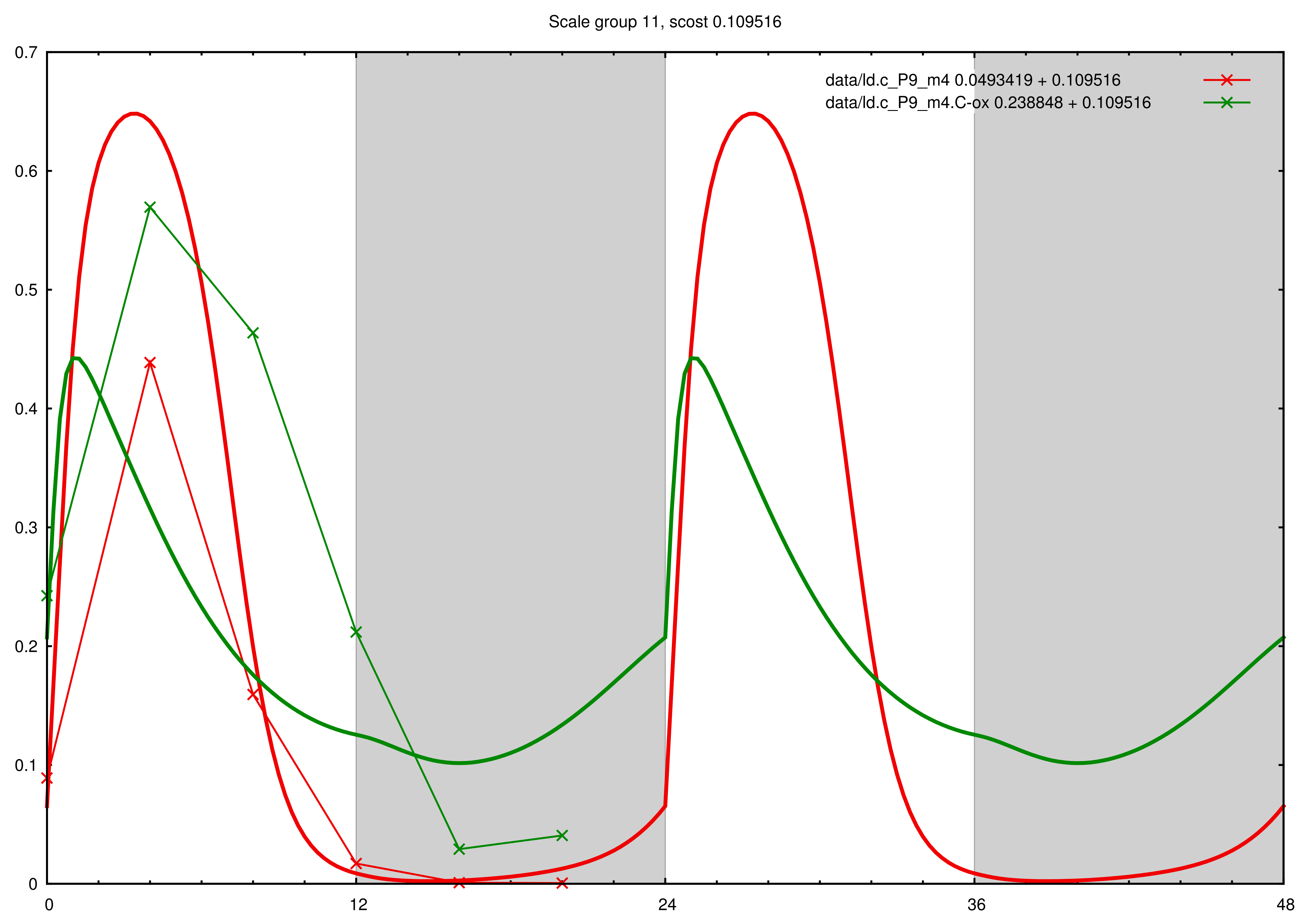
Scale group 11, scost 0.109516
data/ld.c_P9_m4 0.0493419 + 0.109516
data/ld.c_P9_m4.C-ox 0.238848 + 0.109516
0.7
0.6
0.5
0.4
0.3
0.2
0.1
0
0
12
24
36
48

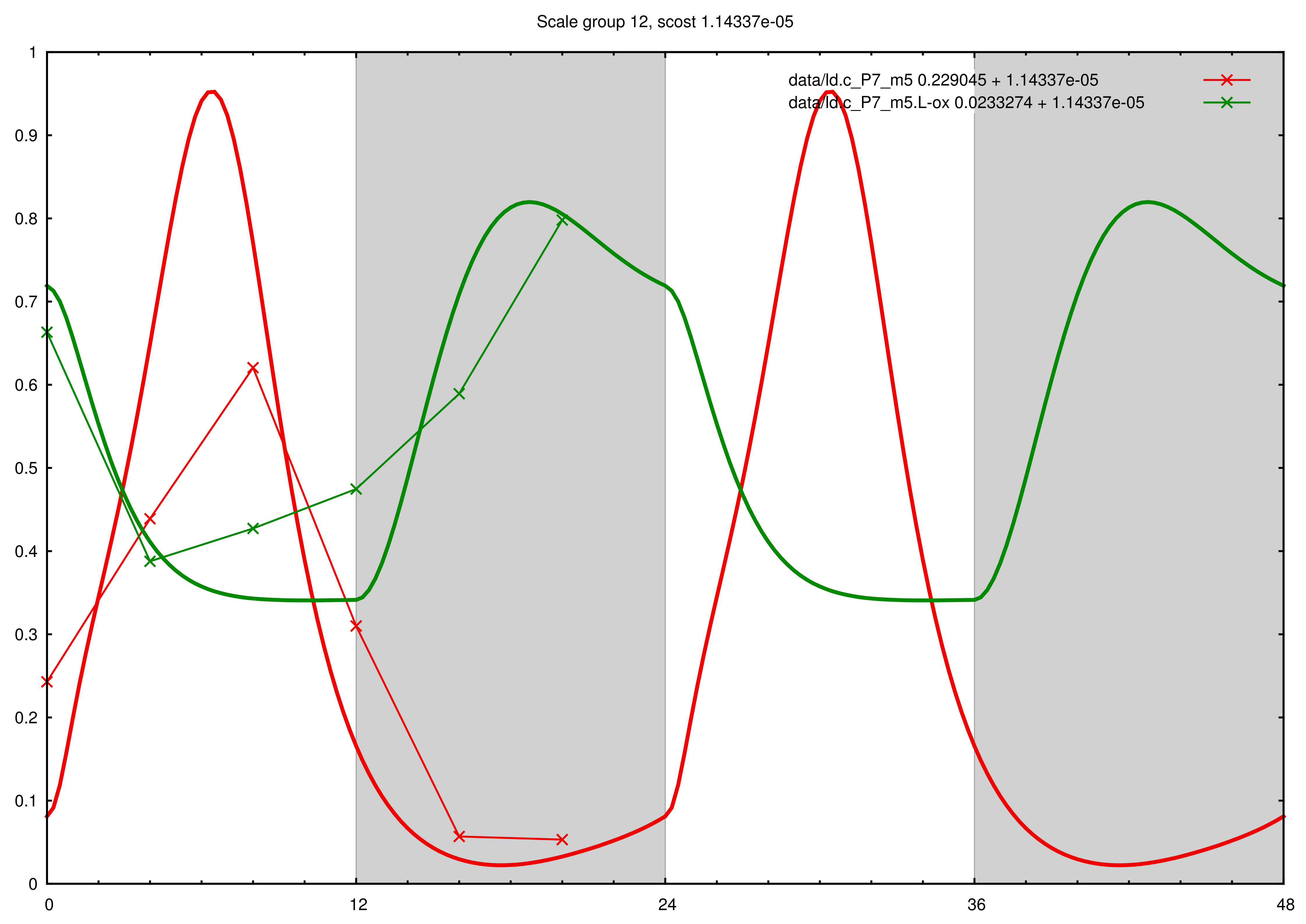

Scale group 12, scost 1.14337e-05
data/ld.c_P7_m5 0.229045 + 1.14337e-05
data/ld.c_P7_m5.L-ox 0.0233274 + 1.14337e-05
1
0.9
0.8
0.7
0.6
0.5
0.4
0.3
0.2
0.1
0
0
12
24
36
48

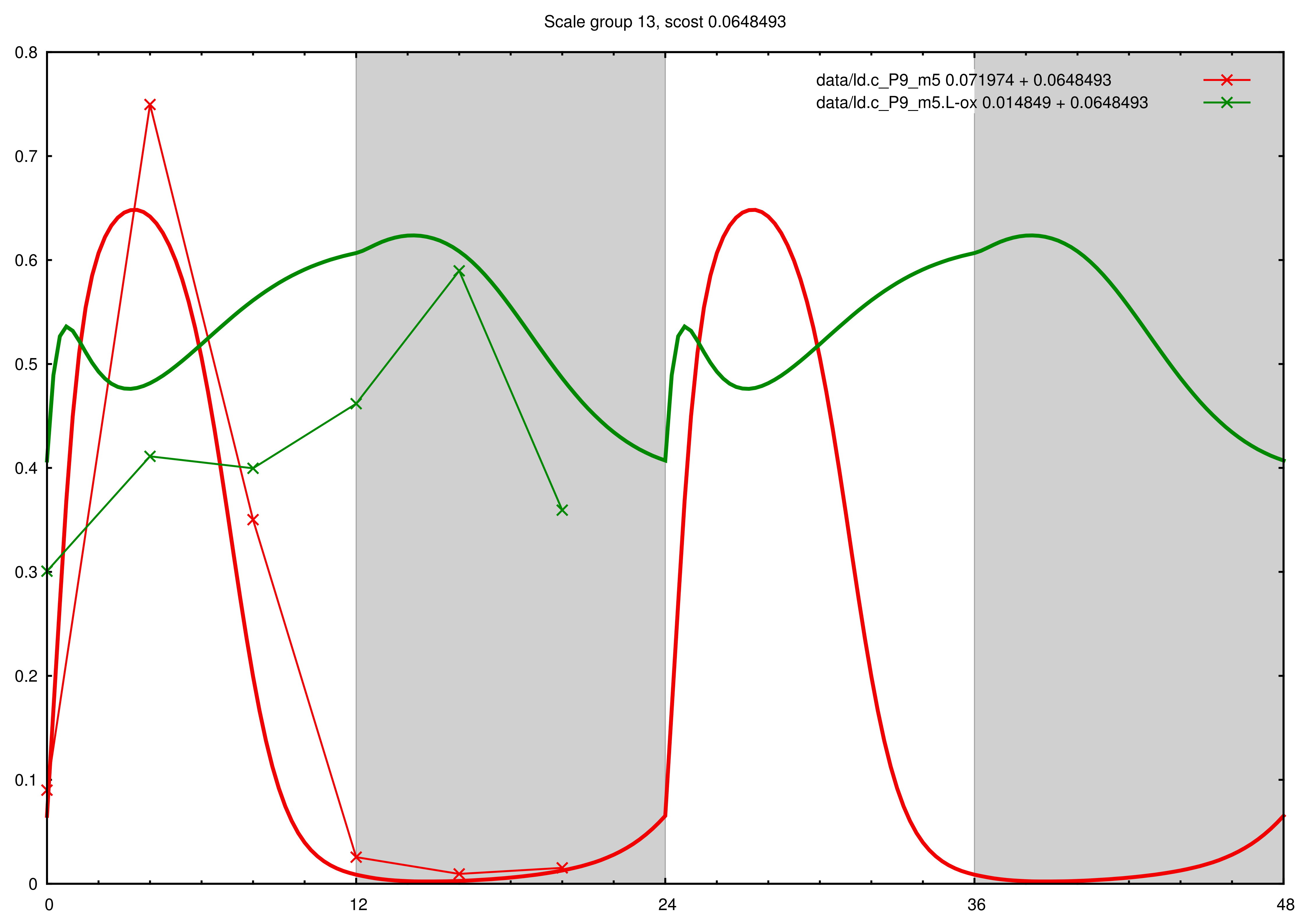
Scale group 13, scost 0.0648493
data/ld.c_P9_m5 0.071974 + 0.0648493
data/ld.c_P9_m5.L-ox 0.014849 + 0.0648493
0.8
0.7
0.6
0.5
0.4
0.3
0.2
0.1
0
0
12
24
36
48

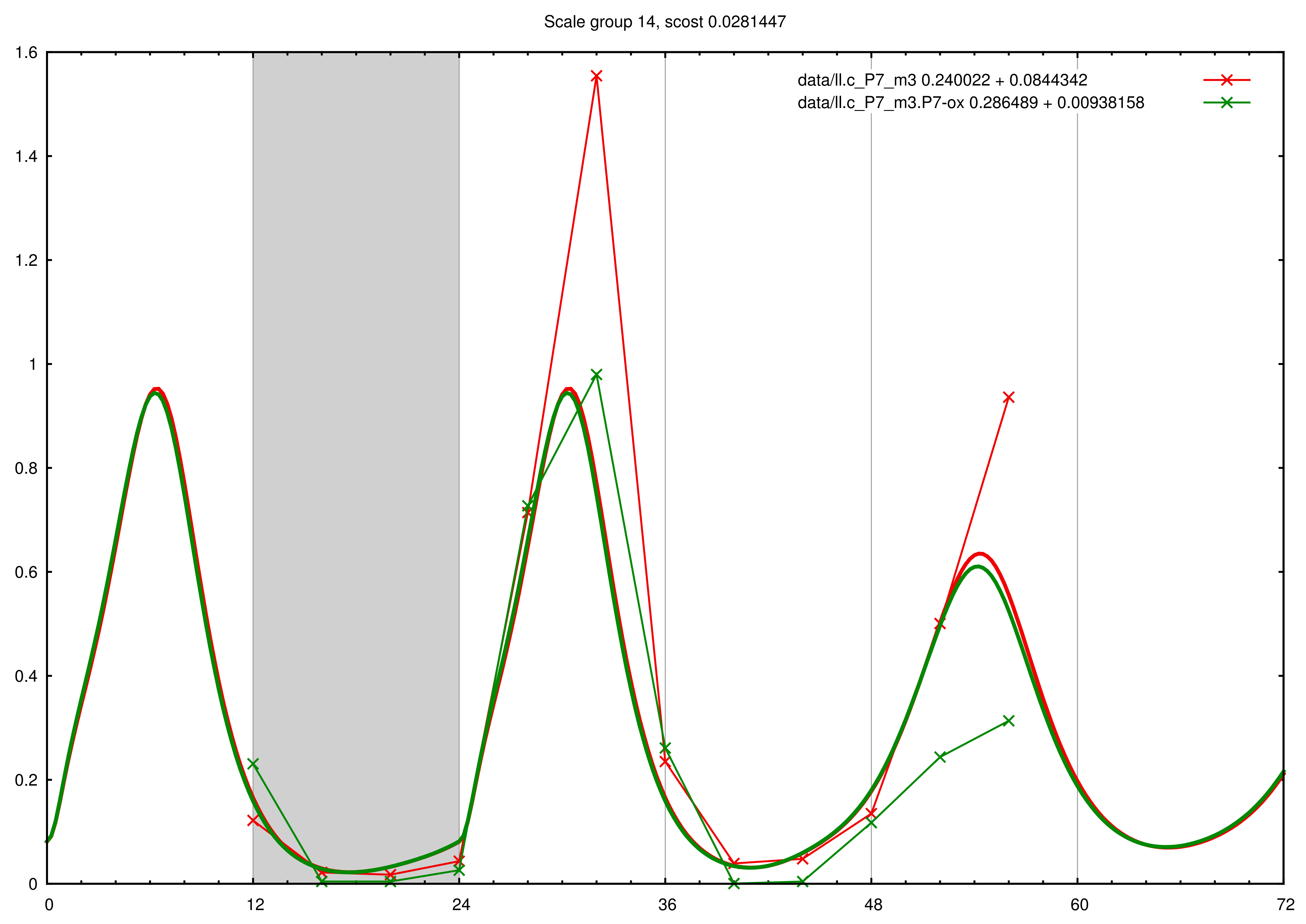

Scale group 14, scost 0.0281447
data/ll.c_P7_m3 0.240022 + 0.0844342
data/ll.c_P7_m3.P7-ox 0.286489 + 0.00938158
0
0.2
0.4
0.6
0.8
1
1.2
1.4
1.6
0
12
24
36
48
60
72

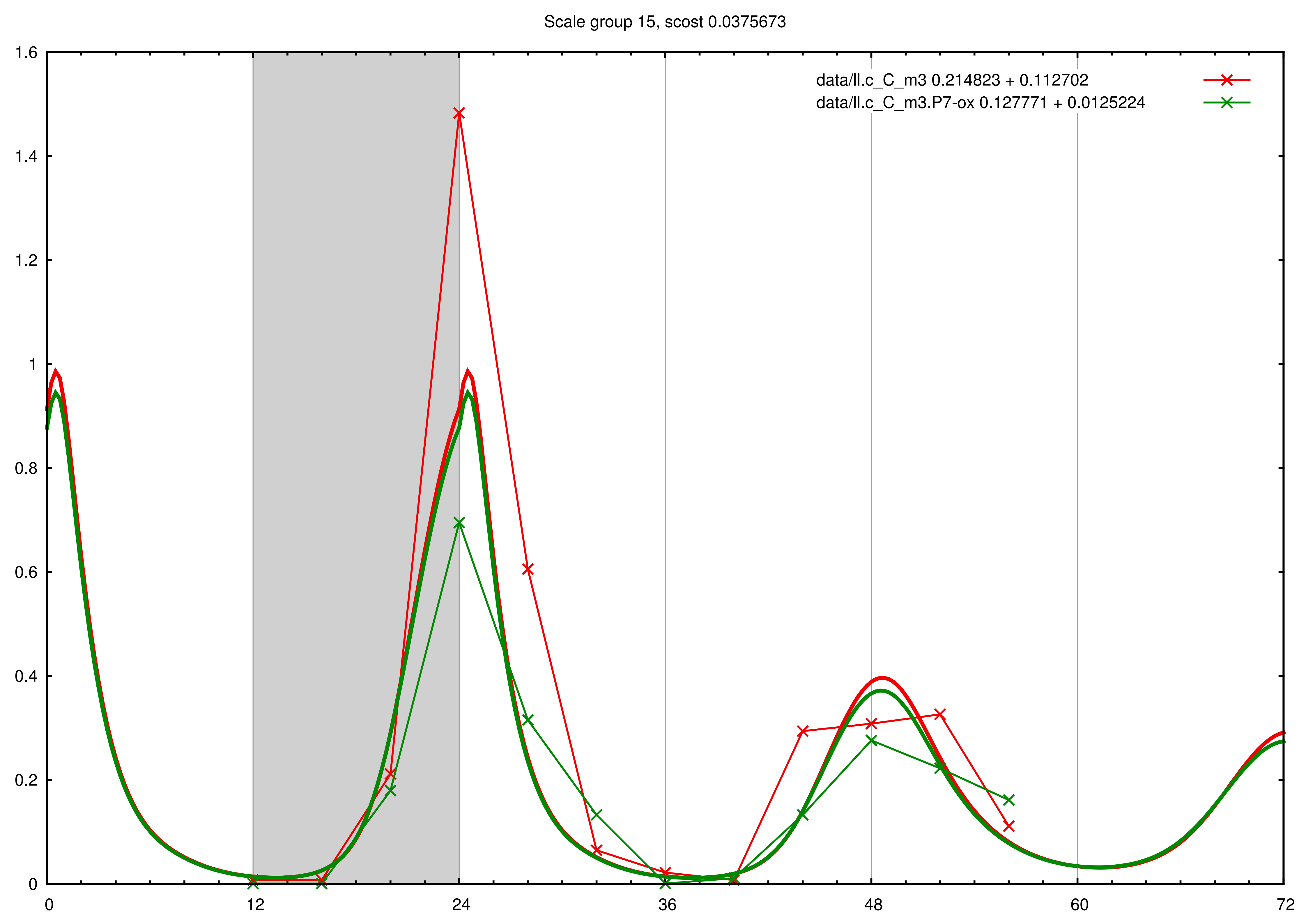
Scale group 15, scost 0.0375673
data/ll.c_C_m3 0.214823 + 0.112702
data/ll.c_C_m3.P7-ox 0.127771 + 0.0125224
1.6
1.4
1.2
1
0.8
0.6
0.4
0.2
0
0
12
24
36
48
60
72

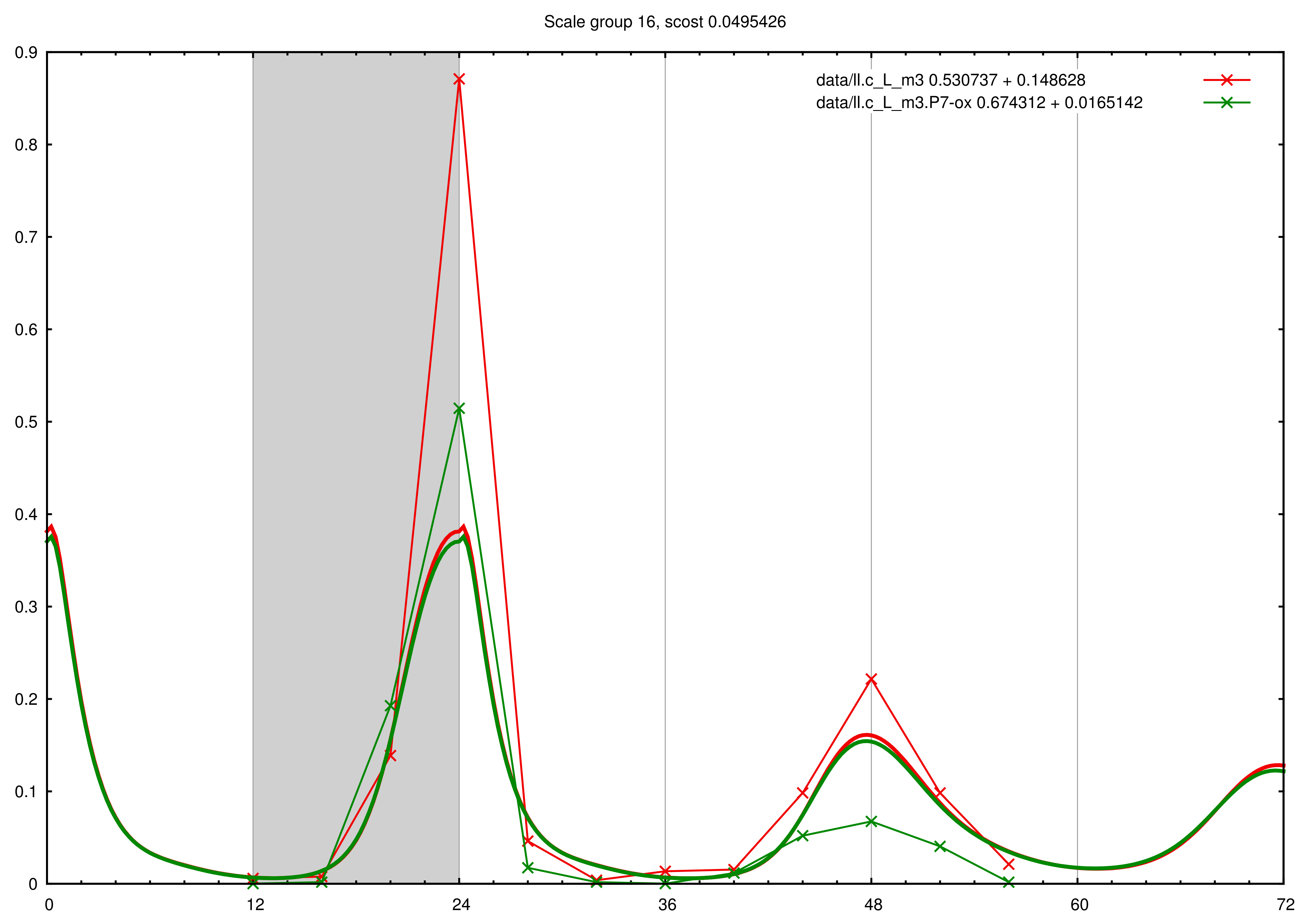

Scale group 16, scost 0.0495426
data/ll.c_L_m3 0.530737 + 0.148628
data/ll.c_L_m3.P7-ox 0.674312 + 0.0165142
0.9
0.8
0.7
0.6
0.5
0.4
0.3
0.2
0.1
0
0
12
24
36
48
60
72

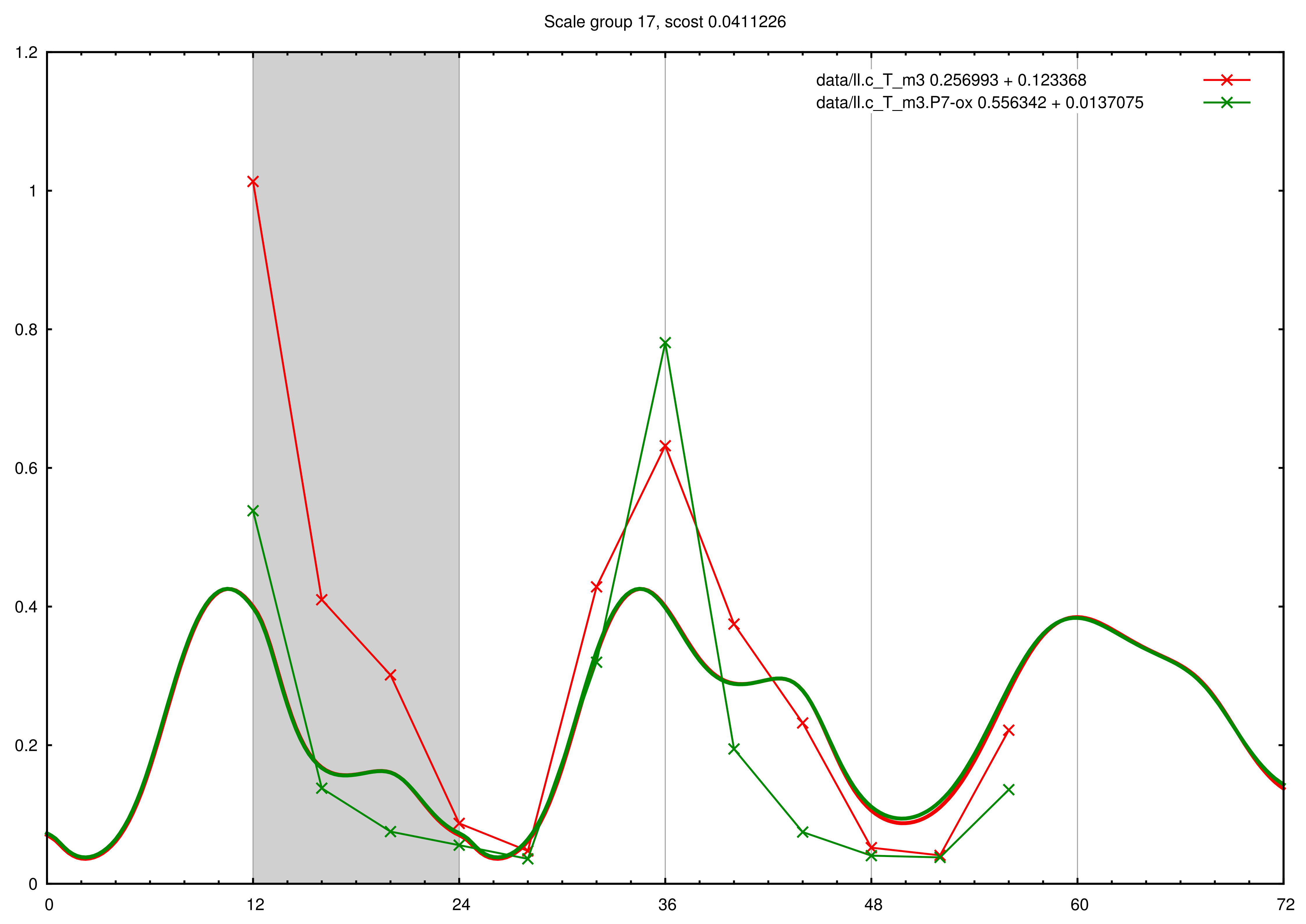
Scale group 17, scost 0.0411226
data/ll.c_T_m3 0.256993 + 0.123368
data/ll.c_T_m3.P7-ox 0.556342 + 0.0137075
1.2
1
0.8
0.6
0.4
0.2
0
0
12
24
36
48
60
72

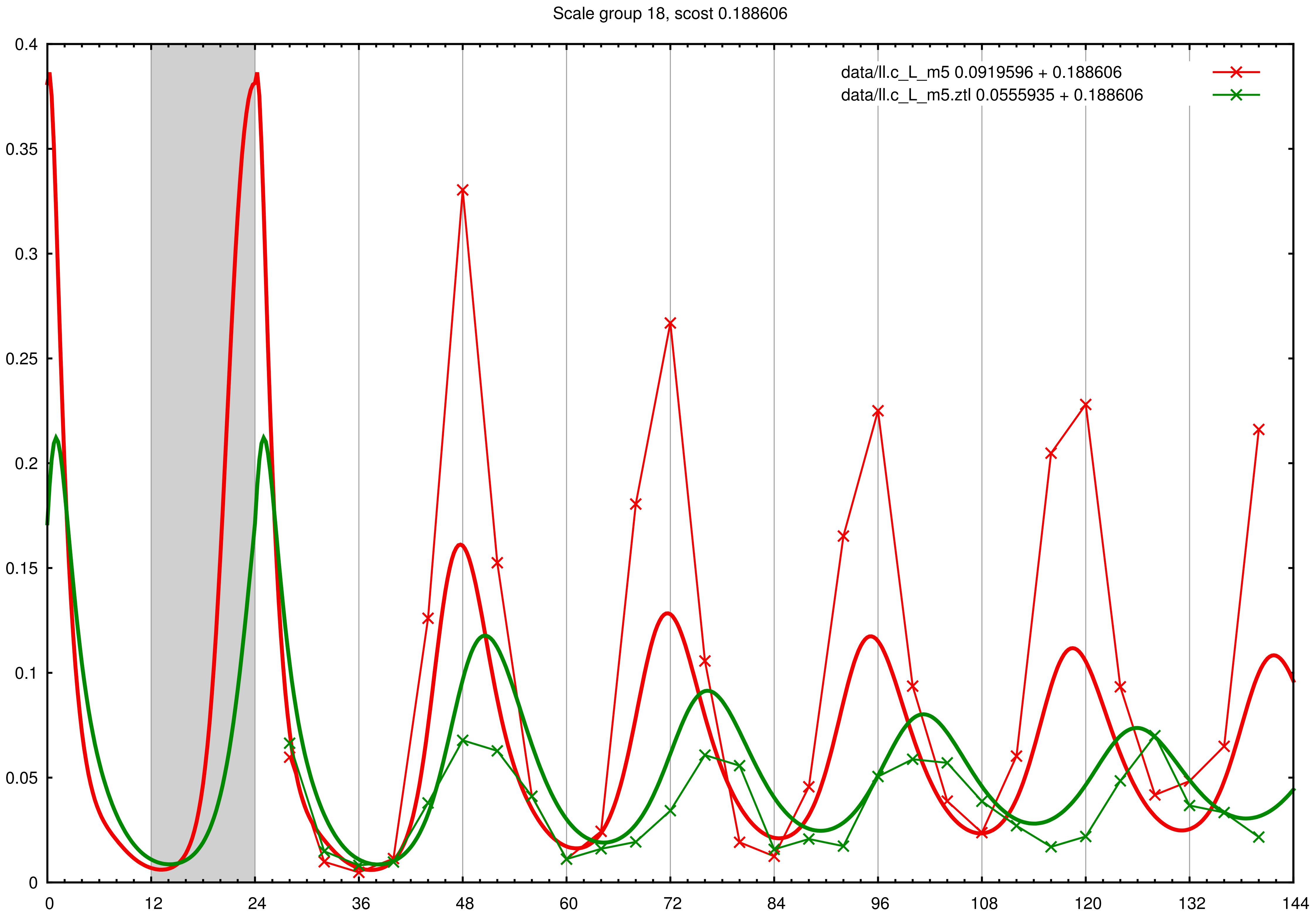

Scale group 18, scost 0.188606
data/ll.c_L_m5 0.0919596 + 0.188606
data/ll.c_L_m5.ztl 0.0555935 + 0.188606
0.4
0.35
0.3
0.25
0.2
0.15
0.1
0.05
0
0
12
24
36
48
60
72
84
96
108
120
132
144

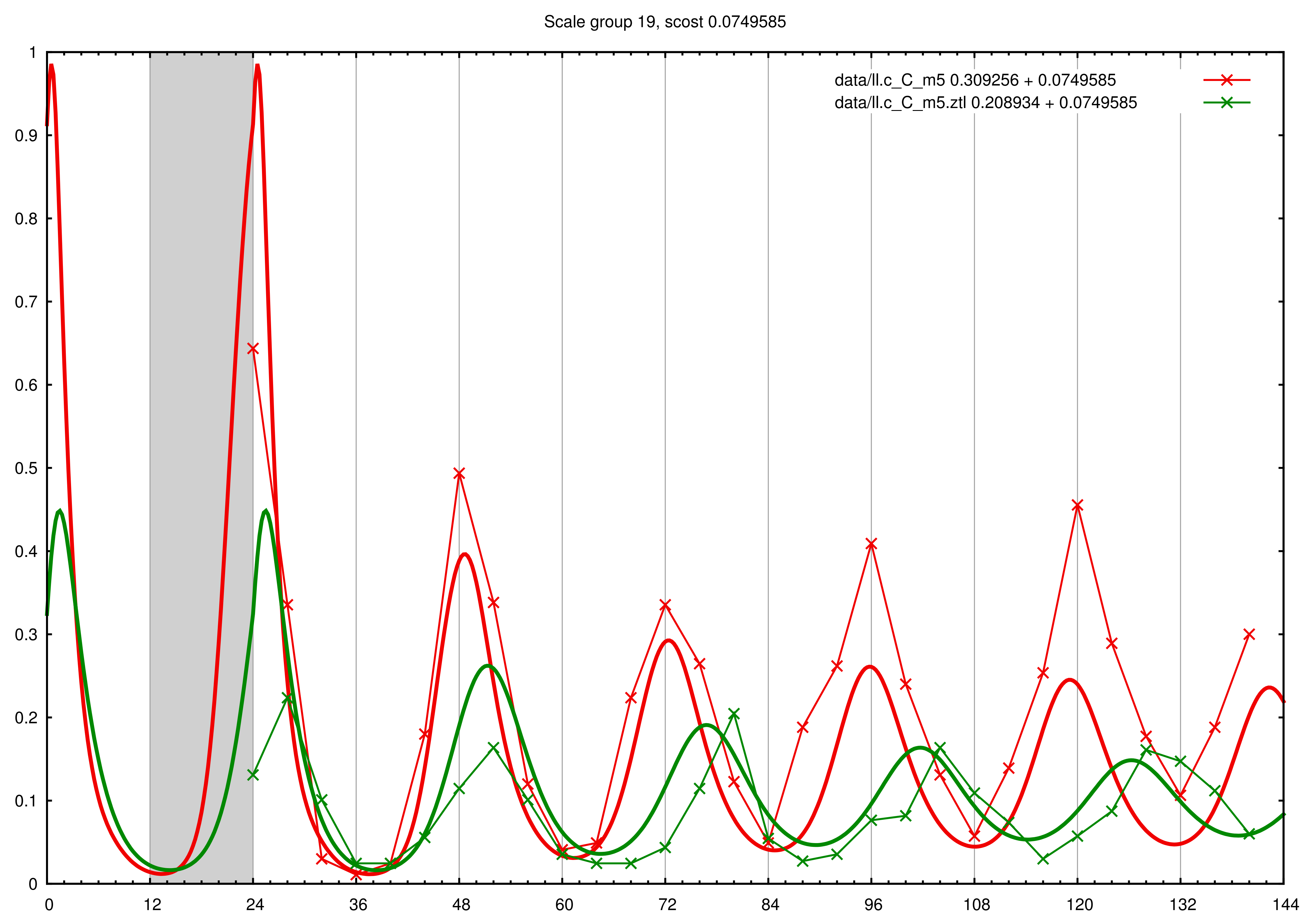

Scale group 19, scost 0.0749585
data/ll.c_C_m5 0.309256 + 0.0749585
data/ll.c_C_m5.ztl 0.208934 + 0.0749585
0
0.1
0.2
0.3
0.4
0.5
0.6
0.7
0.8
0.9
1
0
12
24
36
48
60
72
84
96
108
120
132
144

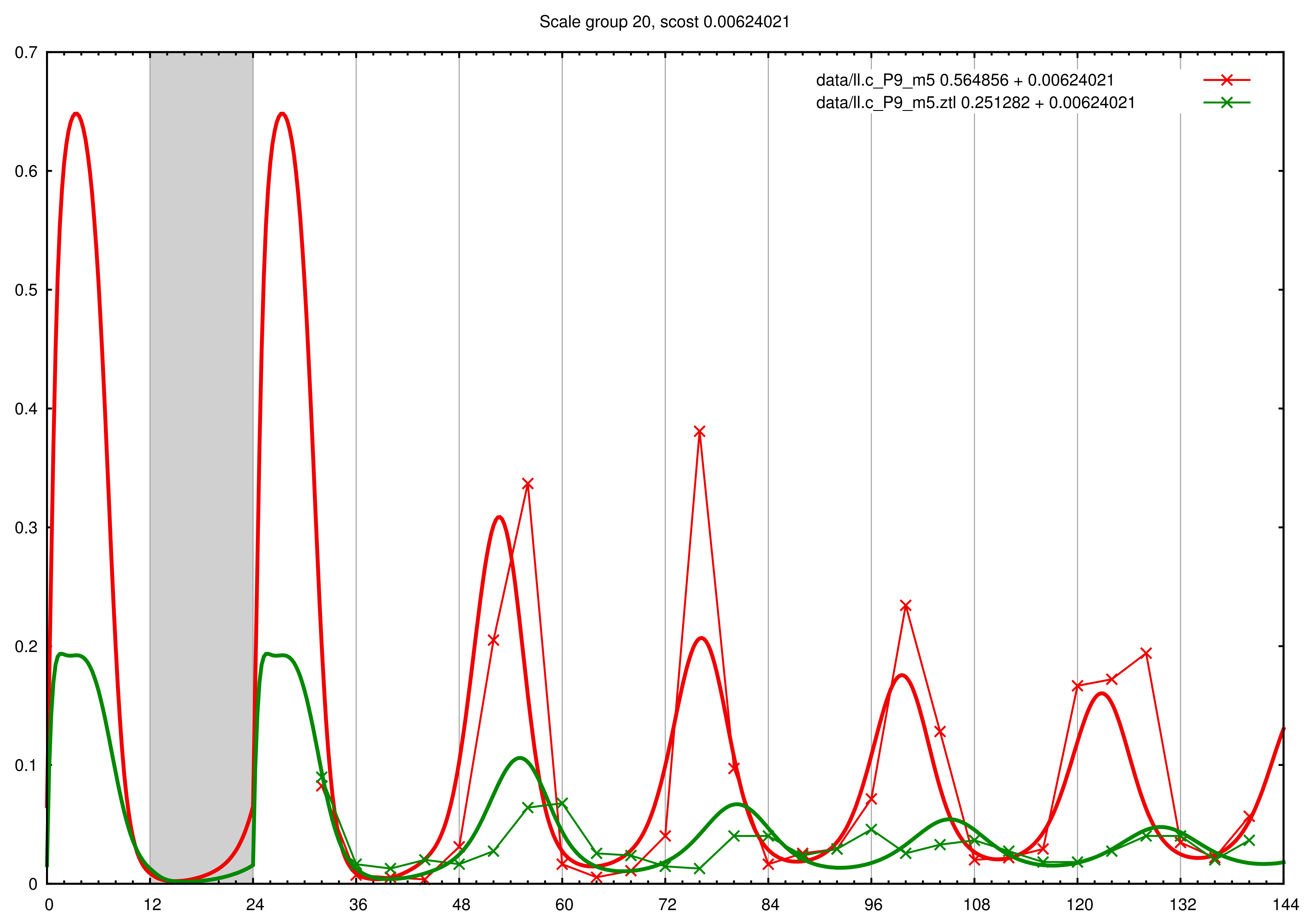

Scale group 20, scost 0.00624021
data/ll.c_P9_m5 0.564856 + 0.00624021
data/ll.c_P9_m5.ztl 0.251282 + 0.00624021
0.7
0.6
0.5
0.4
0.3
0.2
0.1
0
0
12
24
36
48
60
72
84
96
108
120
132
144

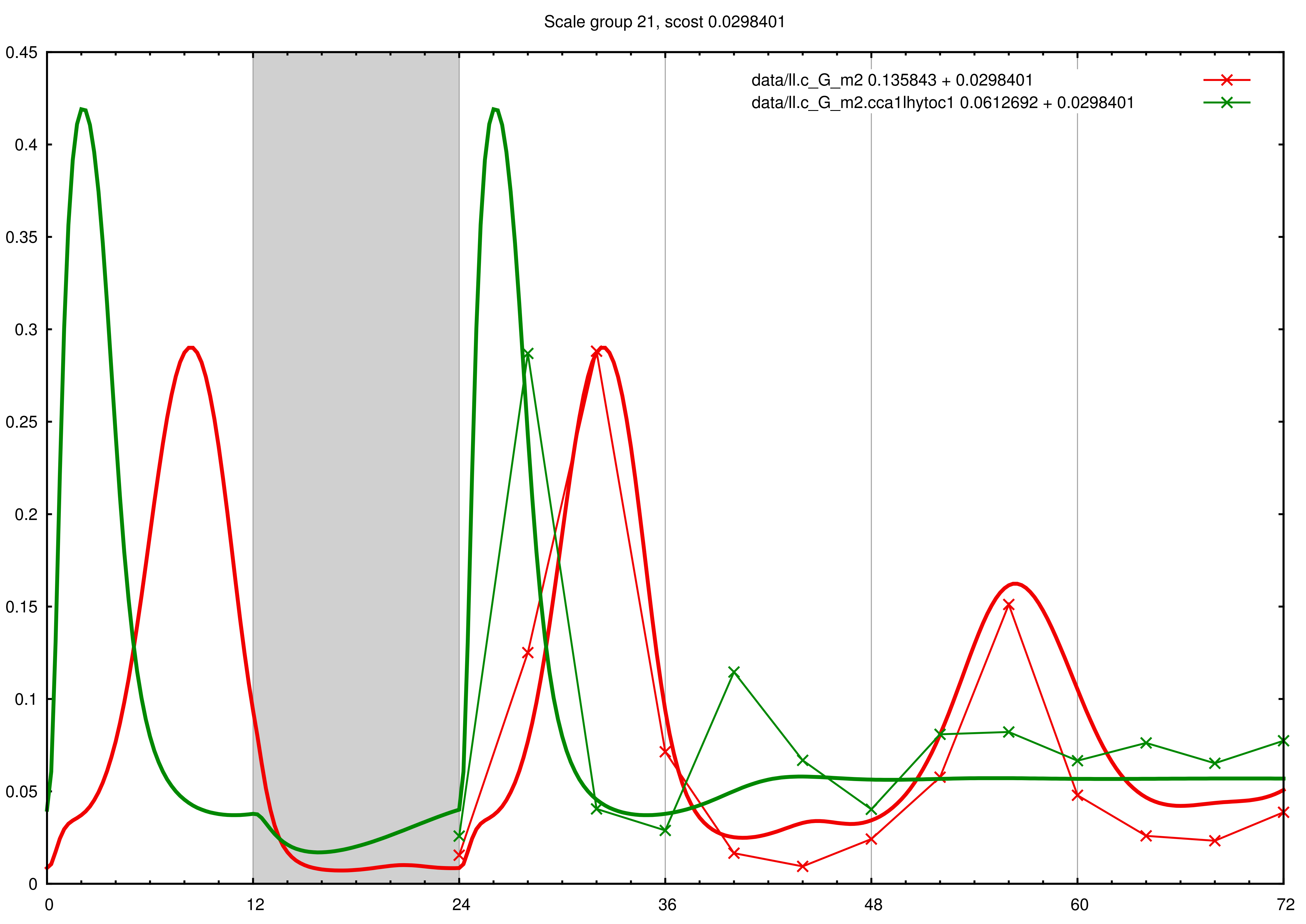

Scale group 21, scost 0.0298401
data/ll.c_G_m2 0.135843 + 0.0298401
data/ll.c_G_m2.cca1lhytoc1 0.0612692 + 0.0298401
0.45
0.4
0.35
0.3
0.25
0.2
0.15
0.1
0.05
0
0
12
24
36
48
60
72

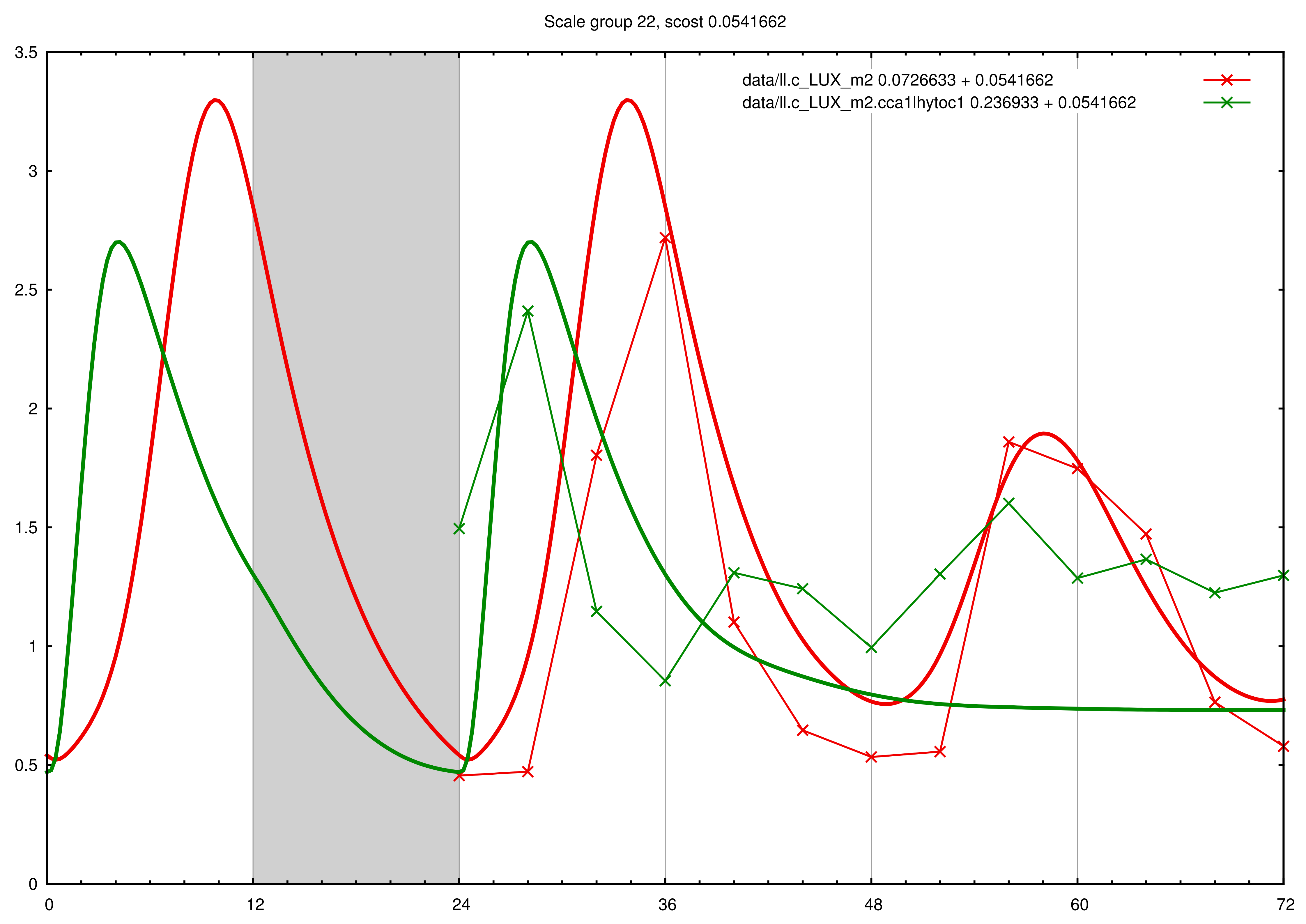

Scale group 22, scost 0.0541662
data/ll.c_LUX_m2 0.0726633 + 0.0541662
data/ll.c_LUX_m2.cca1lhytoc1 0.236933 + 0.0541662
3.5
3
2.5
2
1.5
1
0.5
0
0
12
24
36
48
60
72

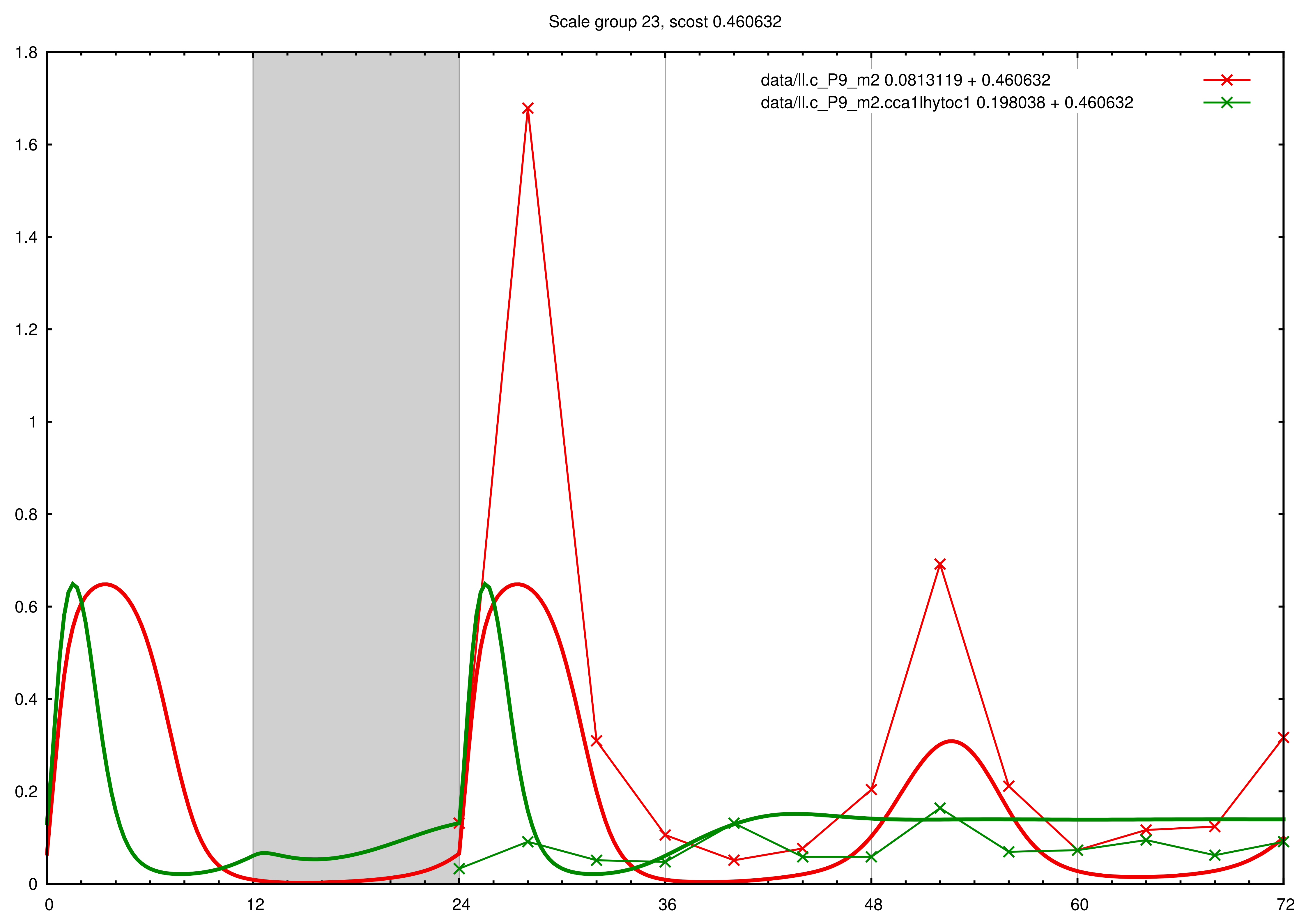

Scale group 23, scost 0.460632
data/ll.c_P9_m2 0.0813119 + 0.460632
data/ll.c_P9_m2.cca1lhytoc1 0.198038 + 0.460632
1.8
1.6
1.4
1.2
1
0.8
0.6
0.4
0.2
0
0
12
24
36
48
60
72

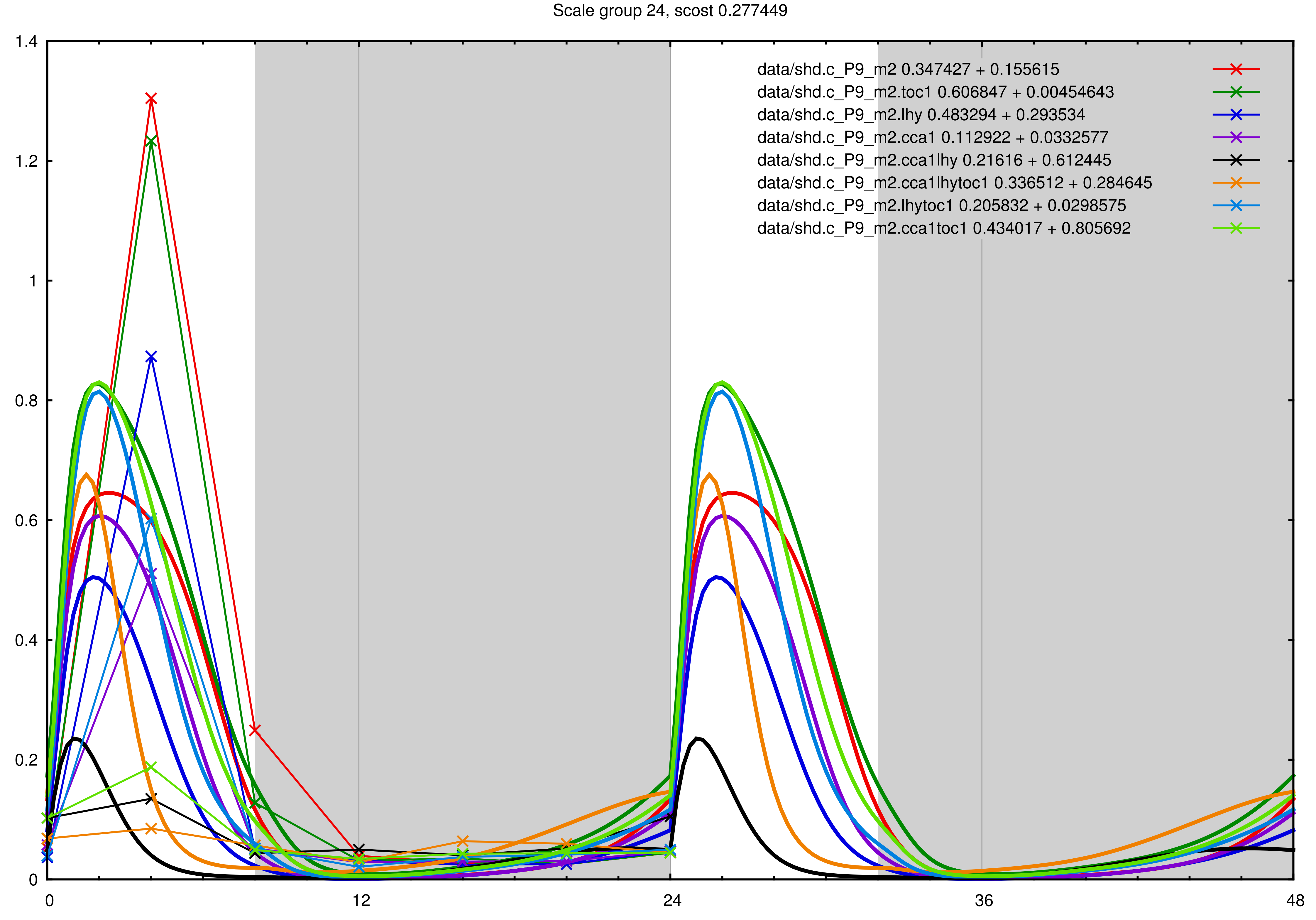

Scale group 24, scost 0.277449
data/shd.c_P9_m2 0.347427 + 0.155615
data/shd.c_P9_m2.toc1 0.606847 + 0.00454643
data/shd.c_P9_m2.lhy 0.483294 + 0.293534
data/shd.c_P9_m2.cca1 0.112922 + 0.0332577
data/shd.c_P9_m2.cca1lhy 0.21616 + 0.612445
data/shd.c_P9_m2.cca1lhytoc1 0.336512 + 0.284645
data/shd.c_P9_m2.lhytoc1 0.205832 + 0.0298575
data/shd.c_P9_m2.cca1toc1 0.434017 + 0.805692
1.4
1.2
1
0.8
0.6
0.4
0.2
0
0
12
24
36
48

Scale group 25, scost 0.207231

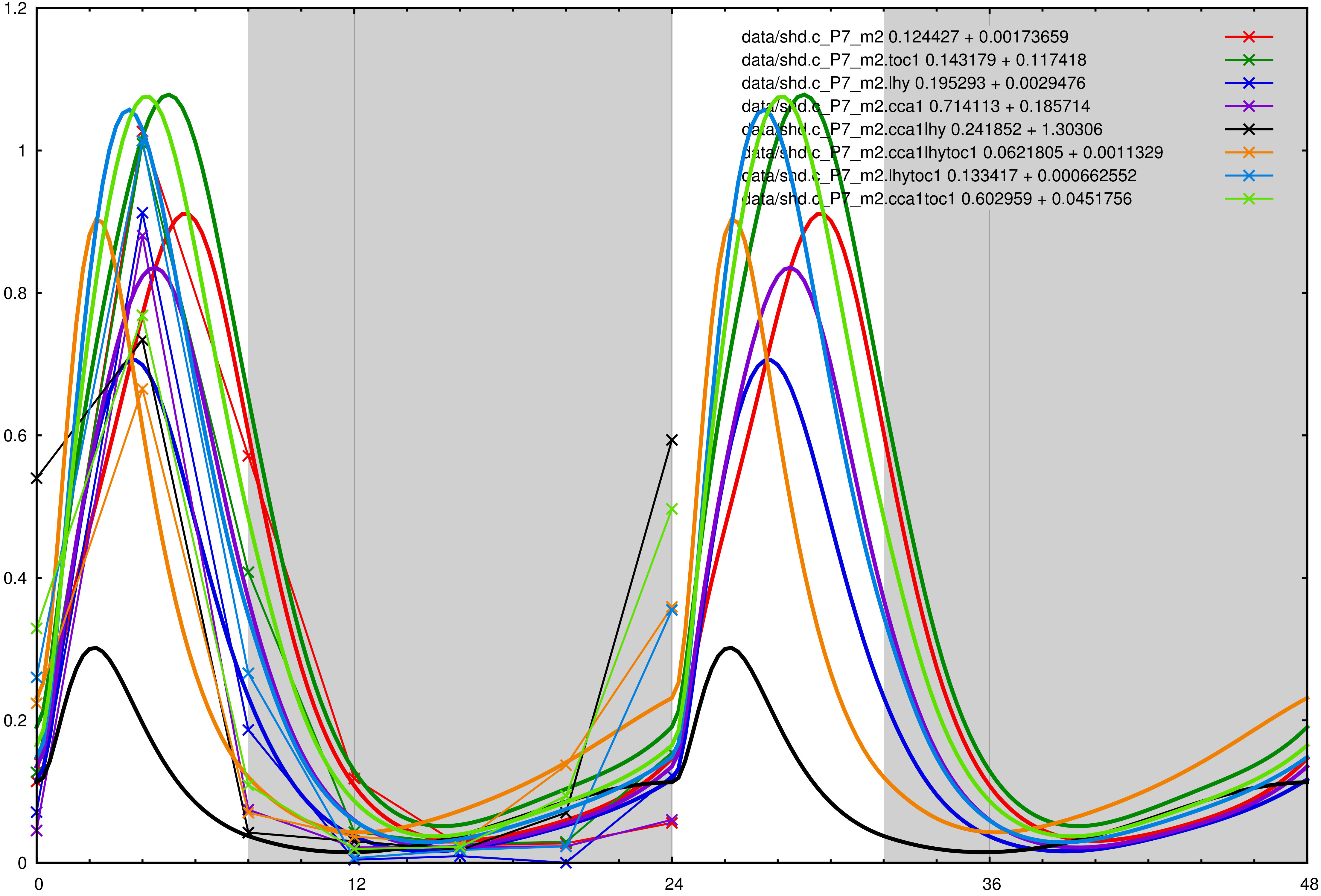

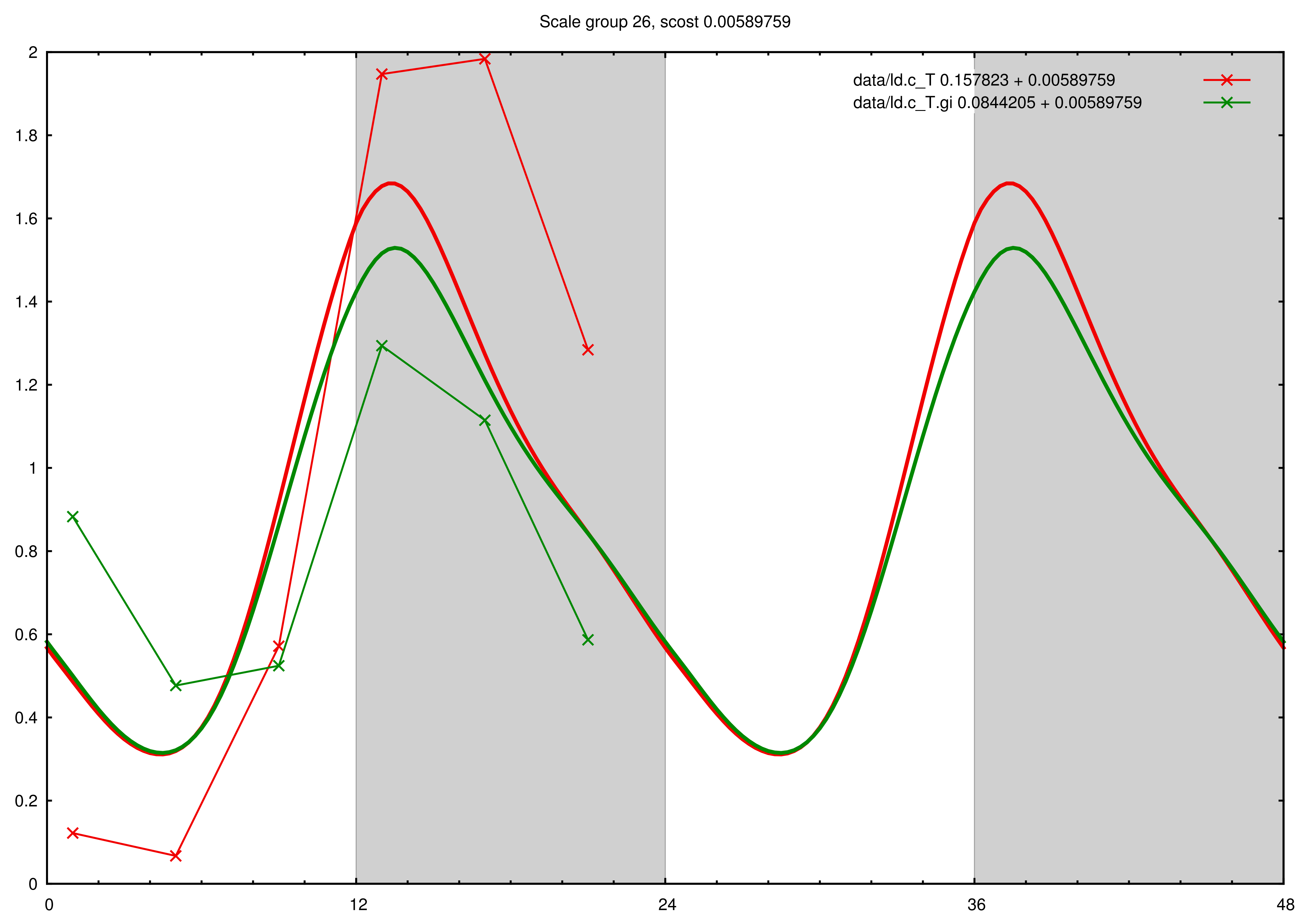
Scale group 26, scost 0.00589759
data/ld.c_T 0.157823 + 0.00589759
data/ld.c_T.gi 0.0844205 + 0.00589759
0
0.2
0.4
0.6
0.8
1
1.2
1.4
1.6
1.8
2
0
12
24
36
48

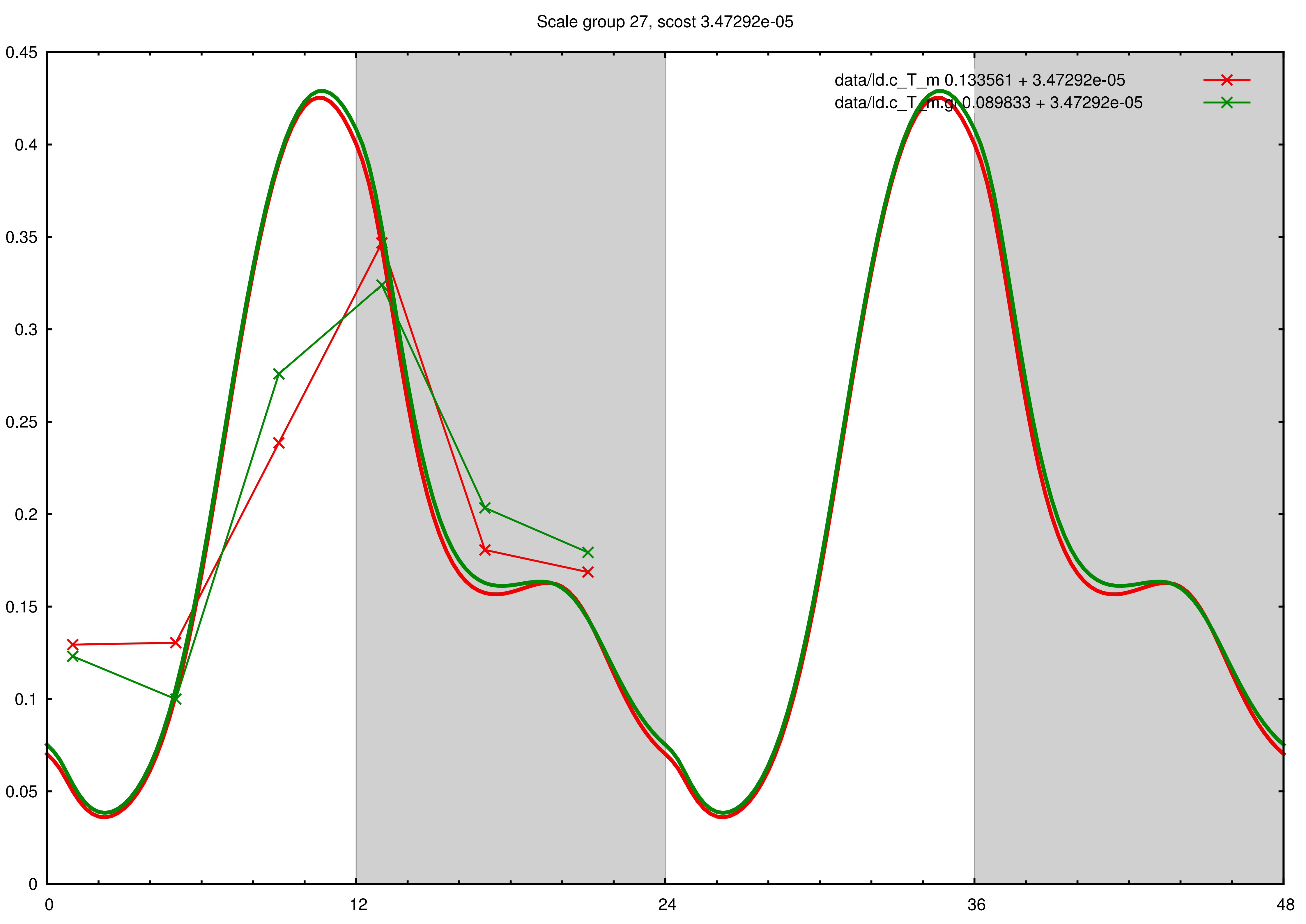
Scale group 27, scost 3.47292e-05
data/ld.c_T_m 0.133561 + 3.47292e-05
data/ld.c_T_m.gl 0.089833 + 3.47292e-05
0.45
0.4
0.35
0.3
0.25
0.2
0.15
0.1
0.05
0
0
12
24
36
48

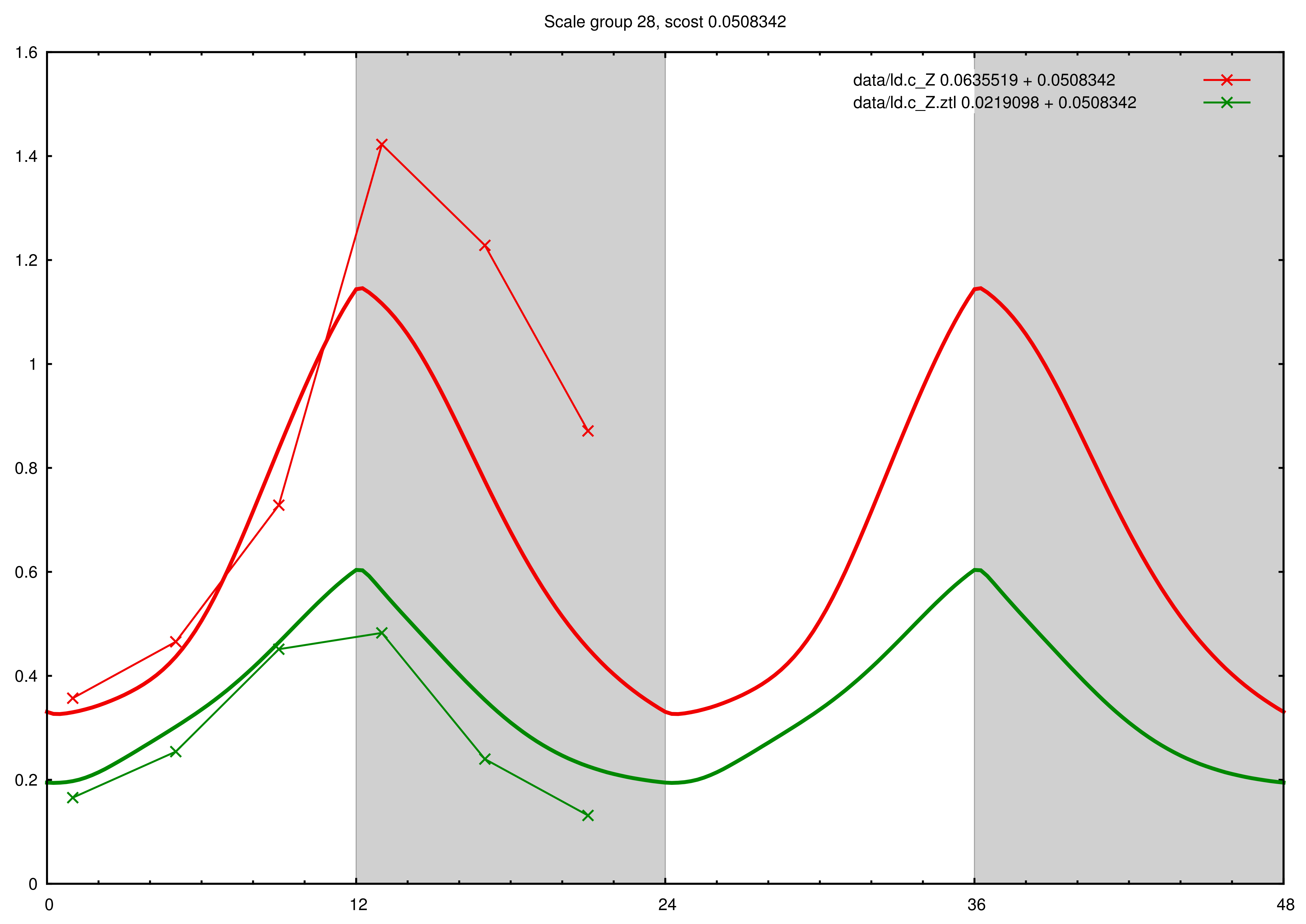

Scale group 28, scost 0.0508342
data/ld.c_Z 0.0635519 + 0.0508342
data/ld.c_Z.ztl 0.0219098 + 0.0508342
1.6
1.4
1.2
1
0.8
0.6
0.4
0.2
0
0
12
24
36
48

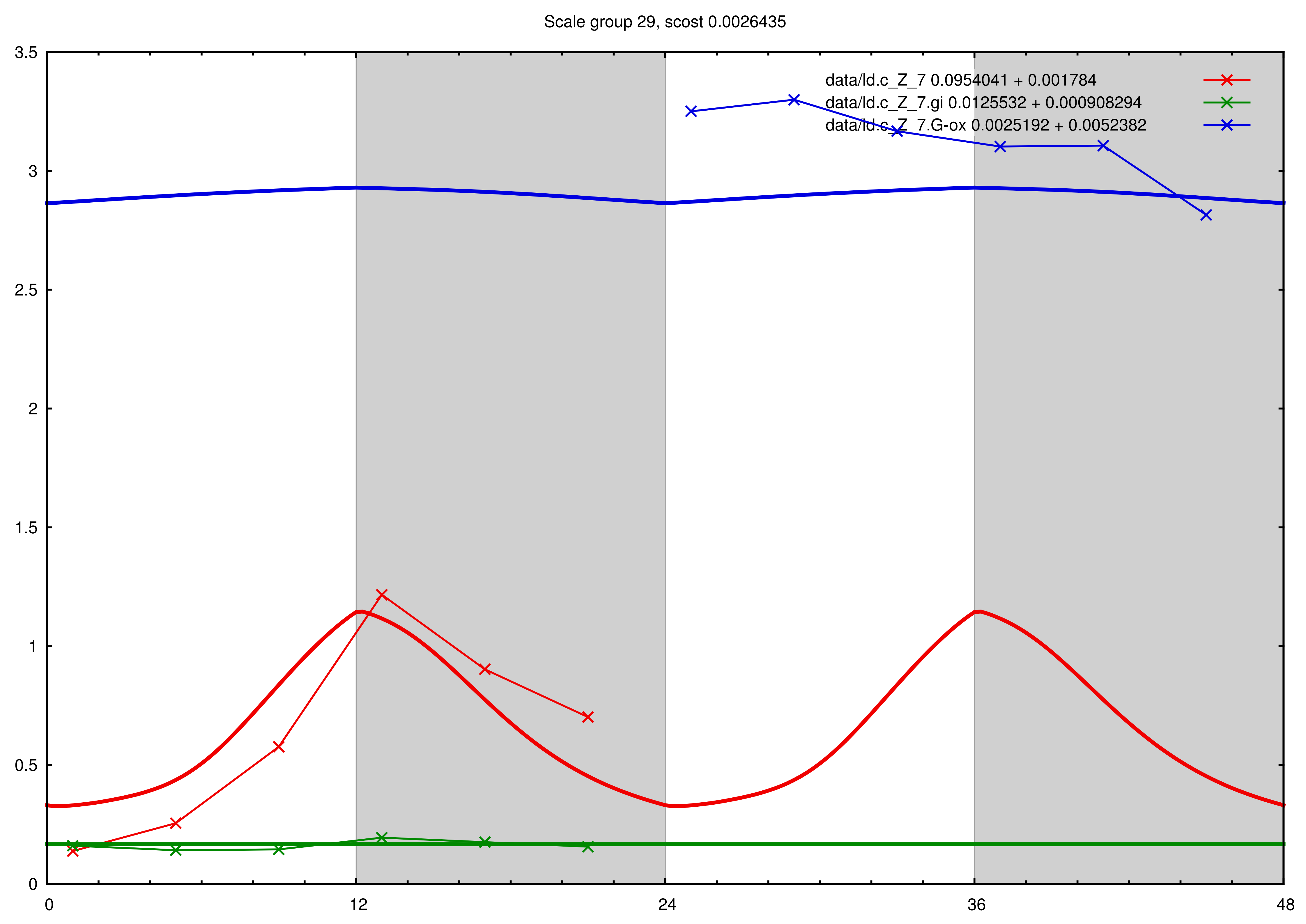

Scale group 29, scost 0.0026435
data/ld.c_Z_7 0.0954041 + 0.001784
data/ld.c_Z_7.gi 0.0125532 + 0.000908294
data/ld.c_Z_7.G-ox 0.0025192 + 0.0052382
3.5
3
2.5
2
1.5
1
0.5
0
0
12
24
36
48

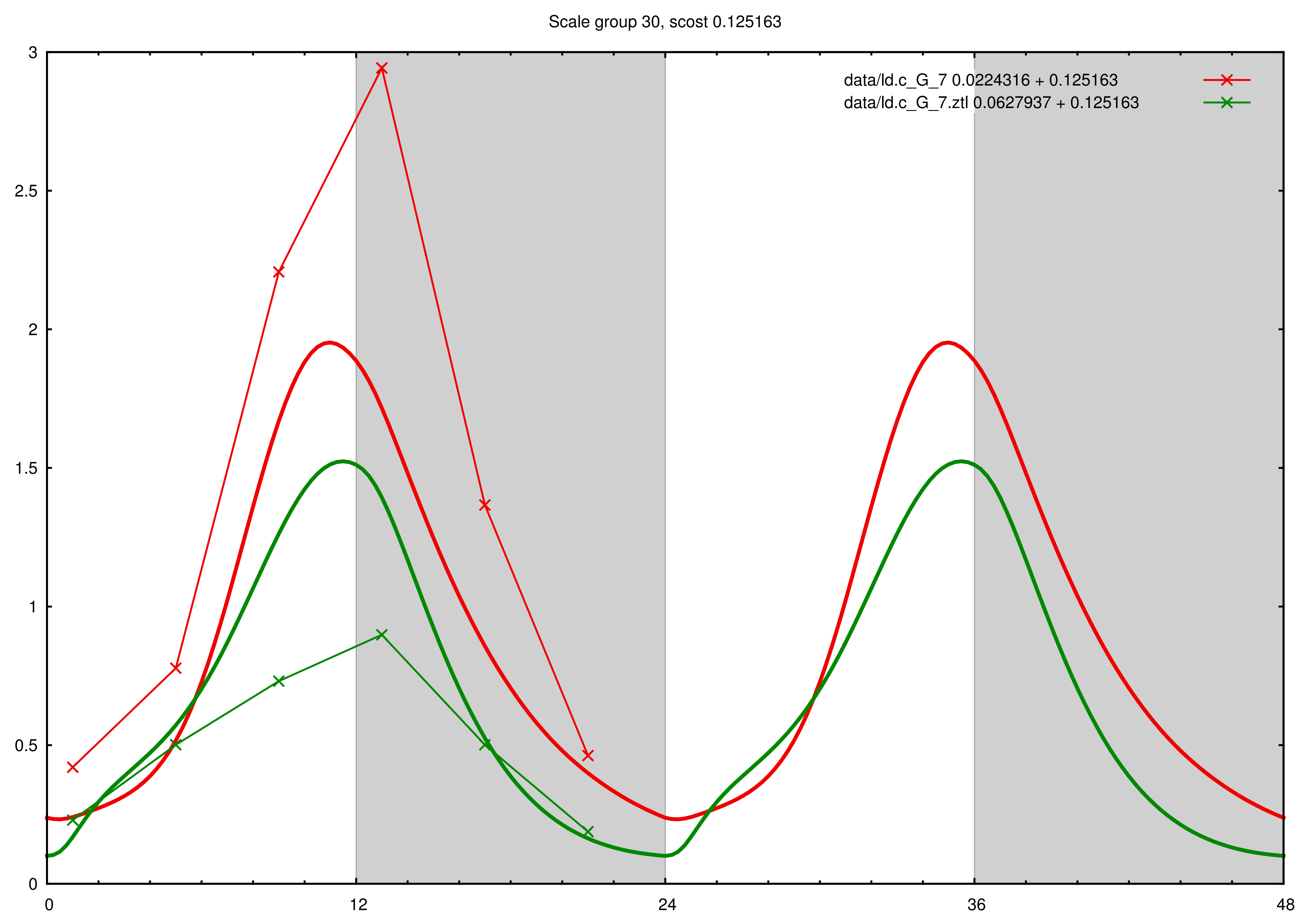
Scale group 30, scost 0.125163
data/ld.c_G_7 0.0224316 + 0.125163
data/ld.c_G_7.ztl 0.0627937 + 0.125163
3
2.5
2
1.5
1
0.5
0
0
12
24
36
48

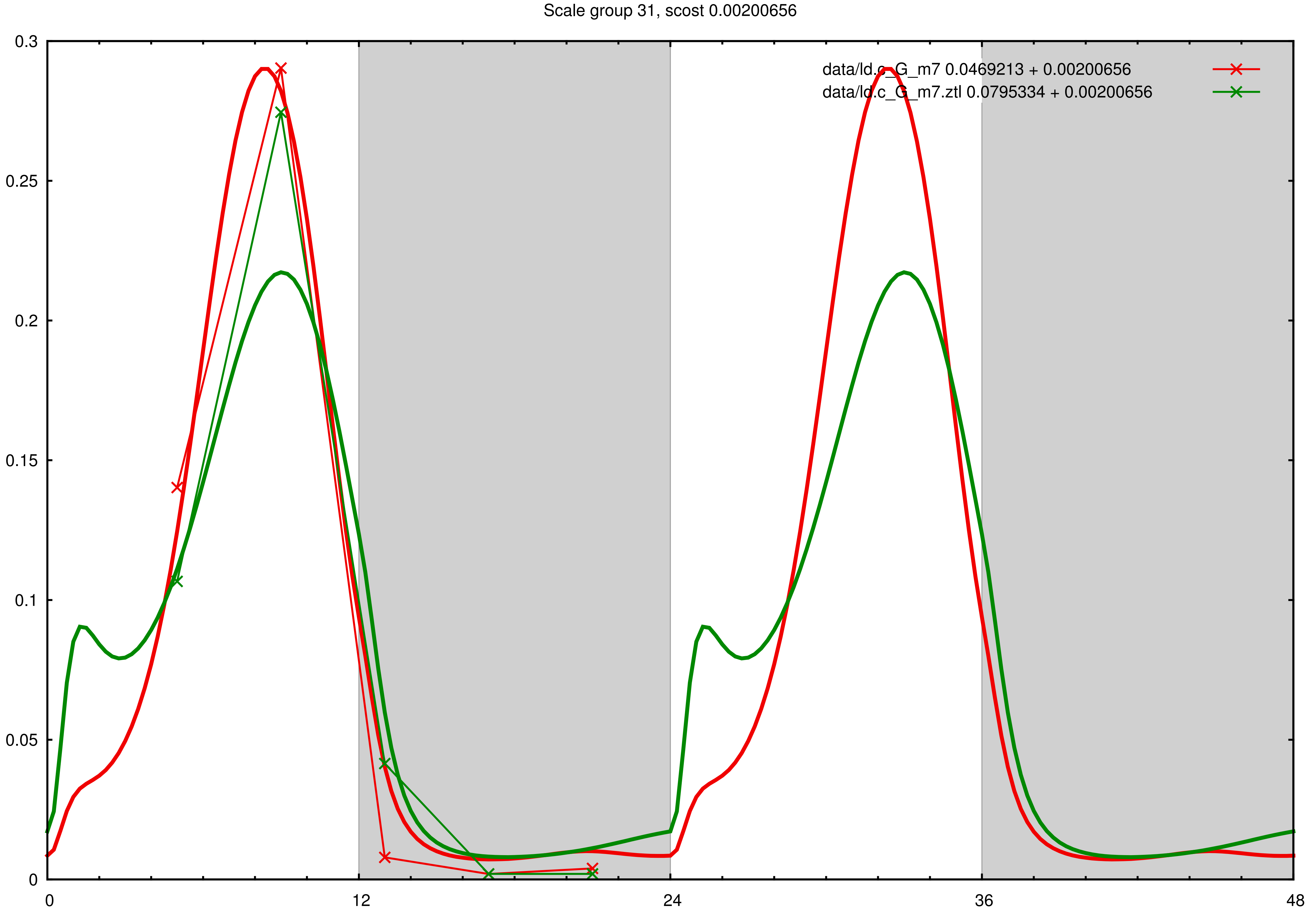
Scale group 31, scost 0.00200656
data/ld.c_G_m7 0.0469213 + 0.00200656
data/ld.c_G_m7.ztl 0.0795334 + 0.00200656
0.3
0.25
0.2
0.15
0.1
0.05
0
0
12
24
36
48

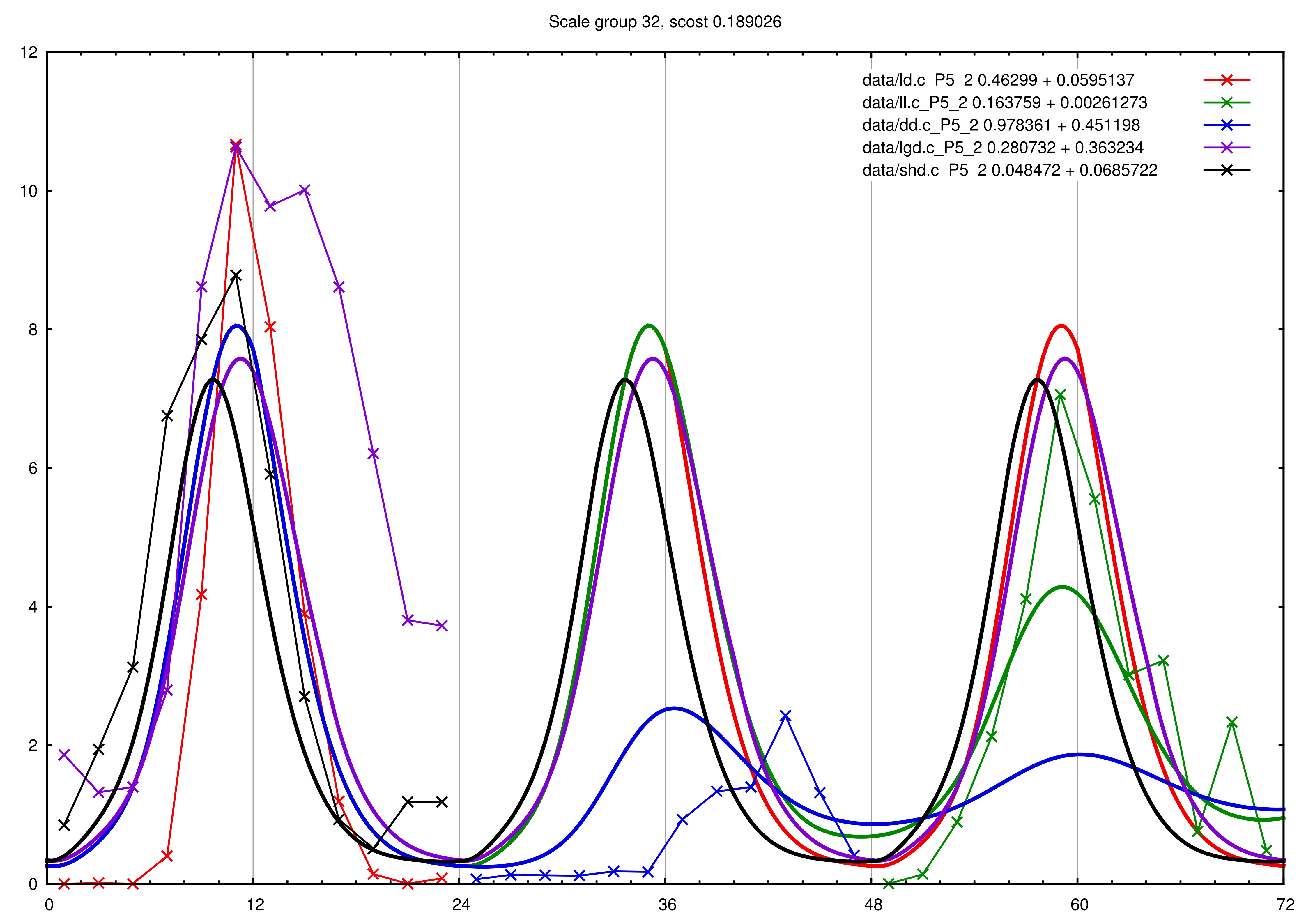

Scale group 32, scost 0.189026
data/ld.c_P5_2 0.46299 + 0.0595137
data/ll.c_P5_2 0.163759 + 0.00261273
data/dd.c_P5_2 0.978361 + 0.451198
data/lgd.c_P5_2 0.280732 + 0.363234
data/shd.c_P5_2 0.048472 + 0.0685722
0
2
4
6
8
10
12
0
12
24
36
48
60
72

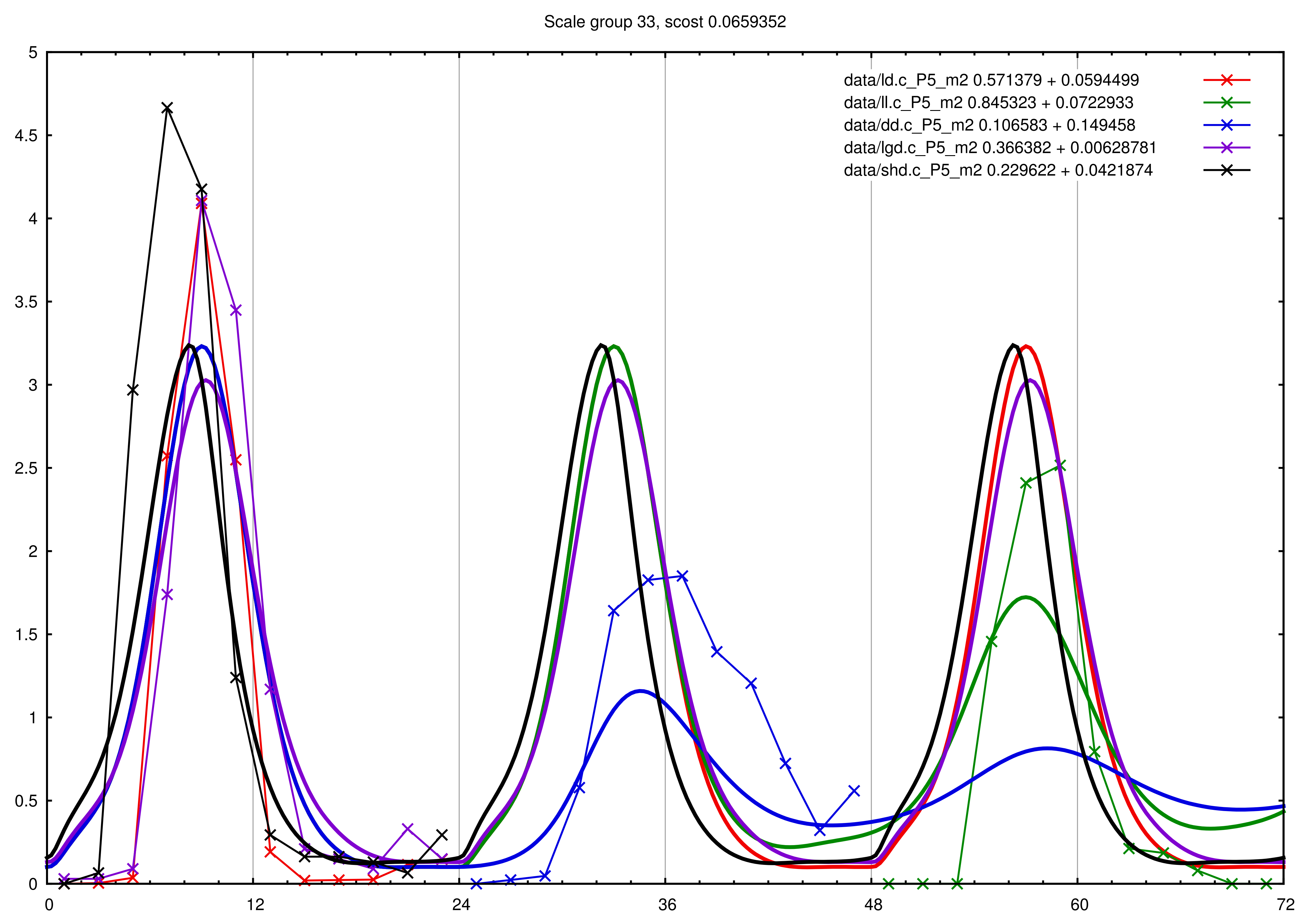

Scale group 33, scost 0.0659352
data/ld.c_P5_m2 0.571379 + 0.0594499
data/ll.c_P5_m2 0.845323 + 0.0722933
data/dd.c_P5_m2 0.106583 + 0.149458
data/lgd.c_P5_m2 0.366382 + 0.00628781
data/shd.c_P5_m2 0.229622 + 0.0421874
0
0.5
1
1.5
2
2.5
3
3.5
4
4.5
5
0
12
24
36
48
60
72

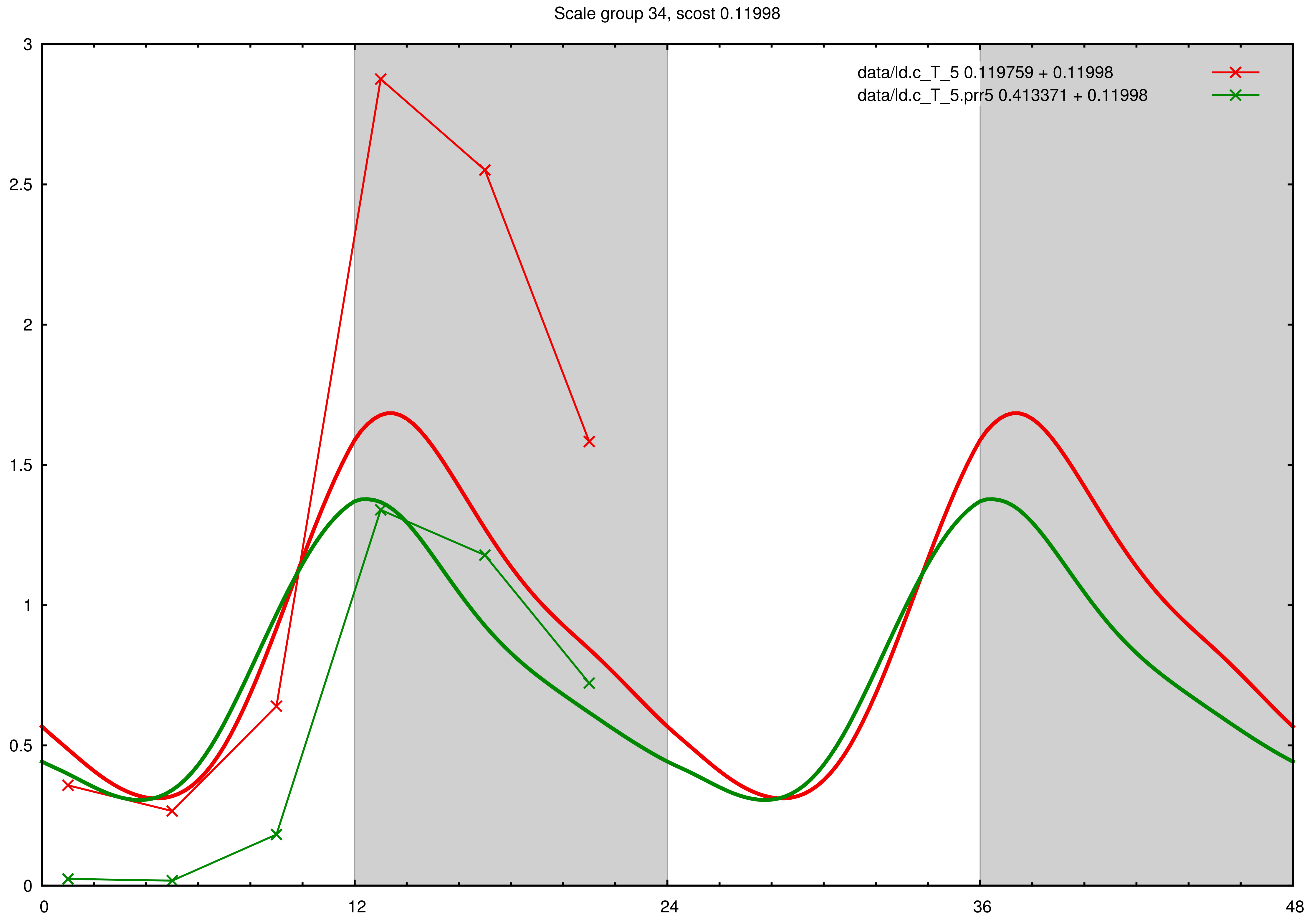
Scale group 34, scost 0.11998
data/ld.c_T_5 0.119759 + 0.11998
data/ld.c_T_5.prr5 0.413371 + 0.11998
0
0.5
1
1.5
2
2.5
3
0
12
24
36
48

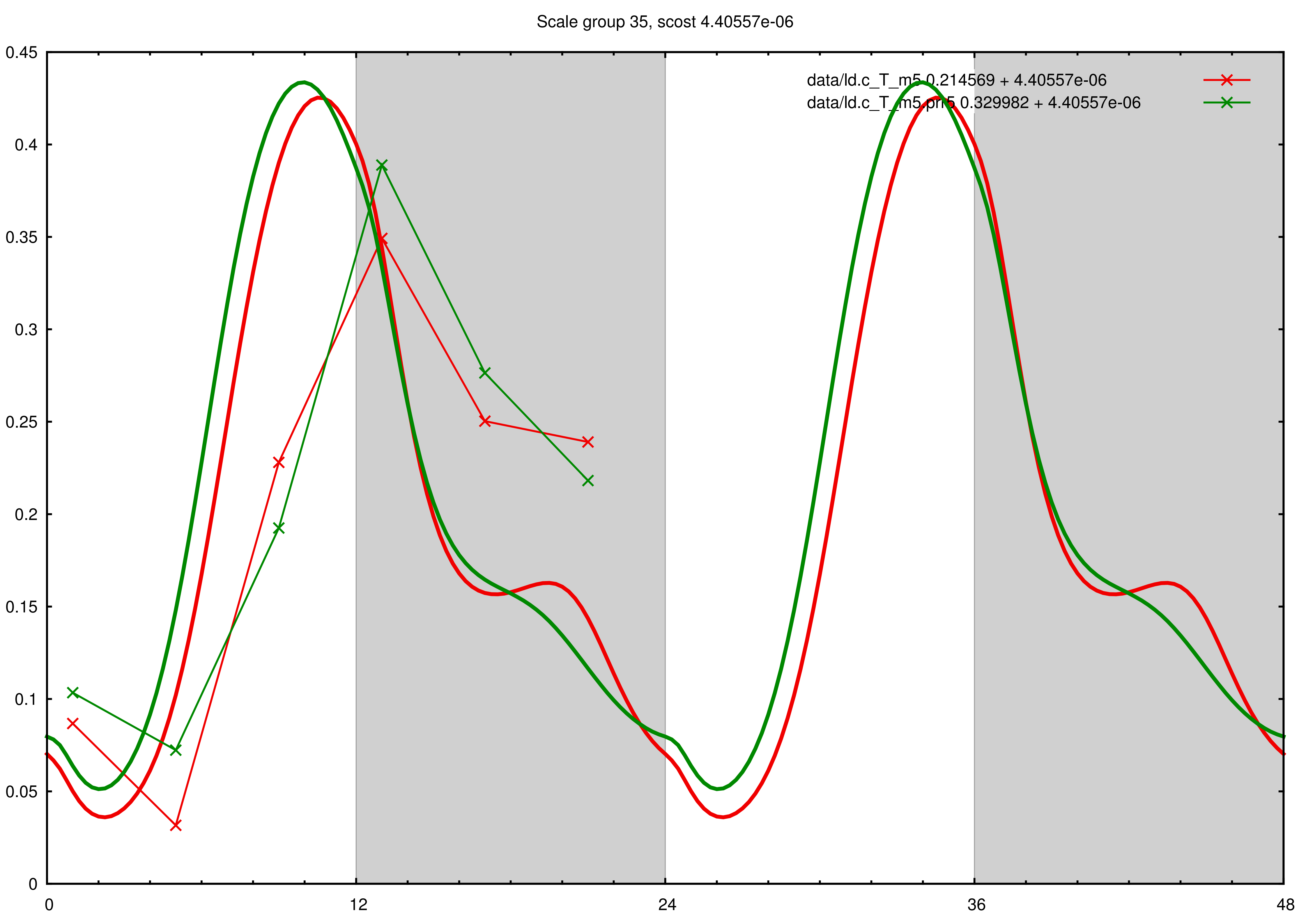

Scale group 35, scost 4.40557e-06
data/ld.c_T_m5 0.214569 + 4.40557e-06
data/ld.c_T_m5.pri5 0.329982 + 4.40557e-06
0.45
0.4
0.35
0.3
0.25
0.2
0.15
0.1
0.05
0
0
12
24
36
48

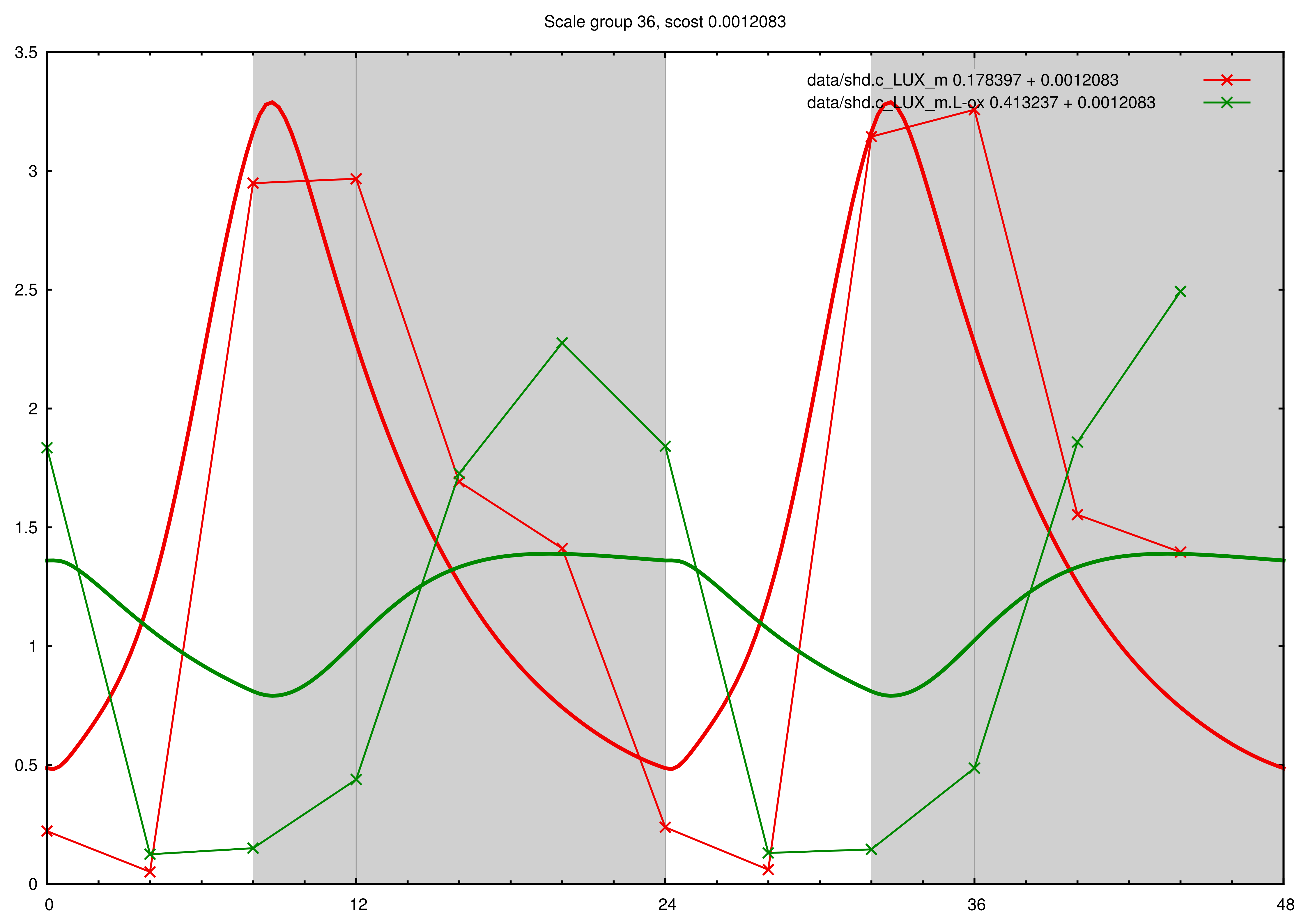
Scale group 36, scost 0.0012083
data/shd.c_LUX_m 0.178397 + 0.0012083
data/shd.c_LUX_m.L-ox 0.413237 + 0.0012083
3.5
3
2.5
2
1.5
1
0.5
0
0
12
24
36
48

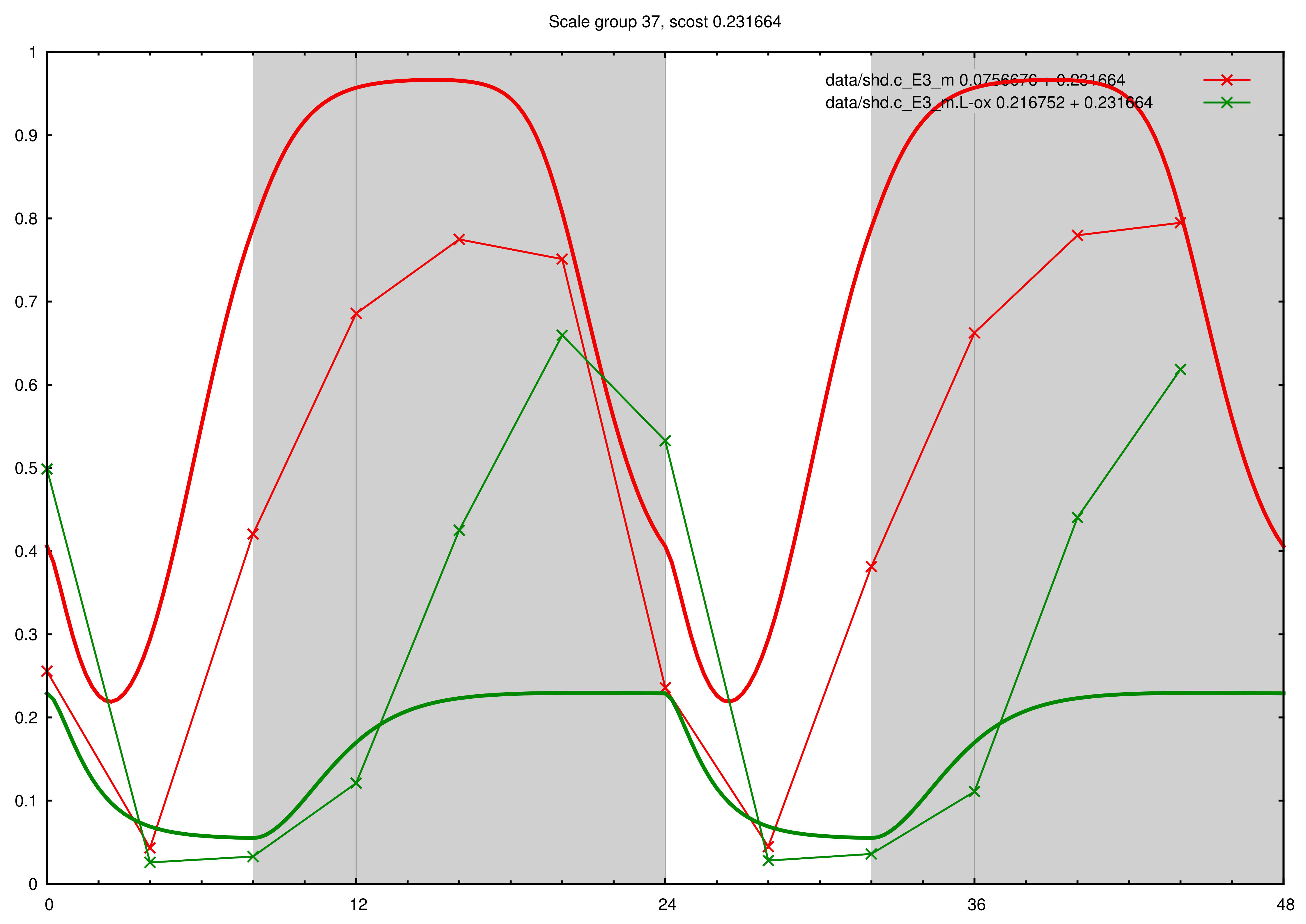
Scale group 37, scost 0.231664
data/shd.c_E3_m 0.0756676 + 0.231664
data/shd.c_E3_m.L-ox 0.216752 + 0.231664
0
0.1
0.2
0.3
0.4
0.5
0.6
0.7
0.8
0.9
1
0
12
24
36
48

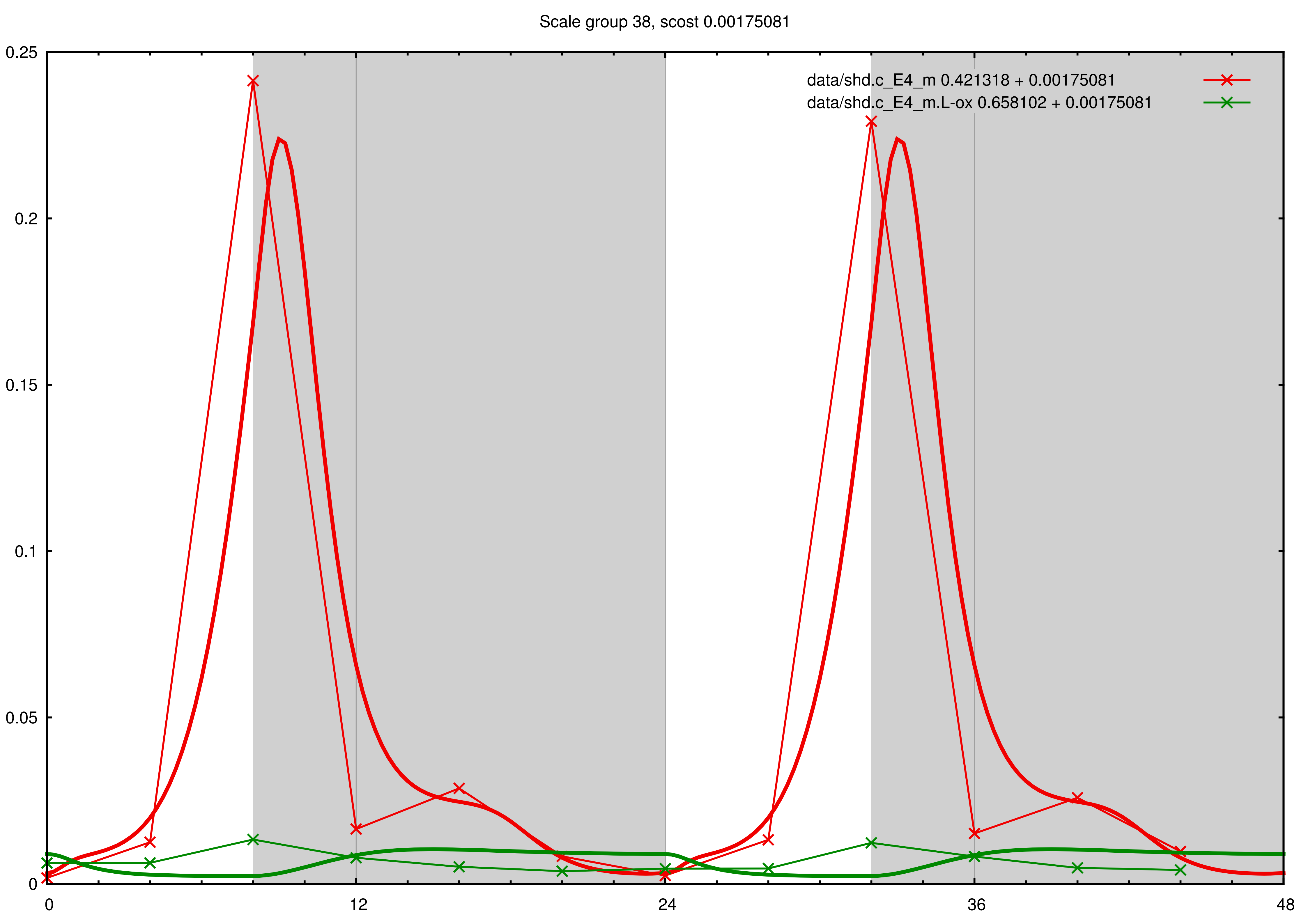

Scale group 38, scost 0.00175081
data/shd.c_E4_m 0.421318 + 0.00175081
data/shd.c_E4_m.L-ox 0.658102 + 0.00175081
0.25
0.2
0.15
0.1
0.05
0
0
12
24
36
48

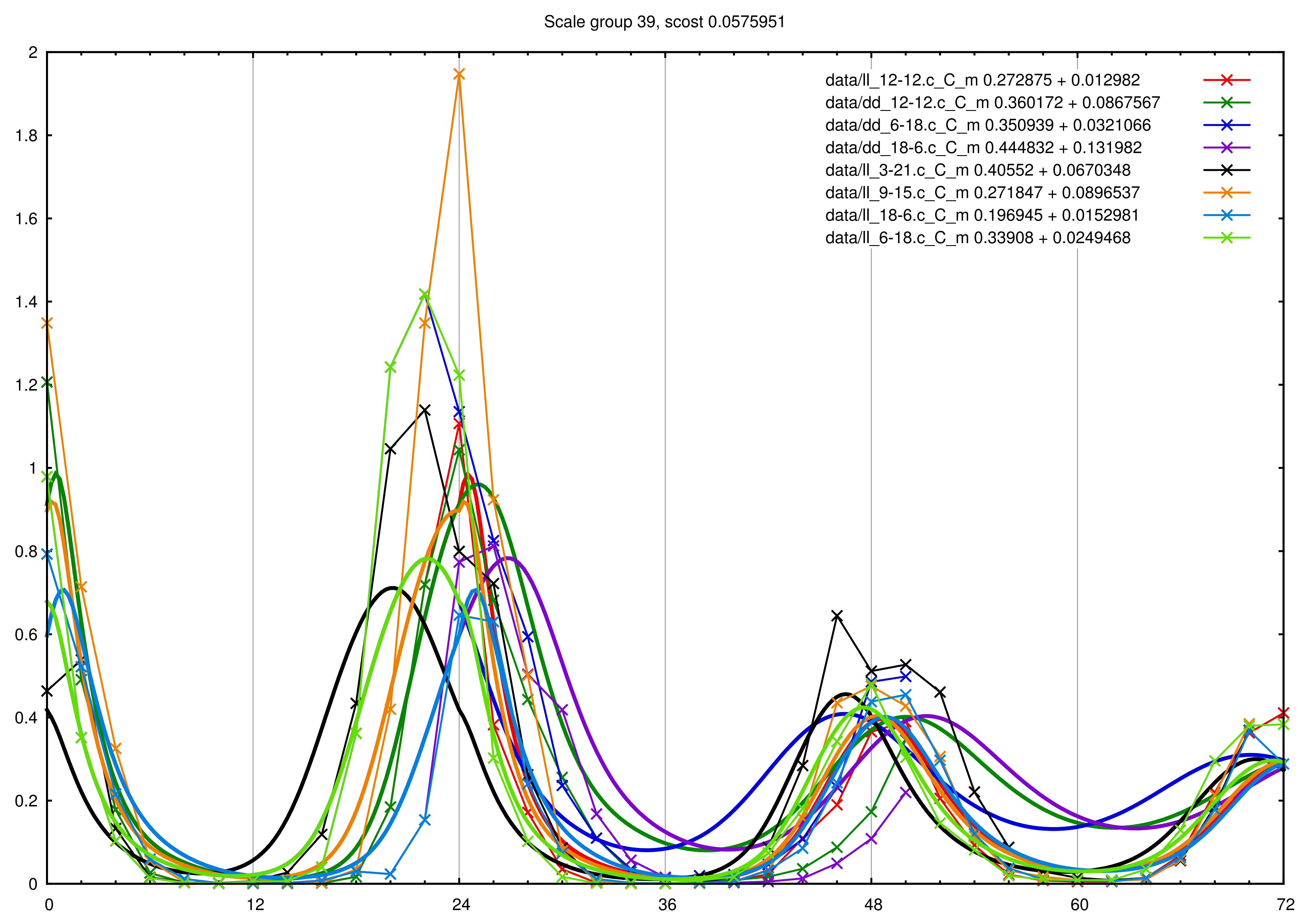

Scale group 39, scost 0.0575951
data/ll_12-12.c_C_m 0.272875 + 0.012982
data/dd_12-12.c_C_m 0.360172 + 0.0867567
data/dd_6-18.c_C_m 0.350939 + 0.0321066
data/dd_18-6.c_C_m 0.444832 + 0.131982
data/ll_3-21.c_C_m 0.40552 + 0.0670348
data/ll_9-15.c_C_m 0.271847 + 0.0896537
data/ll_18-6.c_C_m 0.196945 + 0.0152981
data/ll_6-18.c_C_m 0.33908 + 0.0249468
0
0.2
0.4
0.6
0.8
1
1.2
1.4
1.6
1.8
2
0
12
24
36
48
60
72

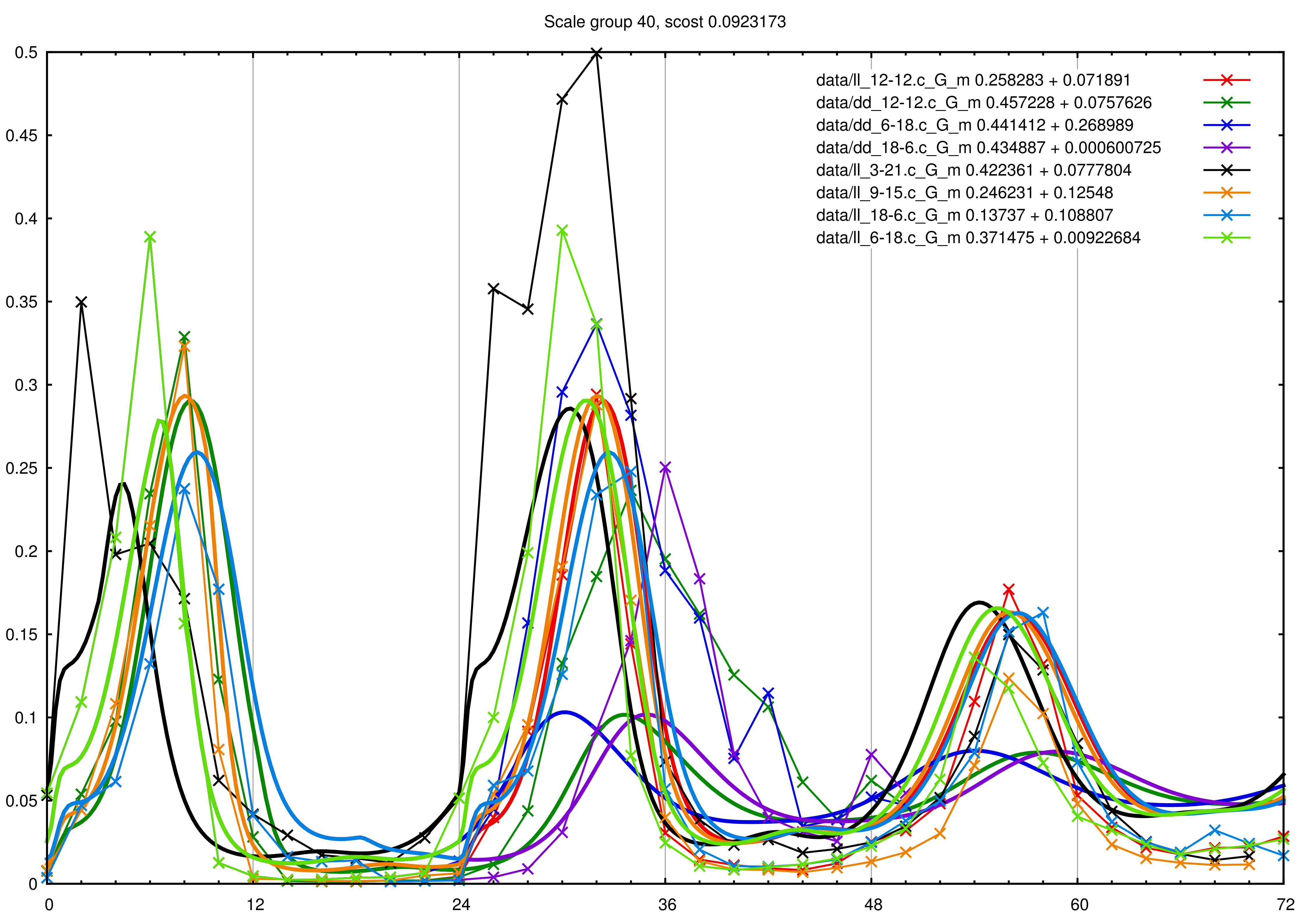
Scale group 40, scost 0.0923173
data/ll_12-12.c_G_m 0.258283 + 0.071891
data/dd_12-12.c_G_m 0.457228 + 0.0757626
data/dd_6-18.c_G_m 0.441412 + 0.268989
data/dd_18-6.c_G_m 0.434887 + 0.000600725
data/ll_3-21.c_G_m 0.422361 + 0.0777804
data/ll_9-15.c_G_m 0.246231 + 0.12548
data/ll_18-6.c_G_m 0.13737 + 0.108807
data/ll_6-18.c_G_m 0.371475 + 0.00922684
0.5
0.45
0.4
0.35
0.3
0.25
0.2
0.15
0.1
0.05
0
0
12
24
36
48
60
72

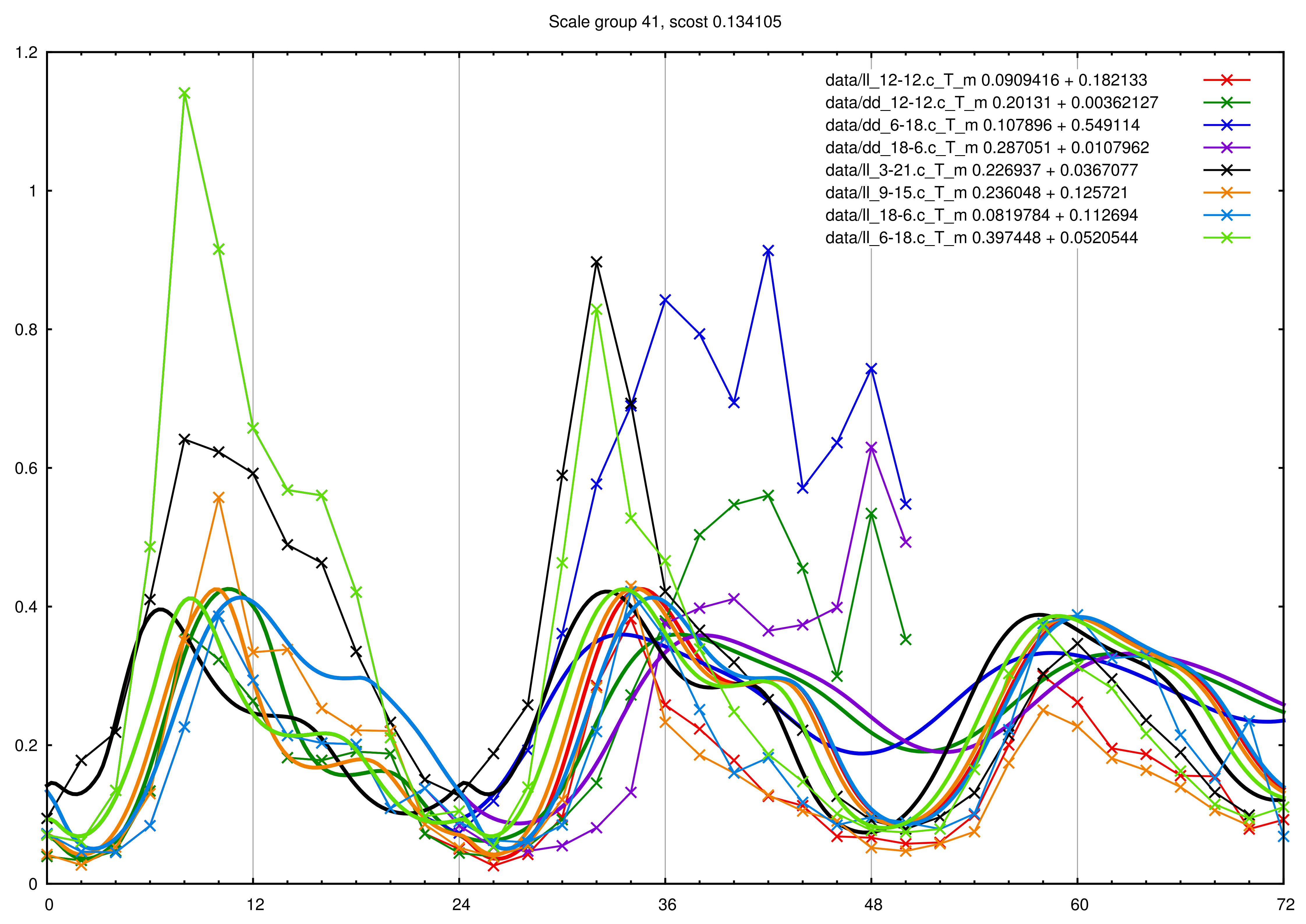

Scale group 41, scost 0.134105
data/ll_12-12.c_T_m 0.0909416 + 0.182133
data/dd_12-12.c_T_m 0.20131 + 0.00362127
data/dd_6-18.c_T_m 0.107896 + 0.549114
data/dd_18-6.c_T_m 0.287051 + 0.0107962
data/ll_3-21.c_T_m 0.226937 + 0.0367077
data/ll_9-15.c_T_m 0.236048 + 0.125721
data/ll_18-6.c_T_m 0.0819784 + 0.112694
data/ll_6-18.c_T_m 0.397448 + 0.0520544
1.2
1
0.8
0.6
0.4
0.2
0
0
12
24
36
48
60
72

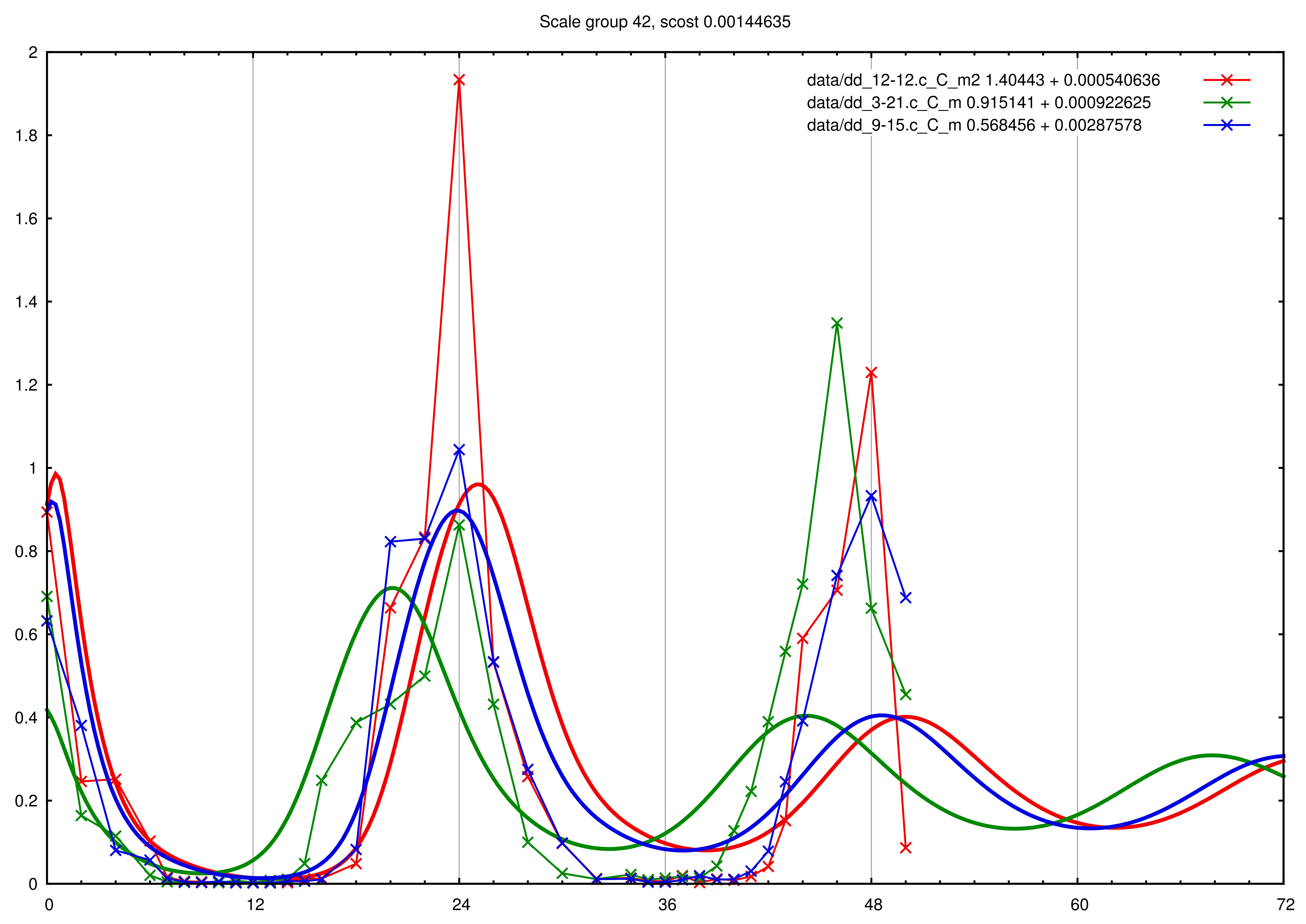
Scale group 42, scost 0.00144635
data/dd_12-12.c_C_m2 1.40443 + 0.000540636
data/dd_3-21.c_C_m 0.915141 + 0.000922625
data/dd_9-15.c_C_m 0.568456 + 0.00287578
0
0.2
0.4
0.6
0.8
1
1.2
1.4
1.6
1.8
2
0
12
24
36
48
60
72

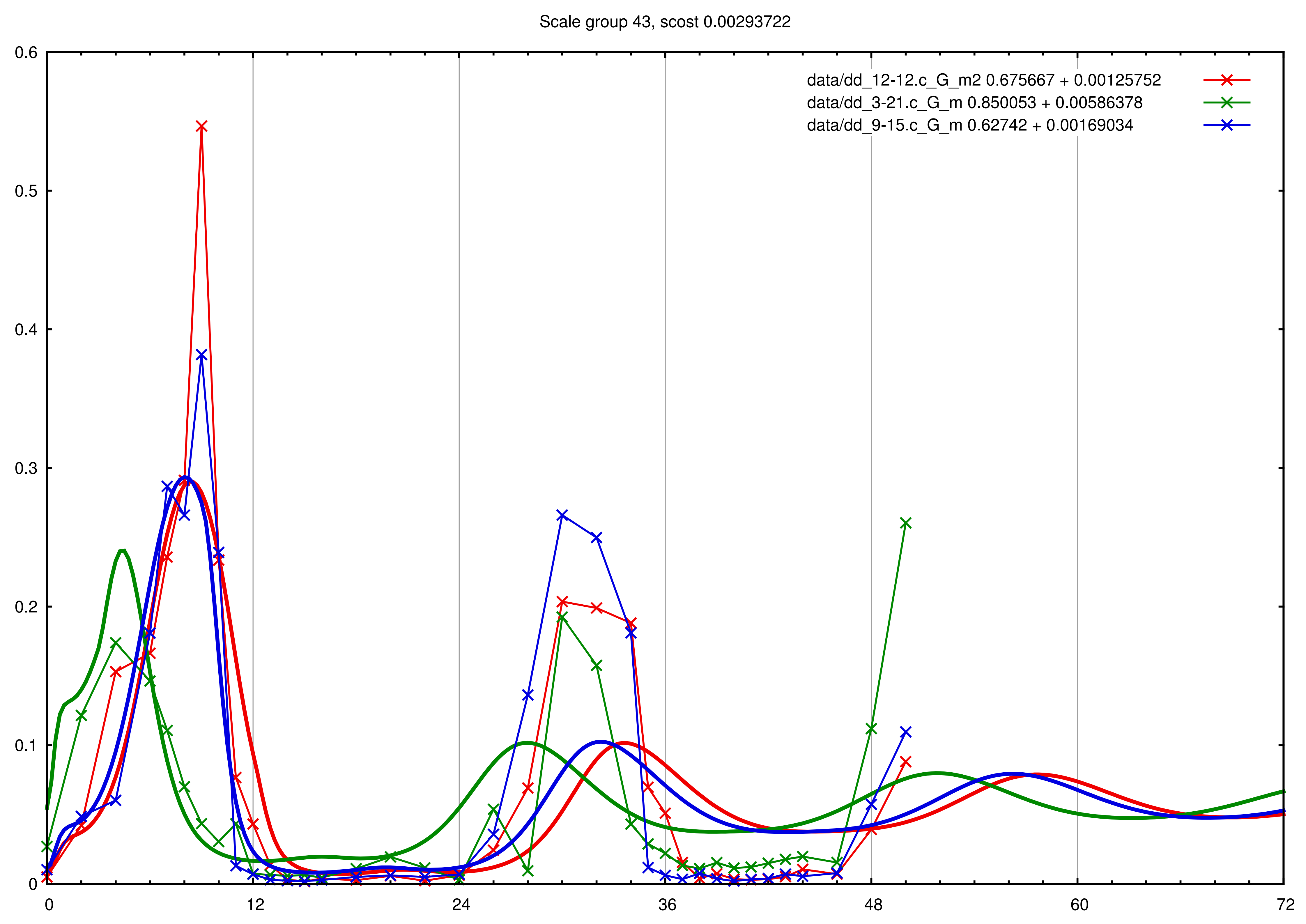

Scale group 43, scost 0.00293722
data/dd_12-12.c_G_m2 0.675667 + 0.00125752
data/dd_3-21.c_G_m 0.850053 + 0.00586378
data/dd_9-15.c_G_m 0.62742 + 0.00169034
0.6
0.5
0.4
0.3
0.2
0.1
0
0
12
24
36
48
60
72

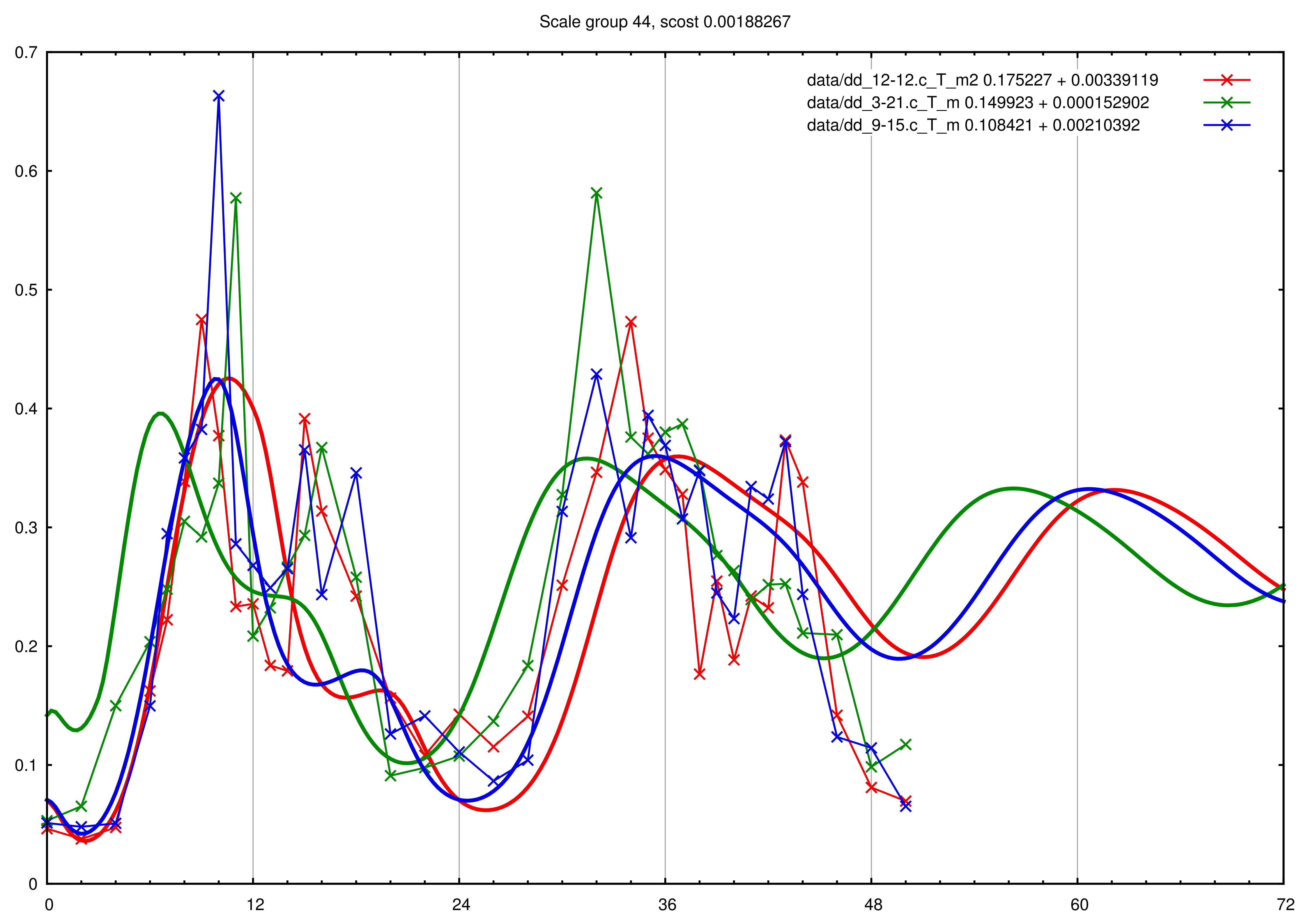

Scale group 44, scost 0.00188267
data/dd_12-12.c_T_m2 0.175227 + 0.00339119
data/dd_3-21.c_T_m 0.149923 + 0.000152902
data/dd_9-15.c_T_m 0.108421 + 0.00210392
0
0.1
0.2
0.3
0.4
0.5
0.6
0.7
0
12
24
36
48
60
72

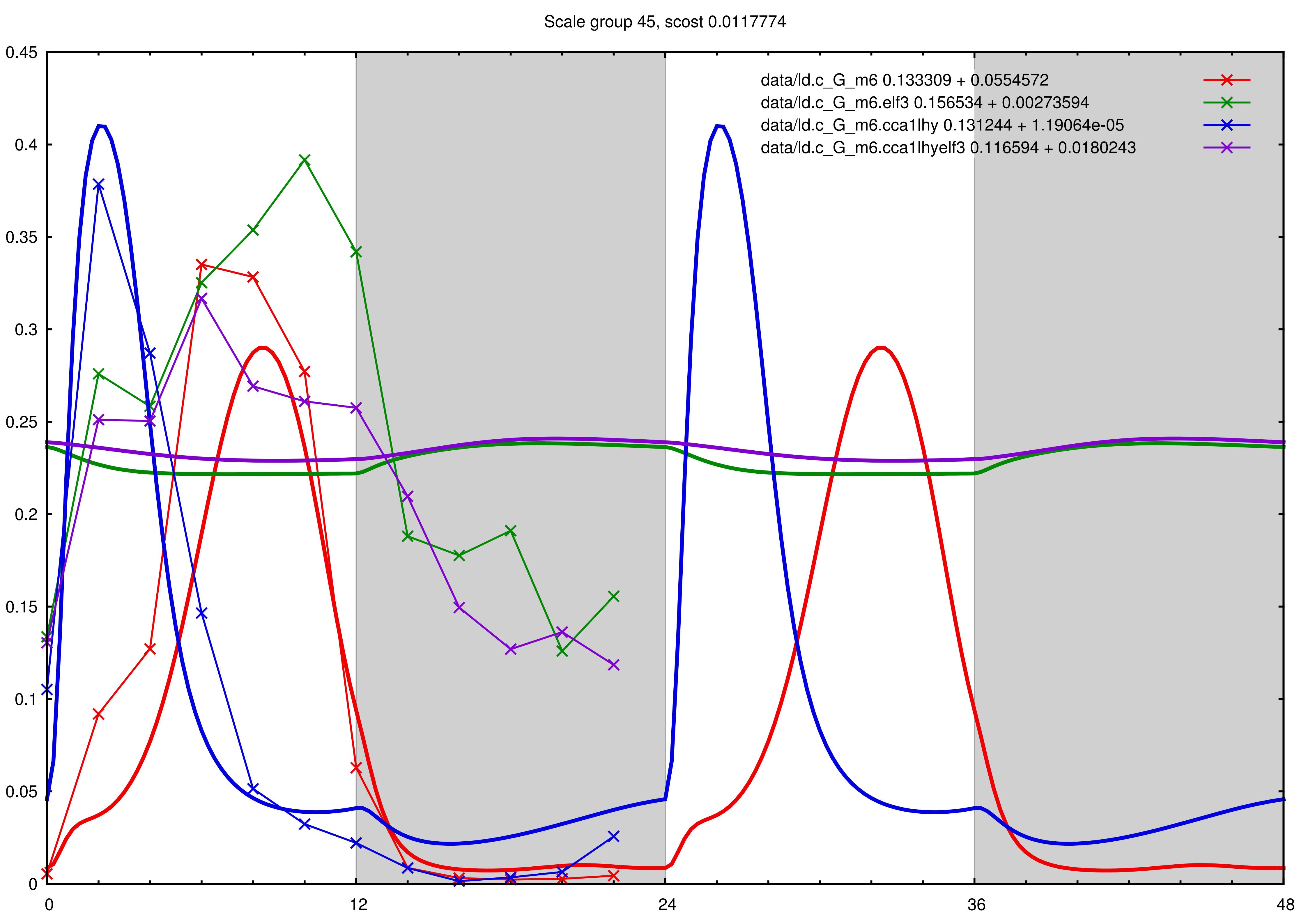
Scale group 45, scost 0.0117774
data/ld.c_G_m6 0.133309 + 0.0554572
data/ld.c_G_m6.elf3 0.156534 + 0.00273594
data/ld.c_G_m6.cca1lhy 0.131244 + 1.19064e-05
data/ld.c_G_m6.cca1lhyelf3 0.116594 + 0.0180243
0.45
0.4
0.35
0.3
0.25
0.2
0.15
0.1
0.05
0
0
12
24
36
48

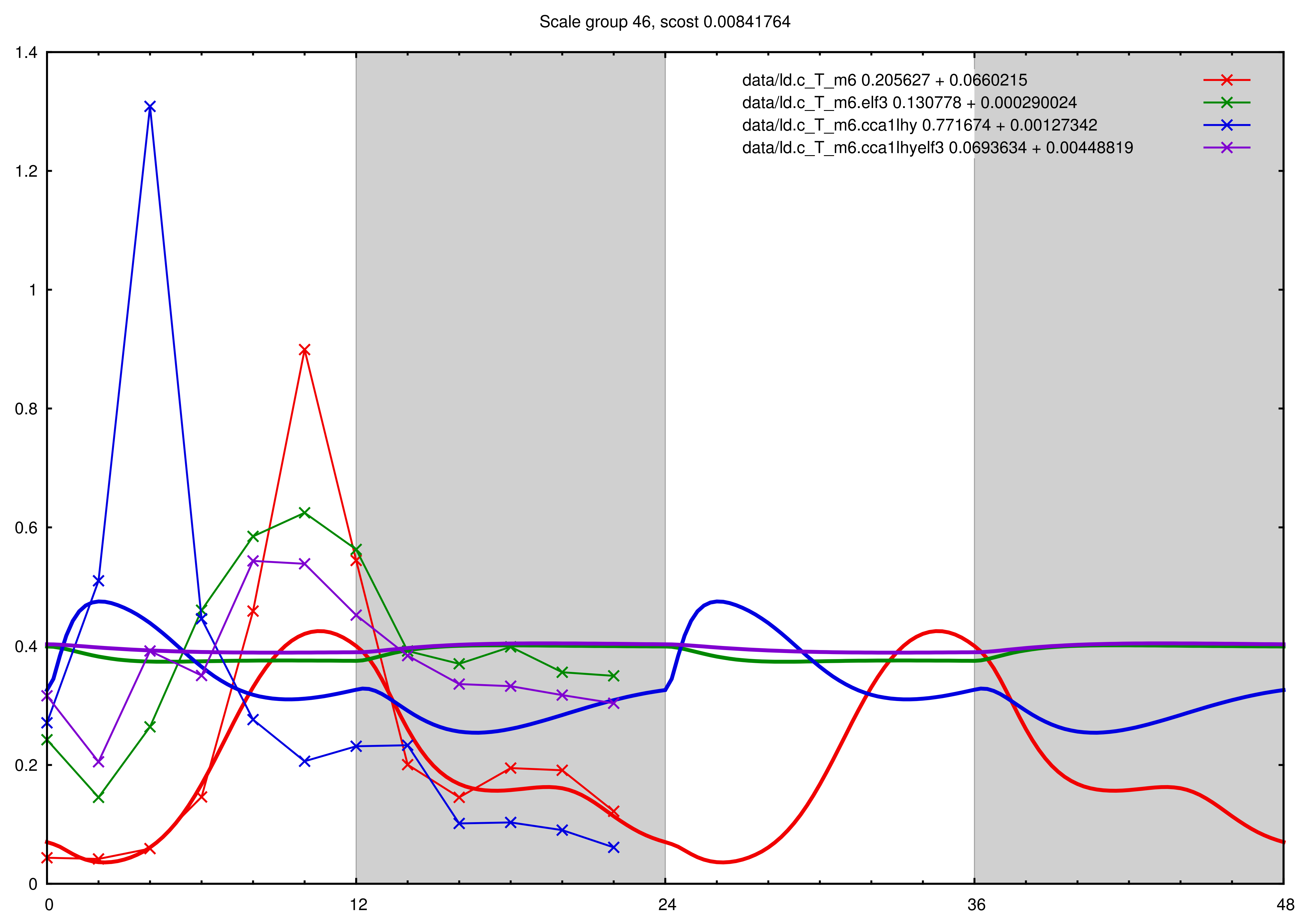

Scale group 46, scost 0.00841764
data/ld.c_T_m6 0.205627 + 0.0660215
data/ld.c_T_m6.elf3 0.130778 + 0.000290024
data/ld.c_T_m6.cca1lhy 0.771674 + 0.00127342
data/ld.c_T_m6.cca1lhyelf3 0.0693634 + 0.00448819
0
0.2
0.4
0.6
0.8
1
1.2
1.4
0
12
24
36
48

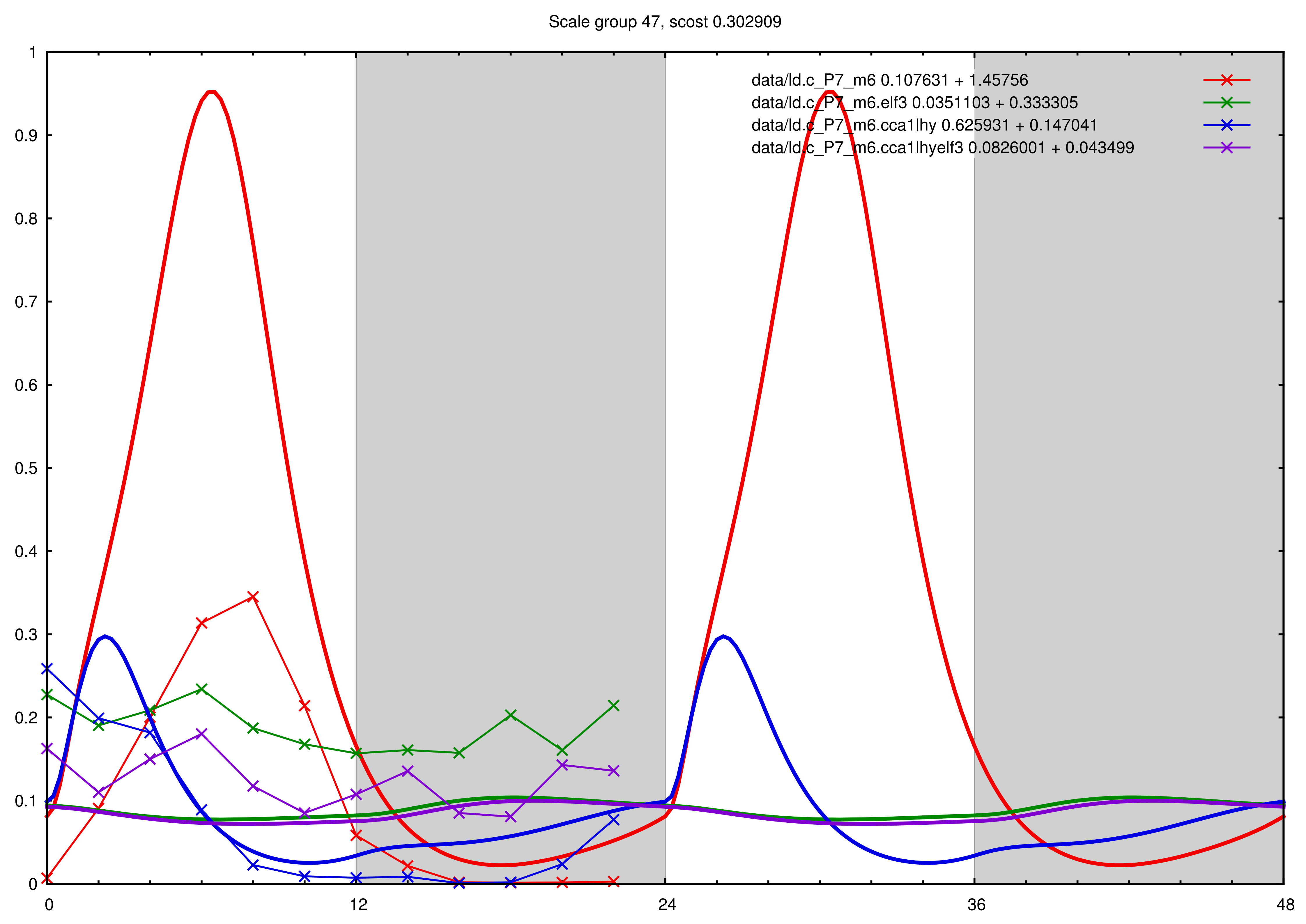
Scale group 47, scost 0.302909
data/ld.c_P7_m6 0.107631 + 1.45756
data/ld.c_P7_m6.elf3 0.0351103 + 0.333305
data/ld.c_P7_m6.cca1lhy 0.625931 + 0.147041
data/ld.c_P7_m6.cca1lhyelf3 0.0826001 + 0.043499
0
0.1
0.2
0.3
0.4
0.5
0.6
0.7
0.8
0.9
1
0
12
24
36
48

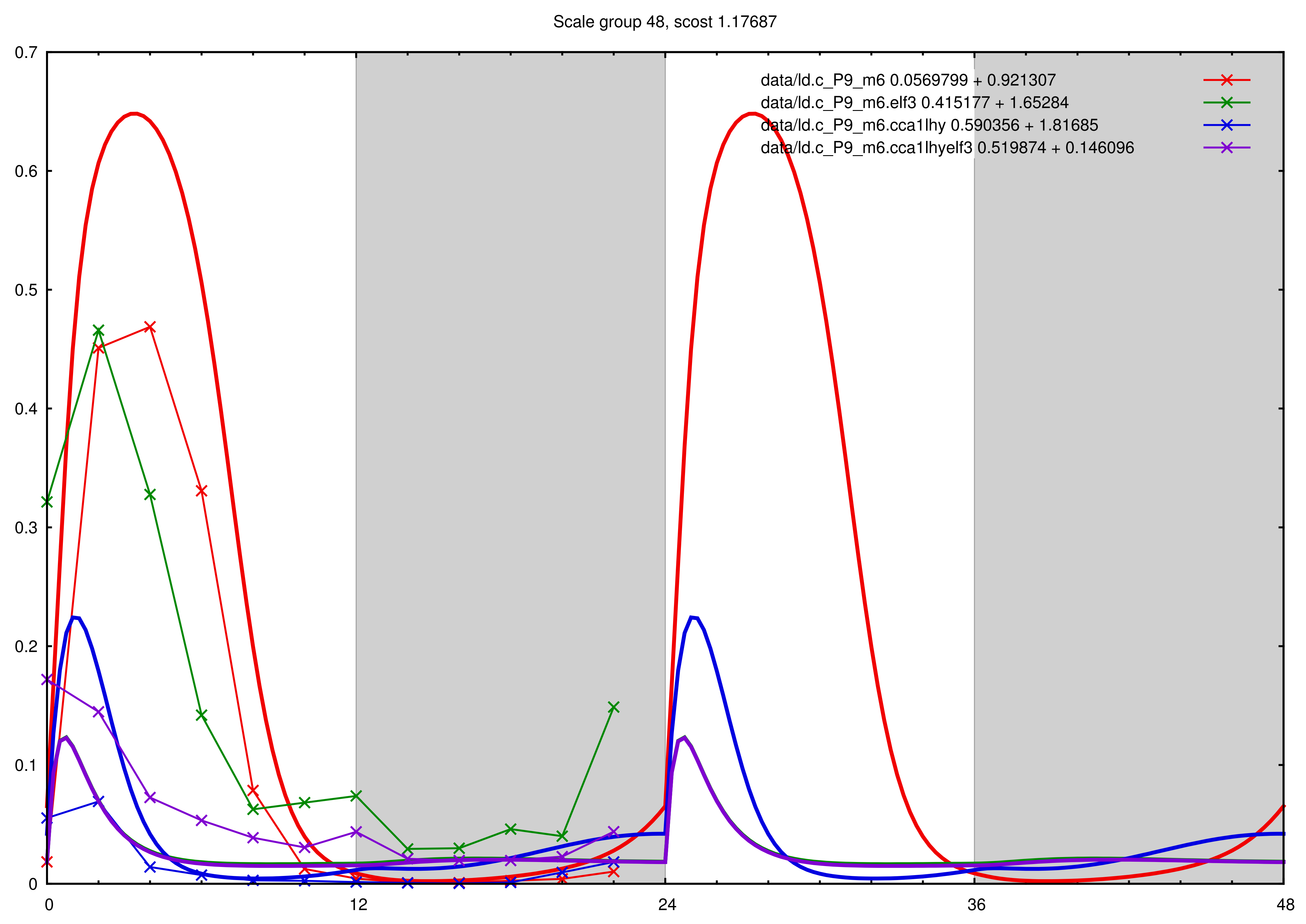

Scale group 48, scost 1.17687
data/ld.c_P9_m6 0.0569799 + 0.921307
data/ld.c_P9_m6.elf3 0.415177 + 1.65284
data/ld.c_P9_m6.cca1lhy 0.590356 + 1.81685
data/ld.c_P9_m6.cca1lhyelf3 0.519874 + 0.146096
0.7
0.6
0.5
0.4
0.3
0.2
0.1
0
0
12
24
36
48

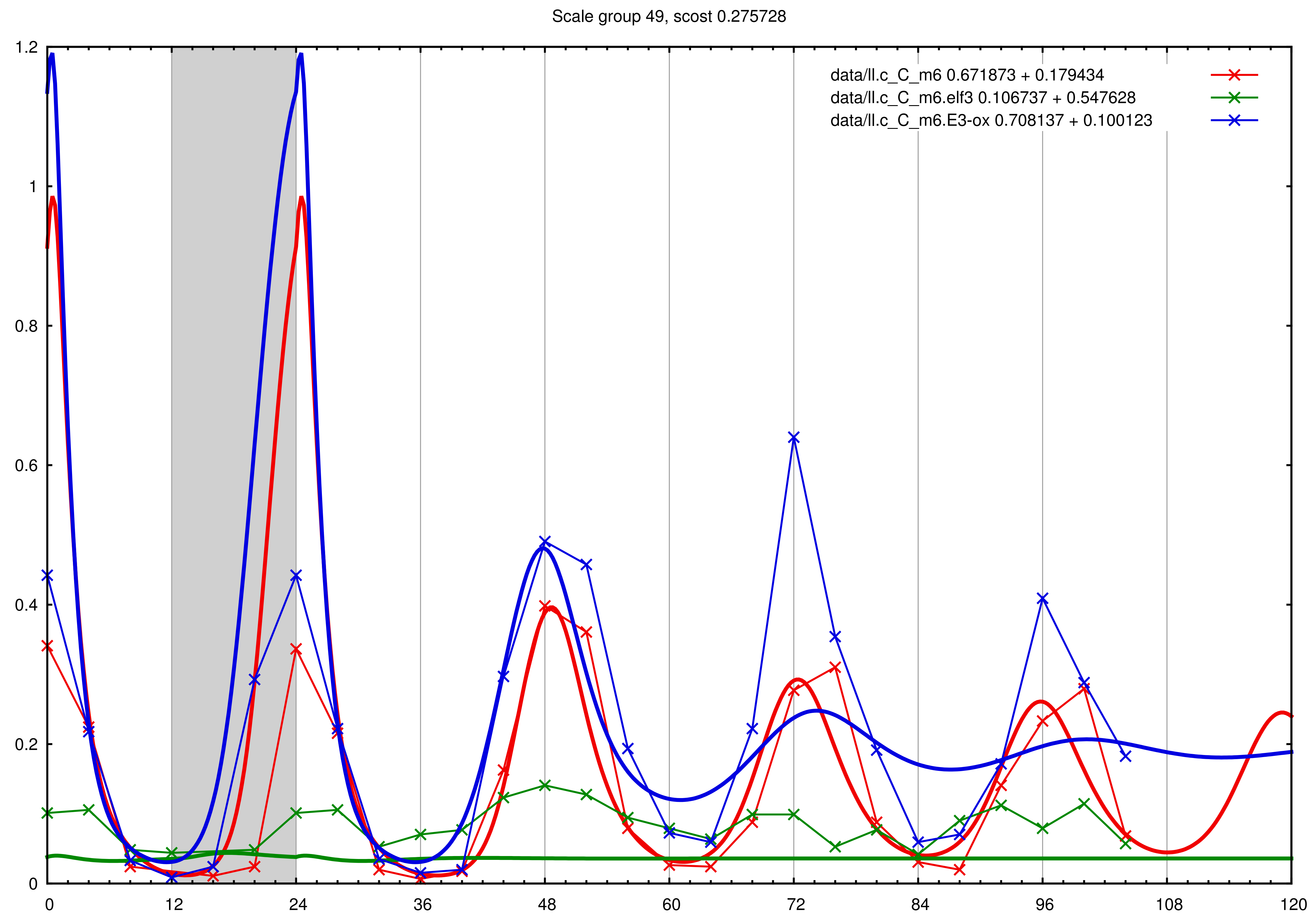

Scale group 49, scost 0.275728
data/ll.c_C_m6 0.671873 + 0.179434
data/ll.c_C_m6.elf3 0.106737 + 0.547628
data/ll.c_C_m6.E3-ox 0.708137 + 0.100123
0
0.2
0.4
0.6
0.8
1
1.2
0
12
24
36
48
60
72
84
96
108
120

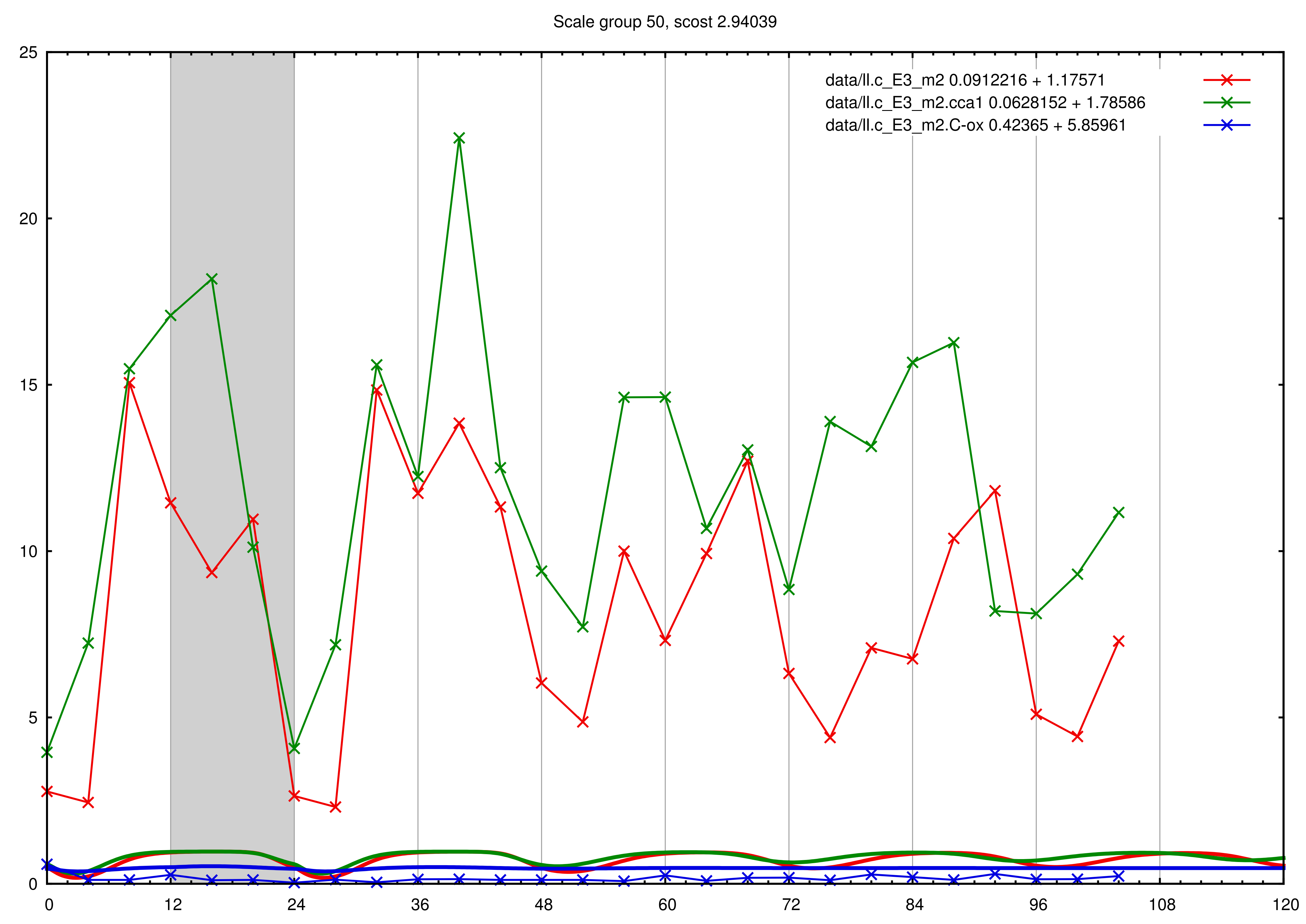

Scale group 50, scost 2.94039
data/ll.c_E3_m2 0.0912216 + 1.17571
data/ll.c_E3_m2.cca1 0.0628152 + 1.78586
data/ll.c_E3_m2.C-ox 0.42365 + 5.85961
0
5
10
15
20
25
0
12
24
36
48
60
72
84
96
108
120

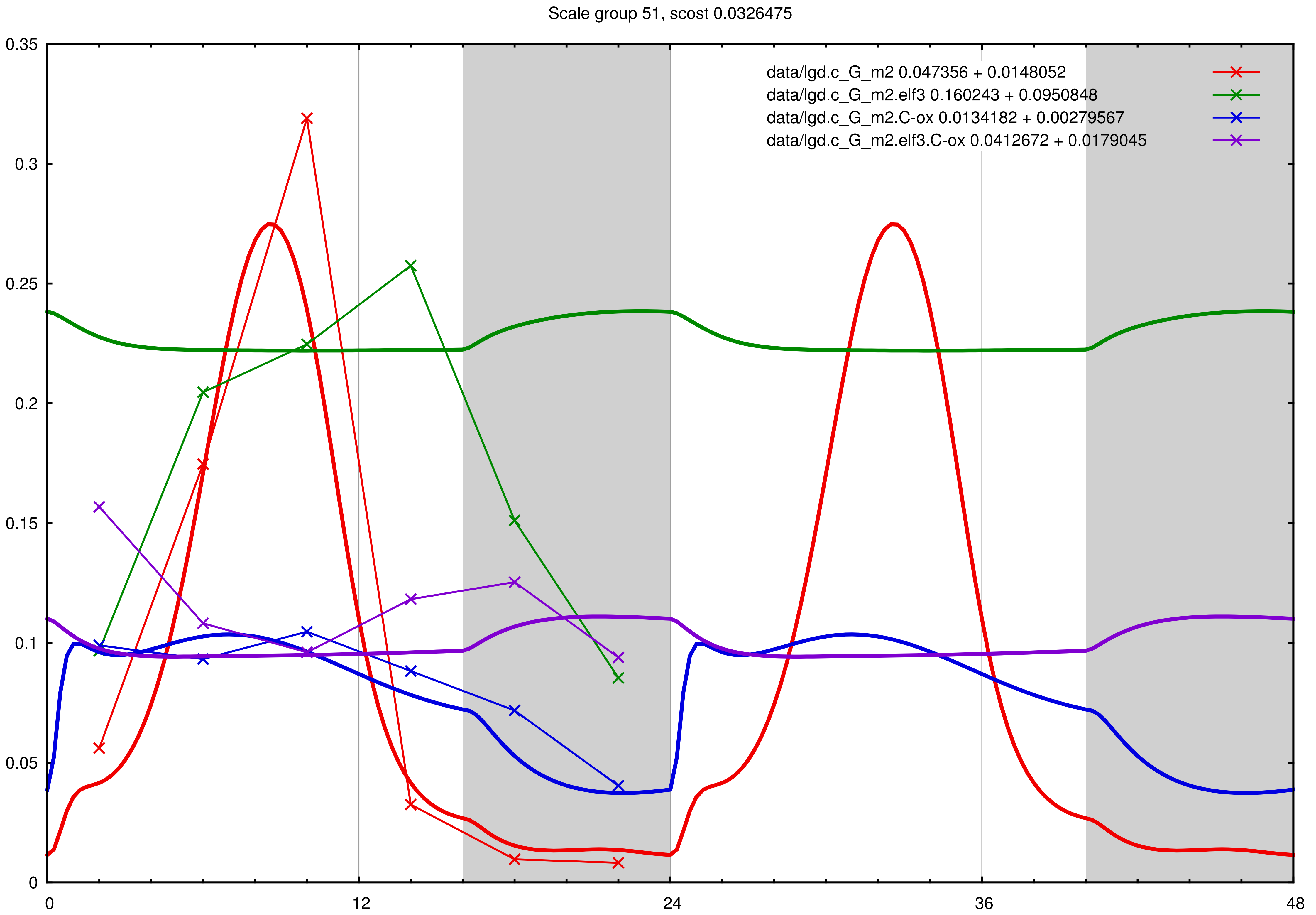

Scale group 51, scost 0.0326475
data/lgd.c_G_m2 0.047356 + 0.0148052
data/lgd.c_G_m2.elf3 0.160243 + 0.0950848
data/lgd.c_G_m2.C-ox 0.0134182 + 0.00279567
data/lgd.c_G_m2.elf3.C-ox 0.0412672 + 0.0179045
0.35
0.3
0.25
0.2
0.15
0.1
0.05
0
0
12
24
36
48

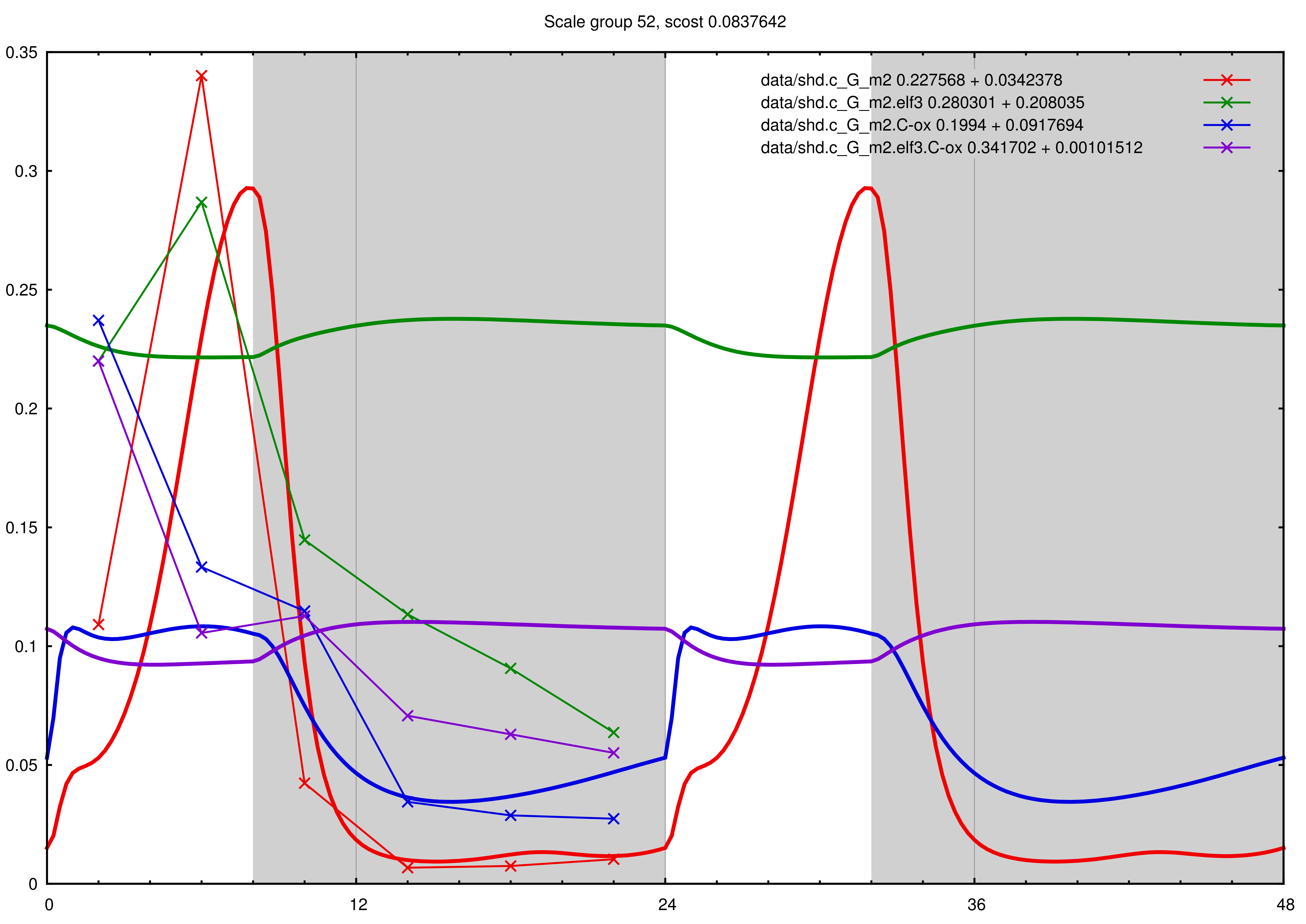
Scale group 52, scost 0.0837642
data/shd.c_G_m2 0.227568 + 0.0342378
data/shd.c_G_m2.elf3 0.280301 + 0.208035
data/shd.c_G_m2.C-ox 0.1994 + 0.0917694
data/shd.c_G_m2.elf3.C-ox 0.341702 + 0.00101512
0.35
0.3
0.25
0.2
0.15
0.1
0.05
0
0
12
24
36
48

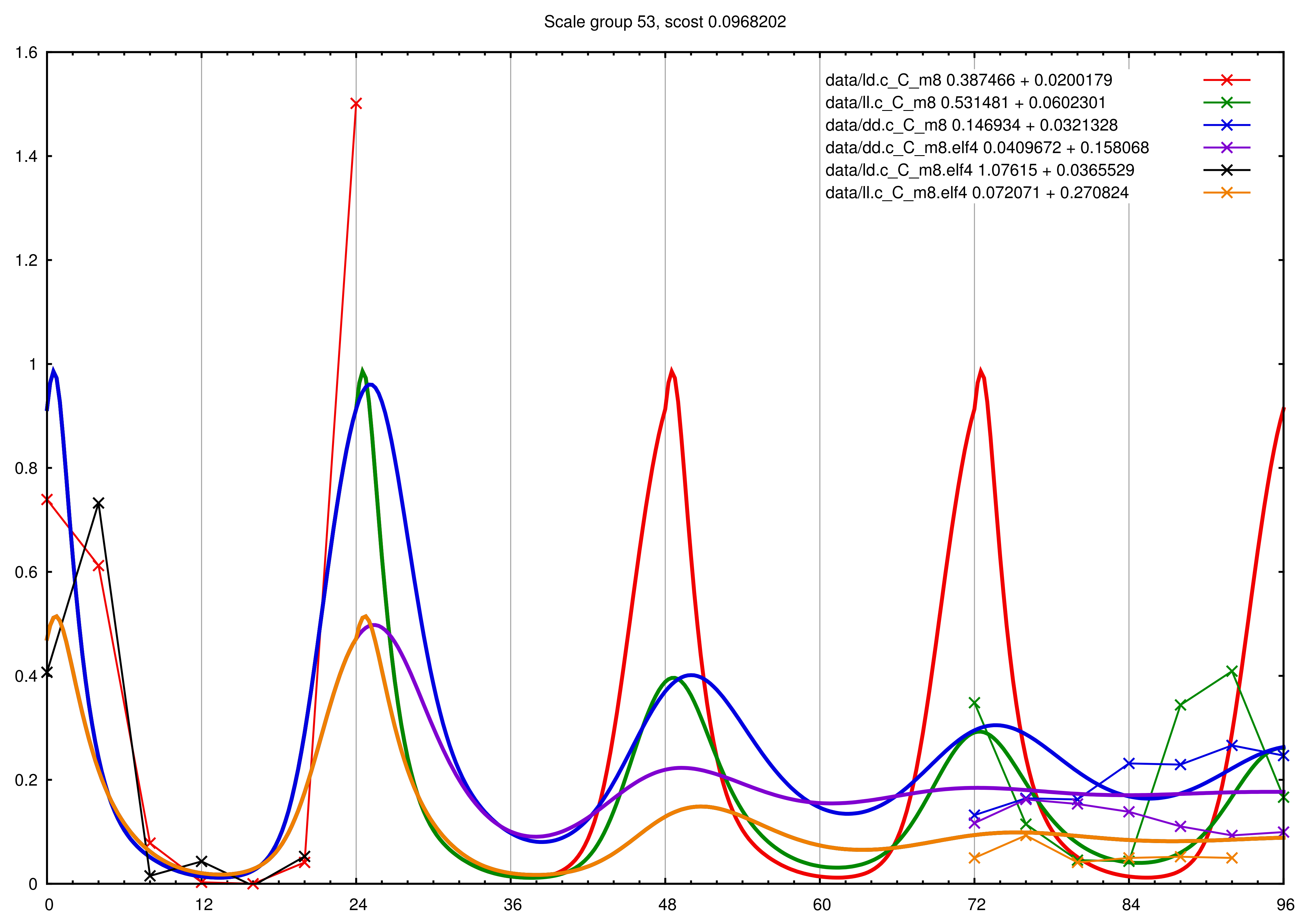
Scale group 53, scost 0.0968202
data/ld.c_C_m8 0.387466 + 0.0200179
data/ll.c_C_m8 0.531481 + 0.0602301
data/dd.c_C_m8 0.146934 + 0.0321328
data/dd.c_C_m8.elf4 0.0409672 + 0.158068
data/ld.c_C_m8.elf4 1.07615 + 0.0365529
data/ll.c_C_m8.elf4 0.072071 + 0.270824
1.6
1.4
1.2
1
0.8
0.6
0.4
0.2
0
0
12
24
36
48
60
72
84
96

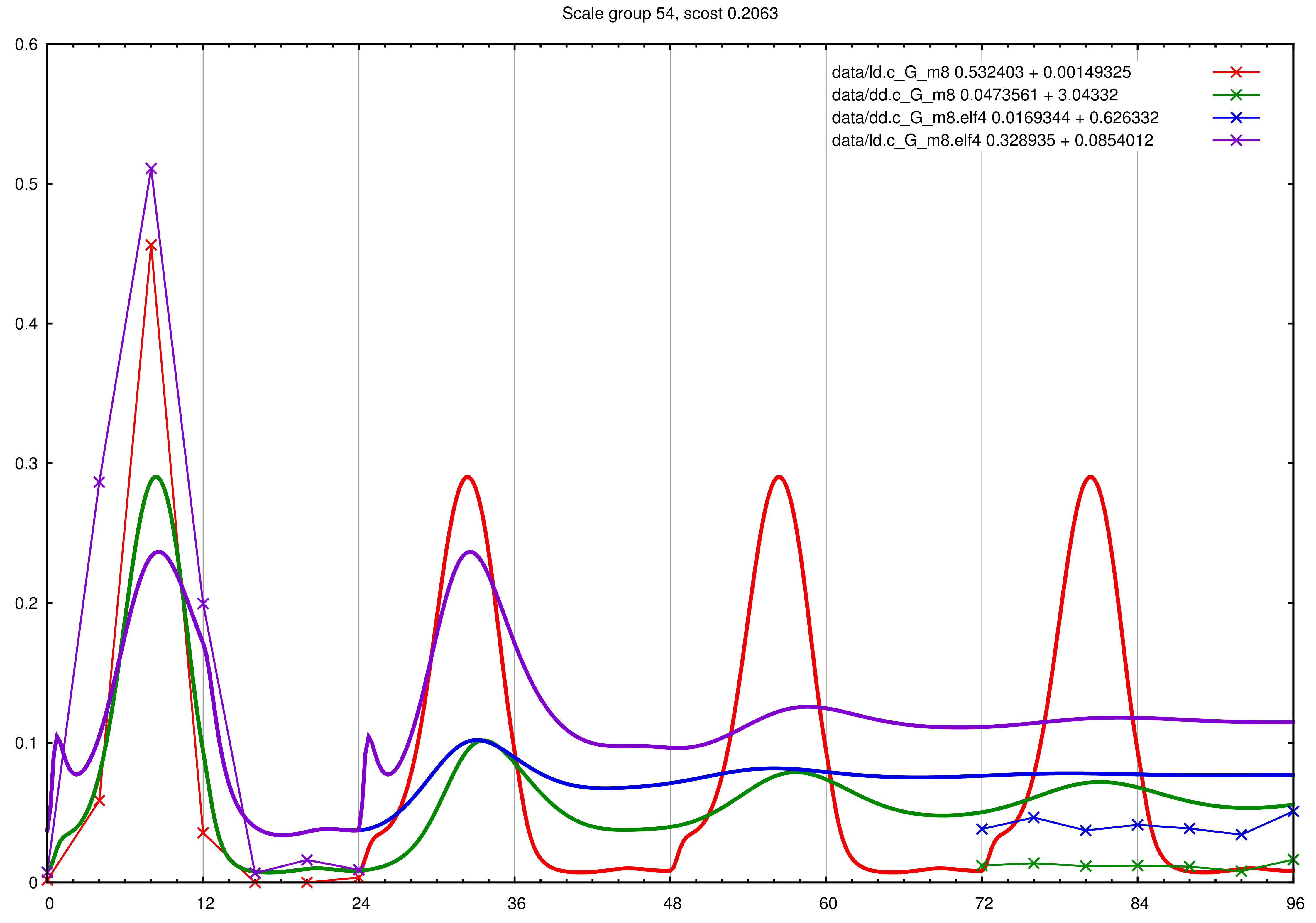

Scale group 54, scost 0.2063
data/ld.c_G_m8 0.532403 + 0.00149325
data/dd.c_G_m8 0.0473561 + 3.04332
data/dd.c_G_m8.elf4 0.0169344 + 0.626332
data/ld.c_G_m8.elf4 0.328935 + 0.0854012
0.6
0.5
0.4
0.3
0.2
0.1
0
0
12
24
36
48
60
72
84
96

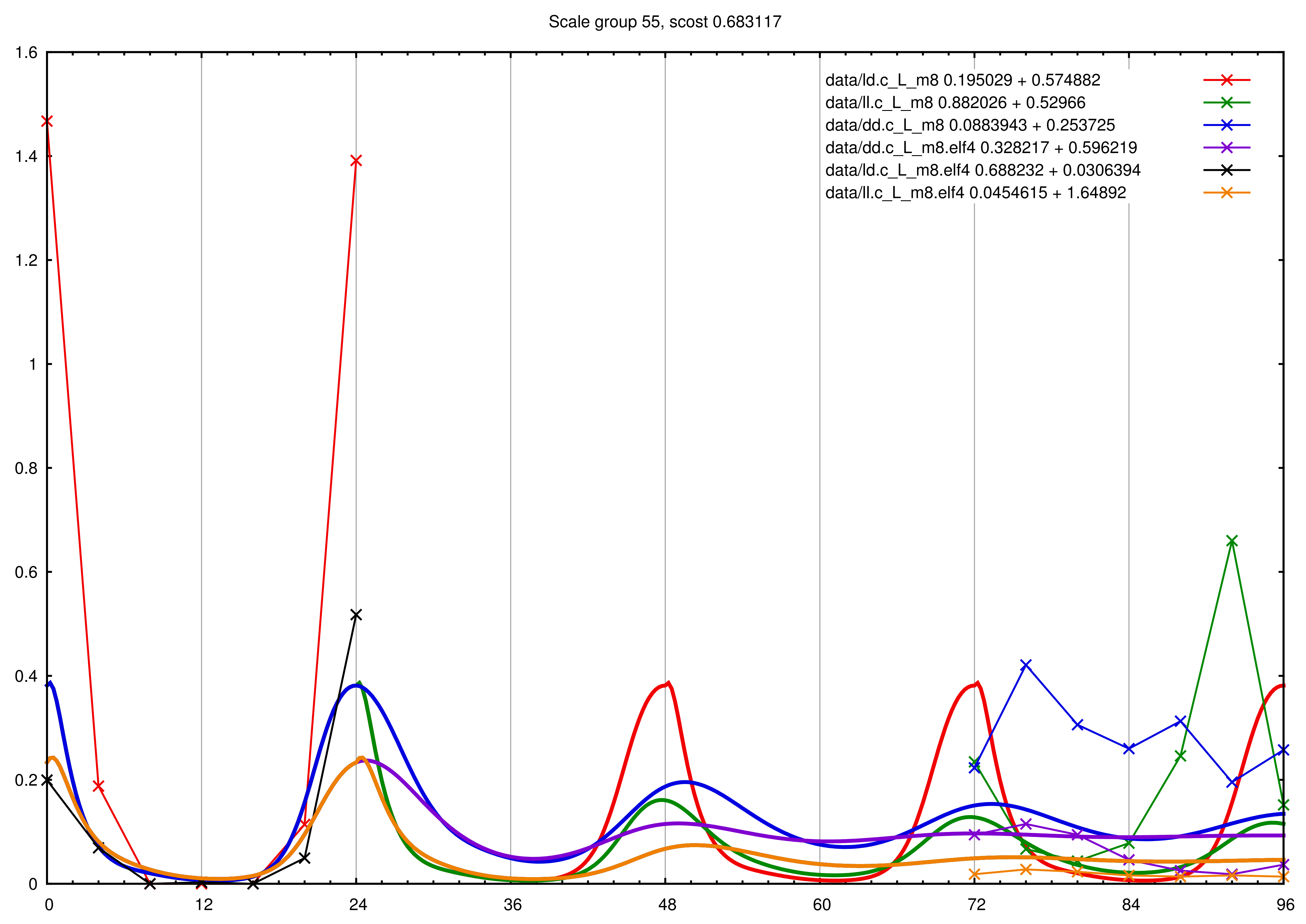
Scale group 55, scost 0.683117
data/ld.c_L_m8 0.195029 + 0.574882
data/ll.c_L_m8 0.882026 + 0.52966
data/dd.c_L_m8 0.0883943 + 0.253725
data/dd.c_L_m8.elf4 0.328217 + 0.596219
data/ld.c_L_m8.elf4 0.688232 + 0.0306394
data/ll.c_L_m8.elf4 0.0454615 + 1.64892
1.6
1.4
1.2
1
0.8
0.6
0.4
0.2
0
0
12
24
36
48
60
72
84
96

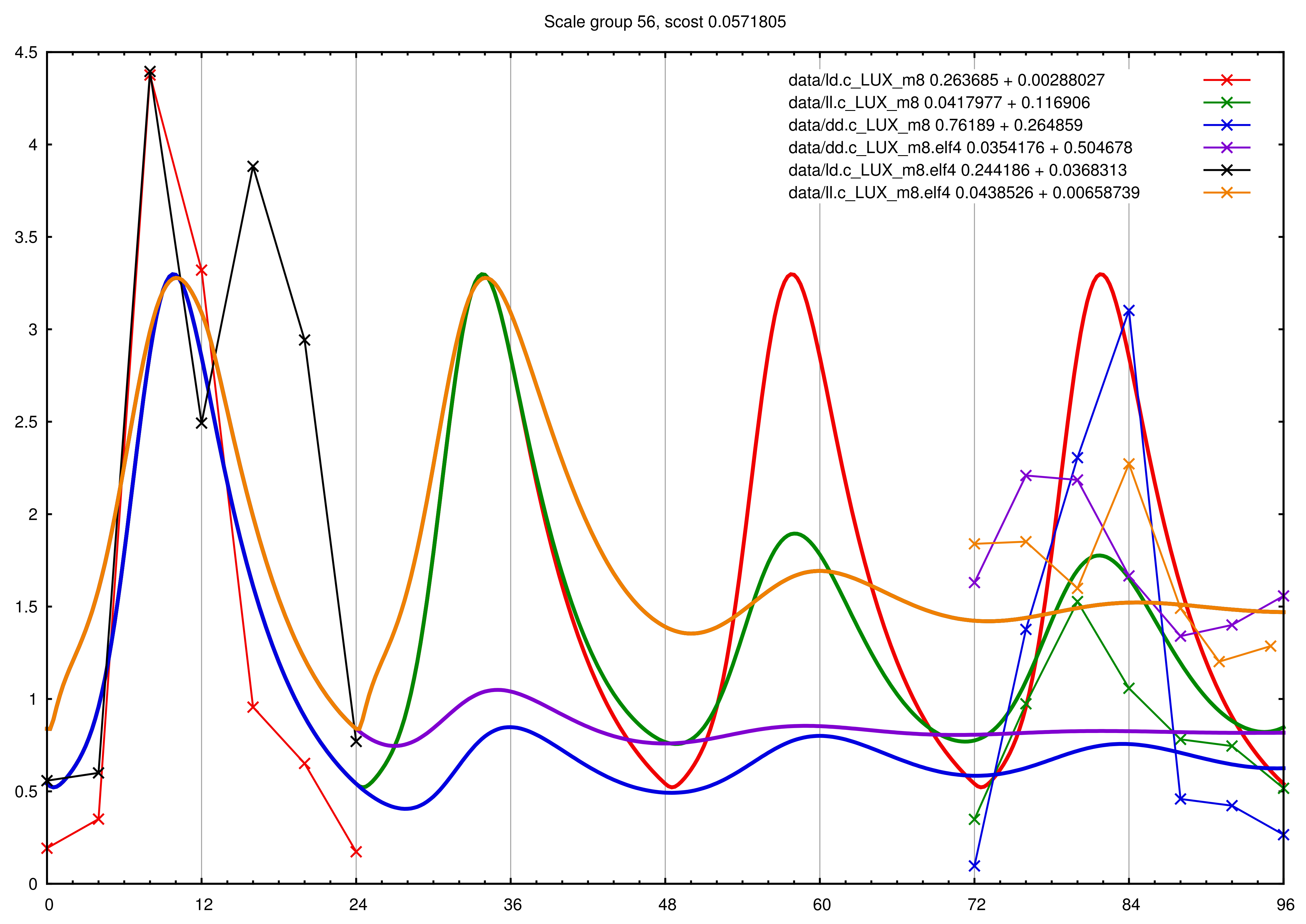

Scale group 56, scost 0.0571805
data/ld.c_LUX_m8 0.263685 + 0.00288027
data/ll.c_LUX_m8 0.0417977 + 0.116906
data/dd.c_LUX_m8 0.76189 + 0.264859
data/dd.c_LUX_m8.elf4 0.0354176 + 0.504678
data/ld.c_LUX_m8.elf4 0.244186 + 0.0368313
data/ll.c_LUX_m8.elf4 0.0438526 + 0.00658739
4.5
4
3.5
3
2.5
2
1.5
1
0.5
0
0
12
24
36
48
60
72
84
96

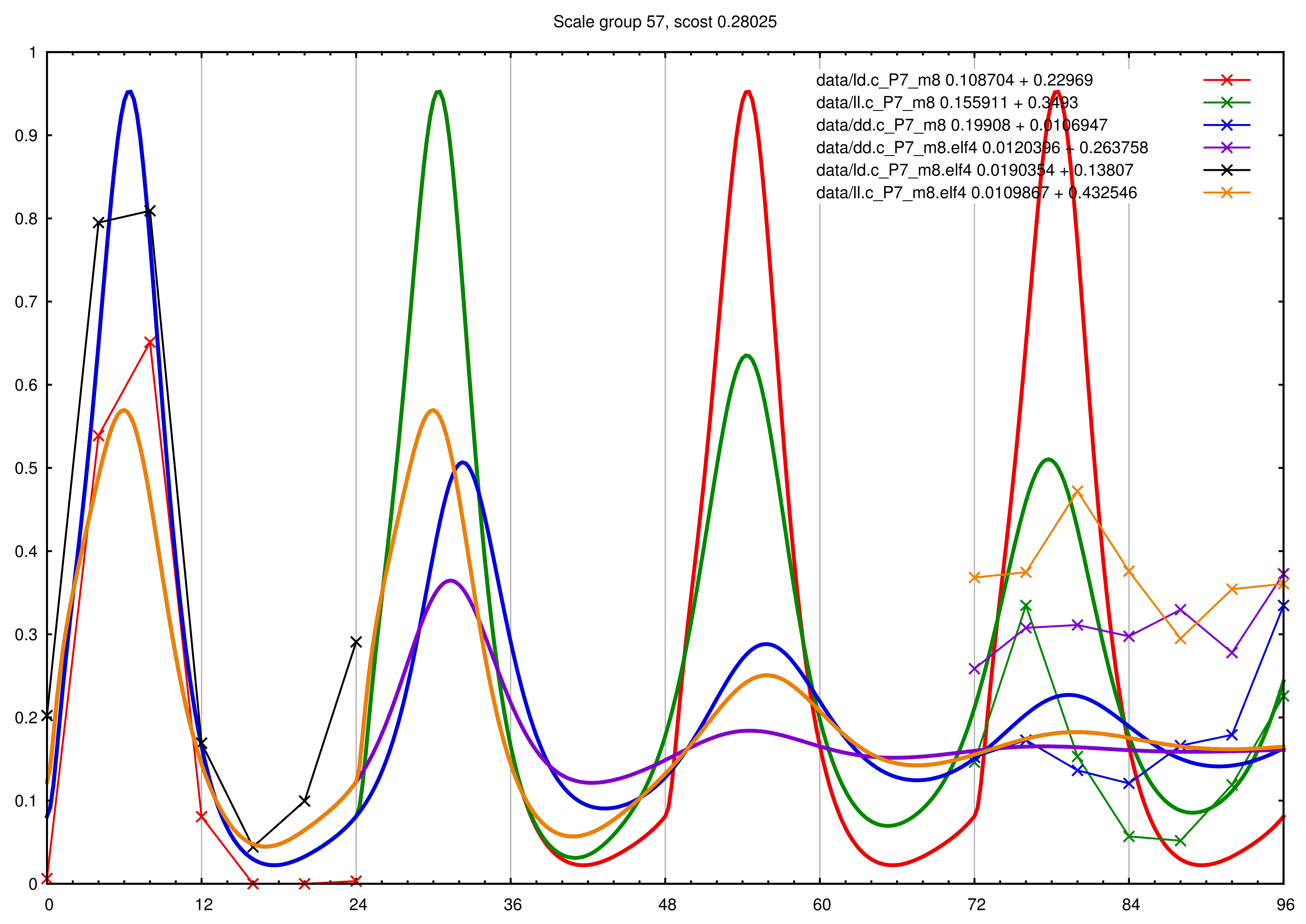
Scale group 57, scost 0.28025
data/ld.c_P7_m8 0.108704 + 0.22969
data/ll.c_P7_m8 0.155911 + 0.3493
data/dd.c_P7_m8 0.19908 + 0.0106947
data/dd.c_P7_m8.elf4 0.0120396 + 0.263758
data/ld.c_P7_m8.elf4 0.0190354 + 0.13807
data/ll.c_P7_m8.elf4 0.0109867 + 0.432546
0
0.1
0.2
0.3
0.4
0.5
0.6
0.7
0.8
0.9
1
0
12
24
36
48
60
72
84
96

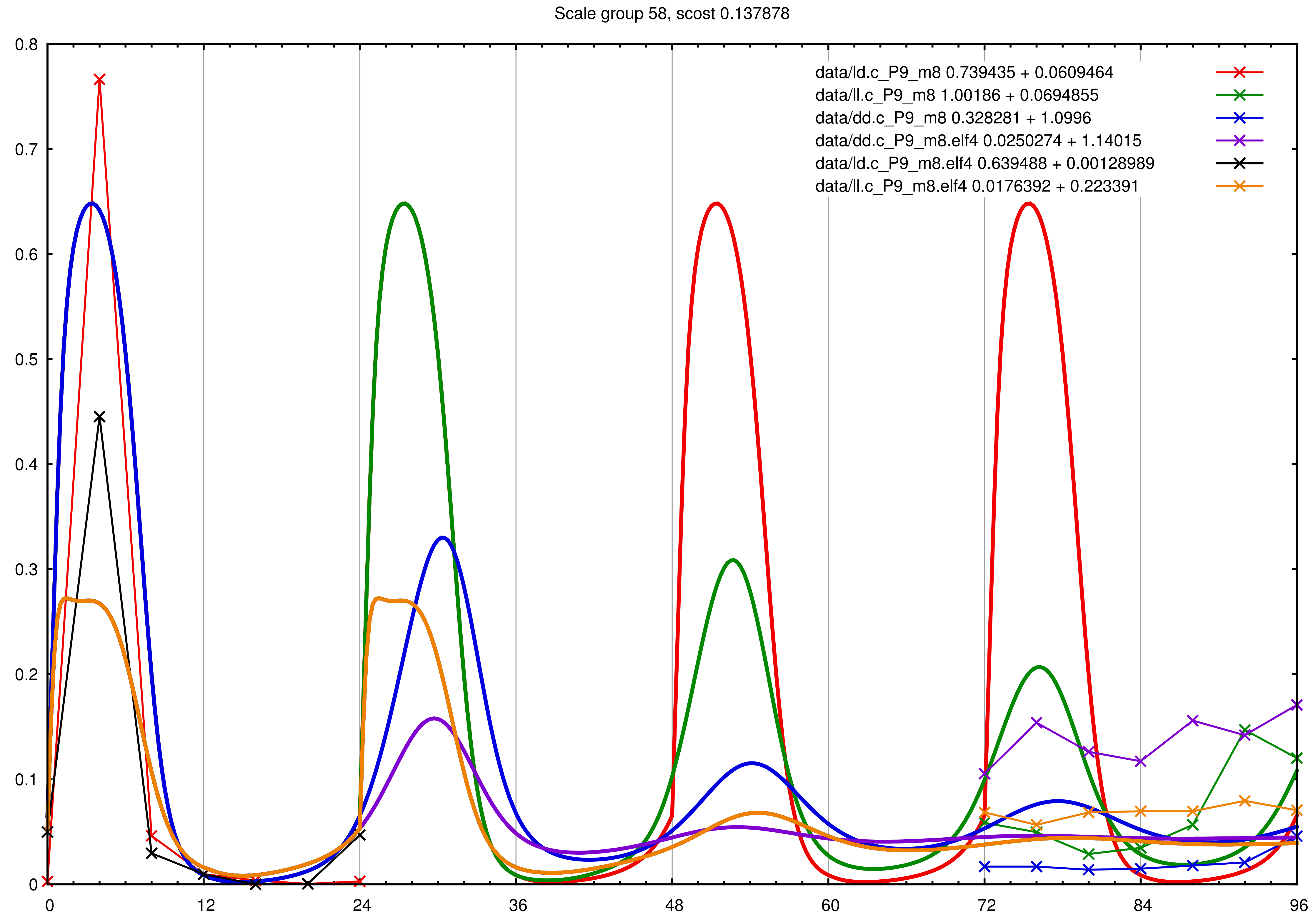

Scale group 58, scost 0.137878
data/ld.c_P9_m8 0.739435 + 0.0609464
data/ll.c_P9_m8 1.00186 + 0.0694855
data/dd.c_P9_m8 0.328281 + 1.0996
data/dd.c_P9_m8.elf4 0.0250274 + 1.14015
data/ld.c_P9_m8.elf4 0.639488 + 0.00128989
data/ll.c_P9_m8.elf4 0.0176392 + 0.223391
0.8
0.7
0.6
0.5
0.4
0.3
0.2
0.1
0
0
12
24
36
48
60
72
84
96

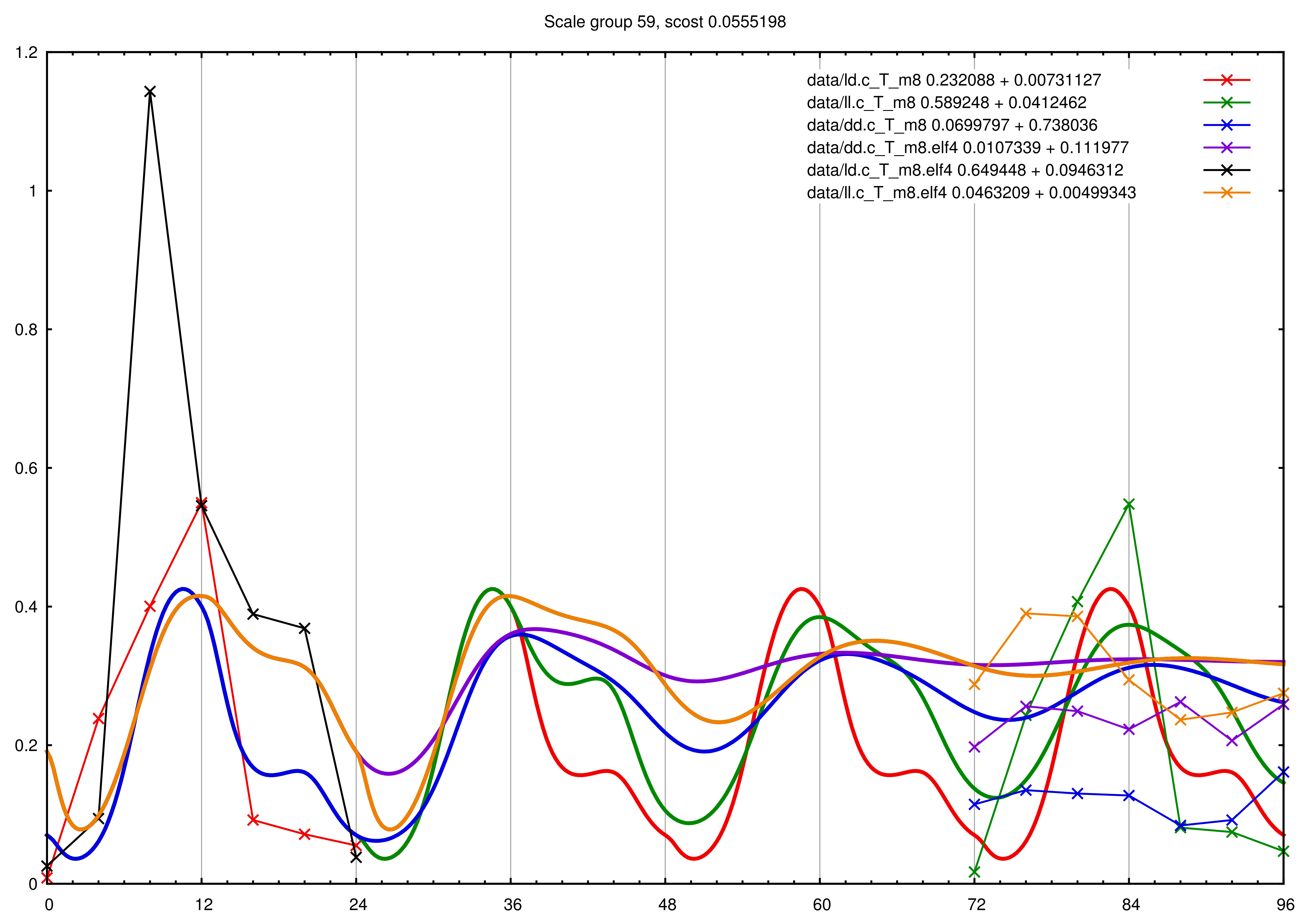

Scale group 59, scost 0.0555198
data/ld.c_T_m8 0.232088 + 0.00731127
data/ll.c_T_m8 0.589248 + 0.0412462
data/dd.c_T_m8 0.0699797 + 0.738036
data/dd.c_T_m8.elf4 0.0107339 + 0.111977
data/ld.c_T_m8.elf4 0.649448 + 0.0946312
data/ll.c_T_m8.elf4 0.0463209 + 0.00499343
1.2
1
0.8
0.6
0.4
0.2
0
0
12
24
36
48
60
72
84
96

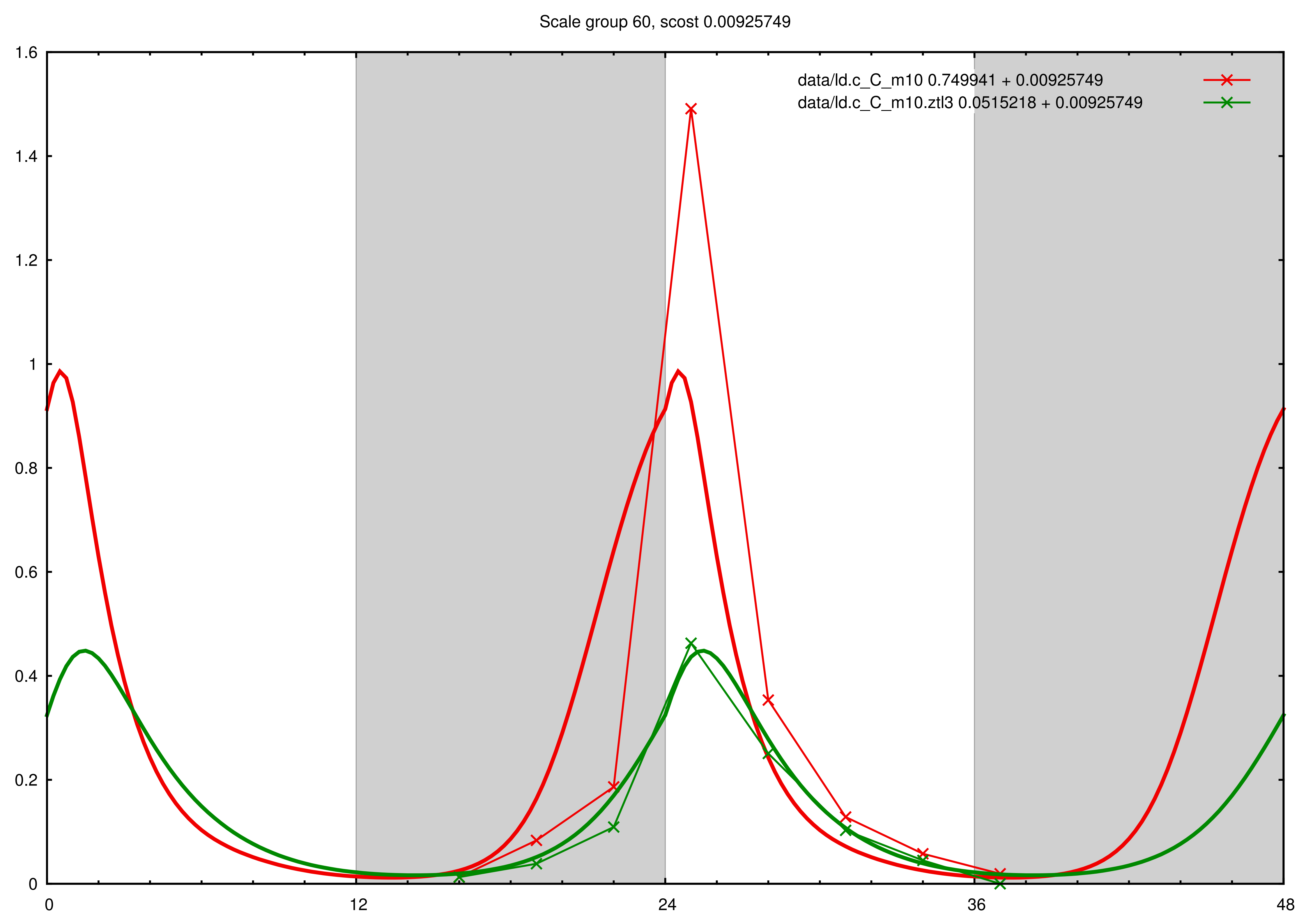

Scale group 60, scost 0.00925749
data/ld.c_C_m10 0.749941 + 0.00925749
data/ld.c_C_m10.ztl3 0.0515218 + 0.00925749
1.6
1.4
1.2
1
0.8
0.6
0.4
0.2
0
0
12
24
36
48

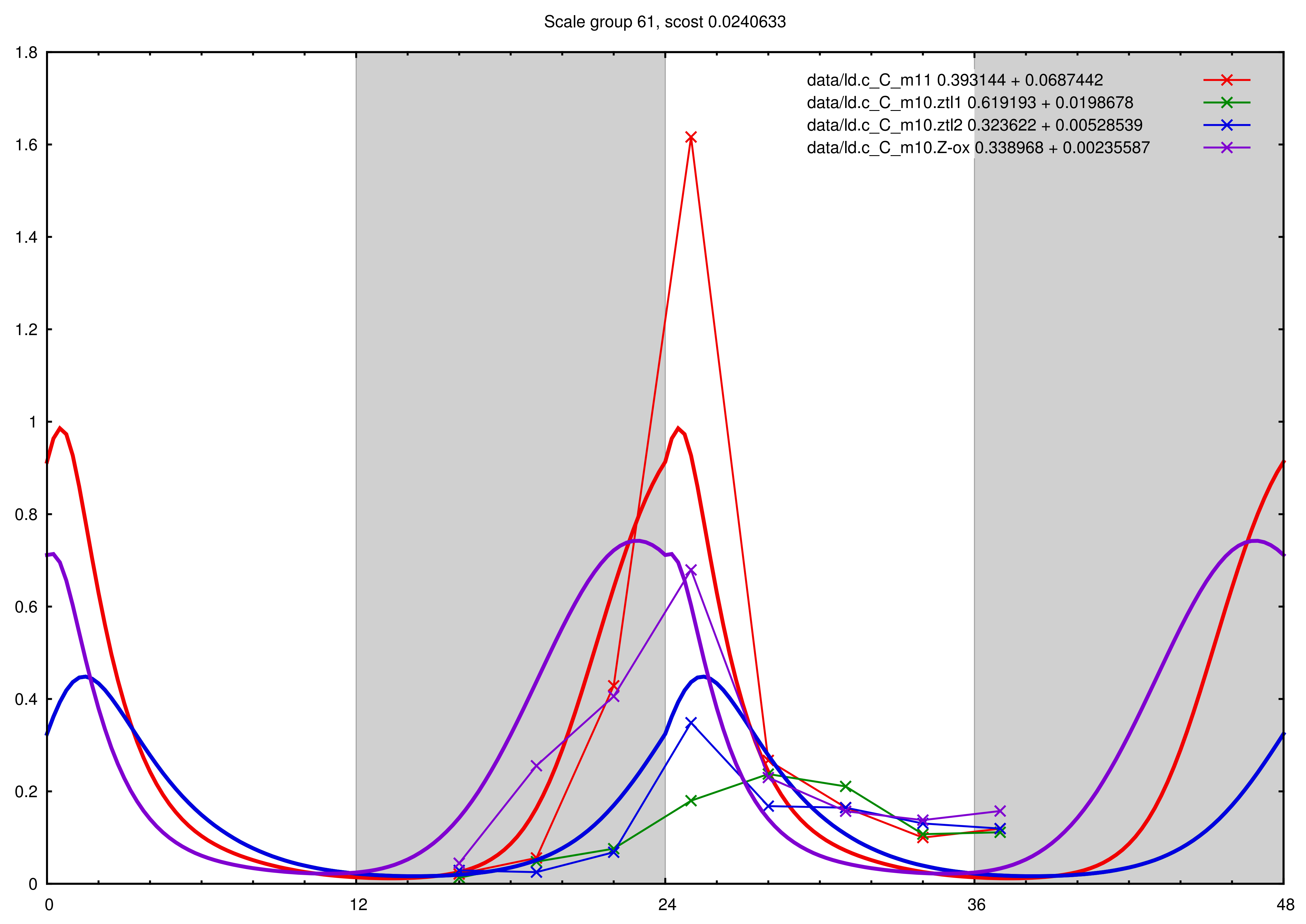
Scale group 61, scost 0.0240633
data/ld.c_C_m11 0.393144 + 0.0687442
data/ld.c_C_m10.ztl1 0.619193 + 0.0198678
data/ld.c_C_m10.ztl2 0.323622 + 0.00528539
data/ld.c_C_m10.Z-ox 0.338968 + 0.00235587
1.8
1.6
1.4
1.2
1
0.8
0.6
0.4
0.2
0
0
12
24
36
48

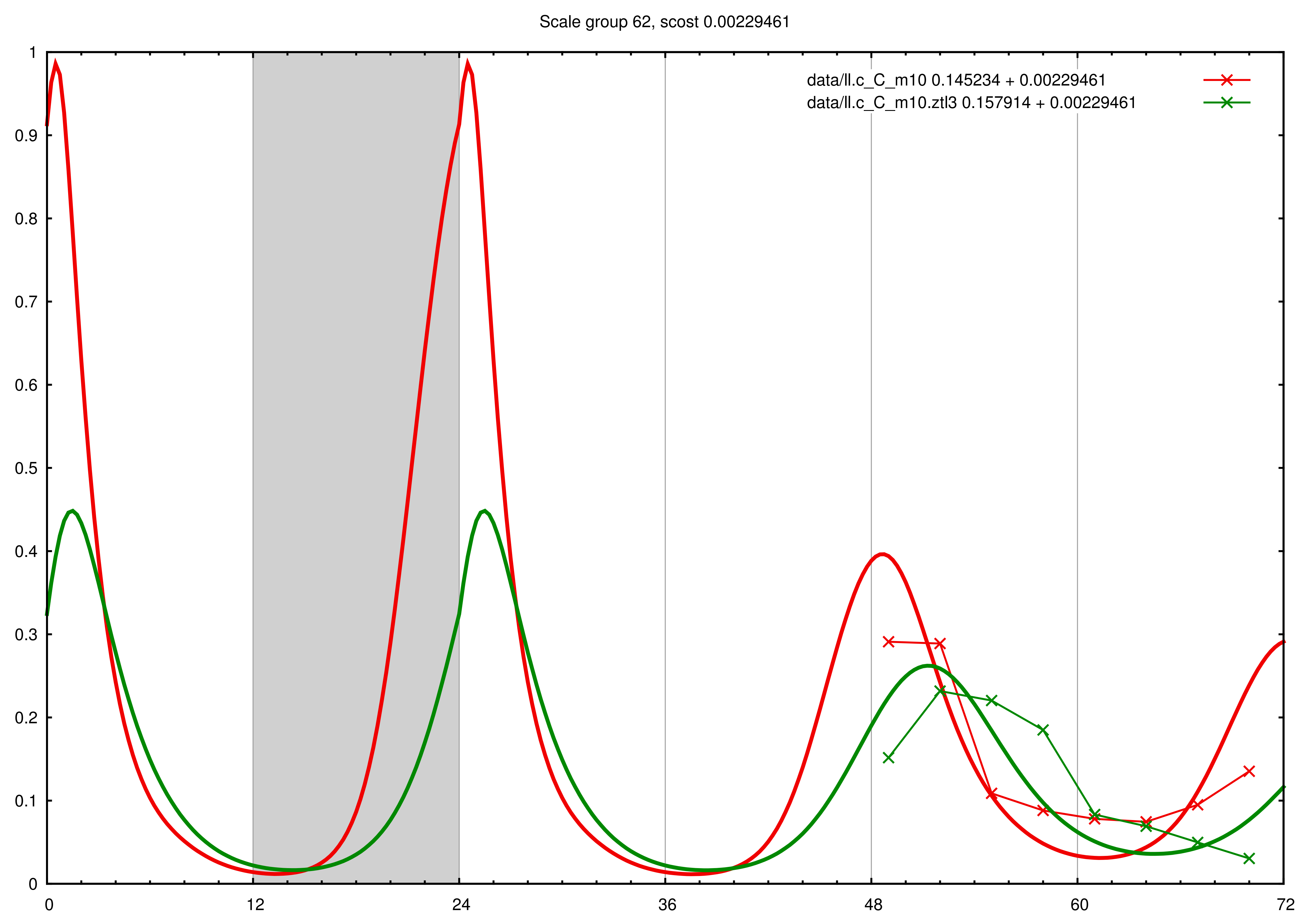

Scale group 62, scost 0.00229461
data/ll.c_C_m10 0.145234 + 0.00229461
data/ll.c_C_m10.ztl3 0.157914 + 0.00229461
0
0.1
0.2
0.3
0.4
0.5
0.6
0.7
0.8
0.9
1
0
12
24
36
48
60
72

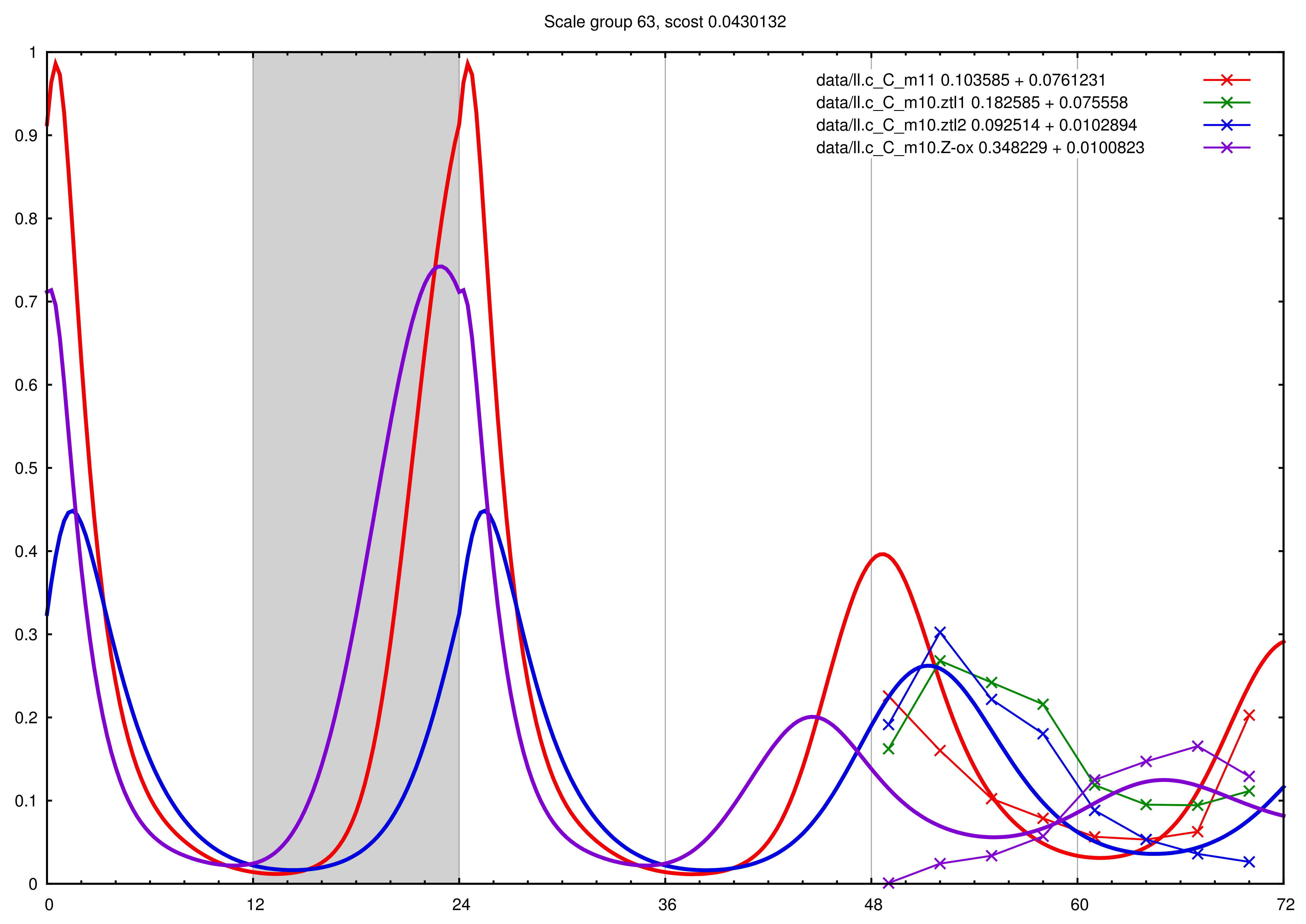
Scale group 63, scost 0.0430132
data/ll.c_C_m11 0.103585 + 0.0761231
data/ll.c_C_m10.ztl1 0.182585 + 0.075558
data/ll.c_C_m10.ztl2 0.092514 + 0.0102894
data/ll.c_C_m10.Z-ox 0.348229 + 0.0100823
0
0.1
0.2
0.3
0.4
0.5
0.6
0.7
0.8
0.9
1
0
12
24
36
48
60
72

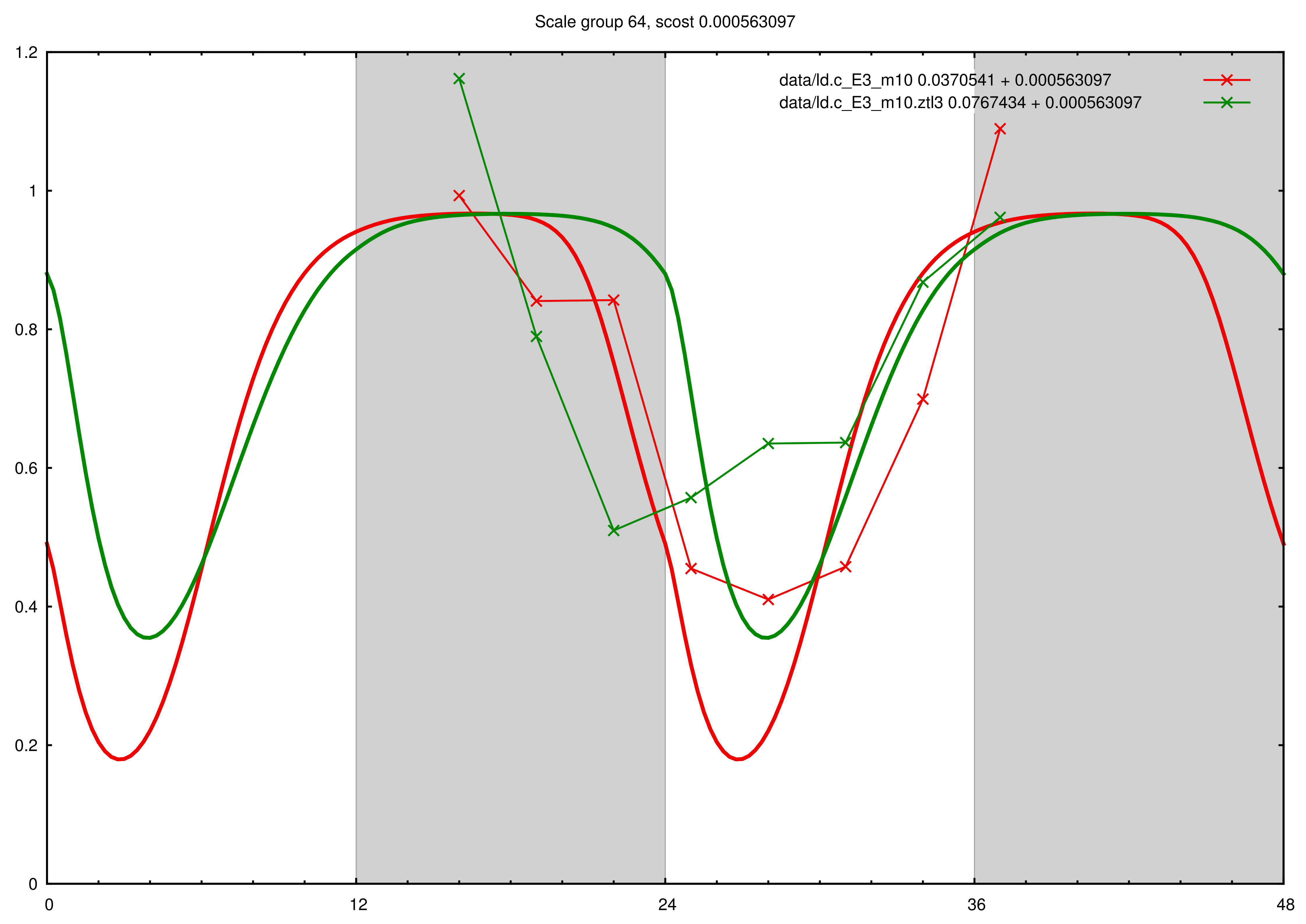

Scale group 64, scost 0.000563097
data/ld.c_E3_m10 0.0370541 + 0.000563097
data/ld.c_E3_m10.ztl3 0.0767434 + 0.000563097
1.2
1
0.8
0.6
0.4
0.2
0
0
12
24
36
48

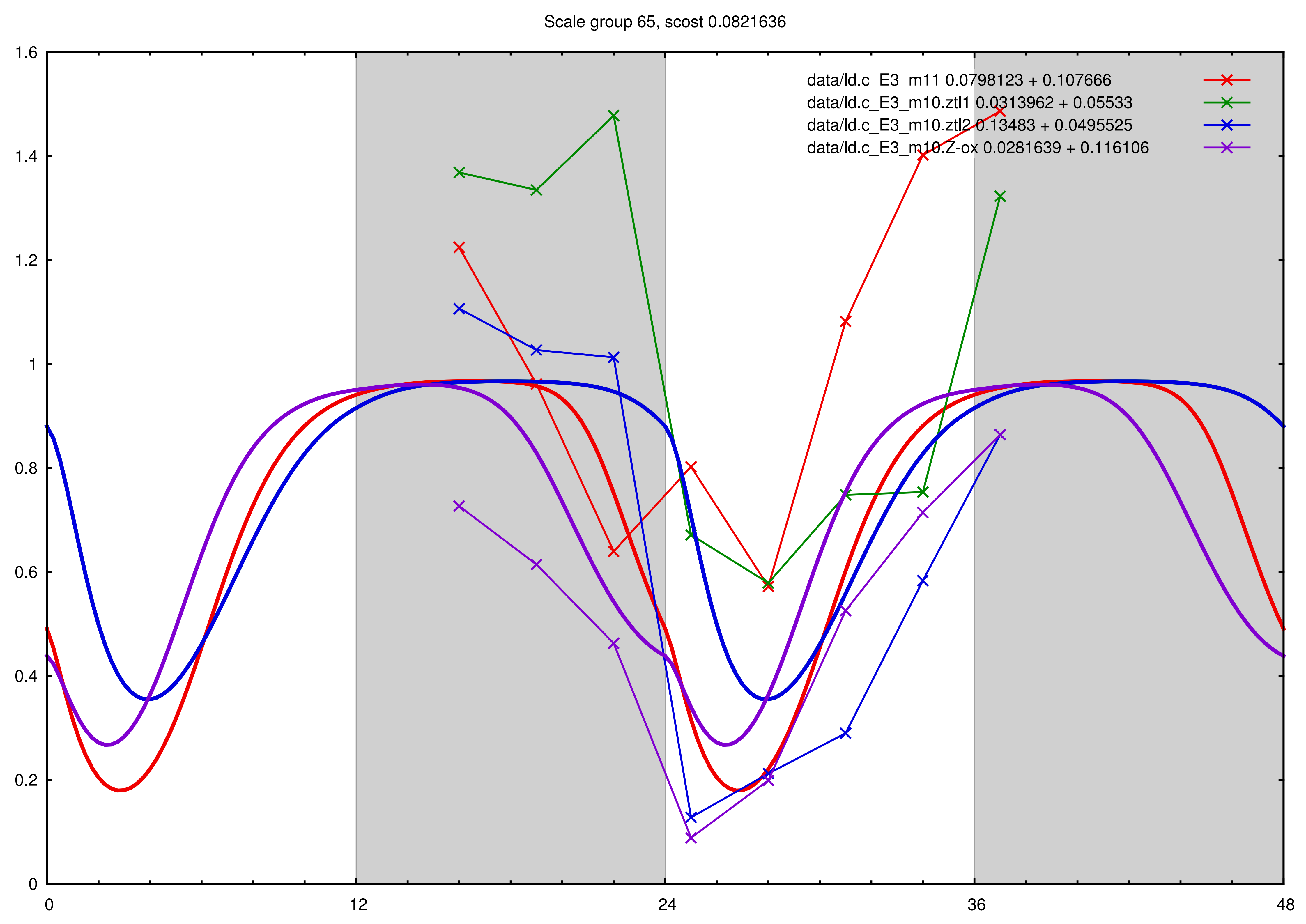
Scale group 65, scost 0.0821636
data/ld.c_E3_m11 0.0798123 + 0.107666
data/ld.c_E3_m10.ztl1 0.0313962 + 0.05533
data/ld.c_E3_m10.ztl2 0.13483 + 0.0495525
data/ld.c_E3_m10.Z-ox 0.0281639 + 0.116106
1.6
1.4
1.2
1
0.8
0.6
0.4
0.2
0
0
12
24
36
48

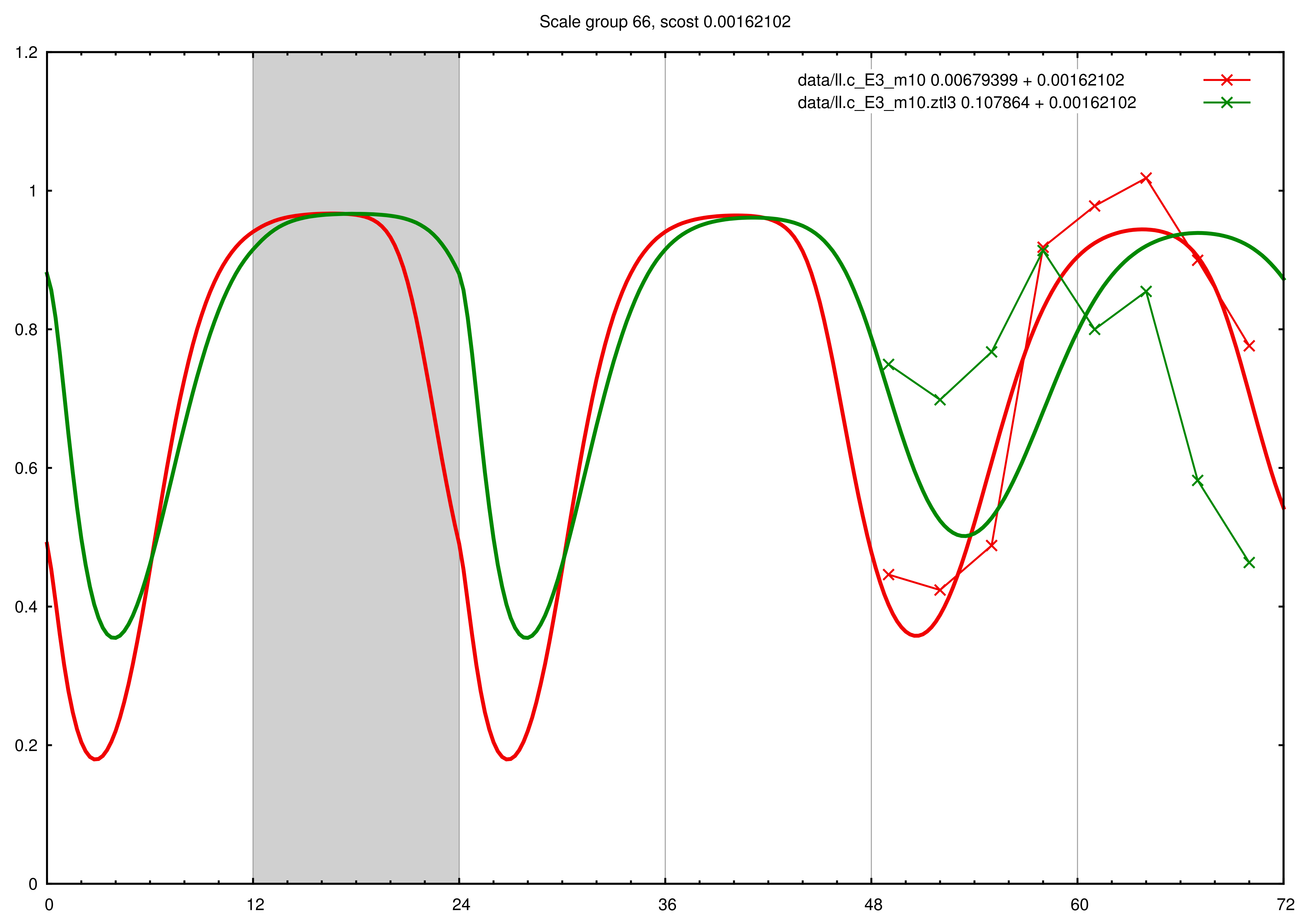

Scale group 66, scost 0.00162102
data/ll.c_E3_m10 0.00679399 + 0.00162102
data/ll.c_E3_m10.ztl3 0.107864 + 0.00162102
1.2
1
0.8
0.6
0.4
0.2
0
0
12
24
36
48
60
72

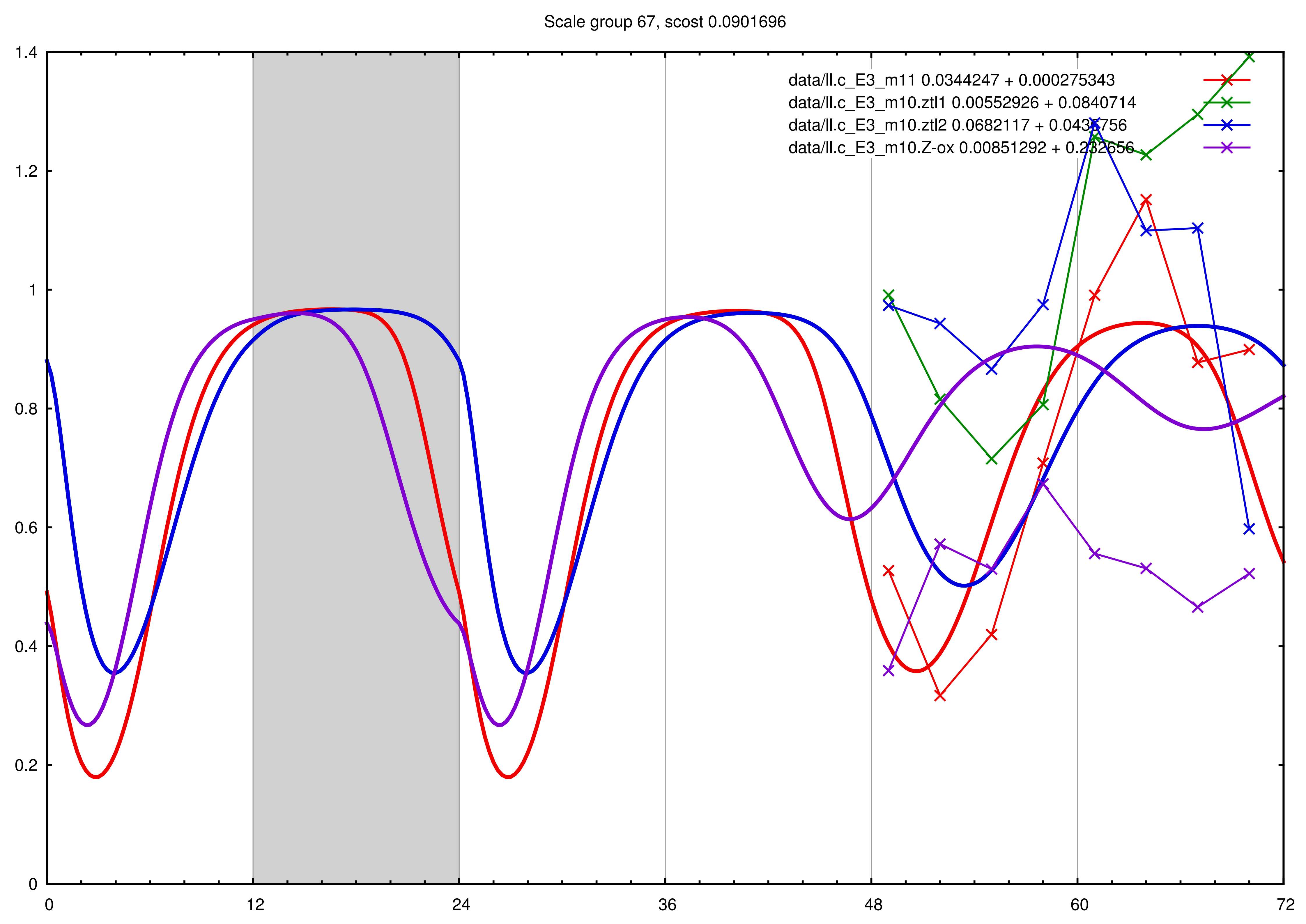

Scale group 67, scost 0.0901696
data/ll.c_E3_m11 0.0344247 + 0.000275343
data/ll.c_E3_m10.ztl1 0.00552926 + 0.0840714
data/ll.c_E3_m10.ztl2 0.0682117 + 0.0435756
data/ll.c_E3_m10.Z-ox 0.00851292 + 0.232656
1.4
1.2
1
0.8
0.6
0.4
0.2
0
0
12
24
36
48
60
72

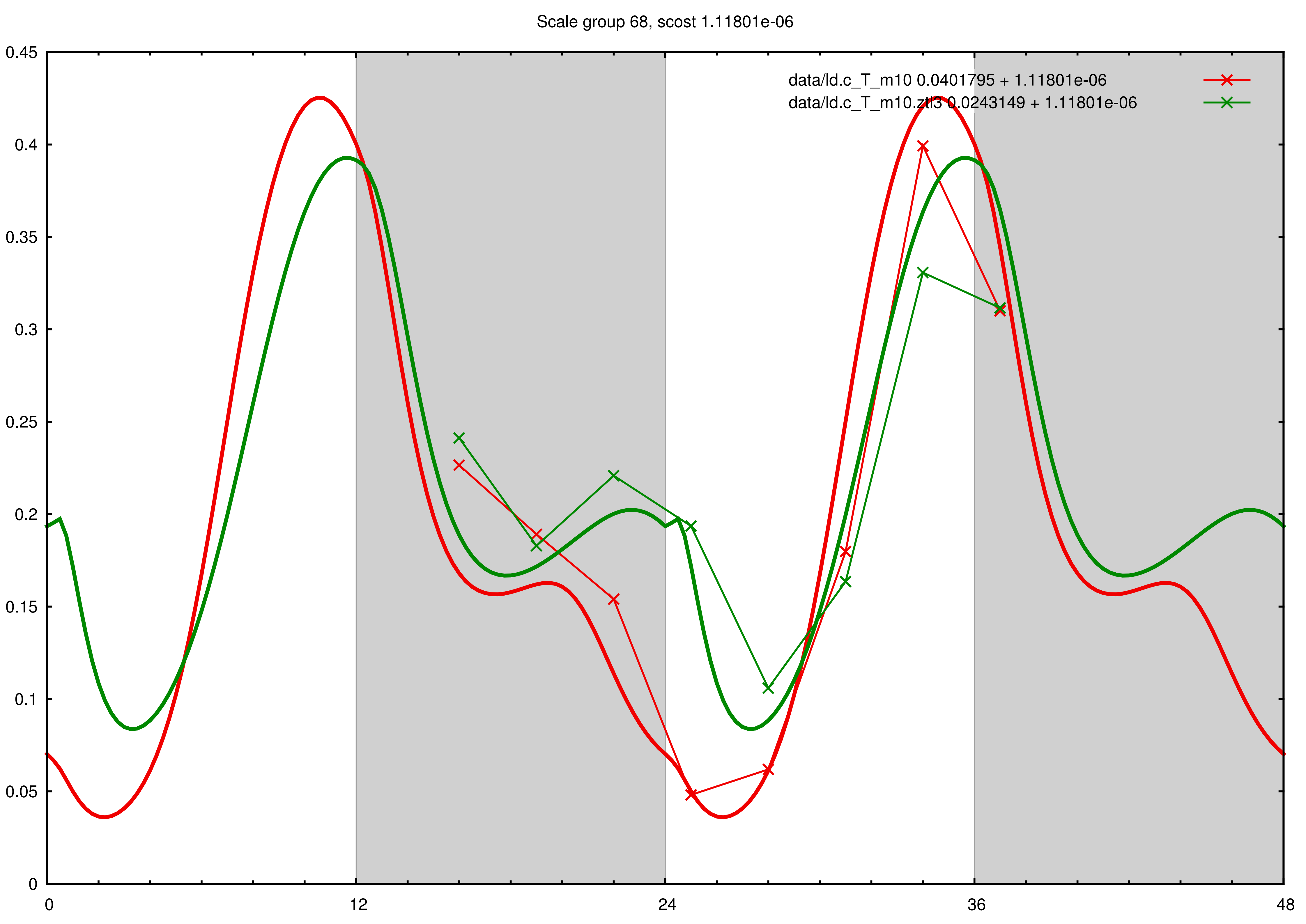

Scale group 68, scost 1.11801e-06
data/ld.c_T_m10 0.0401795 + 1.11801e-06
data/ld.c_T_m10.ztl3 0.0243149 + 1.11801e-06
0.45
0.4
0.35
0.3
0.25
0.2
0.15
0.1
0.05
0
0
12
24
36
48

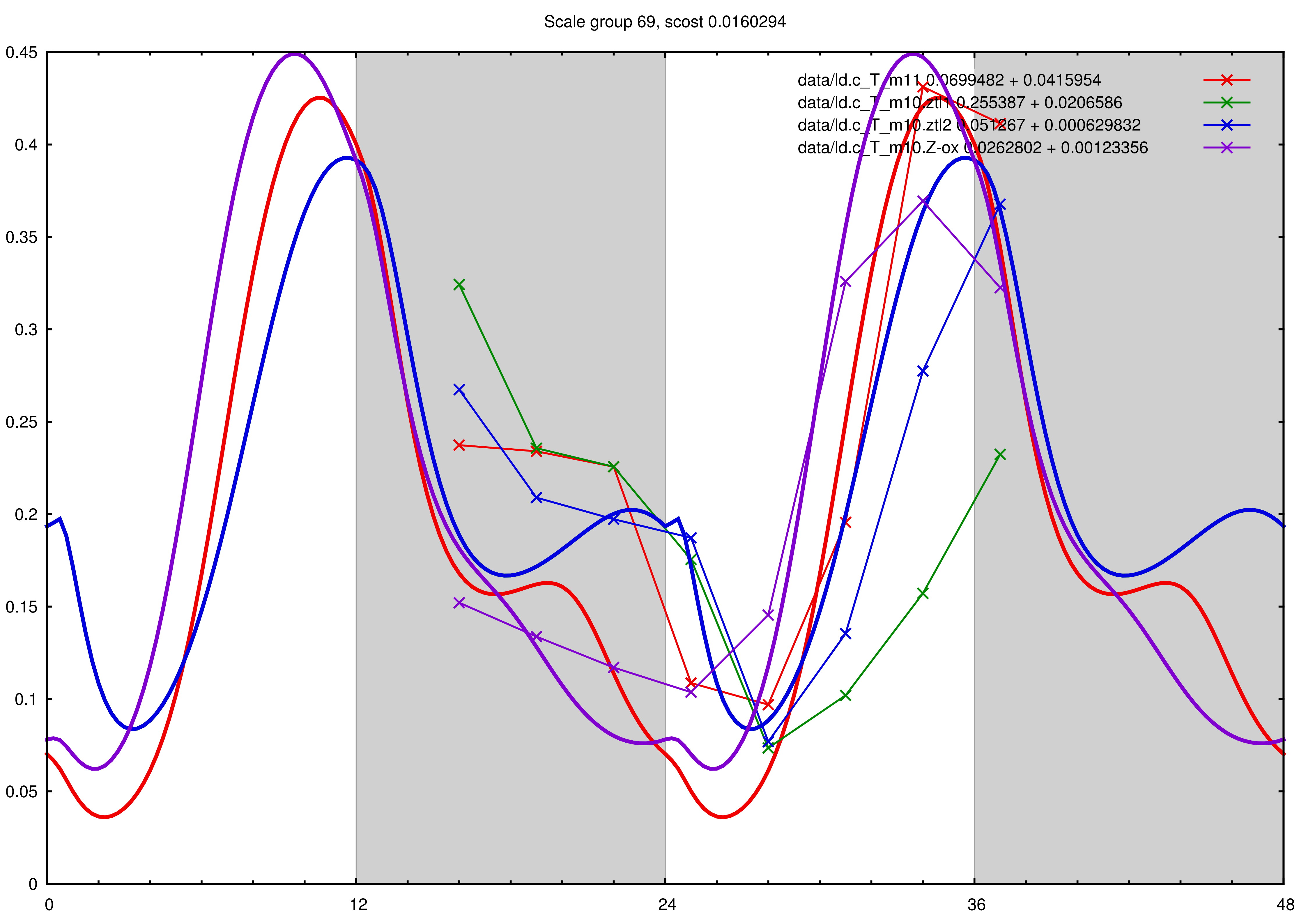
Scale group 69, scost 0.0160294
data/ld.c_T_m11 0.0699482 + 0.0415954
data/ld.c_T_m10.ztl1 0.255387 + 0.0206586
data/ld.c_T_m10.ztl2 0.051267 + 0.000629832
data/ld.c_T_m10.Z-ox 0.0262802 + 0.00123356
0.45
0.4
0.35
0.3
0.25
0.2
0.15
0.1
0.05
0
0
12
24
36
48

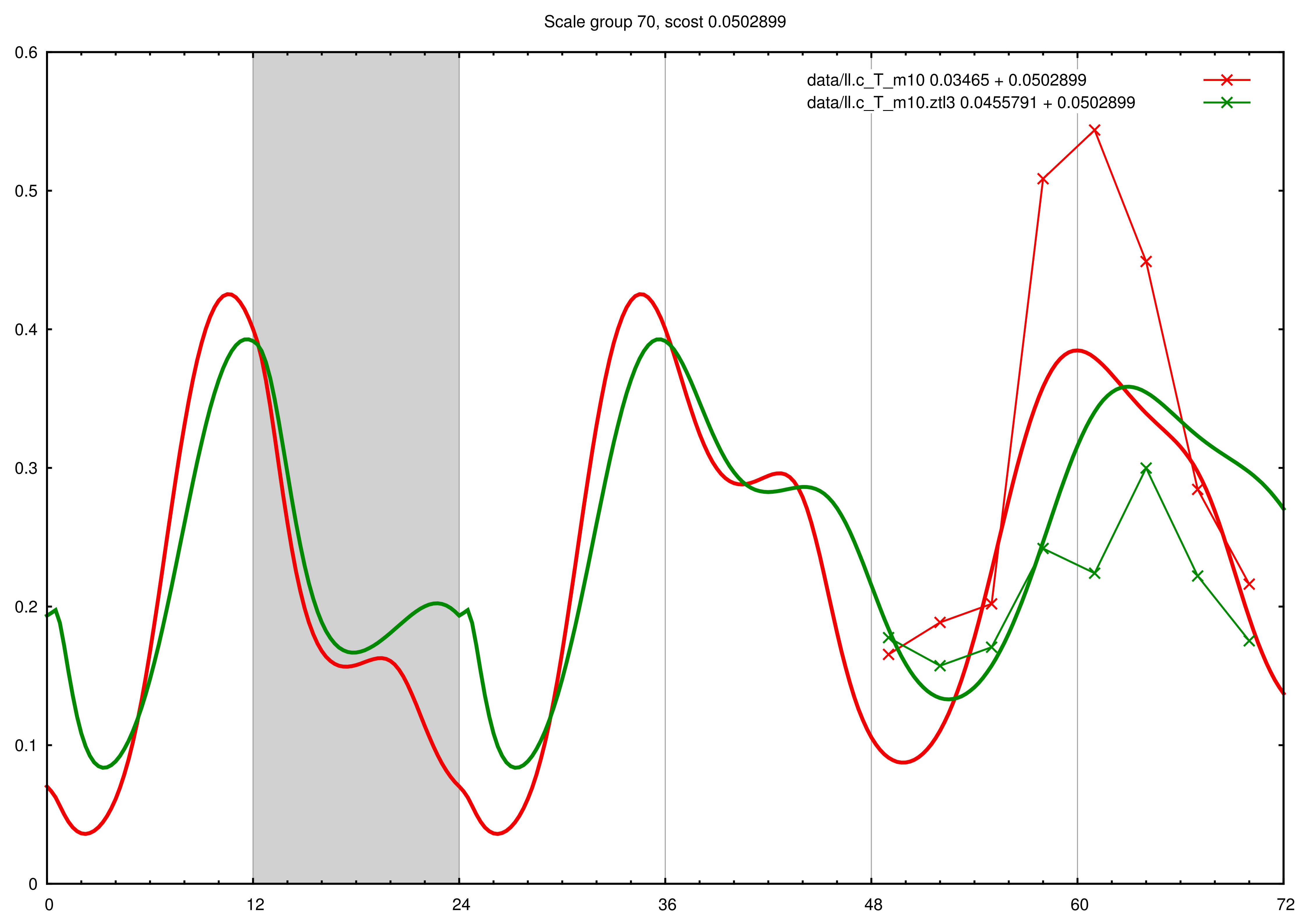
Scale group 70, scost 0.0502899
data/ll.c_T_m10 0.03465 + 0.0502899
data/ll.c_T_m10.ztl3 0.0455791 + 0.0502899
0.6
0.5
0.4
0.3
0.2
0.1
0
0
12
24
36
48
60
72

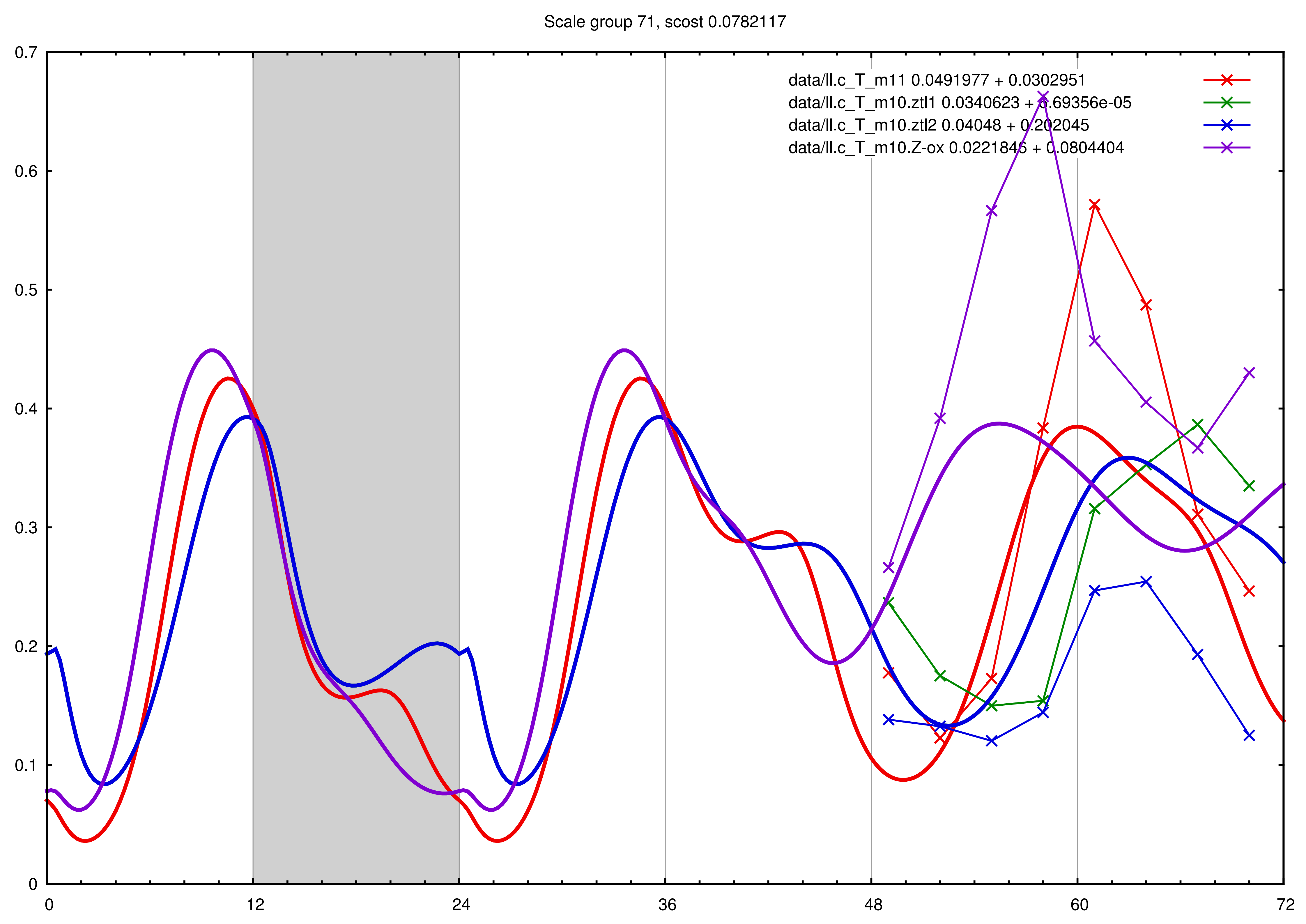

Scale group 71, scost 0.0782117
data/ll.c_T_m11 0.0491977 + 0.0302951
data/ll.c_T_m10.ztl1 0.0340623 + 6.69356e-05
data/ll.c_T_m10.ztl2 0.04048 + 0.202045
data/ll.c_T_m10.Z-ox 0.0221846 + 0.0804404
0
0.1
0.2
0.3
0.4
0.5
0.6
0.7
0
12
24
36
48
60
72

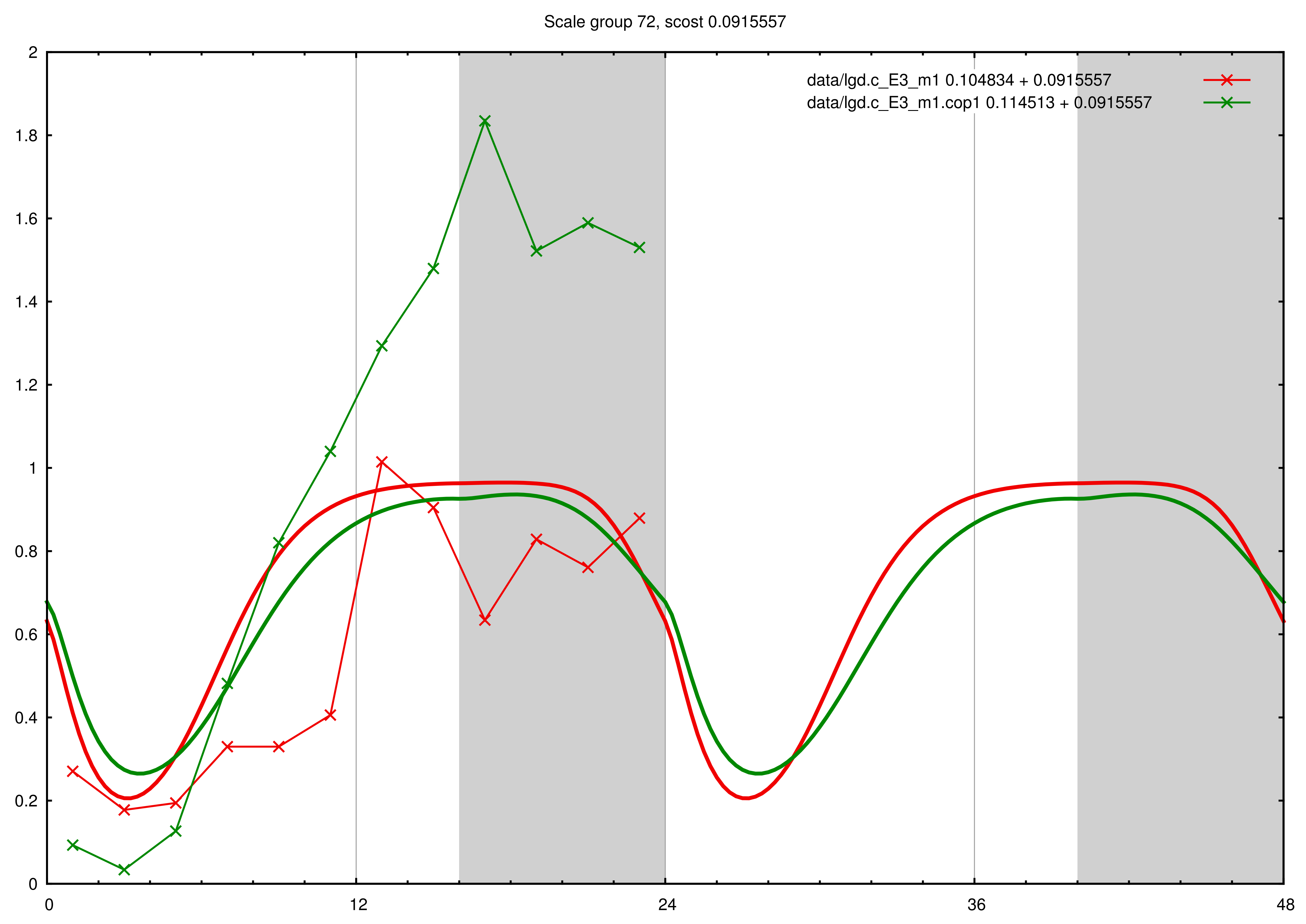

Scale group 72, scost 0.0915557
data/lgd.c_E3_m1 0.104834 + 0.0915557
data/lgd.c_E3_m1.cop1 0.114513 + 0.0915557
2
1.8
1.6
1.4
1.2
1
0.8
0.6
0.4
0.2
0
0
12
24
36
48

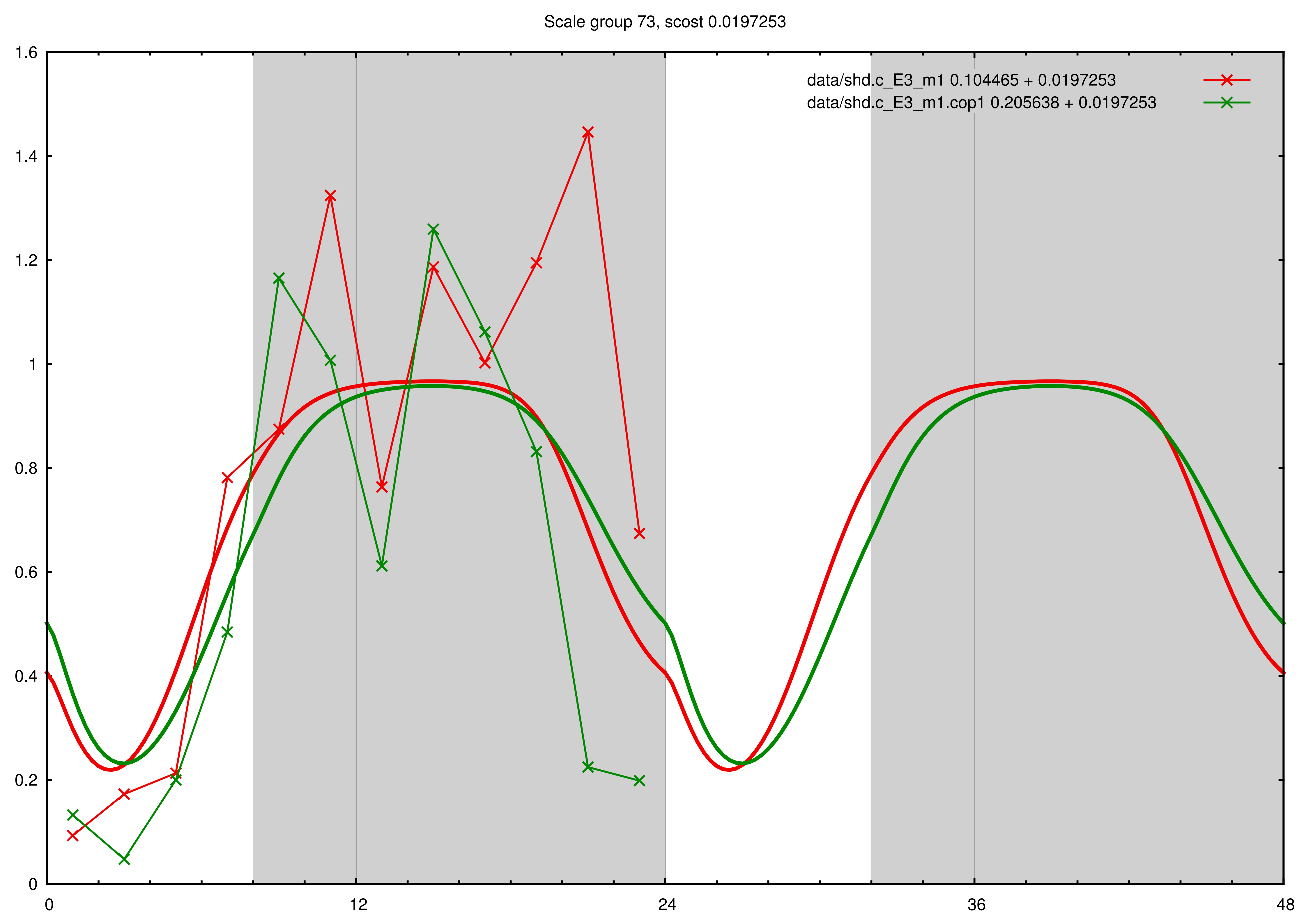

Scale group 73, scost 0.0197253
data/shd.c_E3_m1 0.104465 + 0.0197253
data/shd.c_E3_m1.cop1 0.205638 + 0.0197253
1.6
1.4
1.2
1
0.8
0.6
0.4
0.2
0
0
12
24
36
48

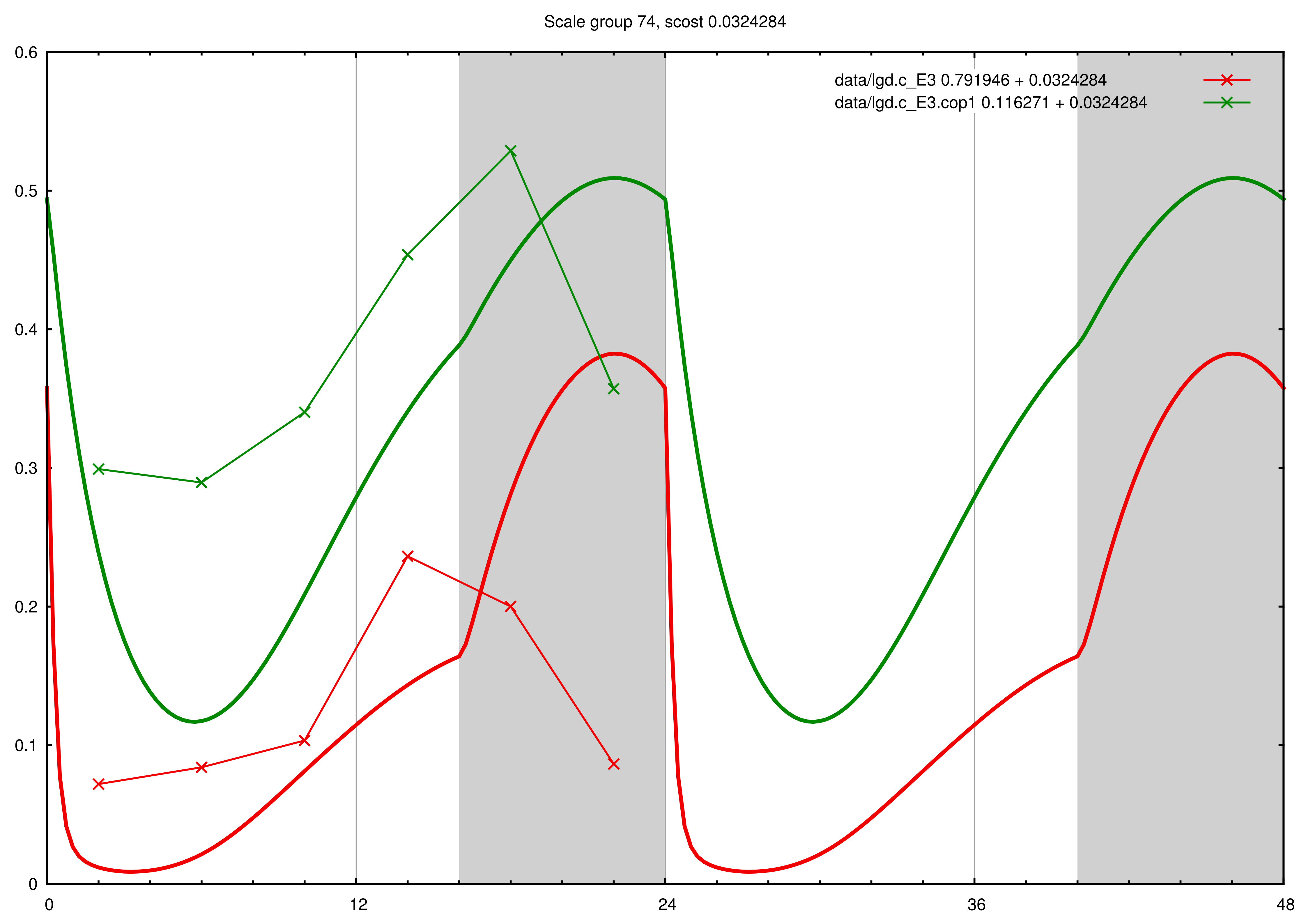

Scale group 74, scost 0.0324284
data/lgd.c_E3 0.791946 + 0.0324284
data/lgd.c_E3.cop1 0.116271 + 0.0324284
0.6
0.5
0.4
0.3
0.2
0.1
0
0
12
24
36
48

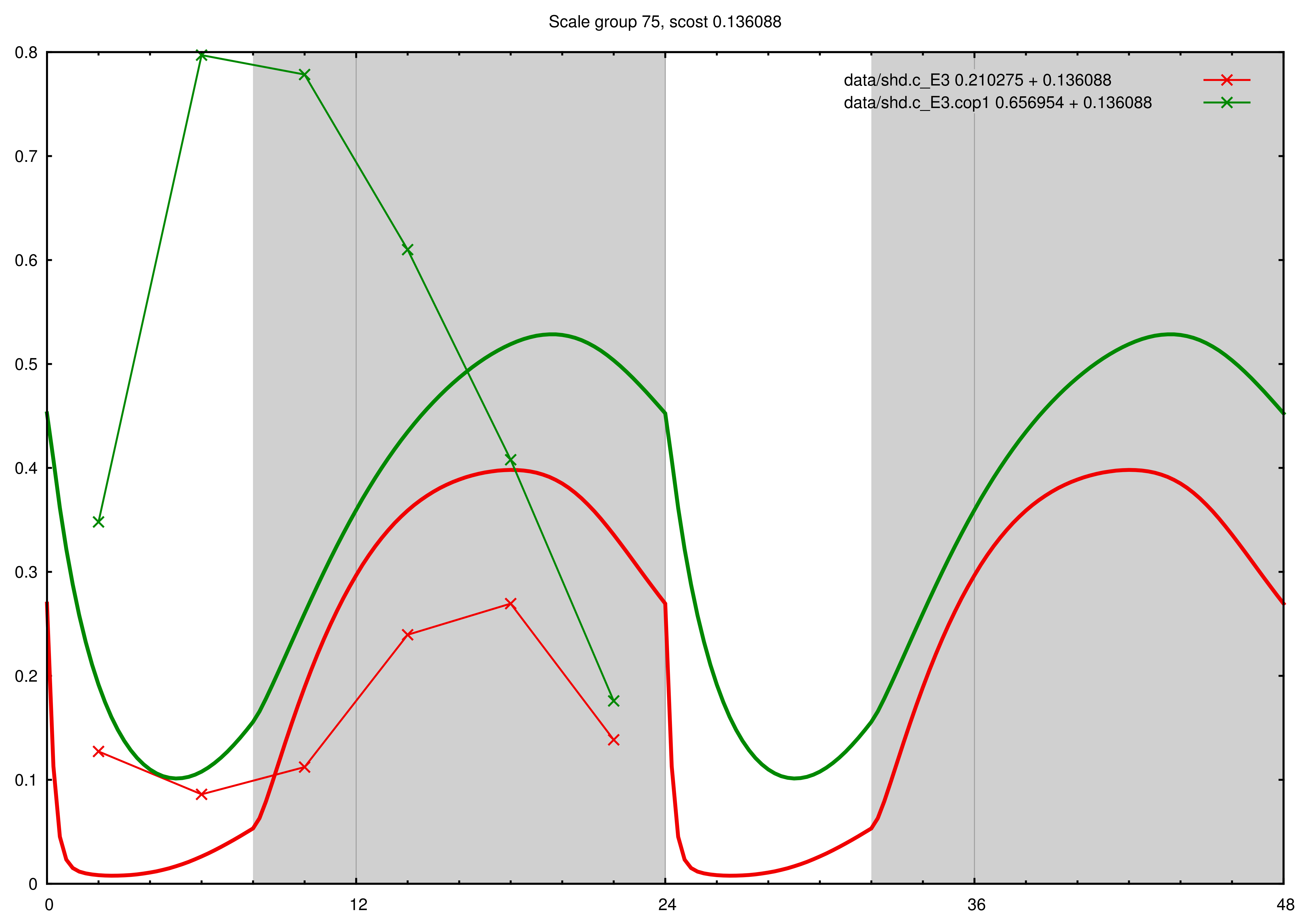

Scale group 75, scost 0.136088
data/shd.c_E3 0.210275 + 0.136088
data/shd.c_E3.cop1 0.656954 + 0.136088
0.8
0.7
0.6
0.5
0.4
0.3
0.2
0.1
0
0
12
24
36
48

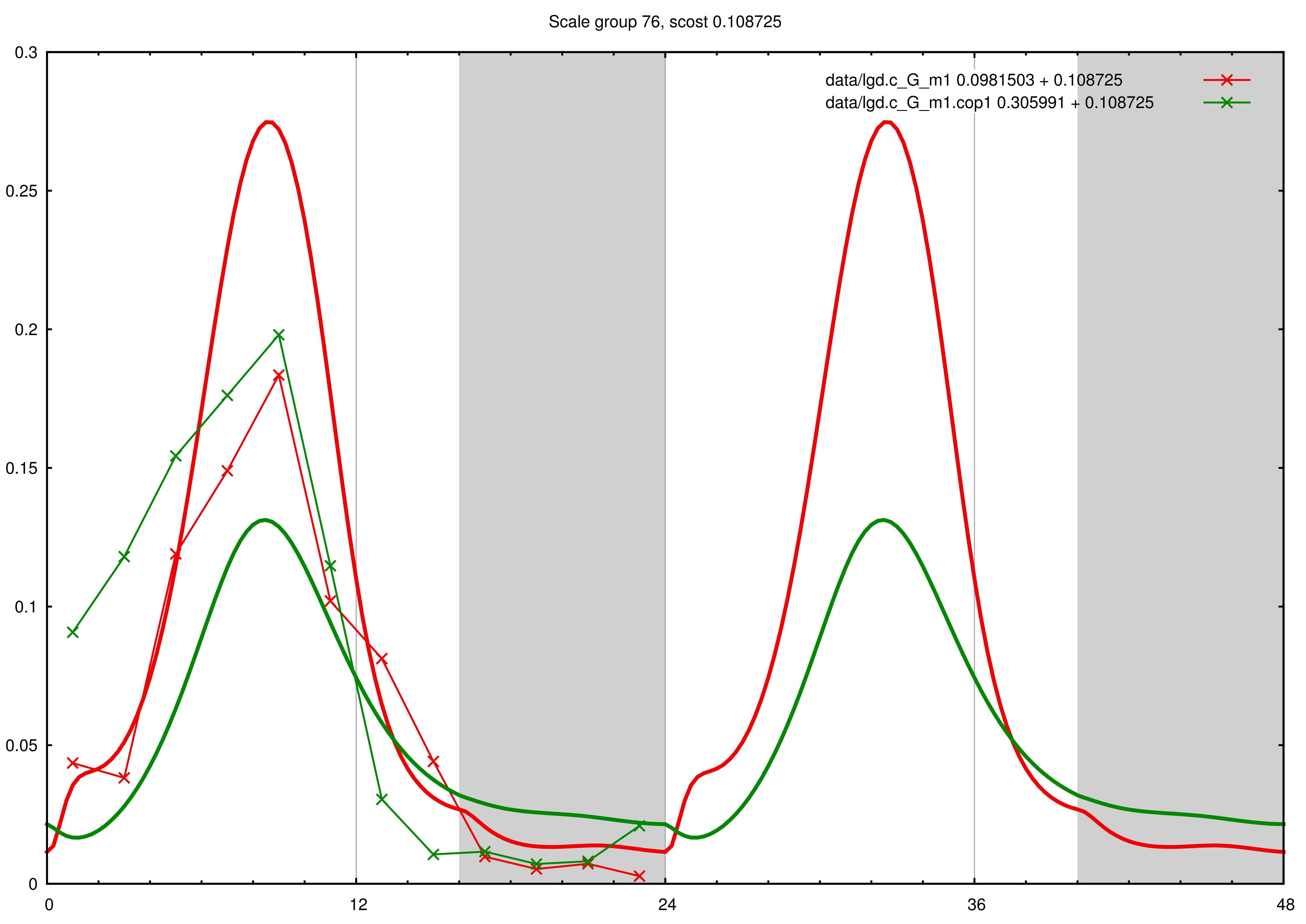
Scale group 76, scost 0.108725
data/lgd.c_G_m1 0.0981503 + 0.108725
data/lgd.c_G_m1.cop1 0.305991 + 0.108725
0.3
0.25
0.2
0.15
0.1
0.05
0
0
12
24
36
48

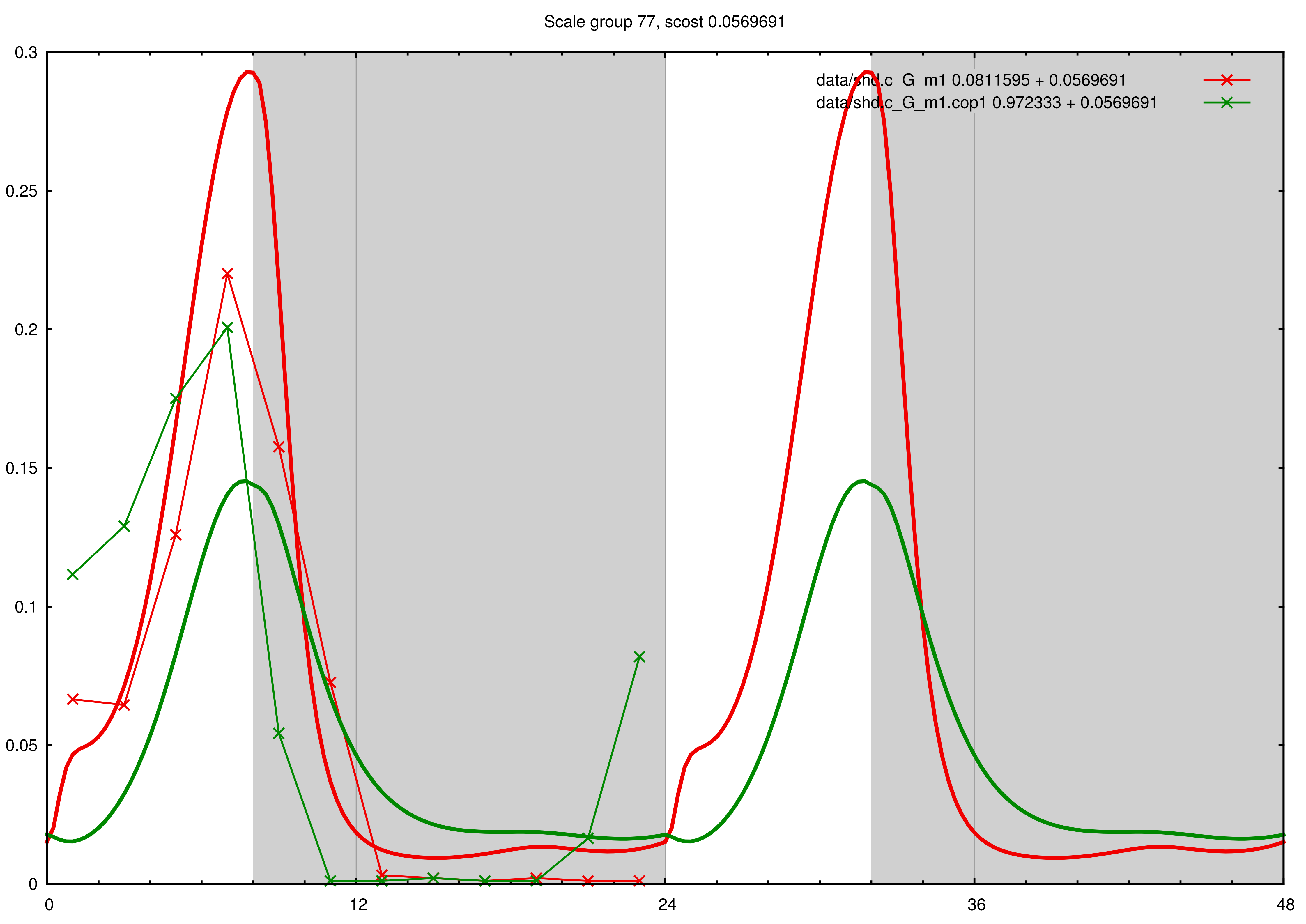

Scale group 77, scost 0.0569691
data/shd.c_G_m1 0.0811595 + 0.0569691
data/shd.c_G_m1.cop1 0.972333 + 0.0569691
0.3
0.25
0.2
0.15
0.1
0.05
0
0
12
24
36
48

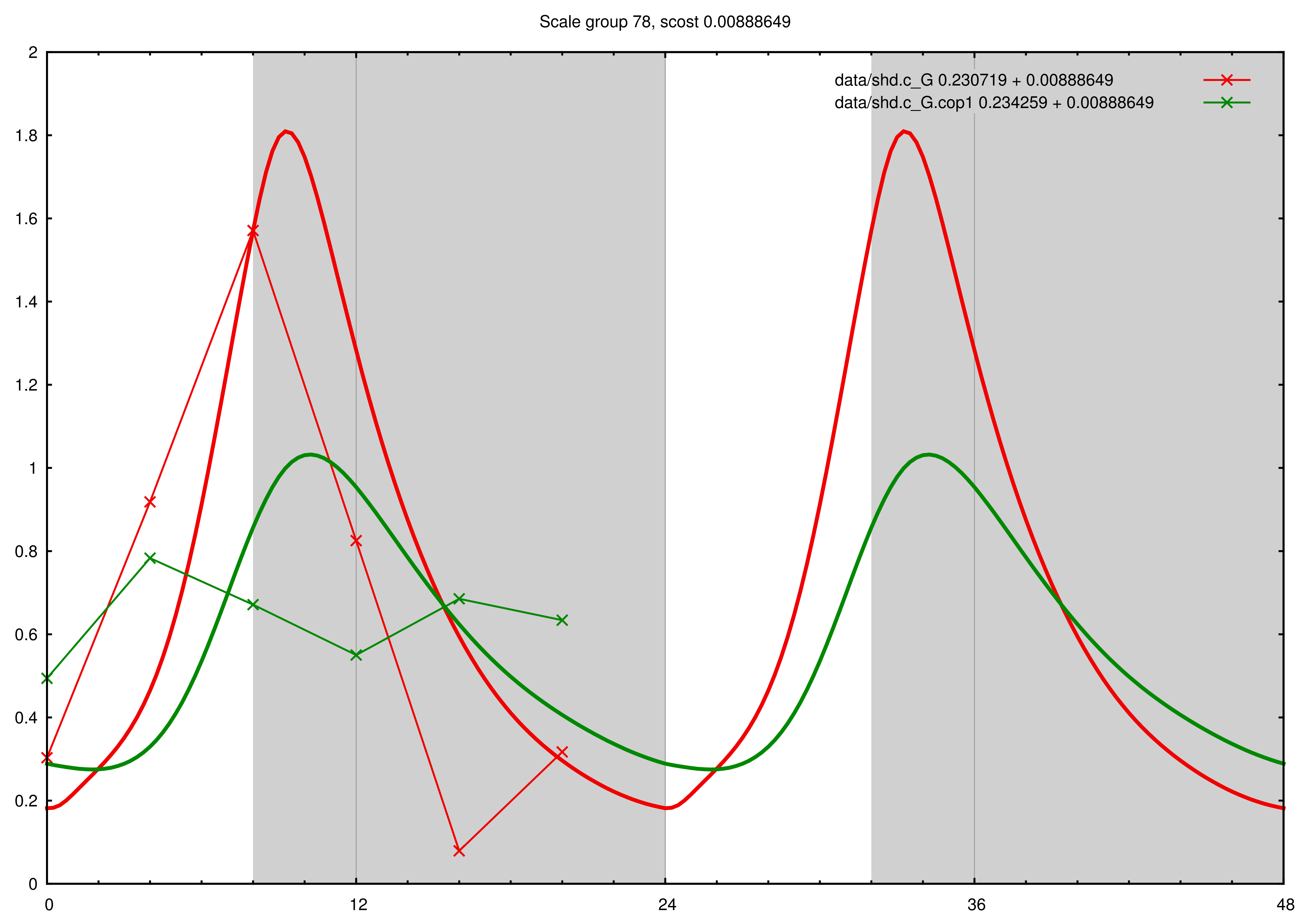

Scale group 78, scost 0.00888649
data/shd.c_G 0.230719 + 0.00888649
data/shd.c_G.cop1 0.234259 + 0.00888649
2
1.8
1.6
1.4
1.2
1
0.8
0.6
0.4
0.2
0
0
12
24
36
48

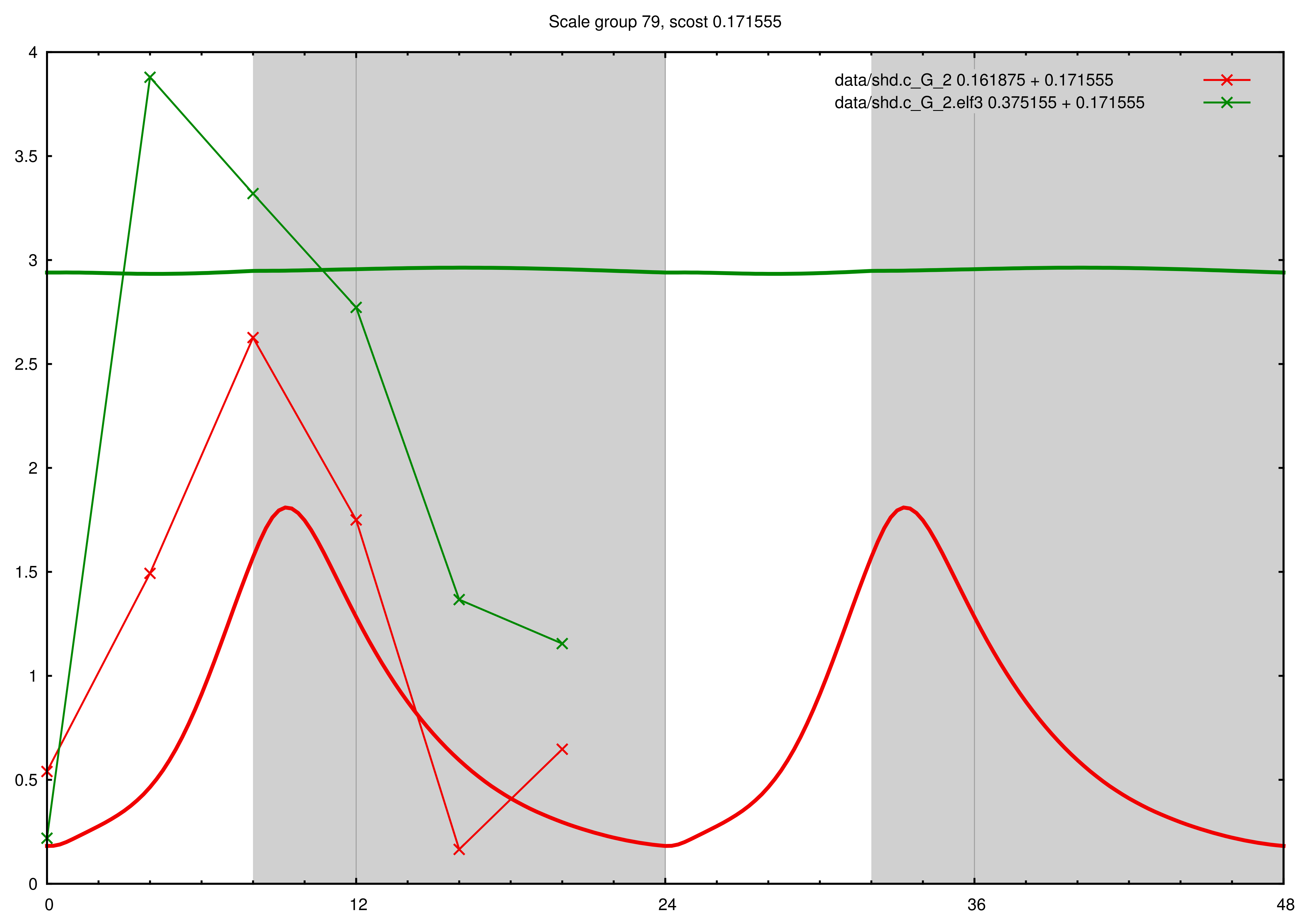
Scale group 79, scost 0.171555
data/shd.c_G_2 0.161875 + 0.171555
data/shd.c_G_2.elf3 0.375155 + 0.171555
4
3.5
3
2.5
2
1.5
1
0.5
0
0
12
24
36
48

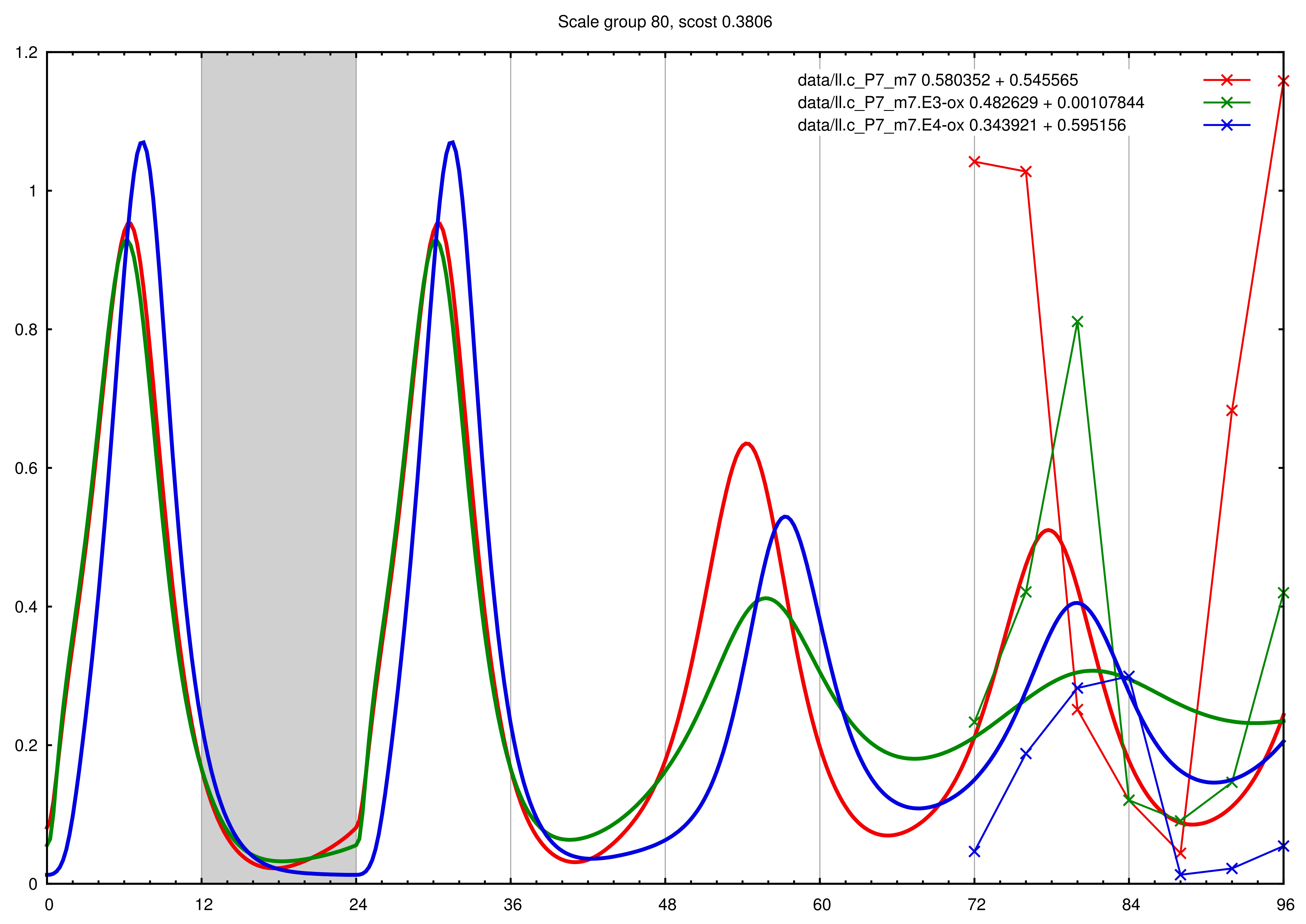

Scale group 80, scost 0.3806
data/ll.c_P7_m7 0.580352 + 0.545565
data/ll.c_P7_m7.E3-ox 0.482629 + 0.00107844
data/ll.c_P7_m7.E4-ox 0.343921 + 0.595156
1.2
1
0.8
0.6
0.4
0.2
0
0
12
24
36
48
60
72
84
96

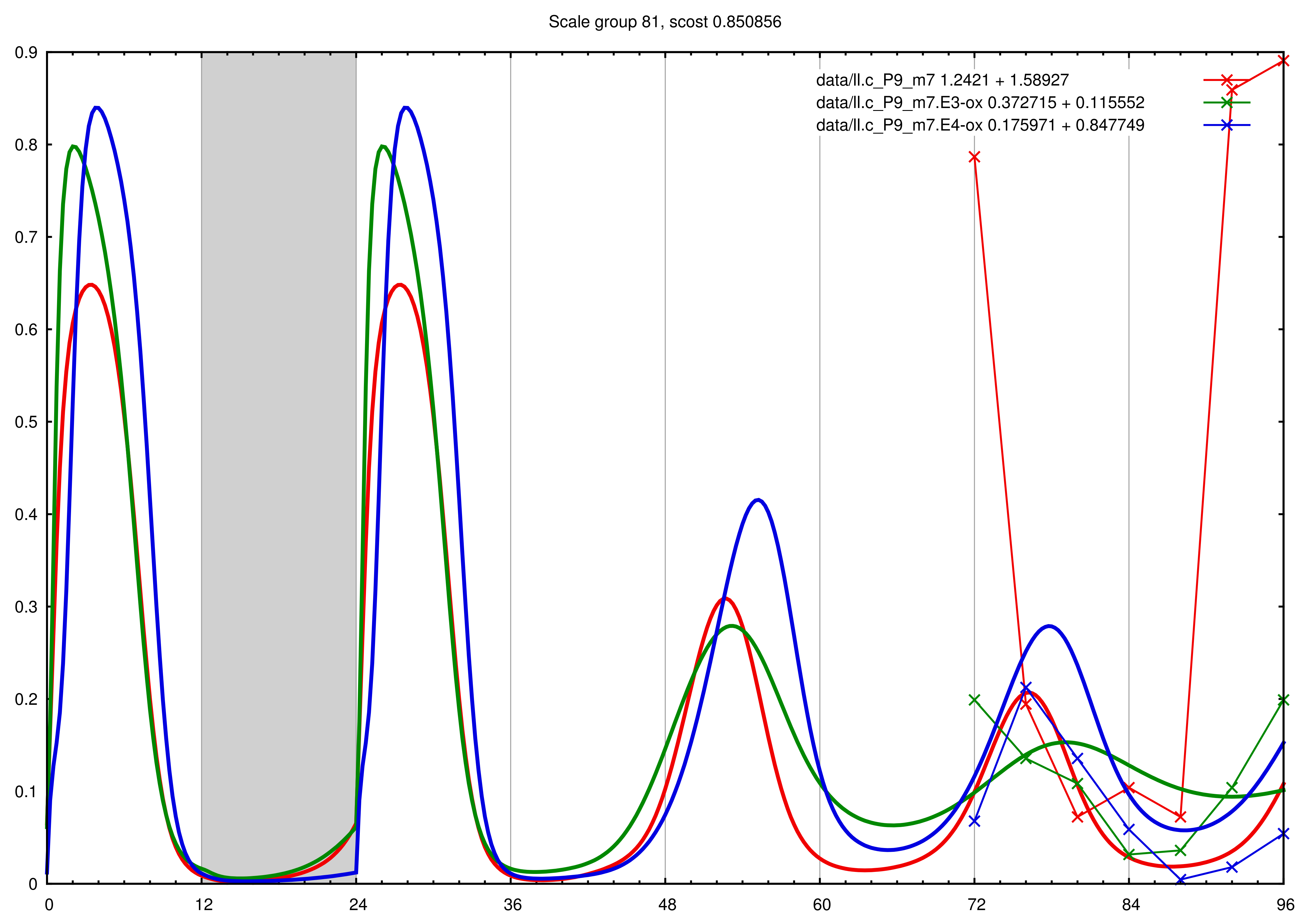
Scale group 81, scost 0.850856
data/ll.c_P9_m7 1.2421 + 1.58927
data/ll.c_P9_m7.E3-ox 0.372715 + 0.115552
data/ll.c_P9_m7.E4-ox 0.175971 + 0.847749
0
0.1
0.2
0.3
0.4
0.5
0.6
0.7
0.8
0.9
0
12
24
36
48
60
72
84
96

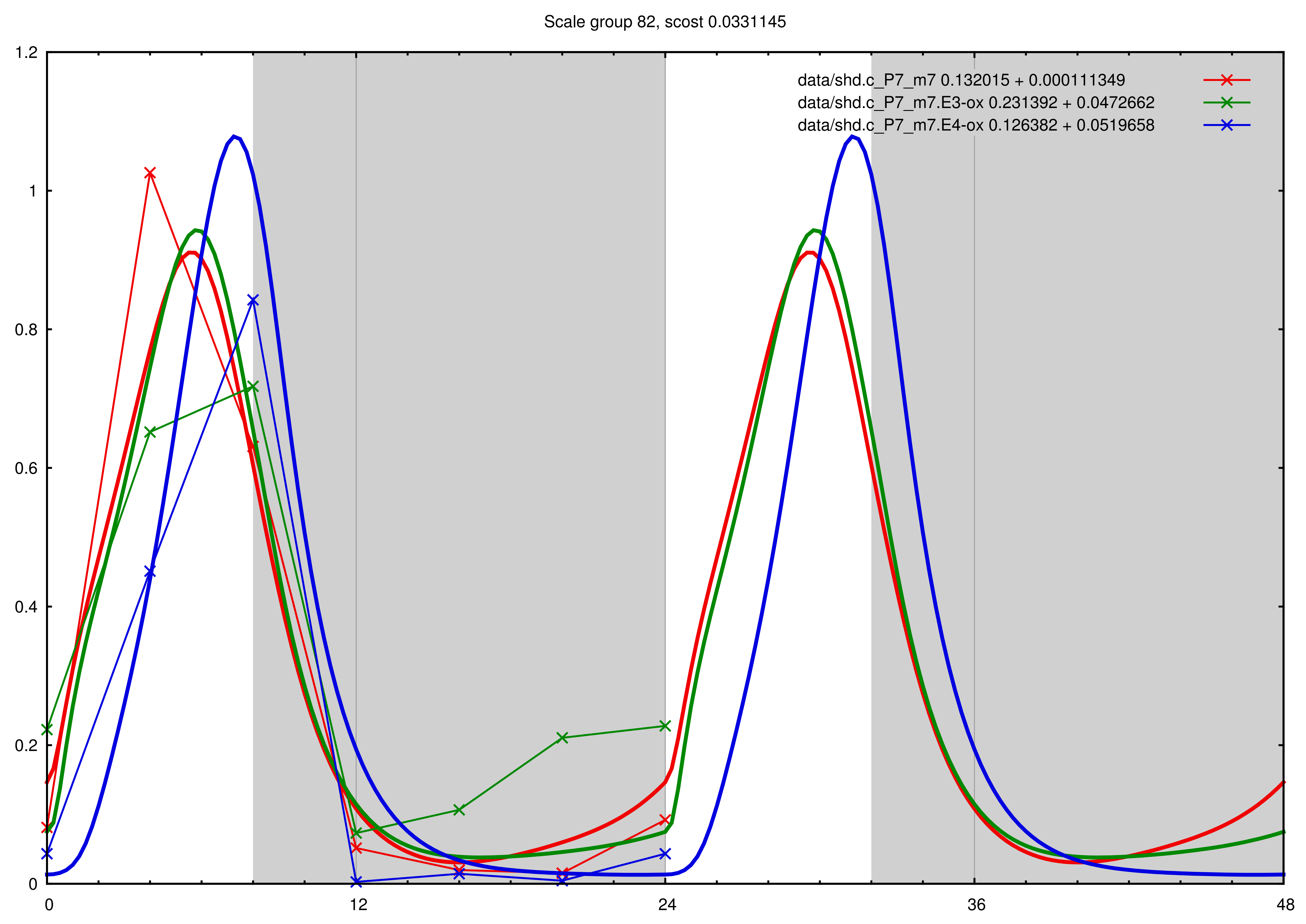
Scale group 82, scost 0.0331145
data/shd.c_P7_m7 0.132015 + 0.000111349
data/shd.c_P7_m7.E3-ox 0.231392 + 0.0472662
data/shd.c_P7_m7.E4-ox 0.126382 + 0.0519658
1.2
1
0.8
0.6
0.4
0.2
0
0
12
24
36
48

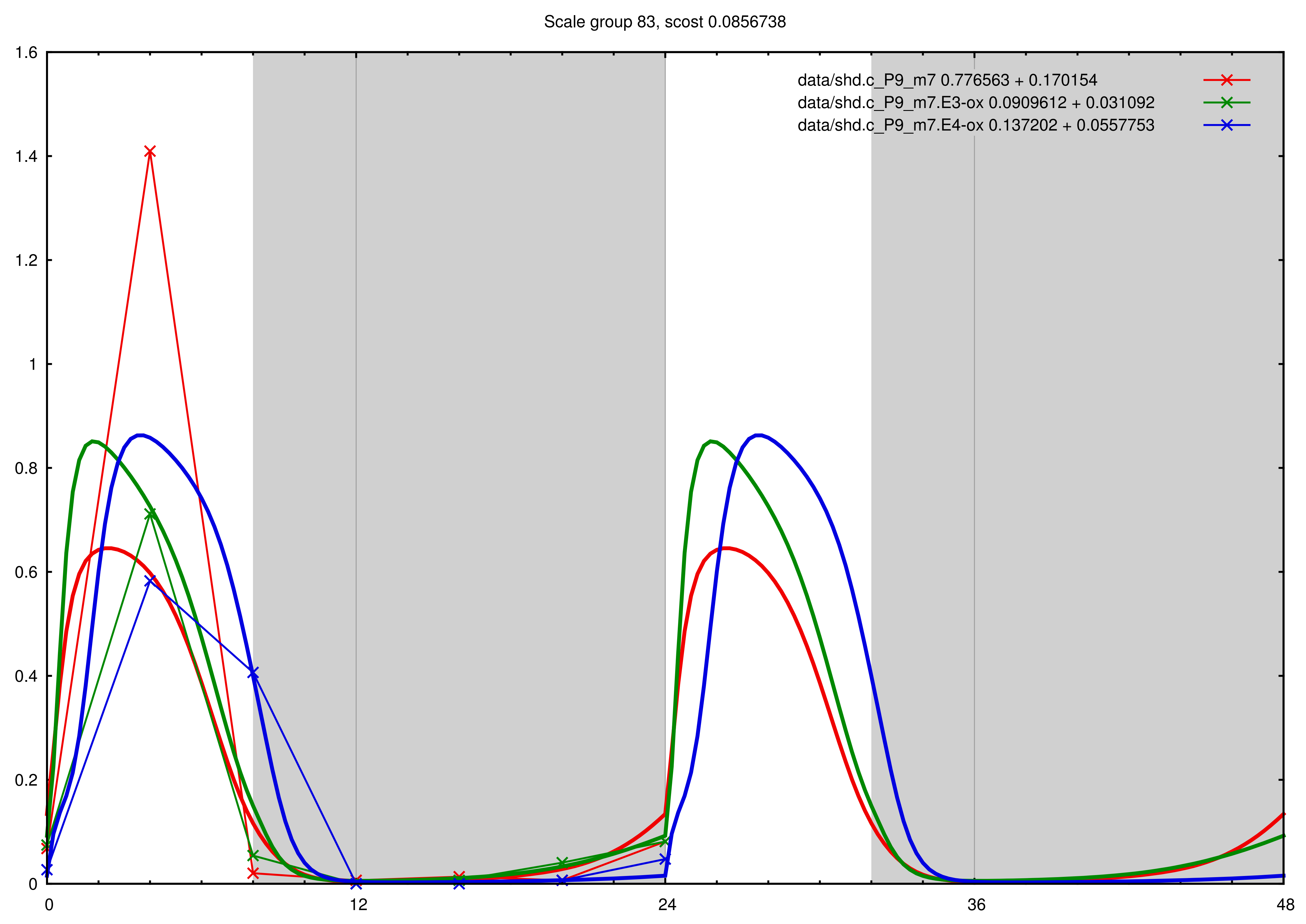
Scale group 83, scost 0.0856738
data/shd.c_P9_m7 0.776563 + 0.170154
data/shd.c_P9_m7.E3-ox 0.0909612 + 0.031092
data/shd.c_P9_m7.E4-ox 0.137202 + 0.0557753
1.6
1.4
1.2
1
0.8
0.6
0.4
0.2
0
0
12
24
36
48

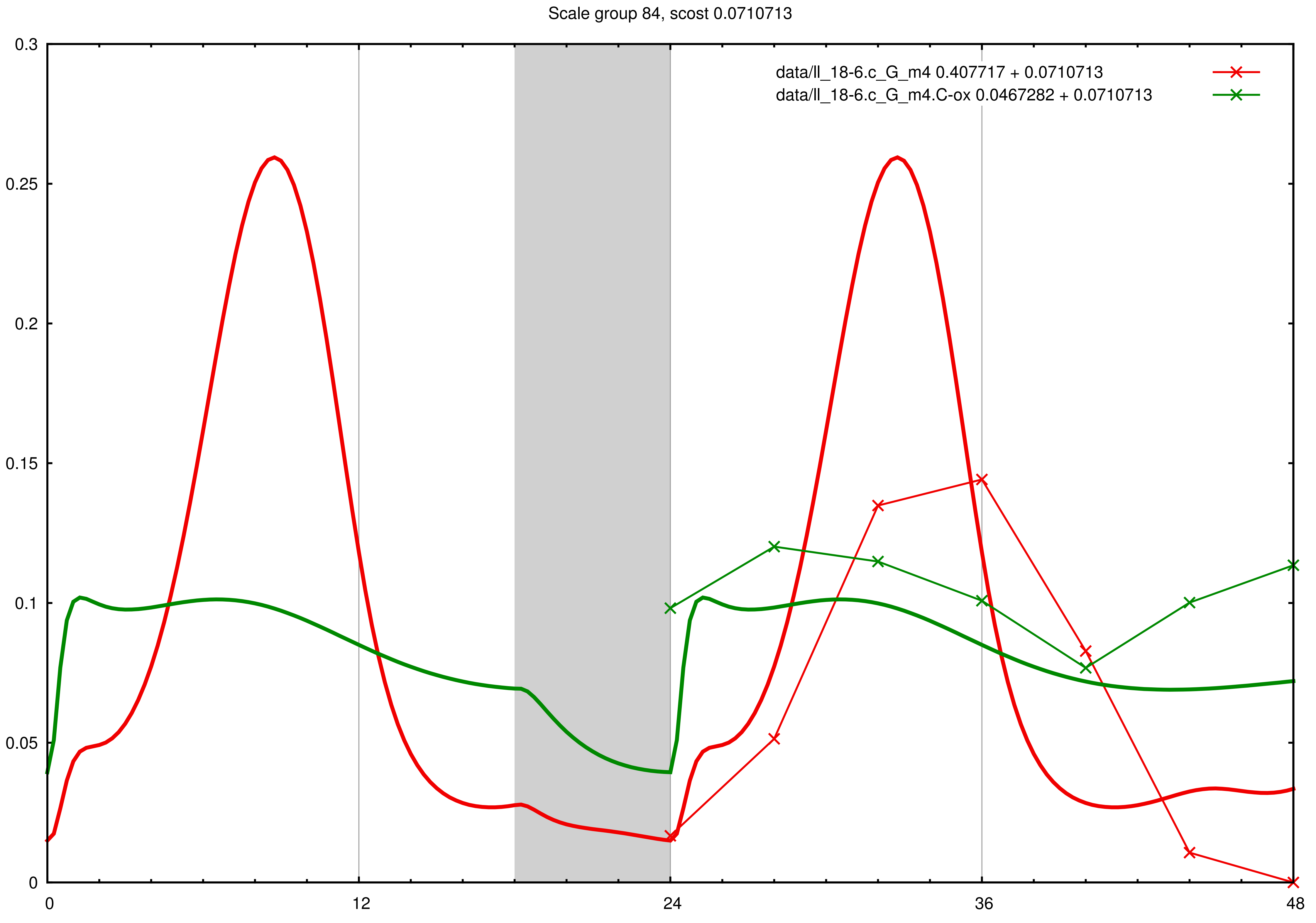

Scale group 84, scost 0.0710713
data/ll_18-6.c_G_m4 0.407717 + 0.0710713
data/ll_18-6.c_G_m4.C-ox 0.0467282 + 0.0710713
0.3
0.25
0.2
0.15
0.1
0.05
0
0
12
24
36
48

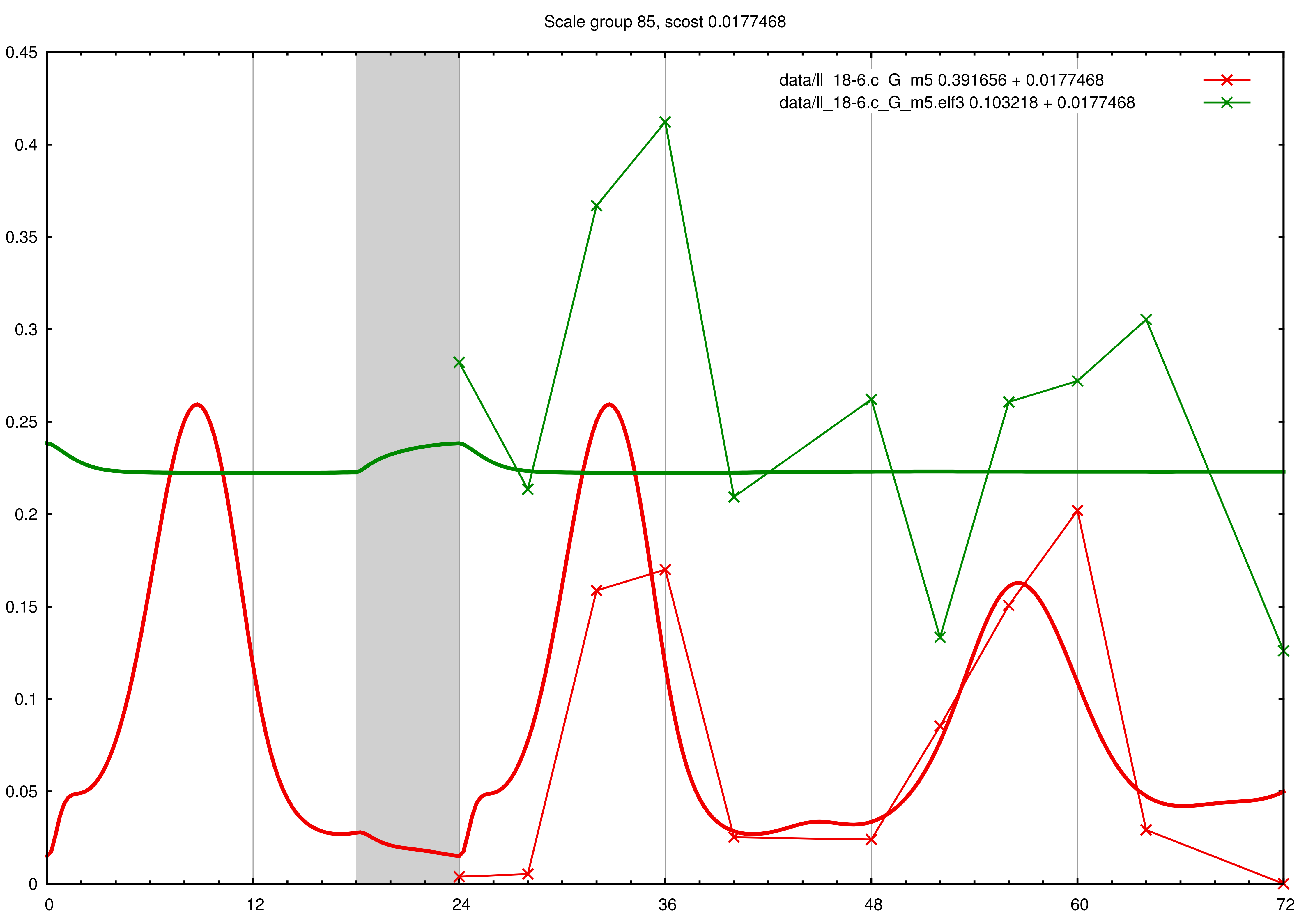

Scale group 85, scost 0.0177468
data/ll_18-6.c_G_m5 0.391656 + 0.0177468
data/ll_18-6.c_G_m5.elf3 0.103218 + 0.0177468
0.45
0.4
0.35
0.3
0.25
0.2
0.15
0.1
0.05
0
0
12
24
36
48
60
72

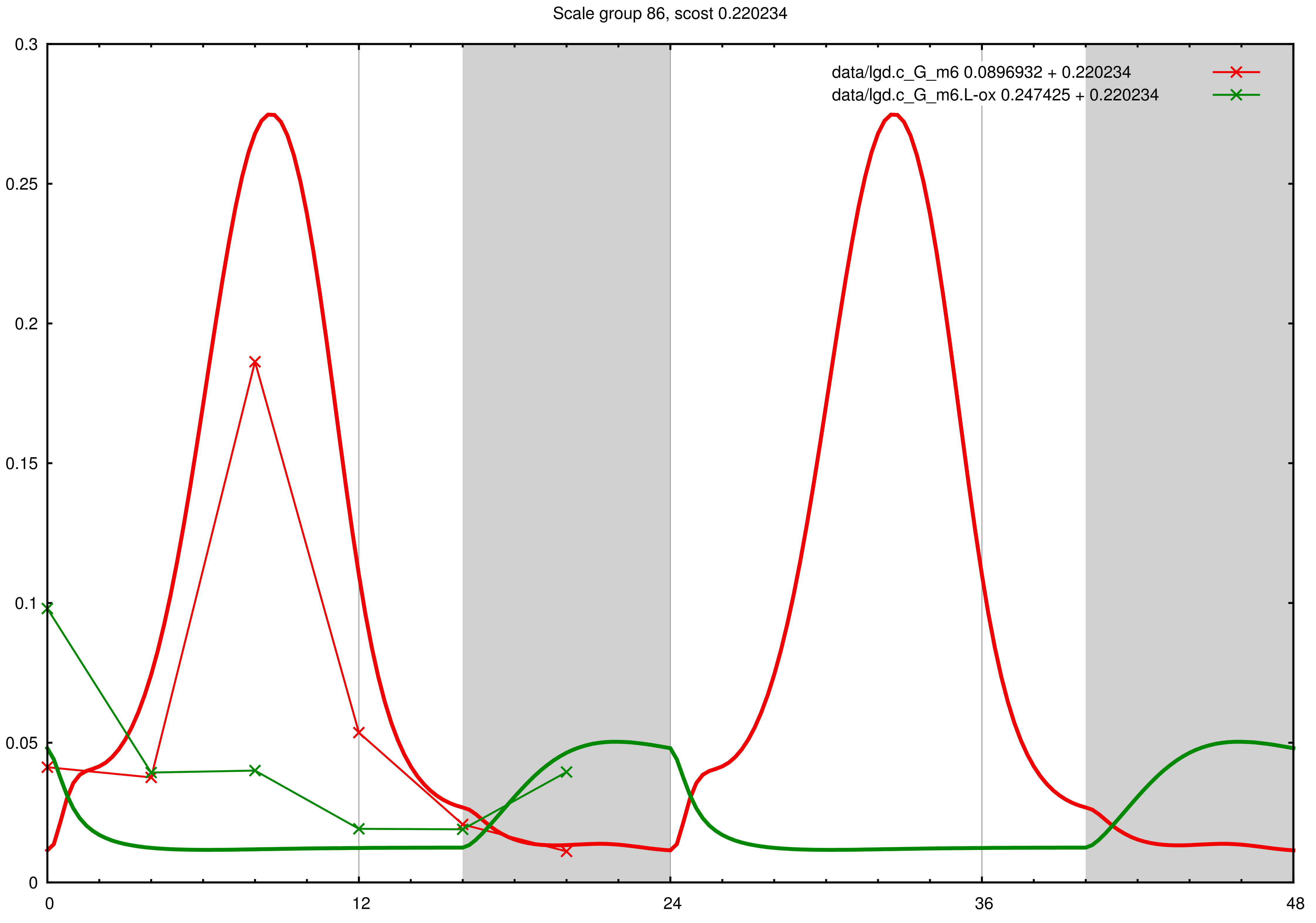
Scale group 86, scost 0.220234
data/lgd.c_G_m6 0.0896932 + 0.220234
data/lgd.c_G_m6.L-ox 0.247425 + 0.220234
0.3
0.25
0.2
0.15
0.1
0.05
0
0
12
24
36
48

Scale group 87, scost 0.000142744

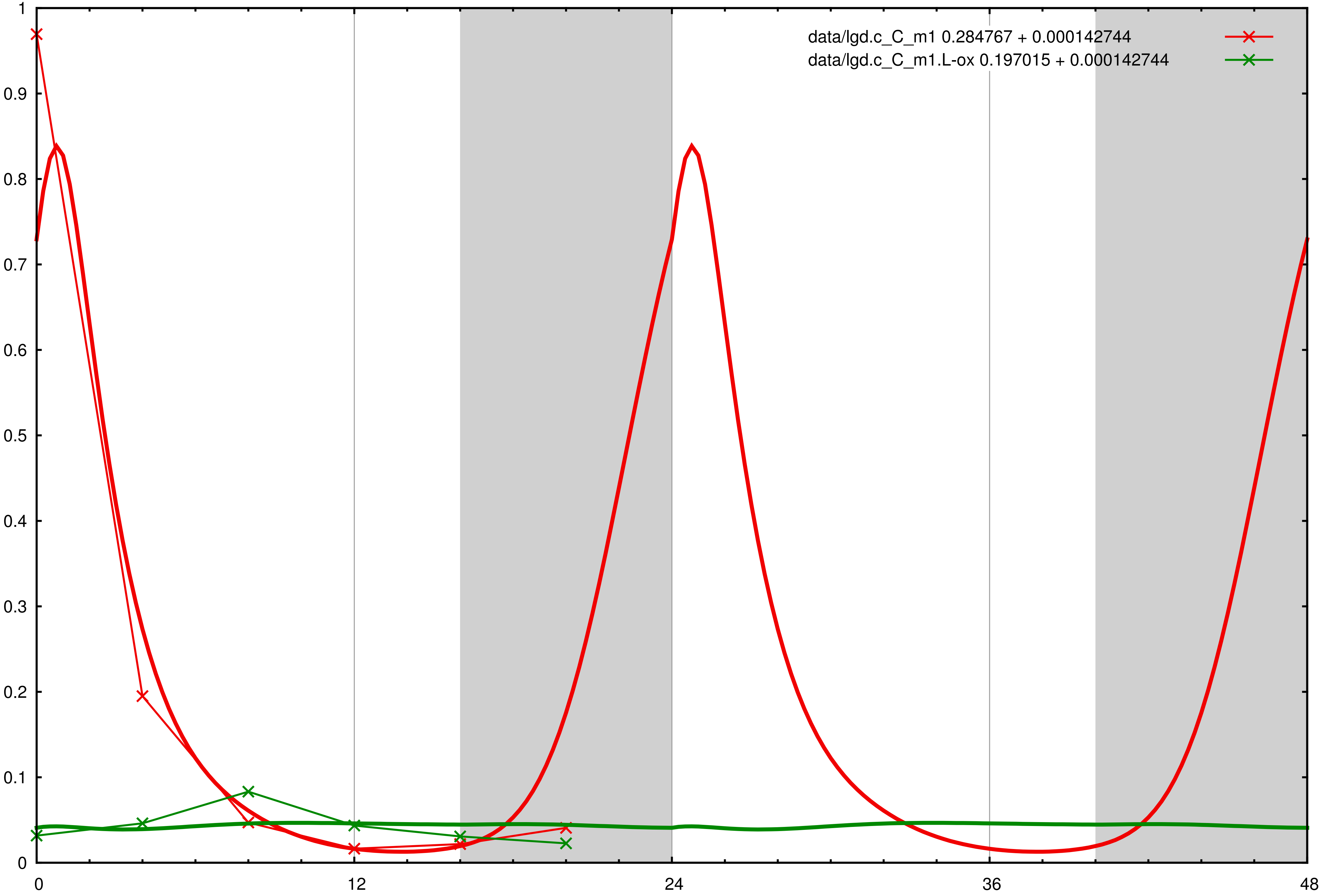

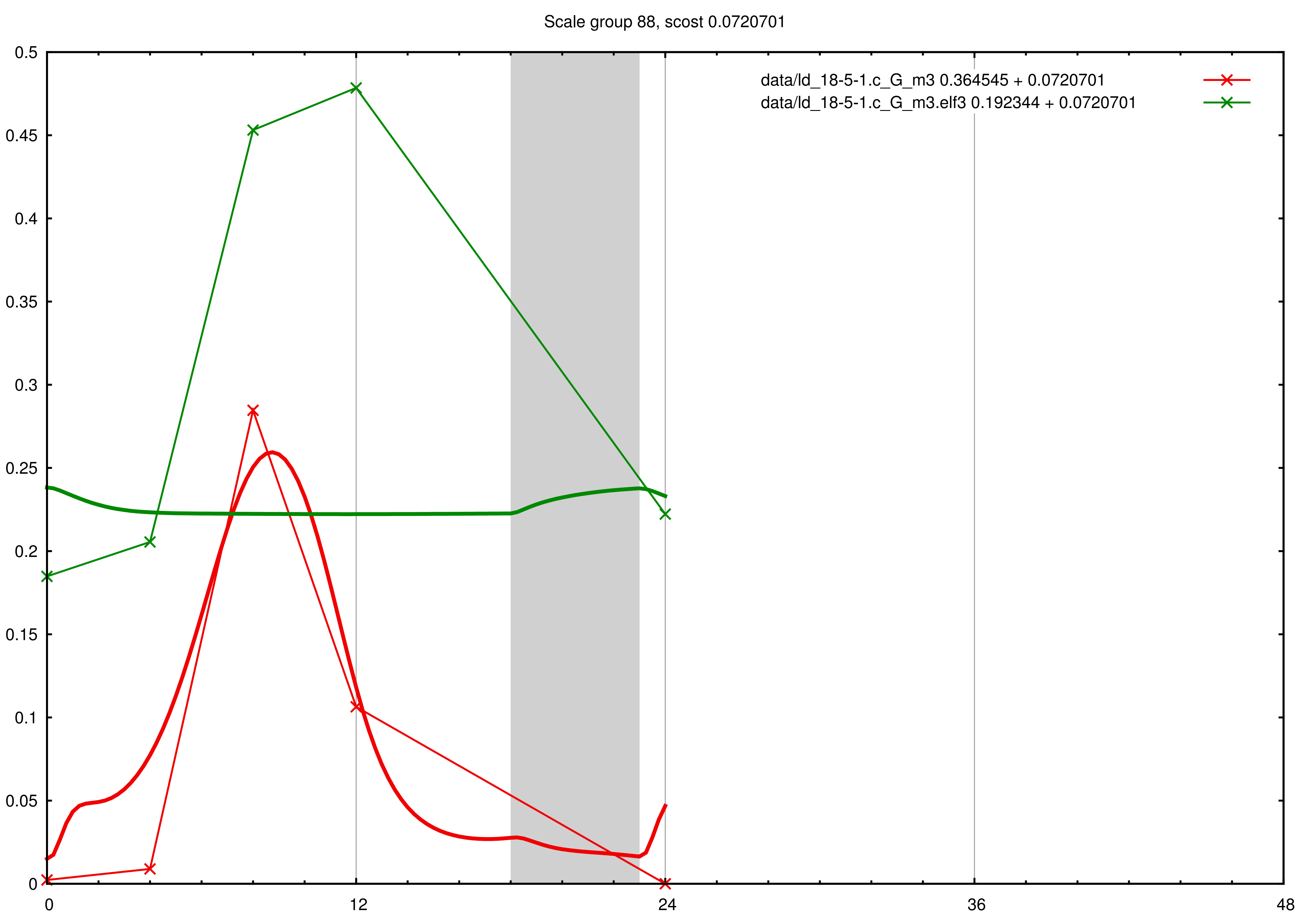
Scale group 88, scost 0.0720701
data/ld_18-5-1.c_G_m3 0.364545 + 0.0720701
data/ld_18-5-1.c_G_m3.elf3 0.192344 + 0.0720701
0.5
0.45
0.4
0.35
0.3
0.25
0.2
0.15
0.1
0.05
0
0
12
24
36
48

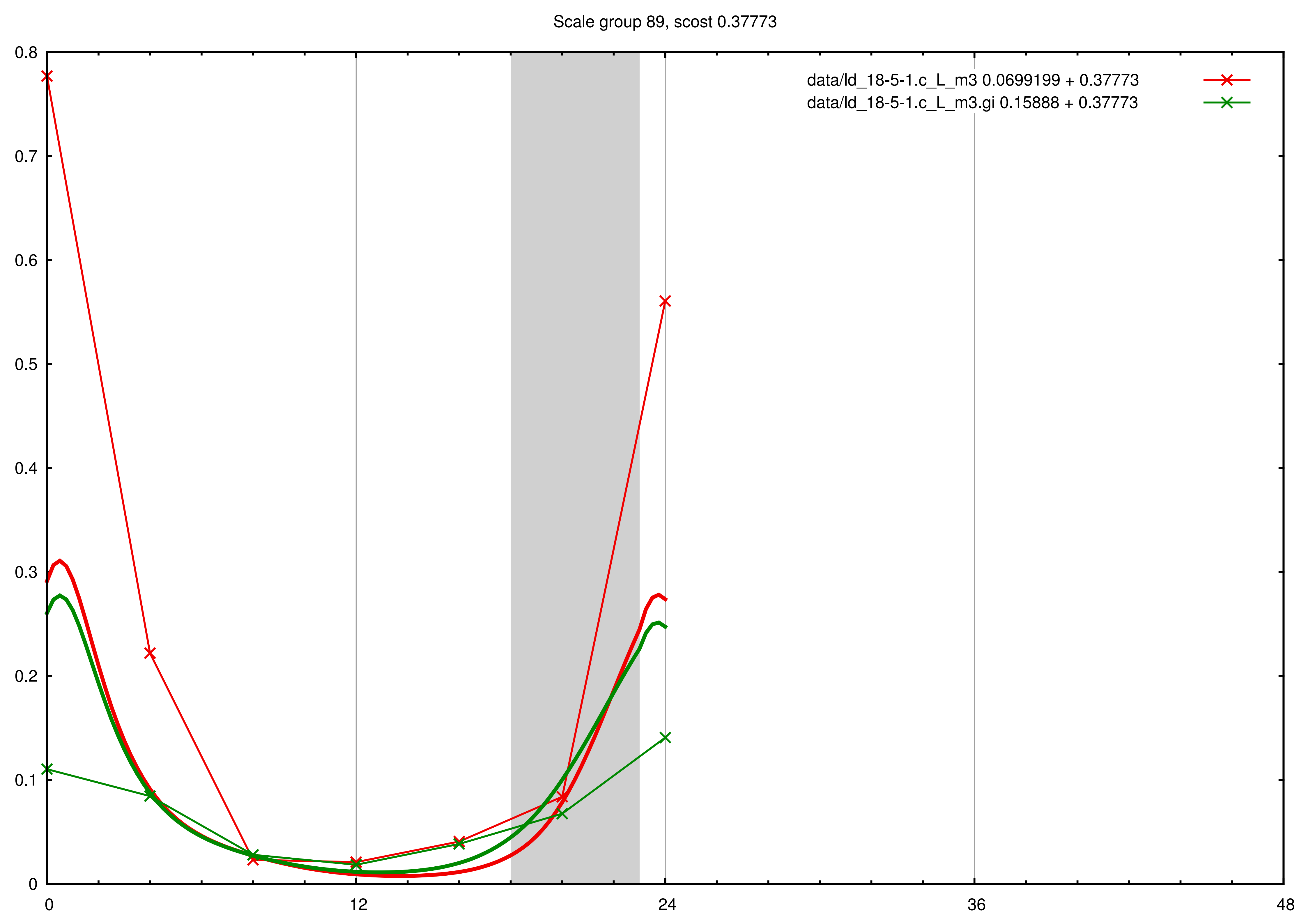

Scale group 89, scost 0.37773
data/ld_18-5-1.c_L_m3 0.0699199 + 0.37773
data/ld_18-5-1.c_L_m3.gi 0.15888 + 0.37773
0.8
0.7
0.6
0.5
0.4
0.3
0.2
0.1
0
0
12
24
36
48

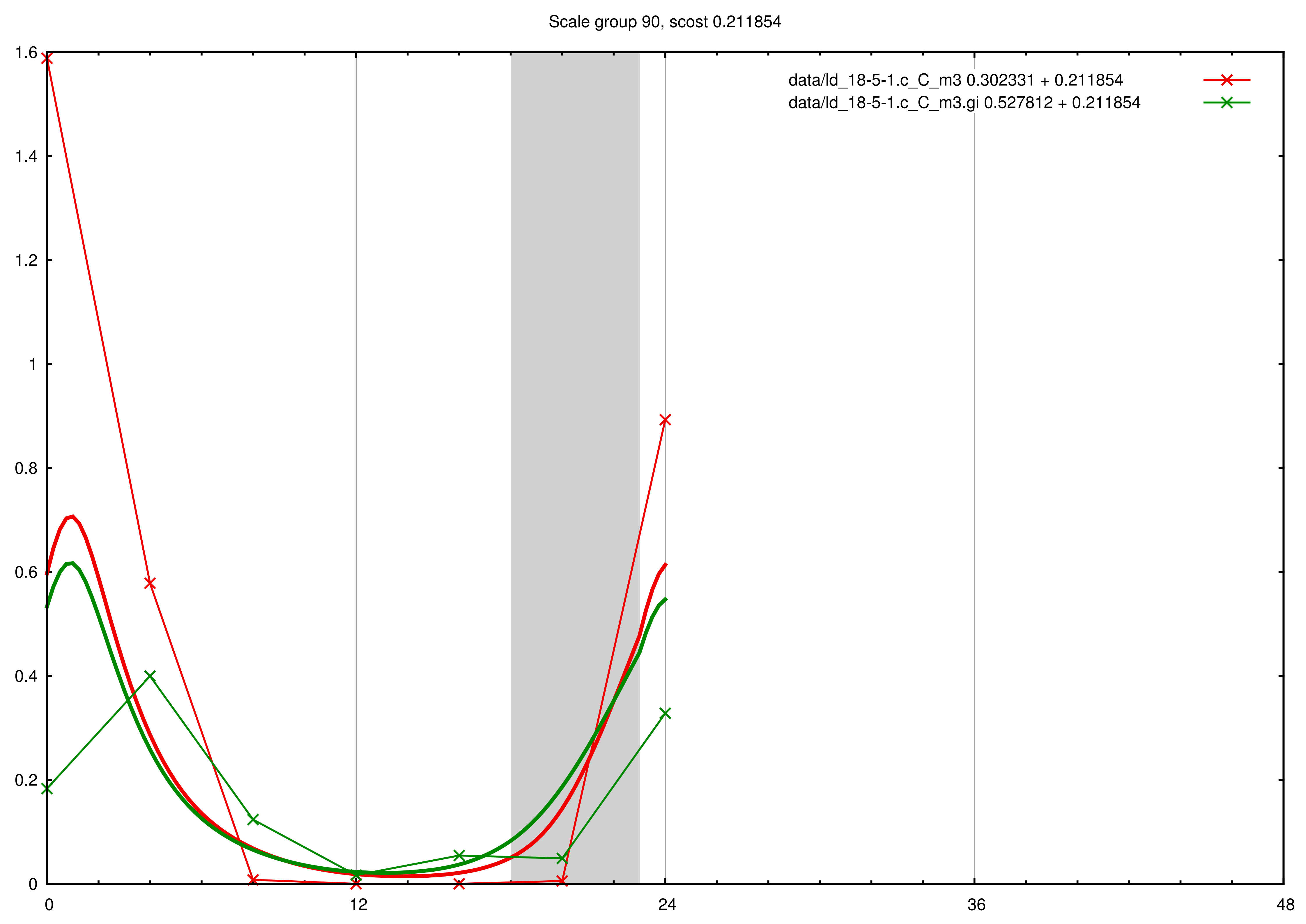
Scale group 90, scost 0.211854
data/ld_18-5-1.c_C_m3 0.302331 + 0.211854
data/ld_18-5-1.c_C_m3.gi 0.527812 + 0.211854
1.6
1.4
1.2
1
0.8
0.6
0.4
0.2
0
0
12
24
36
48

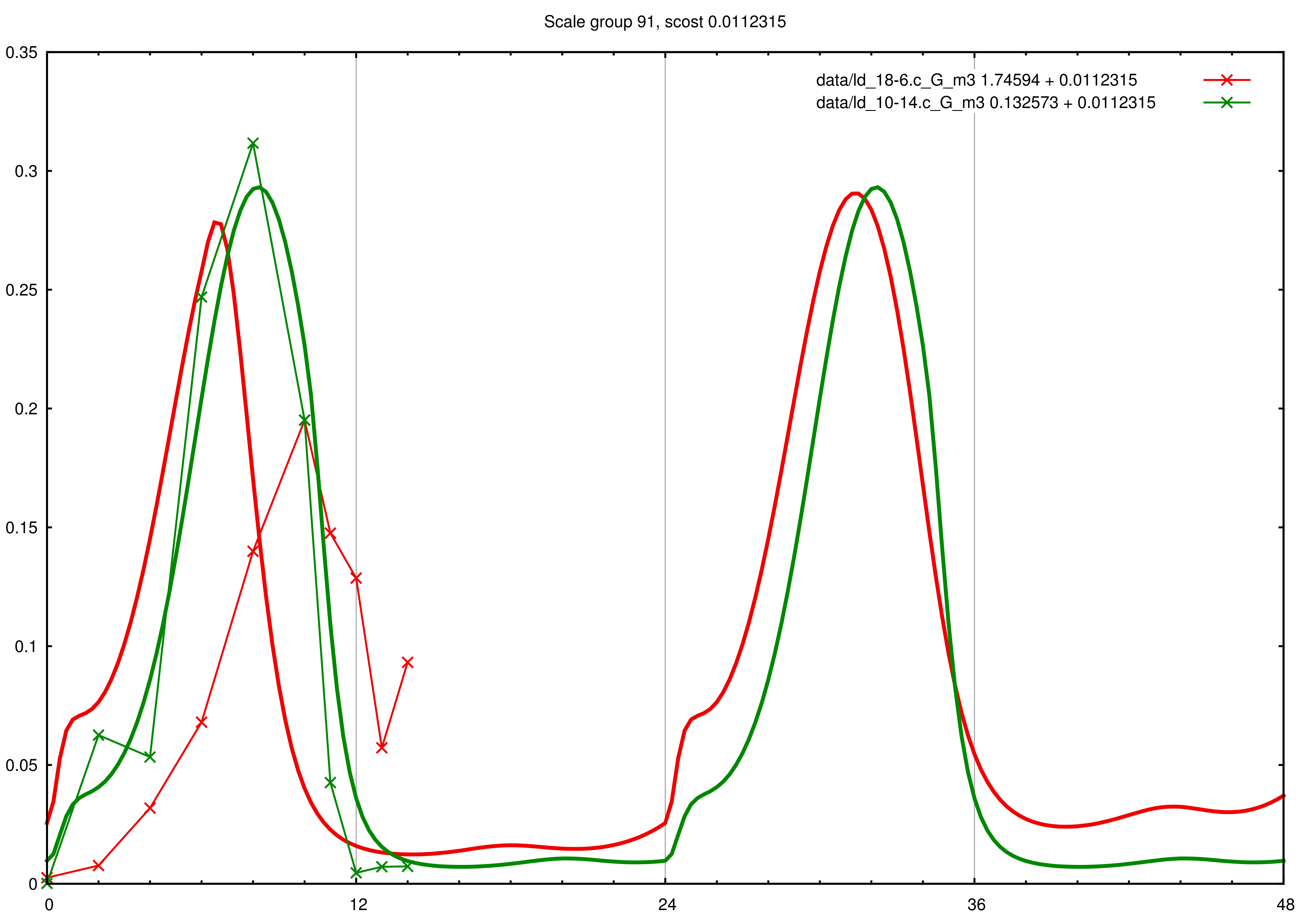
Scale group 91, scost 0.0112315
data/ld_18-6.c_G_m3 1.74594 + 0.0112315
data/ld_10-14.c_G_m3 0.132573 + 0.0112315
0.35
0.3
0.25
0.2
0.15
0.1
0.05
0
0
12
24
36
48

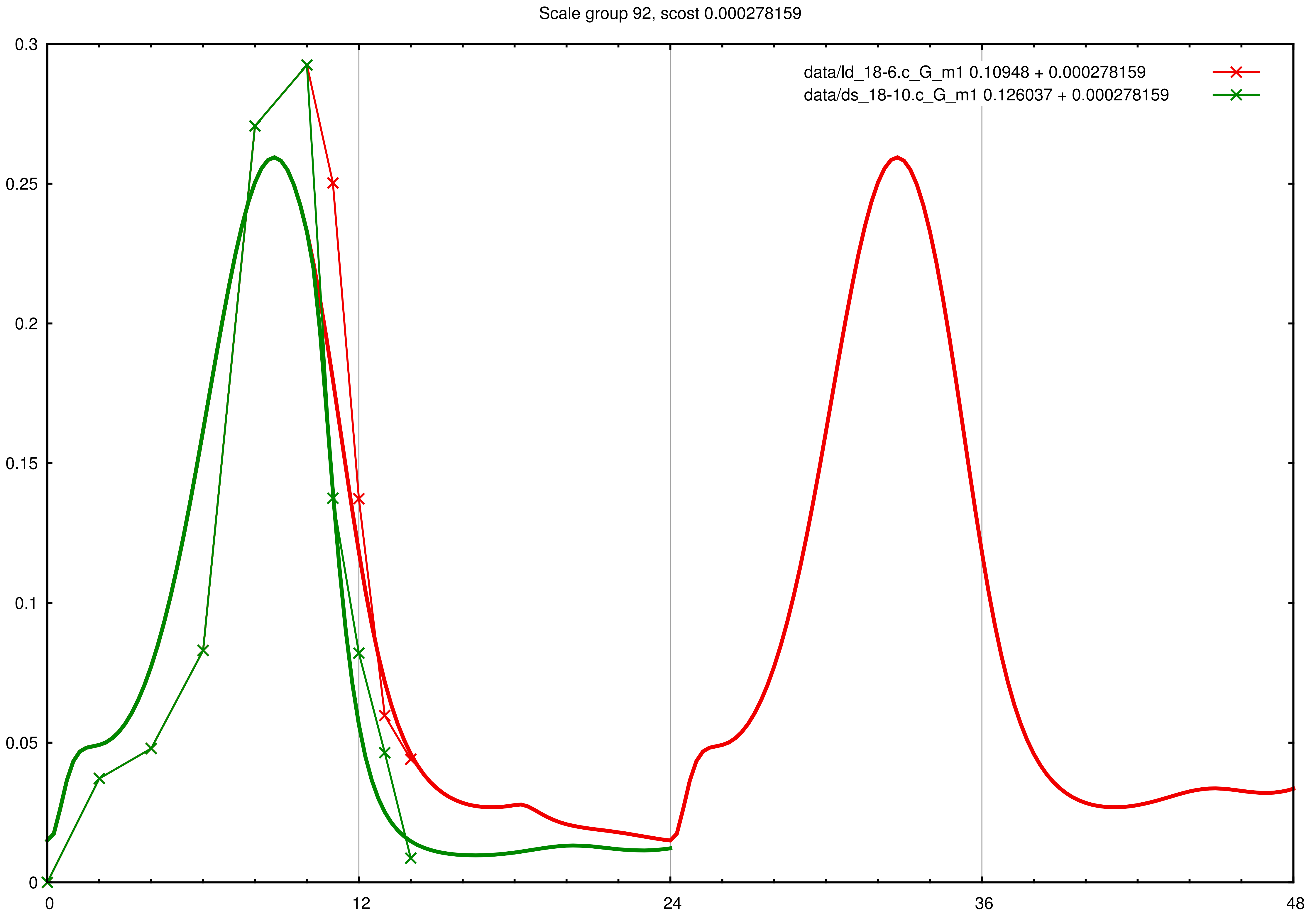

Scale group 92, scost 0.000278159
data/ld_18-6.c_G_m1 0.10948 + 0.000278159
data/ds_18-10.c_G_m1 0.126037 + 0.000278159
0.3
0.25
0.2
0.15
0.1
0.05
0
0
12
24
36
48

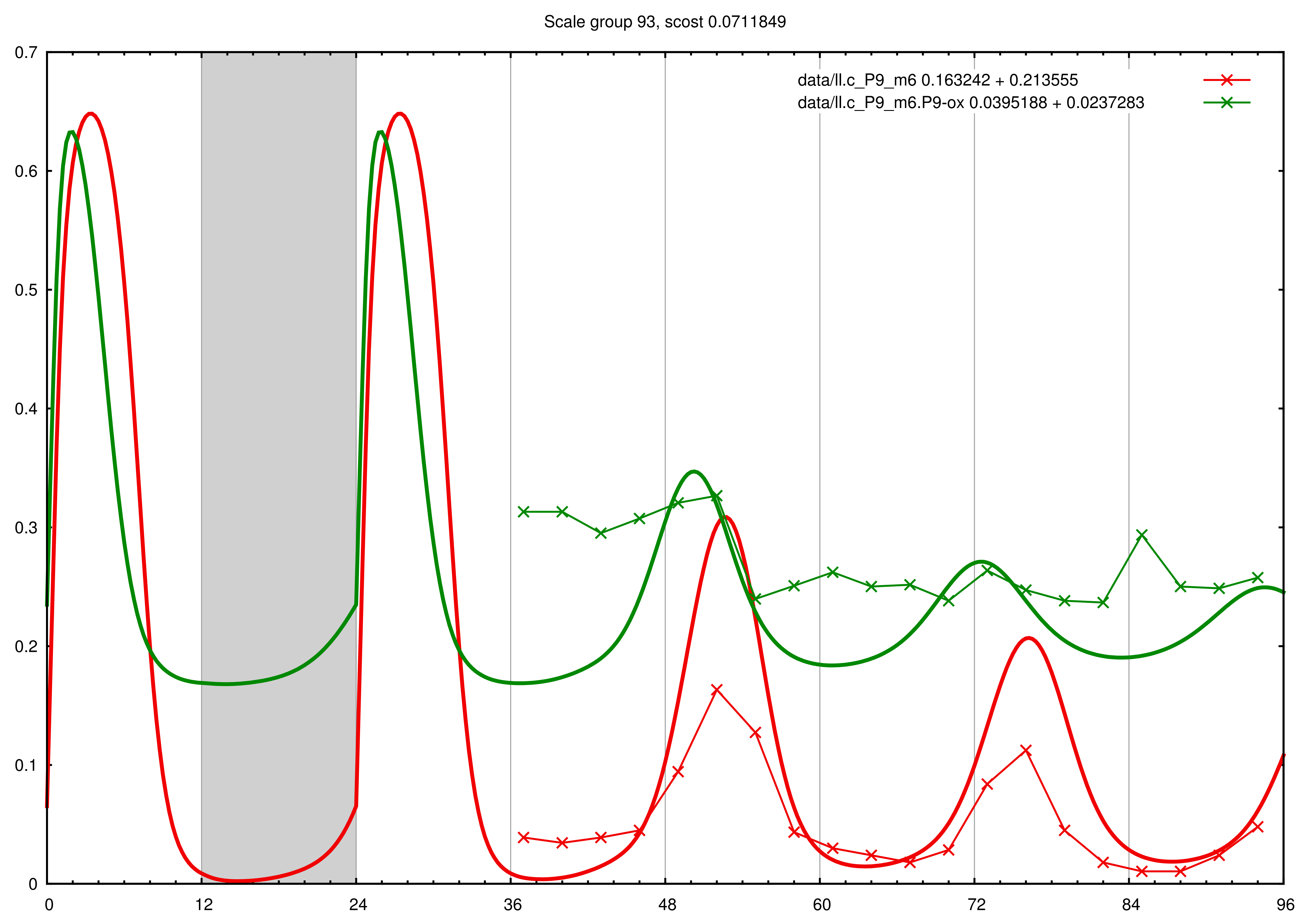

Scale group 93, scost 0.0711849
data/ll.c_P9_m6 0.163242 + 0.213555
data/ll.c_P9_m6.P9-ox 0.0395188 + 0.0237283
0.7
0.6
0.5
0.4
0.3
0.2
0.1
0
0
12
24
36
48
60
72
84
96

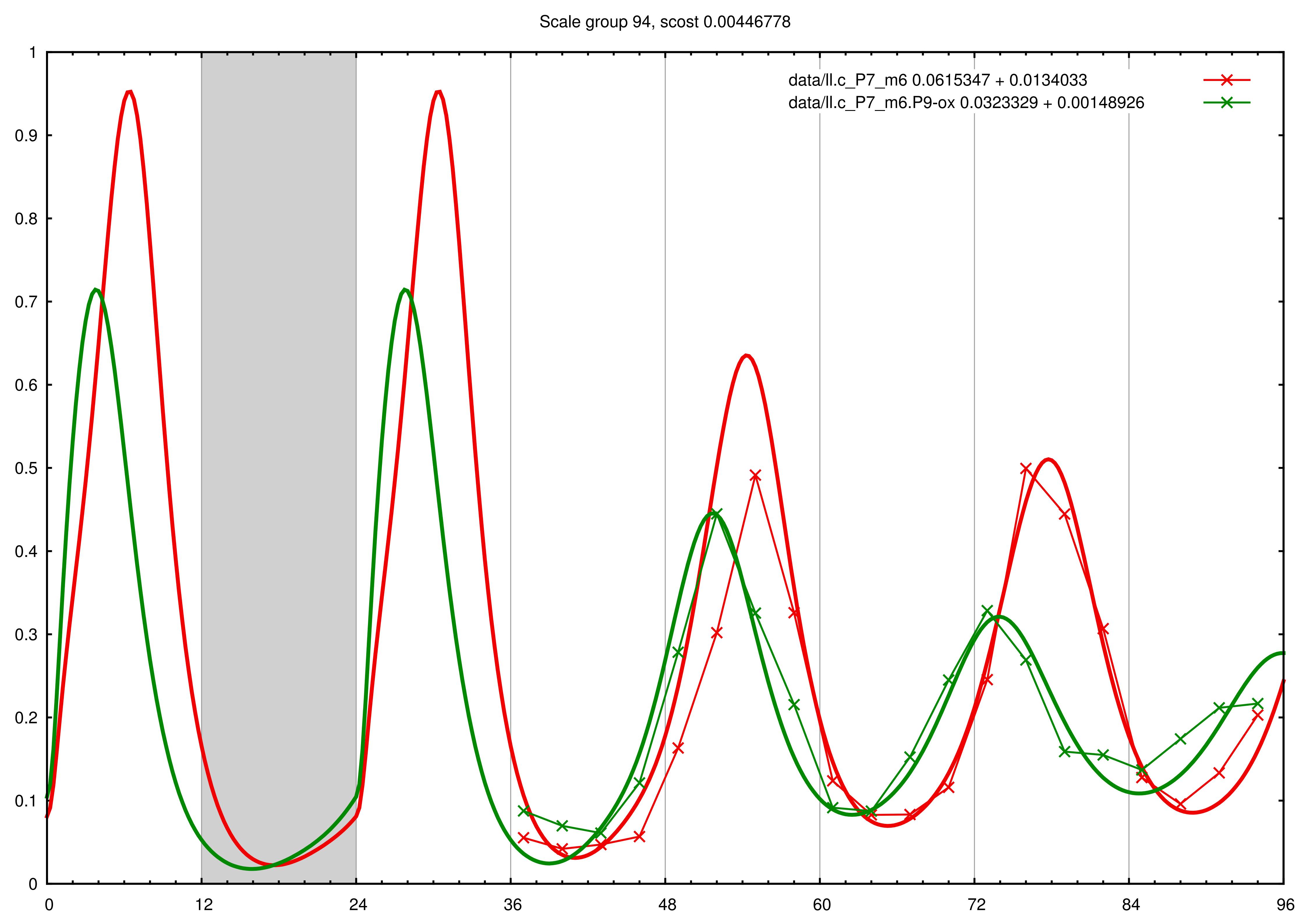

Scale group 94, scost 0.00446778
data/ll.c_P7_m6 0.0615347 + 0.0134033
data/ll.c_P7_m6.P9-ox 0.0323329 + 0.00148926
0
0.1
0.2
0.3
0.4
0.5
0.6
0.7
0.8
0.9
1
0
12
24
36
48
60
72
84
96

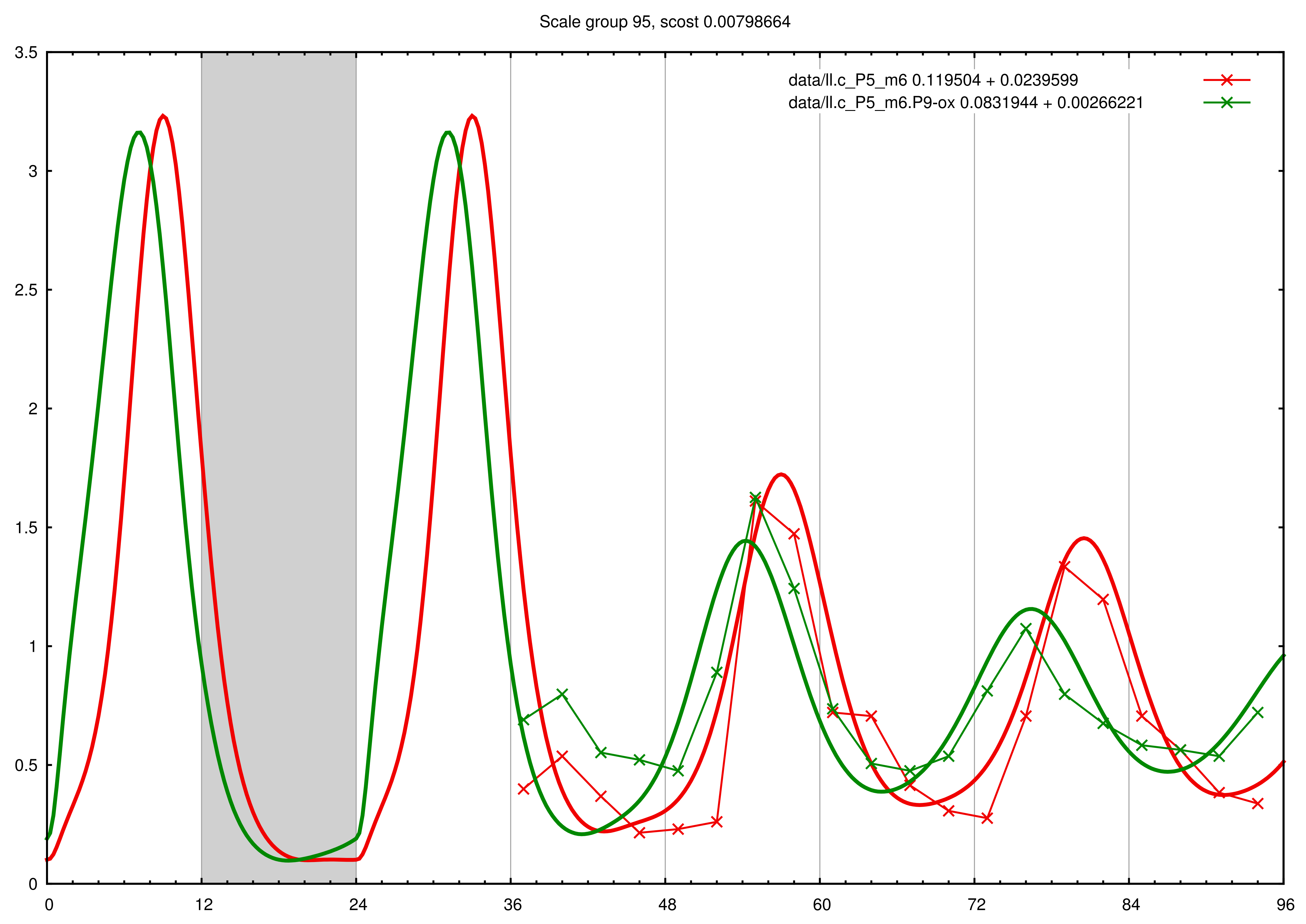

Scale group 95, scost 0.00798664
data/ll.c_P5_m6 0.119504 + 0.0239599
data/ll.c_P5_m6.P9-ox 0.0831944 + 0.00266221
3.5
3
2.5
2
1.5
1
0.5
0
0
12
24
36
48
60
72
84
96

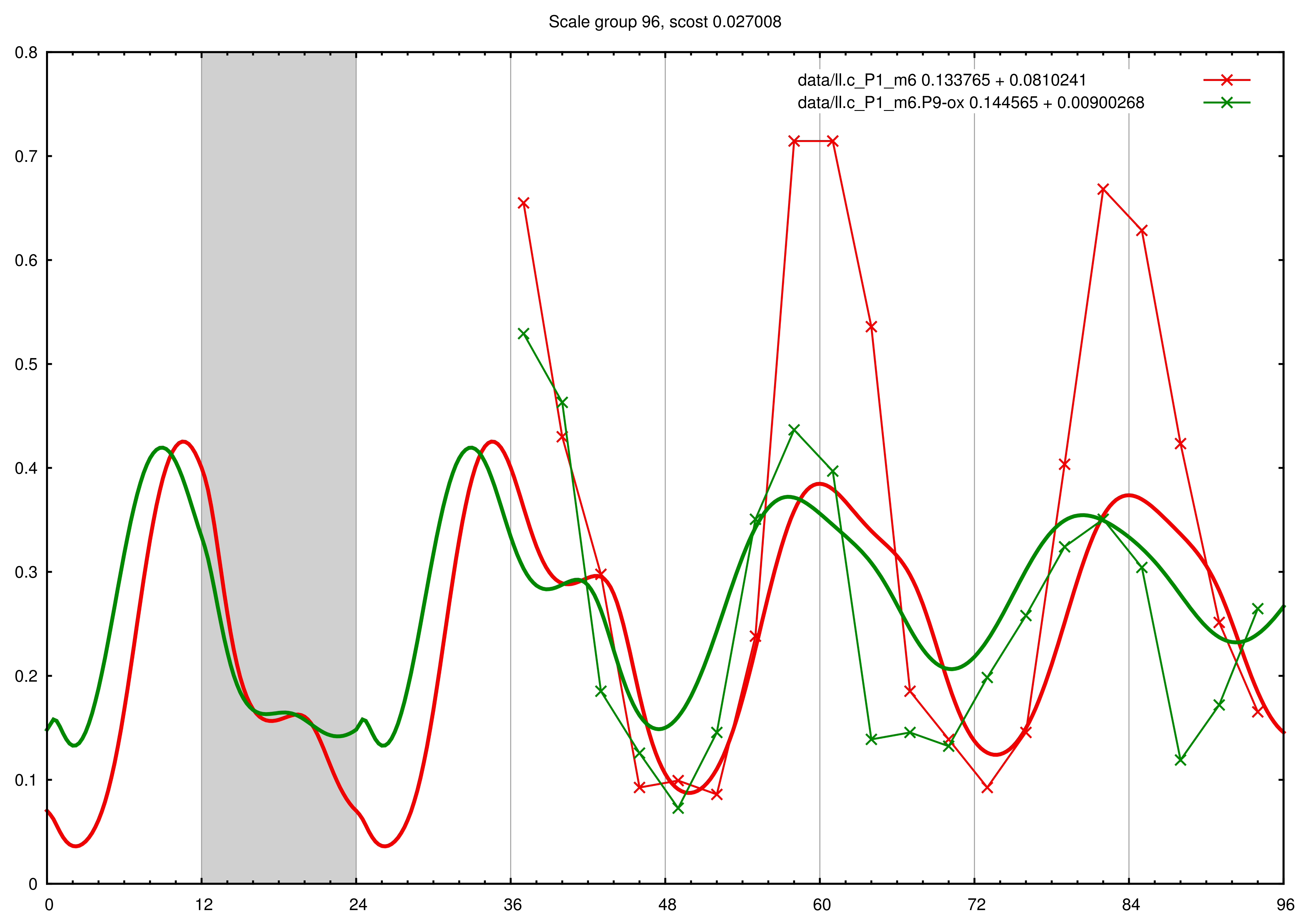
Scale group 96, scost 0.027008
data/ll.c_P1_m6 0.133765 + 0.0810241
data/ll.c_P1_m6.P9-ox 0.144565 + 0.00900268
0.8
0.7
0.6
0.5
0.4
0.3
0.2
0.1
0
0
12
24
36
48
60
72
84
96

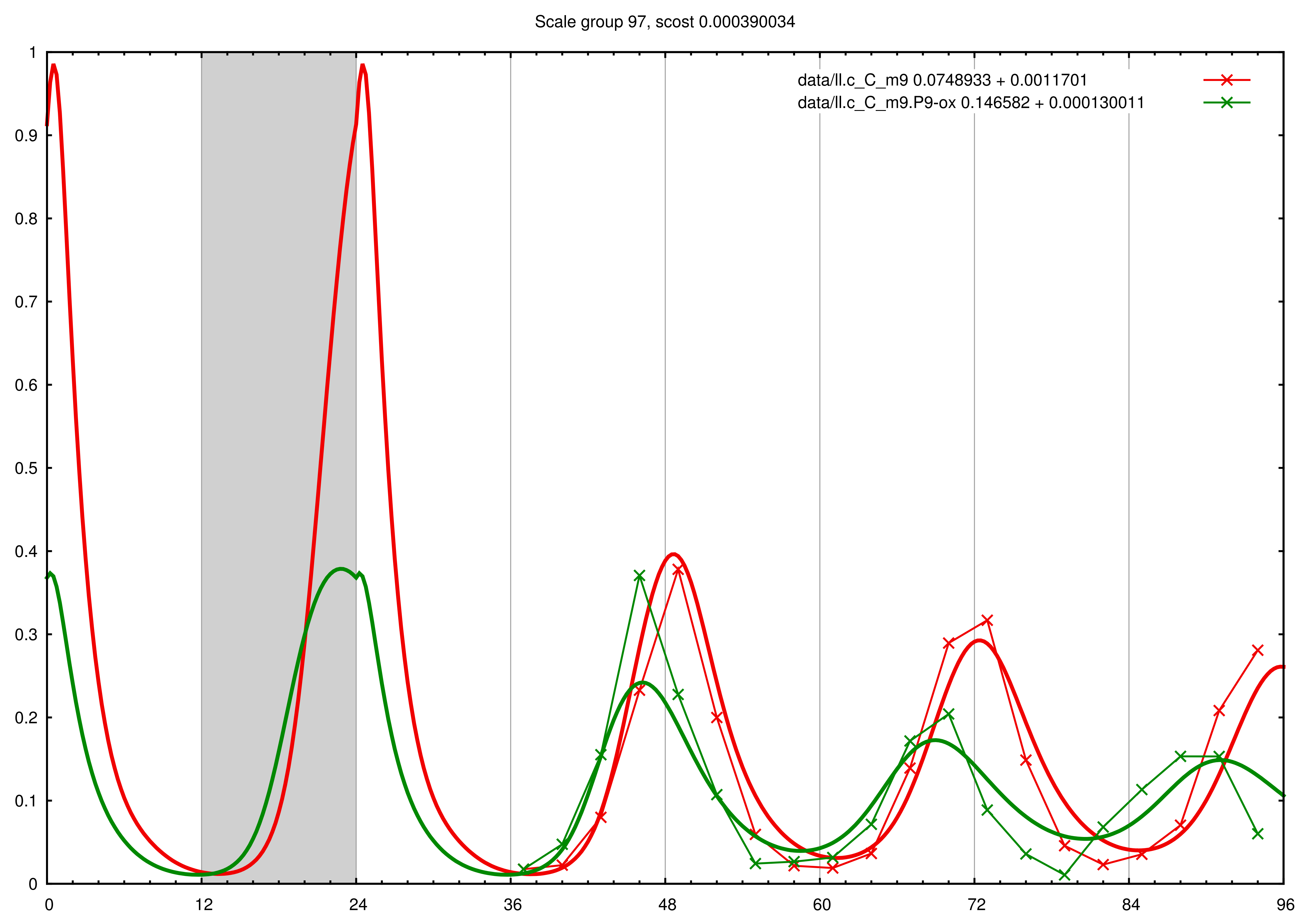

Scale group 97, scost 0.000390034
data/ll.c_C_m9 0.0748933 + 0.0011701
data/ll.c_C_m9.P9-ox 0.146582 + 0.000130011
0
0.1
0.2
0.3
0.4
0.5
0.6
0.7
0.8
0.9
1
0
12
24
36
48
60
72
84
96

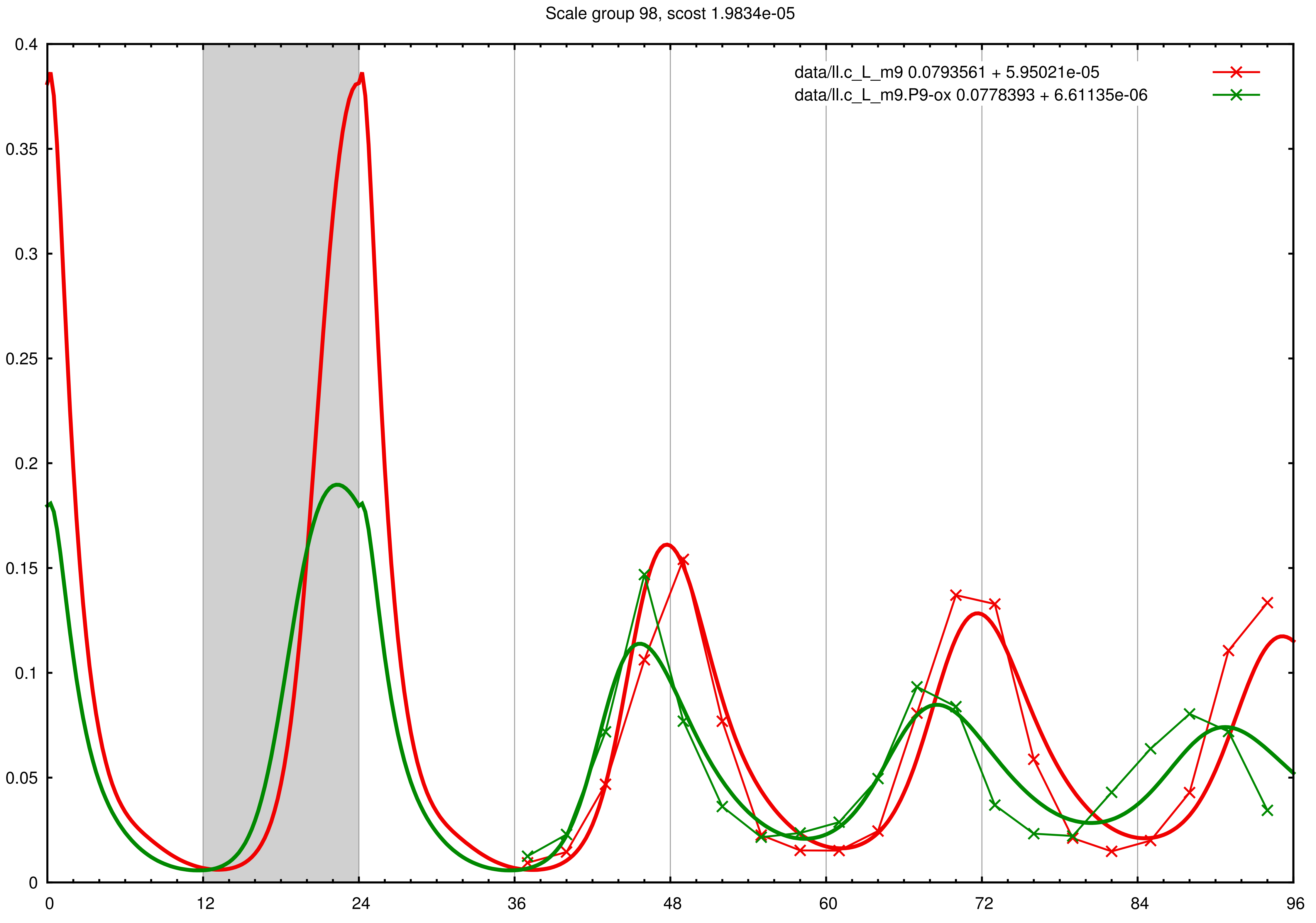
Scale group 98, scost 1.9834e-05
data/ll.c_L_m9 0.0793561 + 5.95021e-05
data/ll.c_L_m9.P9-ox 0.0778393 + 6.61135e-06
0.4
0.35
0.3
0.25
0.2
0.15
0.1
0.05
0
0
12
24
36
48
60
72
84
96

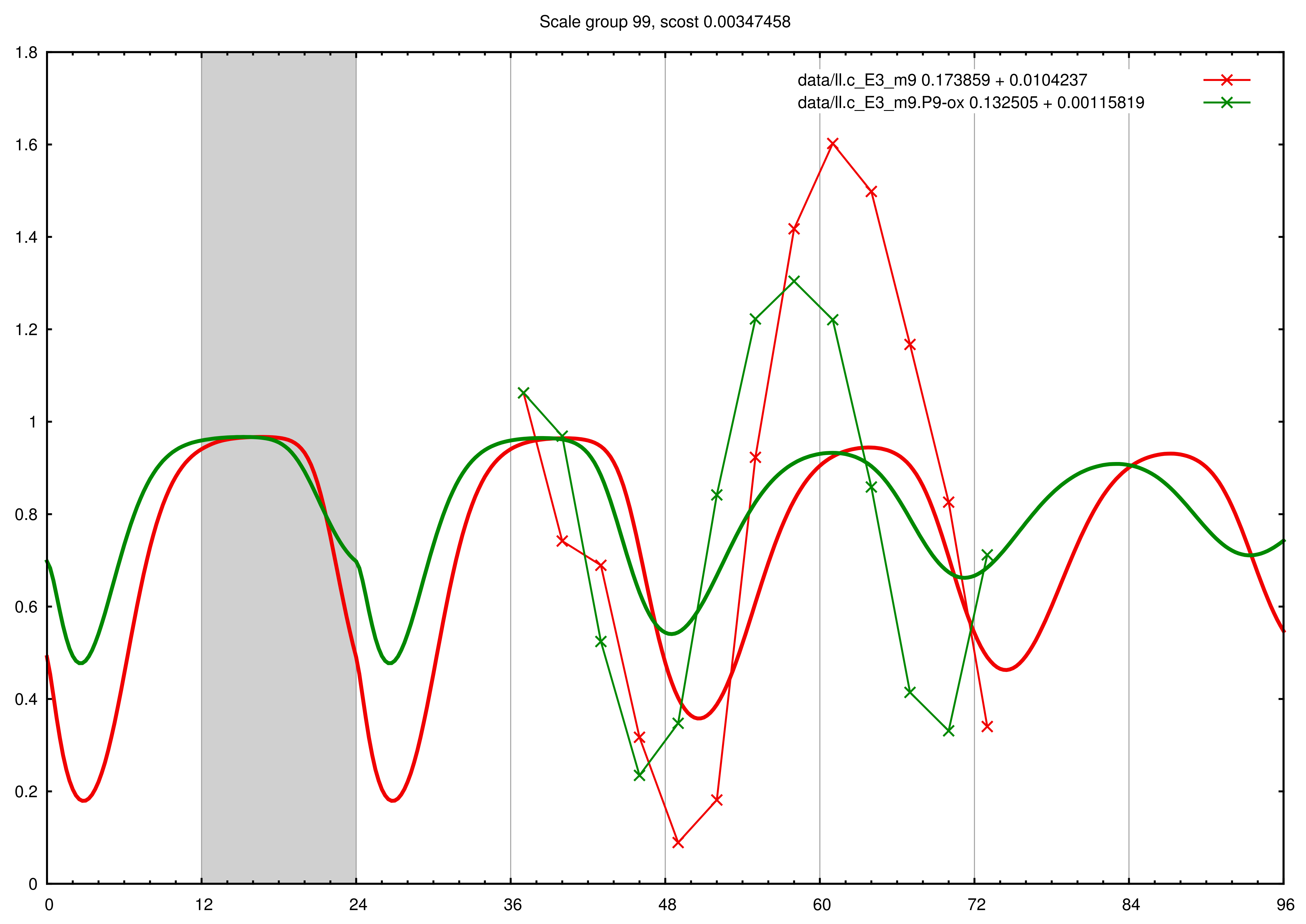

Scale group 99, scost 0.00347458
data/ll.c_E3_m9 0.173859 + 0.0104237
data/ll.c_E3_m9.P9-ox 0.132505 + 0.00115819
1.8
1.6
1.4
1.2
1
0.8
0.6
0.4
0.2
0
0
12
24
36
48
60
72
84
96

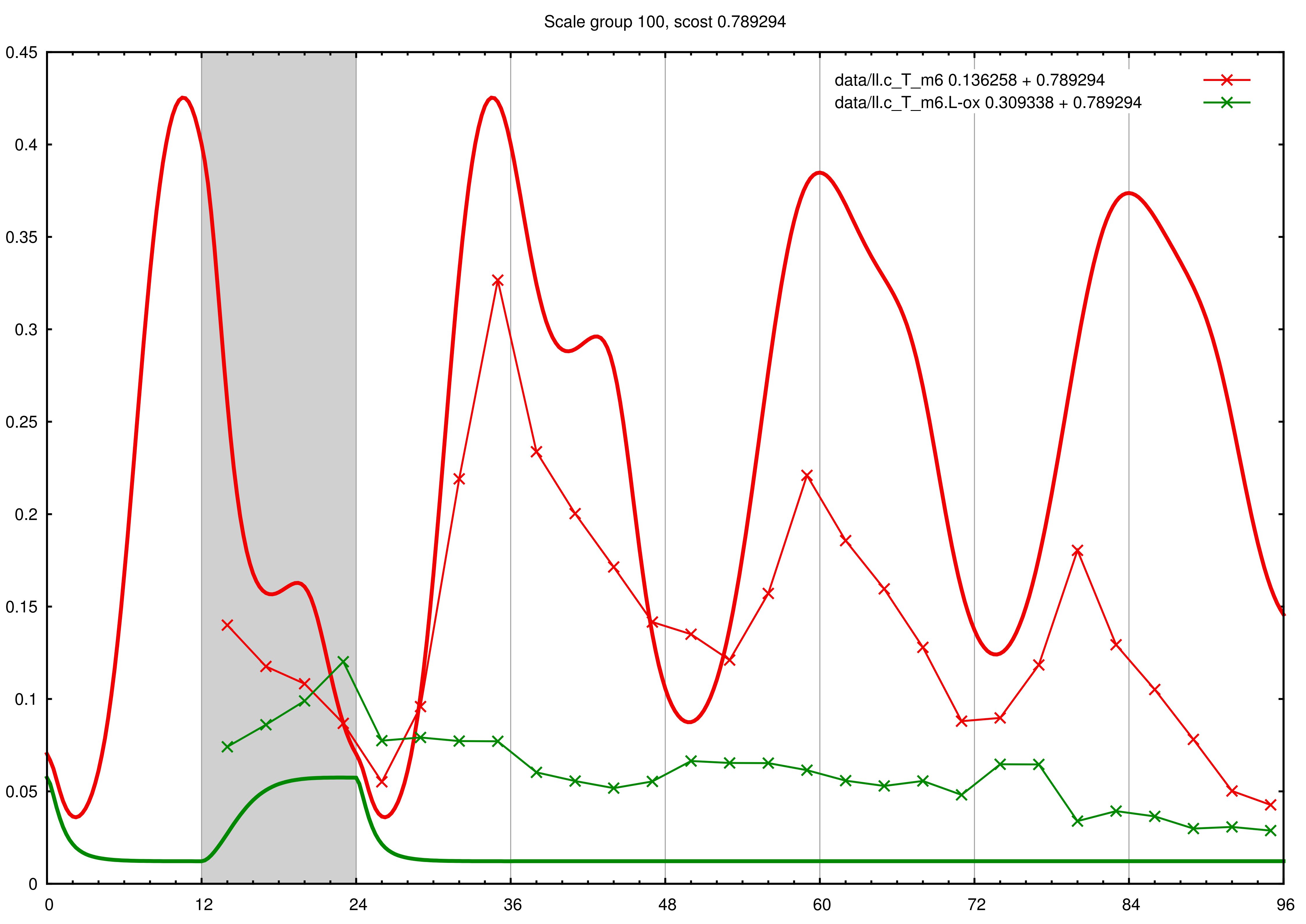

Scale group 100, scost 0.789294
data/ll.c_T_m6 0.136258 + 0.789294
data/ll.c_T_m6.L-ox 0.309338 + 0.789294
0.45
0.4
0.35
0.3
0.25
0.2
0.15
0.1
0.05
0
0
12
24
36
48
60
72
84
96

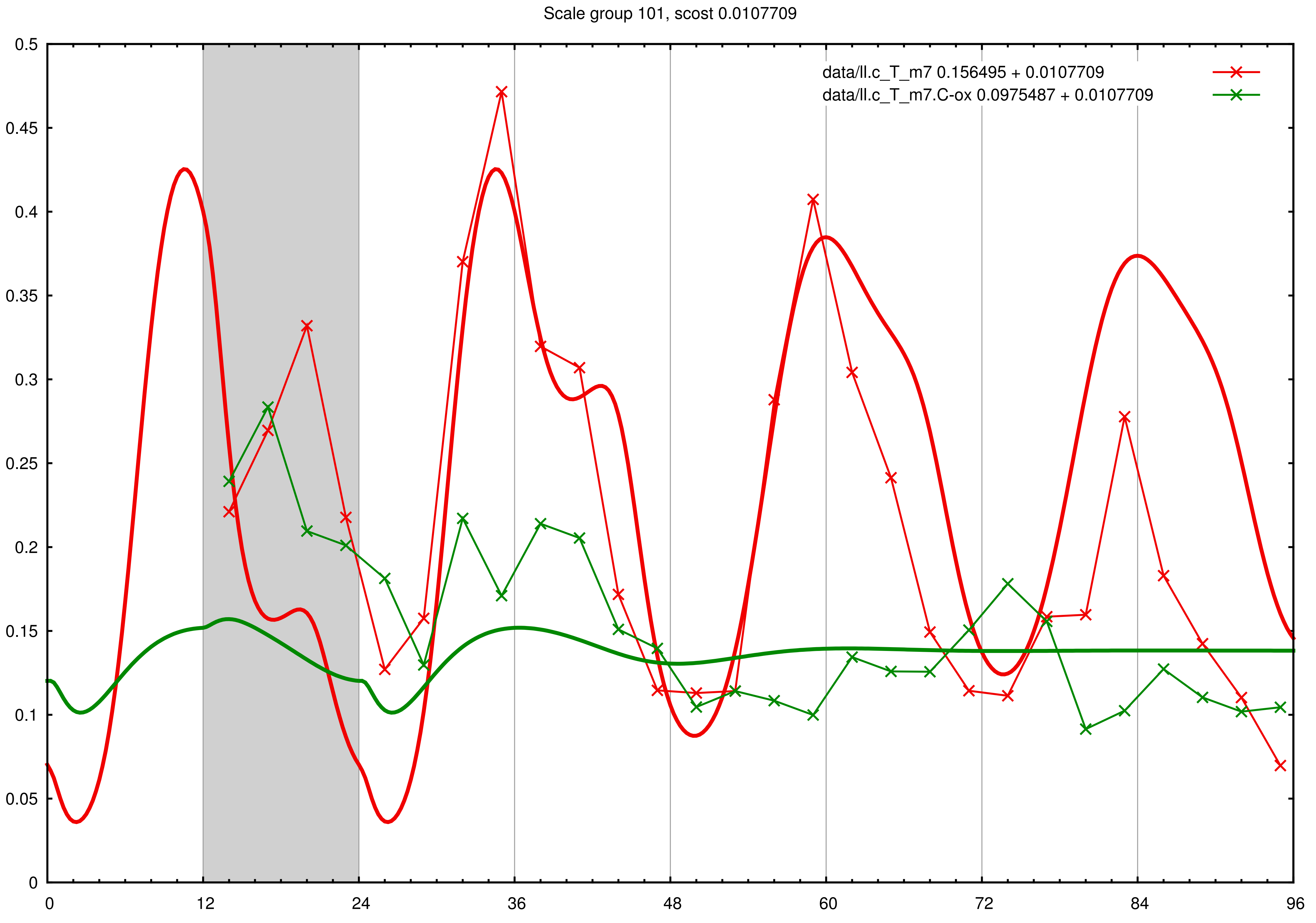

Scale group 101, scost 0.0107709
data/ll.c_T_m7 0.156495 + 0.0107709
data/ll.c_T_m7.C-ox 0.0975487 + 0.0107709
0.5
0.45
0.4
0.35
0.3
0.25
0.2
0.15
0.1
0.05
0
0
12
24
36
48
60
72
84
96

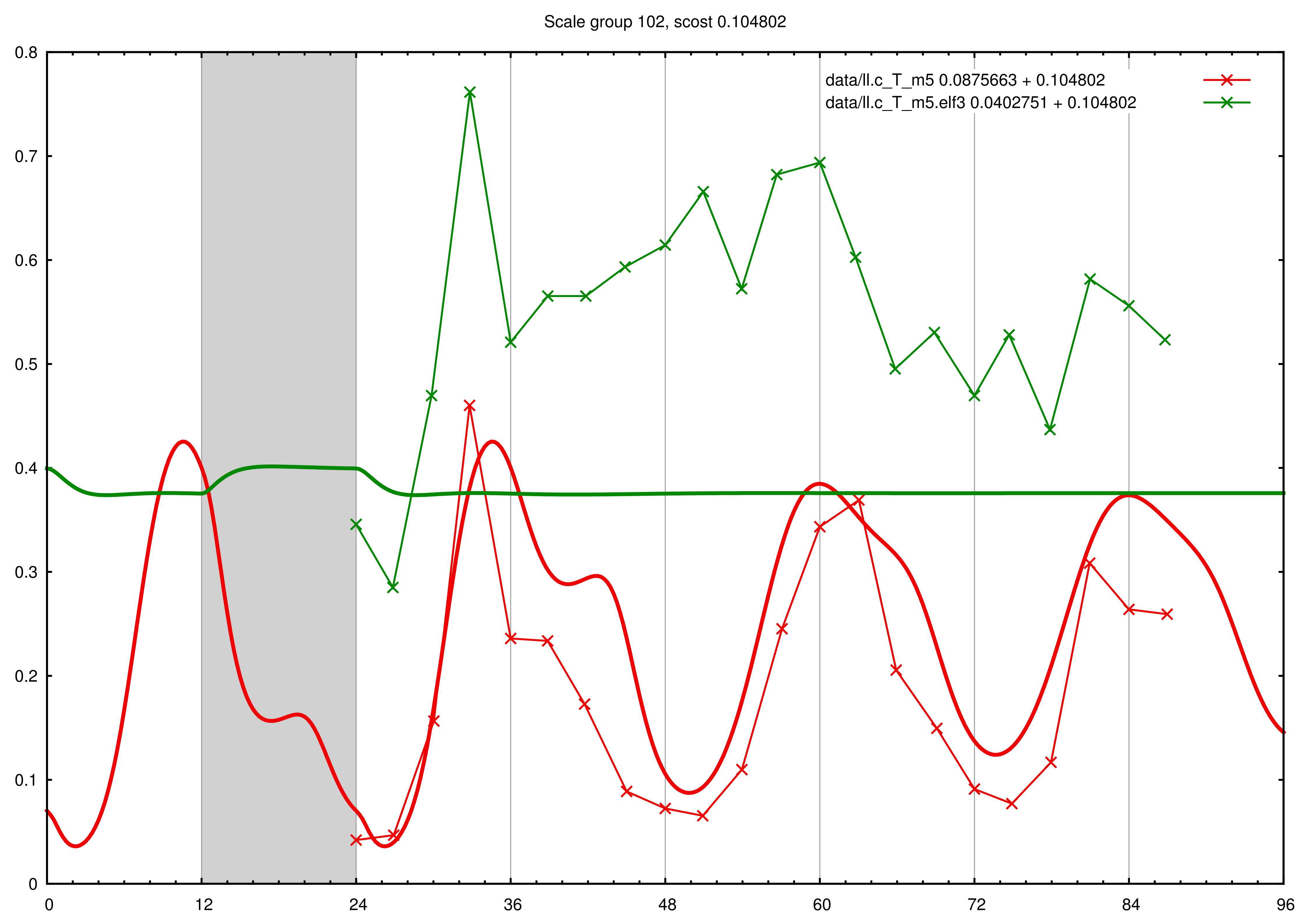
Scale group 102, scost 0.104802
data/ll.c_T_m5 0.0875663 + 0.104802
data/ll.c_T_m5.elf3 0.0402751 + 0.104802
0.8
0.7
0.6
0.5
0.4
0.3
0.2
0.1
0
0
12
24
36
48
60
72
84
96

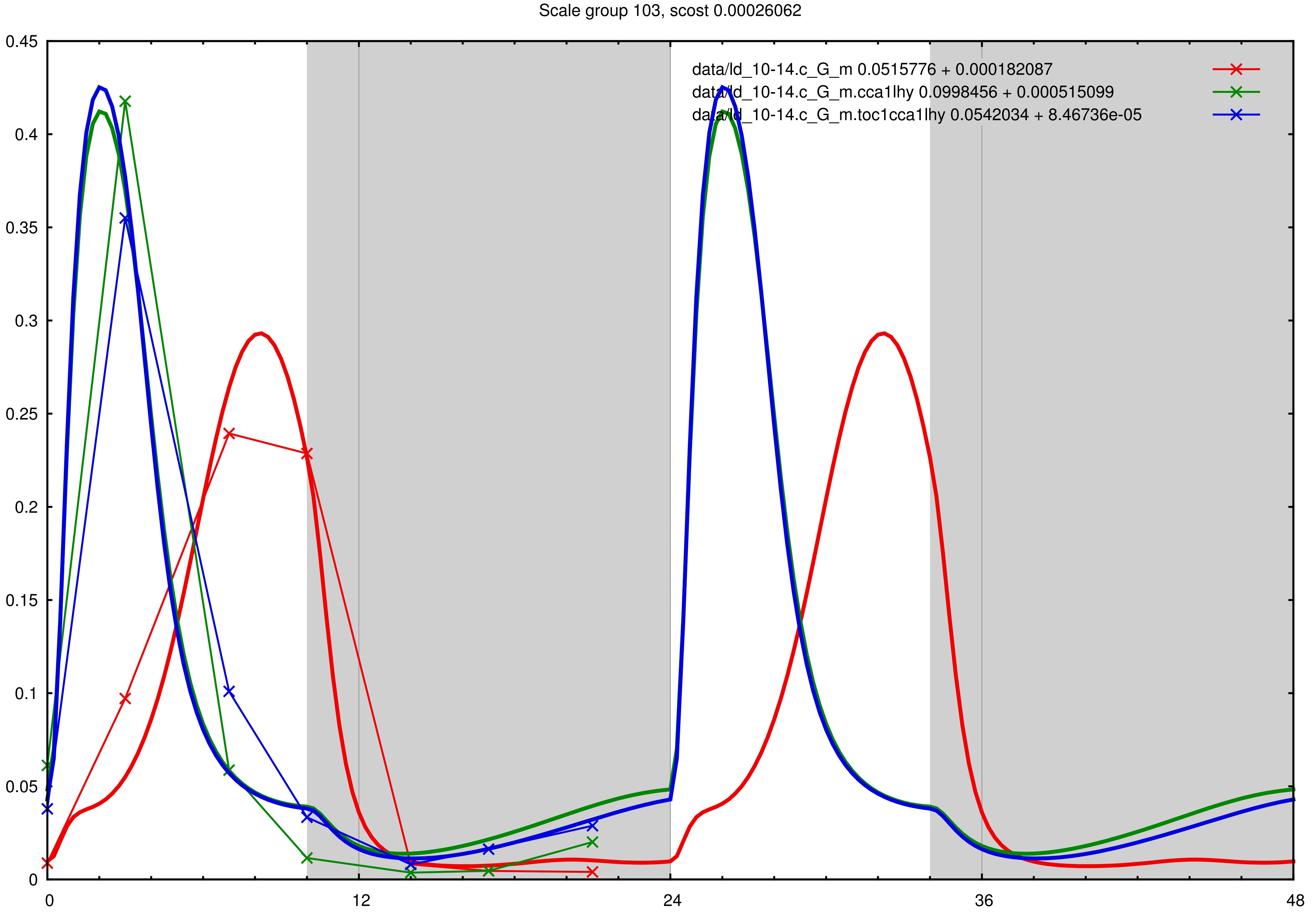

Scale group 103, scost 0.00026062
data/ld_10-14.c_G_m 0.0515776 + 0.000182087
data/ld_10-14.c_G_m.cca1lhy 0.0998456 + 0.000515099
data/ld_10-14.c_G_m.toc1cca1lhy 0.0542034 + 8.46736e-05
0.45
0.4
0.35
0.3
0.25
0.2
0.15
0.1
0.05
0
0
12
24
36
48

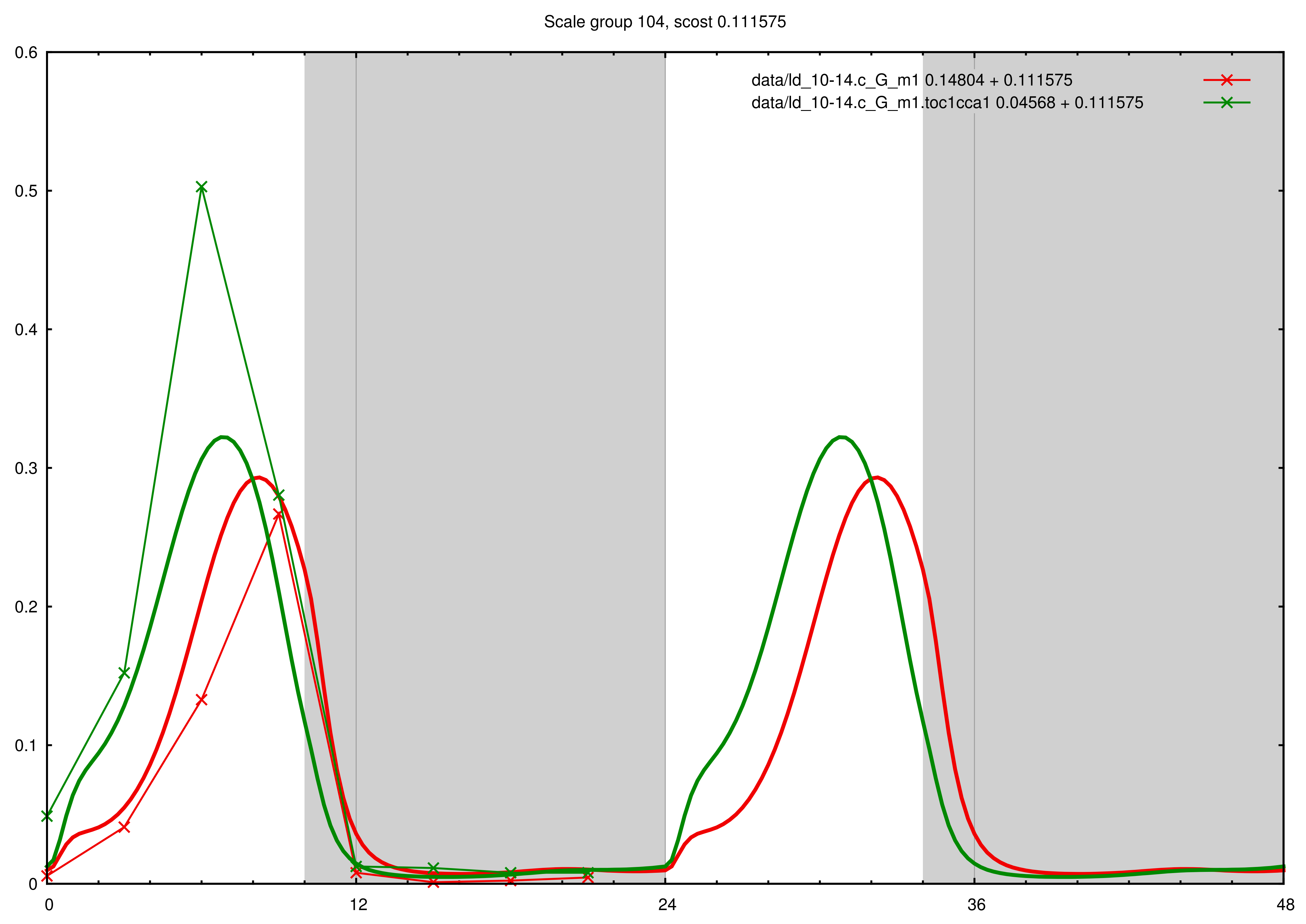

Scale group 104, scost 0.111575
data/ld_10-14.c_G_m1 0.14804 + 0.111575
data/ld_10-14.c_G_m1.toc1cca1 0.04568 + 0.111575
0.6
0.5
0.4
0.3
0.2
0.1
0
0
12
24
36
48

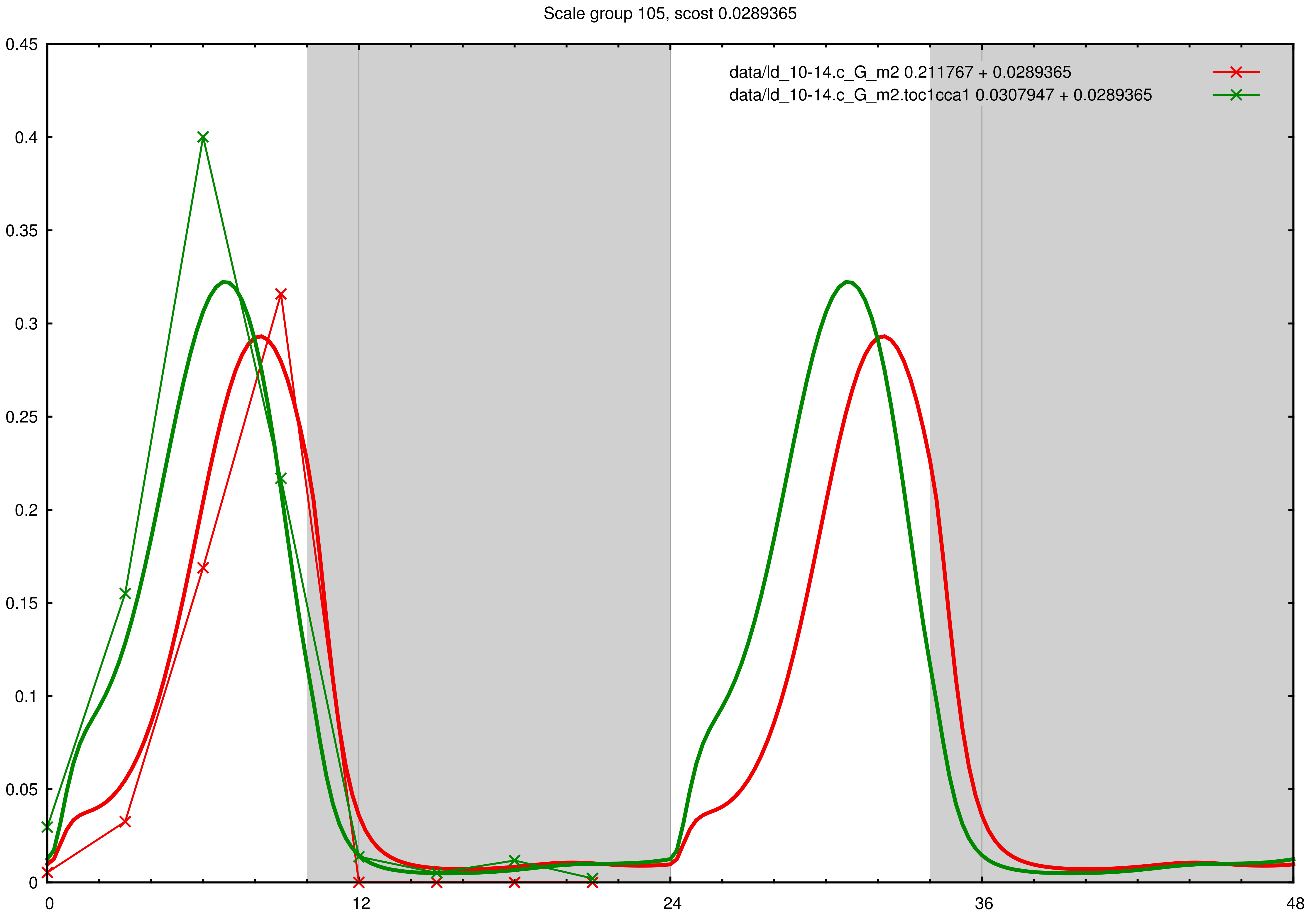
Scale group 105, scost 0.0289365
data/ld_10-14.c_G_m2 0.211767 + 0.0289365
data/ld_10-14.c_G_m2.toc1cca1 0.0307947 + 0.0289365
0.45
0.4
0.35
0.3
0.25
0.2
0.15
0.1
0.05
0
0
12
24
36
48

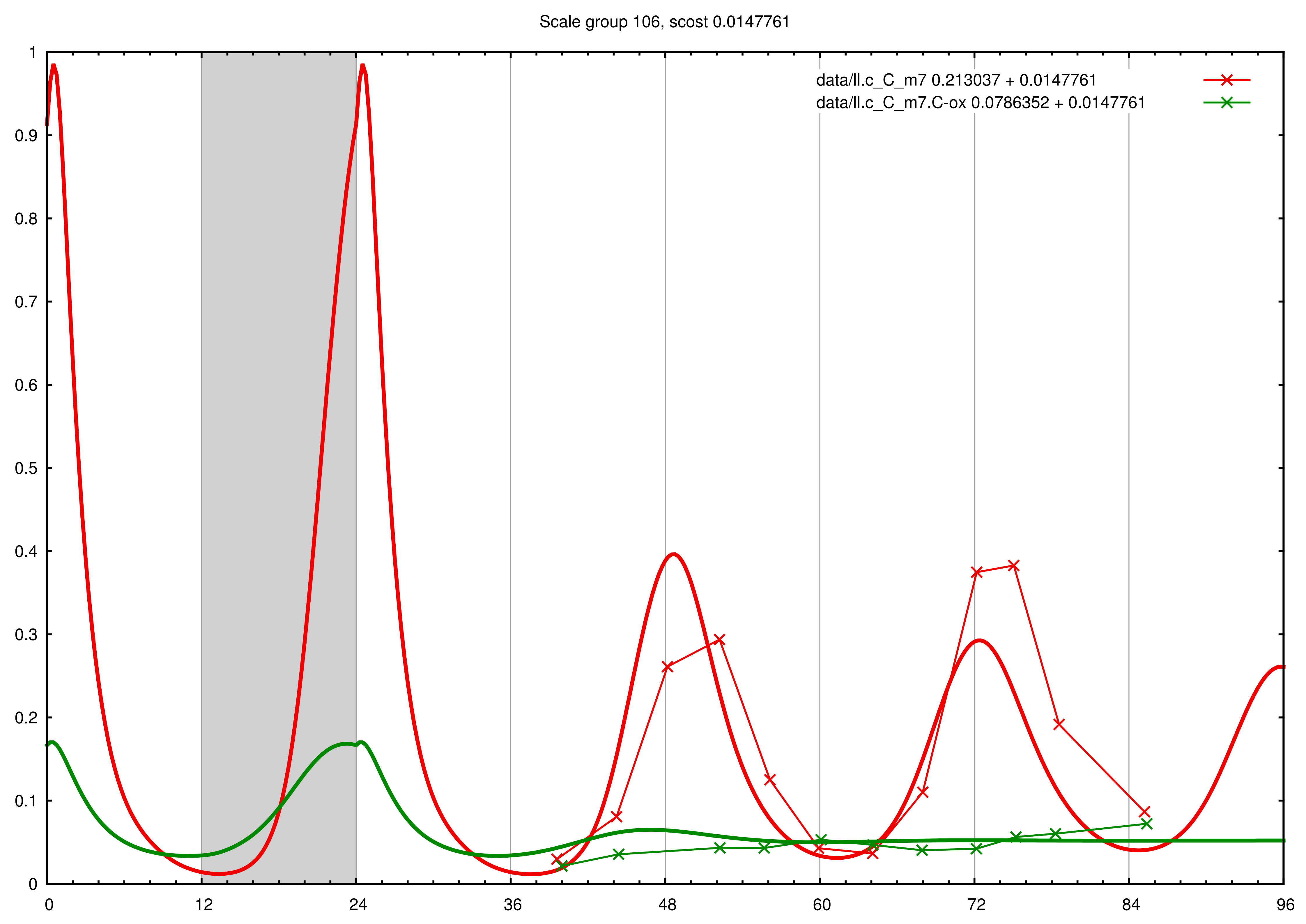

Scale group 106, scost 0.0147761
data/ll.c_C_m7 0.213037 + 0.0147761
data/ll.c_C_m7.C-ox 0.0786352 + 0.0147761
0
0.1
0.2
0.3
0.4
0.5
0.6
0.7
0.8
0.9
1
0
12
24
36
48
60
72
84
96

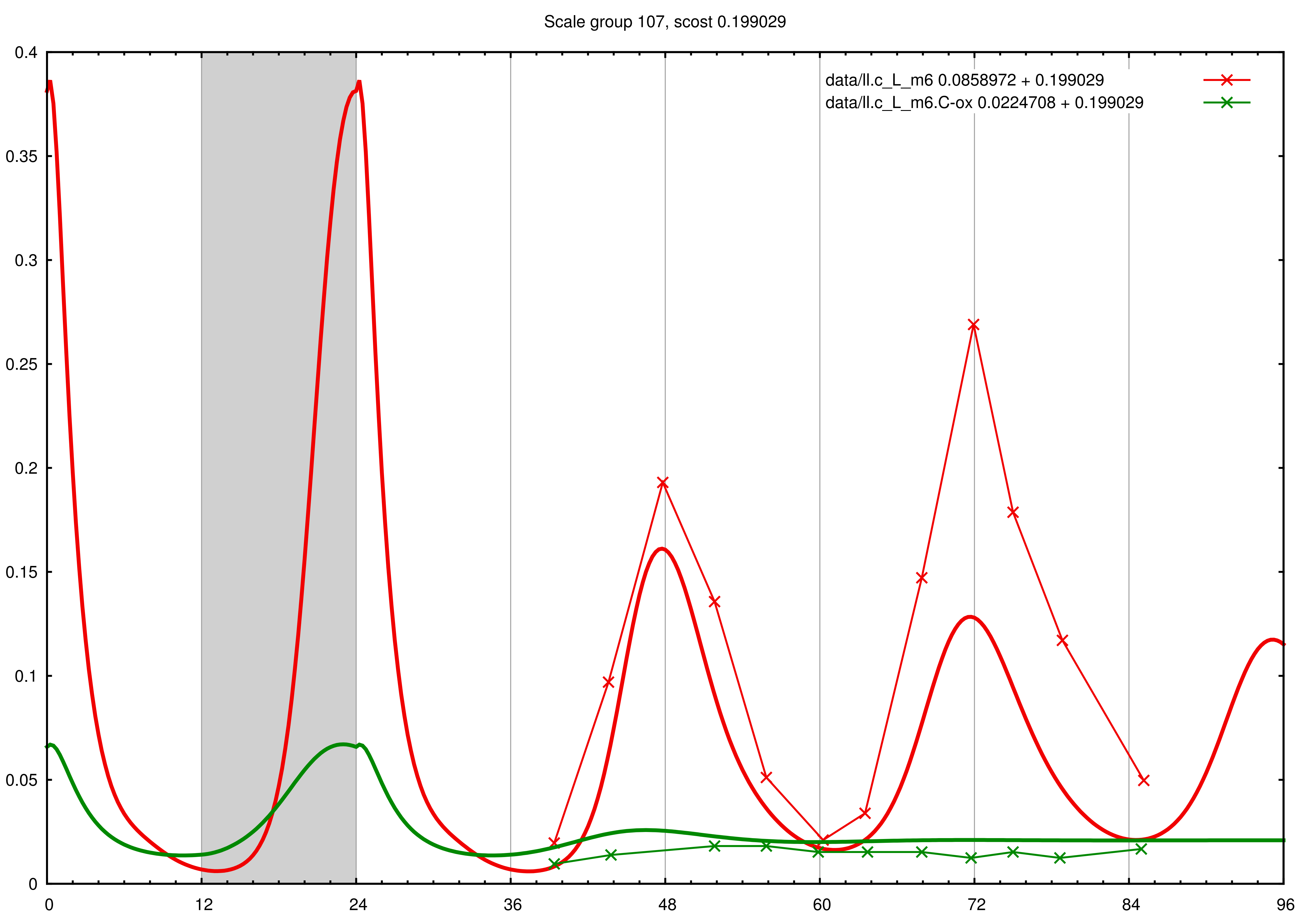
Scale group 107, scost 0.199029
data/ll.c_L_m6 0.0858972 + 0.199029
data/ll.c_L_m6.C-ox 0.0224708 + 0.199029
0.4
0.35
0.3
0.25
0.2
0.15
0.1
0.05
0
0
12
24
36
48
60
72
84
96

Scale group 108, scost 0.136968

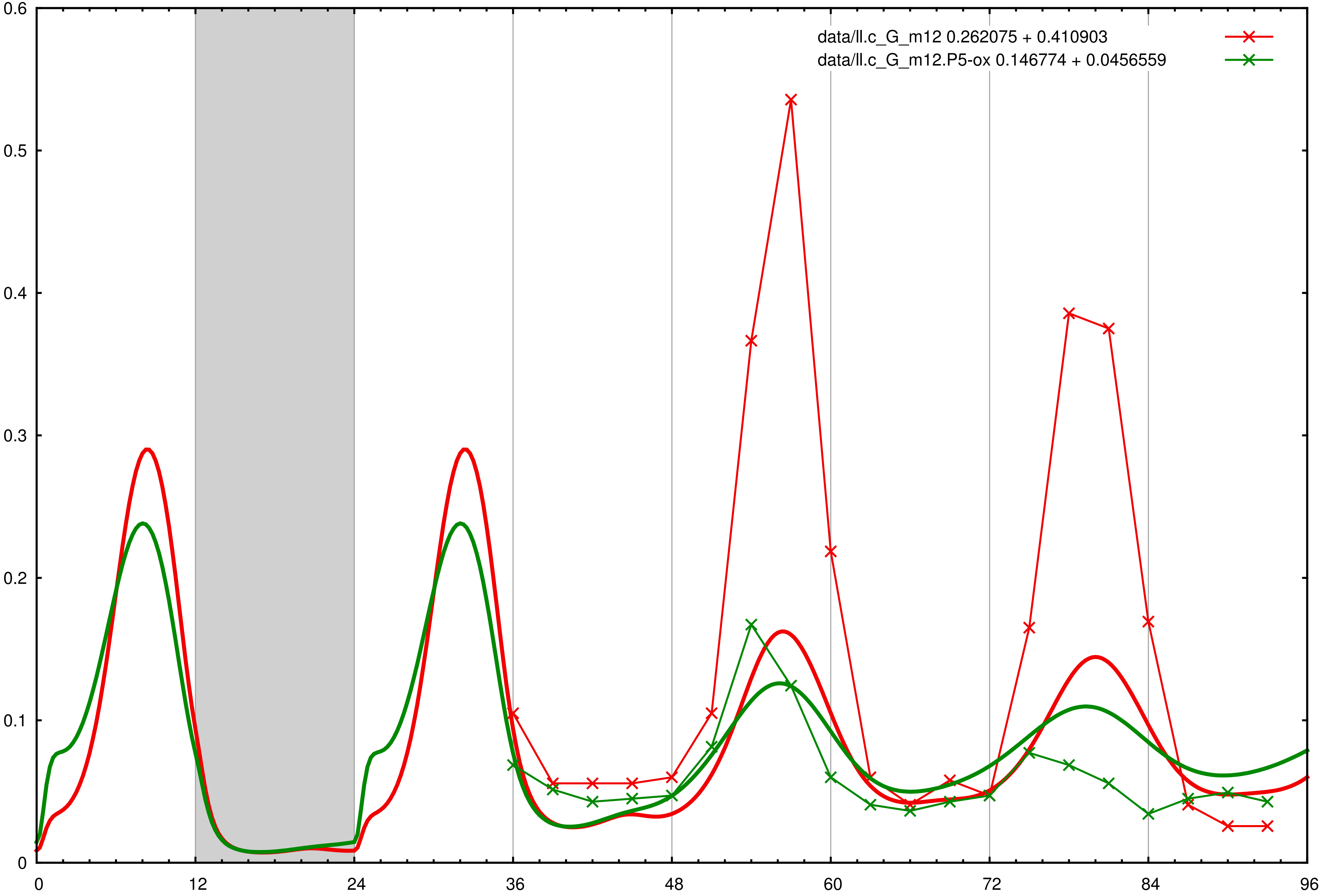

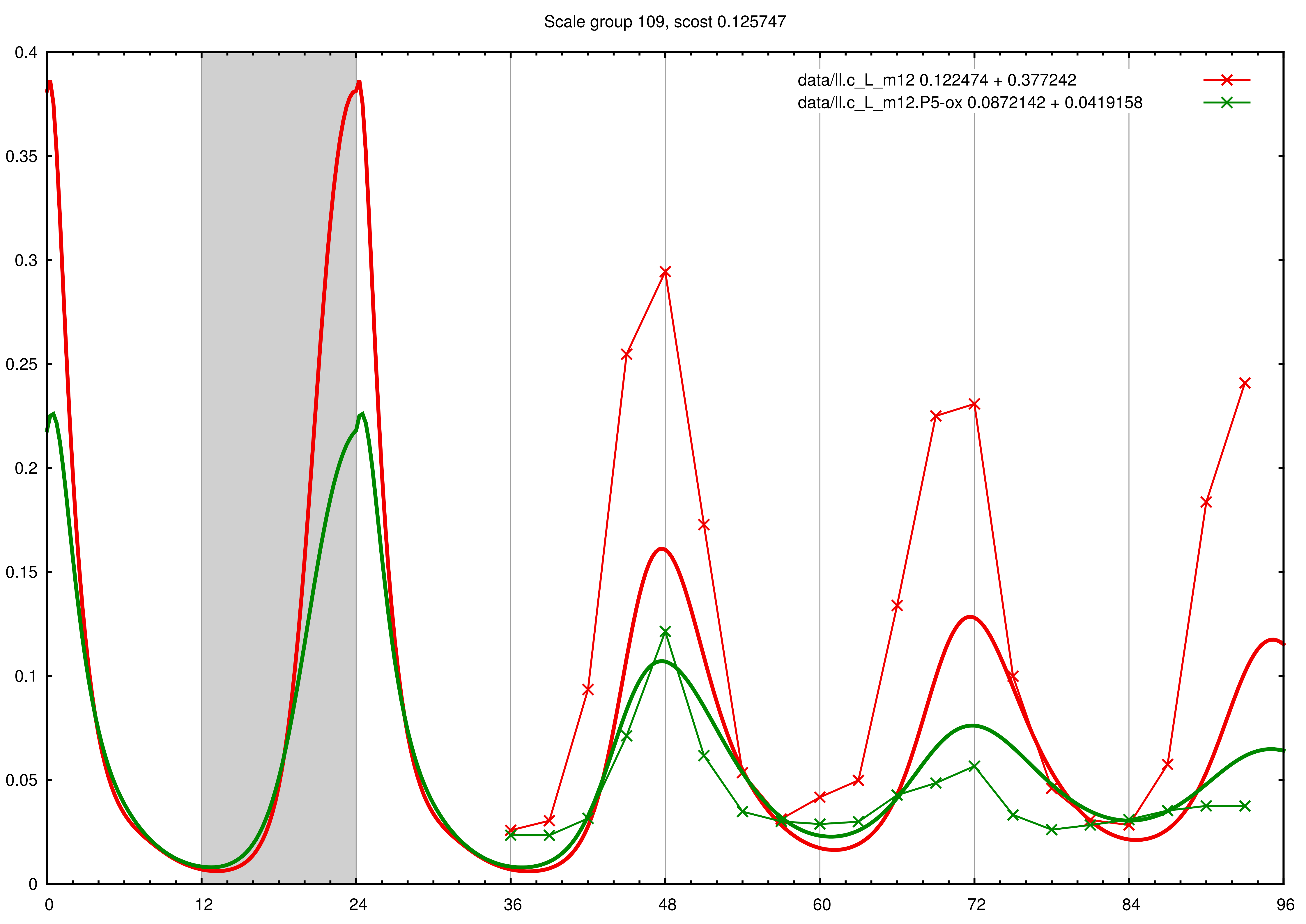
Scale group 109, scost 0.125747
data/ll.c_L_m12 0.122474 + 0.377242
data/ll.c_L_m12.P5-ox 0.0872142 + 0.0419158
0.4
0.35
0.3
0.25
0.2
0.15
0.1
0.05
0
0
12
24
36
48
60
72
84
96

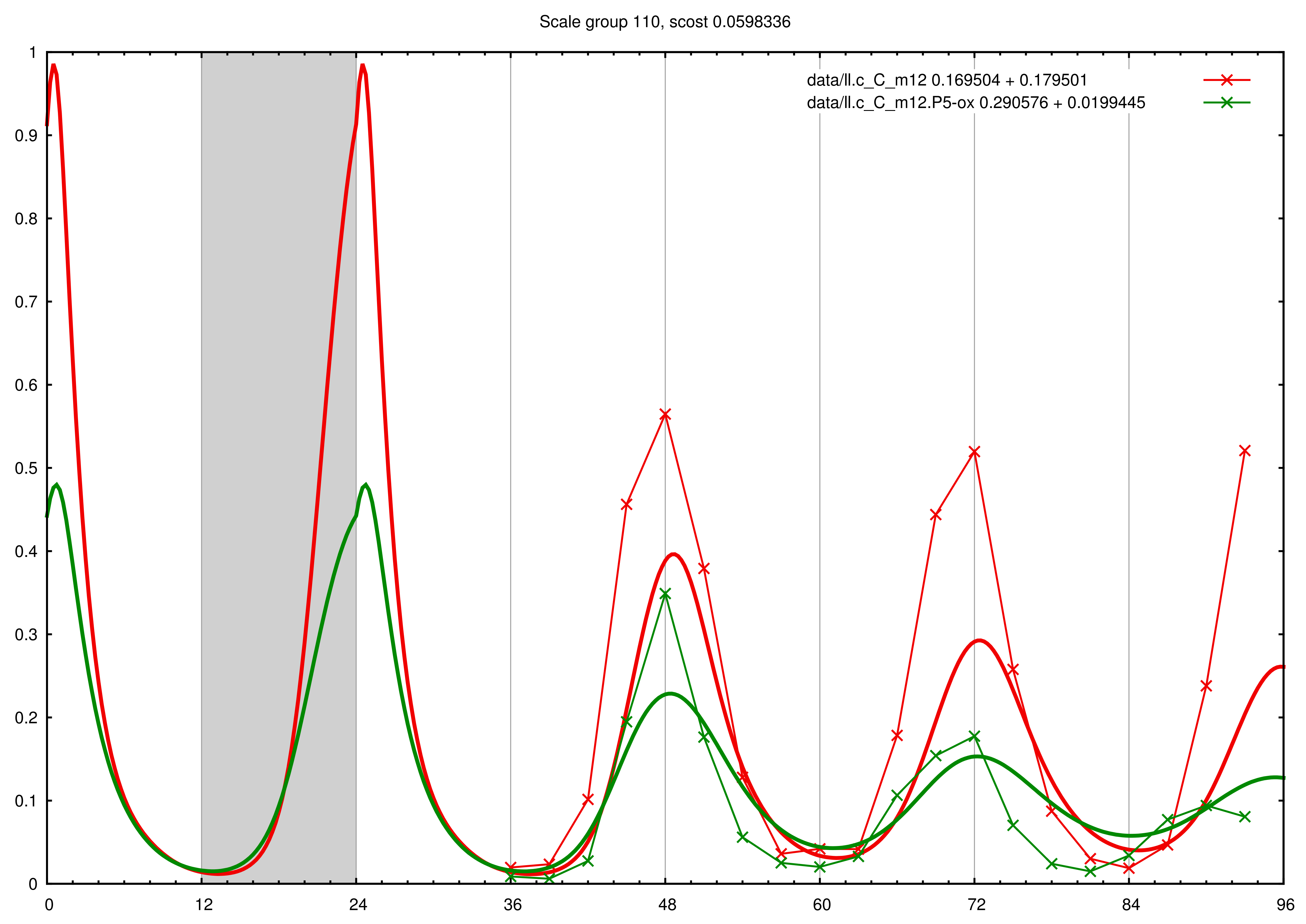

Scale group 110, scost 0.0598336
data/ll.c_C_m12 0.169504 + 0.179501
data/ll.c_C_m12.P5-ox 0.290576 + 0.0199445
0
0.1
0.2
0.3
0.4
0.5
0.6
0.7
0.8
0.9
1
0
12
24
36
48
60
72
84
96

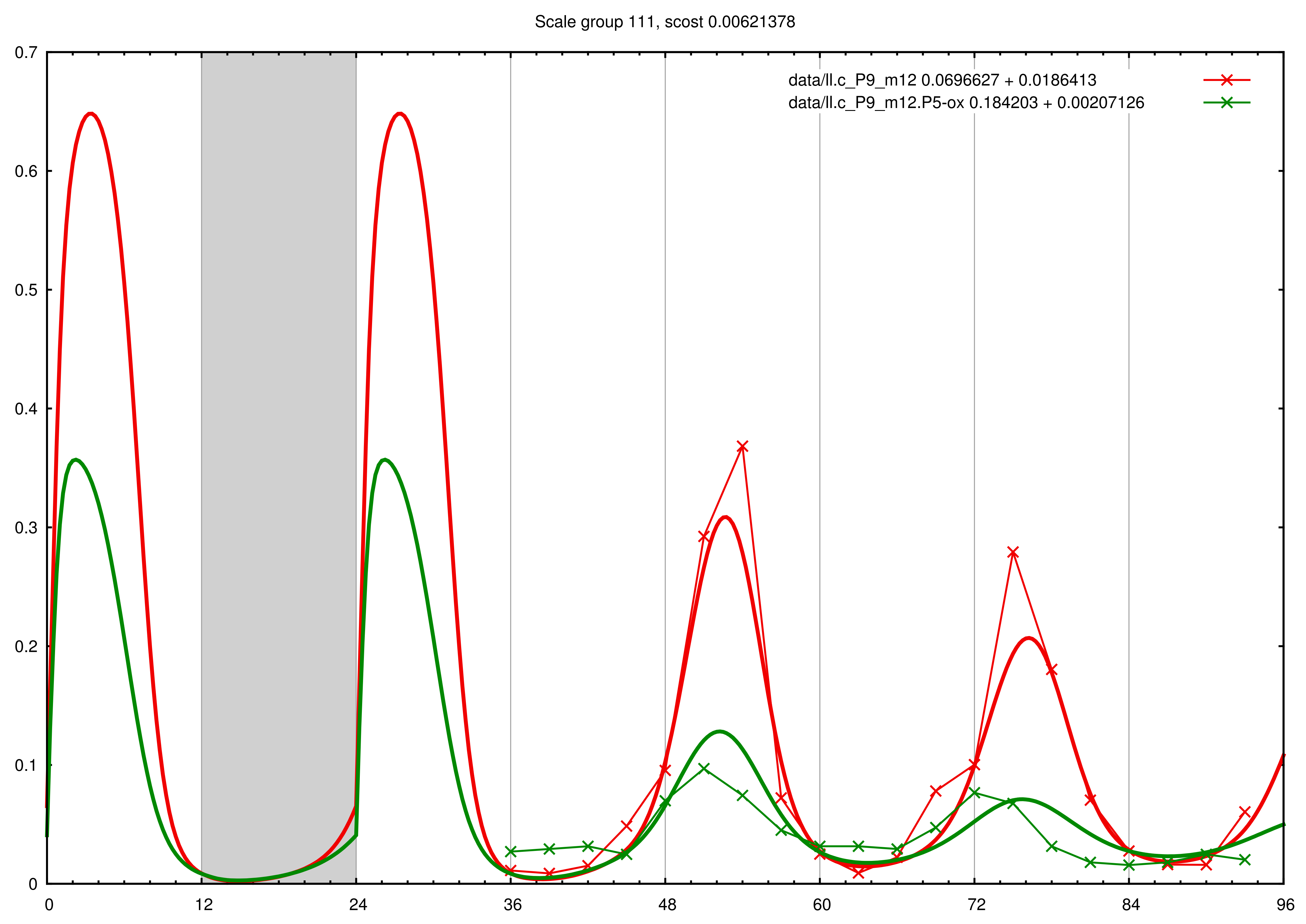

Scale group 111, scost 0.00621378
data/ll.c_P9_m12 0.0696627 + 0.0186413
data/ll.c_P9_m12.P5-ox 0.184203 + 0.00207126
0.7
0.6
0.5
0.4
0.3
0.2
0.1
0
0
12
24
36
48
60
72
84
96

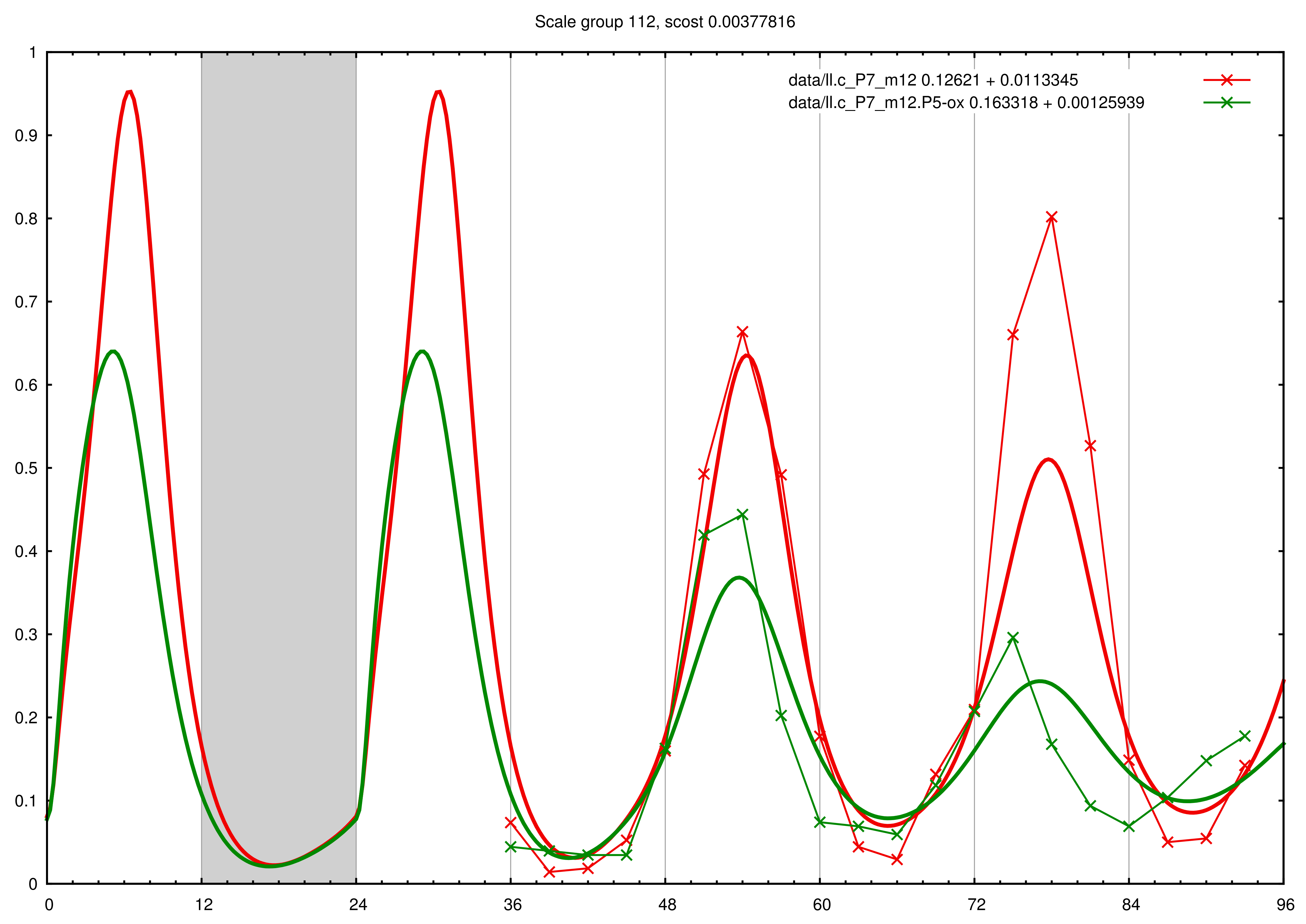

Scale group 112, scost 0.00377816
data/ll.c_P7_m12 0.12621 + 0.0113345
data/ll.c_P7_m12.P5-ox 0.163318 + 0.00125939
0
0.1
0.2
0.3
0.4
0.5
0.6
0.7
0.8
0.9
1
0
12
24
36
48
60
72
84
96

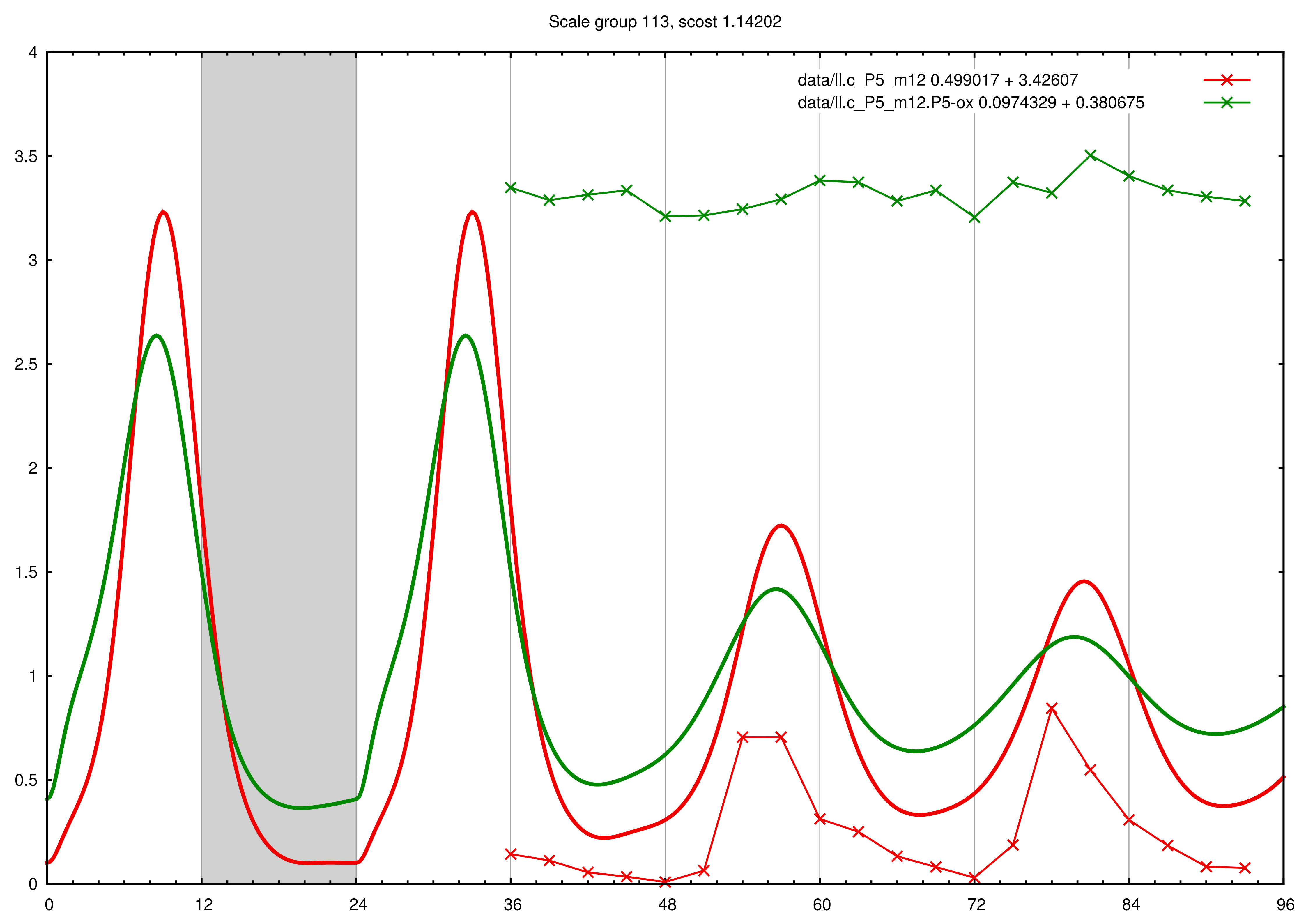
Scale group 113, scost 1.14202
data/ll.c_P5_m12 0.499017 + 3.42607
data/ll.c_P5_m12.P5-ox 0.0974329 + 0.380675
0
0.5
1
1.5
2
2.5
3
3.5
4
0
12
24
36
48
60
72
84
96

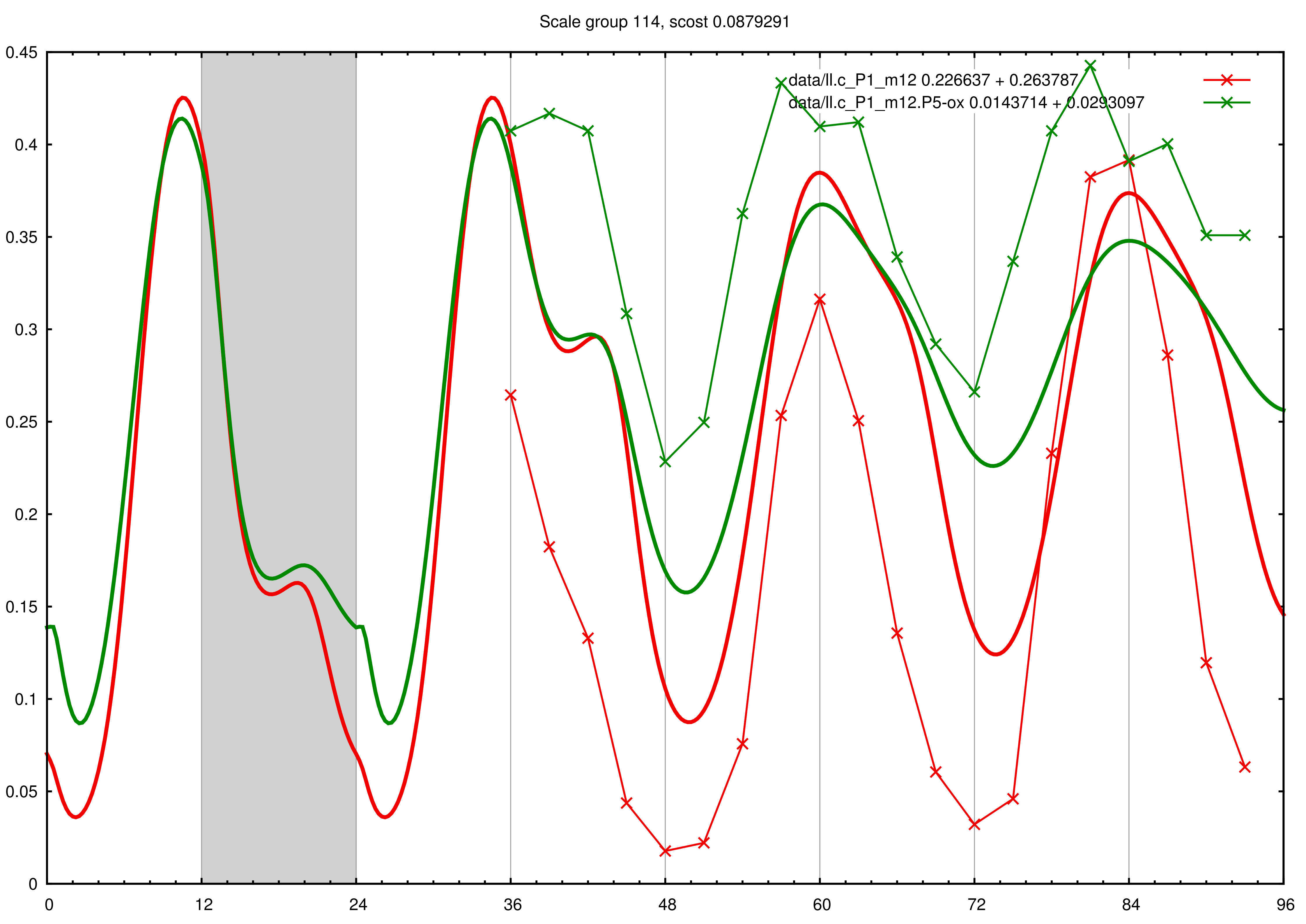

Scale group 114, scost 0.0879291
data/ll.c_P1_m12 0.226637 + 0.263787
data/ll.c_P1_m12.P5-ox 0.0143714 + 0.0293097
0.45
0.4
0.35
0.3
0.25
0.2
0.15
0.1
0.05
0
0
12
24
36
48
60
72
84
96

Scale group 115, scost 0.00564405

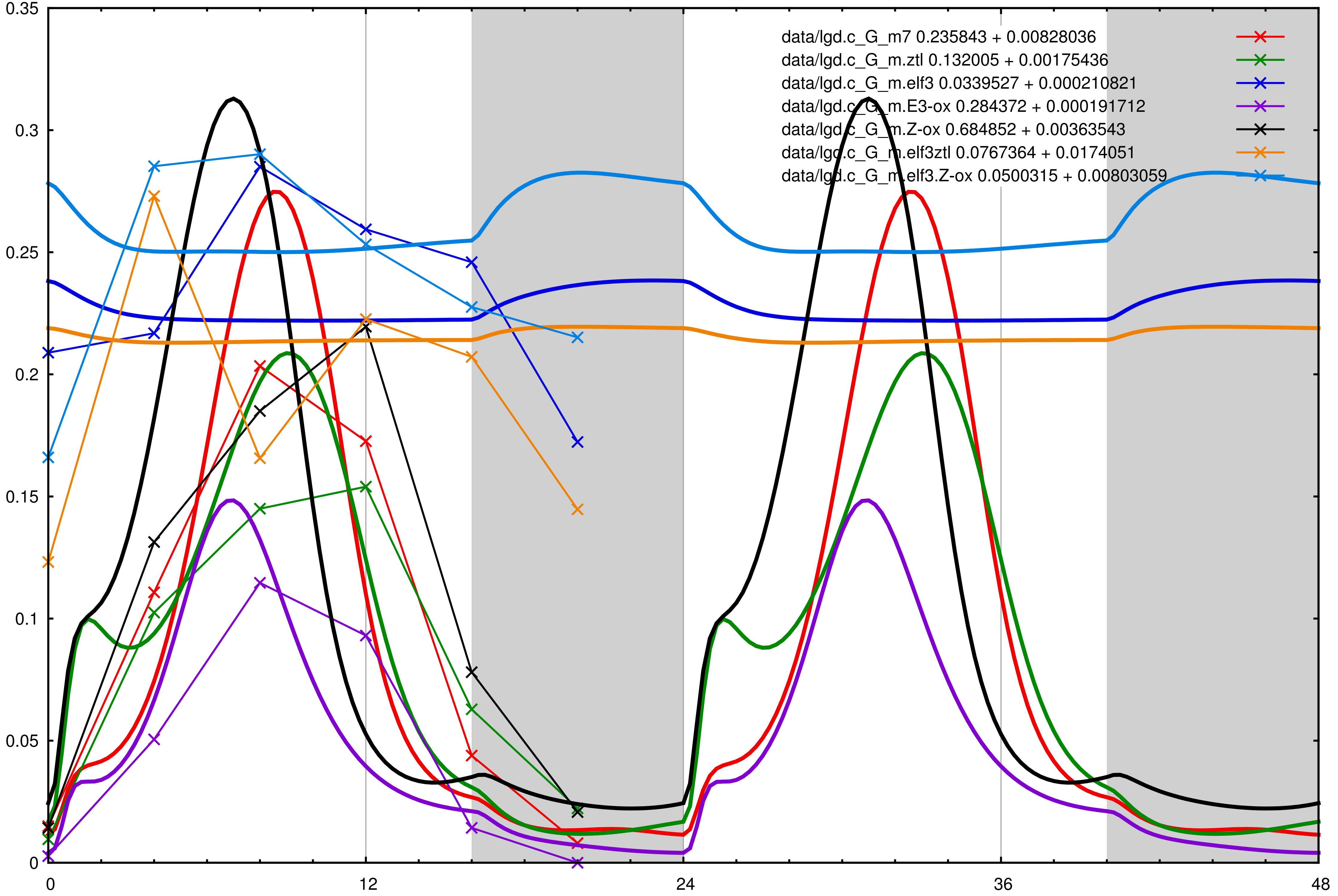

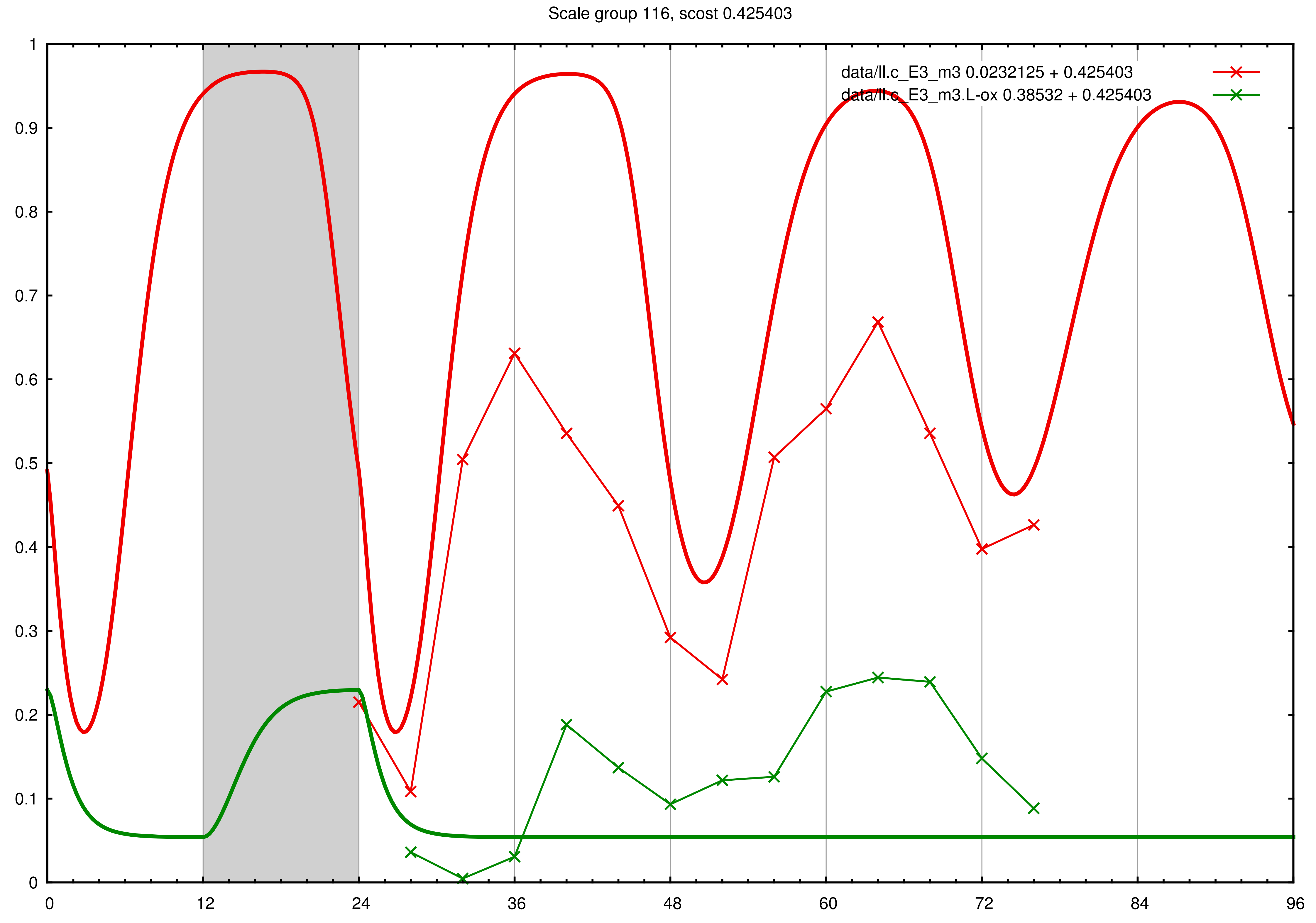

Scale group 116, scost 0.425403
data/ll.c_E3_m3 0.0232125 + 0.425403
data/ll.c_E3_m3.L-ox 0.38532 + 0.425403
1
0.9
0.8
0.7
0.6
0.5
0.4
0.3
0.2
0.1
0
0
12
24
36
48
60
72
84
96

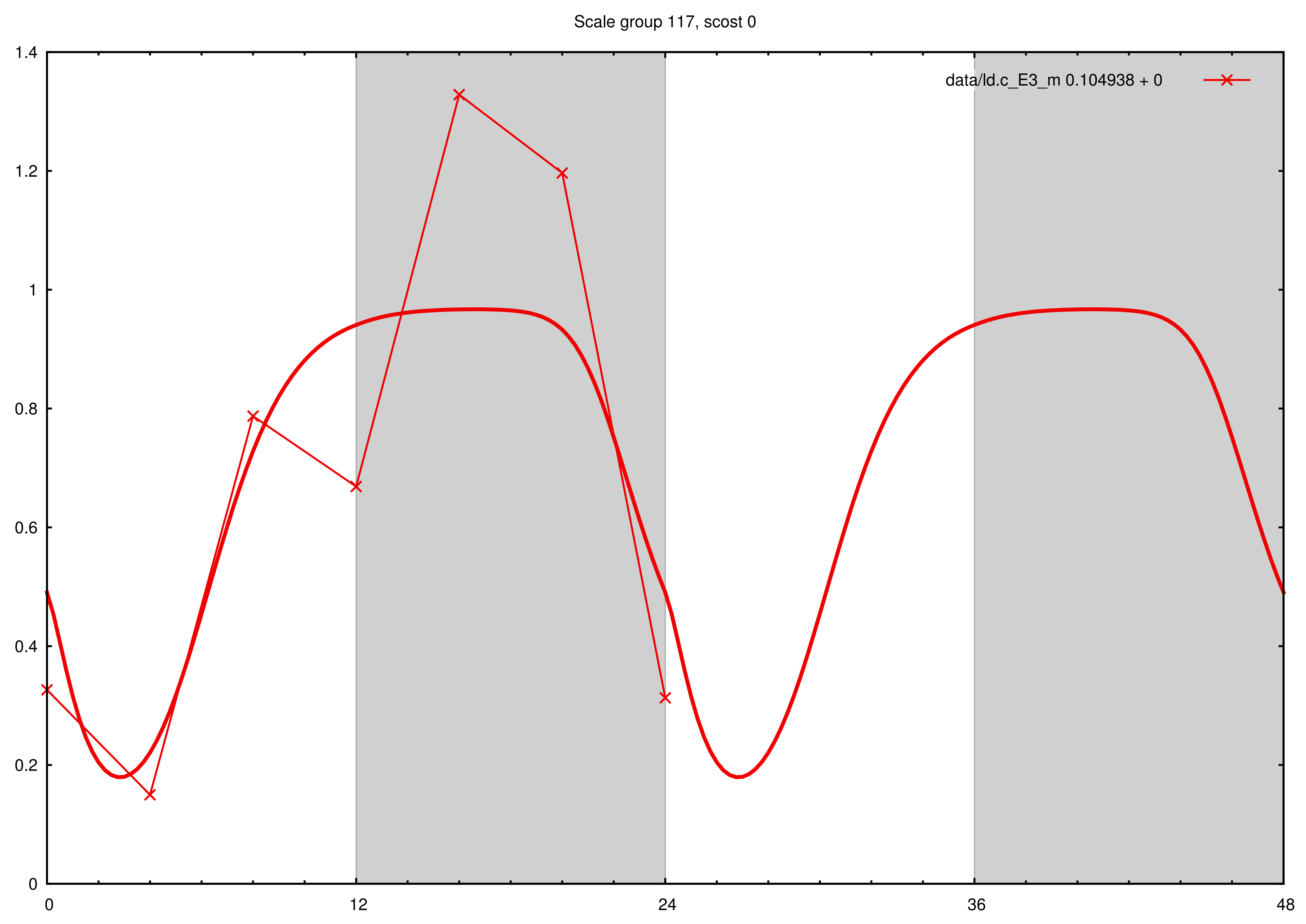
Scale group 117, scost 0
data/ld.c_E3_m 0.104938 + 0
1.4
1.2
1
0.8
0.6
0.4
0.2
0
0
12
24
36
48

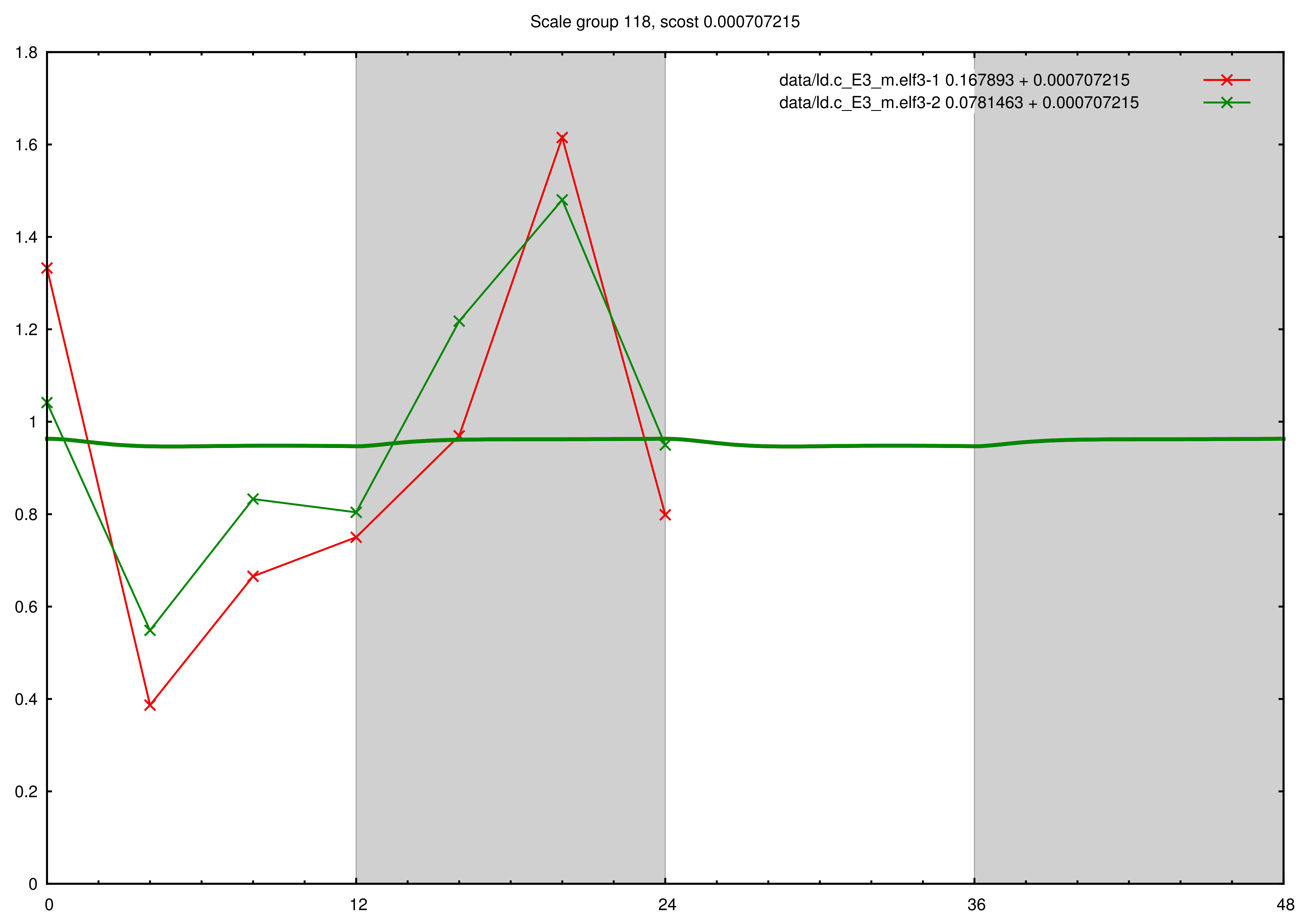

Scale group 118, scost 0.000707215
data/ld.c_E3_m.elf3-1 0.167893 + 0.000707215
data/ld.c_E3_m.elf3-2 0.0781463 + 0.000707215
1.8
1.6
1.4
1.2
1
0.8
0.6
0.4
0.2
0
0
12
24
36
48

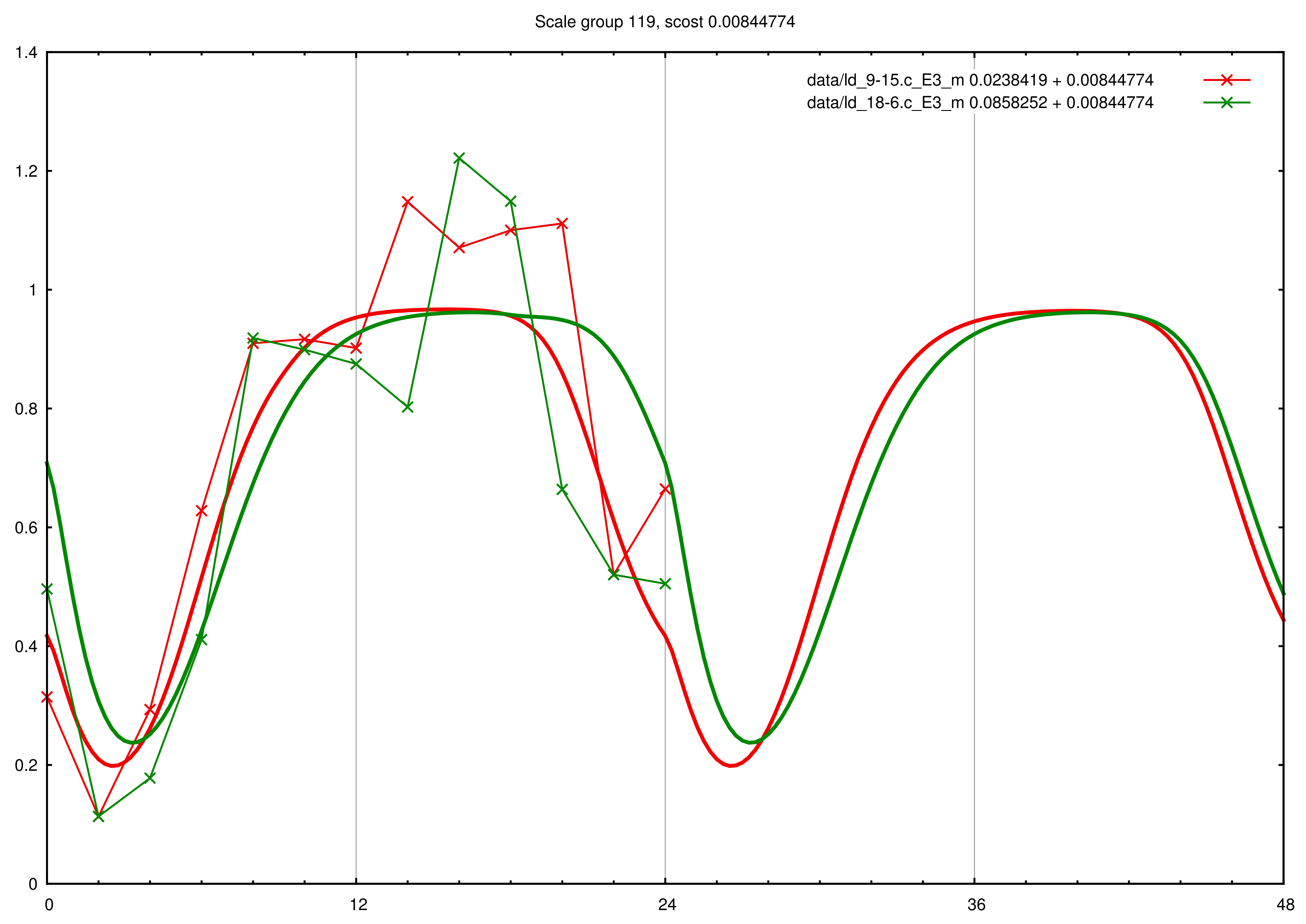

Scale group 119, scost 0.00844774
data/ld_9-15.c_E3_m 0.0238419 + 0.00844774
data/ld_18-6.c_E3_m 0.0858252 + 0.00844774
1.4
1.2
1
0.8
0.6
0.4
0.2
0
0
12
24
36
48

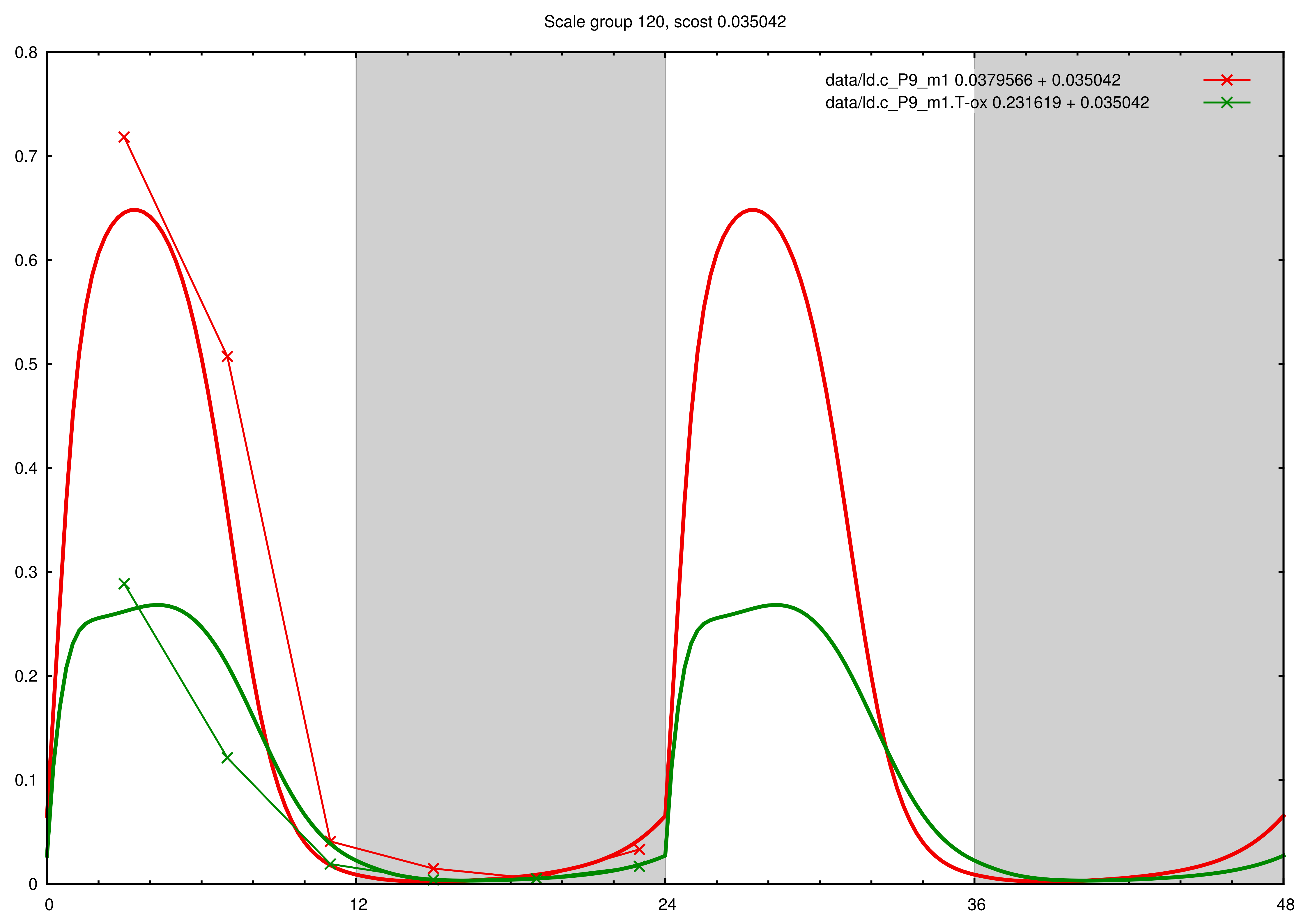

Scale group 120, scost 0.035042
data/ld.c_P9_m1 0.0379566 + 0.035042
data/ld.c_P9_m1.T-ox 0.231619 + 0.035042
0.8
0.7
0.6
0.5
0.4
0.3
0.2
0.1
0
0
12
24
36
48

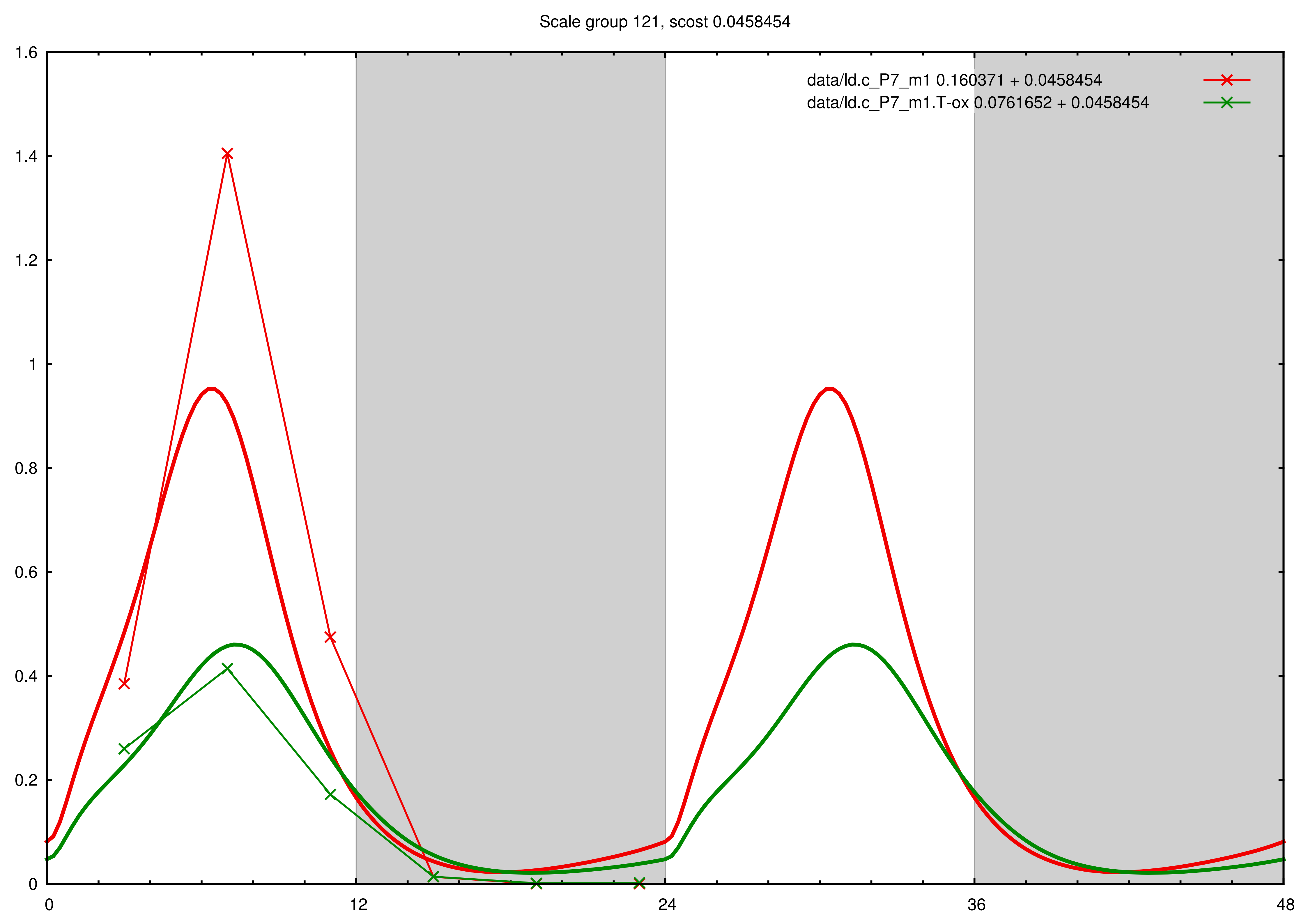

Scale group 121, scost 0.0458454
data/ld.c_P7_m1 0.160371 + 0.0458454
data/ld.c_P7_m1.T-ox 0.0761652 + 0.0458454
1.6
1.4
1.2
1
0.8
0.6
0.4
0.2
0
0
12
24
36
48

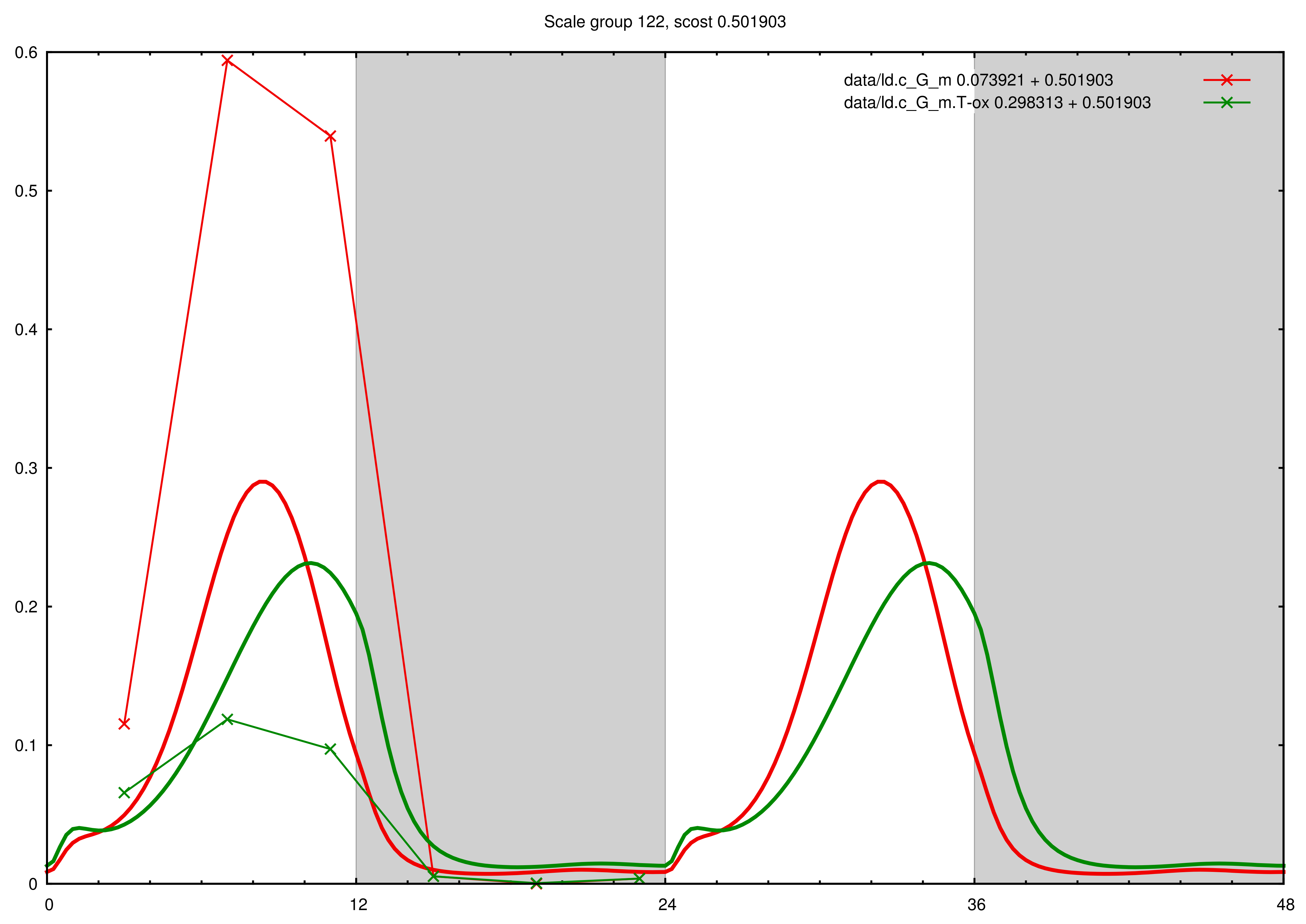

Scale group 122, scost 0.501903
data/ld.c_G_m 0.073921 + 0.501903
data/ld.c_G_m.T-ox 0.298313 + 0.501903
0.6
0.5
0.4
0.3
0.2
0.1
0
0
12
24
36
48

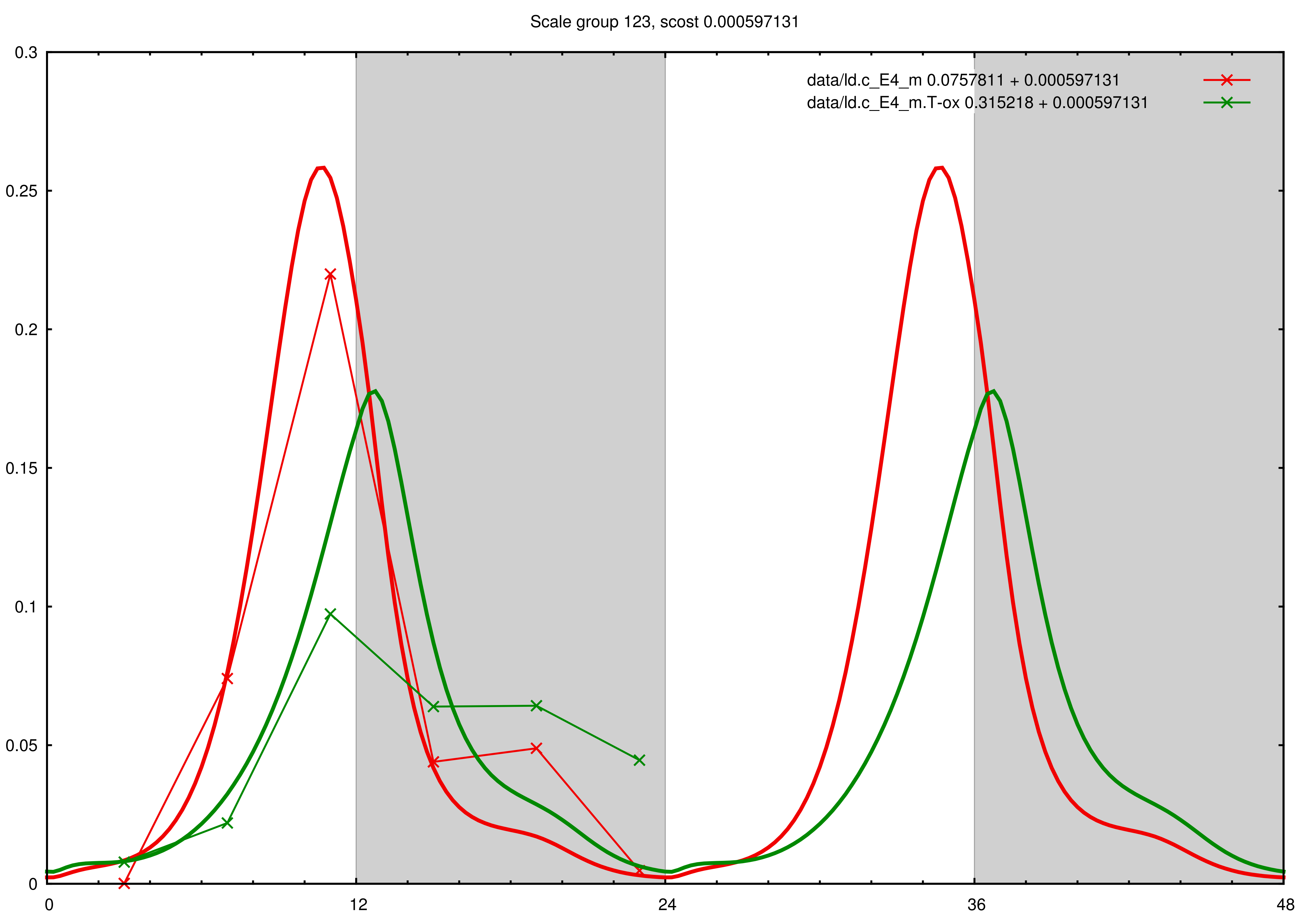

Scale group 123, scost 0.000597131
data/ld.c_E4_m 0.0757811 + 0.000597131
data/ld.c_E4_m.T-ox 0.315218 + 0.000597131
0.3
0.25
0.2
0.15
0.1
0.05
0
0
12
24
36
48

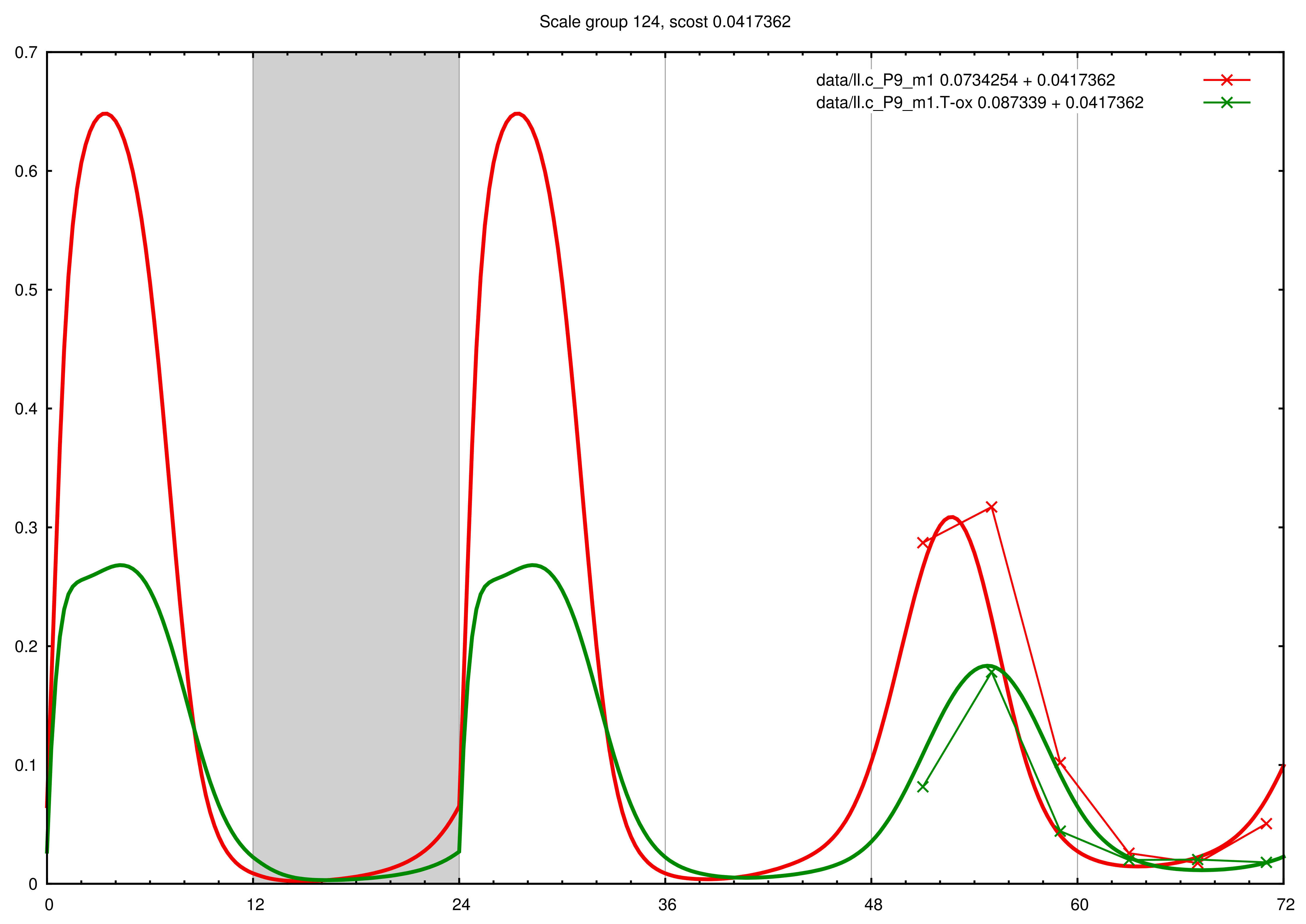

Scale group 124, scost 0.0417362
data/ll.c_P9_m1 0.0734254 + 0.0417362
data/ll.c_P9_m1.T-ox 0.087339 + 0.0417362
0
0.1
0.2
0.3
0.4
0.5
0.6
0.7
0
12
24
36
48
60
72

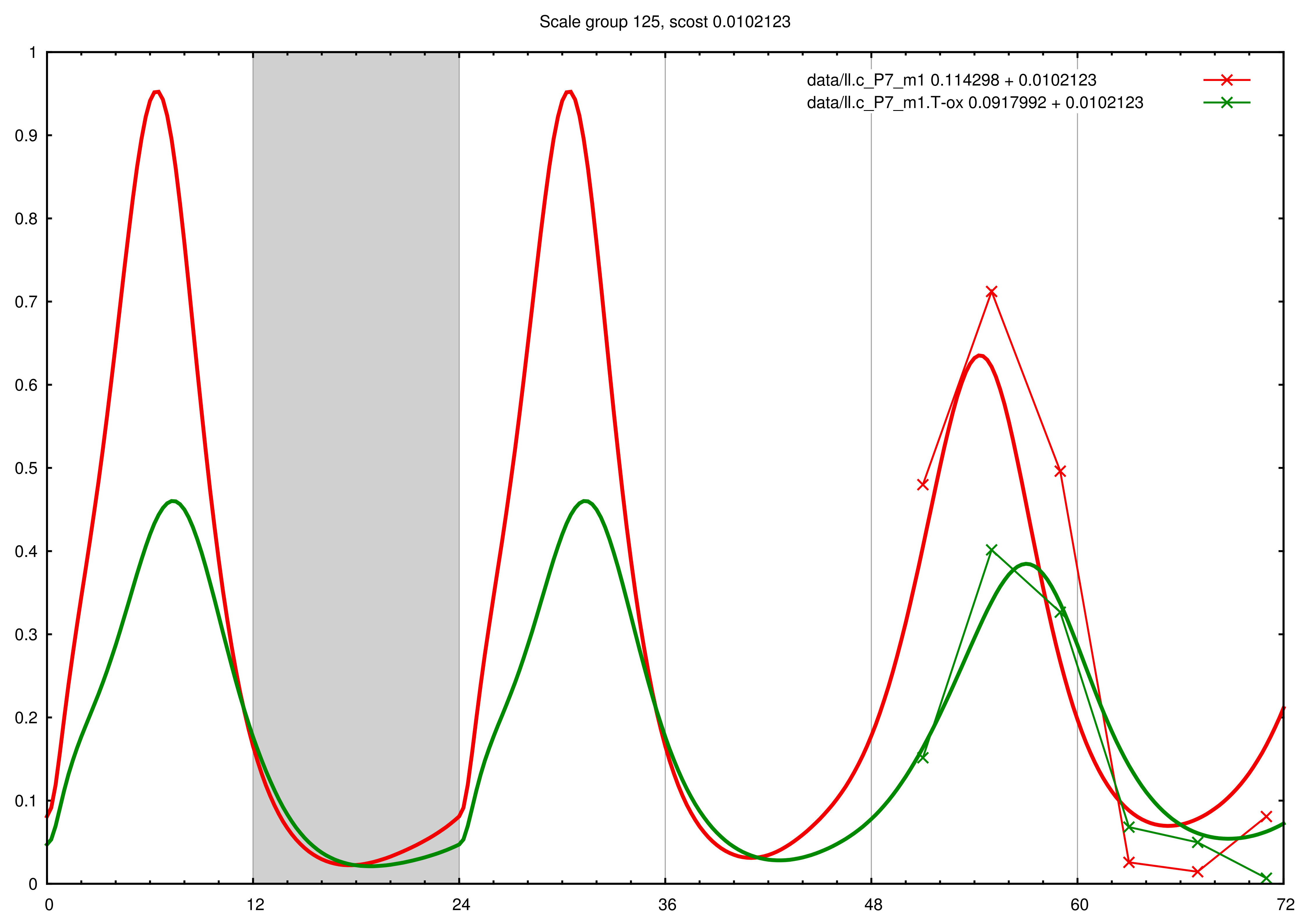

Scale group 125, scost 0.0102123
data/ll.c_P7_m1 0.114298 + 0.0102123
data/ll.c_P7_m1.T-ox 0.0917992 + 0.0102123
0
0.1
0.2
0.3
0.4
0.5
0.6
0.7
0.8
0.9
1
0
12
24
36
48
60
72

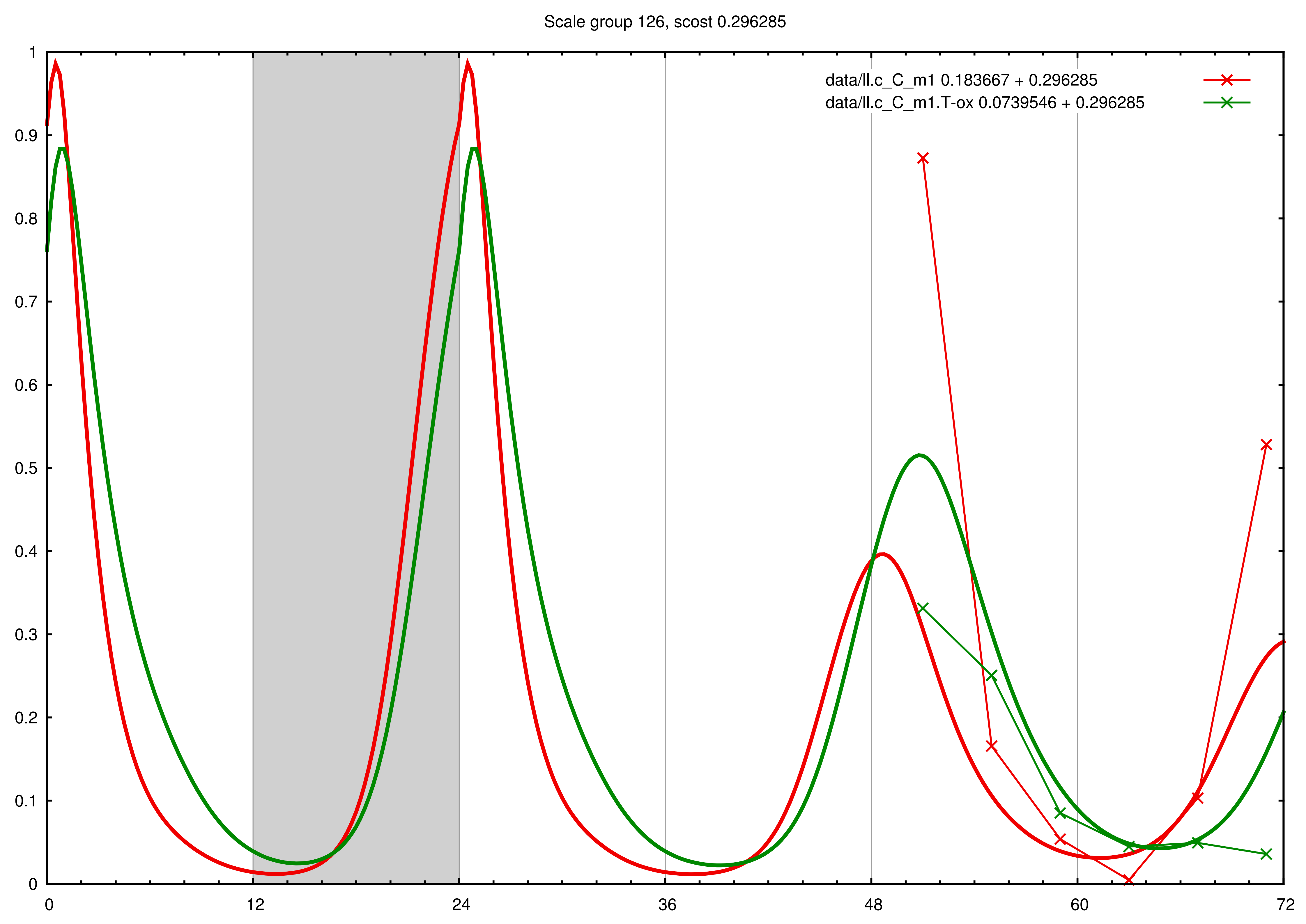

Scale group 126, scost 0.296285
data/ll.c_C_m1 0.183667 + 0.296285
data/ll.c_C_m1.T-ox 0.0739546 + 0.296285
0
0.1
0.2
0.3
0.4
0.5
0.6
0.7
0.8
0.9
1
0
12
24
36
48
60
72

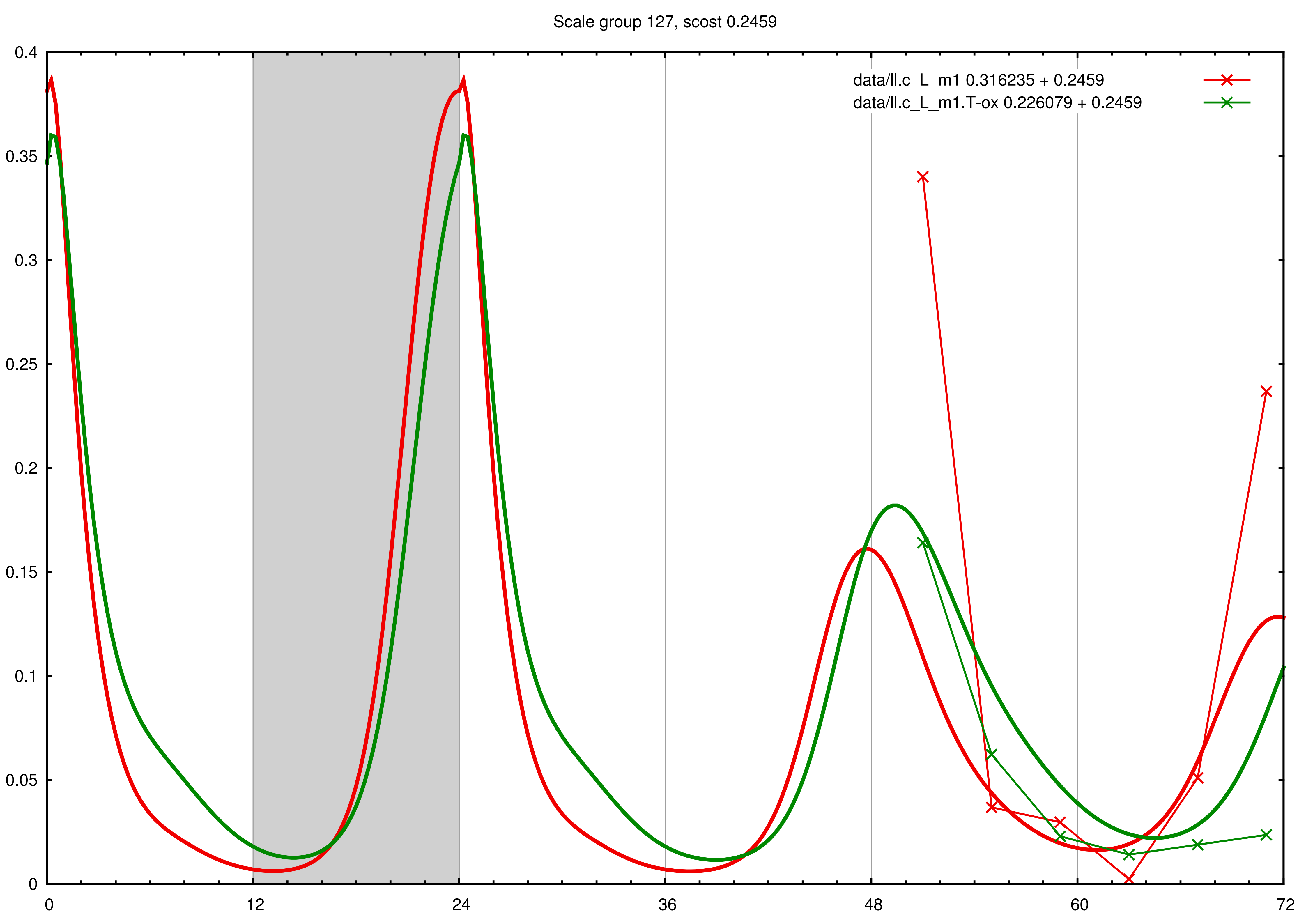
Scale group 127, scost 0.2459
data/ll.c_L_m1 0.316235 + 0.2459
data/ll.c_L_m1.T-ox 0.226079 + 0.2459
0.4
0.35
0.3
0.25
0.2
0.15
0.1
0.05
0
0
12
24
36
48
60
72

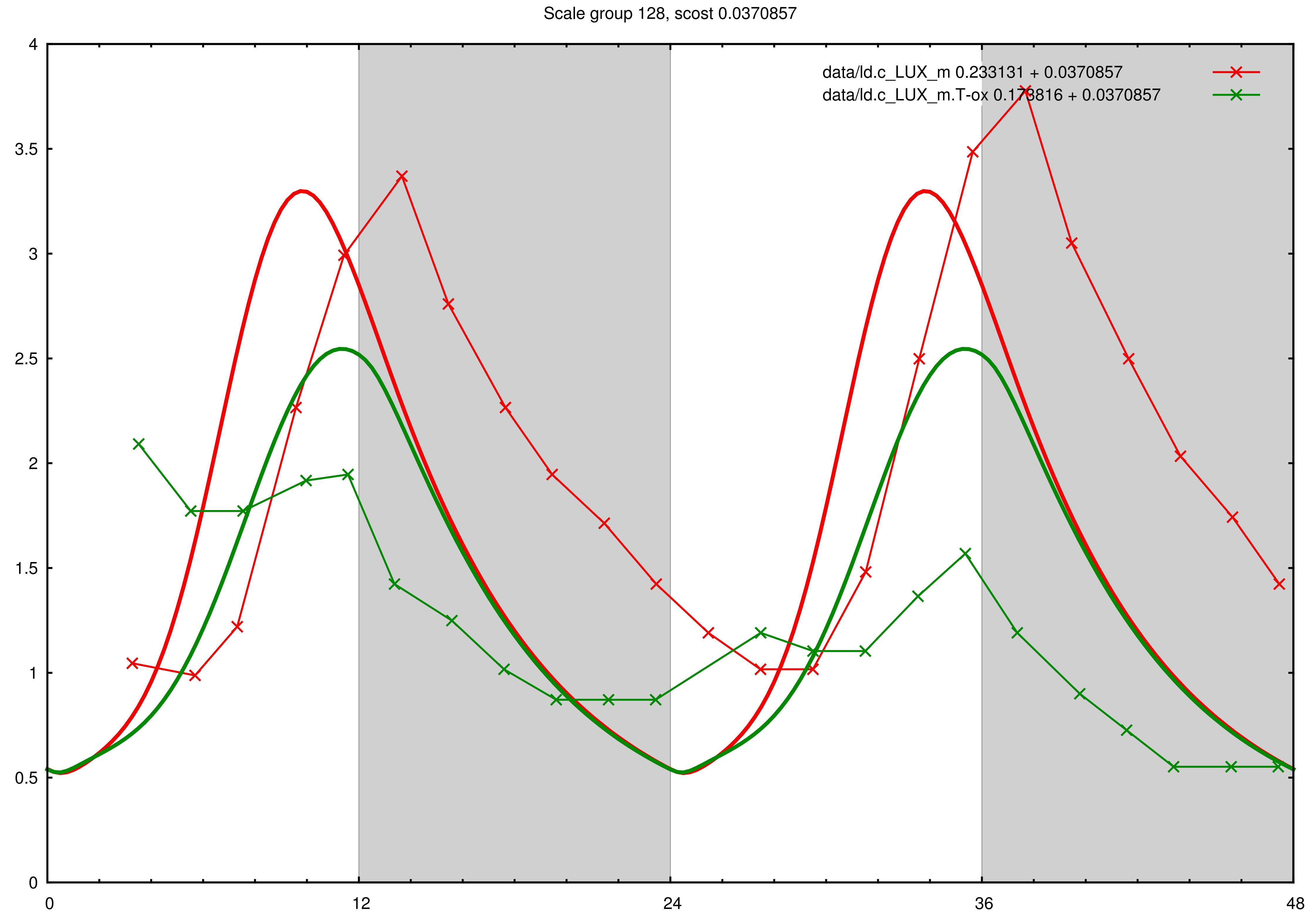
Scale group 128, scost 0.0370857
data/ld.c_LUX_m 0.233131 + 0.0370857
data/ld.c_LUX_m.T-ox 0.173816 + 0.0370857
4
3.5
3
2.5
2
1.5
1
0.5
0
0
12
24
36
48

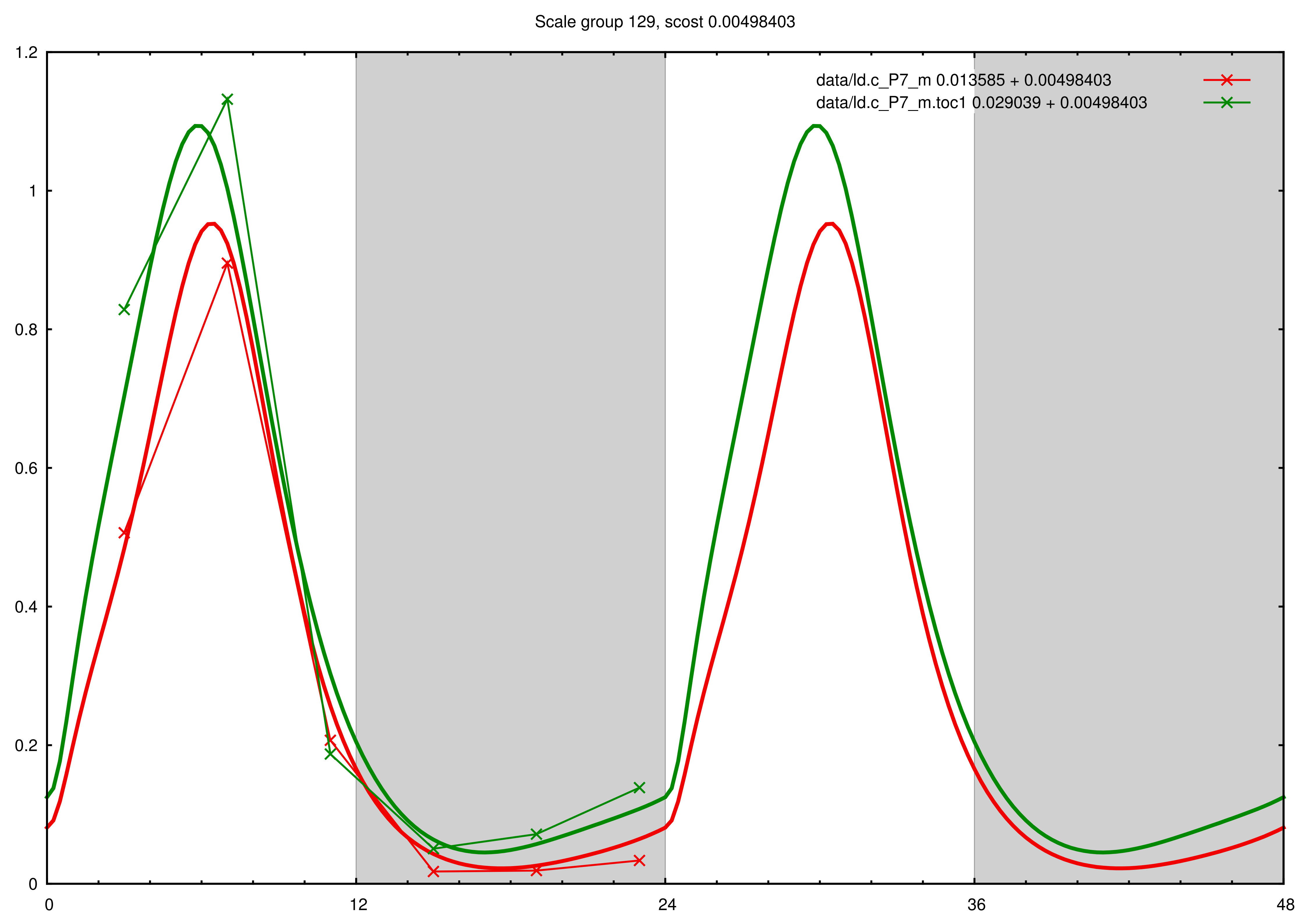

Scale group 129, scost 0.00498403
data/ld.c_P7_m 0.013585 + 0.00498403
data/ld.c_P7_m.toc1 0.029039 + 0.00498403
1.2
1
0.8
0.6
0.4
0.2
0
0
12
24
36
48

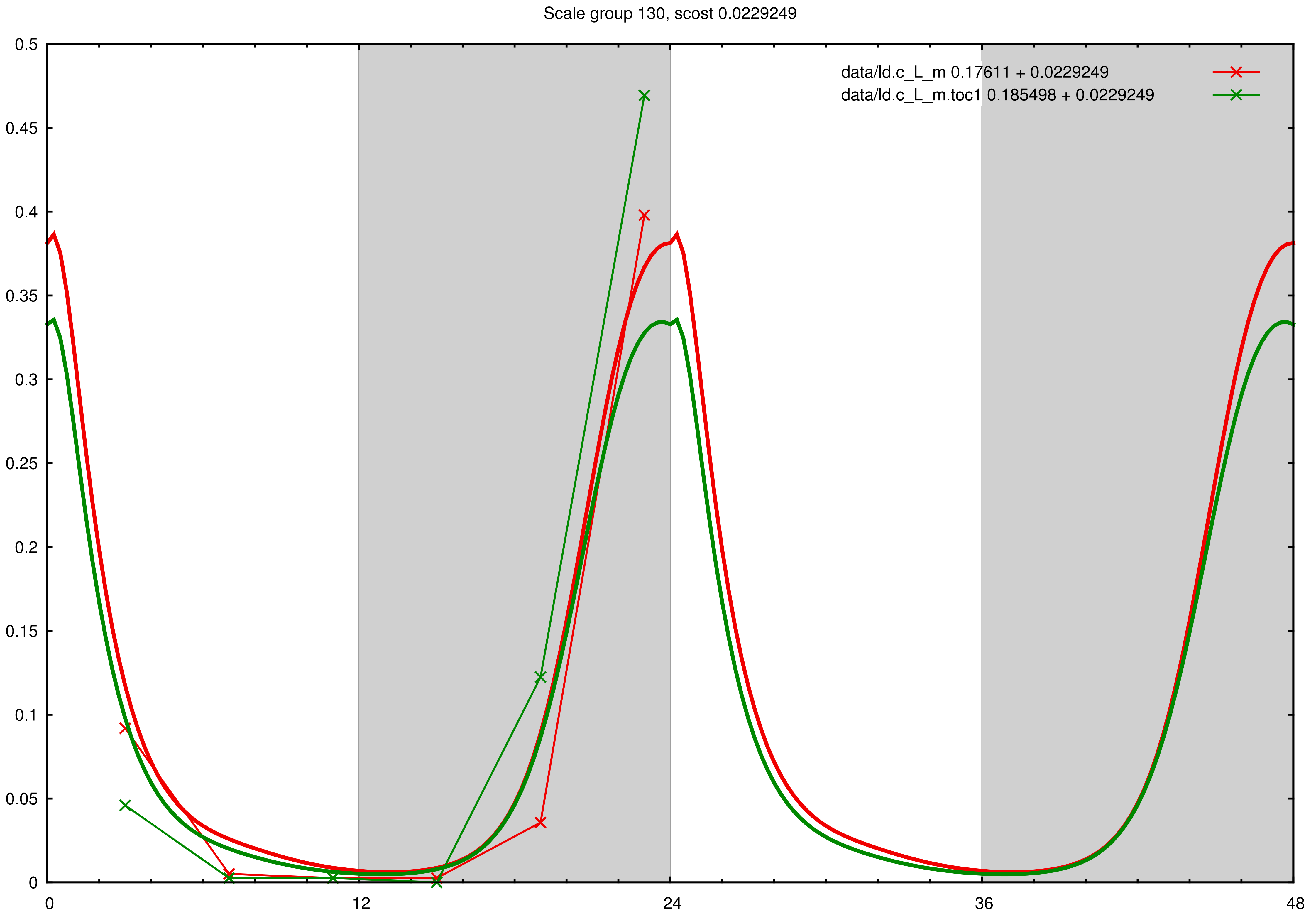

Scale group 130, scost 0.0229249
data/ld.c_L_m 0.17611 + 0.0229249
data/ld.c_L_m.toc1 0.185498 + 0.0229249
0.5
0.45
0.4
0.35
0.3
0.25
0.2
0.15
0.1
0.05
0
0
12
24
36
48

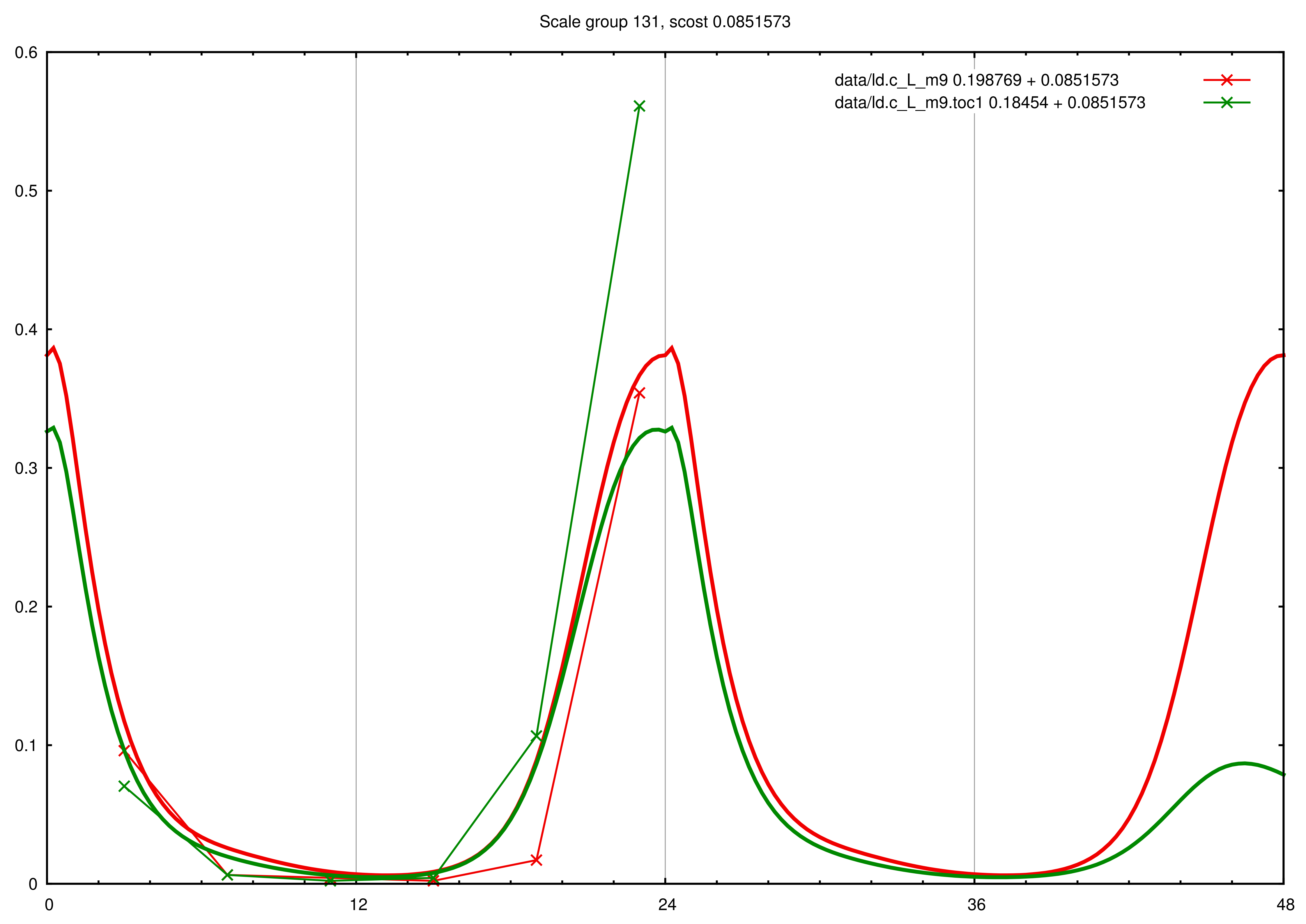

Scale group 131, scost 0.0851573
data/ld.c_L_m9 0.198769 + 0.0851573
data/ld.c_L_m9.toc1 0.18454 + 0.0851573
0.6
0.5
0.4
0.3
0.2
0.1
0
0
12
24
36
48

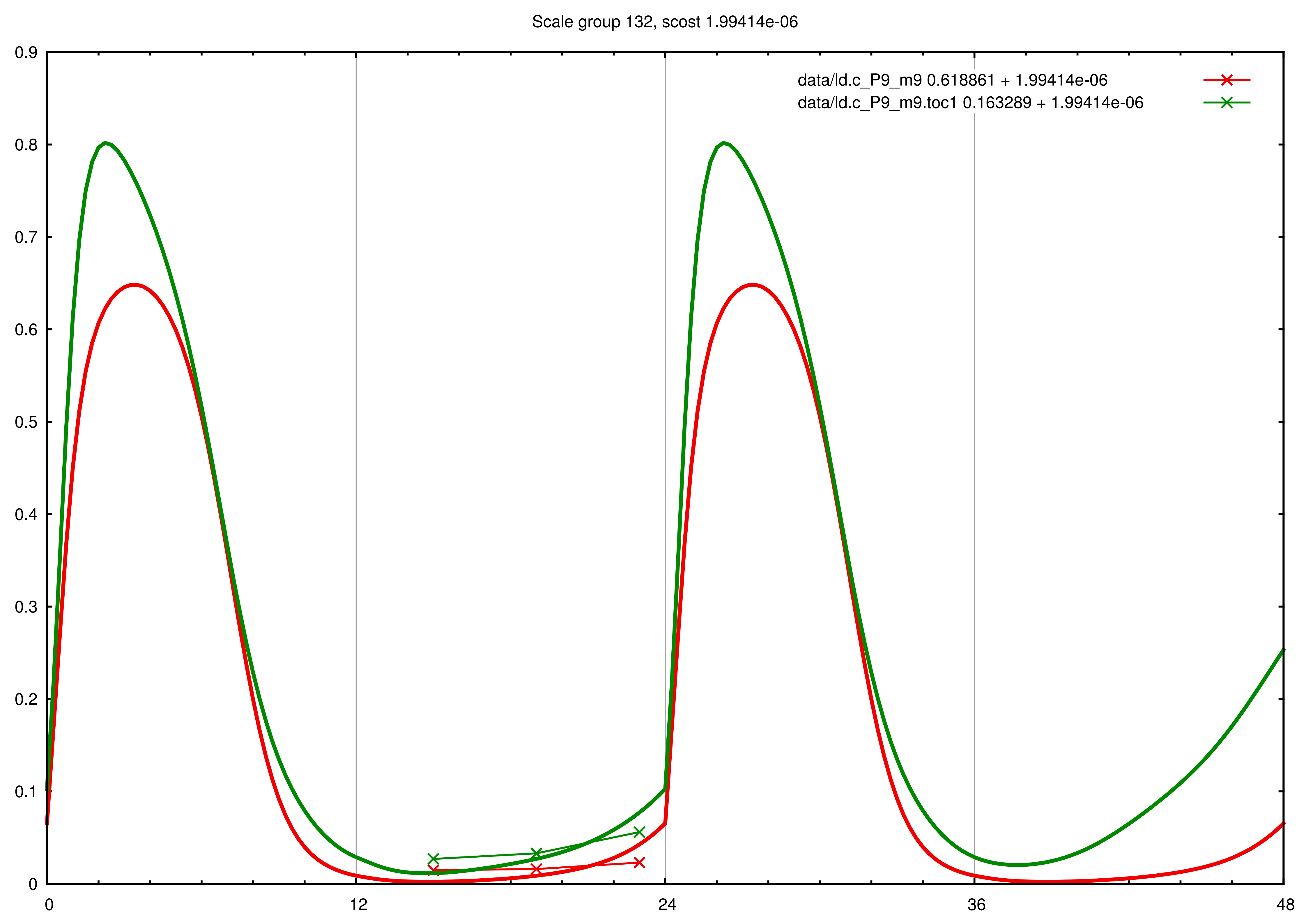

Scale group 132, scost 1.99414e-06
data/ld.c_P9_m9 0.618861 + 1.99414e-06
data/ld.c_P9_m9.toc1 0.163289 + 1.99414e-06
0.9
0.8
0.7
0.6
0.5
0.4
0.3
0.2
0.1
0
0
12
24
36
48

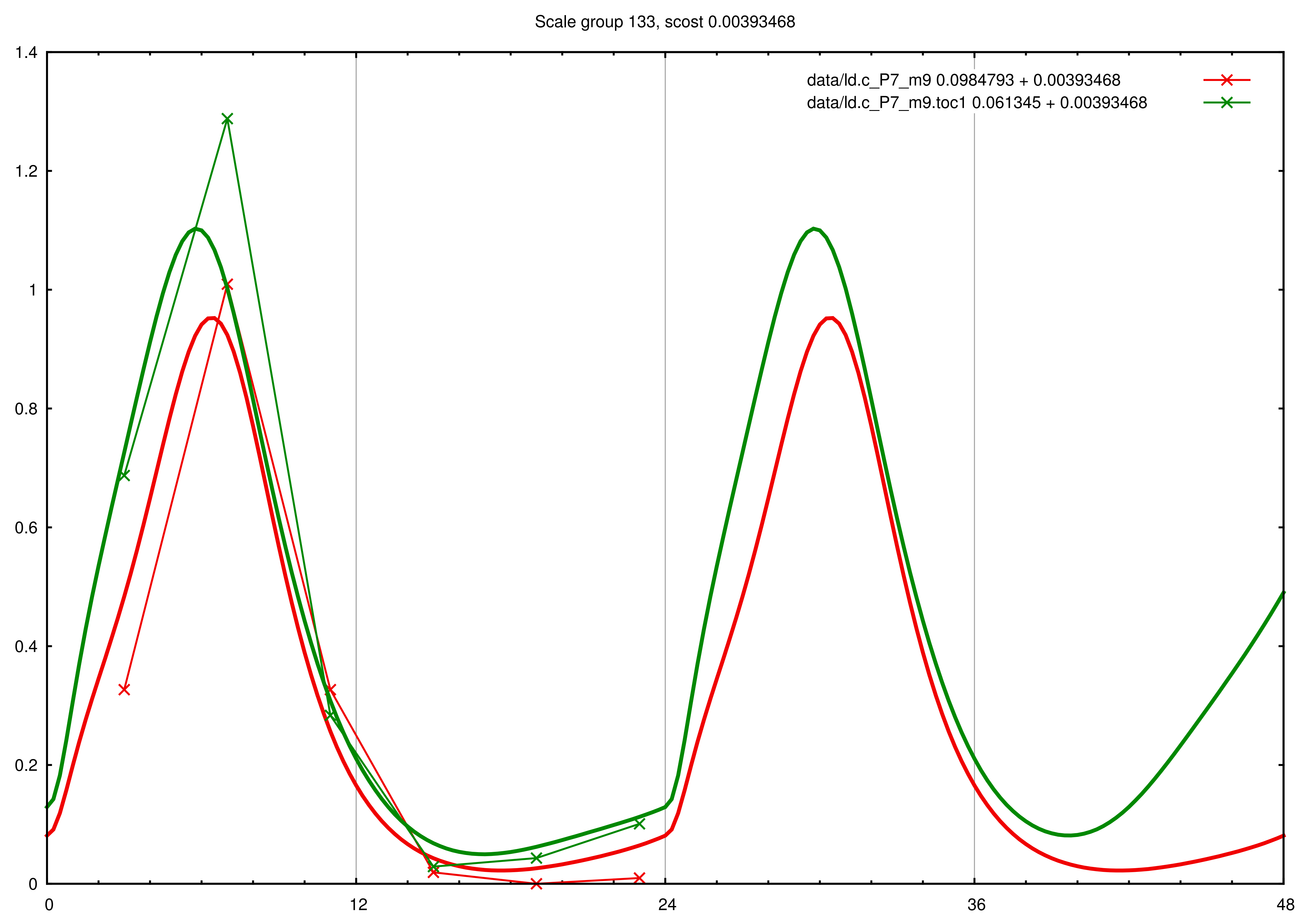

Scale group 133, scost 0.00393468
data/ld.c_P7_m9 0.0984793 + 0.00393468
data/ld.c_P7_m9.toc1 0.061345 + 0.00393468
1.4
1.2
1
0.8
0.6
0.4
0.2
0
0
12
24
36
48

Scale group 134, scost 0.184465

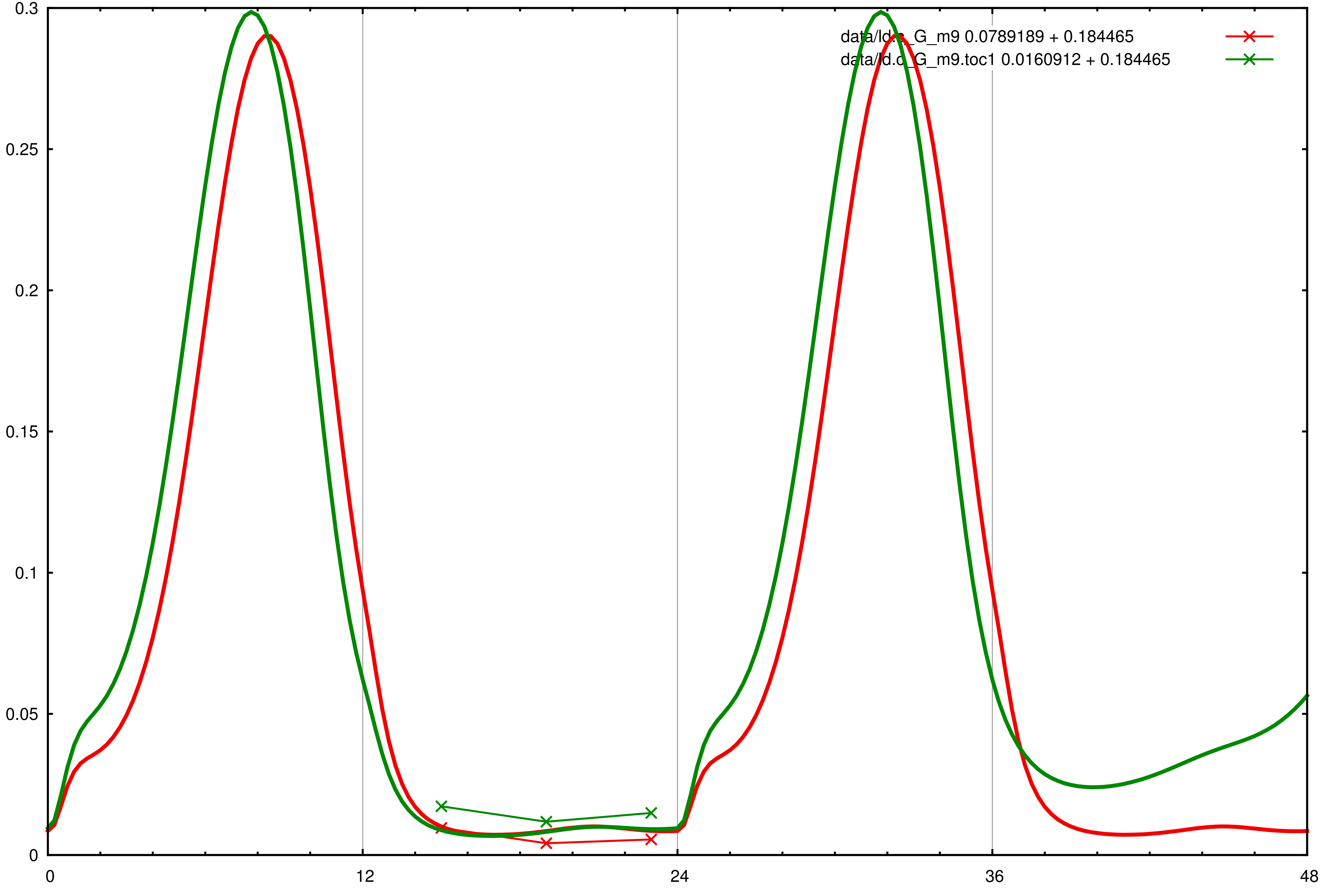

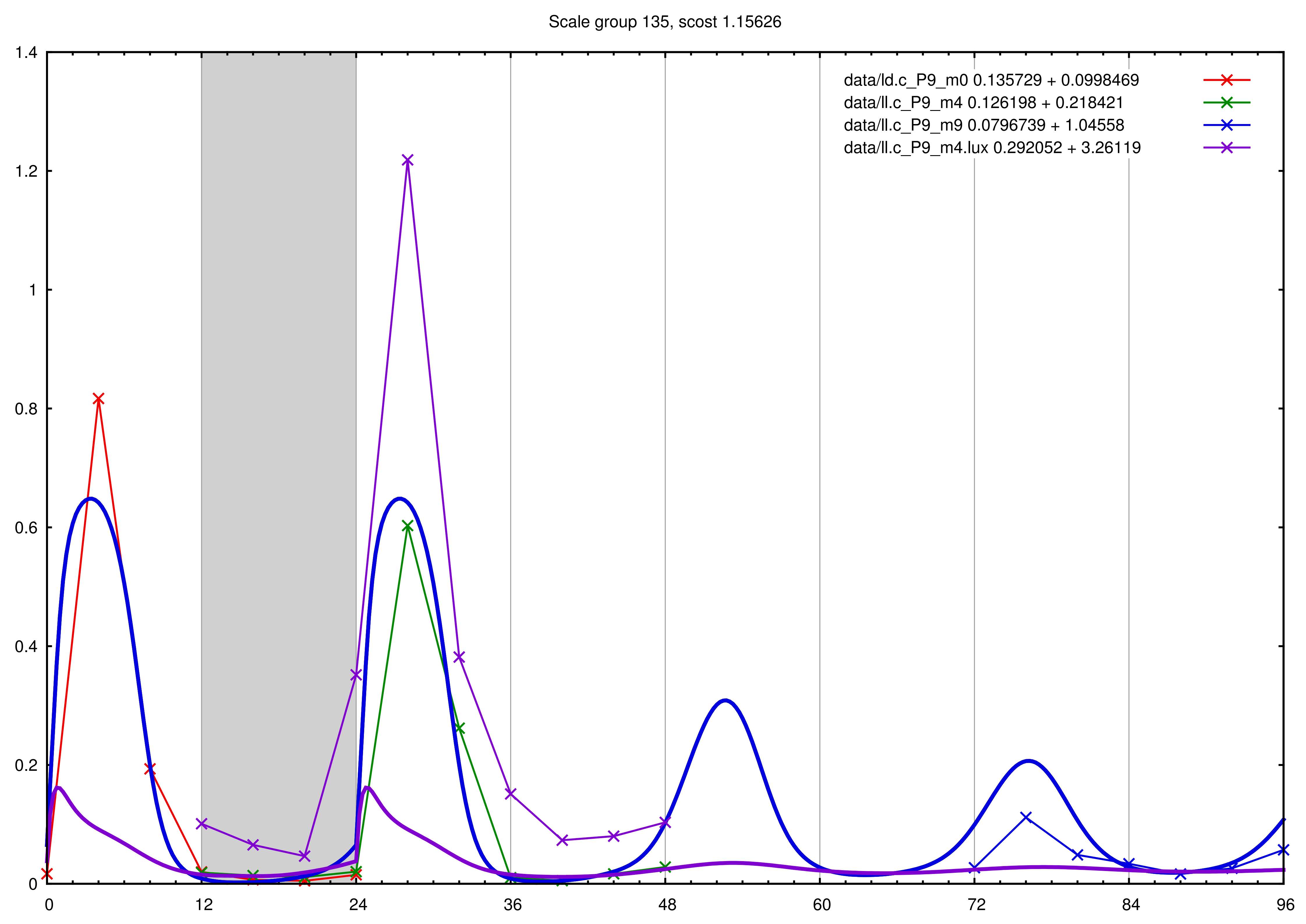

Scale group 135, scost 1.15626
data/ld.c_P9_m0 0.135729 + 0.0998469
data/ll.c_P9_m4 0.126198 + 0.218421
data/ll.c_P9_m9 0.0796739 + 1.04558
data/ll.c_P9_m4.lux 0.292052 + 3.26119
0
0.2
0.4
0.6
0.8
1
1.2
1.4
0
12
24
36
48
60
72
84
96

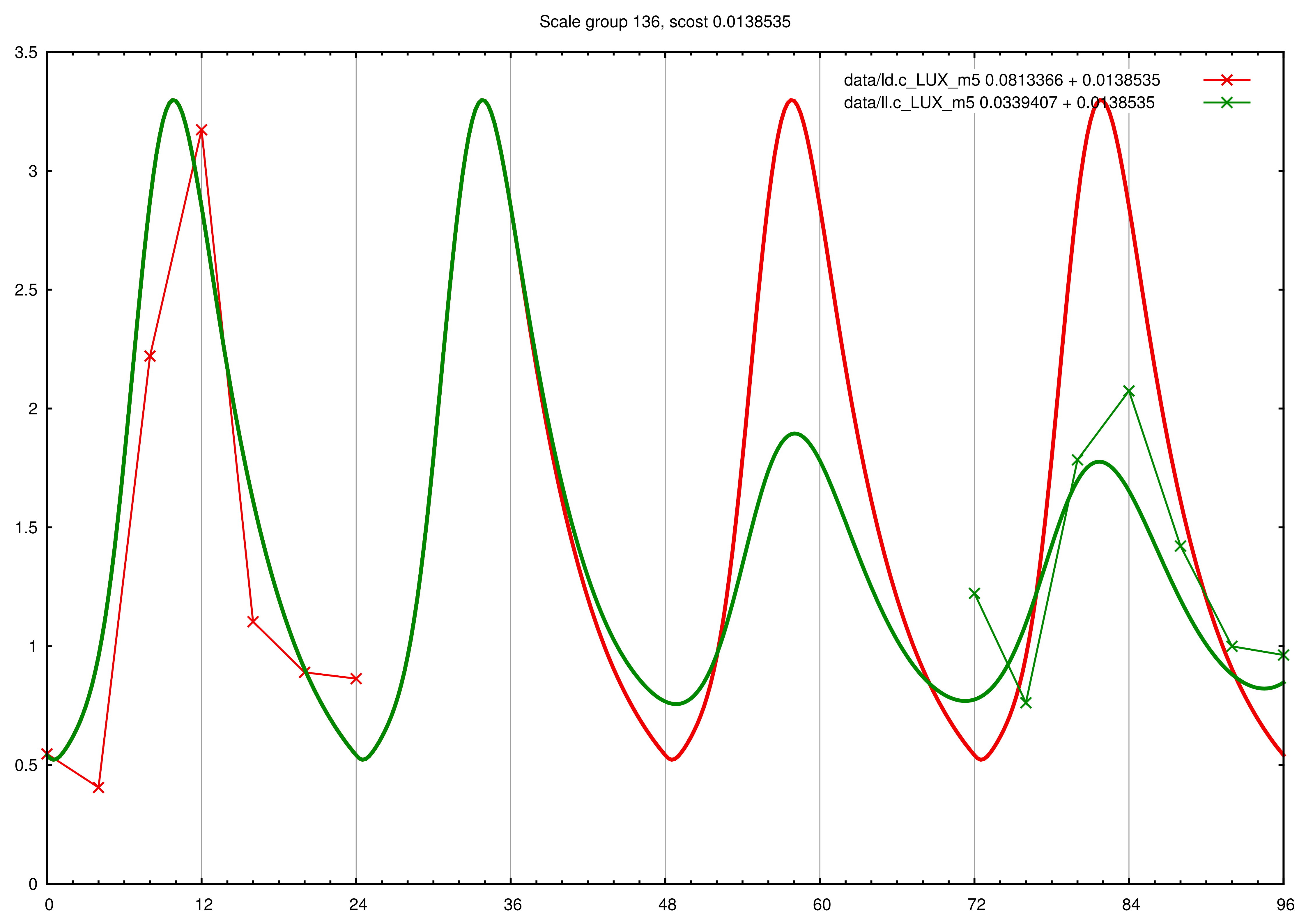

Scale group 136, scost 0.0138535
data/ld.c_LUX_m5 0.0813366 + 0.0138535
data/ll.c_LUX_m5 0.0339407 + 0.0138535
3.5
3
2.5
2
1.5
1
0.5
0
0
12
24
36
48
60
72
84
96

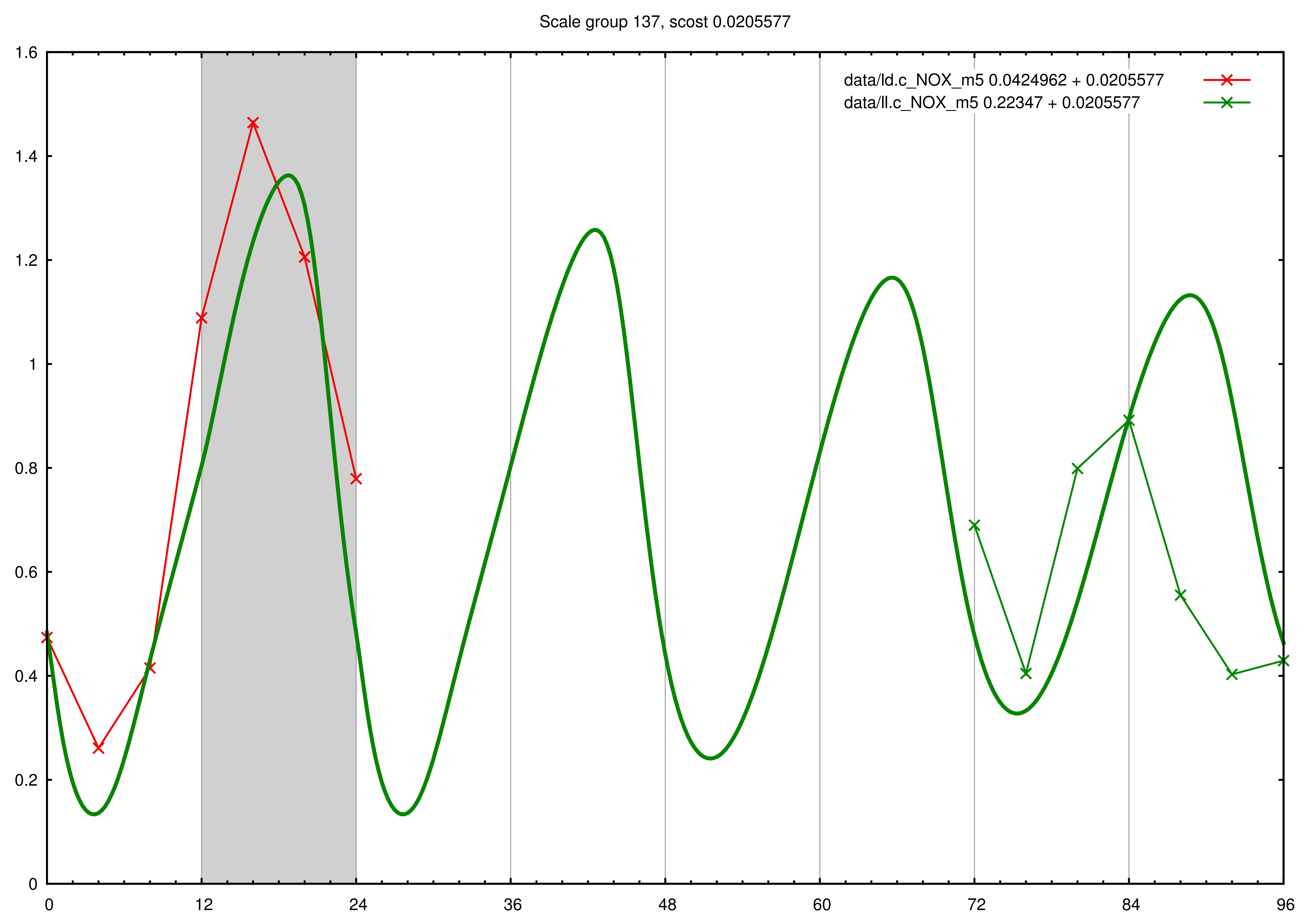

Scale group 137, scost 0.0205577
data/ld.c_NOX_m5 0.0424962 + 0.0205577
data/ll.c_NOX_m5 0.22347 + 0.0205577
0
0.2
0.4
0.6
0.8
1
1.2
1.4
1.6
0
12
24
36
48
60
72
84
96

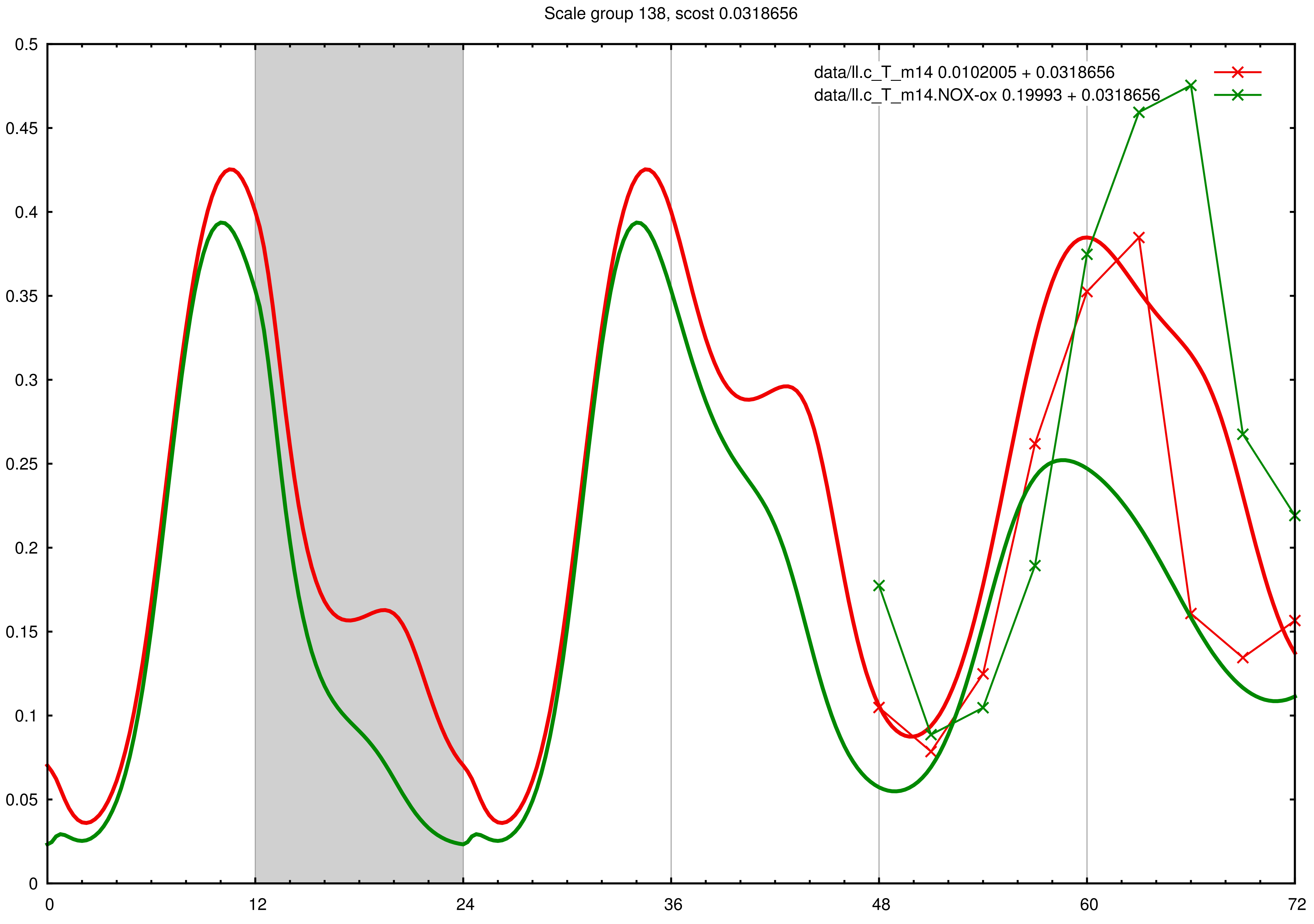
Scale group 138, scost 0.0318656
data/ll.c_T_m14 0.0102005 + 0.0318656
data/ll.c_T_m14.NOX-ox 0.19993 + 0.0318656
0.5
0.45
0.4
0.35
0.3
0.25
0.2
0.15
0.1
0.05
0
0
12
24
36
48
60
72

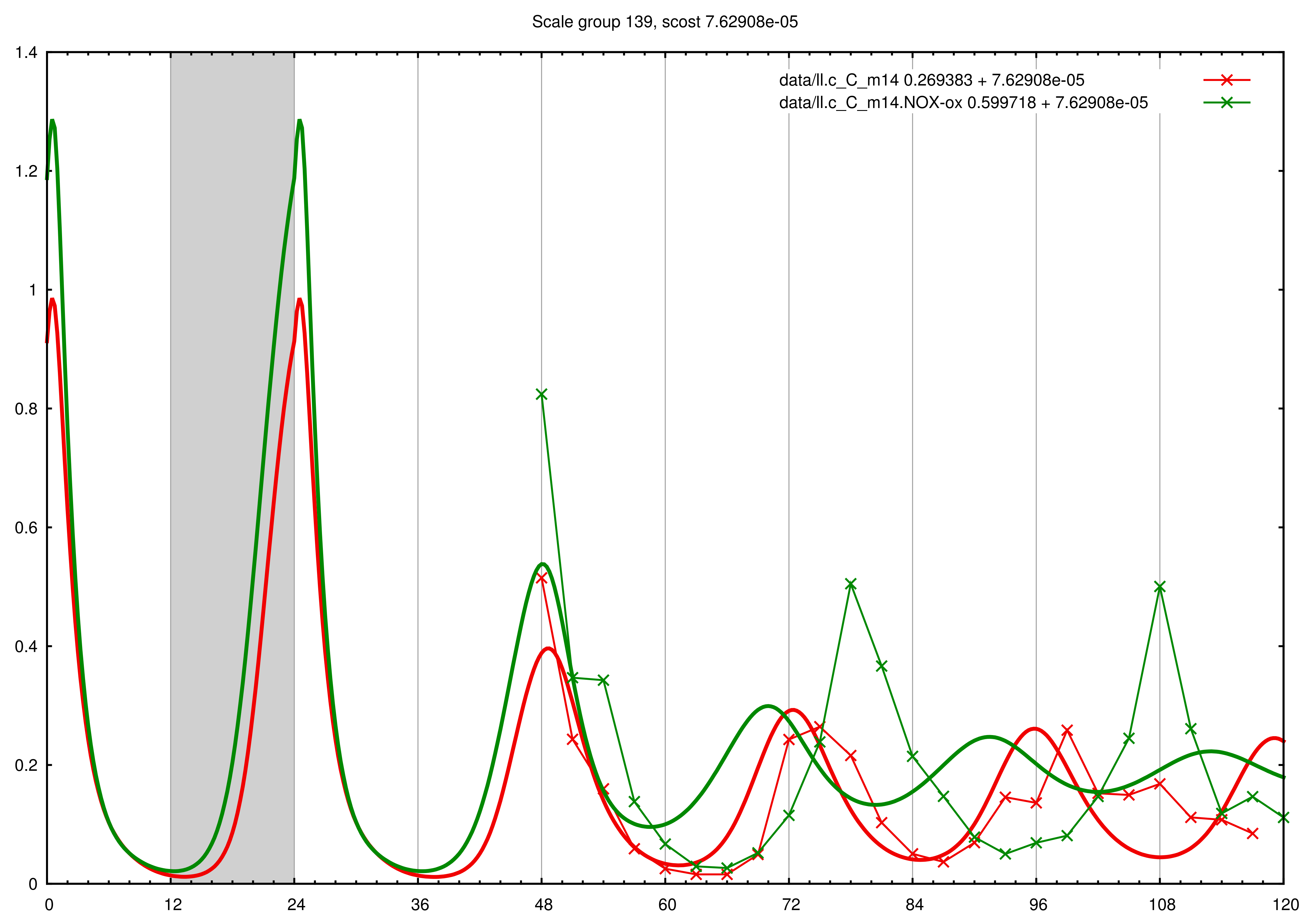

Scale group 139, scost 7.62908e-05
data/ll.c_C_m14 0.269383 + 7.62908e-05
data/ll.c_C_m14.NOX-ox 0.599718 + 7.62908e-05
0
0.2
0.4
0.6
0.8
1
1.2
1.4
0
12
24
36
48
60
72
84
96
108
120

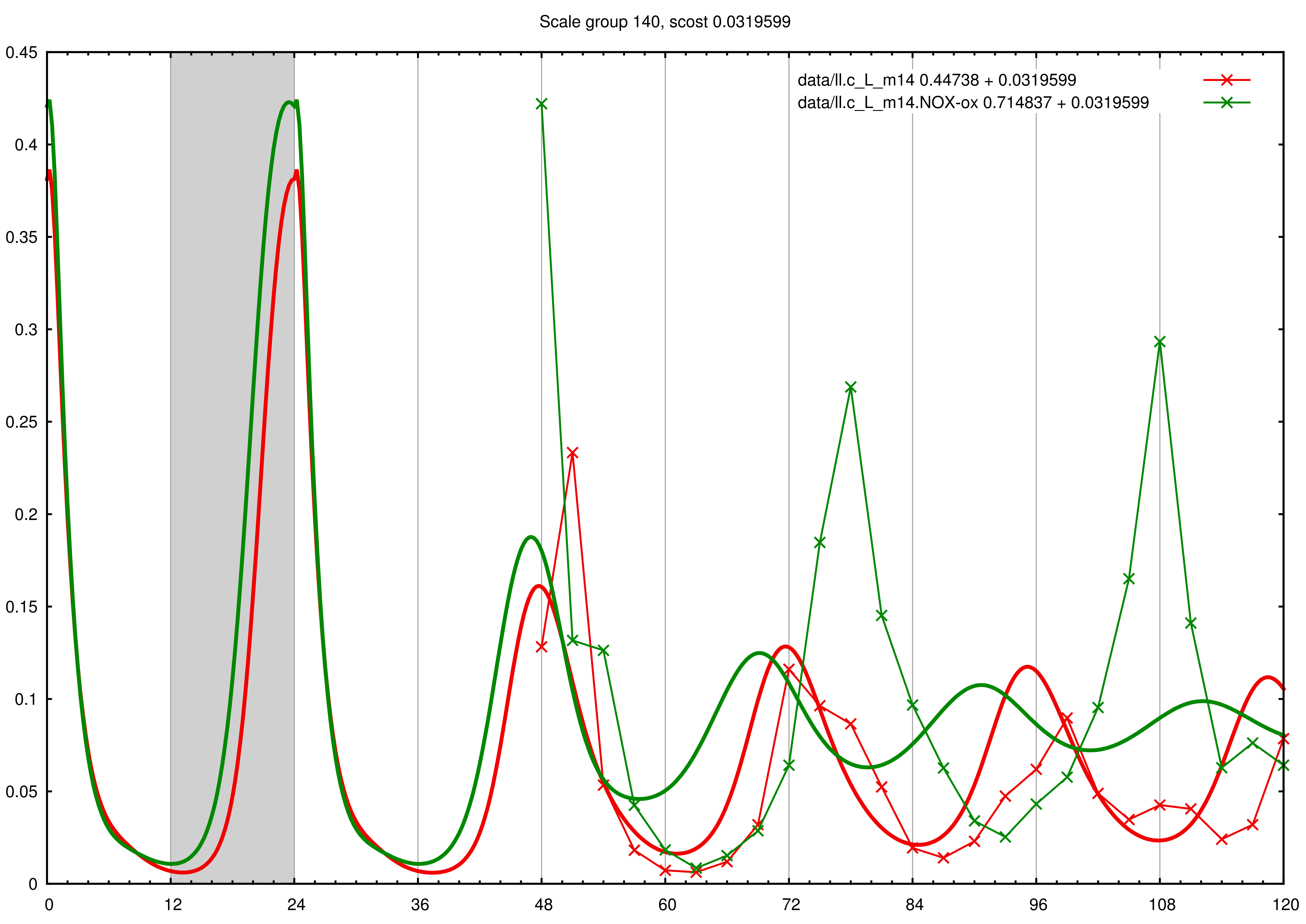
Scale group 140, scost 0.0319599
data/ll.c_L_m14 0.44738 + 0.0319599
data/ll.c_L_m14.NOX-ox 0.714837 + 0.0319599
0
0.05
0.1
0.15
0.2
0.25
0.3
0.35
0.4
0.45
0
12
24
36
48
60
72
84
96
108
120

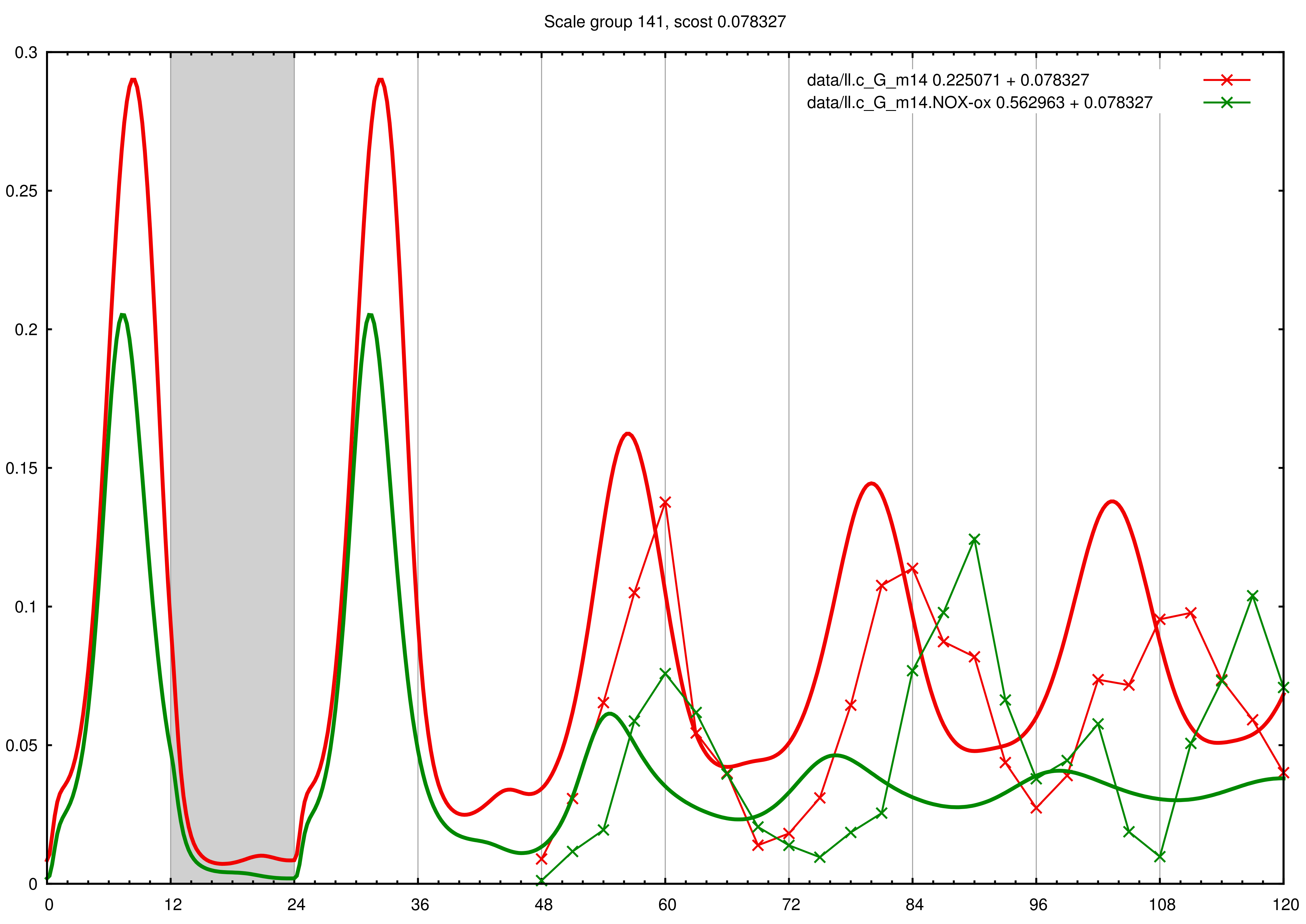

Scale group 141, scost 0.078327
data/ll.c_G_m14 0.225071 + 0.078327
data/ll.c_G_m14.NOX-ox 0.562963 + 0.078327
0.3
0.25
0.2
0.15
0.1
0.05
0
0
12
24
36
48
60
72
84
96
108
120

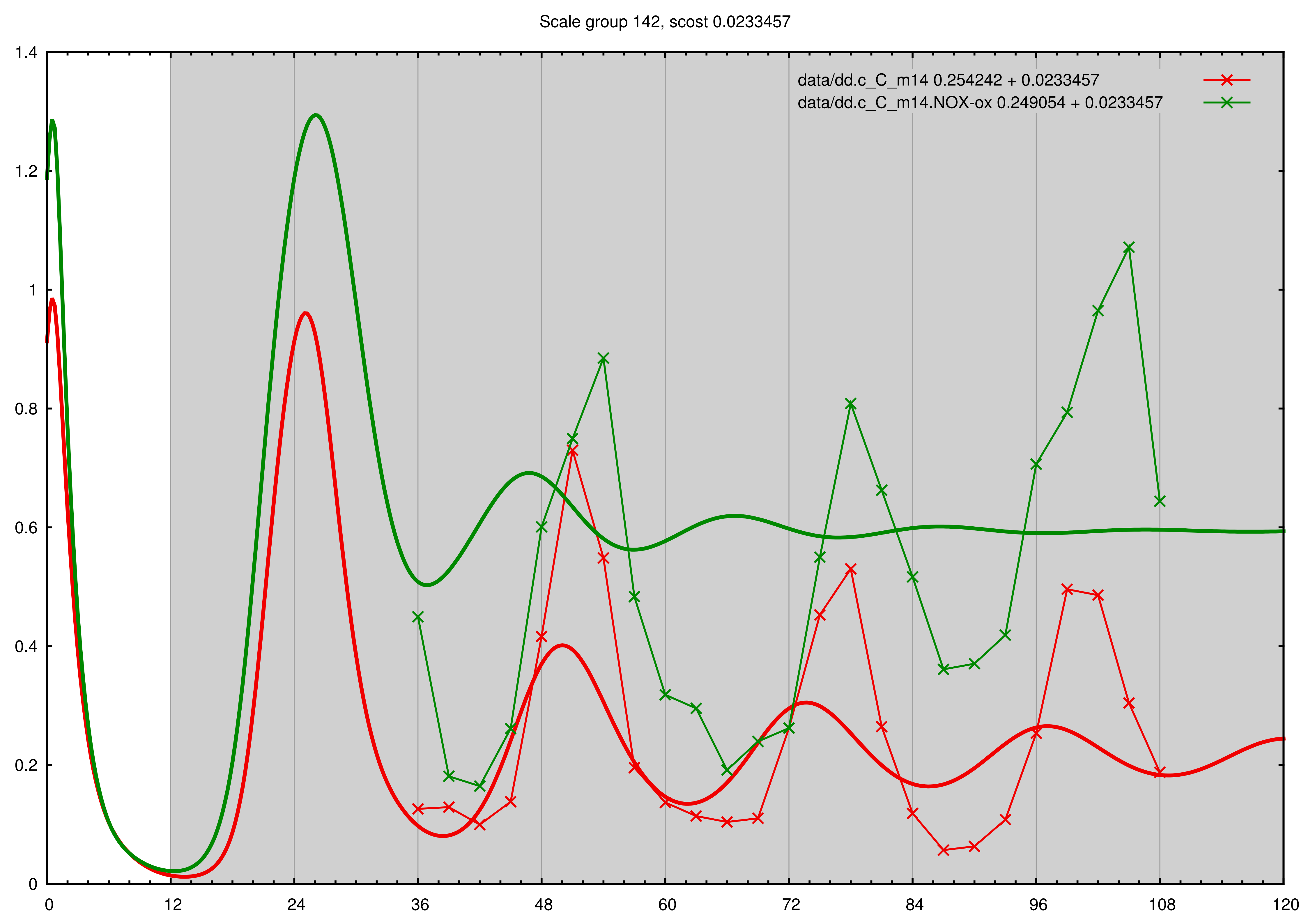

Scale group 142, scost 0.0233457
data/dd.c_C_m14 0.254242 + 0.0233457
data/dd.c_C_m14.NOX-ox 0.249054 + 0.0233457
0
0.2
0.4
0.6
0.8
1
1.2
1.4
0
12
24
36
48
60
72
84
96
108
120

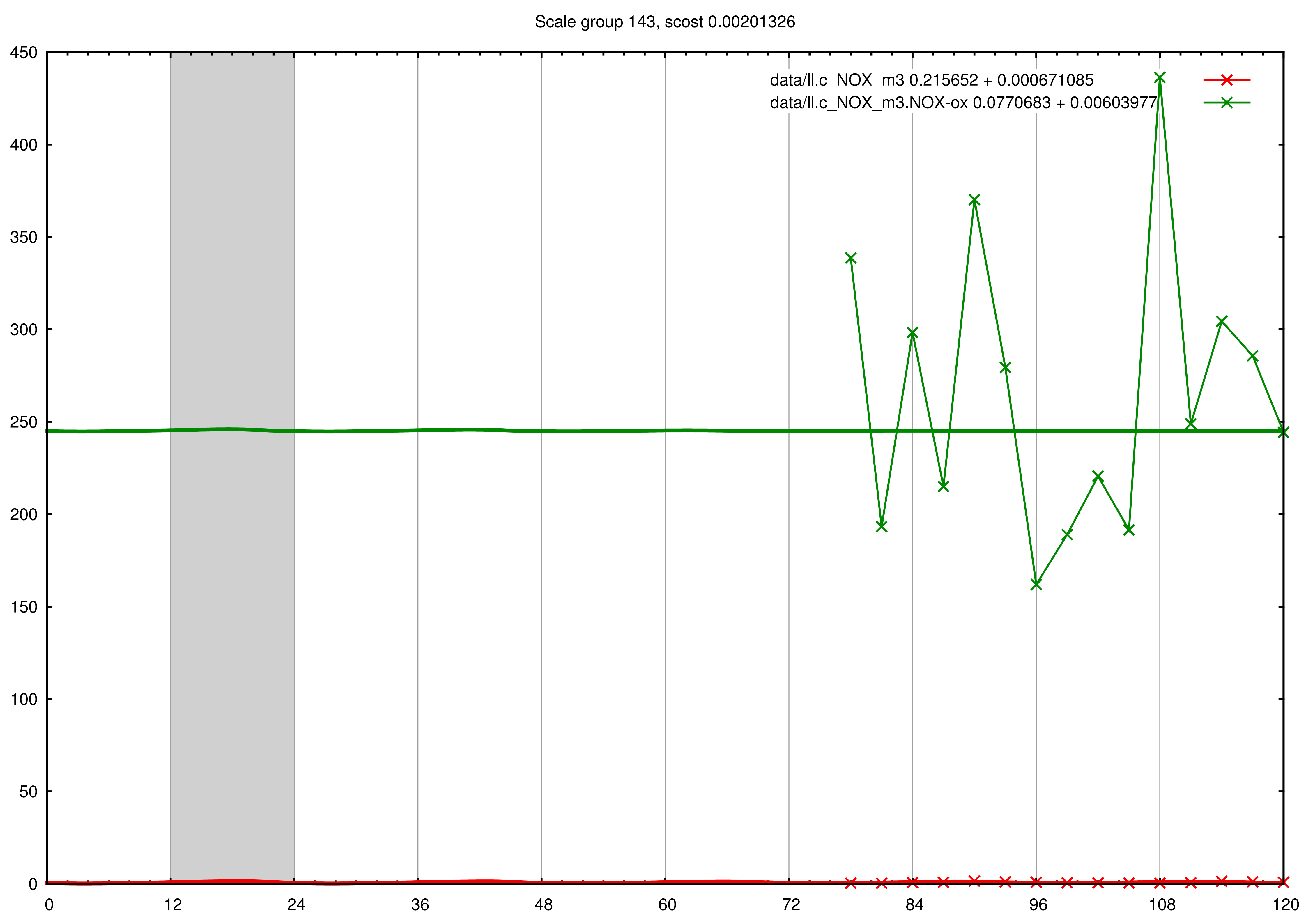
Scale group 143, scost 0.00201326
data/ll.c_NOX_m3 0.215652 + 0.000671085
data/ll.c_NOX_m3.NOX-ox 0.0770683 + 0.00603977
450
400
350
300
250
200
150
100
50
0
0
12
24
36
48
60
72
84
96
108
120

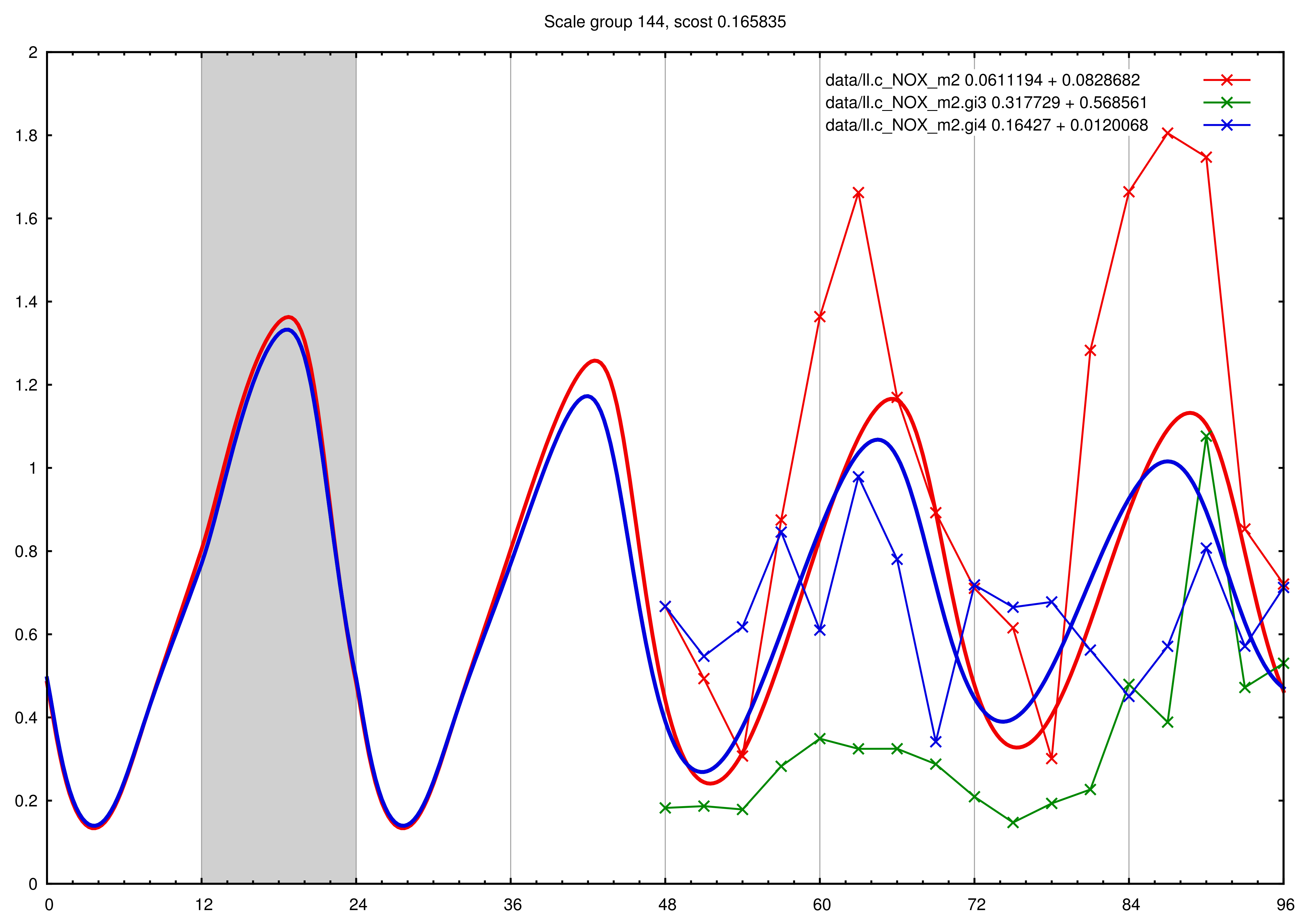

Scale group 144, scost 0.165835
data/ll.c_NOX_m2 0.0611194 + 0.0828682
data/ll.c_NOX_m2.gi3 0.317729 + 0.568561
data/ll.c_NOX_m2.gi4 0.16427 + 0.0120068
2
1.8
1.6
1.4
1.2
1
0.8
0.6
0.4
0.2
0
0
12
24
36
48
60
72
84
96

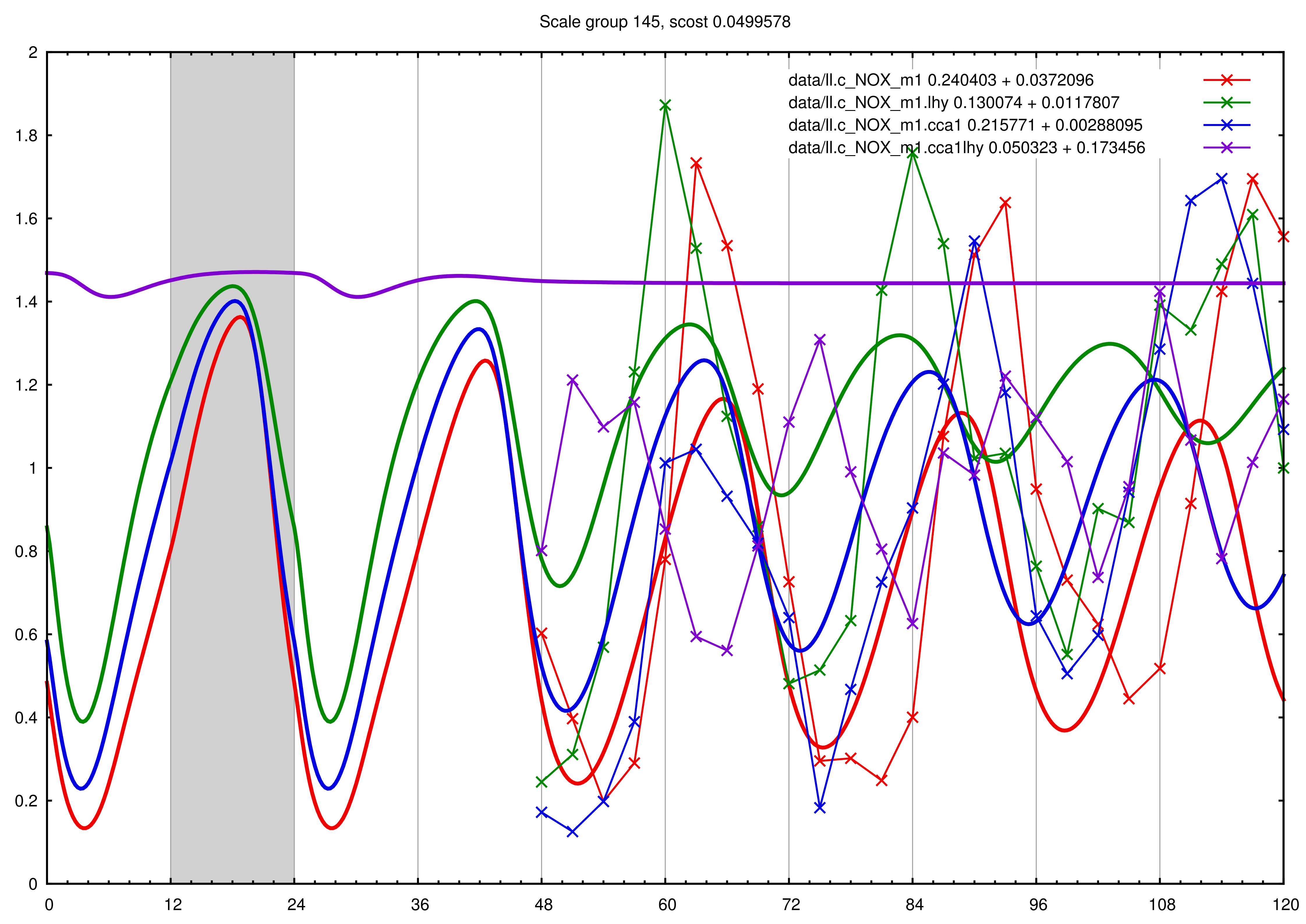

Scale group 145, scost 0.0499578
data/ll.c_NOX_m1 0.240403 + 0.0372096
data/ll.c_NOX_m1.lhy 0.130074 + 0.0117807
data/ll.c_NOX_m1.cca1 0.215771 + 0.00288095
data/ll.c_NOX_m1.cca1lhy 0.050323 + 0.173456
0
0.2
0.4
0.6
0.8
1
1.2
1.4
1.6
1.8
2
0
12
24
36
48
60
72
84
96
108
120

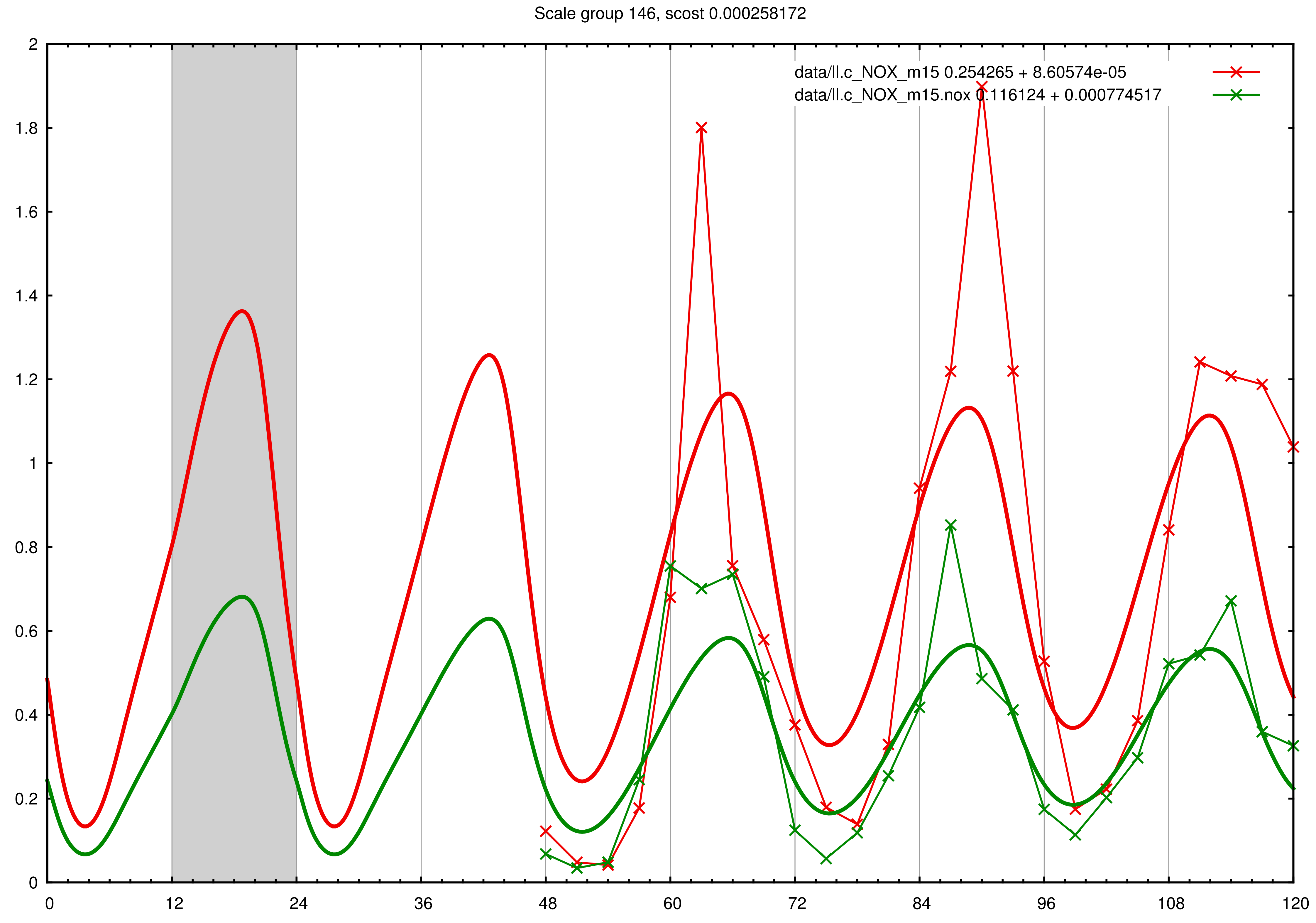
Scale group 146, scost 0.000258172
data/ll.c_NOX_m15 0.254265 + 8.60574e-05
data/ll.c_NOX_m15.nox 0.116124 + 0.000774517
0
0.2
0.4
0.6
0.8
1
1.2
1.4
1.6
1.8
2
0
12
24
36
48
60
72
84
96
108
120

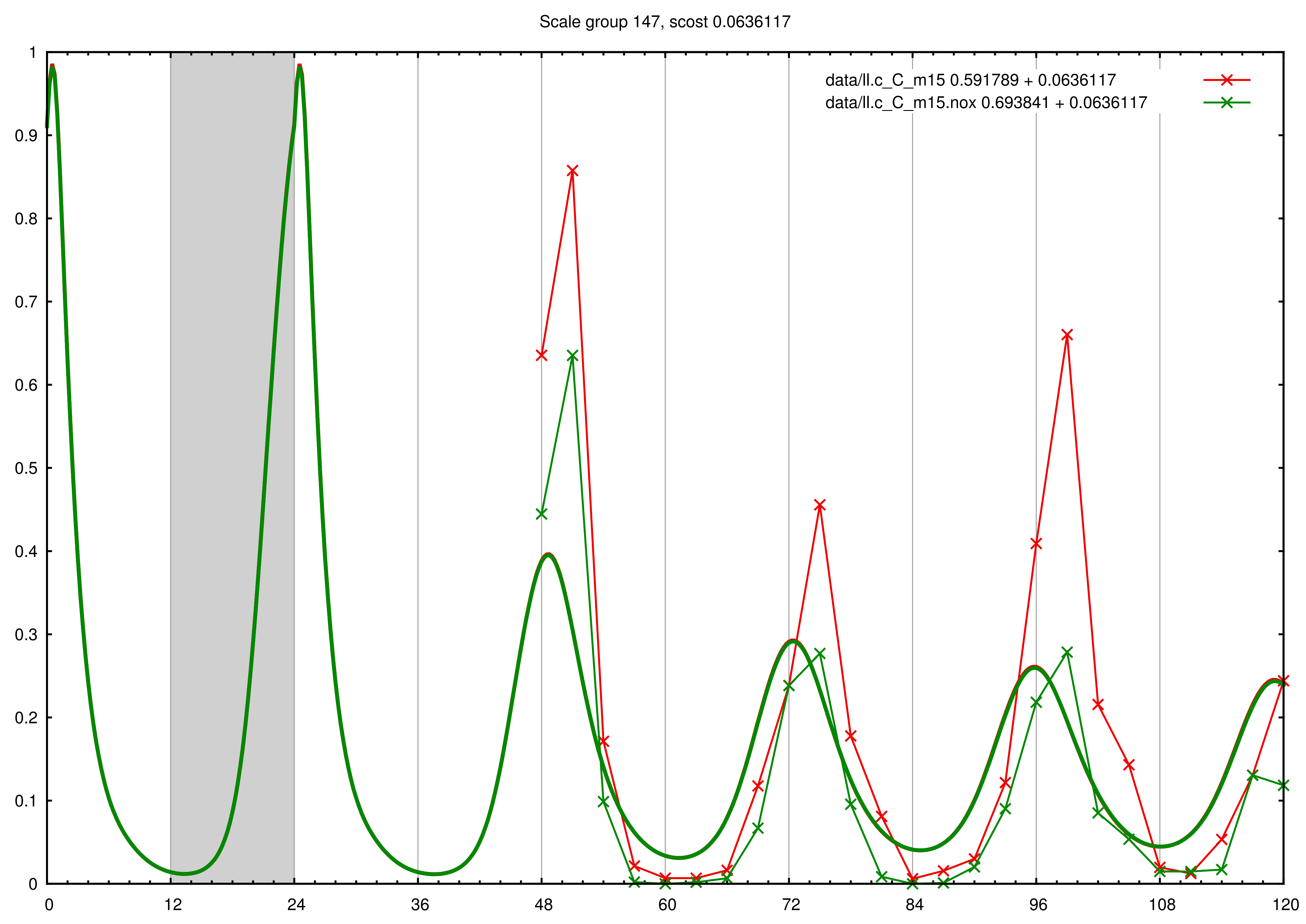

Scale group 147, scost 0.0636117
data/ll.c_C_m15 0.591789 + 0.0636117
data/ll.c_C_m15.nox 0.693841 + 0.0636117
1
0.9
0.8
0.7
0.6
0.5
0.4
0.3
0.2
0.1
0
0
12
24
36
48
60
72
84
96
108
120

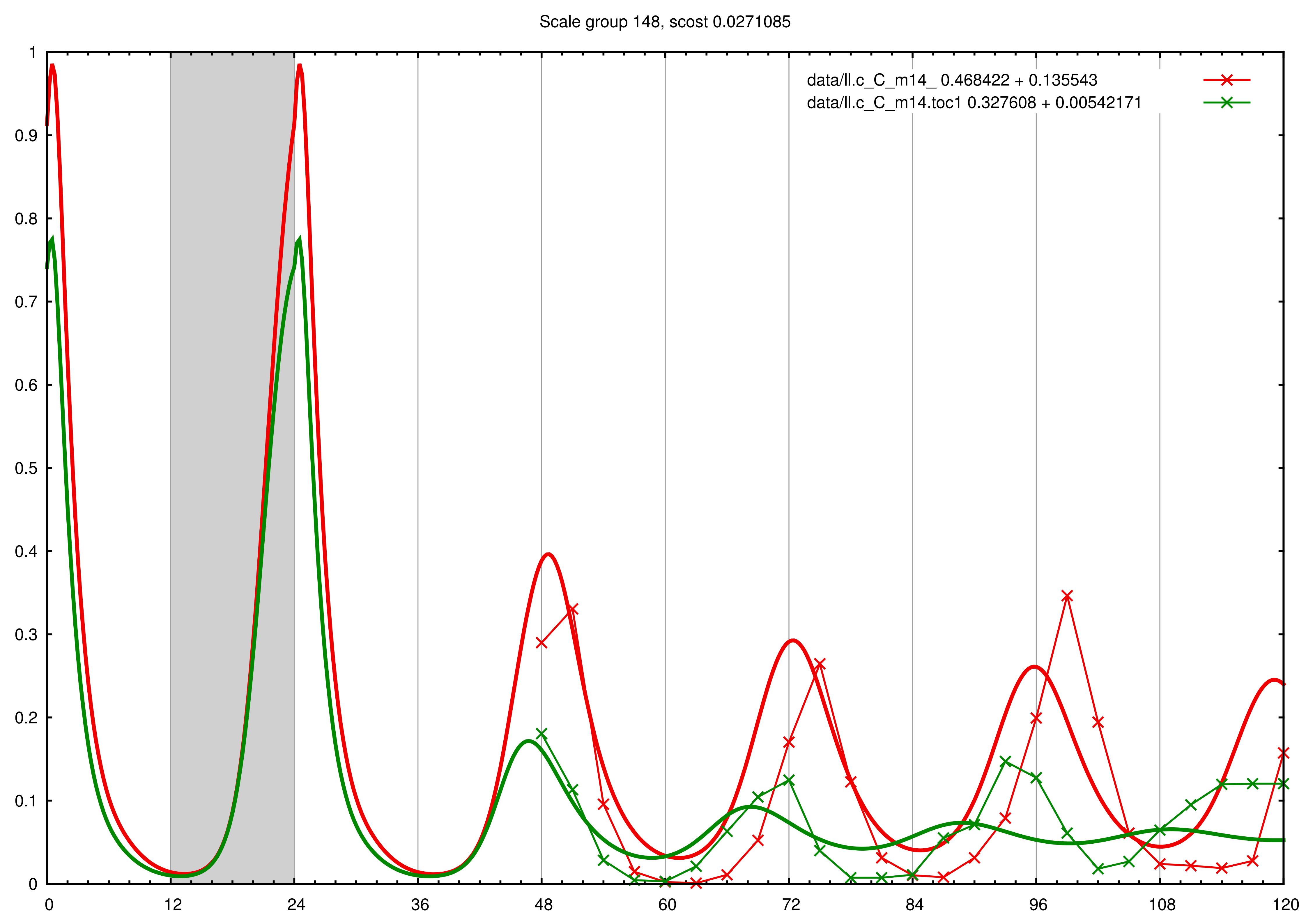

Scale group 148, scost 0.0271085
data/ll.c_C_m14_ 0.468422 + 0.135543
data/ll.c_C_m14.toc1 0.327608 + 0.00542171
1
0.9
0.8
0.7
0.6
0.5
0.4
0.3
0.2
0.1
0
0
12
24
36
48
60
72
84
96
108
120

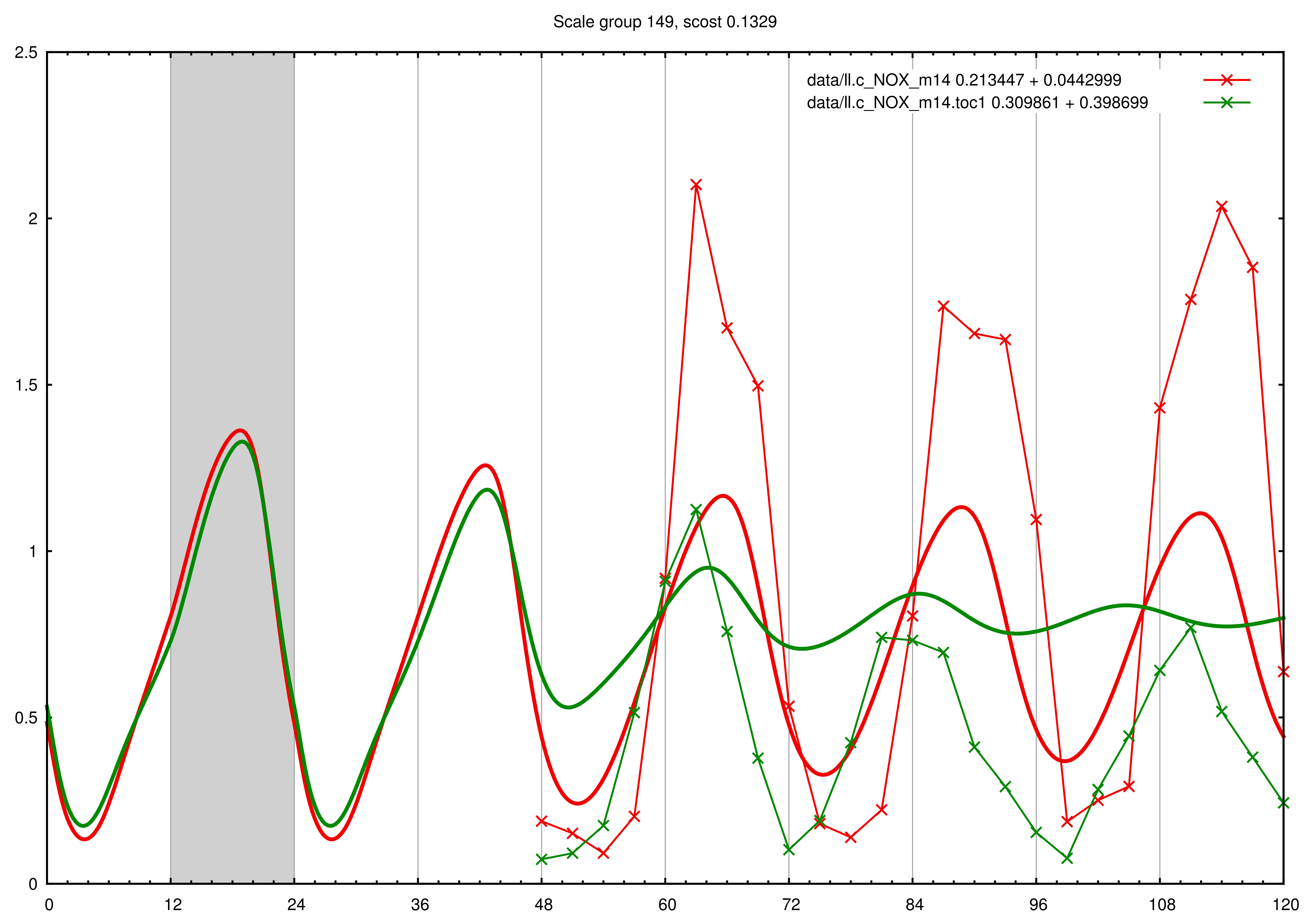

Scale group 149, scost 0.1329
data/ll.c_NOX_m14 0.213447 + 0.0442999
data/ll.c_NOX_m14.toc1 0.309861 + 0.398699
2.5
2
1.5
1
0.5
0
0
12
24
36
48
60
72
84
96
108
120

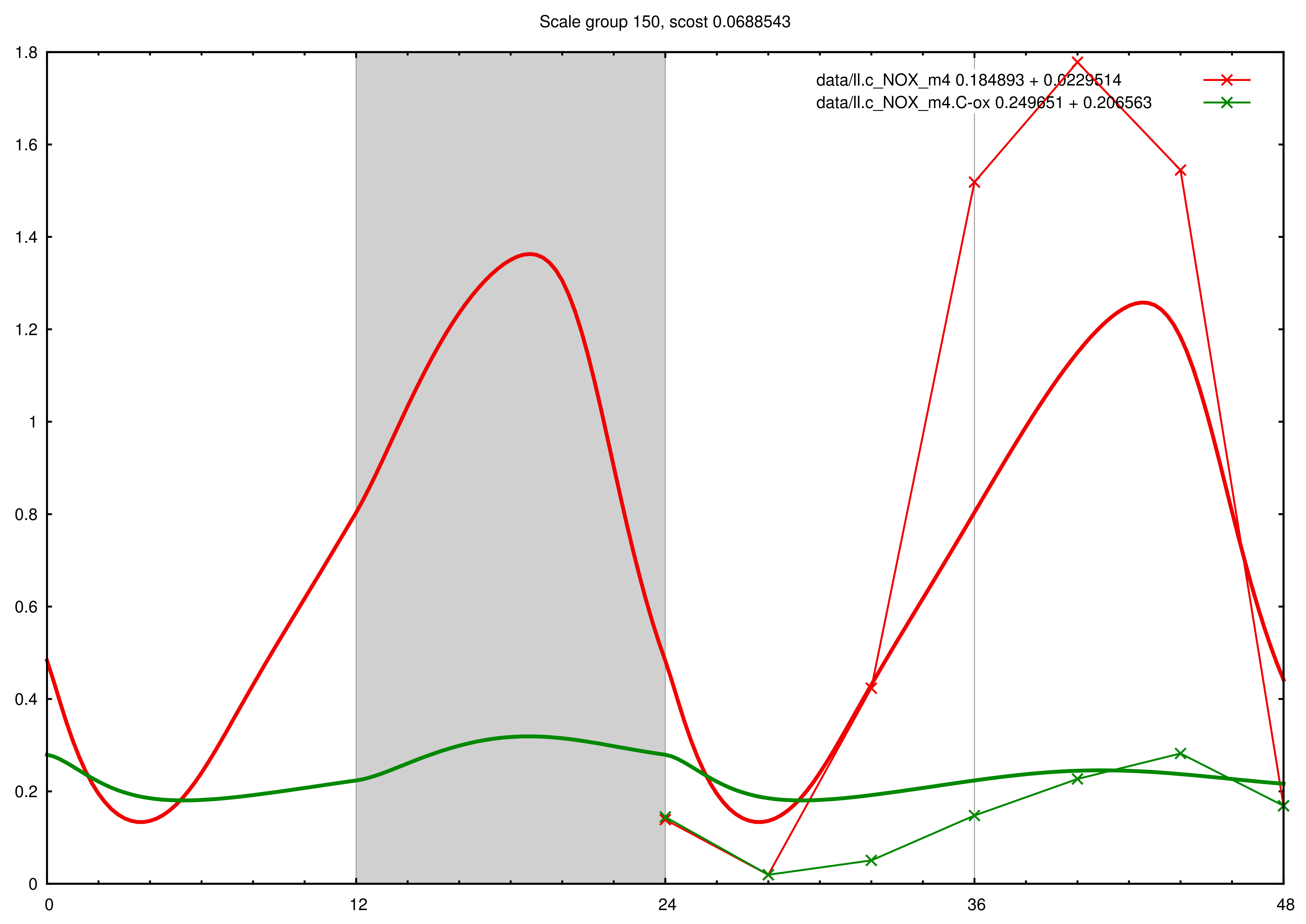

Scale group 150, scost 0.0688543
data/ll.c_NOX_m4 0.184893 + 0.0229514
data/ll.c_NOX_m4.C-ox 0.249651 + 0.206563
1.8
1.6
1.4
1.2
1
0.8
0.6
0.4
0.2
0
0
12
24
36
48

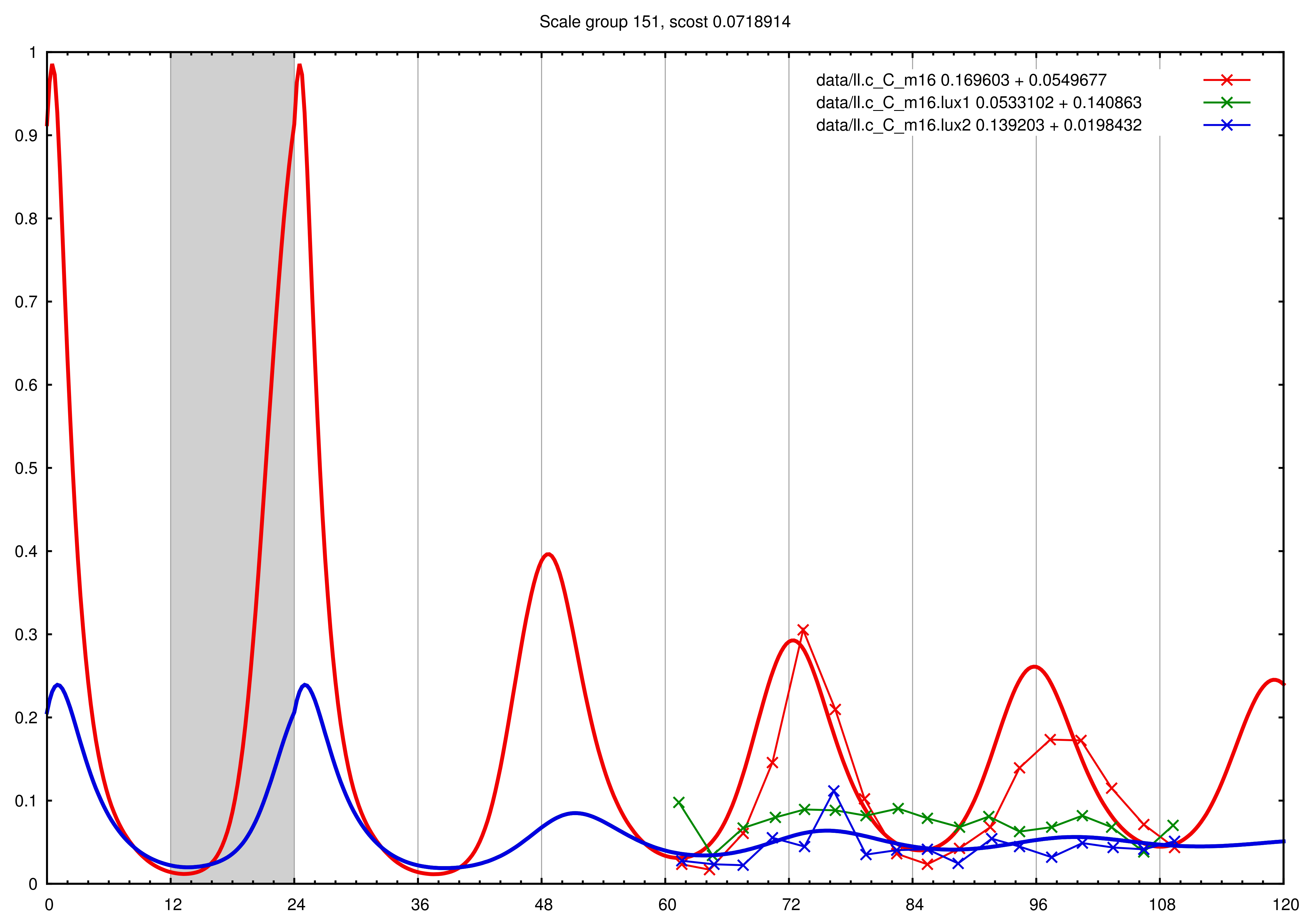

Scale group 151, scost 0.0718914
data/ll.c_C_m16 0.169603 + 0.0549677
data/ll.c_C_m16.lux1 0.0533102 + 0.140863
data/ll.c_C_m16.lux2 0.139203 + 0.0198432
0
0.1
0.2
0.3
0.4
0.5
0.6
0.7
0.8
0.9
1
0
12
24
36
48
60
72
84
96
108
120

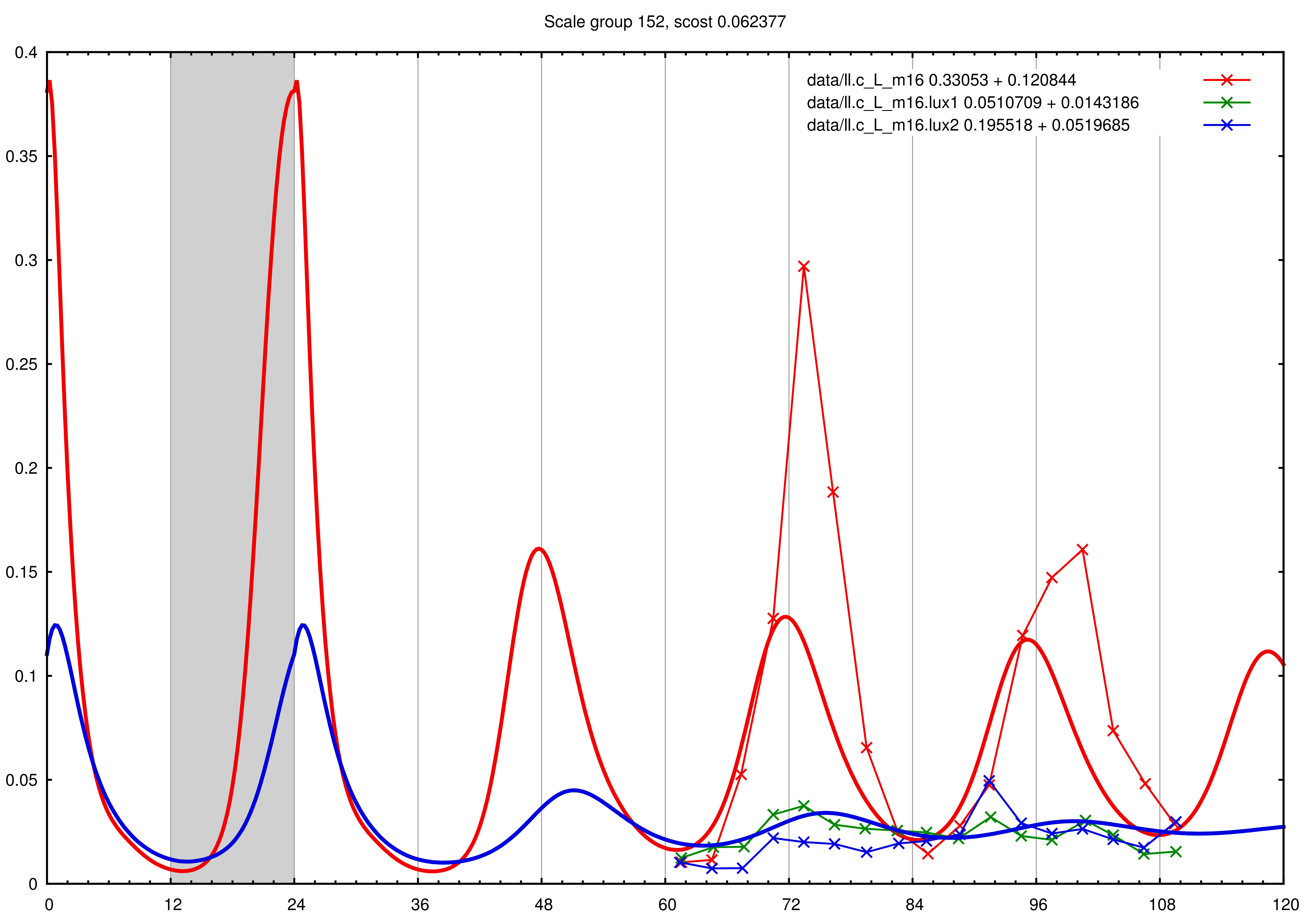

Scale group 152, scost 0.062377
data/ll.c_L_m16 0.33053 + 0.120844
data/ll.c_L_m16.lux1 0.0510709 + 0.0143186
data/ll.c_L_m16.lux2 0.195518 + 0.0519685
0.4
0.35
0.3
0.25
0.2
0.15
0.1
0.05
0
0
12
24
36
48
60
72
84
96
108
120

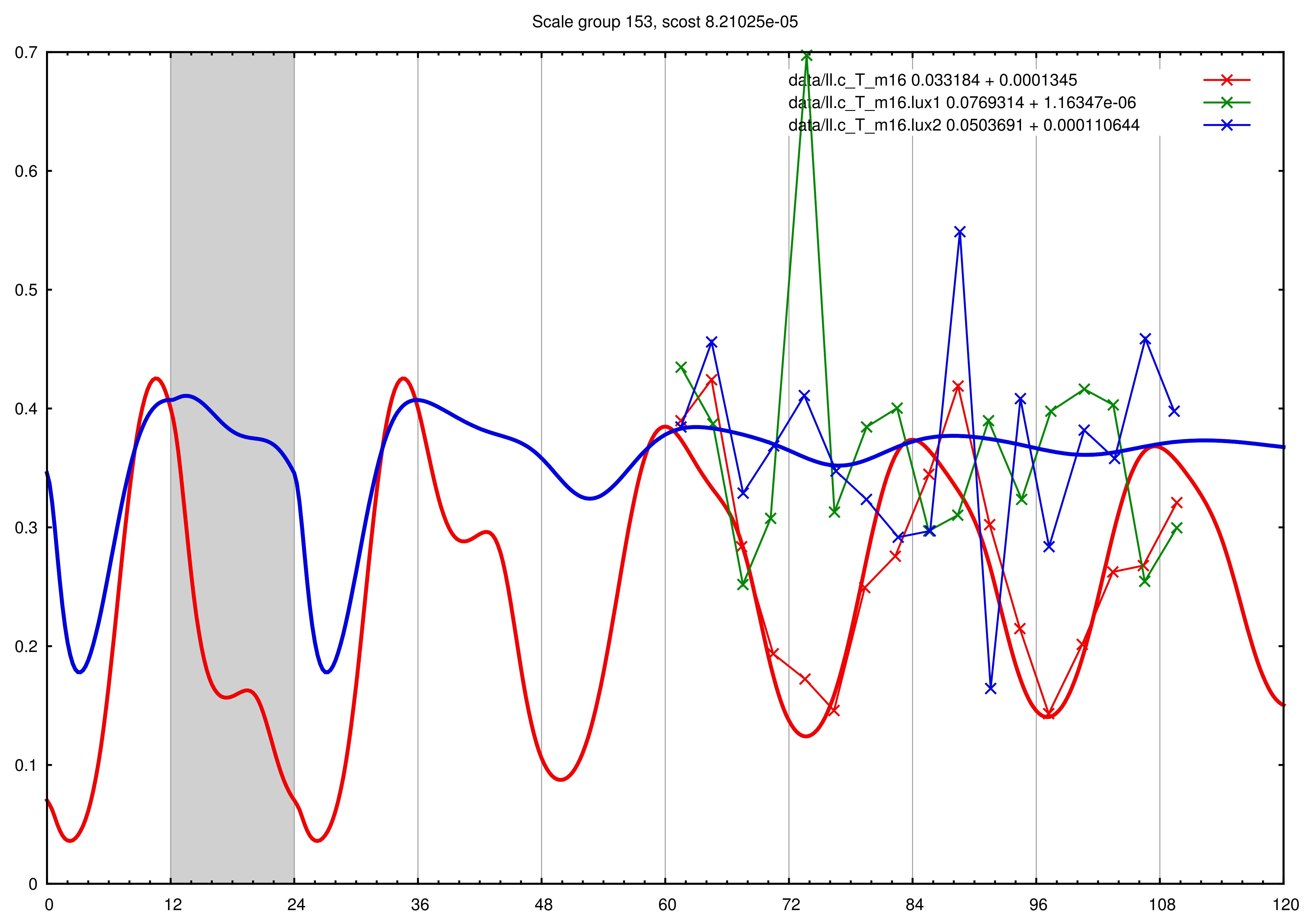
Scale group 153, scost 8.21025e-05
data/ll.c_T_m16 0.033184 + 0.0001345
data/ll.c_T_m16.lux1 0.0769314 + 1.16347e-06
data/ll.c_T_m16.lux2 0.0503691 + 0.000110644
0
0.1
0.2
0.3
0.4
0.5
0.6
0.7
0
12
24
36
48
60
72
84
96
108
120

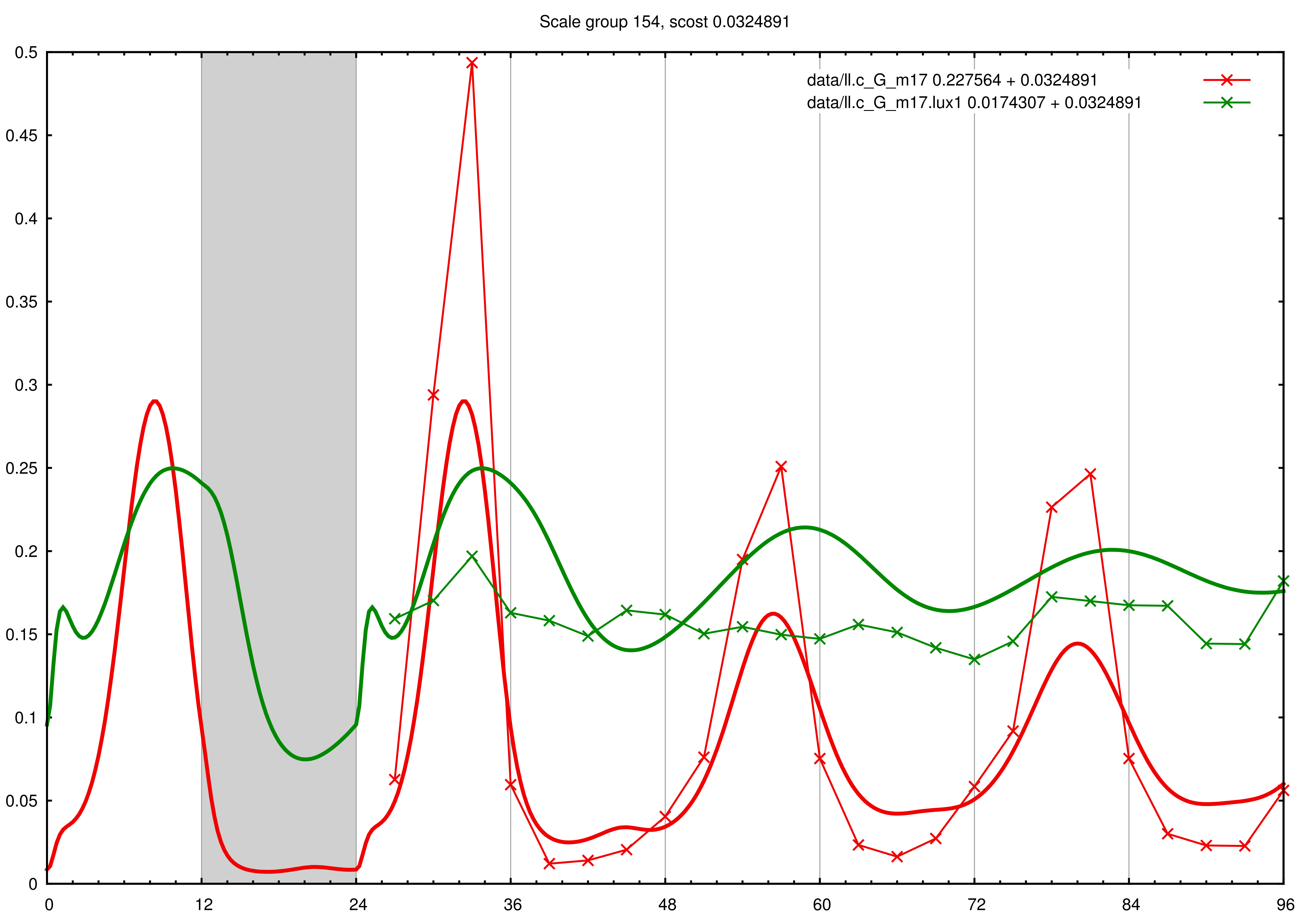
Scale group 154, scost 0.0324891
data/ll.c_G_m17 0.227564 + 0.0324891
data/ll.c_G_m17.lux1 0.0174307 + 0.0324891
0.5
0.45
0.4
0.35
0.3
0.25
0.2
0.15
0.1
0.05
0
0
12
24
36
48
60
72
84
96

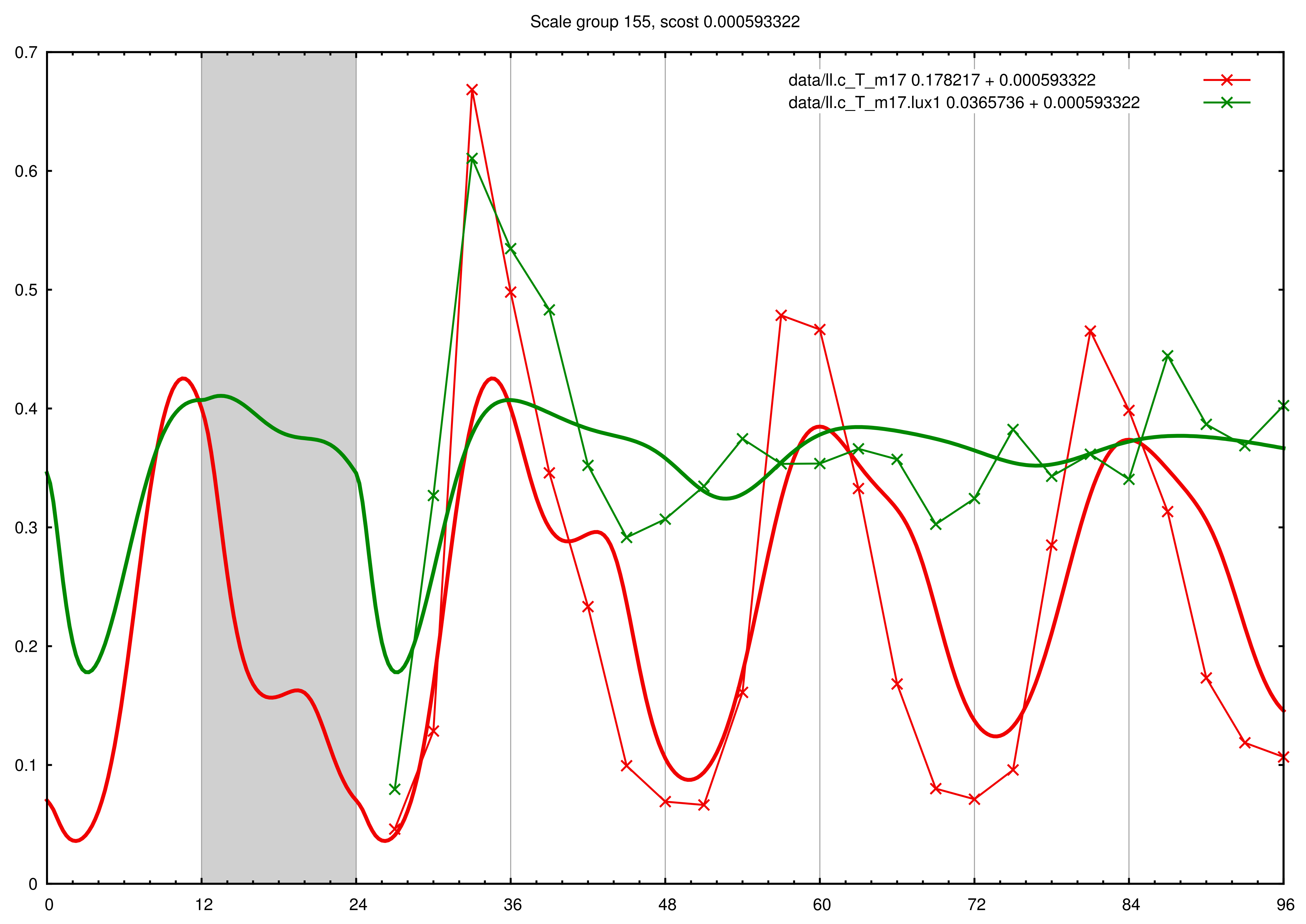

Scale group 155, scost 0.000593322
data/ll.c_T_m17 0.178217 + 0.000593322
data/ll.c_T_m17.lux1 0.0365736 + 0.000593322
0
0.1
0.2
0.3
0.4
0.5
0.6
0.7
0
12
24
36
48
60
72
84
96

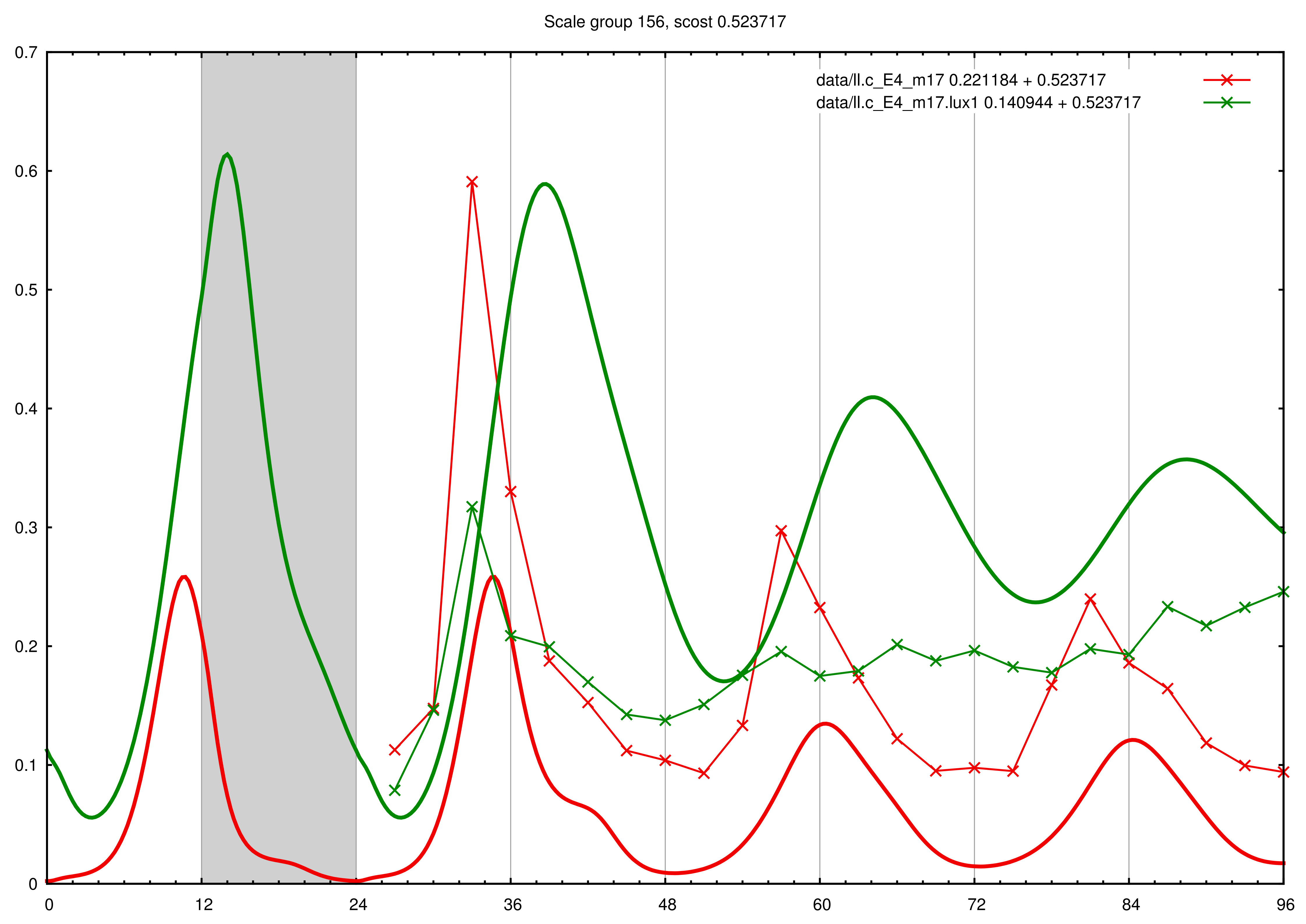

Scale group 156, scost 0.523717
data/ll.c_E4_m17 0.221184 + 0.523717
data/ll.c_E4_m17.lux1 0.140944 + 0.523717
0.7
0.6
0.5
0.4
0.3
0.2
0.1
0
0
12
24
36
48
60
72
84
96

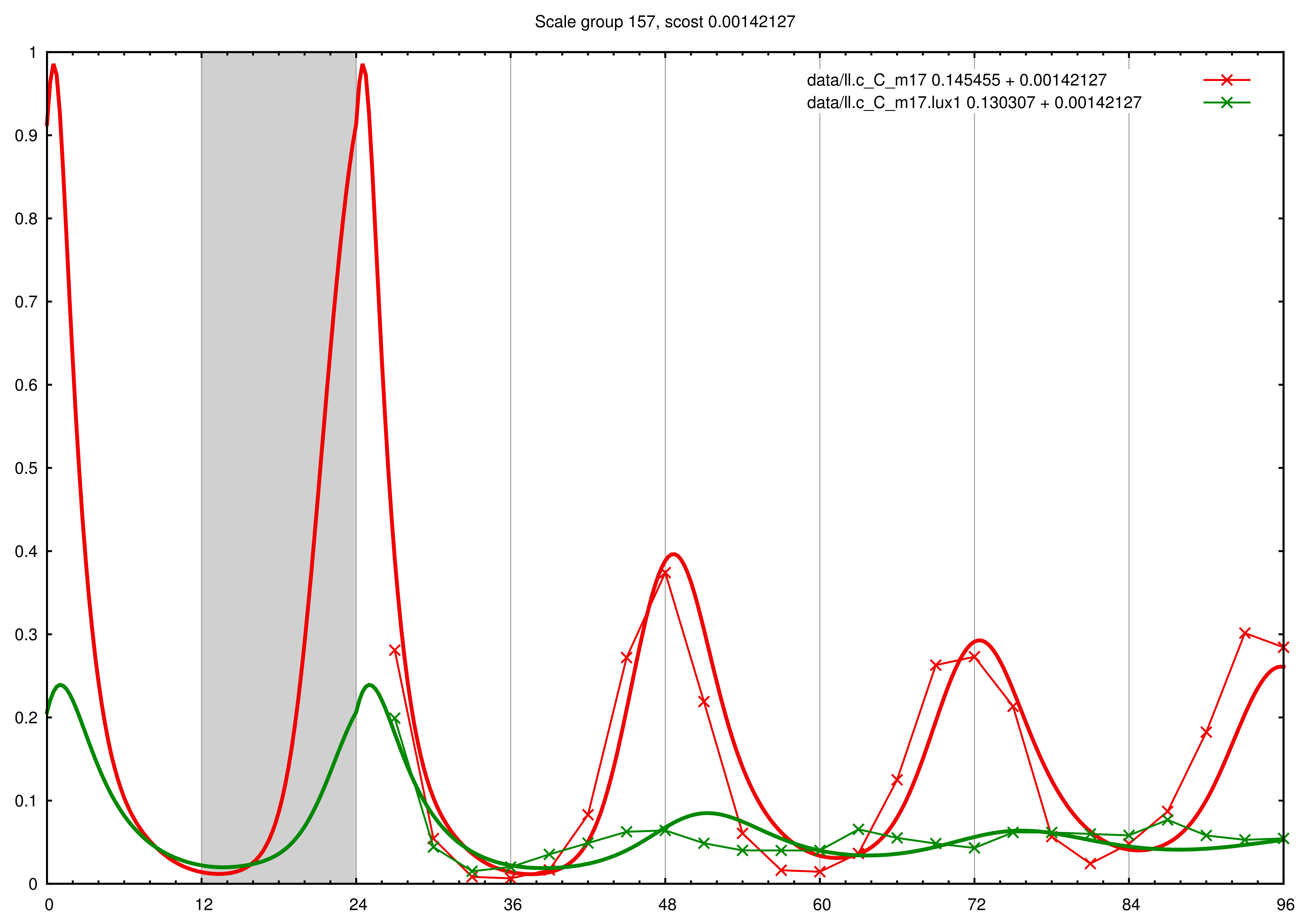
Scale group 157, scost 0.00142127
data/ll.c_C_m17 0.145455 + 0.00142127
data/ll.c_C_m17.lux1 0.130307 + 0.00142127
0
0.1
0.2
0.3
0.4
0.5
0.6
0.7
0.8
0.9
1
0
12
24
36
48
60
72
84
96

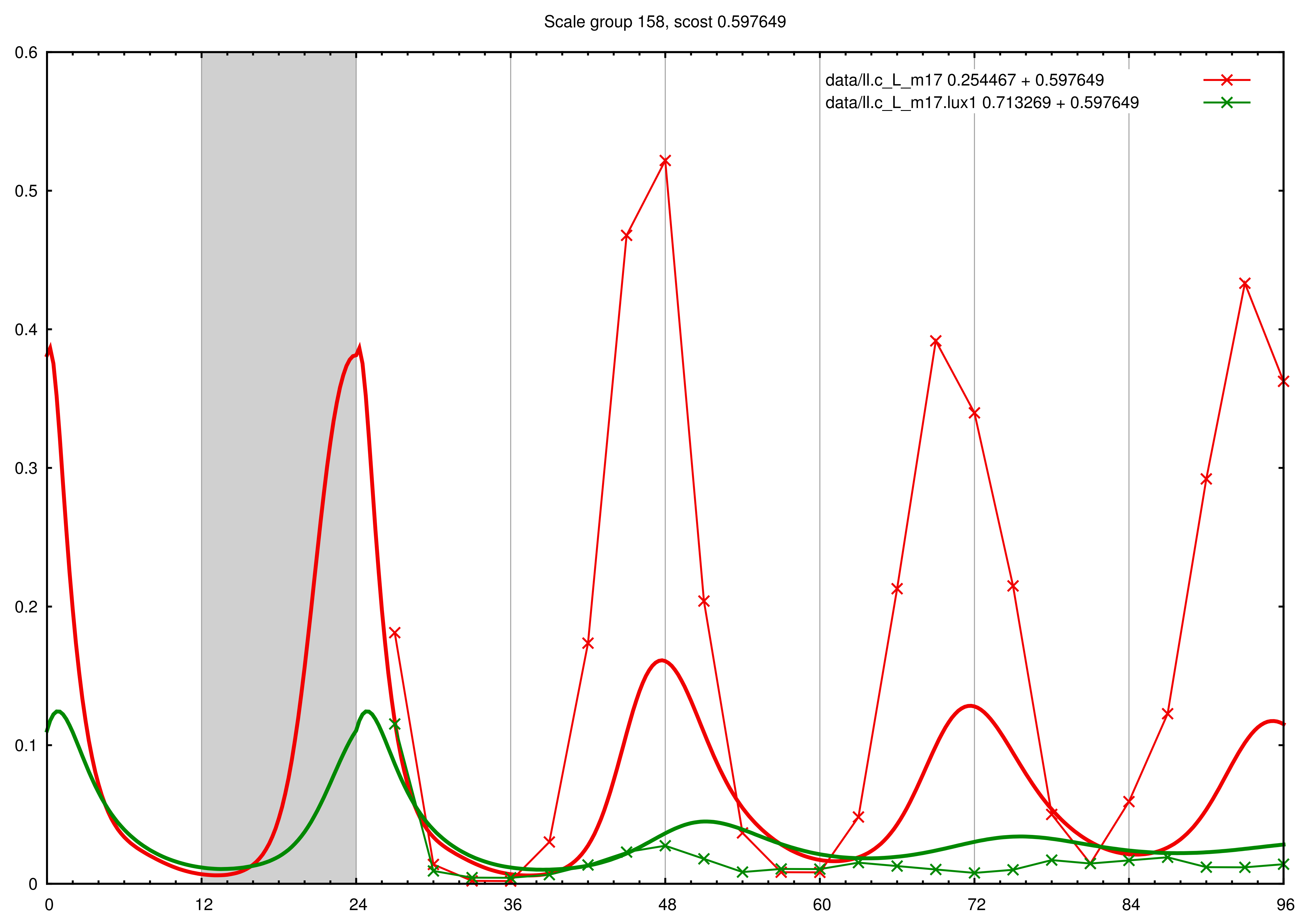

Scale group 158, scost 0.597649
data/ll.c_L_m17 0.254467 + 0.597649
data/ll.c_L_m17.lux1 0.713269 + 0.597649
0.6
0.5
0.4
0.3
0.2
0.1
0
0
12
24
36
48
60
72
84
96

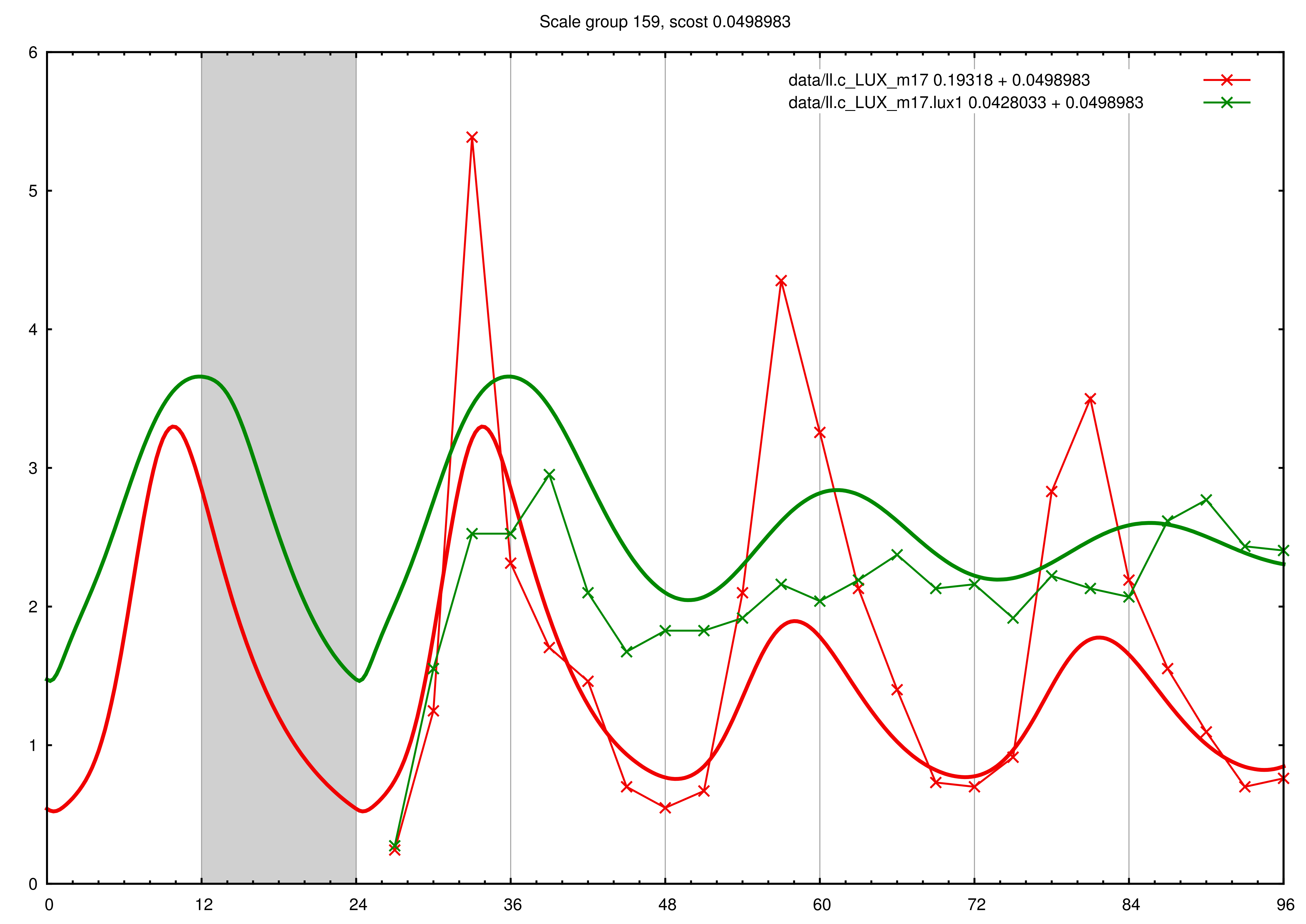

Scale group 159, scost 0.0498983
data/ll.c_LUX_m17 0.19318 + 0.0498983
data/ll.c_LUX_m17.lux1 0.0428033 + 0.0498983
0
1
2
3
4
5
6
0
12
24
36
48
60
72
84
96

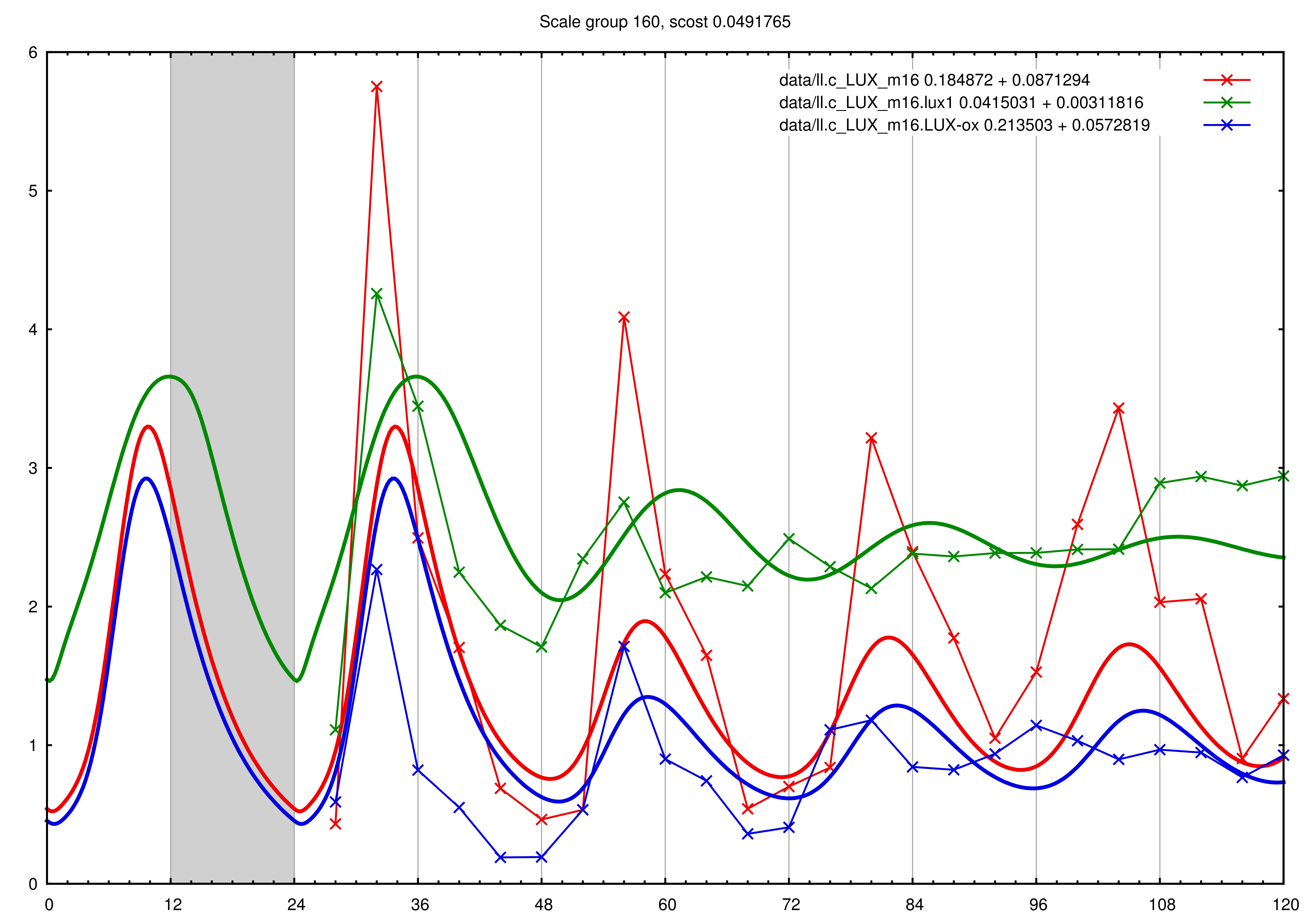

Scale group 160, scost 0.0491765
data/ll.c_LUX_m16 0.184872 + 0.0871294
data/ll.c_LUX_m16.lux1 0.0415031 + 0.00311816
data/ll.c_LUX_m16.LUX-ox 0.213503 + 0.0572819
0
1
2
3
4
5
6
0
12
24
36
48
60
72
84
96
108
120

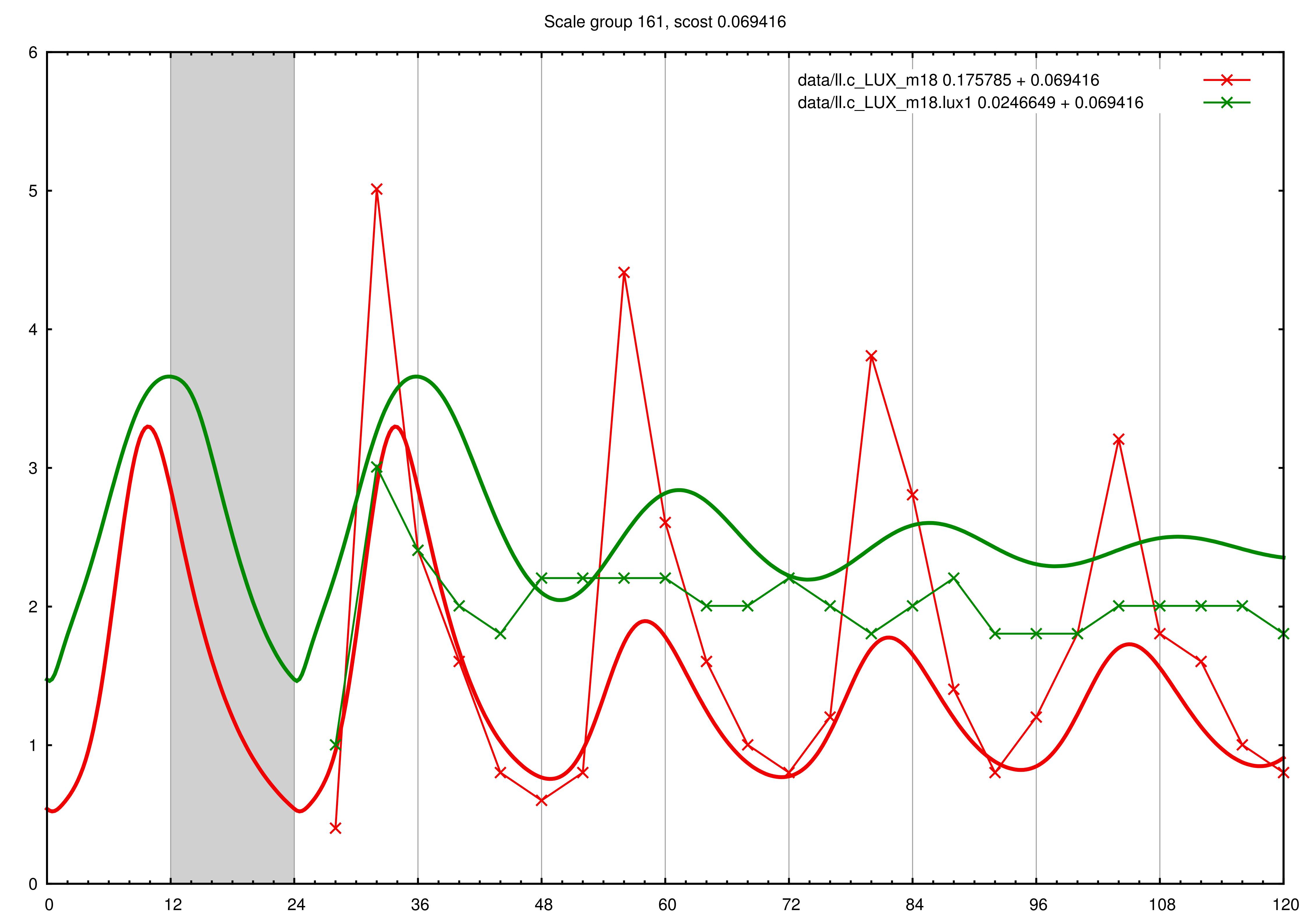

Scale group 161, scost 0.069416
data/ll.c_LUX_m18 0.175785 + 0.069416
data/ll.c_LUX_m18.lux1 0.0246649 + 0.069416
0
1
2
3
4
5
6
0
12
24
36
48
60
72
84
96
108
120

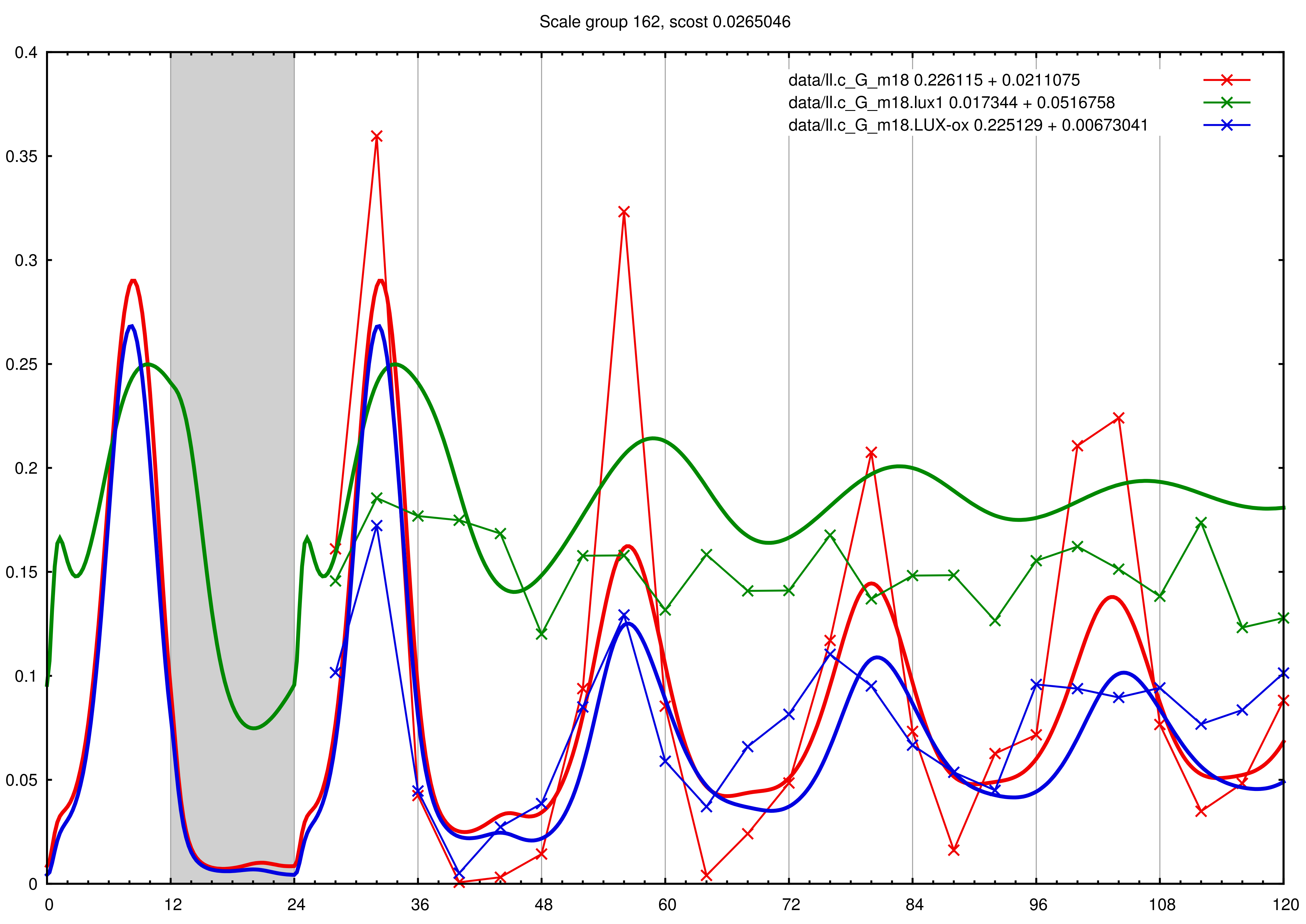
Scale group 162, scost 0.0265046
data/ll.c_G_m18 0.226115 + 0.0211075
data/ll.c_G_m18.lux1 0.017344 + 0.0516758
data/ll.c_G_m18.LUX-ox 0.225129 + 0.00673041
0
0.05
0.1
0.15
0.2
0.25
0.3
0.35
0.4
0
12
24
36
48
60
72
84
96
108
120

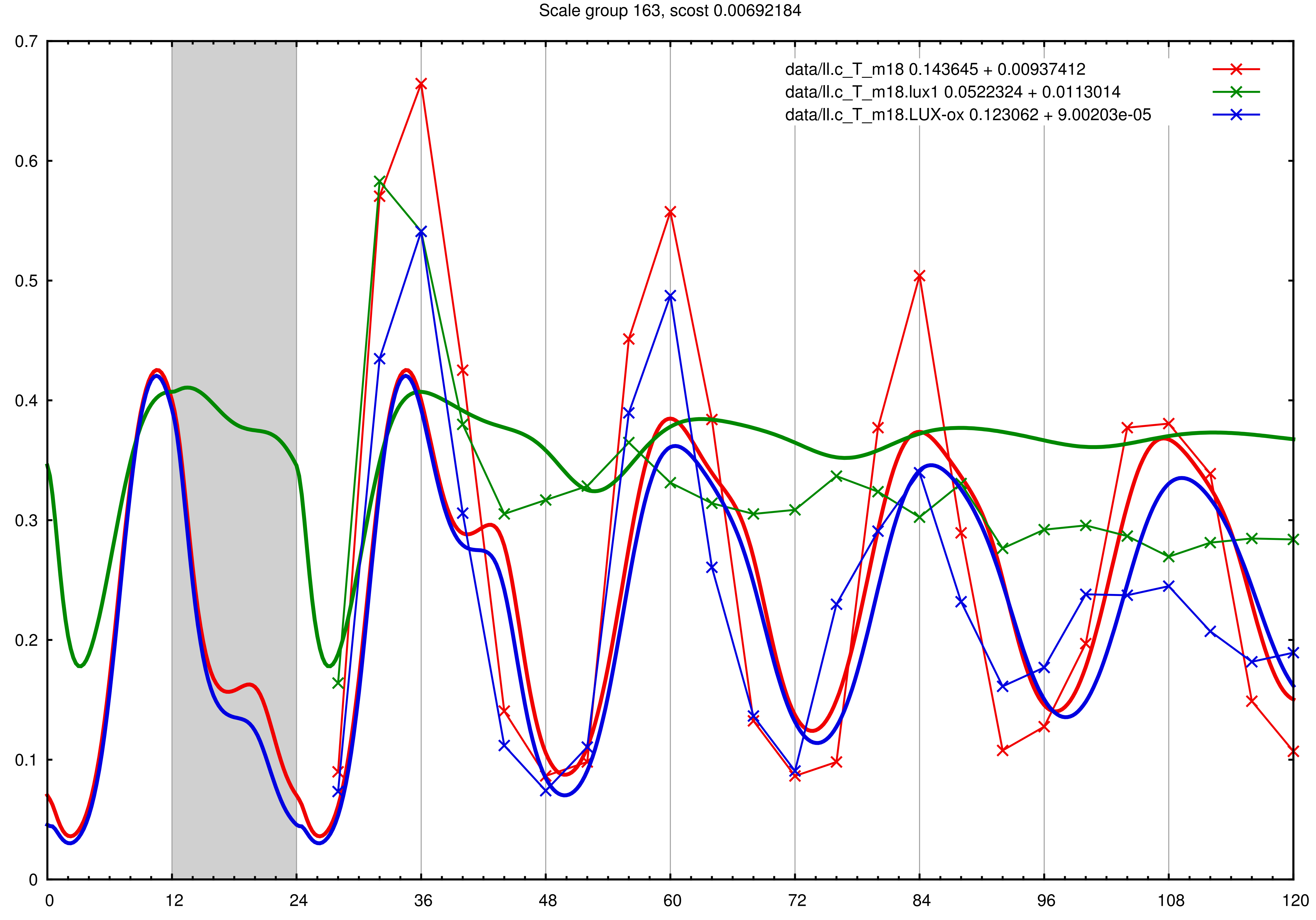

Scale group 163, scost 0.00692184
data/ll.c_T_m18 0.143645 + 0.00937412
data/ll.c_T_m18.lux1 0.0522324 + 0.0113014
data/ll.c_T_m18.LUX-ox 0.123062 + 9.00203e-05
0
0.1
0.2
0.3
0.4
0.5
0.6
0.7
0
12
24
36
48
60
72
84
96
108
120

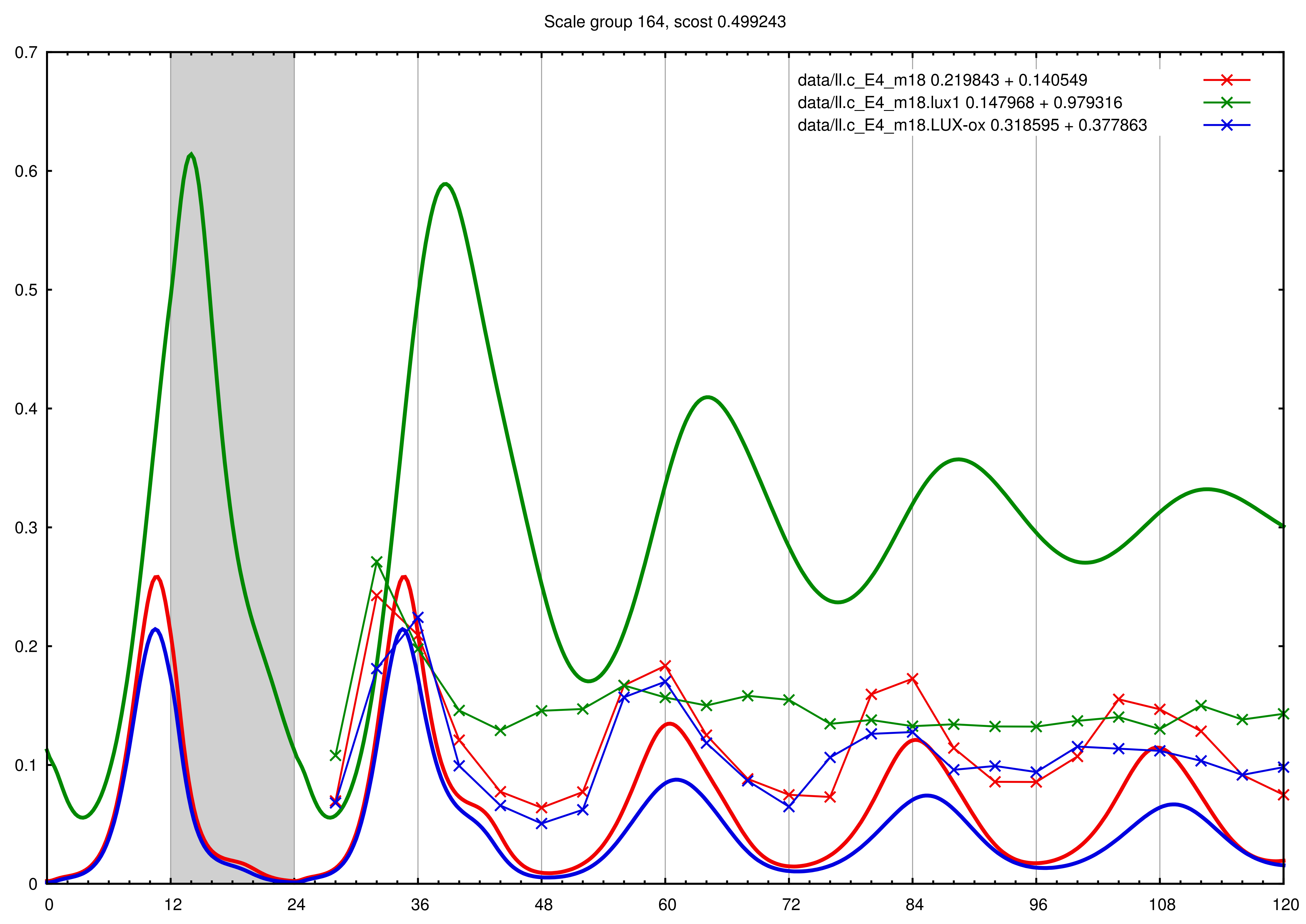

Scale group 164, scost 0.499243
data/ll.c_E4_m18 0.219843 + 0.140549
data/ll.c_E4_m18.lux1 0.147968 + 0.979316
data/ll.c_E4_m18.LUX-ox 0.318595 + 0.377863
0
0.1
0.2
0.3
0.4
0.5
0.6
0.7
0
12
24
36
48
60
72
84
96
108
120

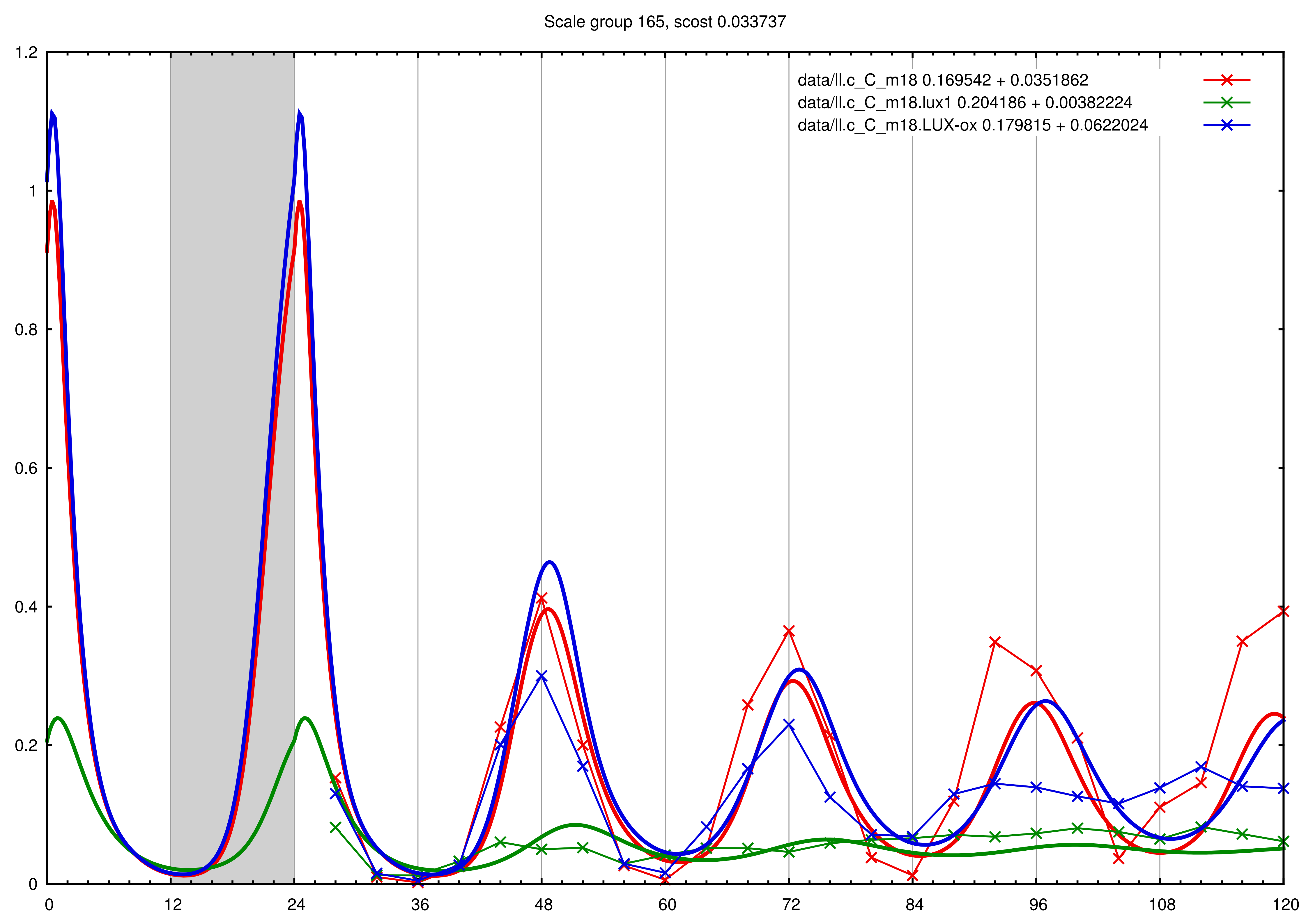

Scale group 165, scost 0.033737
data/ll.c_C_m18 0.169542 + 0.0351862
data/ll.c_C_m18.lux1 0.204186 + 0.00382224
data/ll.c_C_m18.LUX-ox 0.179815 + 0.0622024
1.2
1
0.8
0.6
0.4
0.2
0
0
12
24
36
48
60
72
84
96
108
120

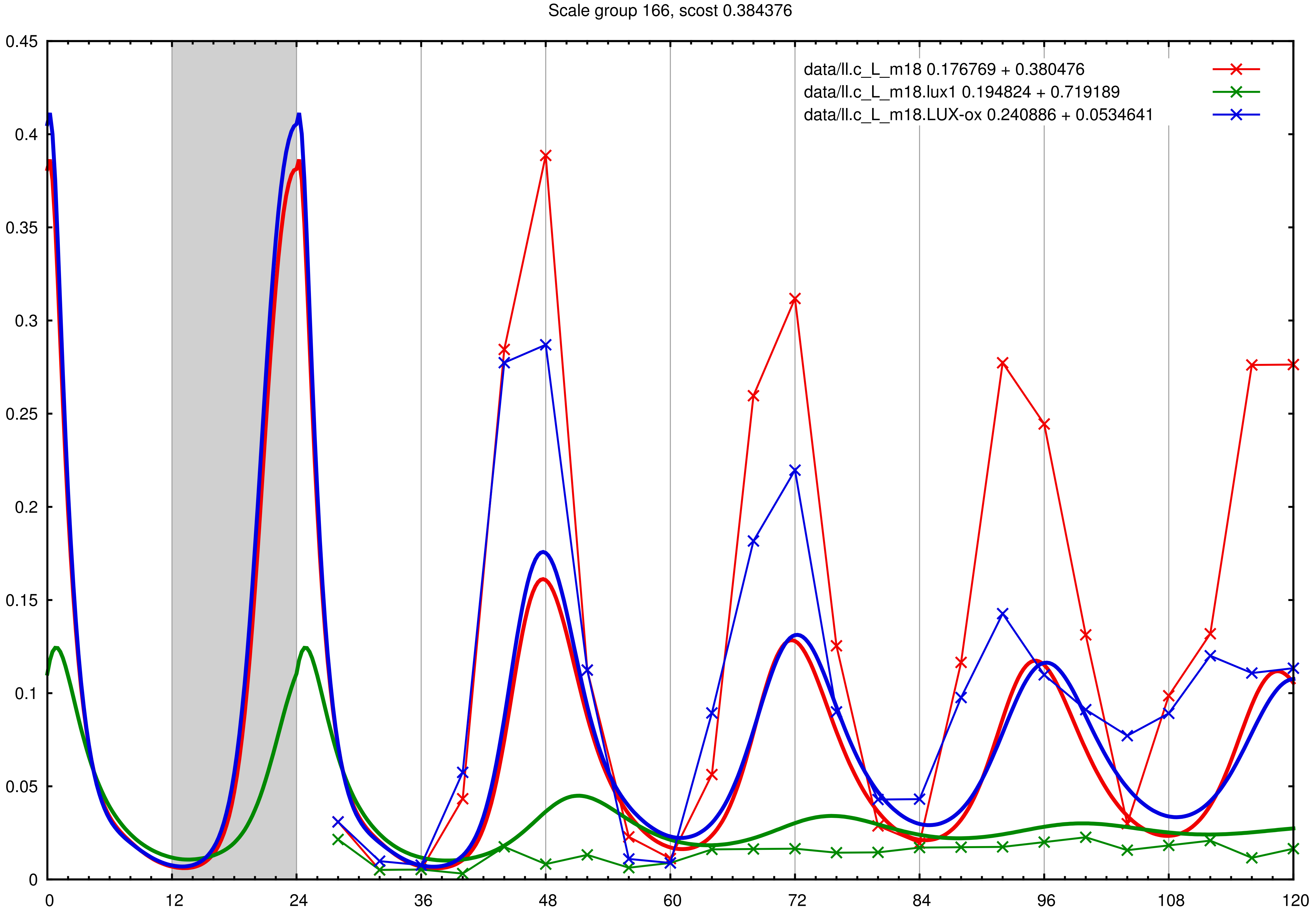

Scale group 166, scost 0.384376
data/ll.c_L_m18 0.176769 + 0.380476
data/ll.c_L_m18.lux1 0.194824 + 0.719189
data/ll.c_L_m18.LUX-ox 0.240886 + 0.0534641
0.45
0.4
0.35
0.3
0.25
0.2
0.15
0.1
0.05
0
0
12
24
36
48
60
72
84
96
108
120

Scale group 167, scost 0.028789

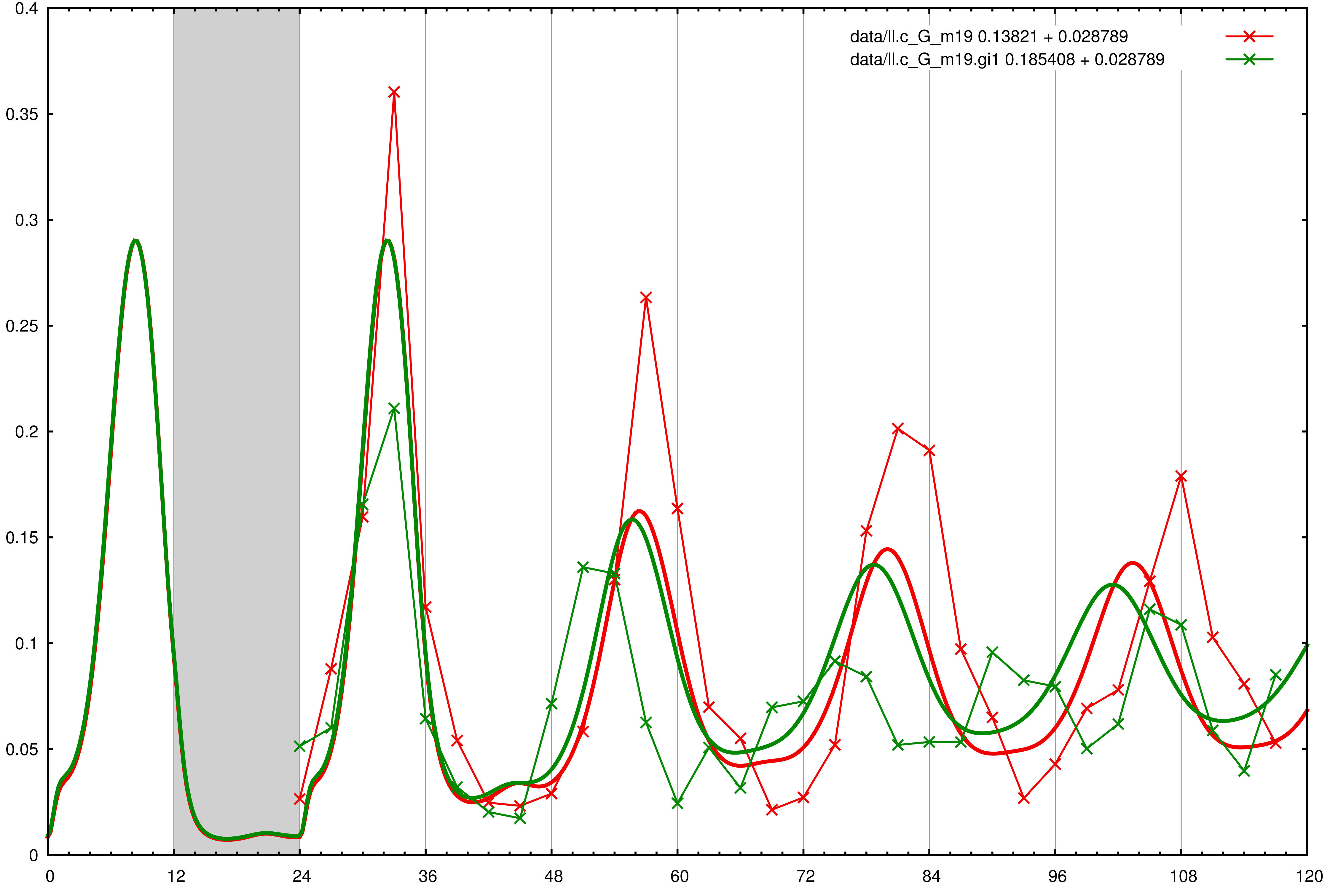

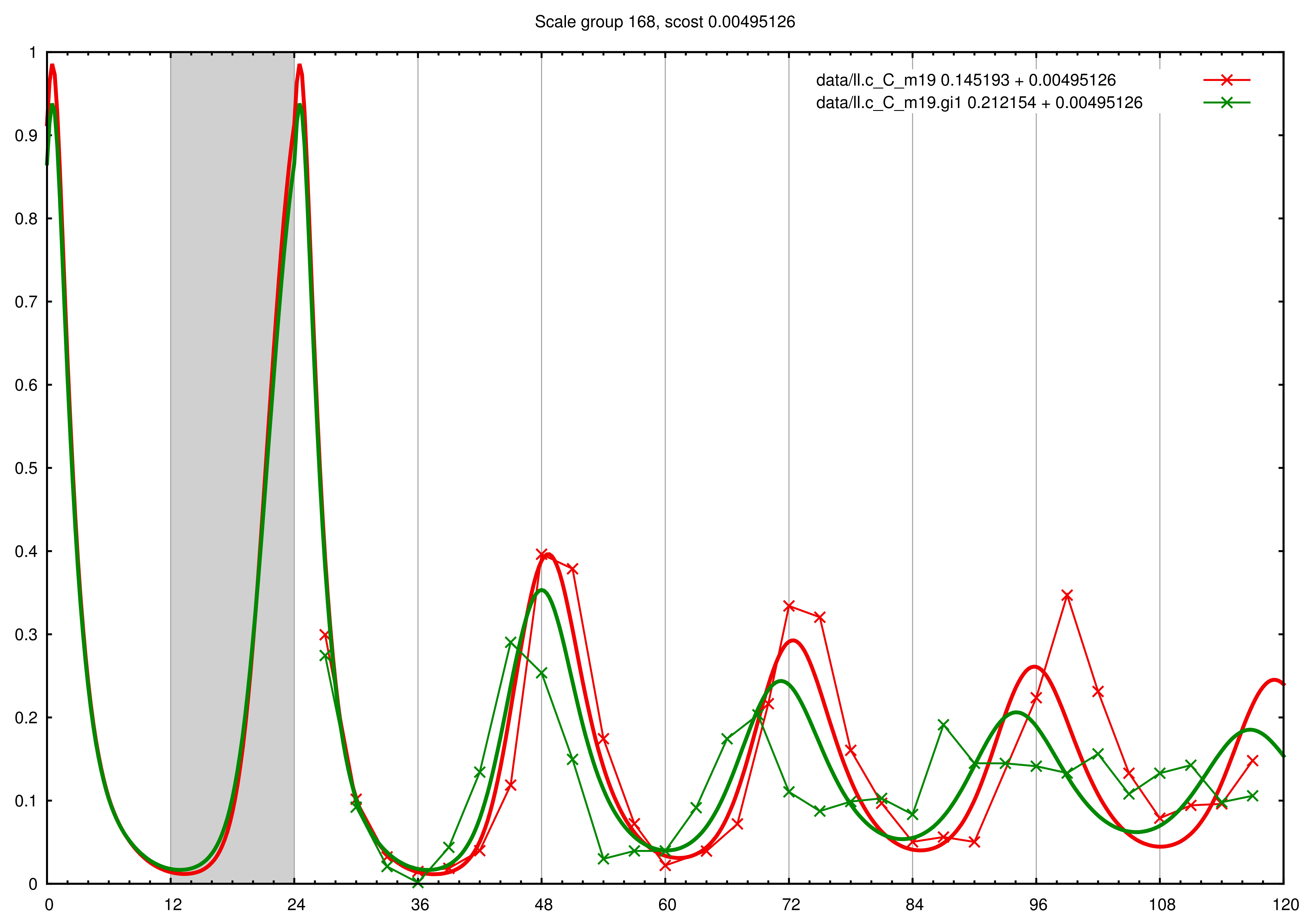

Scale group 168, scost 0.00495126
data/ll.c_C_m19 0.145193 + 0.00495126
data/ll.c_C_m19.gi1 0.212154 + 0.00495126
1
0.9
0.8
0.7
0.6
0.5
0.4
0.3
0.2
0.1
0
0
12
24
36
48
60
72
84
96
108
120

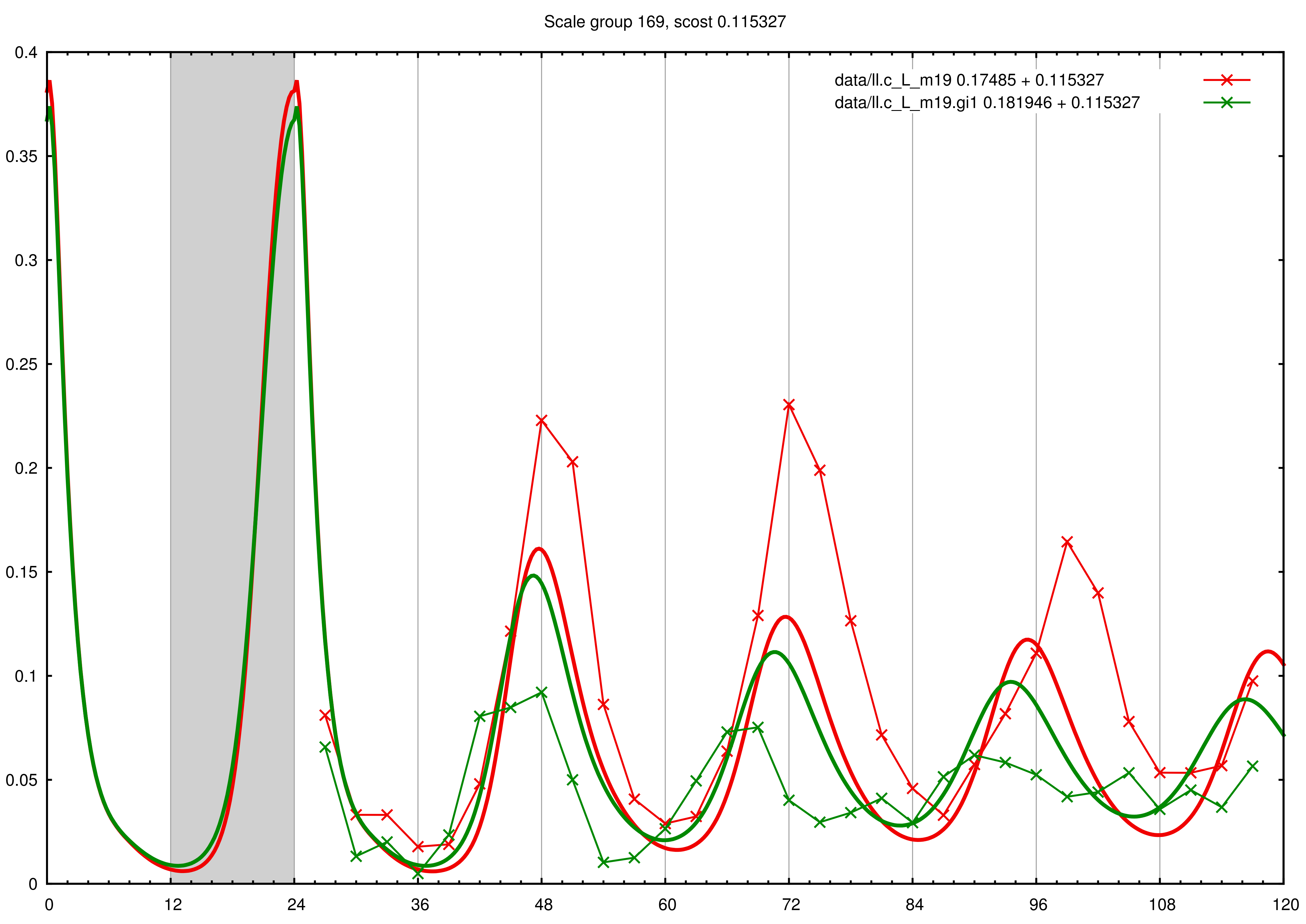

Scale group 169, scost 0.115327
data/ll.c_L_m19 0.17485 + 0.115327
data/ll.c_L_m19.gi1 0.181946 + 0.115327
0.4
0.35
0.3
0.25
0.2
0.15
0.1
0.05
0
0
12
24
36
48
60
72
84
96
108
120

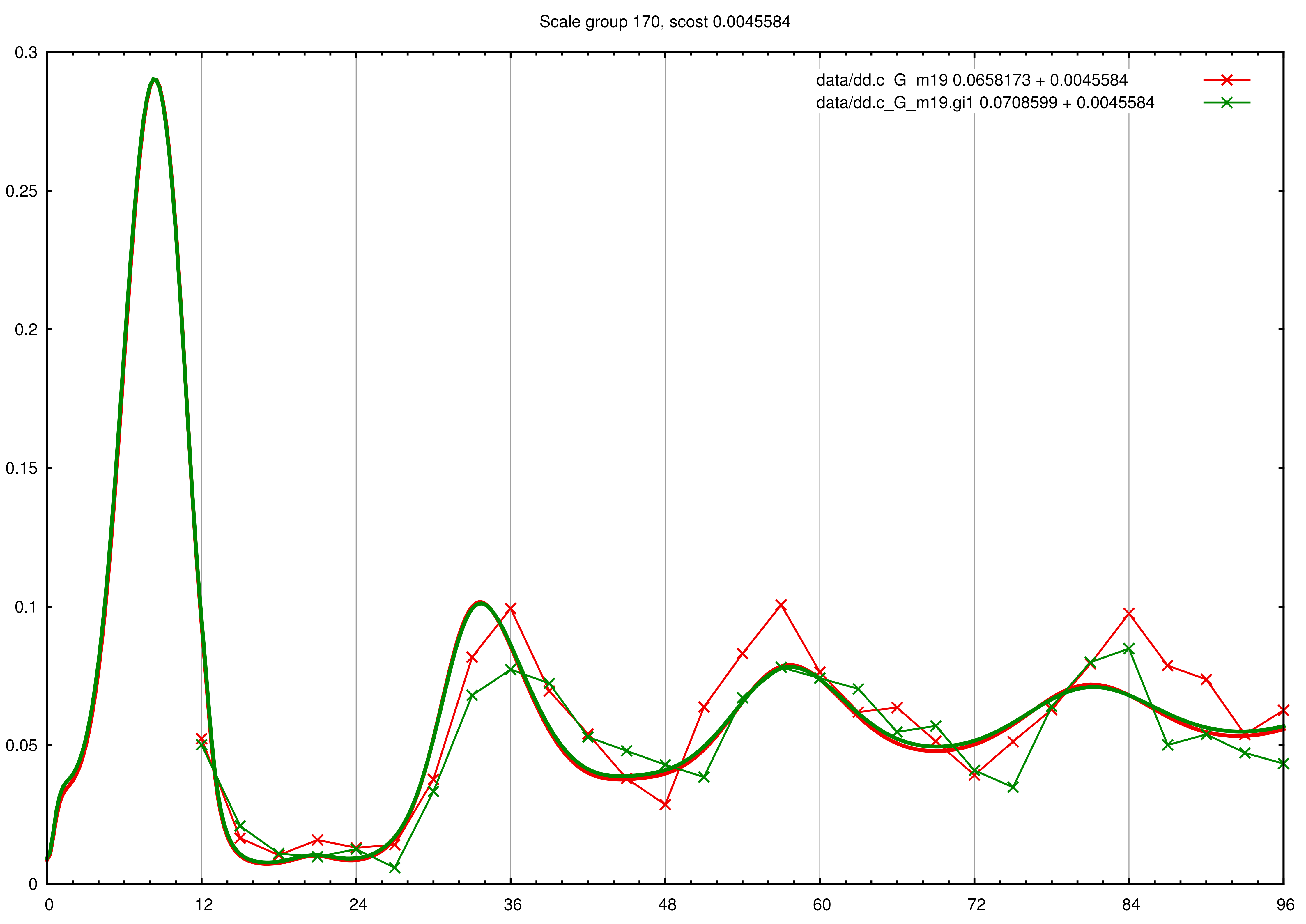

Scale group 170, scost 0.0045584
data/dd.c_G_m19 0.0658173 + 0.0045584
data/dd.c_G_m19.gi1 0.0708599 + 0.0045584
0.3
0.25
0.2
0.15
0.1
0.05
0
0
12
24
36
48
60
72
84
96

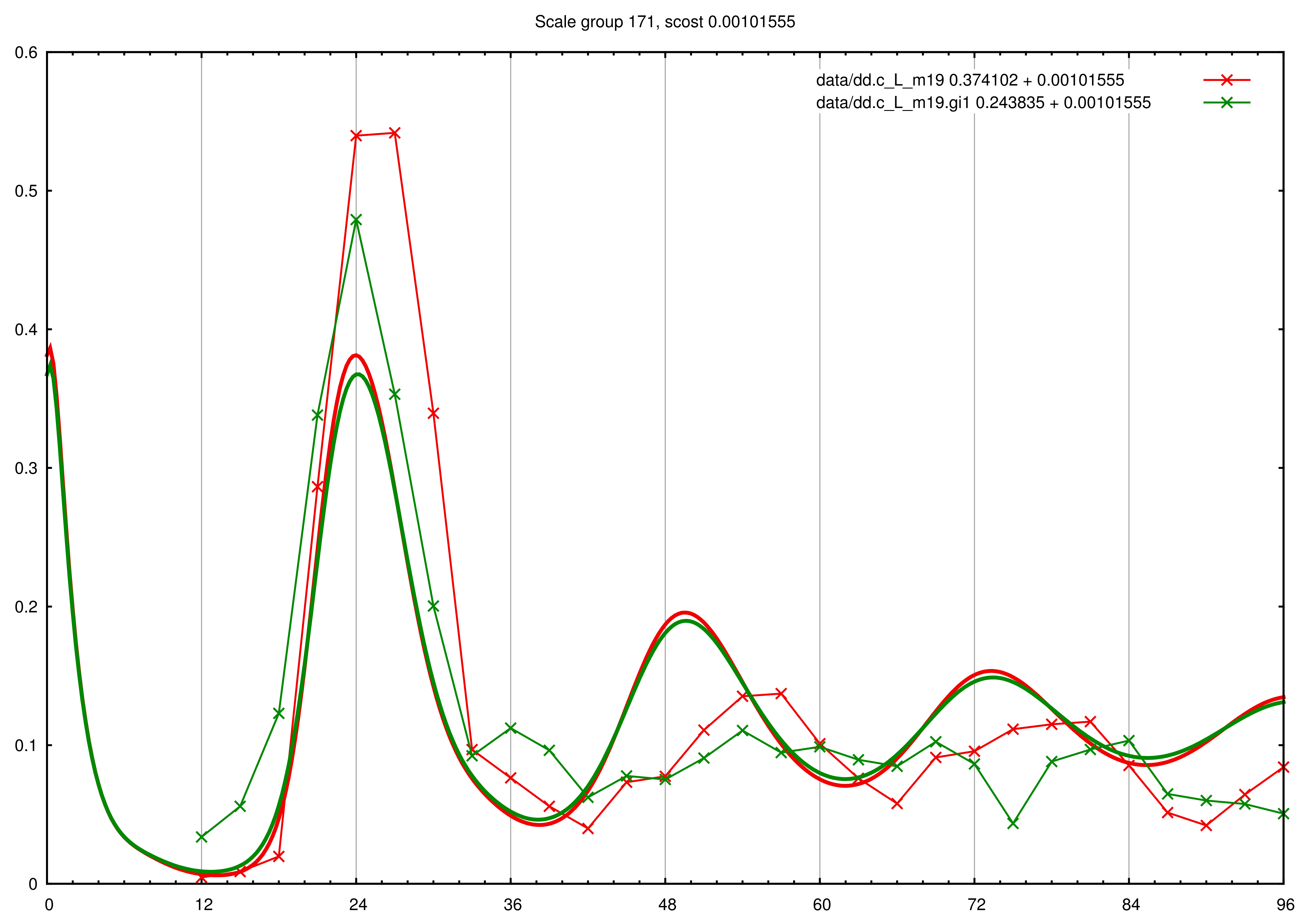

Scale group 171, scost 0.00101555
data/dd.c_L_m19 0.374102 + 0.00101555
data/dd.c_L_m19.gi1 0.243835 + 0.00101555
0.6
0.5
0.4
0.3
0.2
0.1
0
0
12
24
36
48
60
72
84
96

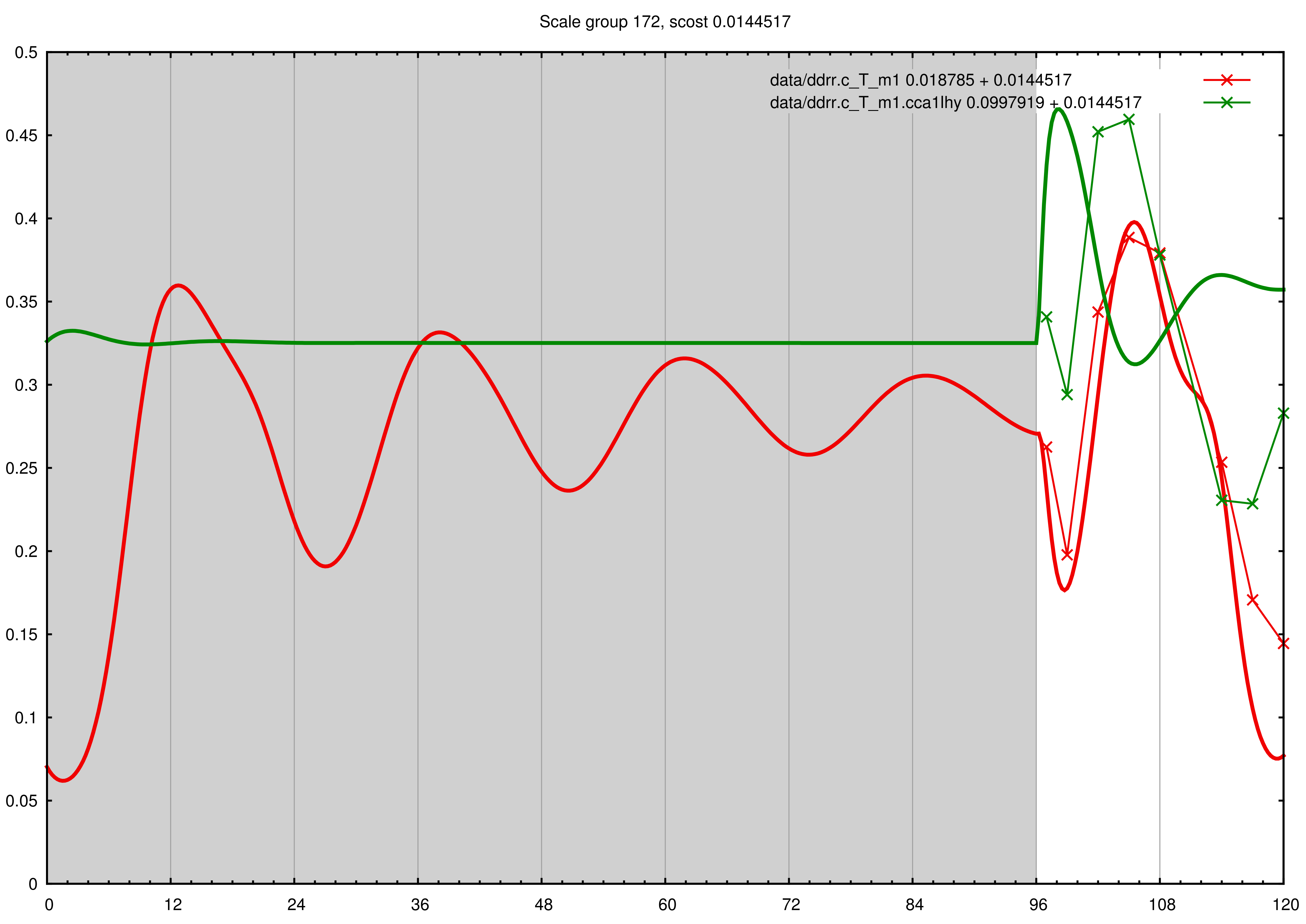
Scale group 172, scost 0.0144517
data/ddrr.c_T_m1 0.018785 + 0.0144517
data/ddrr.c_T_m1.cca1lhy 0.0997919 + 0.0144517
0.5
0.45
0.4
0.35
0.3
0.25
0.2
0.15
0.1
0.05
0
0
12
24
36
48
60
72
84
96
108
120

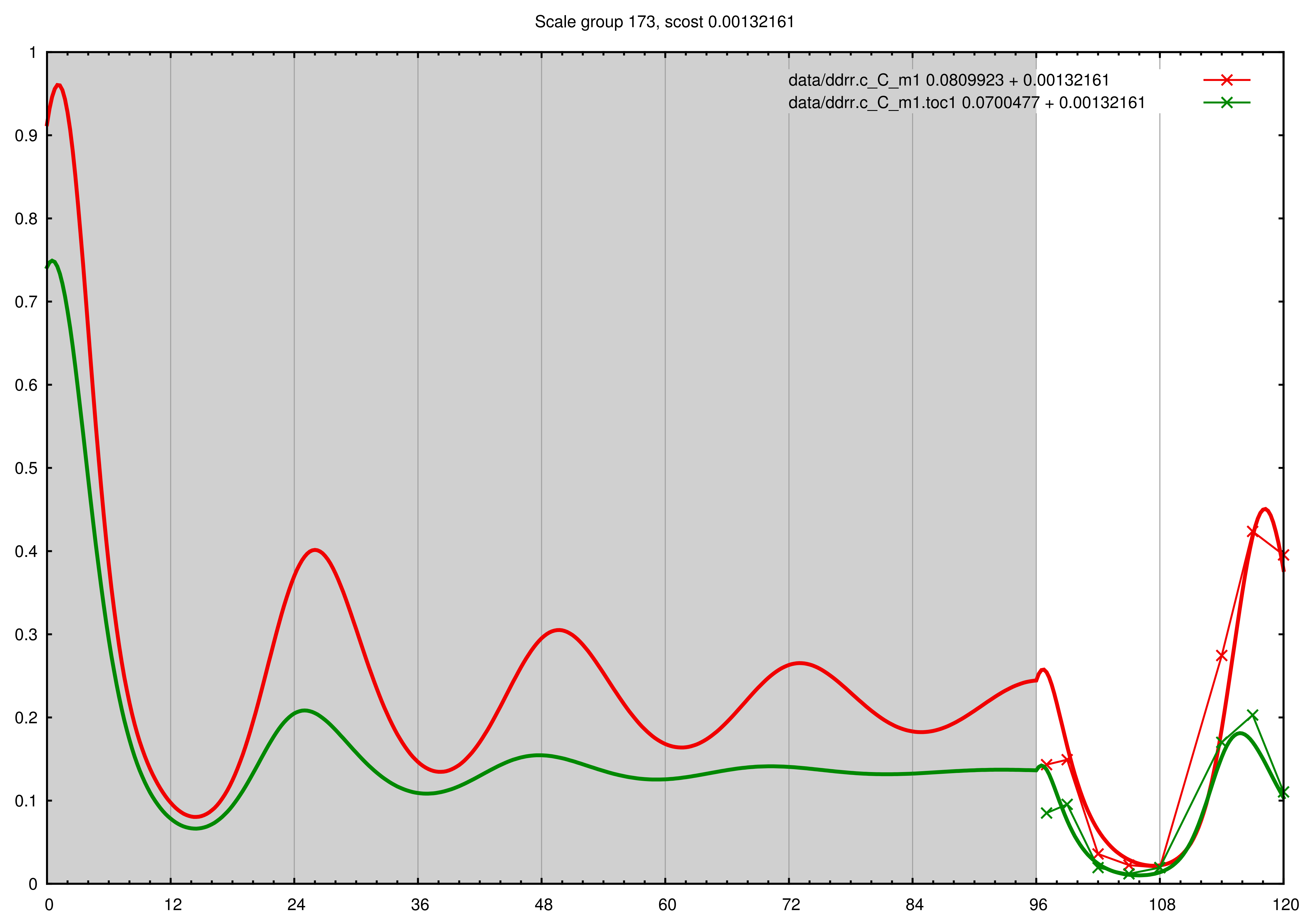

Scale group 173, scost 0.00132161
data/ddrr.c_C_m1 0.0809923 + 0.00132161
data/ddrr.c_C_m1.toc1 0.0700477 + 0.00132161
1
0.9
0.8
0.7
0.6
0.5
0.4
0.3
0.2
0.1
0
0
12
24
36
48
60
72
84
96
108
120

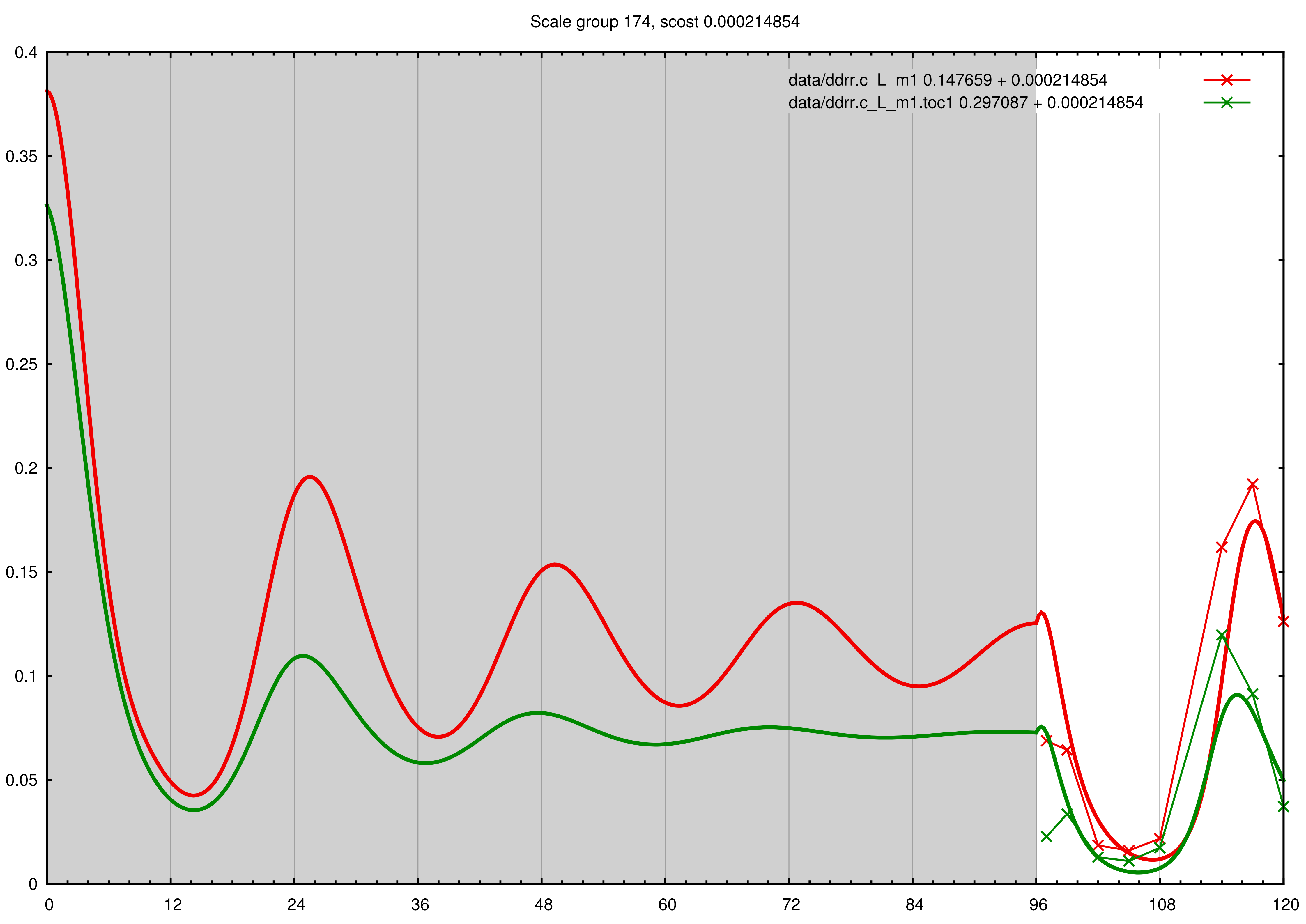

Scale group 174, scost 0.000214854
data/ddrr.c_L_m1 0.147659 + 0.000214854
data/ddrr.c_L_m1.toc1 0.297087 + 0.000214854
0.4
0.35
0.3
0.25
0.2
0.15
0.1
0.05
0
0
12
24
36
48
60
72
84
96
108
120

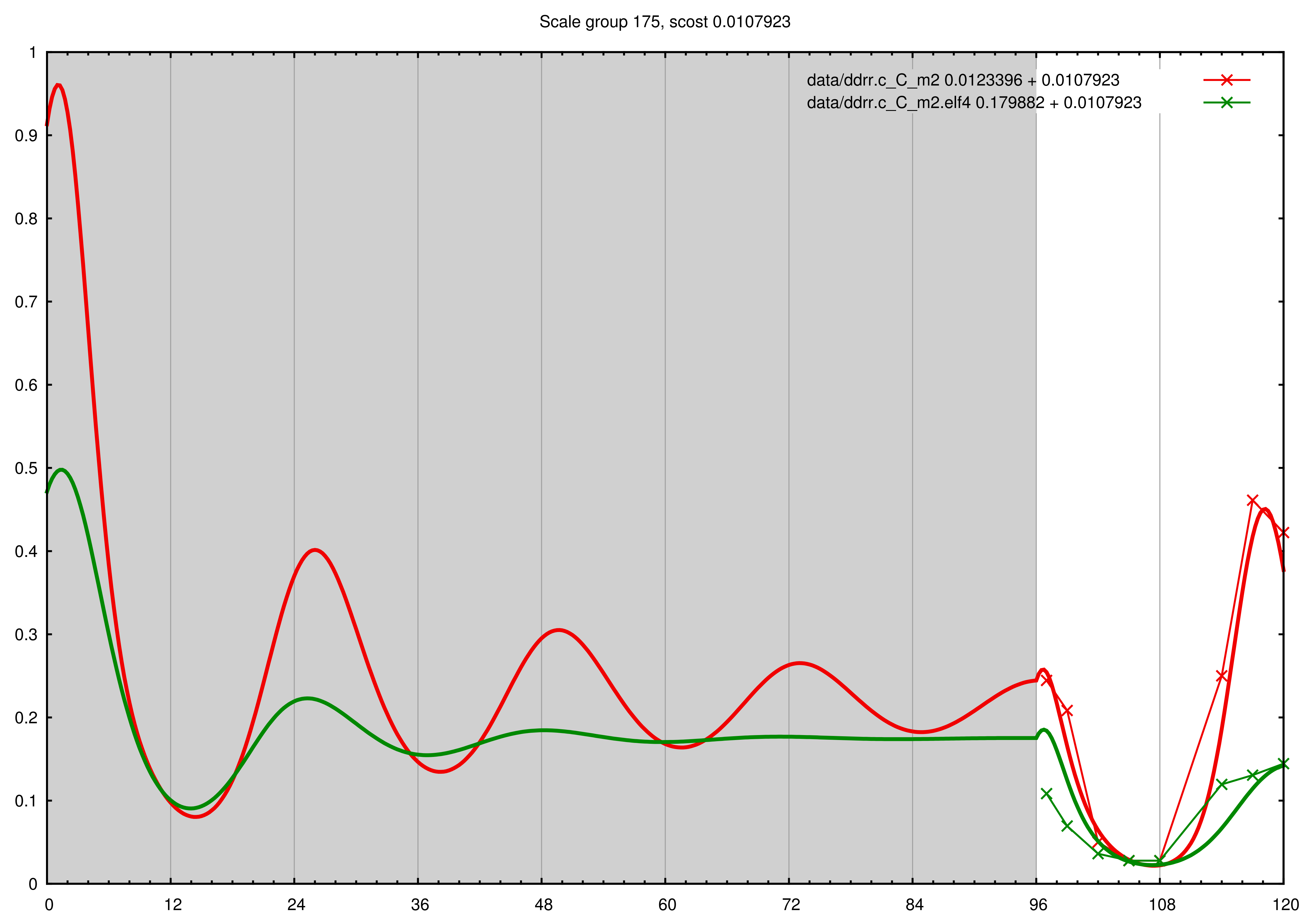
Scale group 175, scost 0.0107923
data/ddrr.c_C_m2 0.0123396 + 0.0107923
data/ddrr.c_C_m2.elf4 0.179882 + 0.0107923
0
0.1
0.2
0.3
0.4
0.5
0.6
0.7
0.8
0.9
1
0
12
24
36
48
60
72
84
96
108
120

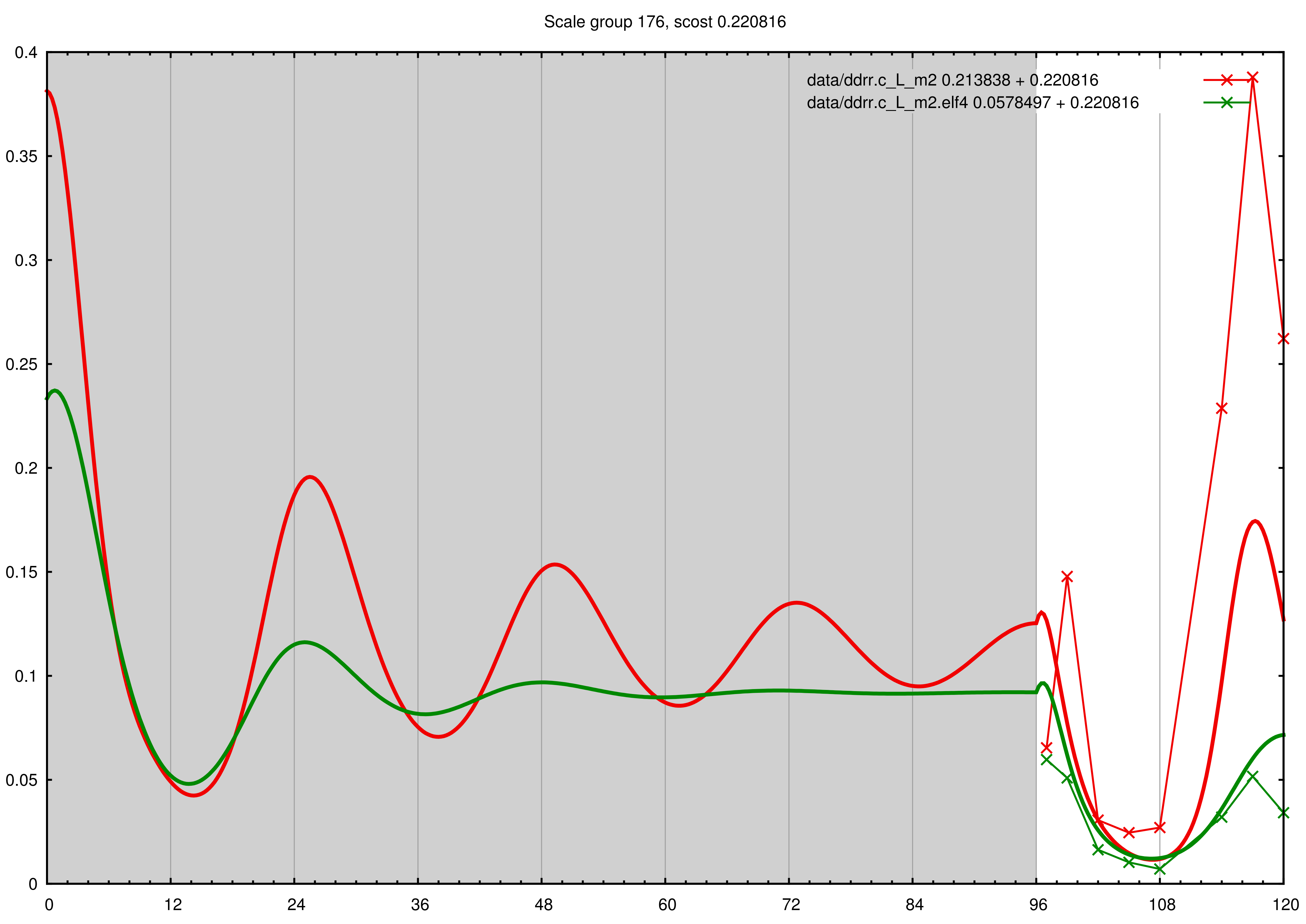
Scale group 176, scost 0.220816
data/ddrr.c_L_m2 0.213838 + 0.220816
data/ddrr.c_L_m2.elf4 0.0578497 + 0.220816
0.4
0.35
0.3
0.25
0.2
0.15
0.1
0.05
0
0
12
24
36
48
60
72
84
96
108
120

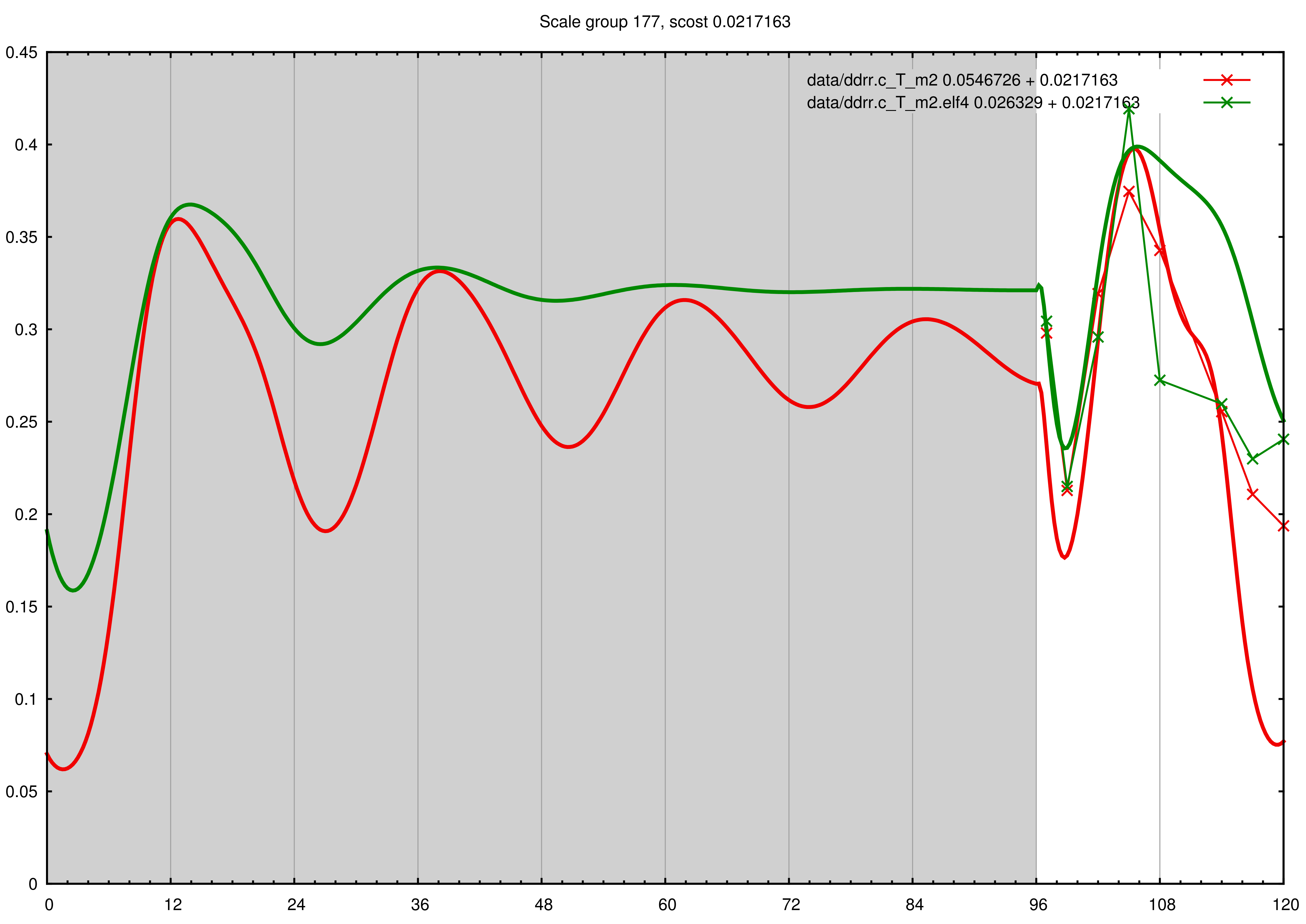

Scale group 177, scost 0.0217163
data/ddrr.c_T_m2 0.0546726 + 0.0217163
data/ddrr.c_T_m2.elf4 0.026329 + 0.0217163
0.45
0.4
0.35
0.3
0.25
0.2
0.15
0.1
0.05
0
0
12
24
36
48
60
72
84
96
108
120

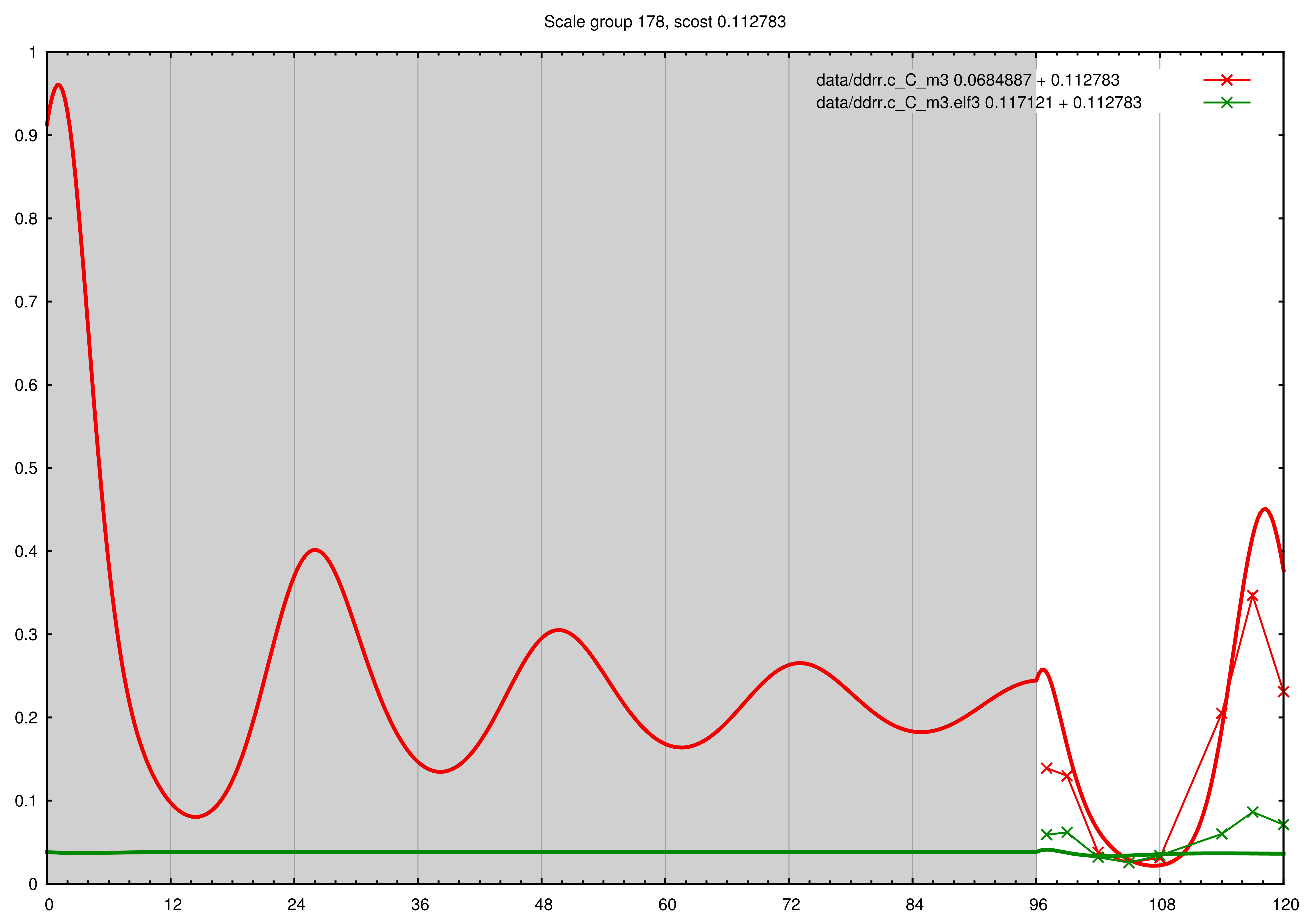

Scale group 178, scost 0.112783
data/ddrr.c_C_m3 0.0684887 + 0.112783
data/ddrr.c_C_m3.elf3 0.117121 + 0.112783
1
0.9
0.8
0.7
0.6
0.5
0.4
0.3
0.2
0.1
0
0
12
24
36
48
60
72
84
96
108
120

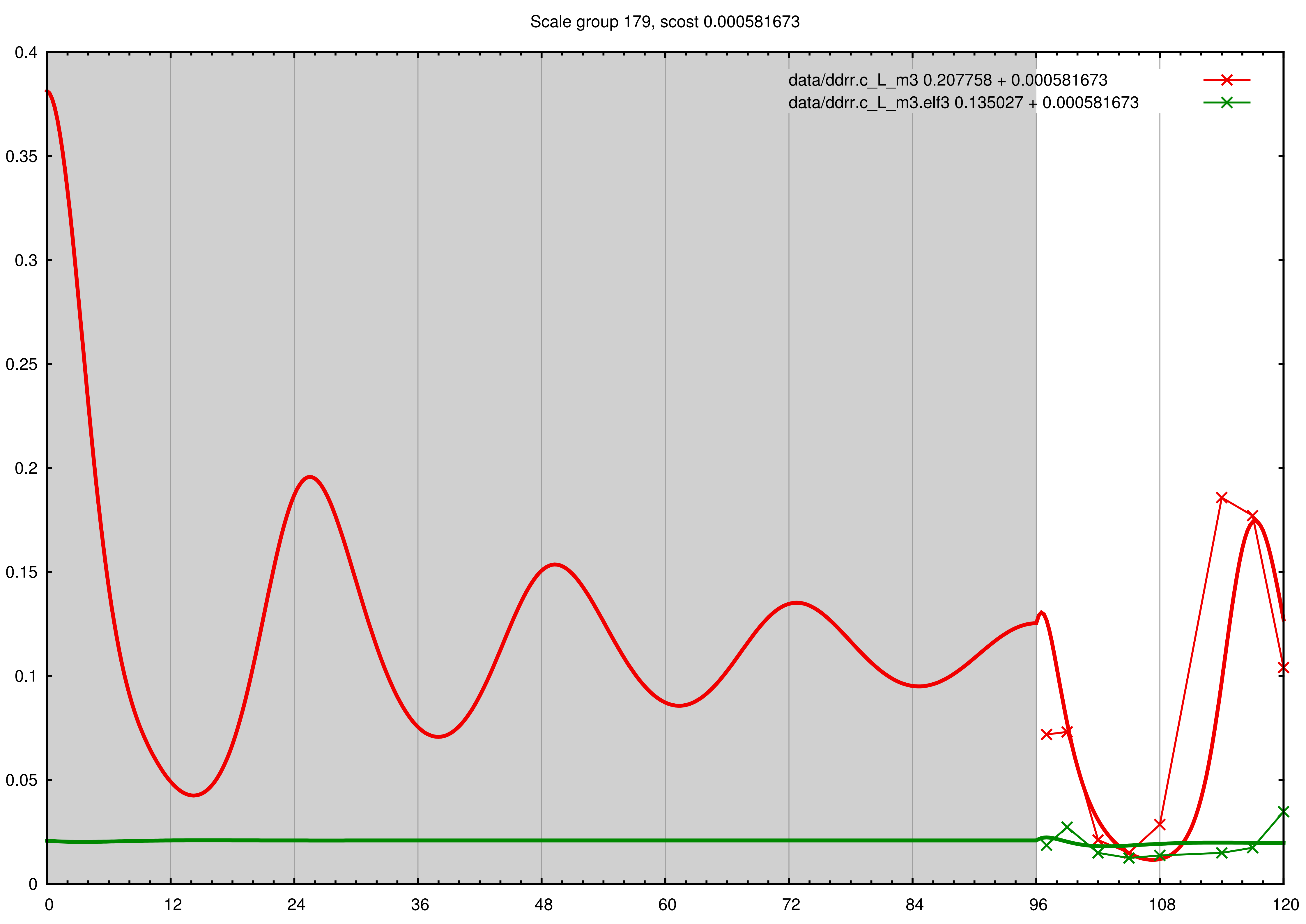

Scale group 179, scost 0.000581673
data/ddrr.c_L_m3 0.207758 + 0.000581673
data/ddrr.c_L_m3.elf3 0.135027 + 0.000581673
0.4
0.35
0.3
0.25
0.2
0.15
0.1
0.05
0
0
12
24
36
48
60
72
84
96
108
120

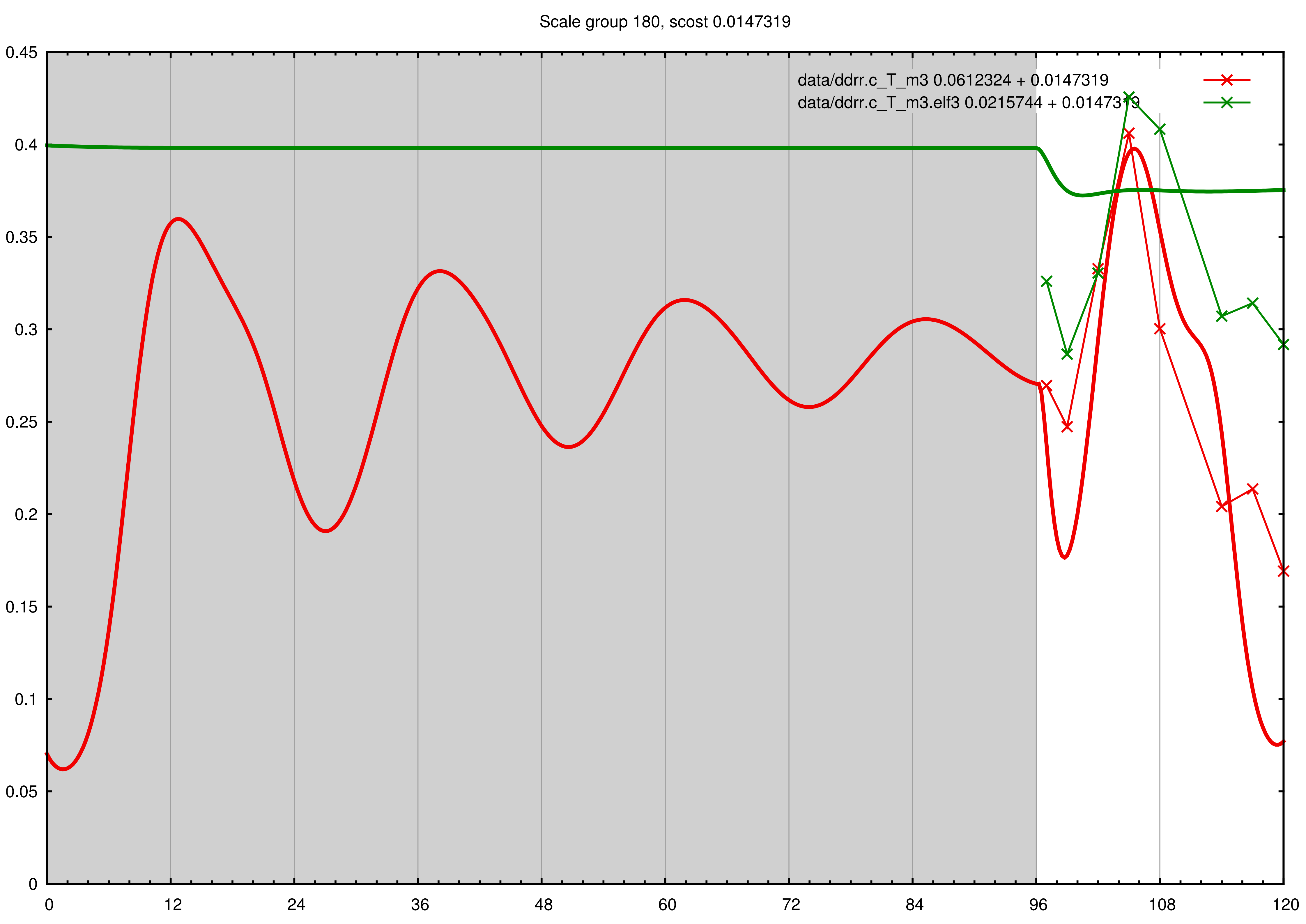
Scale group 180, scost 0.0147319
data/ddrr.c_T_m3 0.0612324 + 0.0147319
data/ddrr.c_T_m3.elf3 0.0215744 + 0.0147319
0.45
0.4
0.35
0.3
0.25
0.2
0.15
0.1
0.05
0
0
12
24
36
48
60
72
84
96
108
120

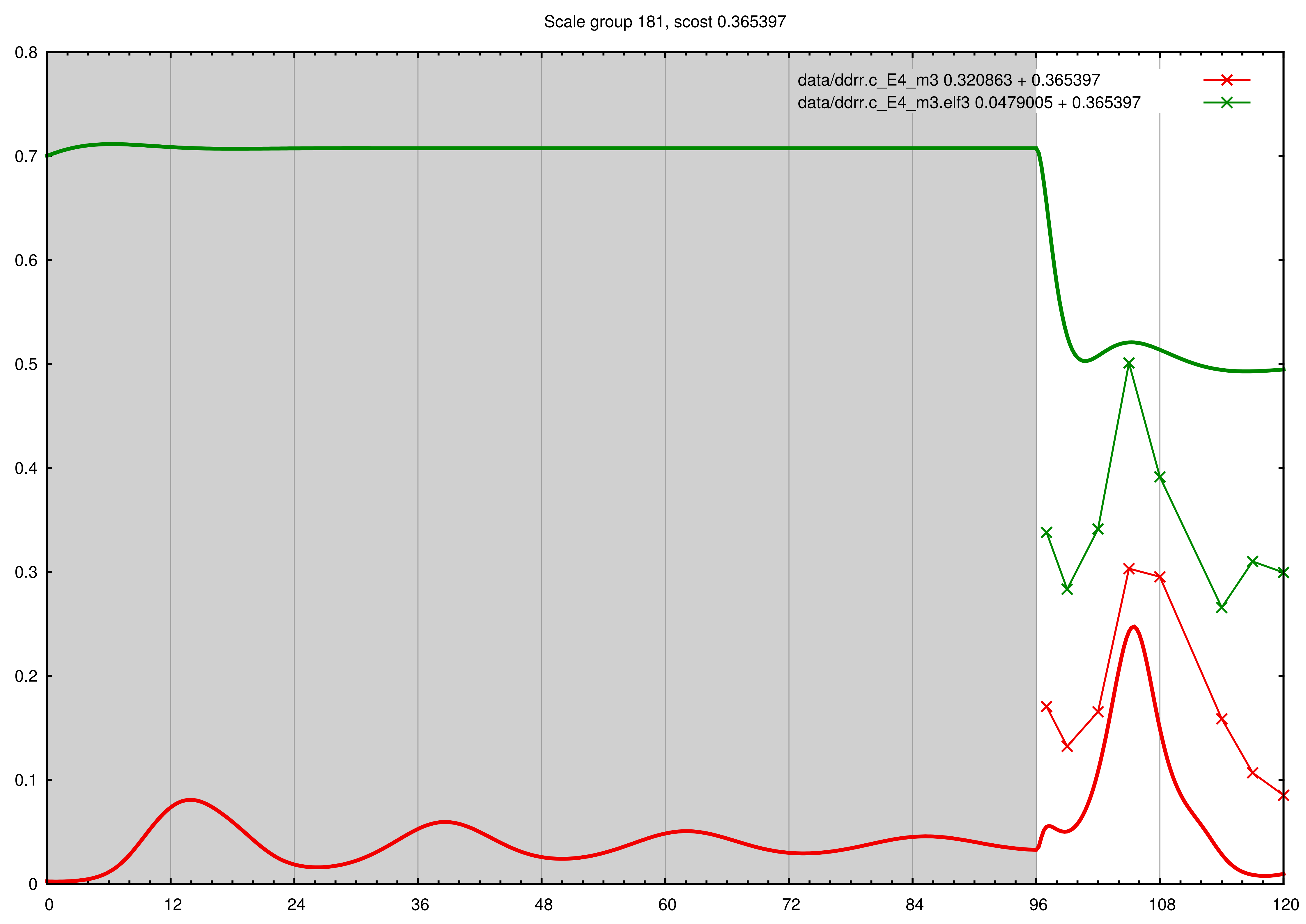

Scale group 181, scost 0.365397
data/ddrr.c_E4_m3 0.320863 + 0.365397
data/ddrr.c_E4_m3.elf3 0.0479005 + 0.365397
0
0.1
0.2
0.3
0.4
0.5
0.6
0.7
0.8
0
12
24
36
48
60
72
84
96
108
120

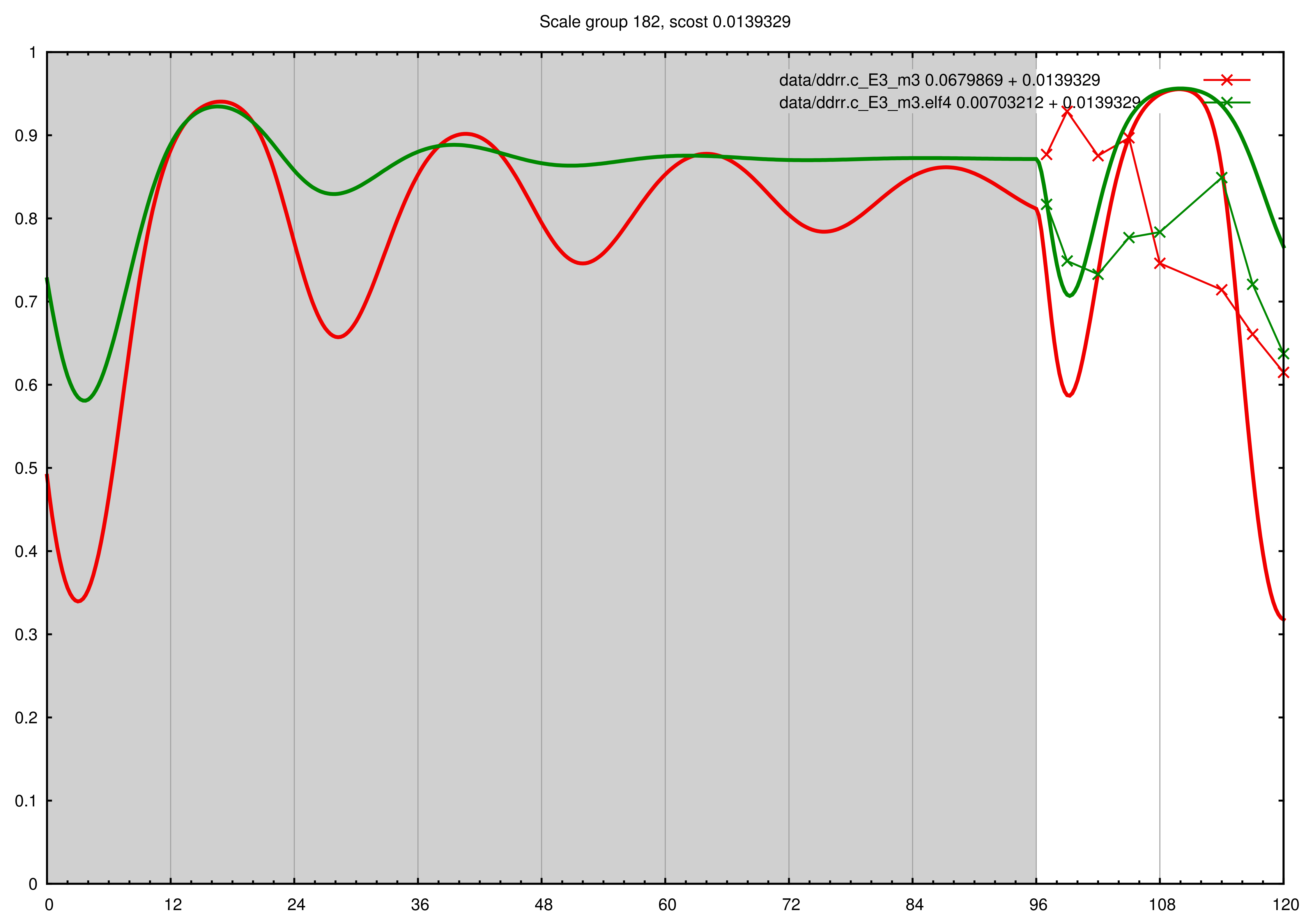

Scale group 182, scost 0.0139329
data/ddrr.c_E3_m3 0.0679869 + 0.0139329
data/ddrr.c_E3_m3.elf4 0.00703212 + 0.0139329
0
0.1
0.2
0.3
0.4
0.5
0.6
0.7
0.8
0.9
1
0
12
24
36
48
60
72
84
96
108
120

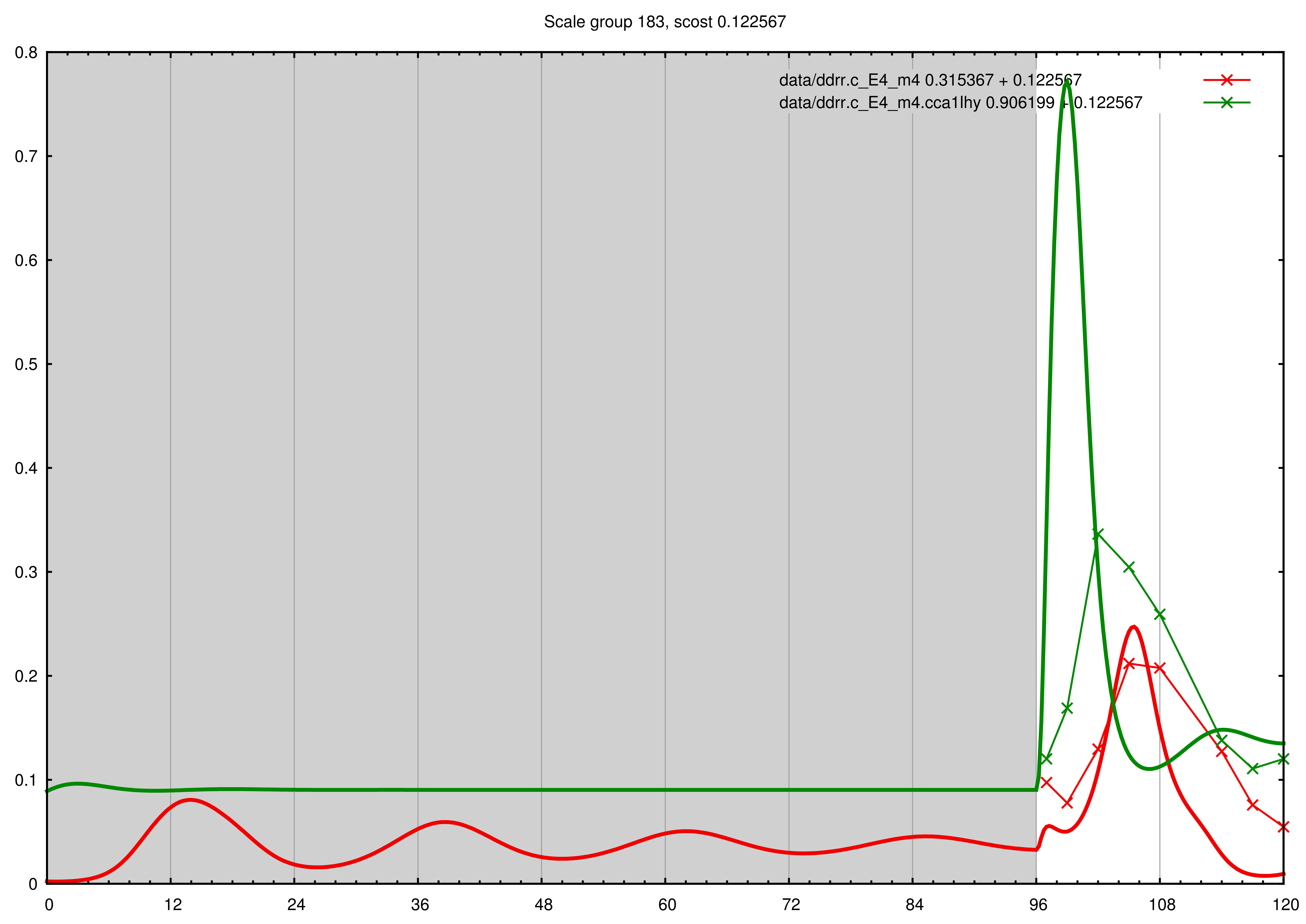
Scale group 183, scost 0.122567
data/ddrr.c_E4_m4 0.315367 + 0.122567
data/ddrr.c_E4_m4.cca1lhy 0.906199 + 0.122567
0.8
0.7
0.6
0.5
0.4
0.3
0.2
0.1
0
0
12
24
36
48
60
72
84
96
108
120

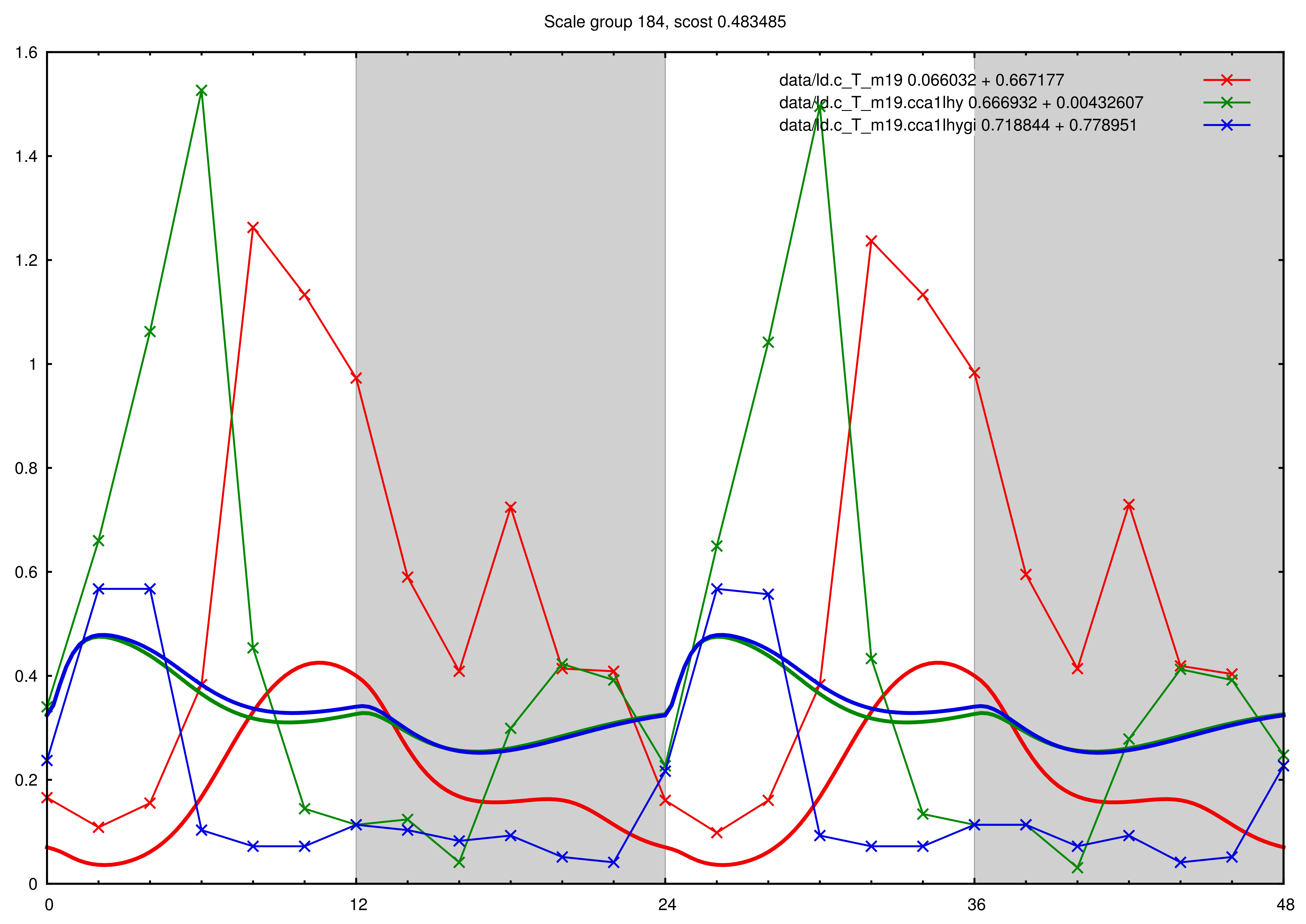

Scale group 184, scost 0.483485
data/ld.c_T_m19 0.066032 + 0.667177
data/ld.c_T_m19.cca1lhy 0.666932 + 0.00432607
data/ld.c_T_m19.cca1lhygi 0.718844 + 0.778951
1.6
1.4
1.2
1
0.8
0.6
0.4
0.2
0
0
12
24
36
48

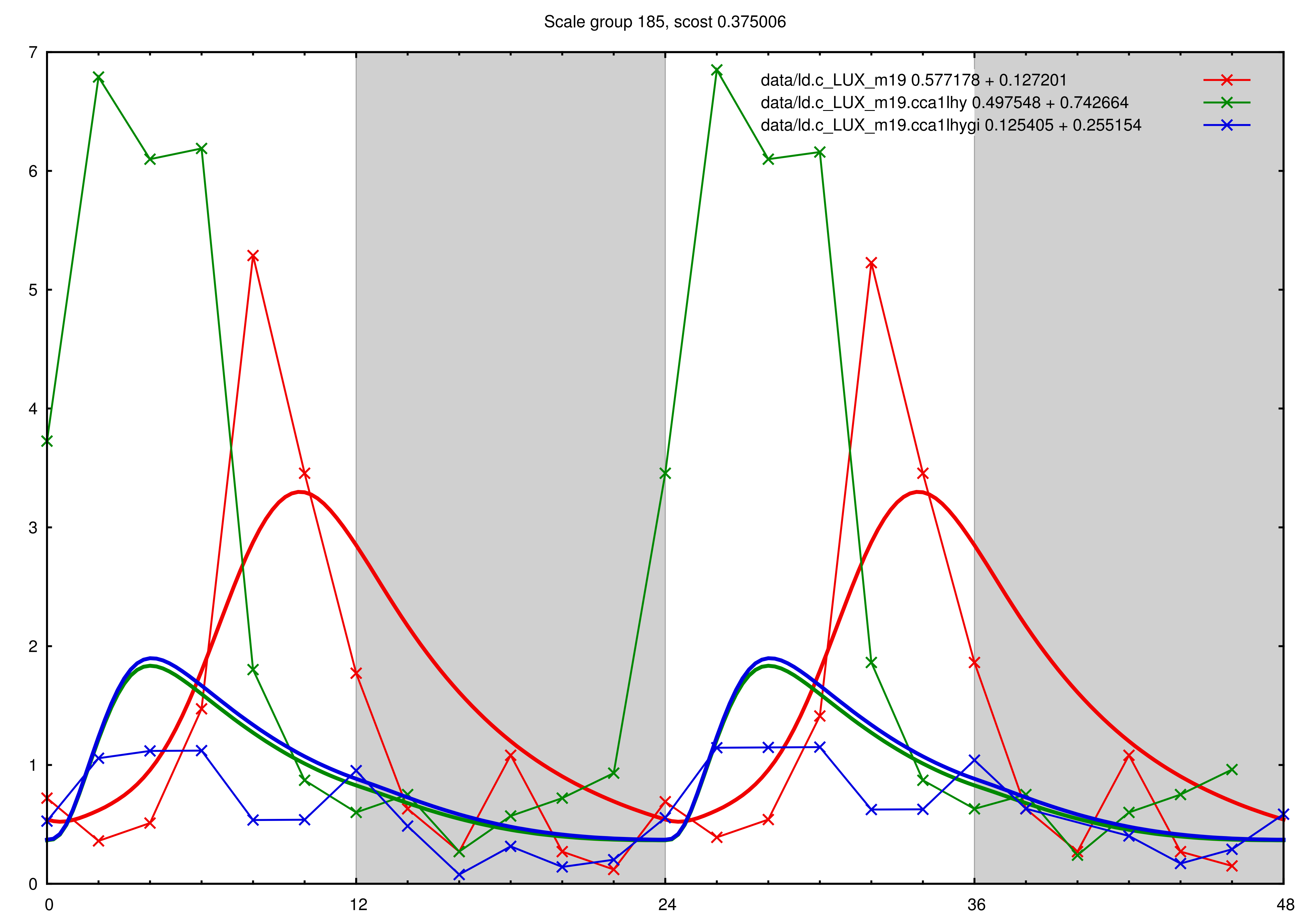
Scale group 185, scost 0.375006
data/ld.c_LUX_m19 0.577178 + 0.127201
data/ld.c_LUX_m19.cca1lhy 0.497548 + 0.742664
data/ld.c_LUX_m19.cca1lhygi 0.125405 + 0.255154
0
1
2
3
4
5
6
7
0
12
24
36
48

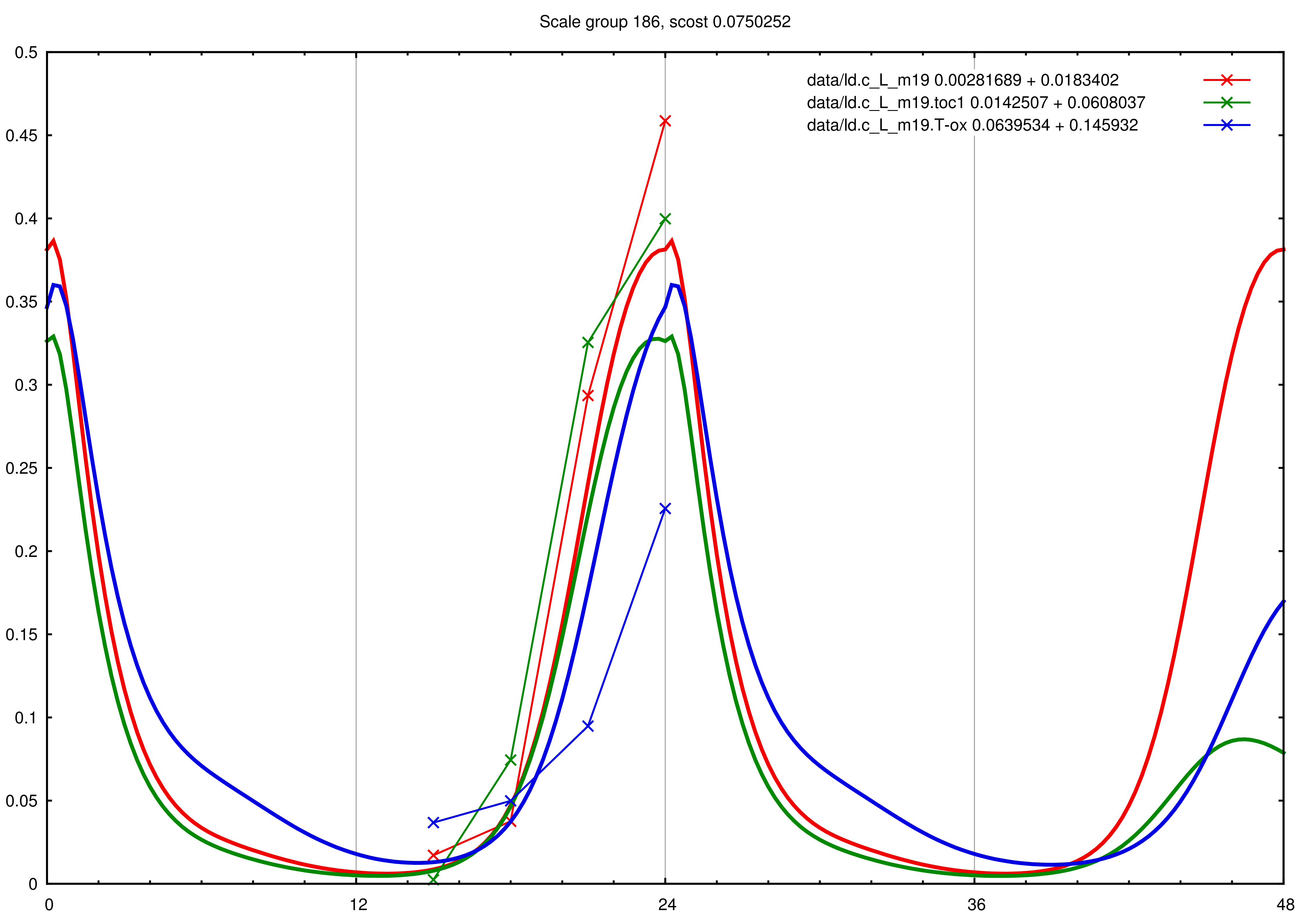
Scale group 186, scost 0.0750252
data/ld.c_L_m19 0.00281689 + 0.0183402
data/ld.c_L_m19.toc1 0.0142507 + 0.0608037
data/ld.c_L_m19.T-ox 0.0639534 + 0.145932
0.5
0.45
0.4
0.35
0.3
0.25
0.2
0.15
0.1
0.05
0
0
12
24
36
48

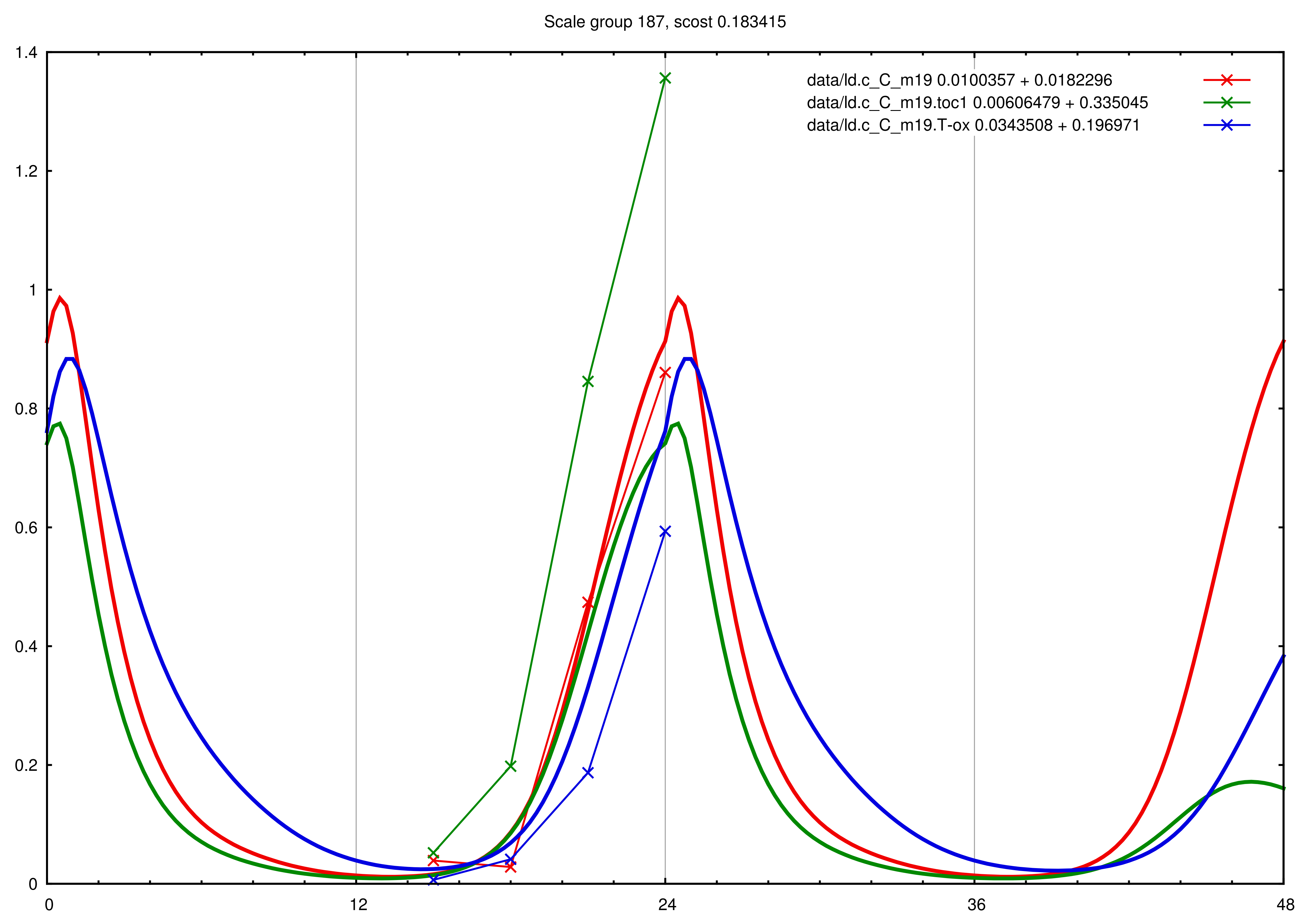

Scale group 187, scost 0.183415
data/ld.c_C_m19 0.0100357 + 0.0182296
data/ld.c_C_m19.toc1 0.00606479 + 0.335045
data/ld.c_C_m19.T-ox 0.0343508 + 0.196971
1.4
1.2
1
0.8
0.6
0.4
0.2
0
0
12
24
36
48

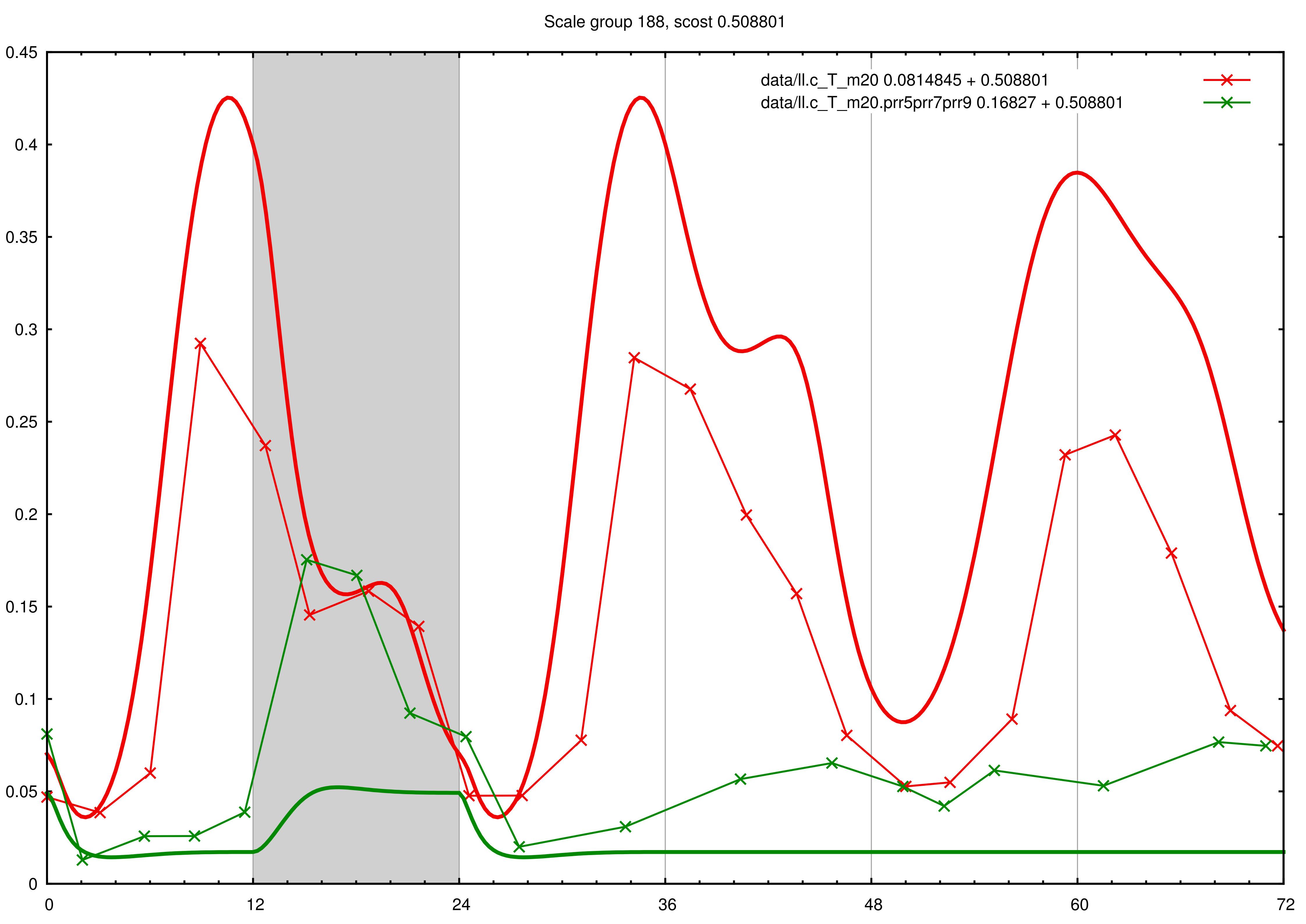

Scale group 188, scost 0.508801
data/ll.c_T_m20 0.0814845 + 0.508801
data/ll.c_T_m20.prr5prr7prr9 0.16827 + 0.508801
0.45
0.4
0.35
0.3
0.25
0.2
0.15
0.1
0.05
0
0
12
24
36
48
60
72

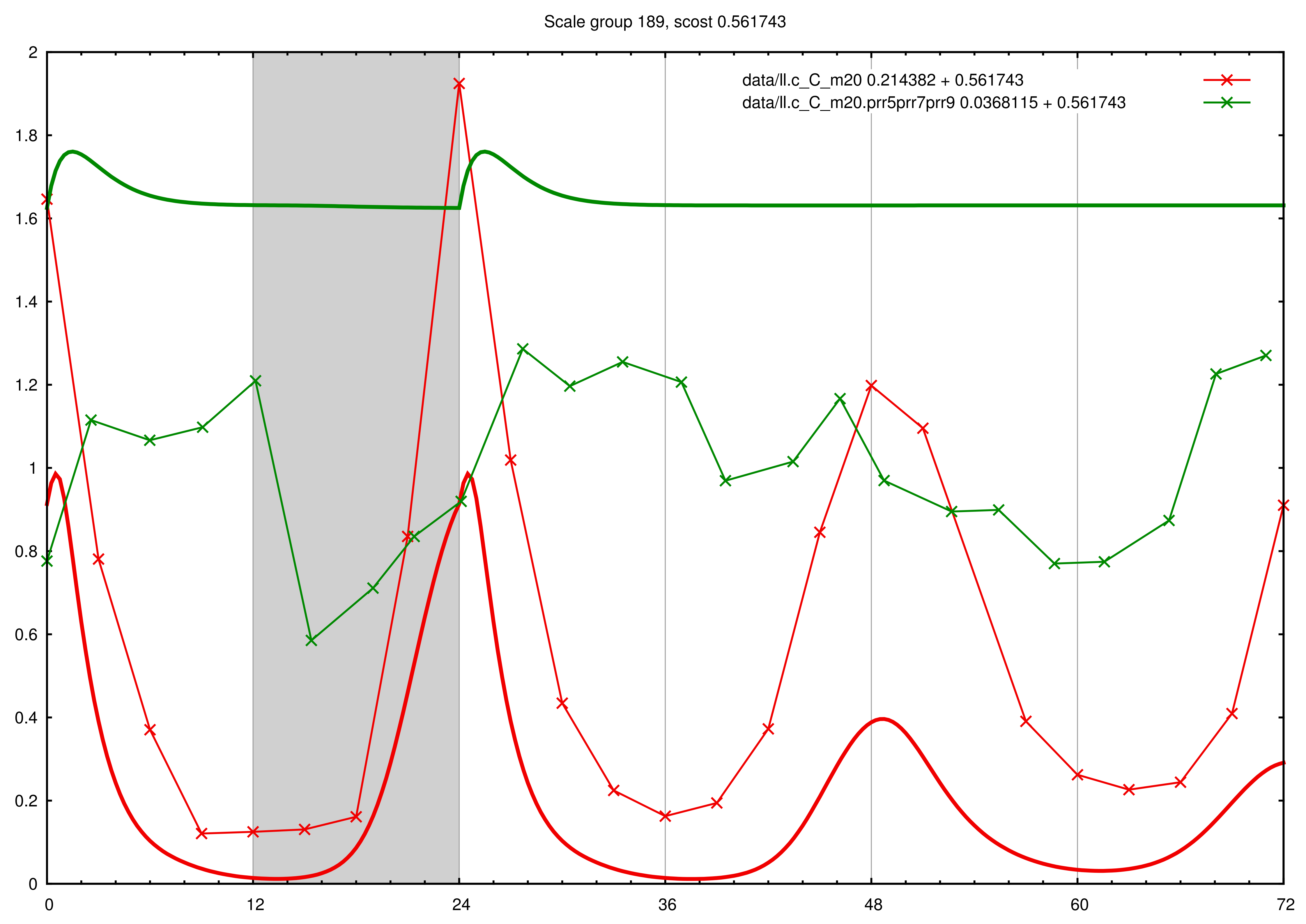

Scale group 189, scost 0.561743
data/ll.c_C_m20 0.214382 + 0.561743
data/ll.c_C_m20.prr5prr7prr9 0.0368115 + 0.561743
2
1.8
1.6
1.4
1.2
1
0.8
0.6
0.4
0.2
0
0
12
24
36
48
60
72

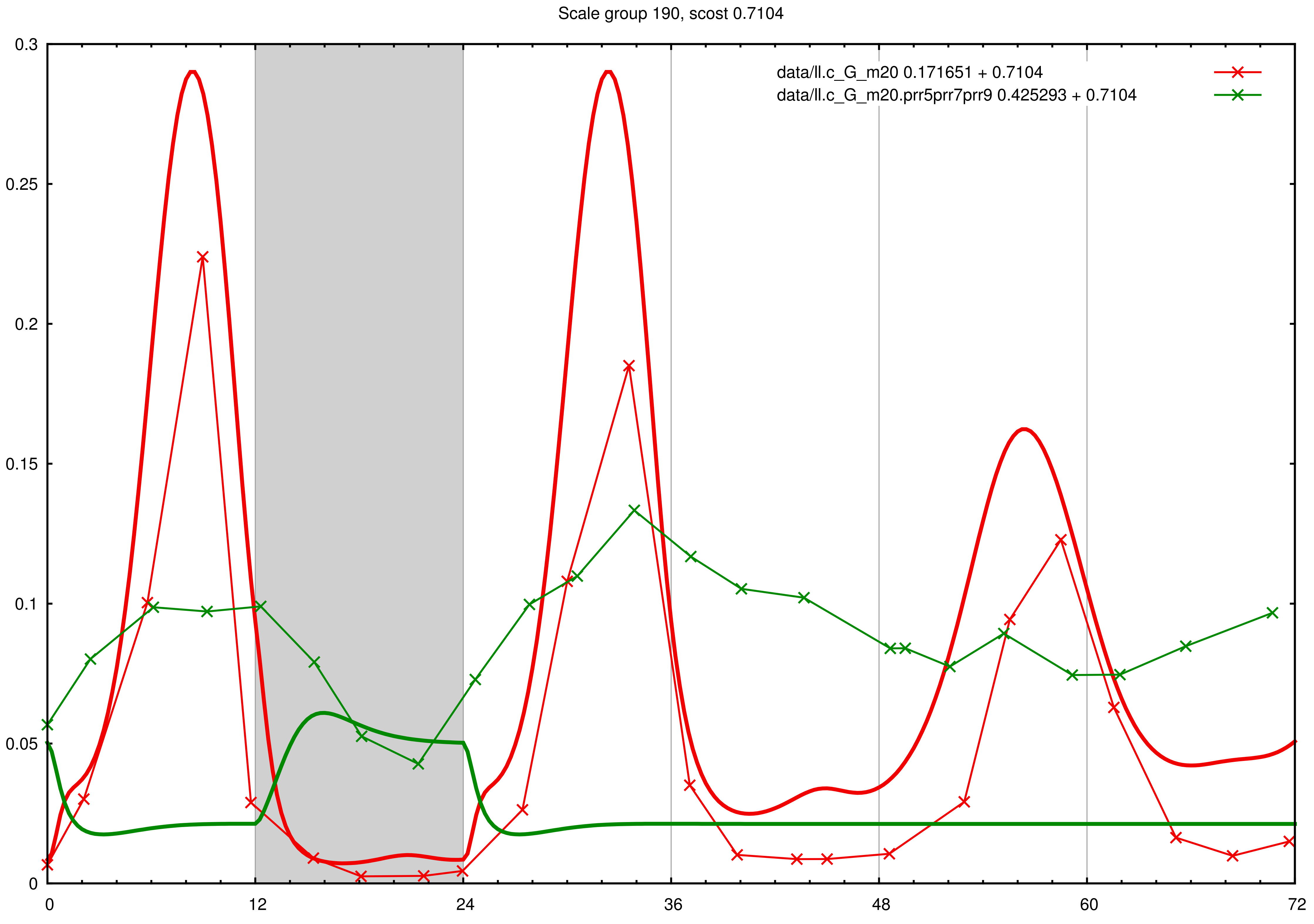
Scale group 190, scost 0.7104
data/ll.c_G_m20 0.171651 + 0.7104
data/ll.c_G_m20.prr5prr7prr9 0.425293 + 0.7104
0
0.05
0.1
0.15
0.2
0.25
0.3
0
12
24
36
48
60
72

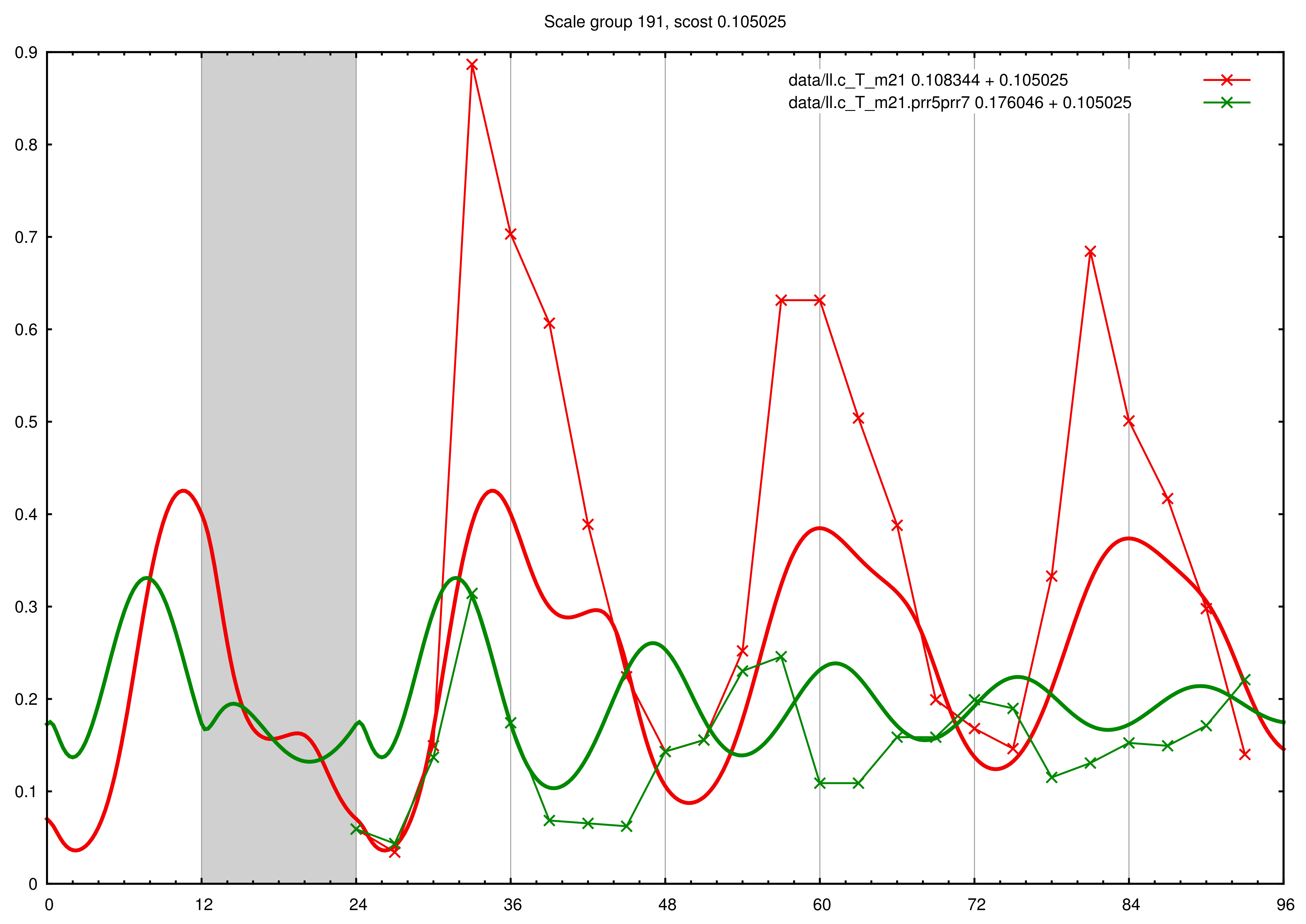

Scale group 191, scost 0.105025
data/ll.c_T_m21 0.108344 + 0.105025
data/ll.c_T_m21.prr5prr7 0.176046 + 0.105025
0.9
0.8
0.7
0.6
0.5
0.4
0.3
0.2
0.1
0
0
12
24
36
48
60
72
84
96

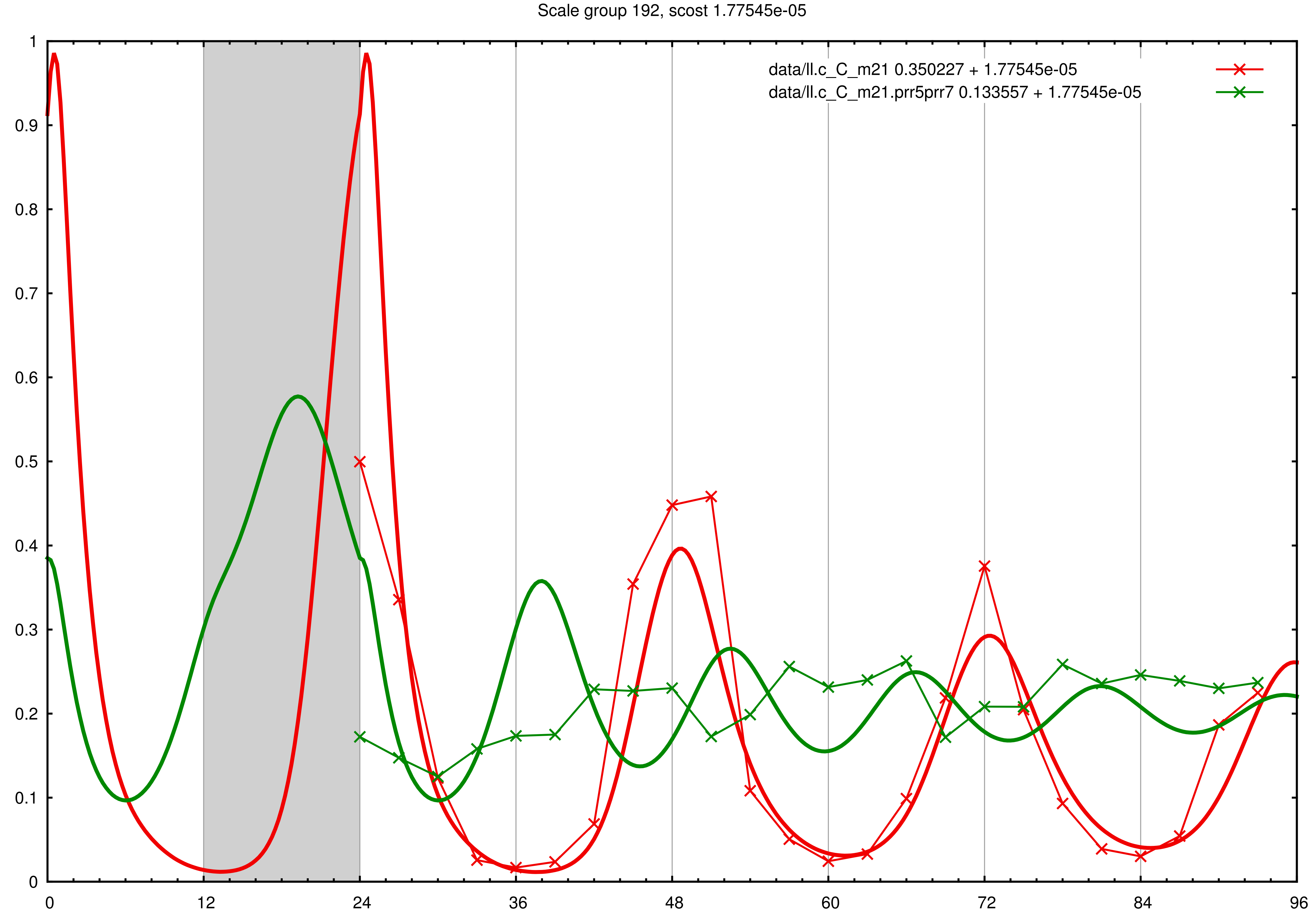

Scale group 192, scost 1.77545e-05
data/ll.c_C_m21 0.350227 + 1.77545e-05
data/ll.c_C_m21.prr5prr7 0.133557 + 1.77545e-05
0
0.1
0.2
0.3
0.4
0.5
0.6
0.7
0.8
0.9
1
0
12
24
36
48
60
72
84
96

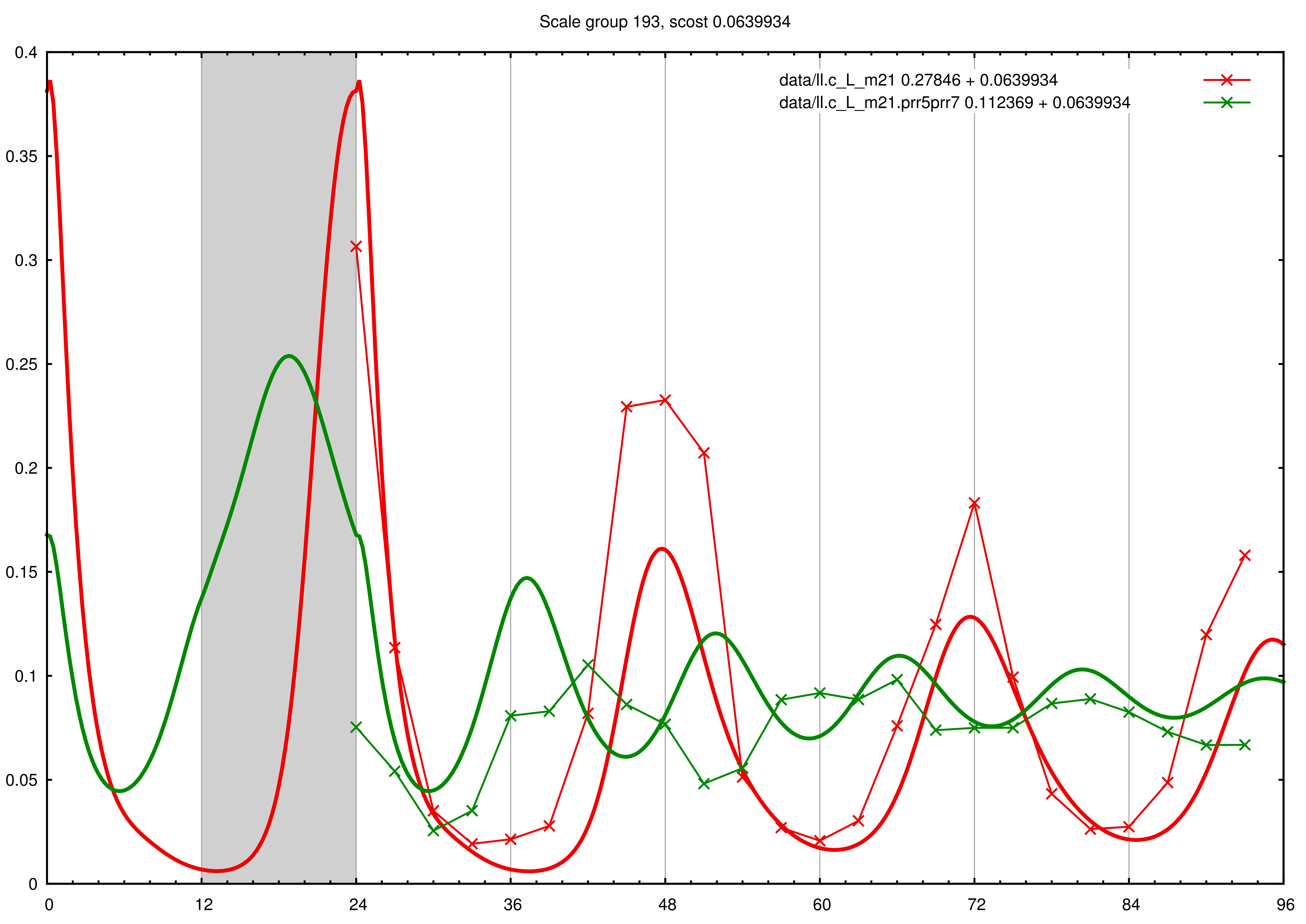
Scale group 193, scost 0.0639934
data/ll.c_L_m21 0.27846 + 0.0639934
data/ll.c_L_m21.prr5prr7 0.112369 + 0.0639934
0.4
0.35
0.3
0.25
0.2
0.15
0.1
0.05
0
0
12
24
36
48
60
72
84
96

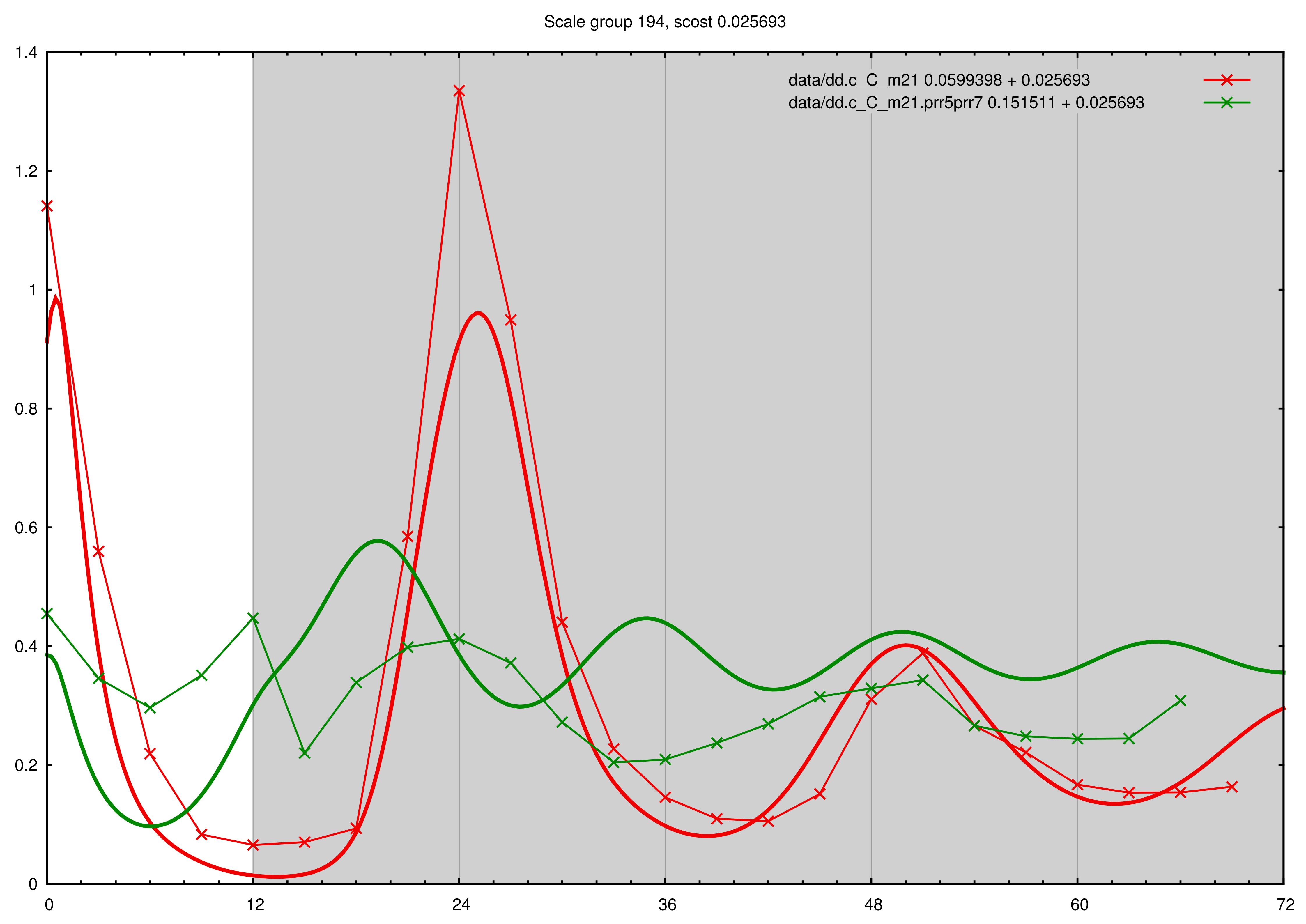

Scale group 194, scost 0.025693
data/dd.c_C_m21 0.0599398 + 0.025693
data/dd.c_C_m21.prr5prr7 0.151511 + 0.025693
1.4
1.2
1
0.8
0.6
0.4
0.2
0
0
12
24
36
48
60
72

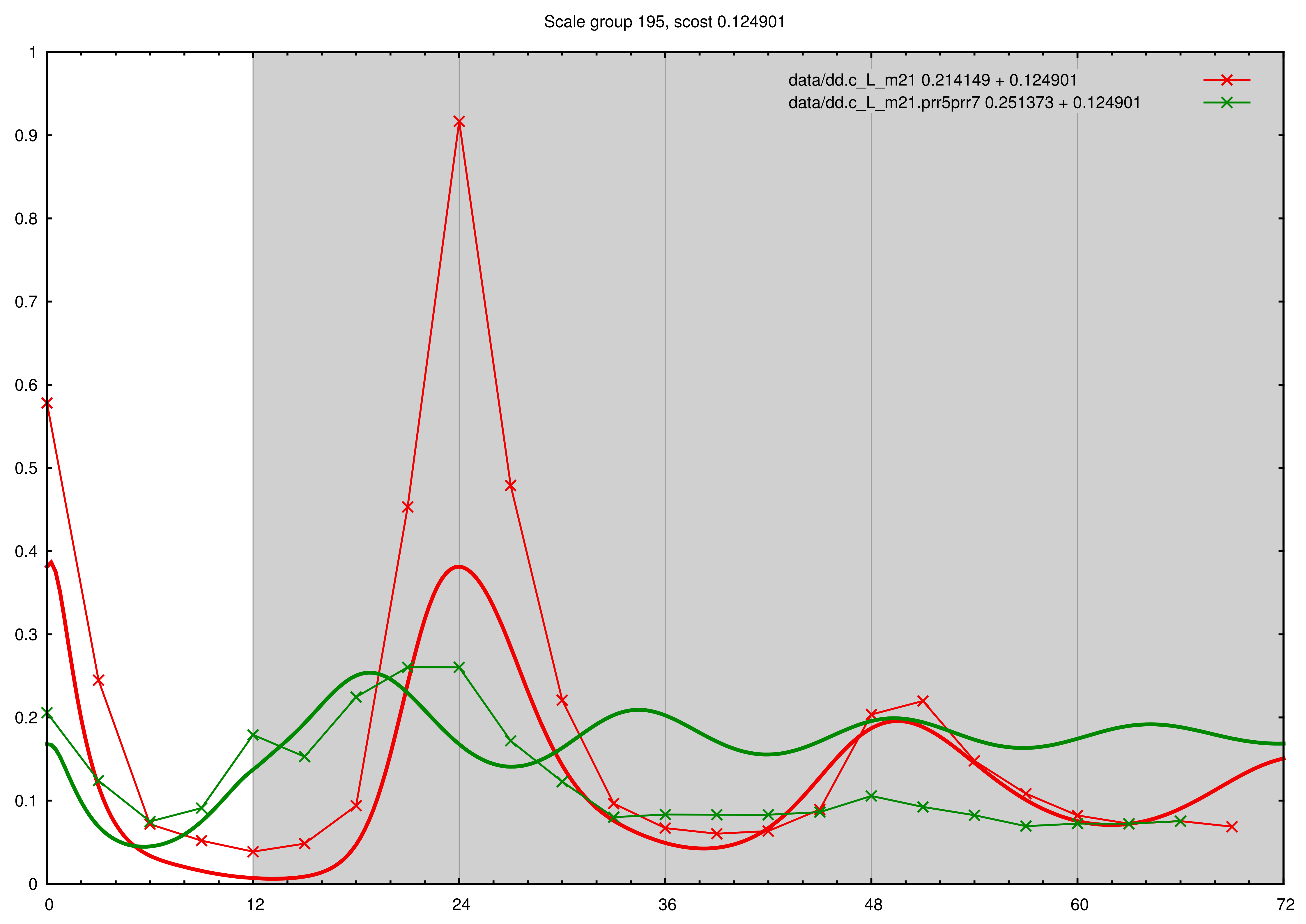

Scale group 195, scost 0.124901
data/dd.c_L_m21 0.214149 + 0.124901
data/dd.c_L_m21.prr5prr7 0.251373 + 0.124901
0
0.1
0.2
0.3
0.4
0.5
0.6
0.7
0.8
0.9
1
0
12
24
36
48
60
72

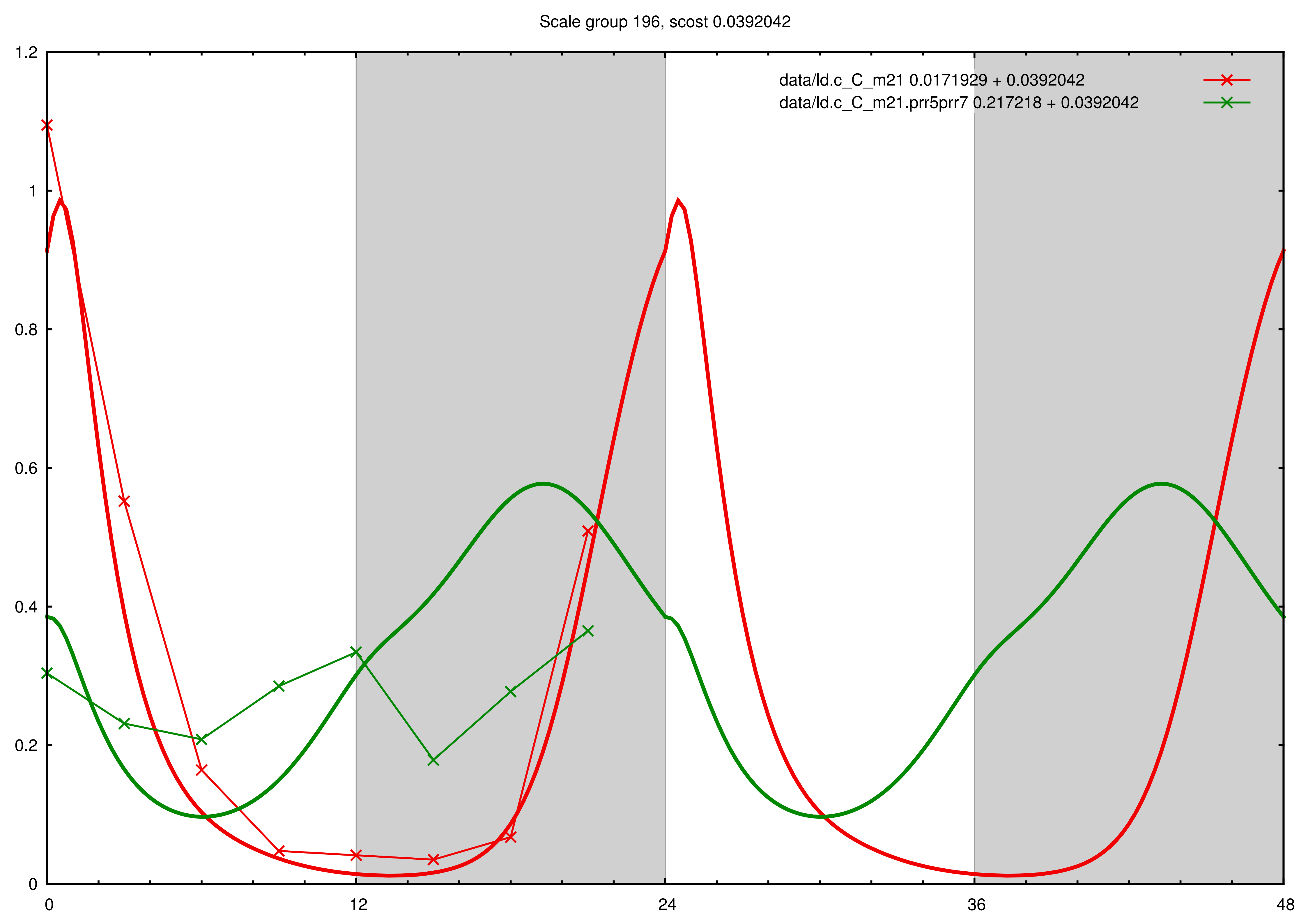
Scale group 196, scost 0.0392042
data/ld.c_C_m21 0.0171929 + 0.0392042
data/ld.c_C_m21.prr5prr7 0.217218 + 0.0392042
1.2
1
0.8
0.6
0.4
0.2
0
0
12
24
36
48

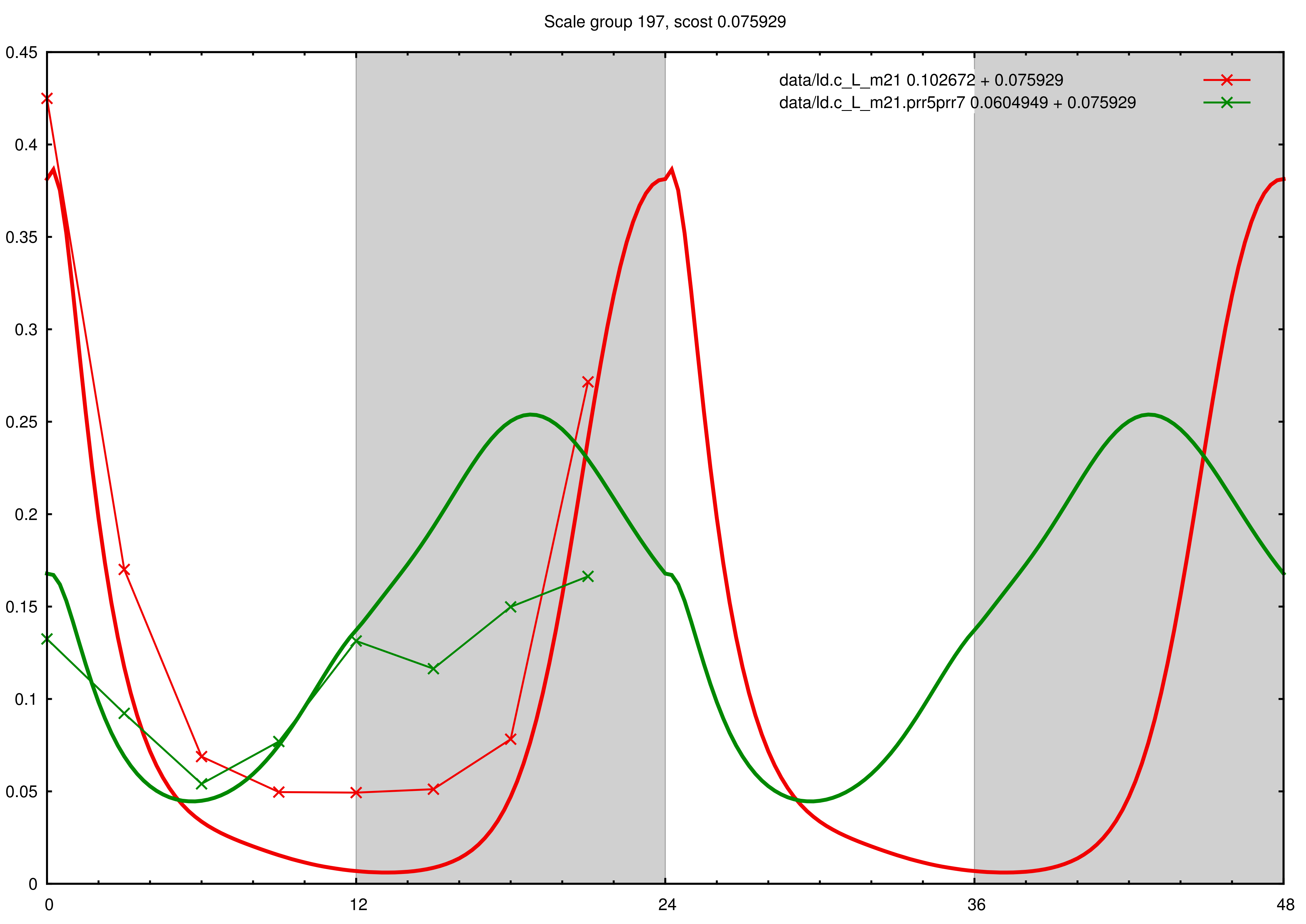

Scale group 197, scost 0.075929
data/ld.c_L_m21 0.102672 + 0.075929
data/ld.c_L_m21.prr5prr7 0.0604949 + 0.075929
0.45
0.4
0.35
0.3
0.25
0.2
0.15
0.1
0.05
0
0
12
24
36
48

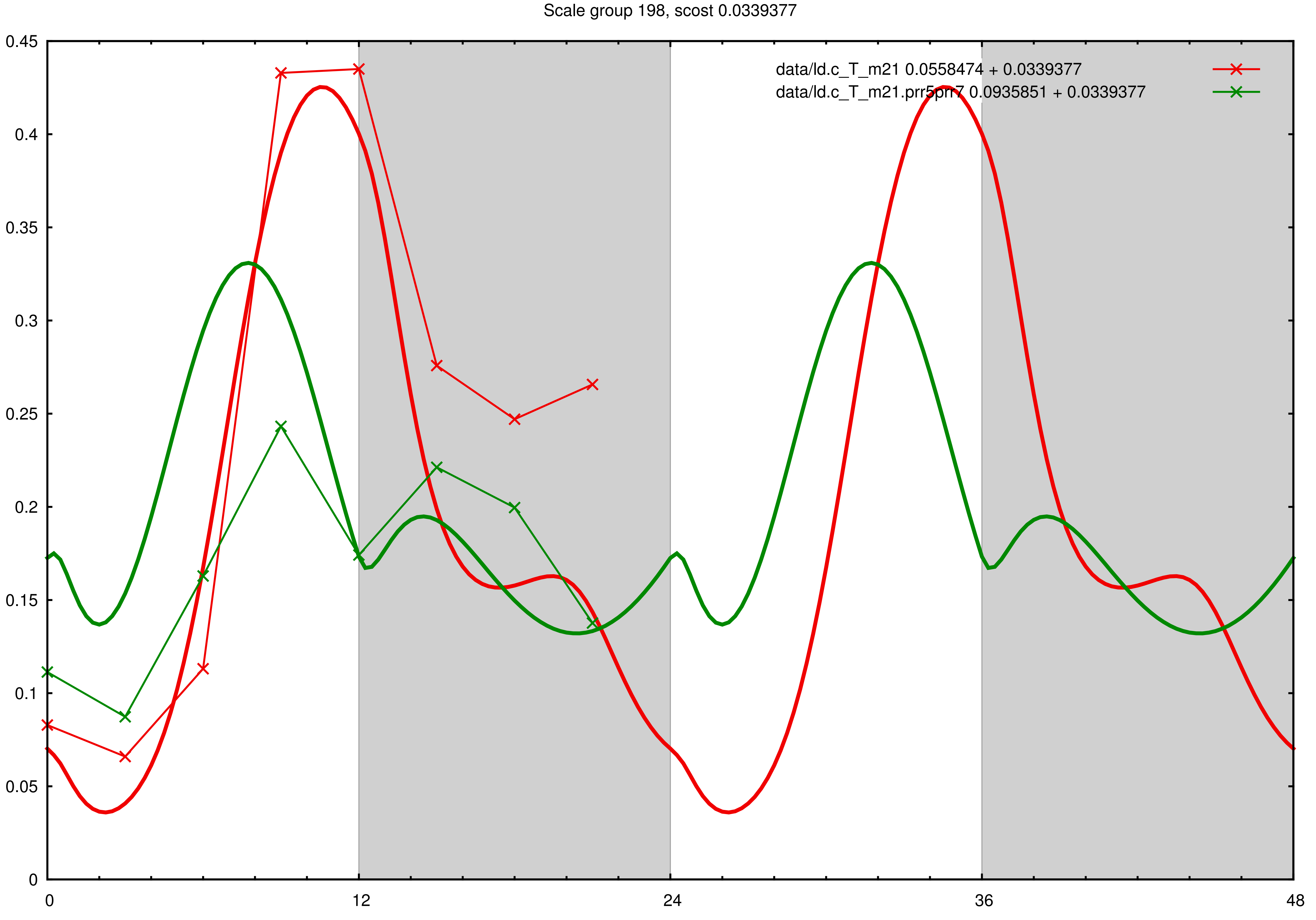

Scale group 198, scost 0.0339377
data/ld.c_T_m21 0.0558474 + 0.0339377
data/ld.c_T_m21.prr5prr7 0.0935851 + 0.0339377
0.45
0.4
0.35
0.3
0.25
0.2
0.15
0.1
0.05
0
0
12
24
36
48

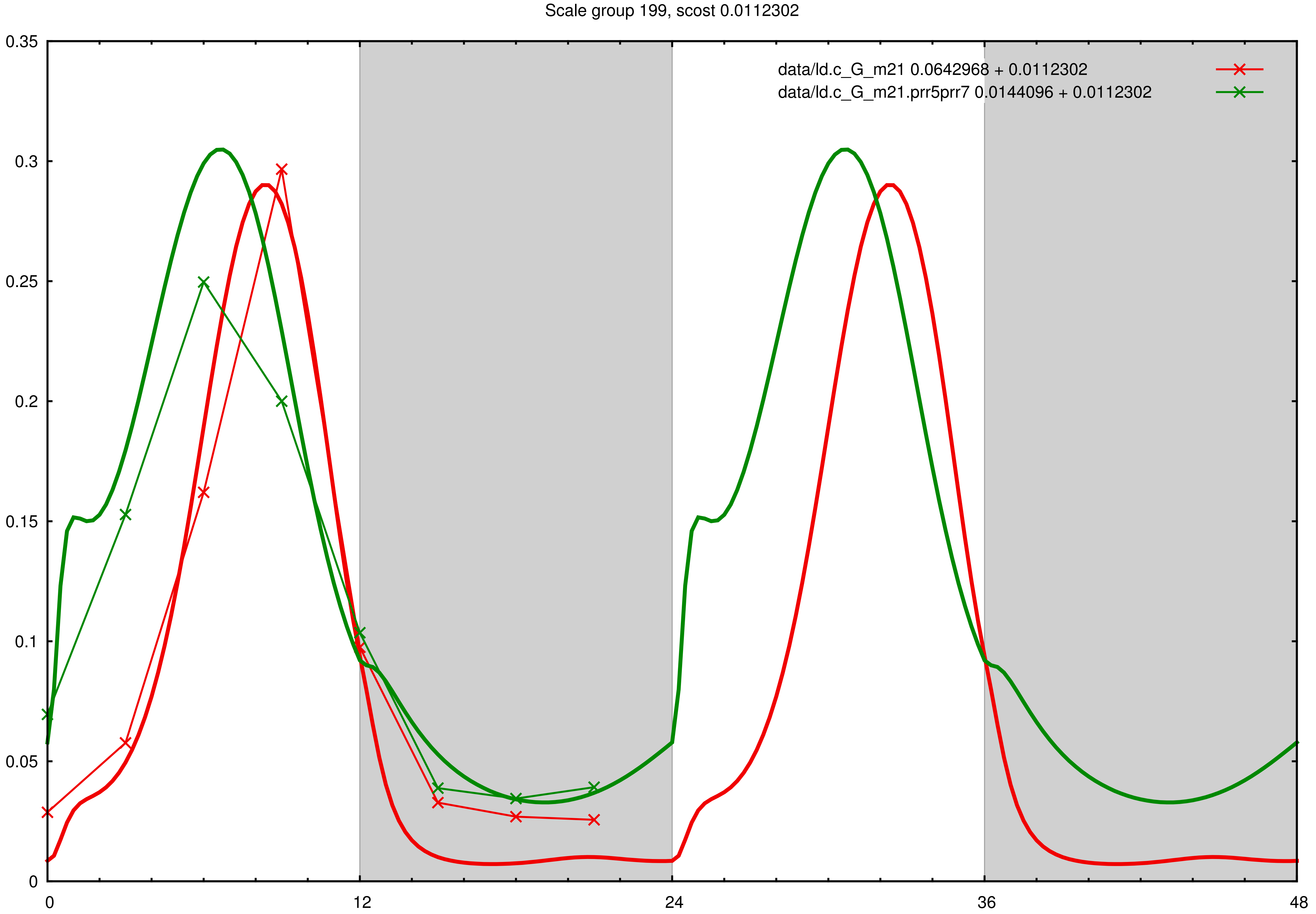
Scale group 199, scost 0.0112302
data/ld.c_G_m21 0.0642968 + 0.0112302
data/ld.c_G_m21.prr5prr7 0.0144096 + 0.0112302
0.35
0.3
0.25
0.2
0.15
0.1
0.05
0
0
12
24
36
48

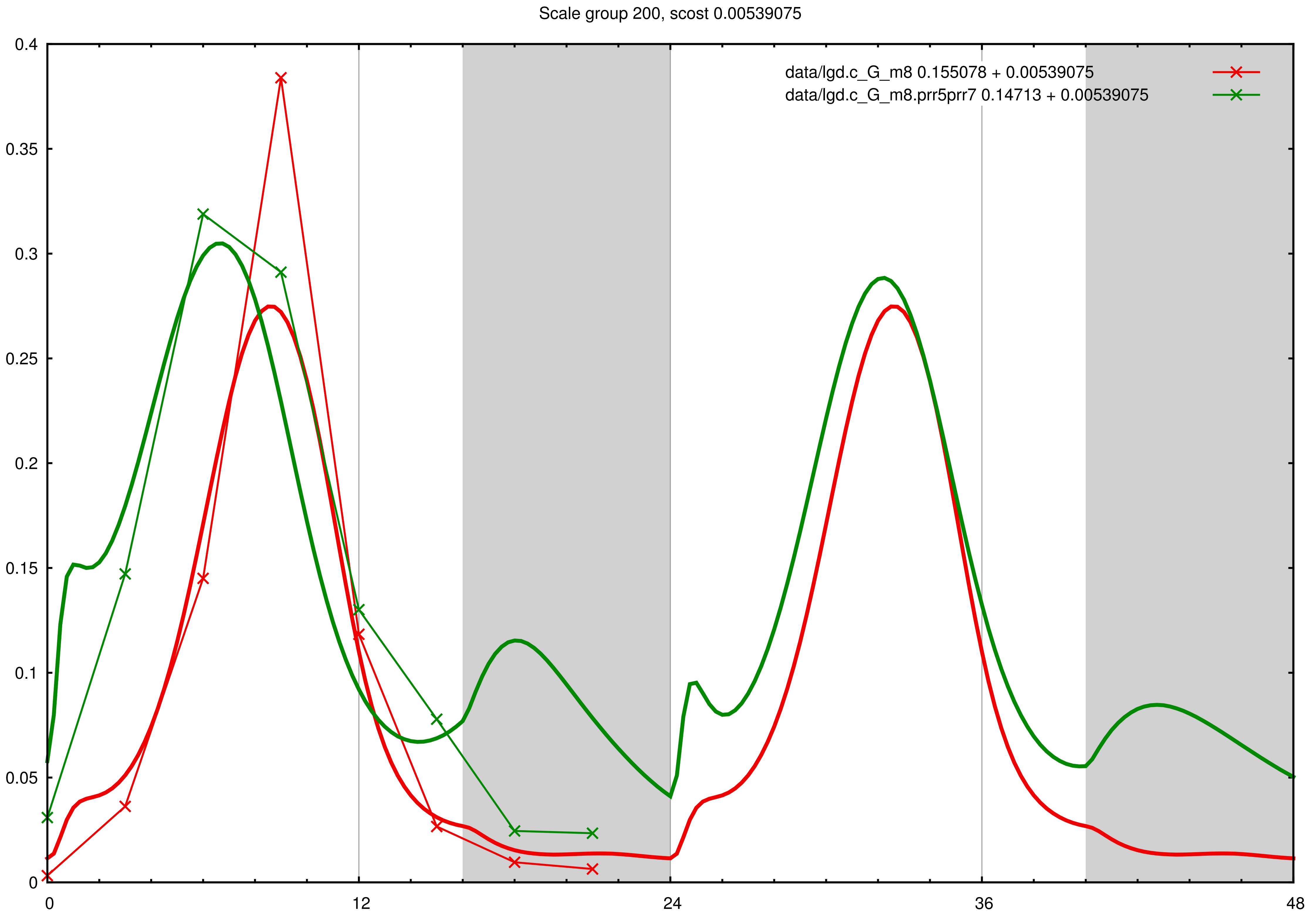

Scale group 200, scost 0.00539075
data/lgd.c_G_m8 0.155078 + 0.00539075
data/lgd.c_G_m8.prr5prr7 0.14713 + 0.00539075
0.4
0.35
0.3
0.25
0.2
0.15
0.1
0.05
0
0
12
24
36
48

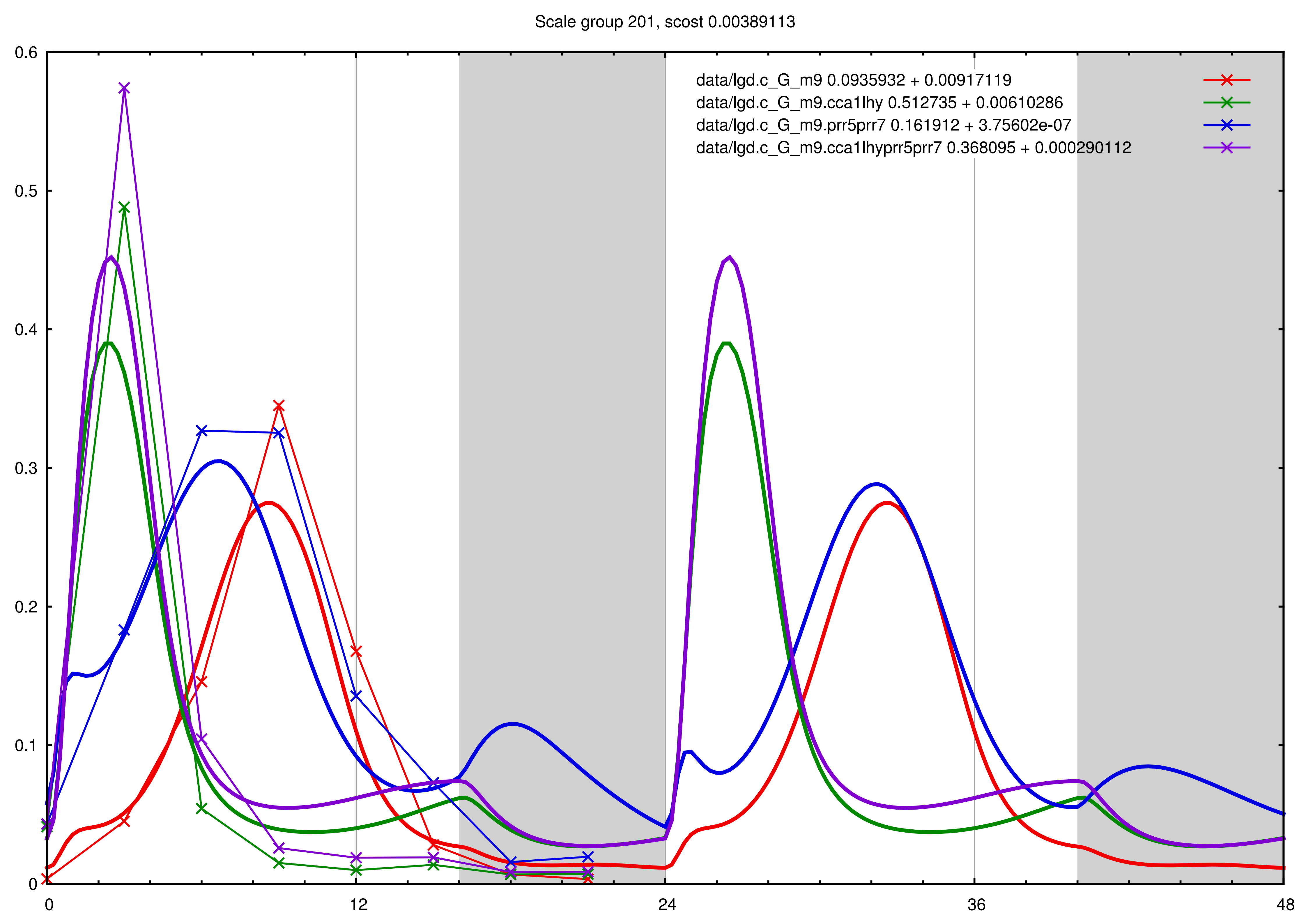

Scale group 201, scost 0.00389113
data/lgd.c_G_m9 0.0935932 + 0.00917119
data/lgd.c_G_m9.cca1lhy 0.512735 + 0.00610286
data/lgd.c_G_m9.prr5prr7 0.161912 + 3.75602e-07
data/lgd.c_G_m9.cca1lhyprr5prr7 0.368095 + 0.000290112
0.6
0.5
0.4
0.3
0.2
0.1
0
0
12
24
36
48

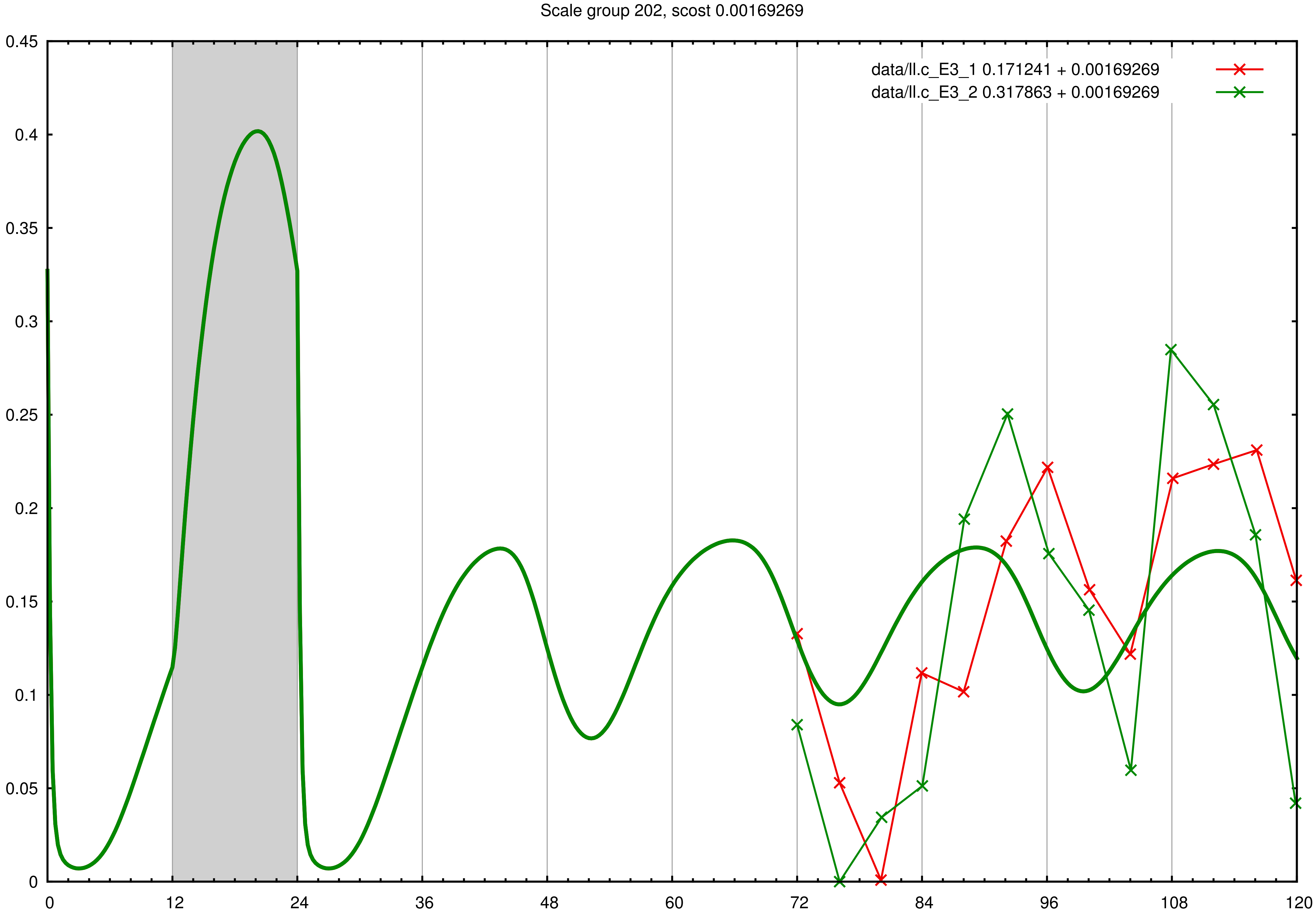

Scale group 202, scost 0.00169269
data/ll.c_E3_1 0.171241 + 0.00169269
data/ll.c_E3_2 0.317863 + 0.00169269
0.45
0.4
0.35
0.3
0.25
0.2
0.15
0.1
0.05
0
0
12
24
36
48
60
72
84
96
108
120

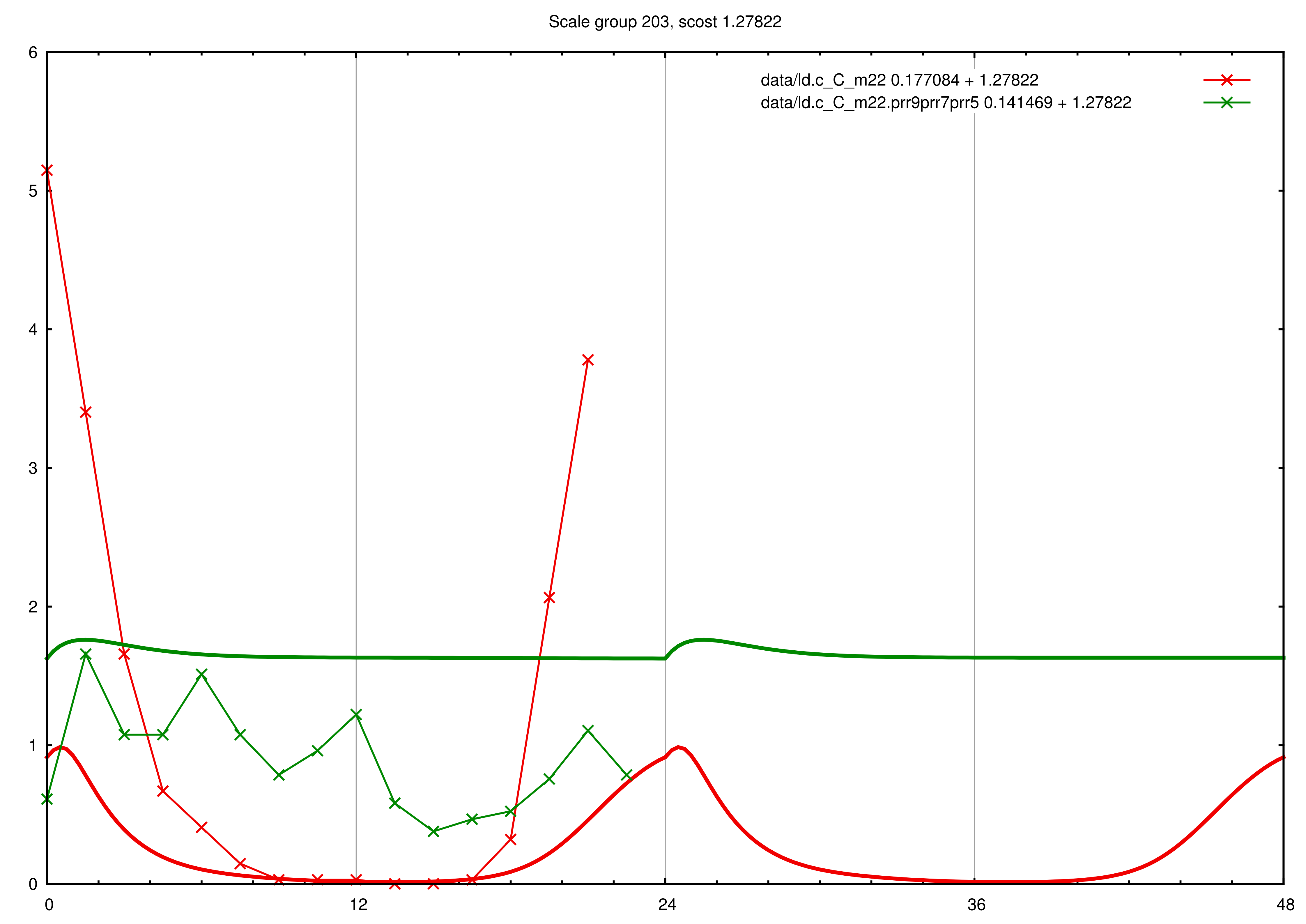
Scale group 203, scost 1.27822
data/ld.c_C_m22 0.177084 + 1.27822
data/ld.c_C_m22.prr9prr7prr5 0.141469 + 1.27822
0
1
2
3
4
5
6
0
12
24
36
48

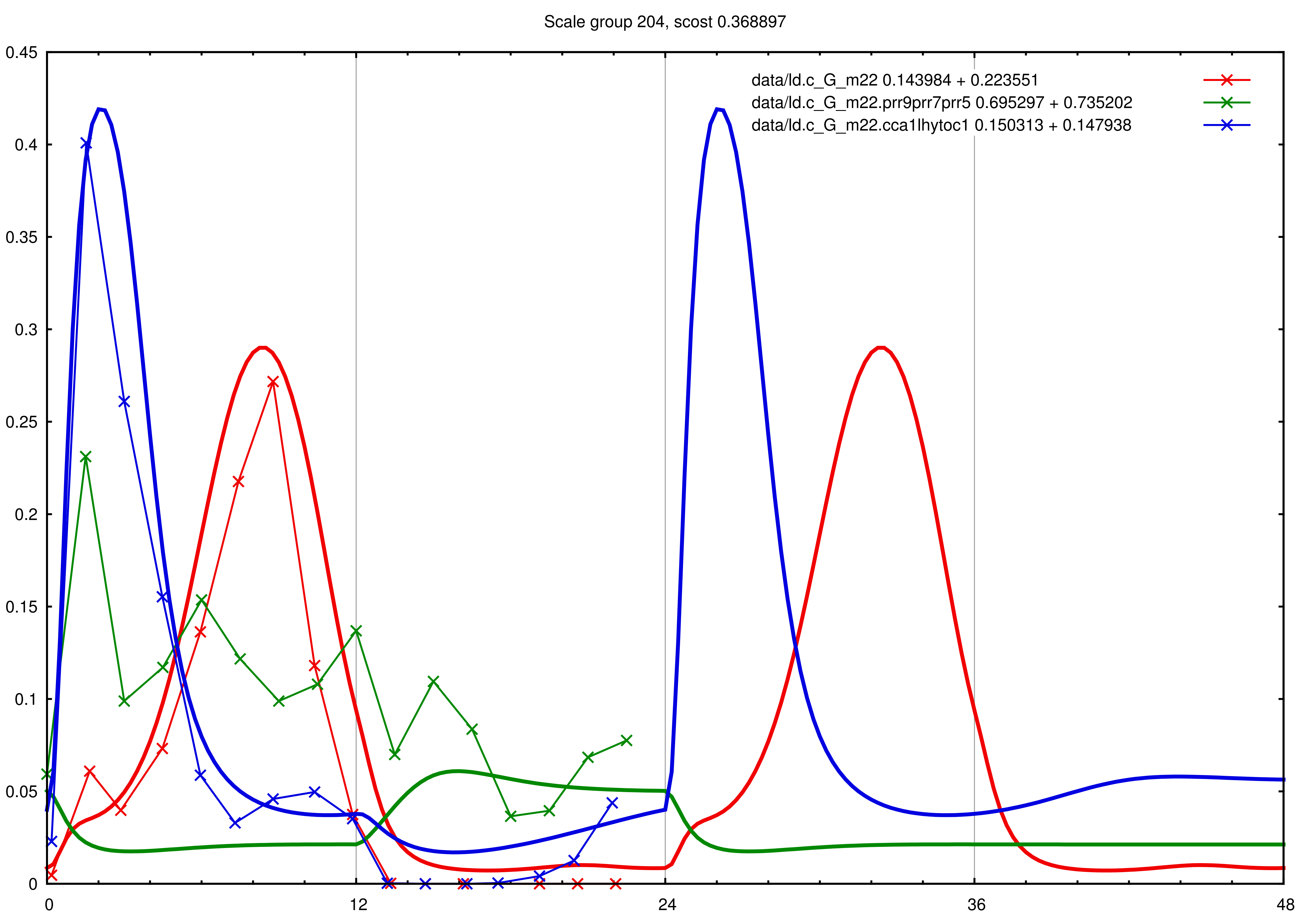

Scale group 204, scost 0.368897
data/ld.c_G_m22 0.143984 + 0.223551
data/ld.c_G_m22.prr9prr7prr5 0.695297 + 0.735202
data/ld.c_G_m22.cca1lhytoc1 0.150313 + 0.147938
0.45
0.4
0.35
0.3
0.25
0.2
0.15
0.1
0.05
0
0
12
24
36
48

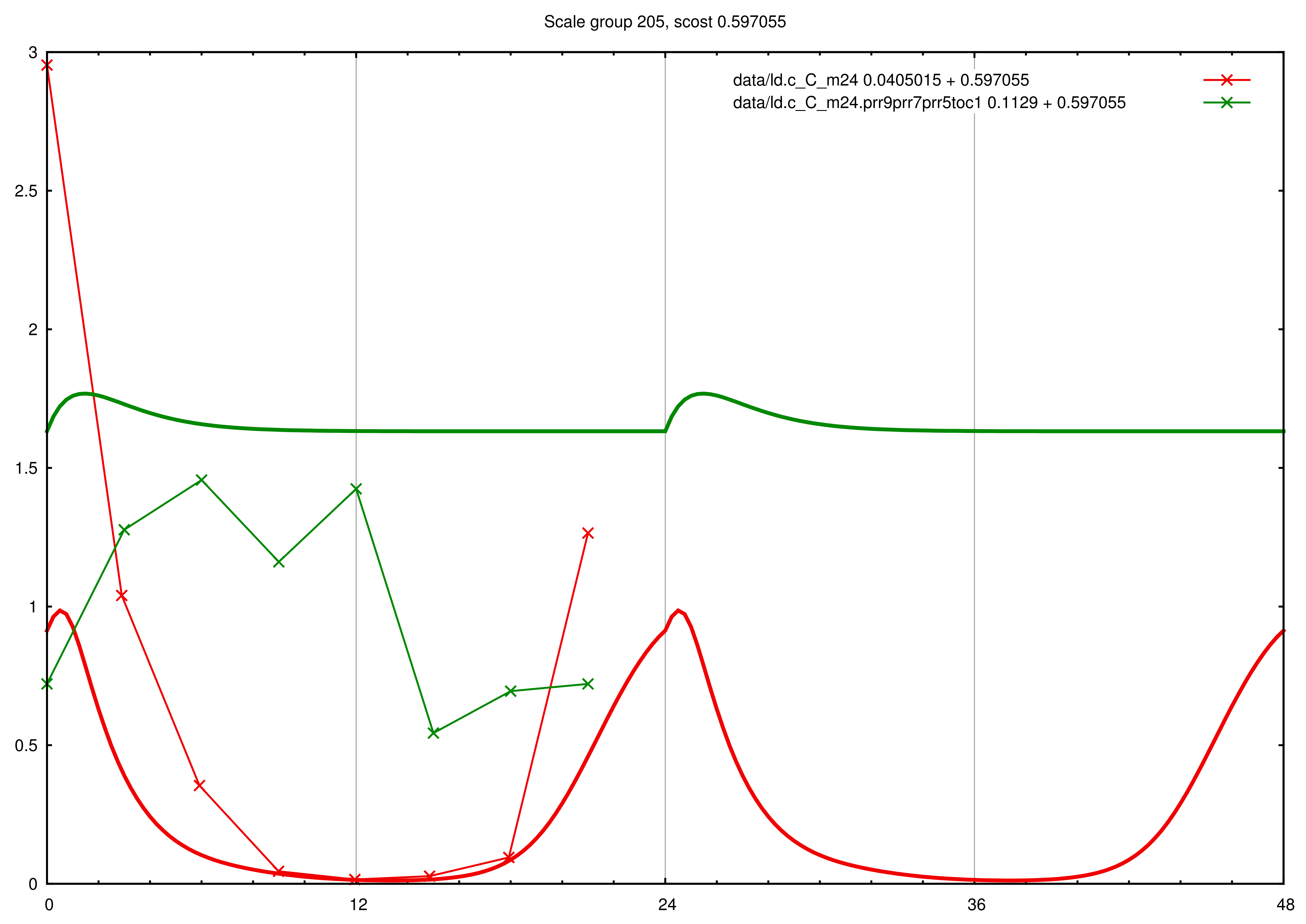

Scale group 205, scost 0.597055
data/ld.c_C_m24 0.0405015 + 0.597055
data/ld.c_C_m24.prr9prr7prr5toc1 0.1129 + 0.597055
3
2.5
2
1.5
1
0.5
0
0
12
24
36
48

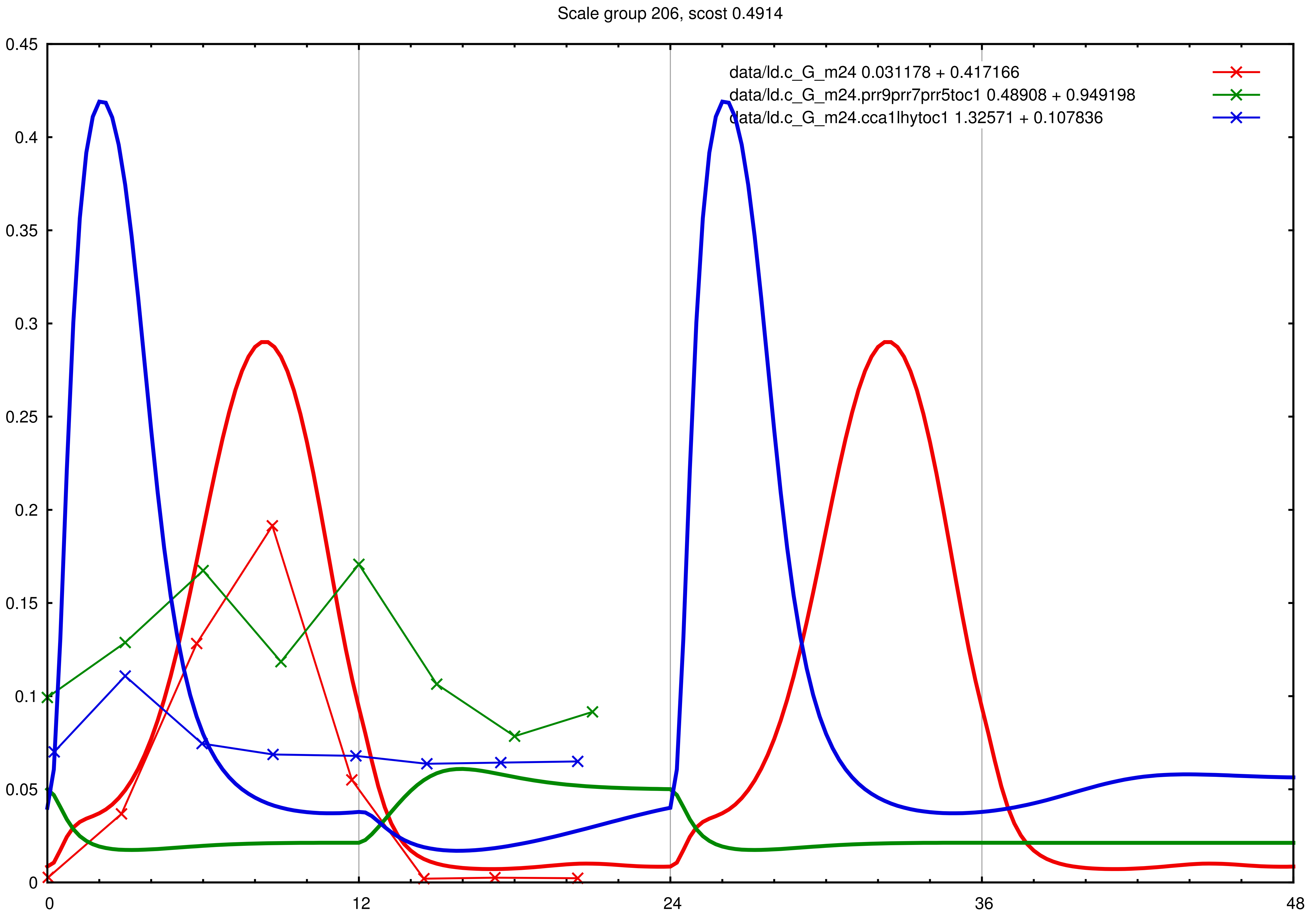

Scale group 206, scost 0.4914
data/ld.c_G_m24 0.031178 + 0.417166
data/ld.c_G_m24.prr9prr7prr5toc1 0.48908 + 0.949198
data/ld.c_G_m24.cca1lhytoc1 1.32571 + 0.107836
0.45
0.4
0.35
0.3
0.25
0.2
0.15
0.1
0.05
0
0
12
24
36
48

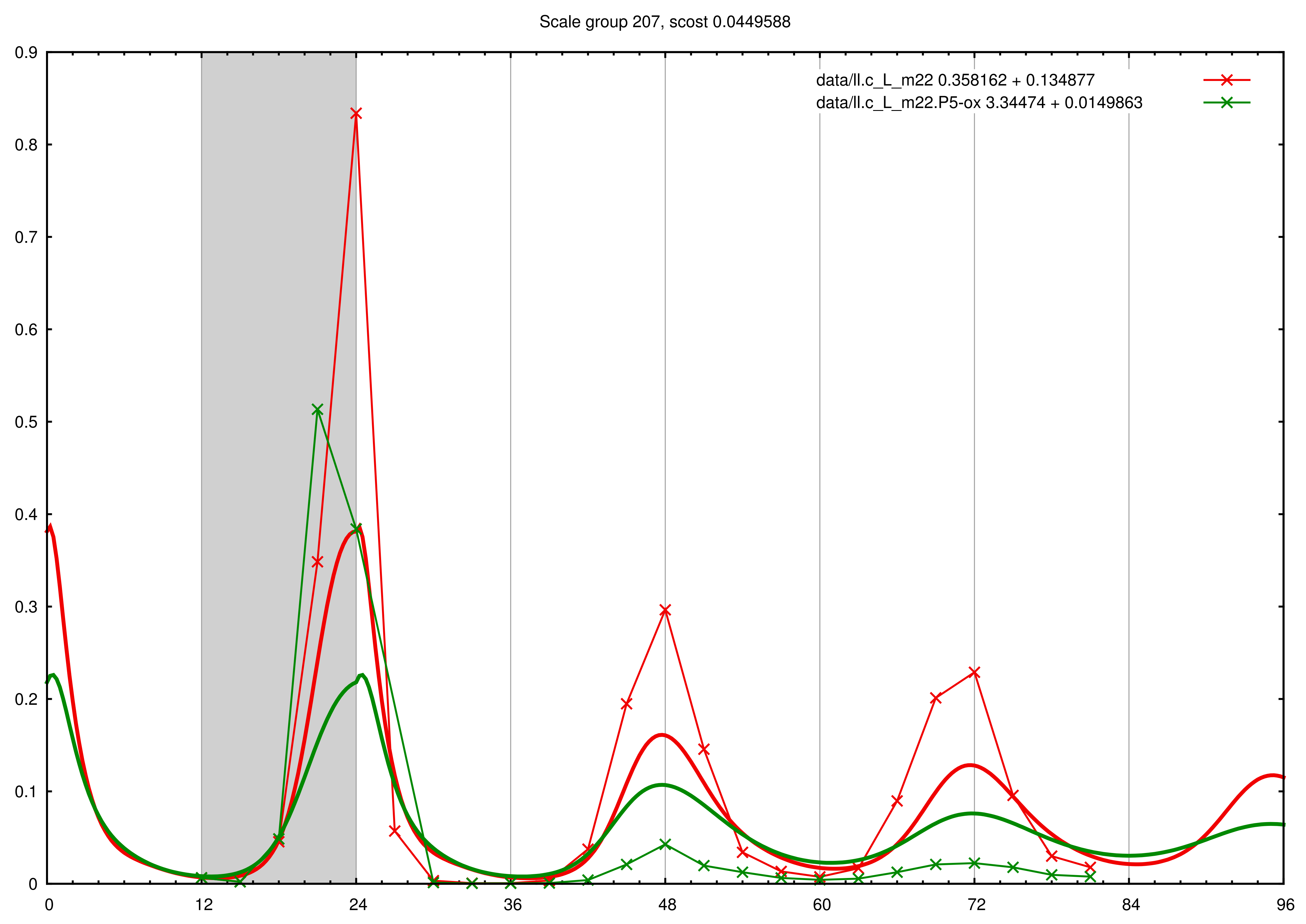

Scale group 207, scost 0.0449588
data/ll.c_L_m22 0.358162 + 0.134877
data/ll.c_L_m22.P5-ox 3.34474 + 0.0149863
0.9
0.8
0.7
0.6
0.5
0.4
0.3
0.2
0.1
0
0
12
24
36
48
60
72
84
96

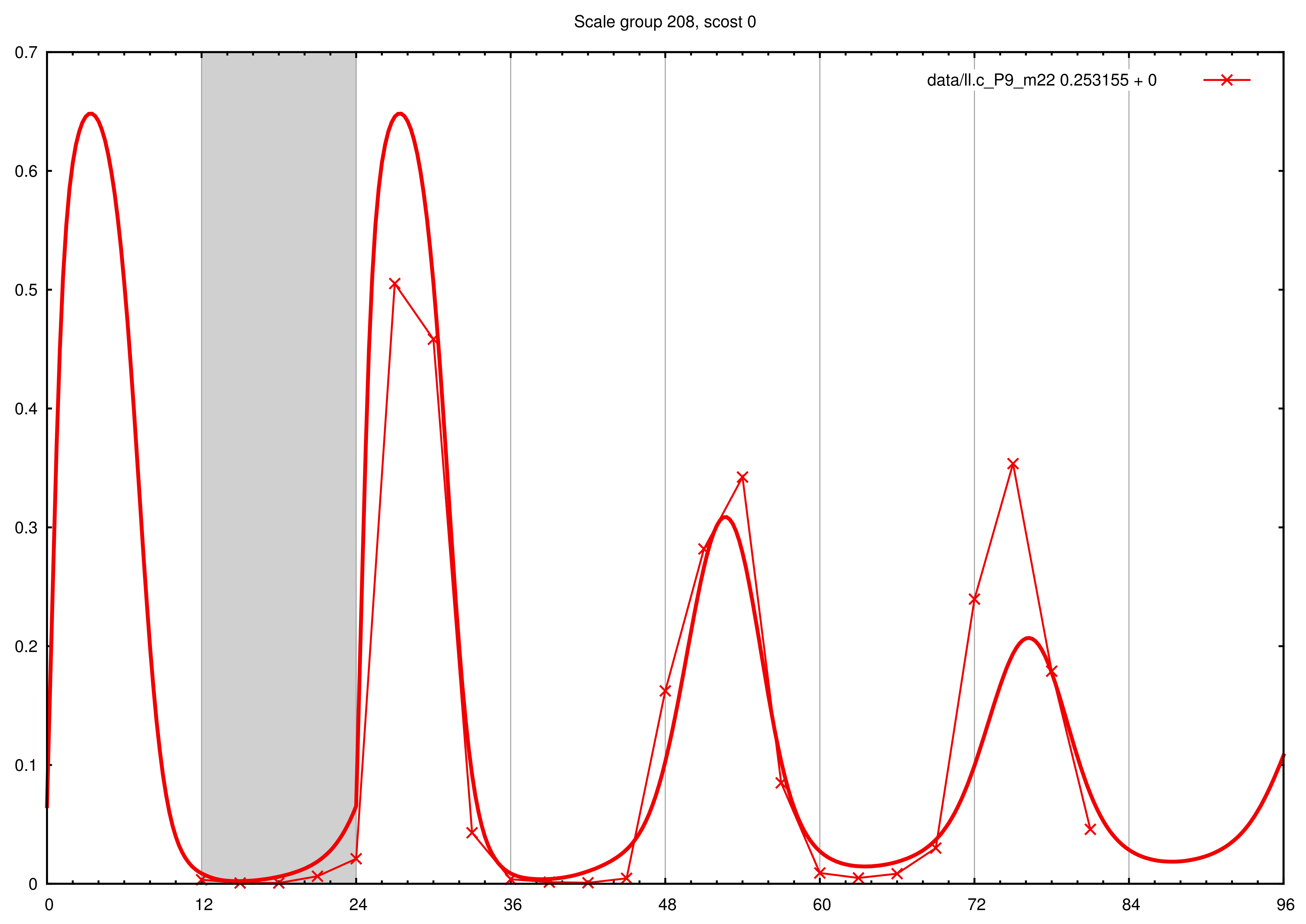
Scale group 208, scost 0
data/ll.c_P9_m22 0.253155 + 0
0.7
0.6
0.5
0.4
0.3
0.2
0.1
0
0
12
24
36
48
60
72
84
96

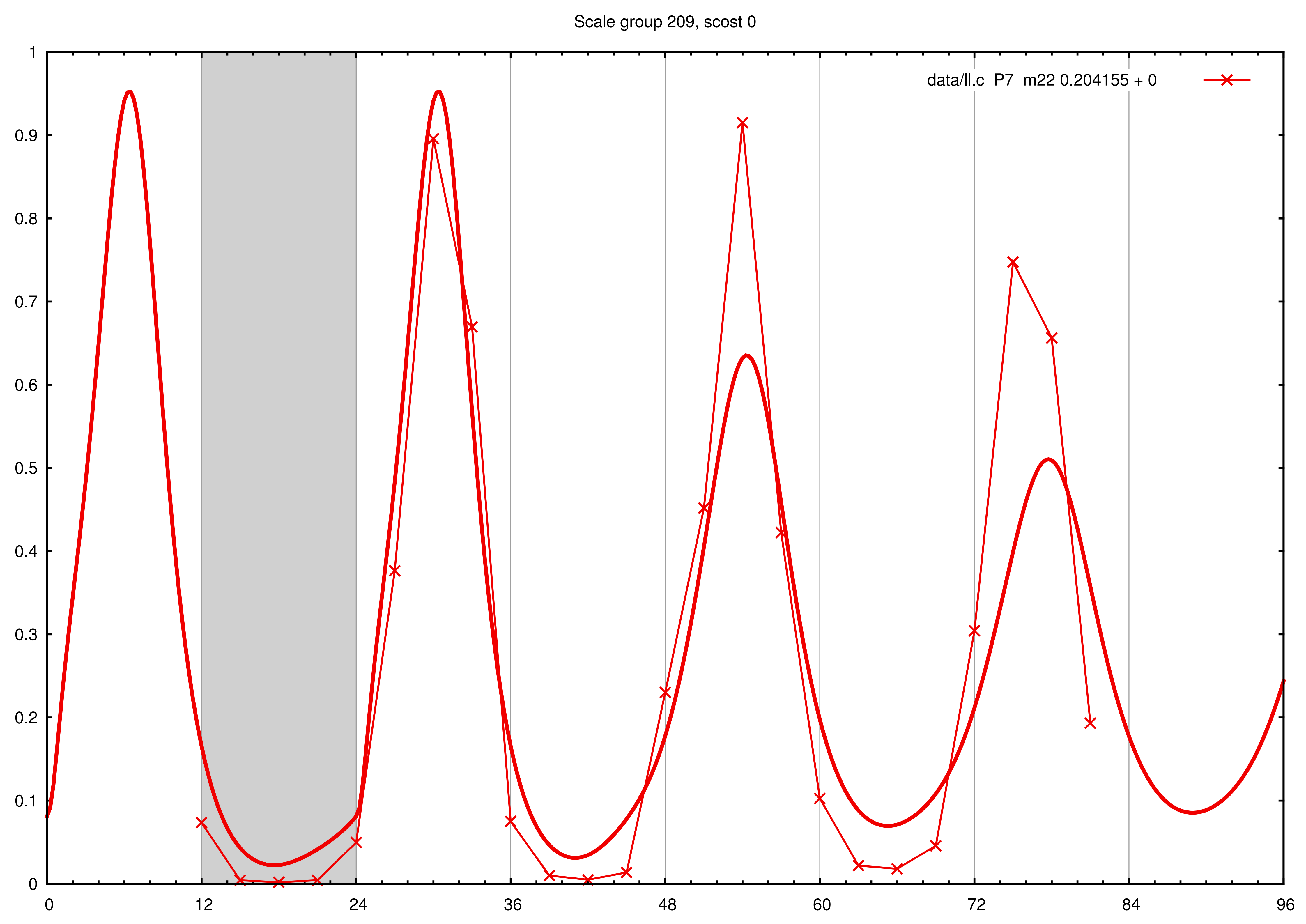

Scale group 209, scost 0
data/ll.c_P7_m22 0.204155 + 0
1
0.9
0.8
0.7
0.6
0.5
0.4
0.3
0.2
0.1
0
0
12
24
36
48
60
72
84
96

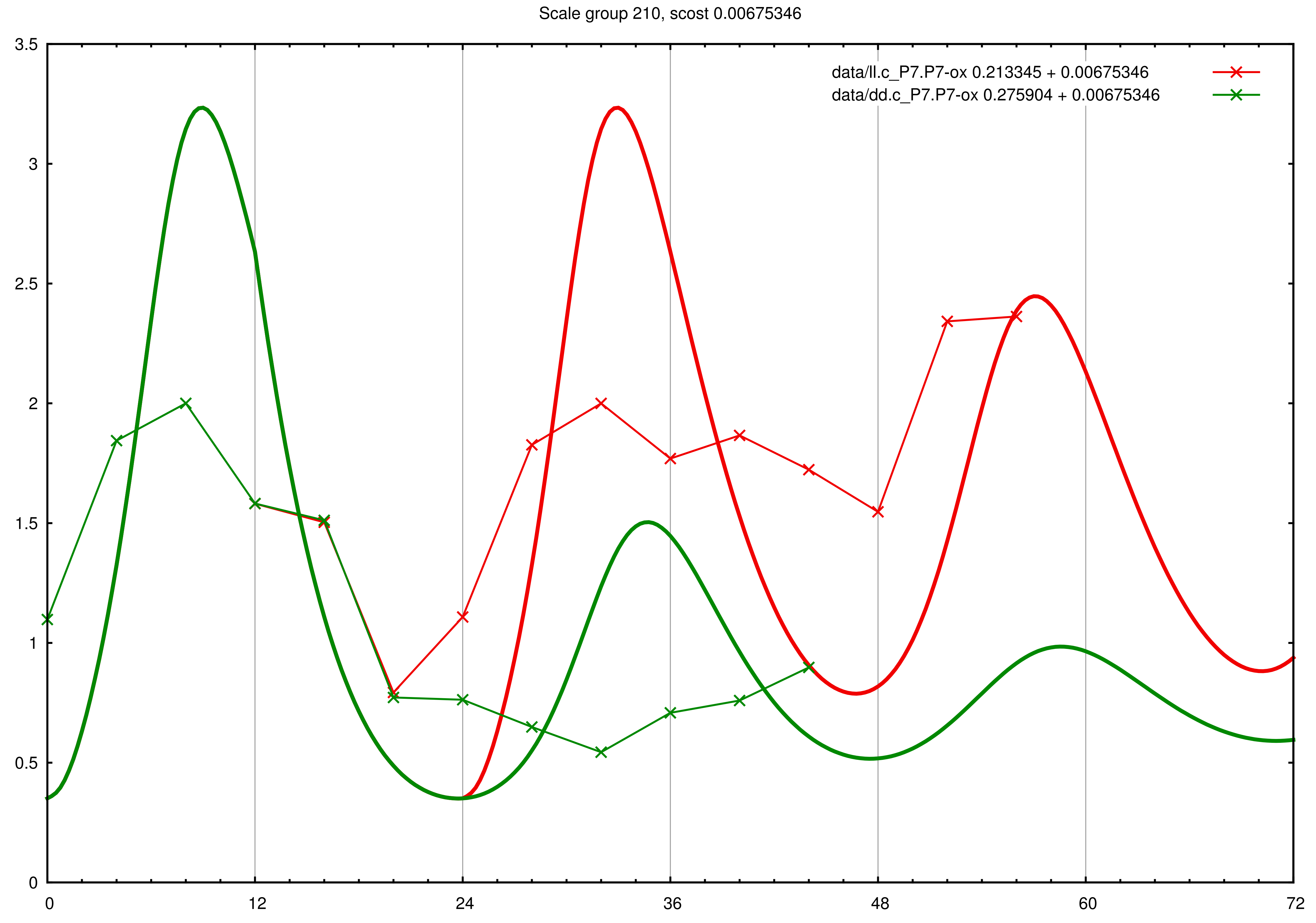
Scale group 210, scost 0.00675346
data/ll.c_P7.P7-ox 0.213345 + 0.00675346
data/dd.c_P7.P7-ox 0.275904 + 0.00675346
3.5
3
2.5
2
1.5
1
0.5
0
0
12
24
36
48
60
72

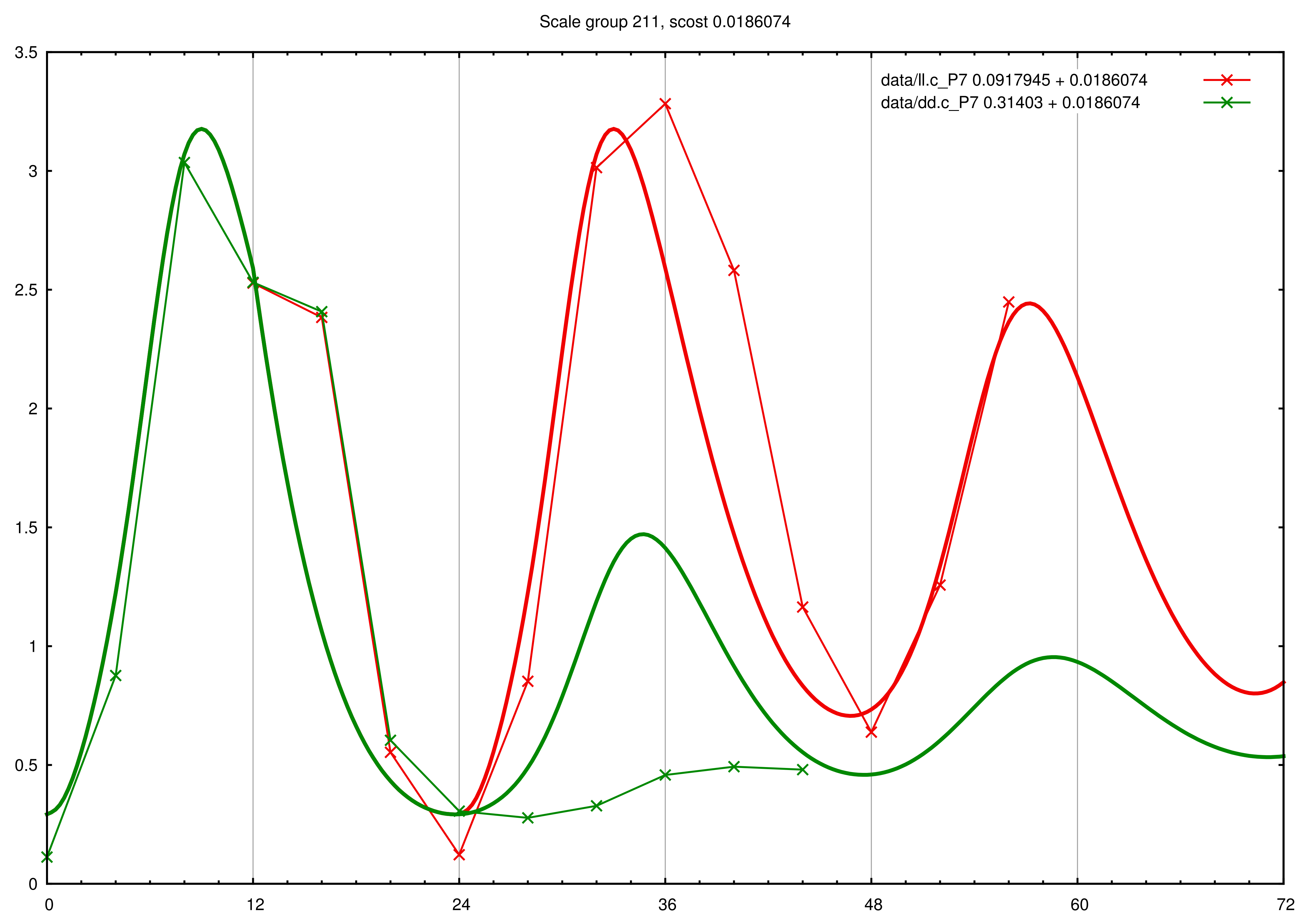

Scale group 211, scost 0.0186074
data/ll.c_P7 0.0917945 + 0.0186074
data/dd.c_P7 0.31403 + 0.0186074
3.5
3
2.5
2
1.5
1
0.5
0
0
12
24
36
48
60
72

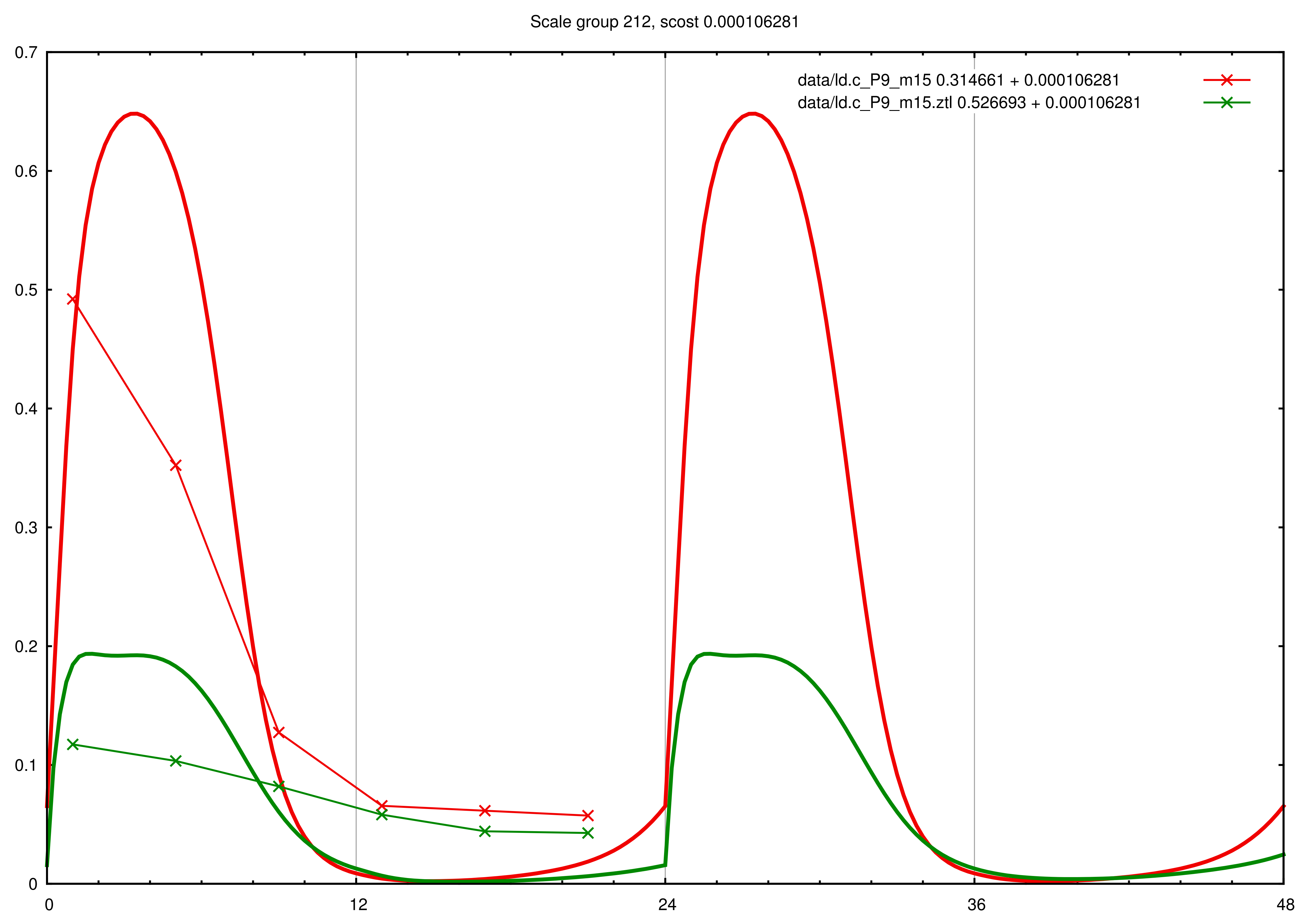

Scale group 212, scost 0.000106281
data/ld.c_P9_m15 0.314661 + 0.000106281
data/ld.c_P9_m15.ztl 0.526693 + 0.000106281
0
0.1
0.2
0.3
0.4
0.5
0.6
0.7
0
12
24
36
48

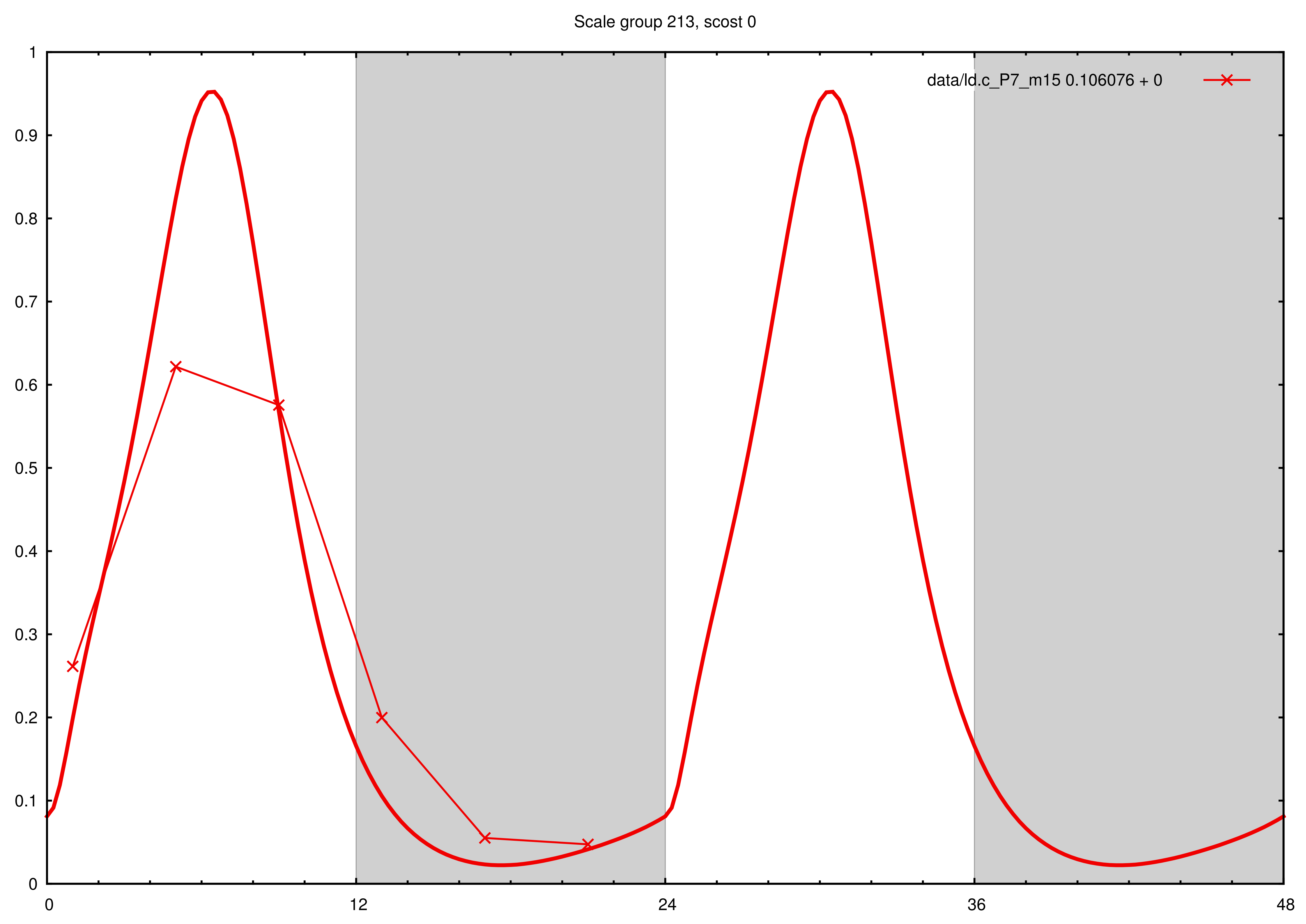

Scale group 213, scost 0
data/ld.c_P7_m15 0.106076 + 0
1
0.9
0.8
0.7
0.6
0.5
0.4
0.3
0.2
0.1
0
0
12
24
36
48

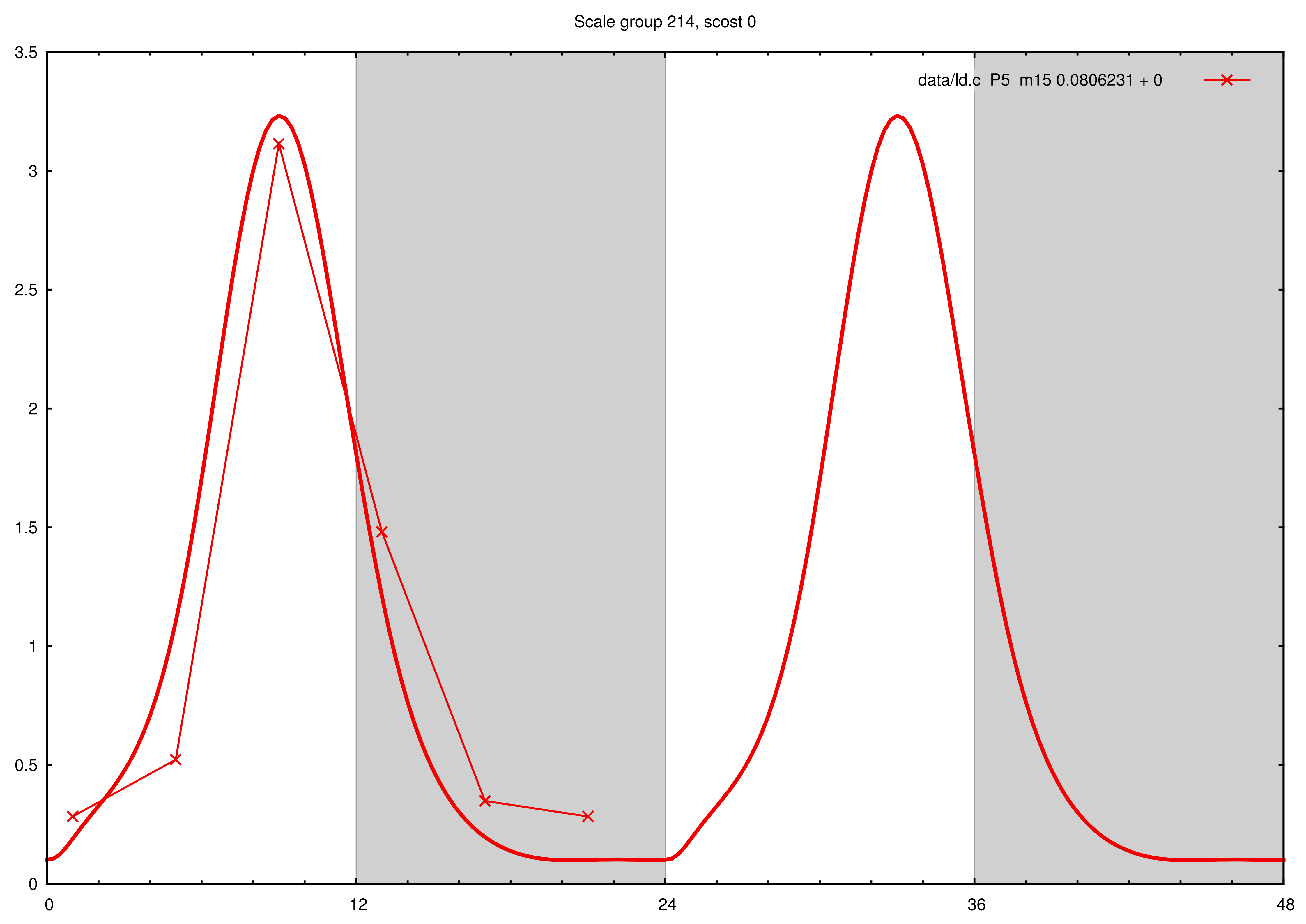
Scale group 214, scost 0
data/ld.c_P5_m15 0.0806231 + 0
3.5
3
2.5
2
1.5
1
0.5
0
0
12
24
36
48

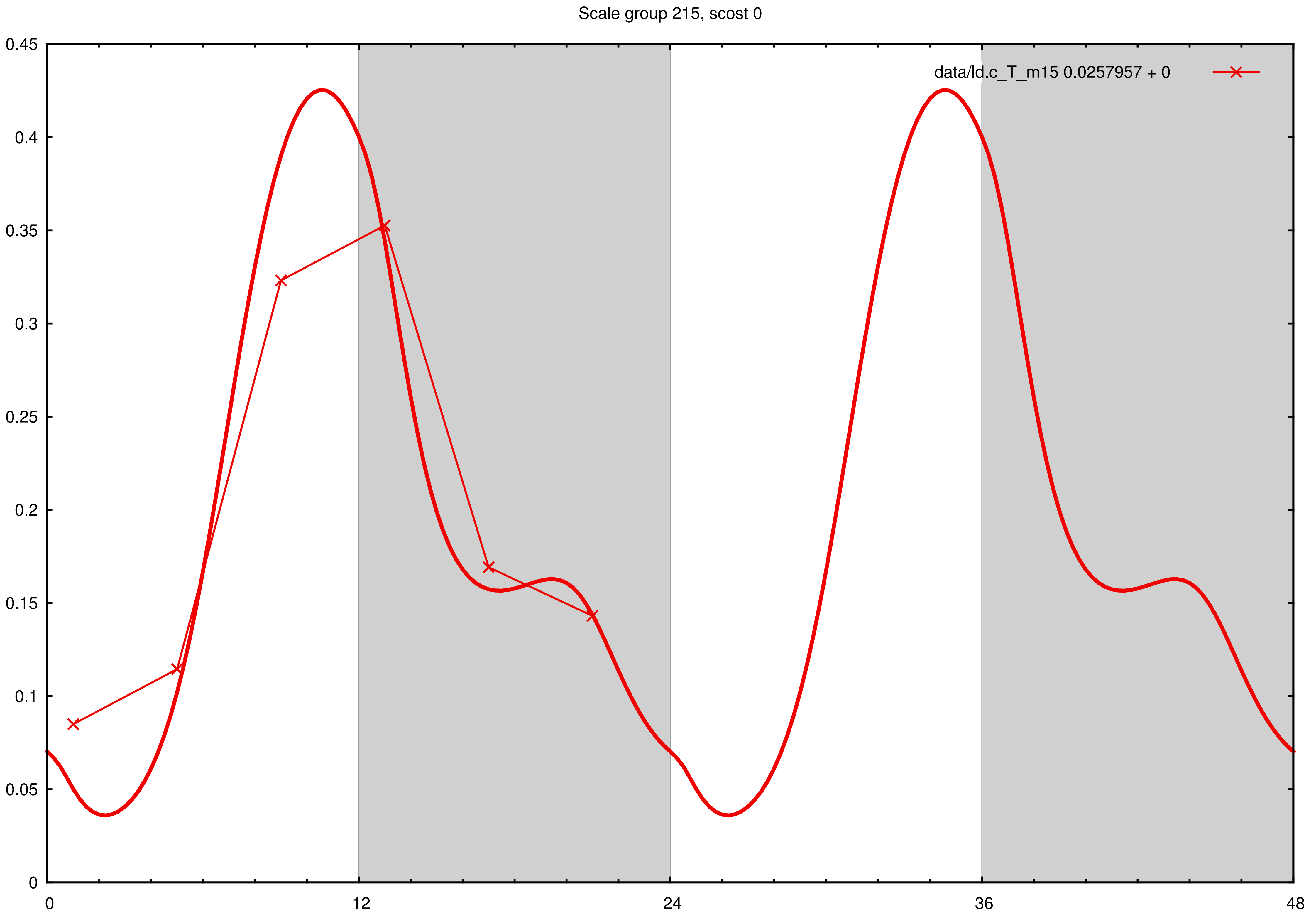

Scale group 215, scost 0
data/ld.c_T_m15 0.0257957 + 0
0.45
0.4
0.35
0.3
0.25
0.2
0.15
0.1
0.05
0
0
12
24
36
48

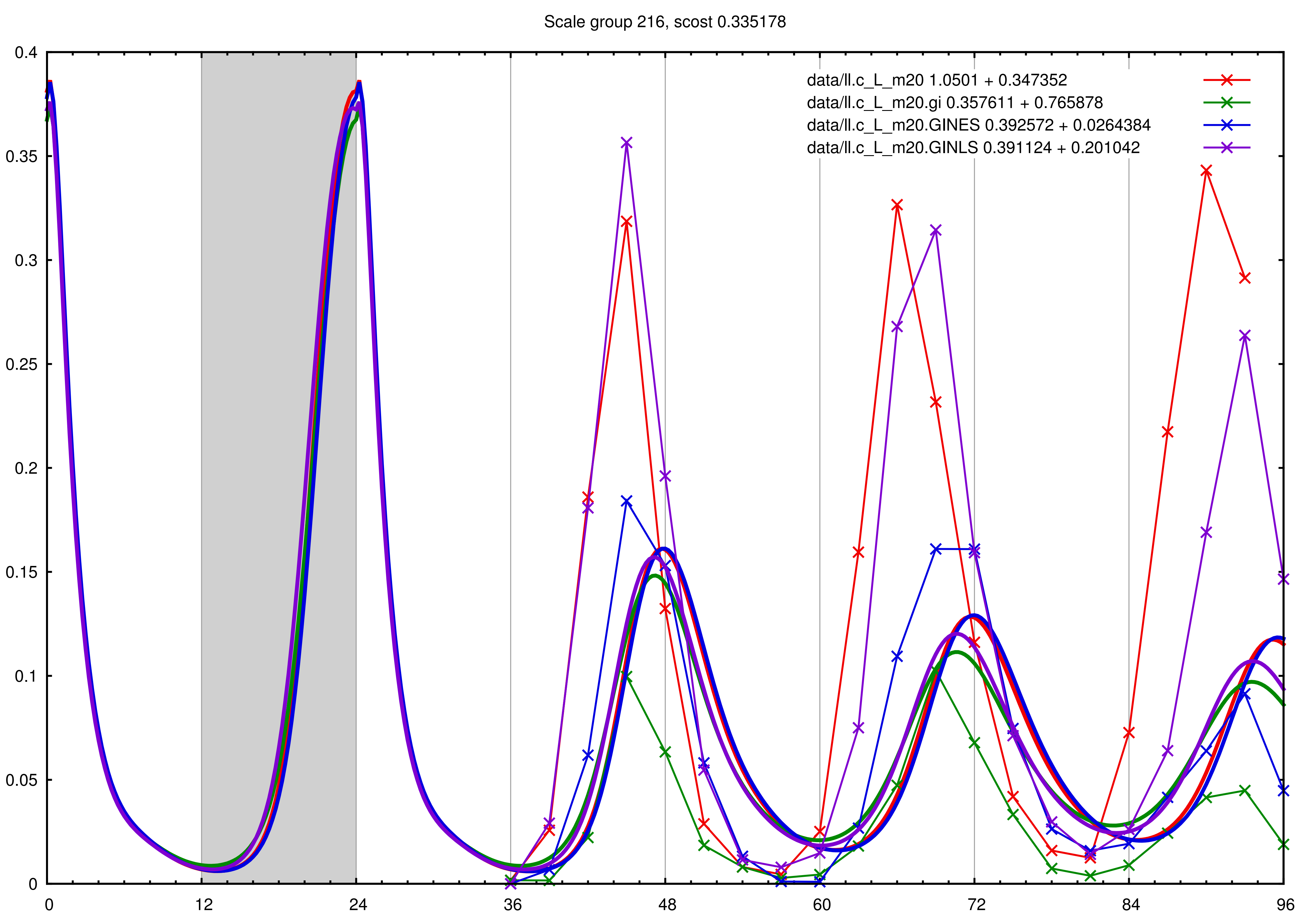

Scale group 216, scost 0.335178
data/ll.c_L_m20 1.0501 + 0.347352
data/ll.c_L_m20.gi 0.357611 + 0.765878
data/ll.c_L_m20.GINES 0.392572 + 0.0264384
data/ll.c_L_m20.GINLS 0.391124 + 0.201042
0.4
0.35
0.3
0.25
0.2
0.15
0.1
0.05
0
0
12
24
36
48
60
72
84
96

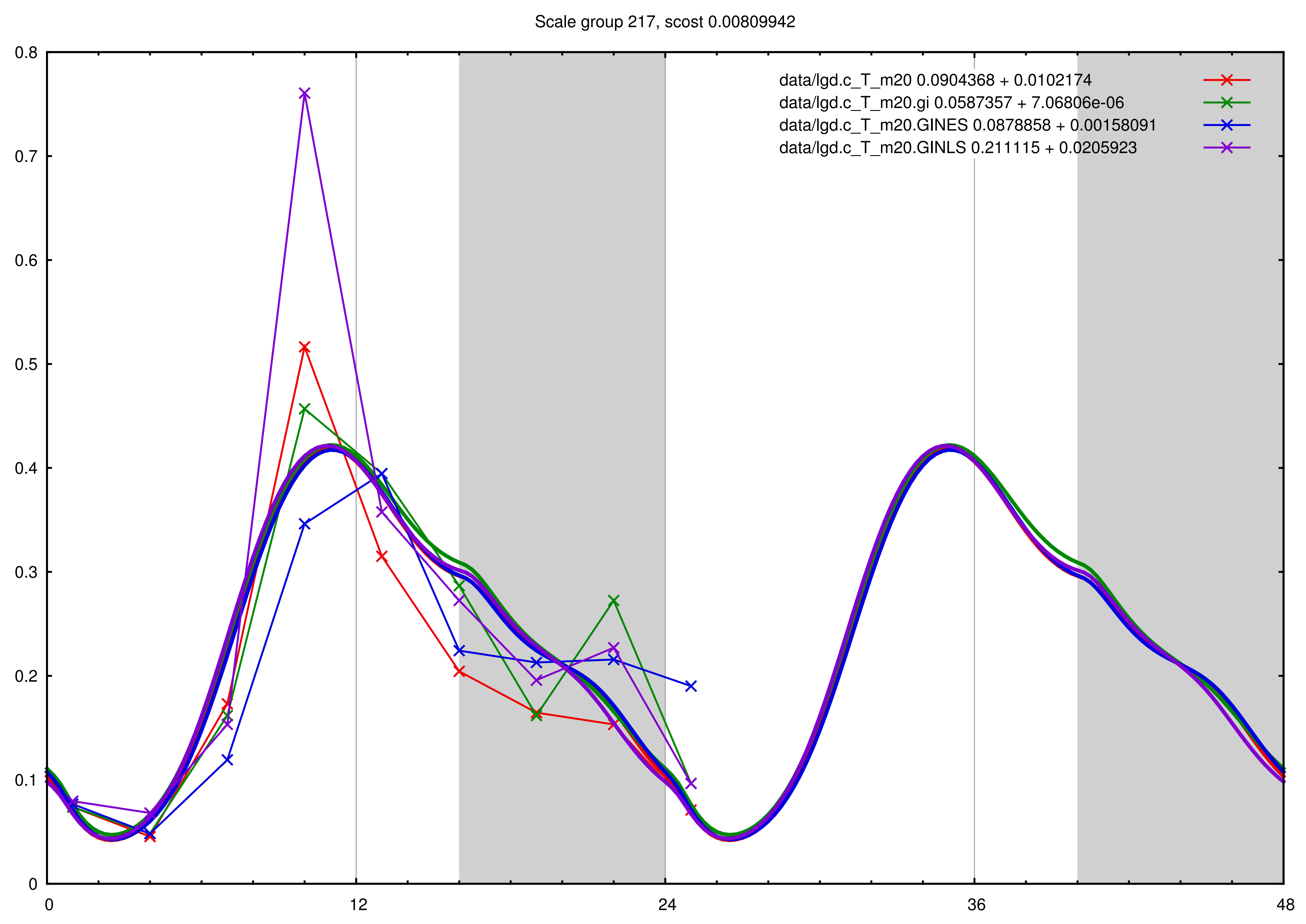

Scale group 217, scost 0.00809942
data/lgd.c_T_m20 0.0904368 + 0.0102174
data/lgd.c_T_m20.gi 0.0587357 + 7.06806e-06
data/lgd.c_T_m20.GINES 0.0878858 + 0.00158091
data/lgd.c_T_m20.GINLS 0.211115 + 0.0205923
0.8
0.7
0.6
0.5
0.4
0.3
0.2
0.1
0
0
12
24
36
48

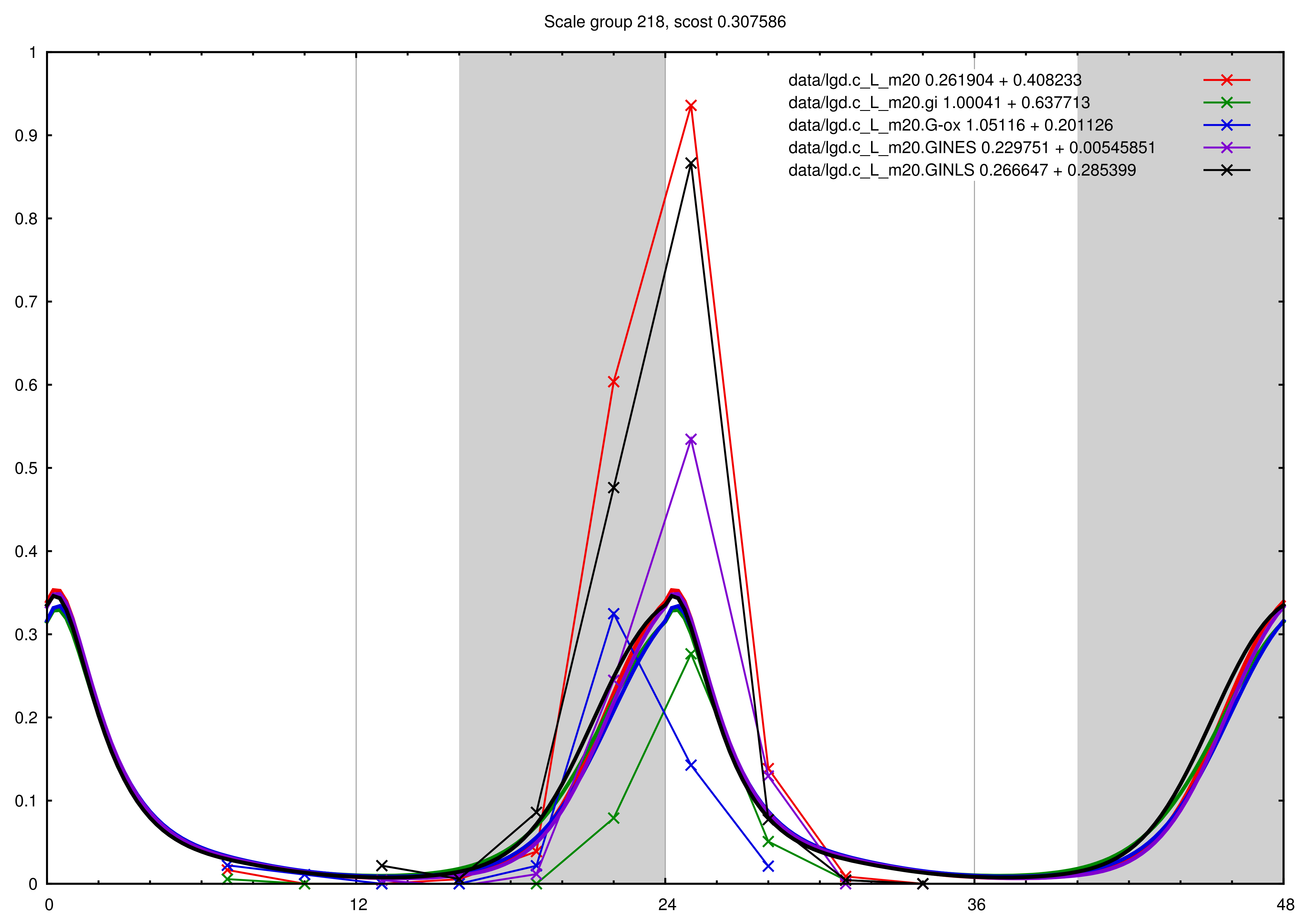

Scale group 218, scost 0.307586
data/lgd.c_L_m20 0.261904 + 0.408233
data/lgd.c_L_m20.gi 1.00041 + 0.637713
data/lgd.c_L_m20.G-ox 1.05116 + 0.201126
data/lgd.c_L_m20.GINES 0.229751 + 0.00545851
data/lgd.c_L_m20.GINLS 0.266647 + 0.285399
0
0.1
0.2
0.3
0.4
0.5
0.6
0.7
0.8
0.9
1
0
12
24
36
48

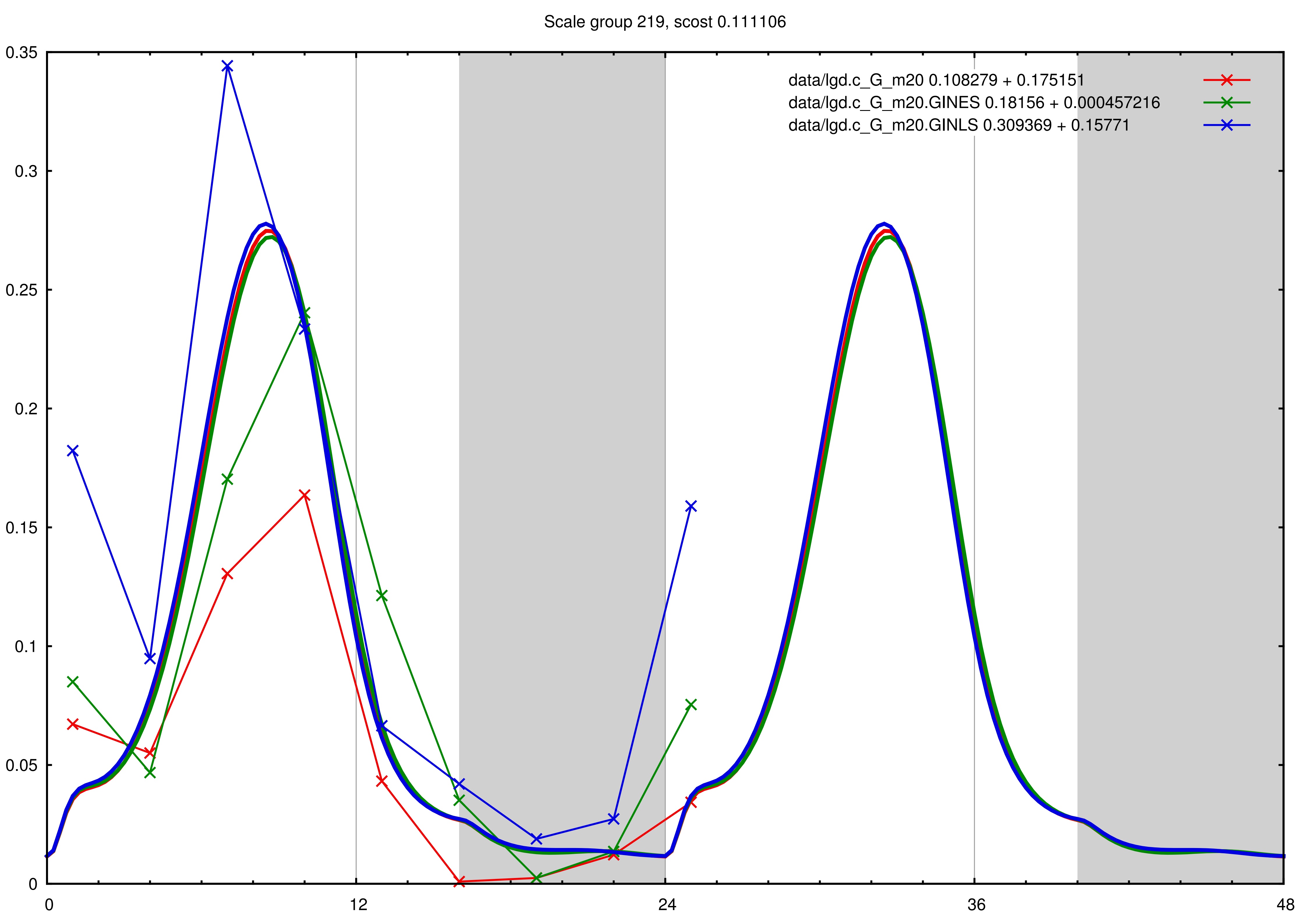

Scale group 219, scost 0.111106
data/lgd.c_G_m20 0.108279 + 0.175151
data/lgd.c_G_m20.GINES 0.18156 + 0.000457216
data/lgd.c_G_m20.GINLS 0.309369 + 0.15771
0.35
0.3
0.25
0.2
0.15
0.1
0.05
0
0
12
24
36
48

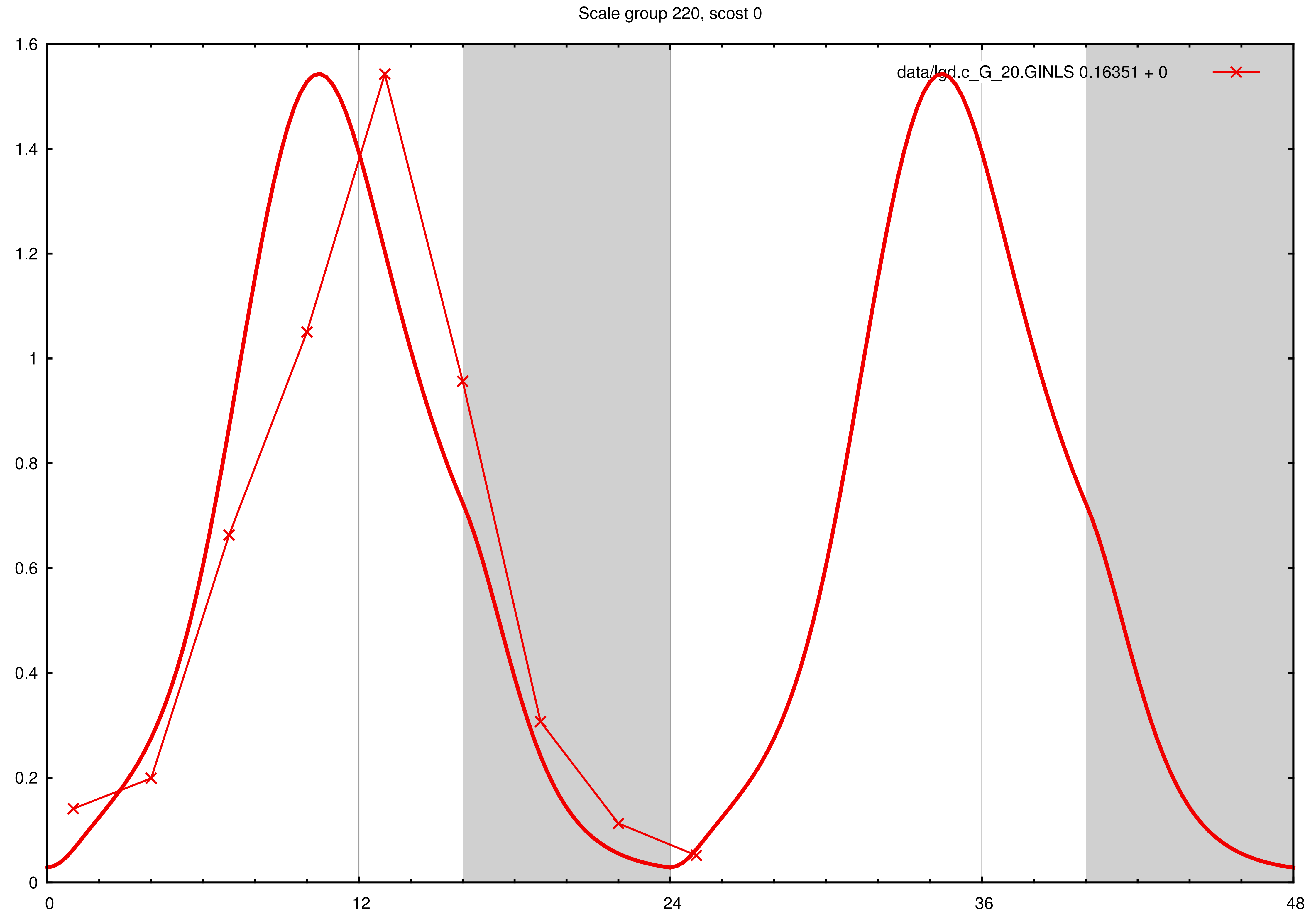

Scale group 220, scost 0
data/lgd.c_G_20.GINLS 0.16351 + 0
1.6
1.4
1.2
1
0.8
0.6
0.4
0.2
0
0
12
24
36
48

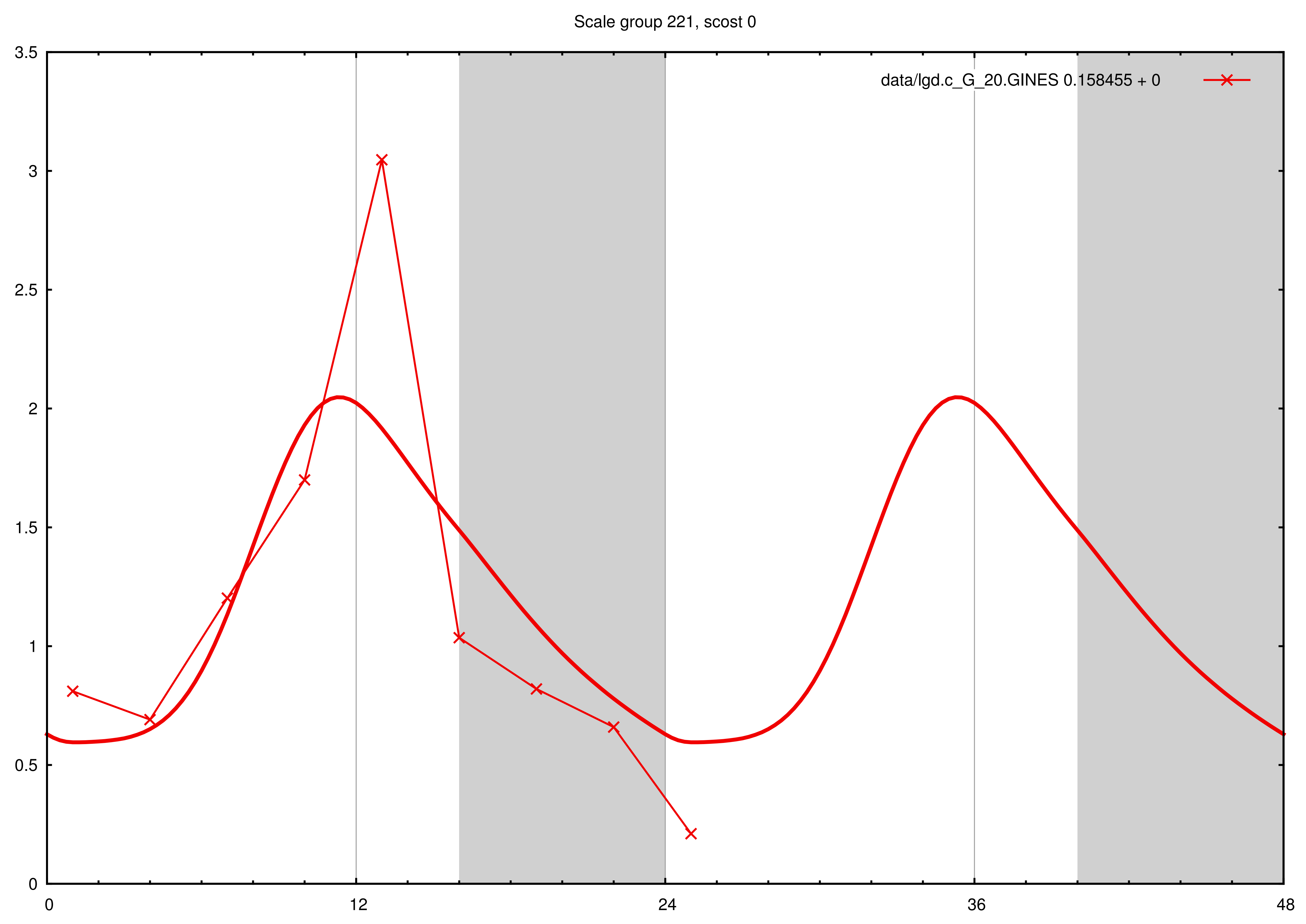

Scale group 221, scost 0
data/lgd.c_G_20.GINES 0.158455 + 0
3.5
3
2.5
2
1.5
1
0.5
0
0
12
24
36
48

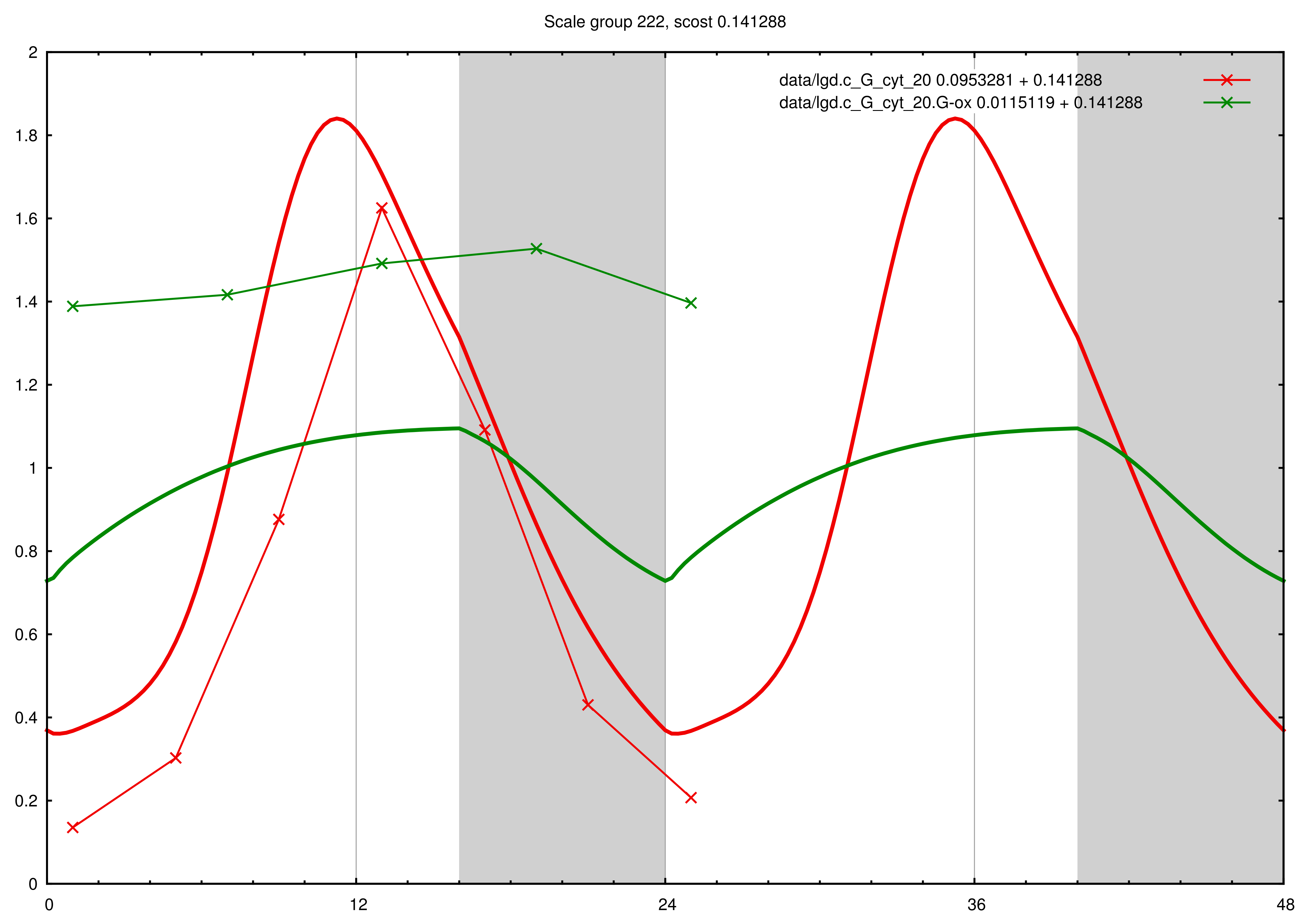

Scale group 222, scost 0.141288
data/lgd.c_G_cyt_20 0.0953281 + 0.141288
data/lgd.c_G_cyt_20.G-ox 0.0115119 + 0.141288
2
1.8
1.6
1.4
1.2
1
0.8
0.6
0.4
0.2
0
0
12
24
36
48

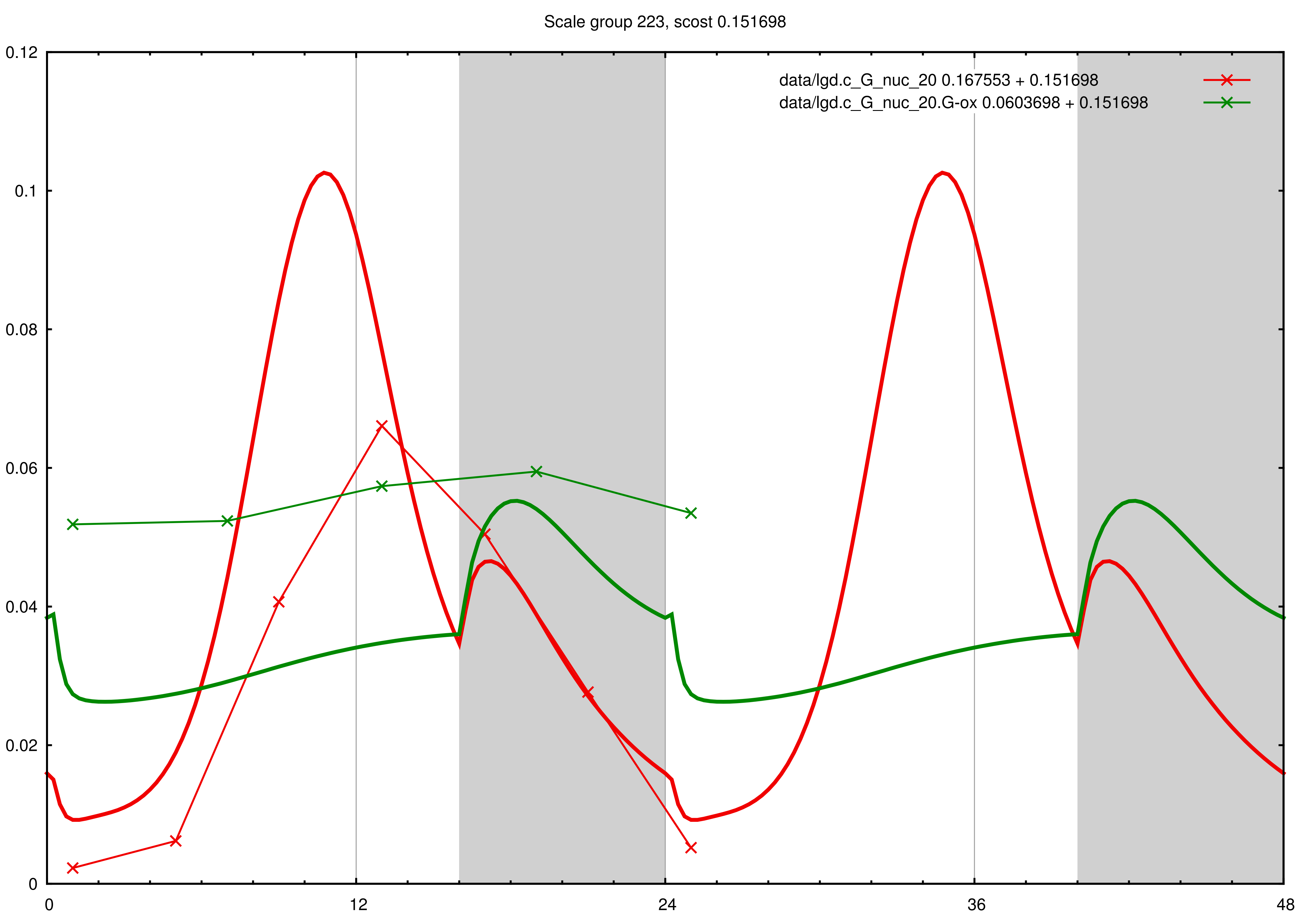

Scale group 223, scost 0.151698
data/lgd.c_G_nuc_20 0.167553 + 0.151698
data/lgd.c_G_nuc_20.G-ox 0.0603698 + 0.151698
0.12
0.1
0.08
0.06
0.04
0.02
0
0
12
24
36
48

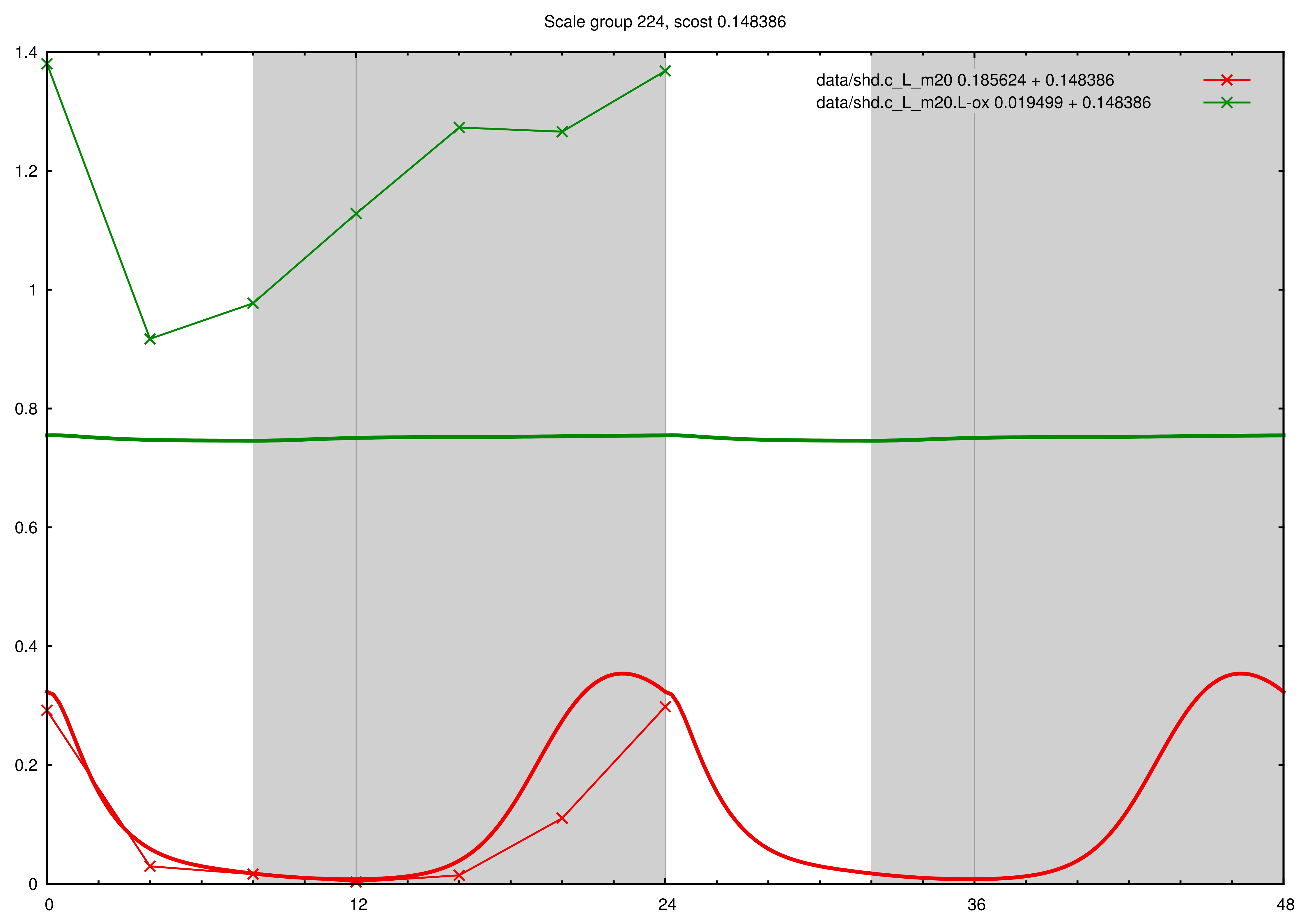

Scale group 224, scost 0.148386
data/shd.c_L_m20 0.185624 + 0.148386
data/shd.c_L_m20.L-ox 0.019499 + 0.148386
1.4
1.2
1
0.8
0.6
0.4
0.2
0
0
12
24
36
48

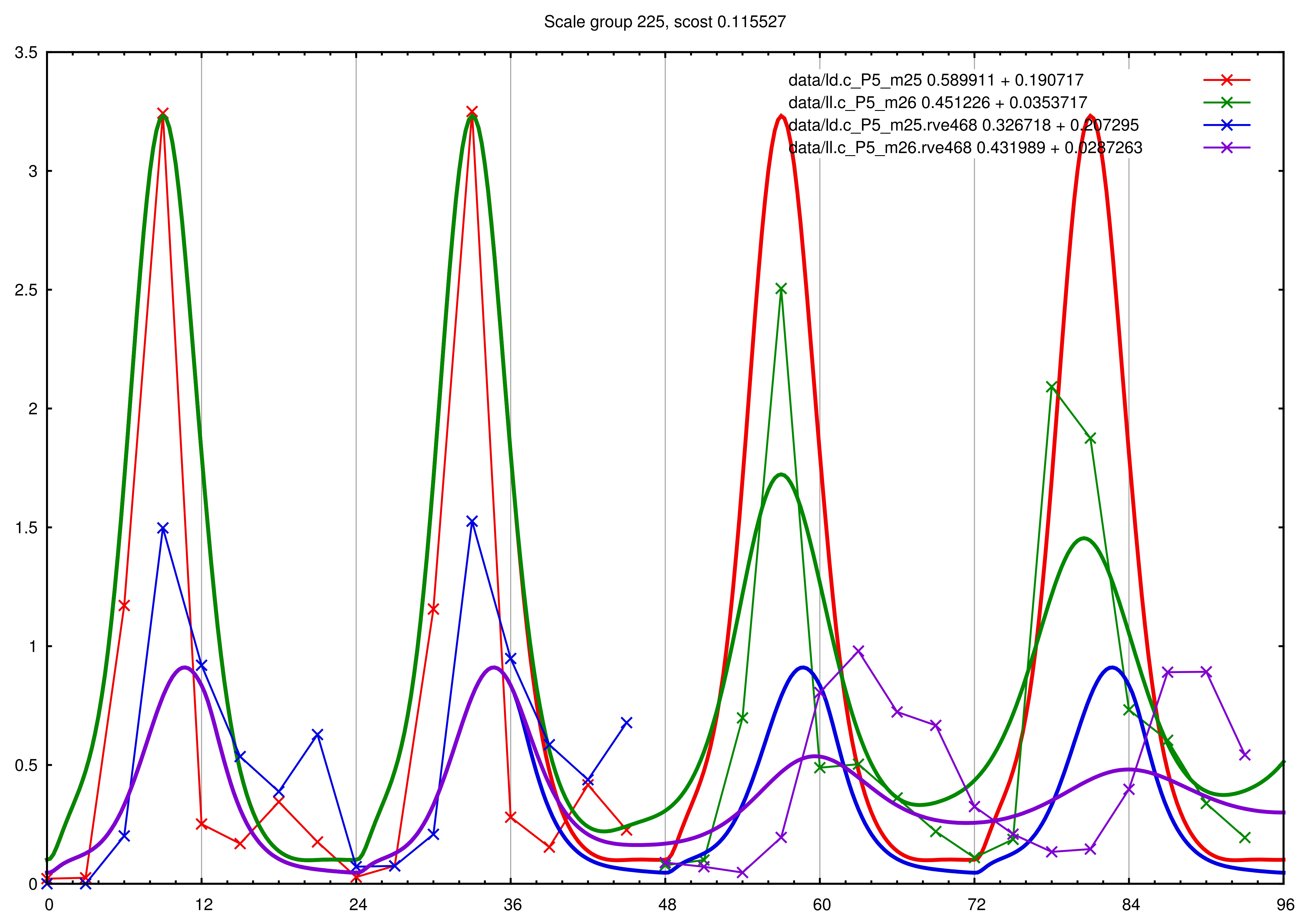
Scale group 225, scost 0.115527
data/ld.c_P5_m25 0.589911 + 0.190717
data/ll.c_P5_m26 0.451226 + 0.0353717
data/ld.c_P5_m25.rve468 0.326718 + 0.207295
data/ll.c_P5_m26.rve468 0.431989 + 0.0287263
0
0.5
1
1.5
2
2.5
3
3.5
0
12
24
36
48
60
72
84
96

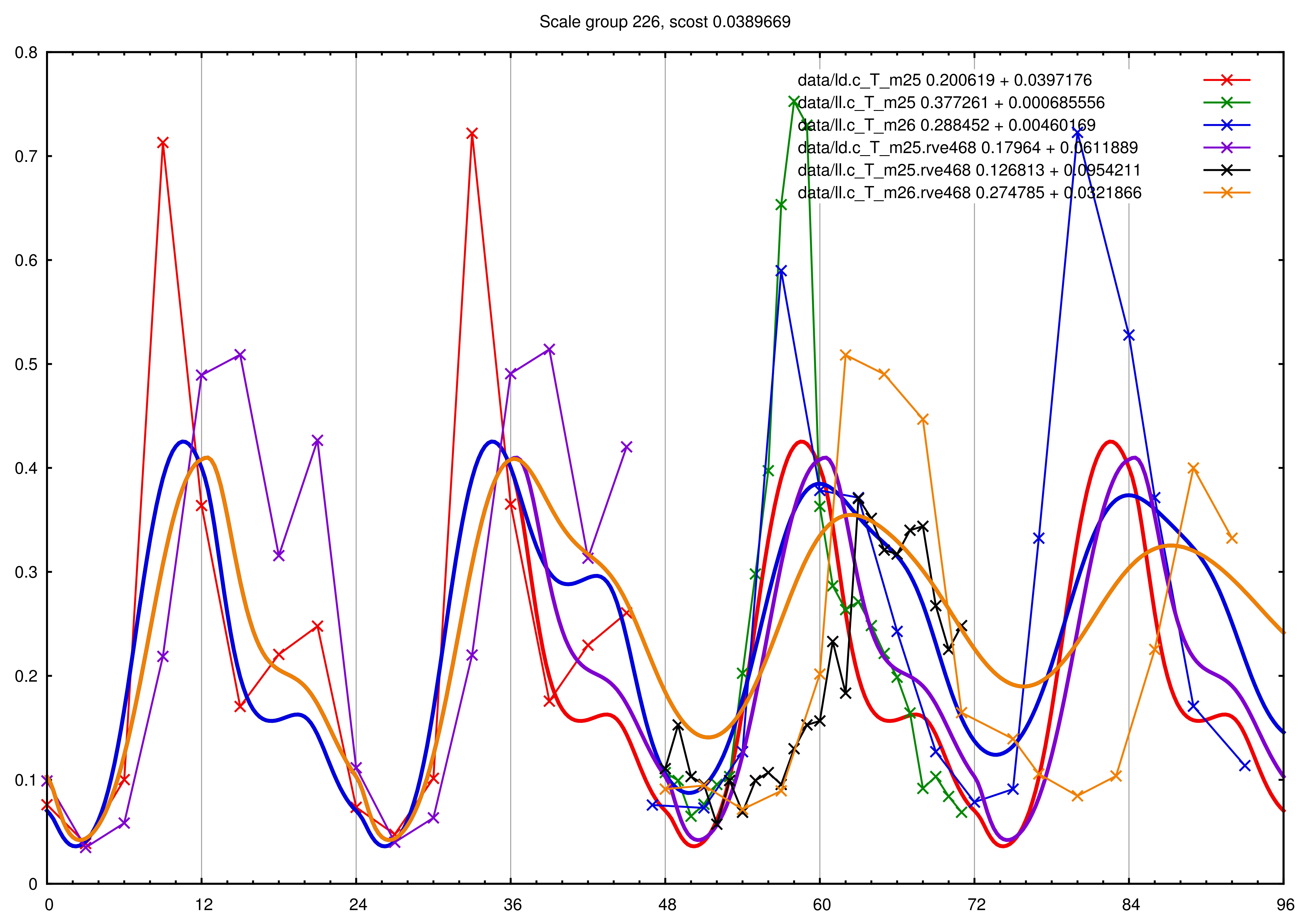
Scale group 226, scost 0.0389669
data/ld.c_T_m25 0.200619 + 0.0397176
data/ll.c_T_m25 0.377261 + 0.000685556
data/ll.c_T_m26 0.288452 + 0.00460169
data/ld.c_T_m25.rve468 0.17964 + 0.0611889
data/ll.c_T_m25.rve468 0.126813 + 0.0954211
data/ll.c_T_m26.rve468 0.274785 + 0.0321866
0
0.1
0.2
0.3
0.4
0.5
0.6
0.7
0.8
0
12
24
36
48
60
72
84
96

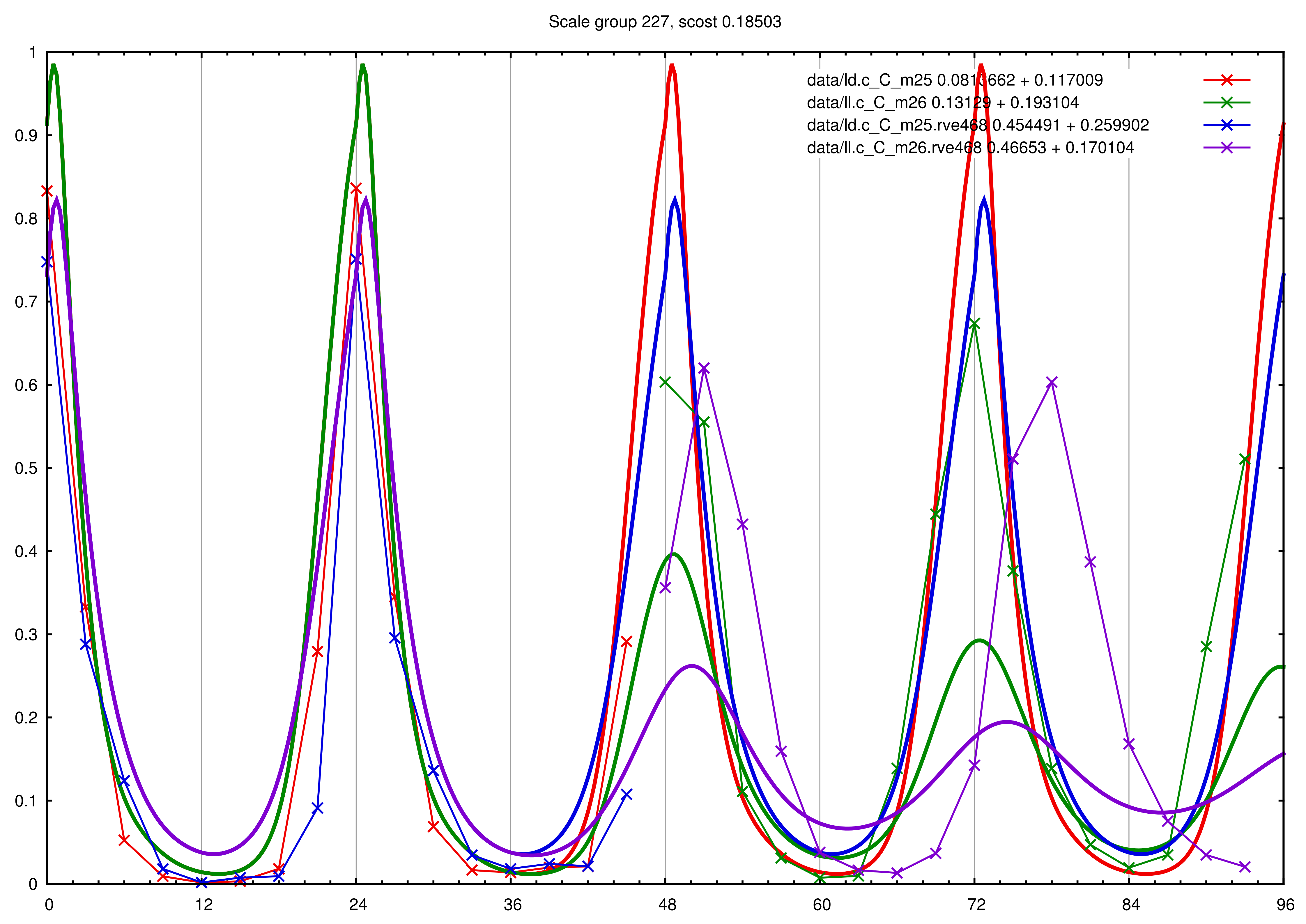

Scale group 227, scost 0.18503
data/ld.c_C_m25 0.0813662 + 0.117009
data/ll.c_C_m26 0.13129 + 0.193104
data/ld.c_C_m25.rve468 0.454491 + 0.259902
data/ll.c_C_m26.rve468 0.46653 + 0.170104
1
0.9
0.8
0.7
0.6
0.5
0.4
0.3
0.2
0.1
0
0
12
24
36
48
60
72
84
96

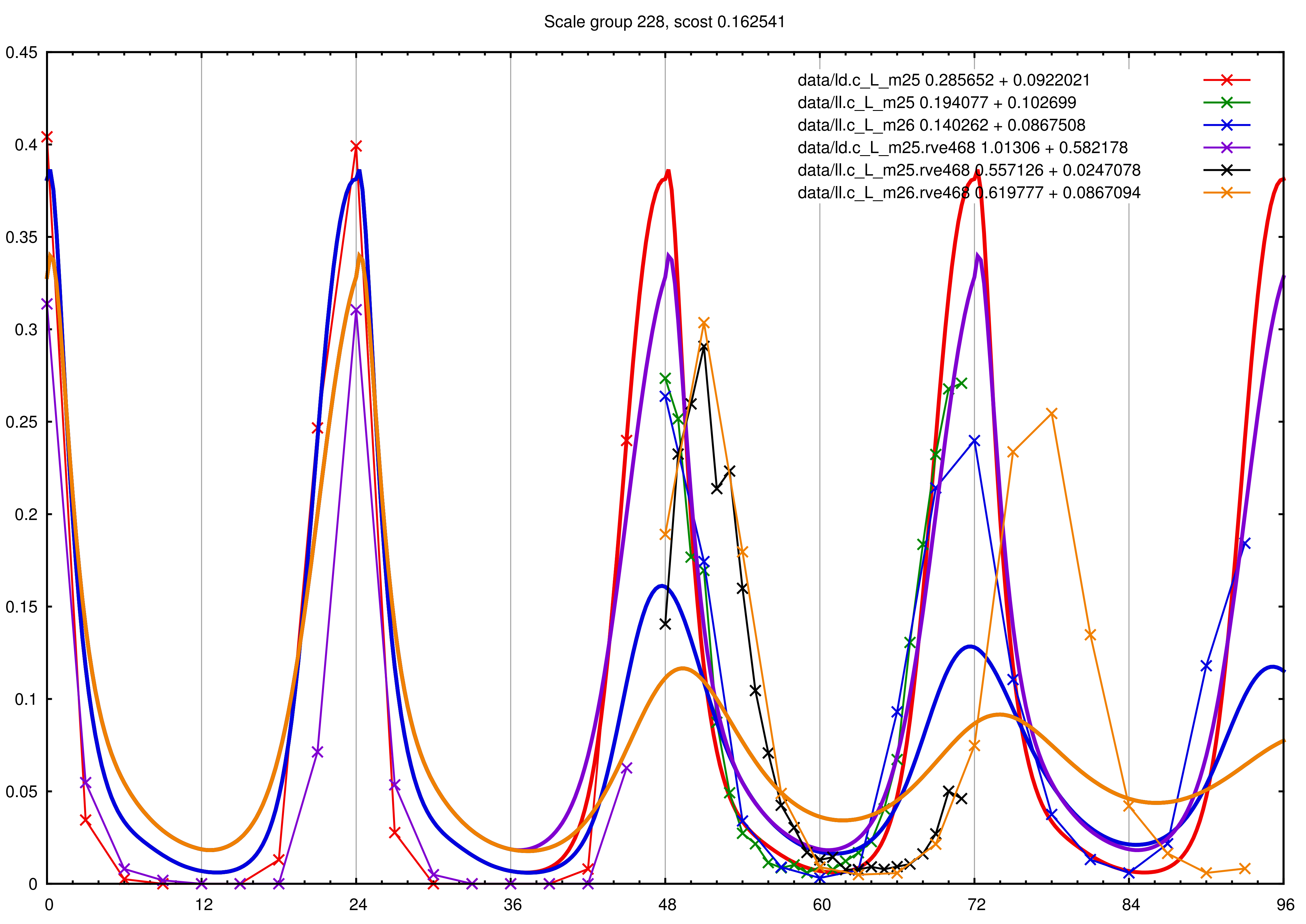

Scale group 228, scost 0.162541
0.45
0.4
0.35
0.3
0.25
0.2
0.15
0.1
0.05
0
0
12
24
36
48
60
72
84
96
data/ld.c_L_m25 0.285652 + 0.0922021
data/ll.c_L_m25 0.194077 + 0.102699
data/ll.c_L_m26 0.140262 + 0.0867508
data/ld.c_L_m25.rve468 1.01306 + 0.582178
data/ll.c_L_m25.rve468 0.557126 + 0.0247078
data/ll.c_L_m26.rve468 0.619777 + 0.0867094

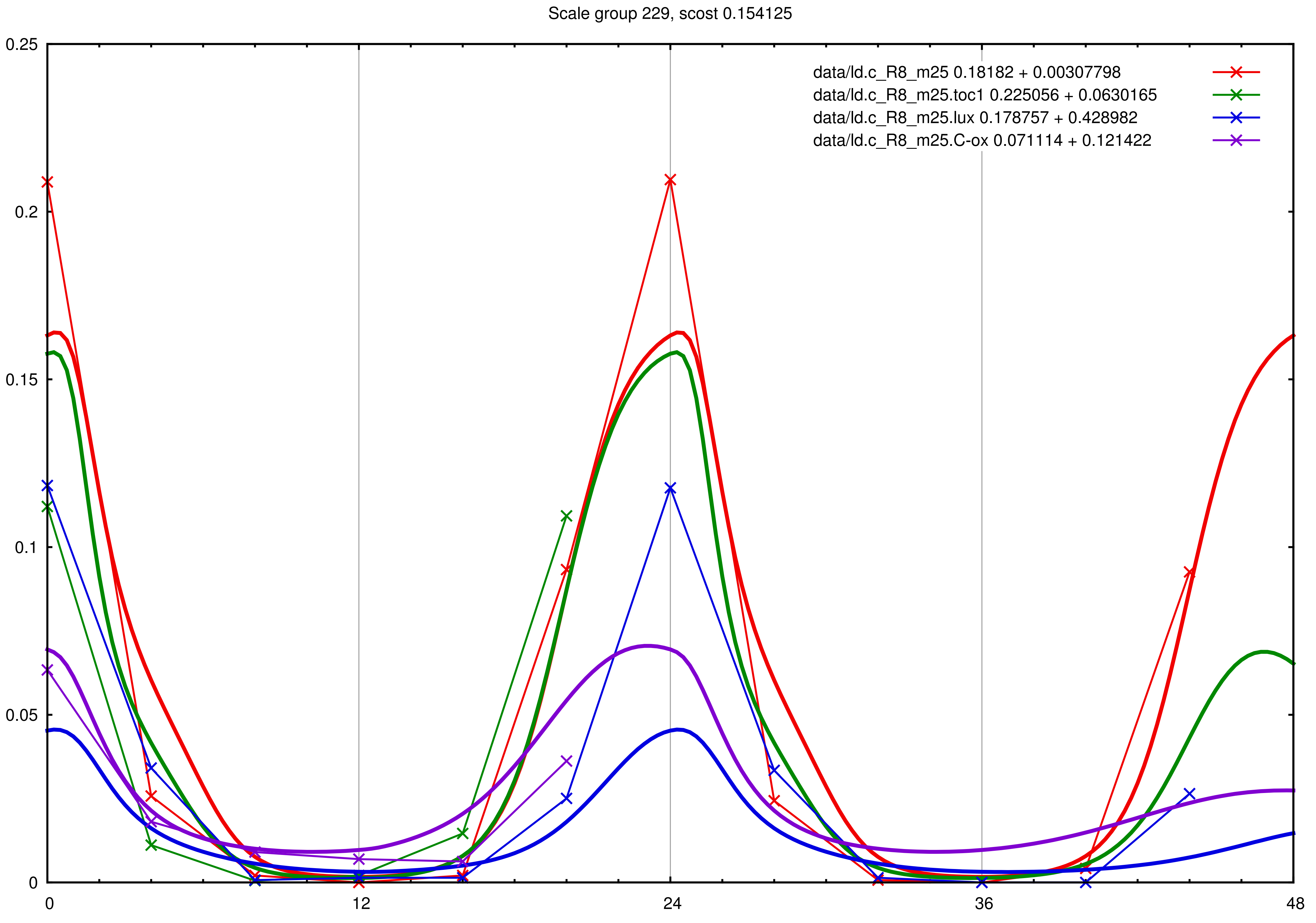
Scale group 229, scost 0.154125
data/ld.c_R8_m25 0.18182 + 0.00307798
data/ld.c_R8_m25.toc1 0.225056 + 0.0630165
data/ld.c_R8_m25.lux 0.178757 + 0.428982
data/ld.c_R8_m25.C-ox 0.071114 + 0.121422
0.25
0.2
0.15
0.1
0.05
0
0
12
24
36
48

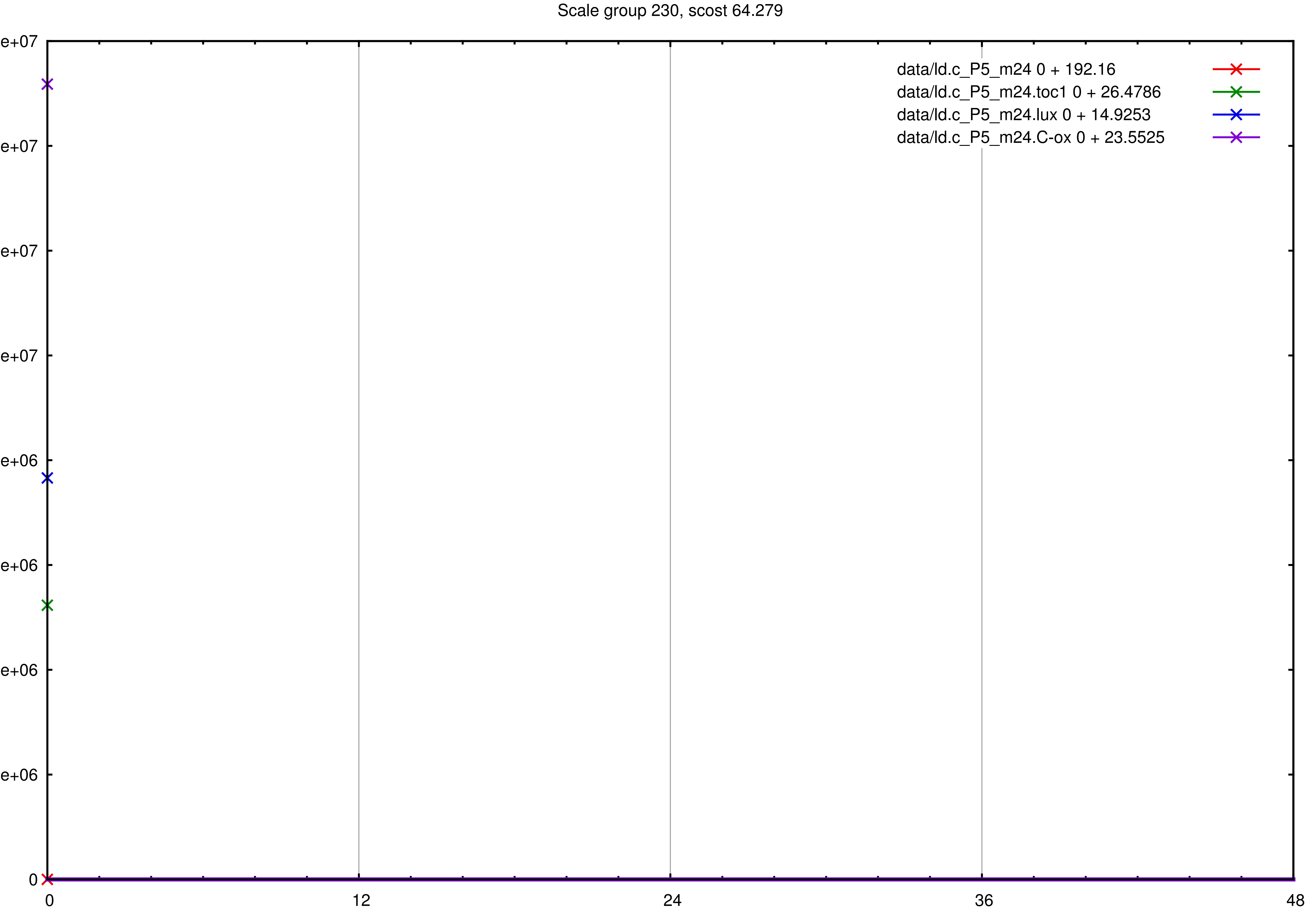
Scale group 230, scost 64.279
data/ld.c_P5_m24 0 + 192.16
data/ld.c_P5_m24.toc1 0 + 26.4786
data/ld.c_P5_m24.lux 0 + 14.9253
data/ld.c_P5_m24.C-ox 0 + 23.5525
e+07
e+07
e+07
e+07
e+06
e+06
e+06
e+06
0
0
12
24
36
48

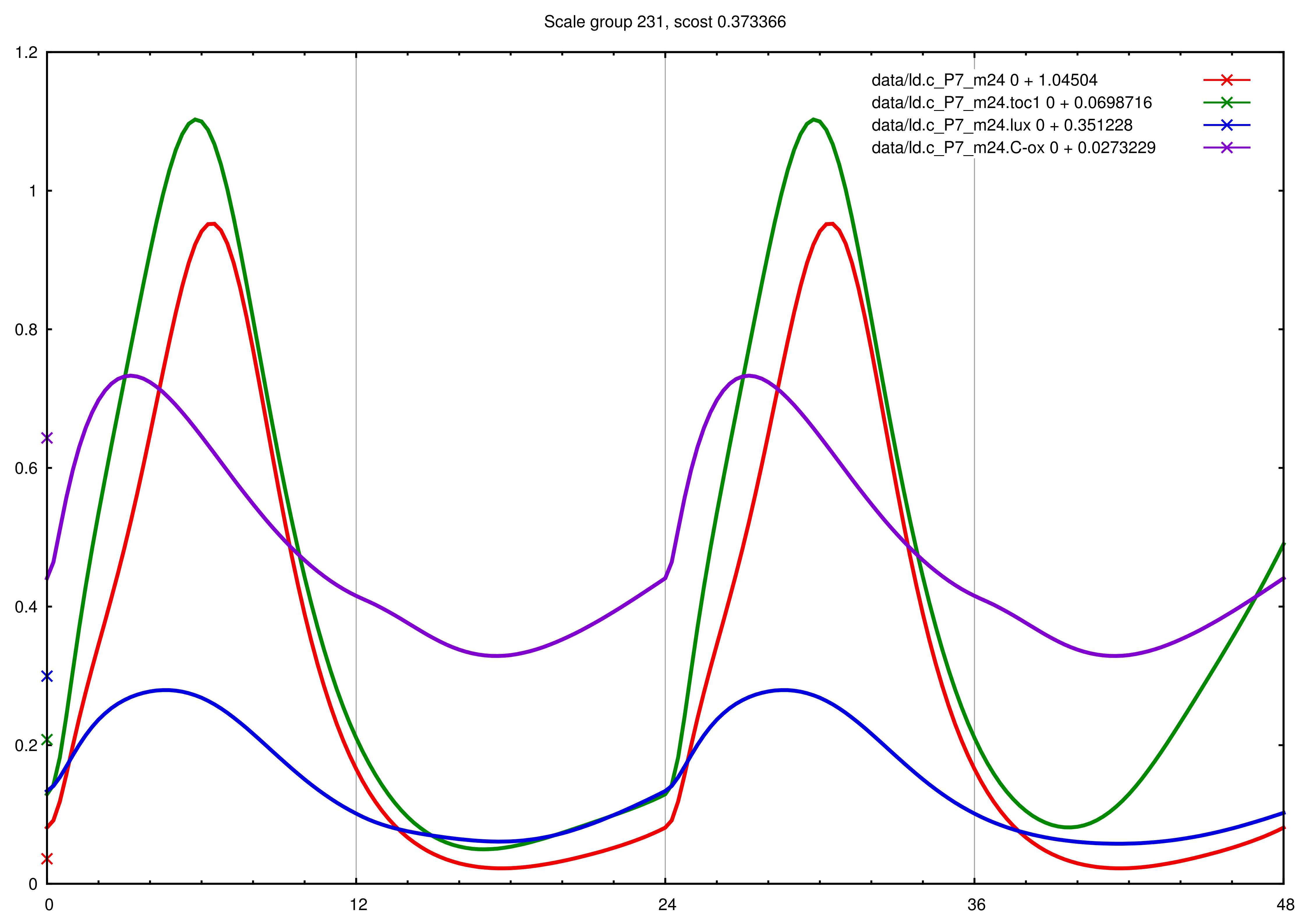
Scale group 231, scost 0.373366
data/ld.c_P7_m24 0 + 1.04504
data/ld.c_P7_m24.toc1 0 + 0.0698716
data/ld.c_P7_m24.lux 0 + 0.351228
data/ld.c_P7_m24.C-ox 0 + 0.0273229
1.2
1
0.8
0.6
0.4
0.2
0
0
12
24
36
48

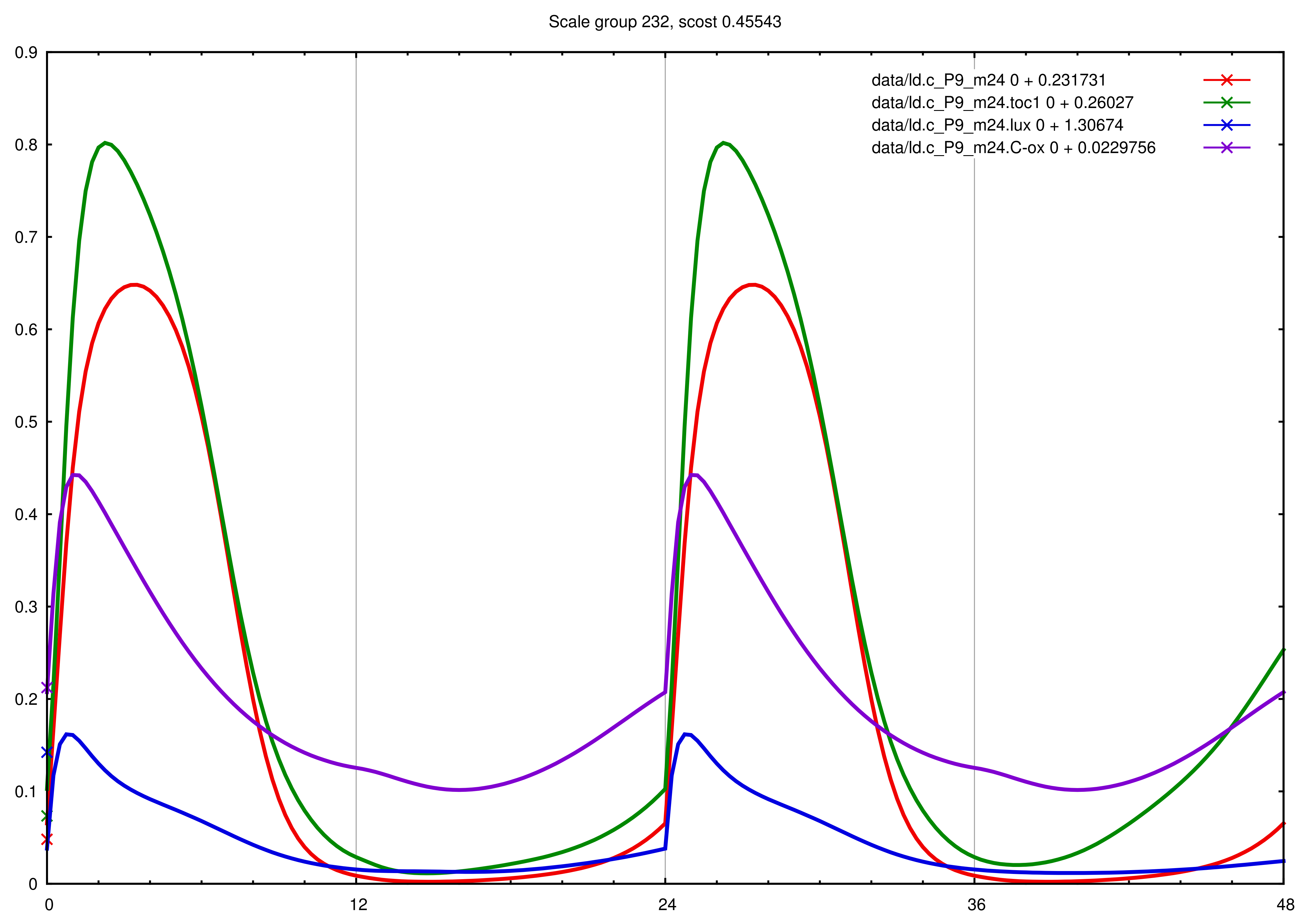
Scale group 232, scost 0.45543
data/ld.c_P9_m24 0 + 0.231731
data/ld.c_P9_m24.toc1 0 + 0.26027
data/ld.c_P9_m24.lux 0 + 1.30674
data/ld.c_P9_m24.C-ox 0 + 0.0229756
0
0.1
0.2
0.3
0.4
0.5
0.6
0.7
0.8
0.9
0
12
24
36
48

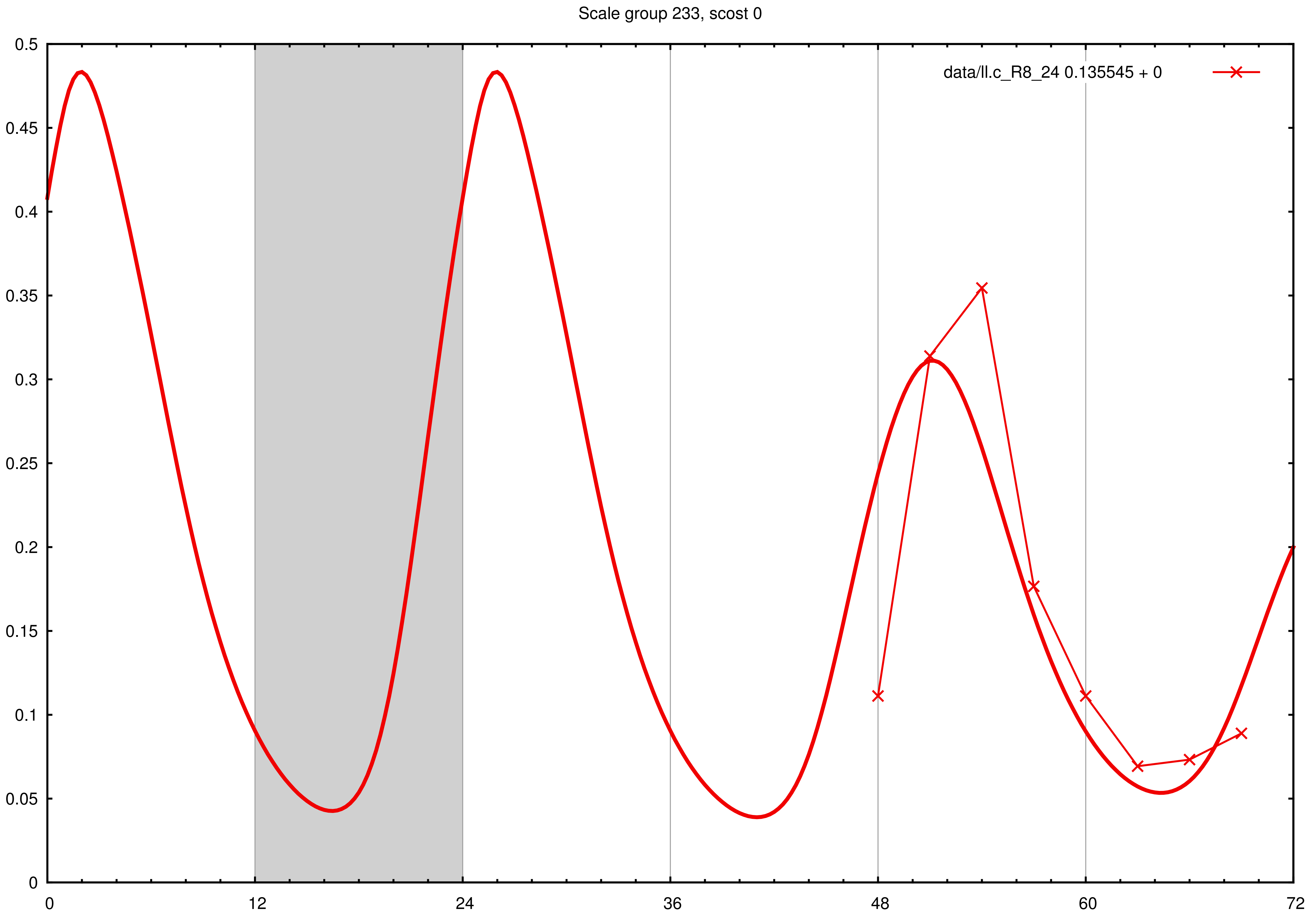

Scale group 233, scost 0
data/ll.c_R8_24 0.135545 + 0
0.5
0.45
0.4
0.35
0.3
0.25
0.2
0.15
0.1
0.05
0
0
12
24
36
48
60
72

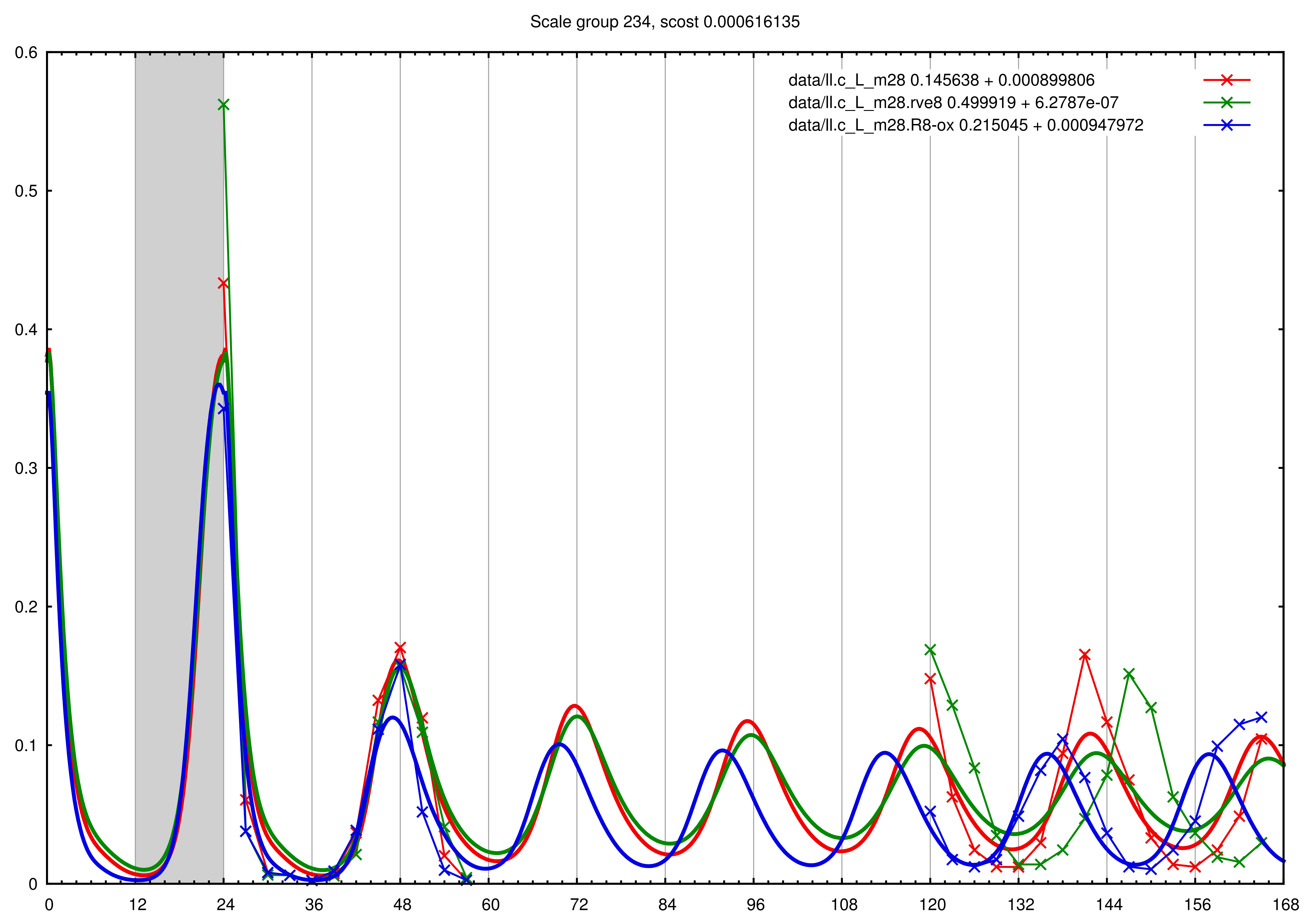

Scale group 234, scost 0.000616135
data/ll.c_L_m28 0.145638 + 0.000899806
data/ll.c_L_m28.rve8 0.499919 + 6.2787e-07
data/ll.c_L_m28.R8-ox 0.215045 + 0.000947972
0.6
0.5
0.4
0.3
0.2
0.1
0
0
12
24
36
48
60
72
84
96
108
120
132
144
156
168

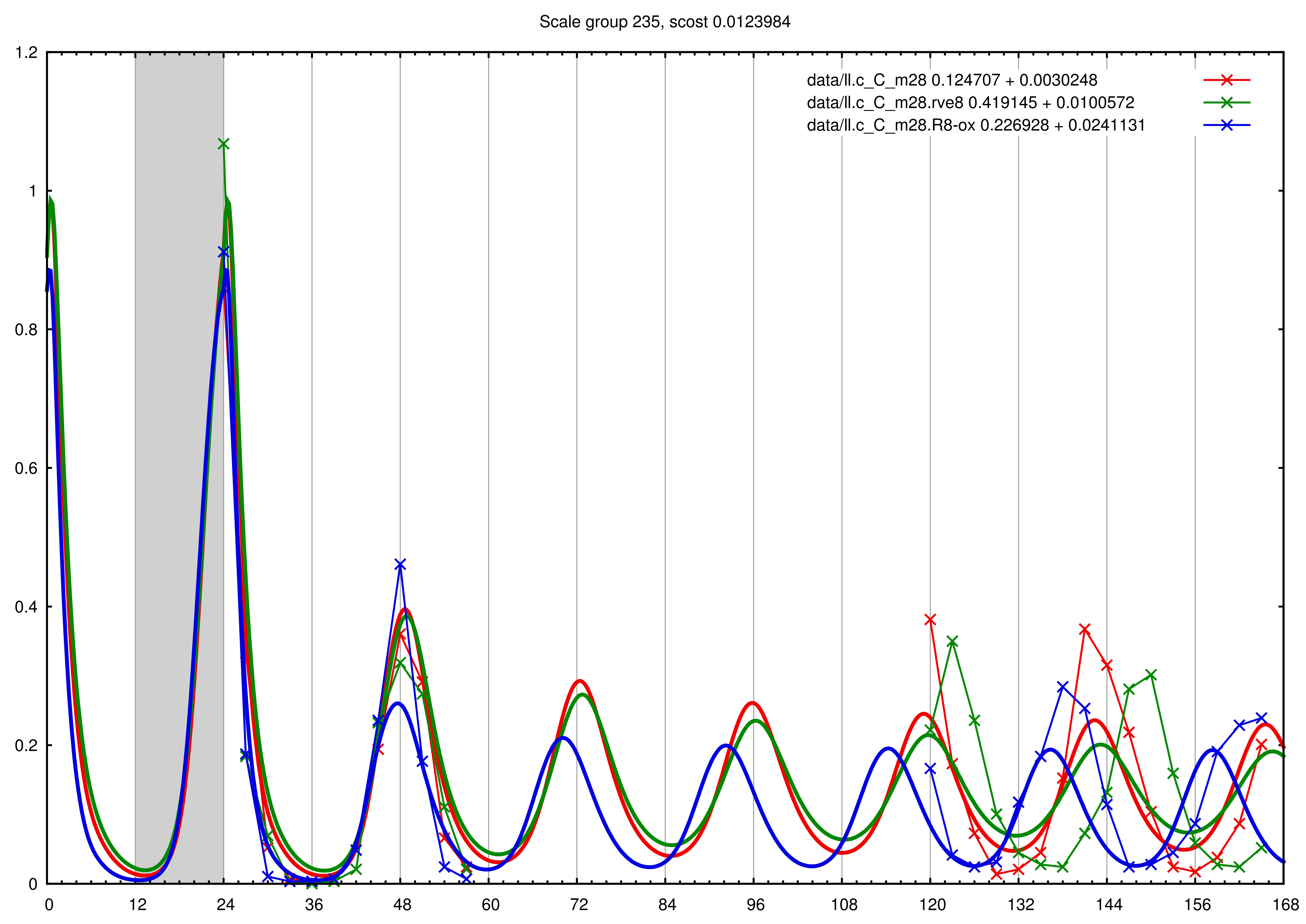

Scale group 235, scost 0.0123984
data/ll.c_C_m28 0.124707 + 0.0030248
data/ll.c_C_m28.rve8 0.419145 + 0.0100572
data/ll.c_C_m28.R8-ox 0.226928 + 0.0241131
1.2
1
0.8
0.6
0.4
0.2
0
0
12
24
36
48
60
72
84
96
108
120
132
144
156
168

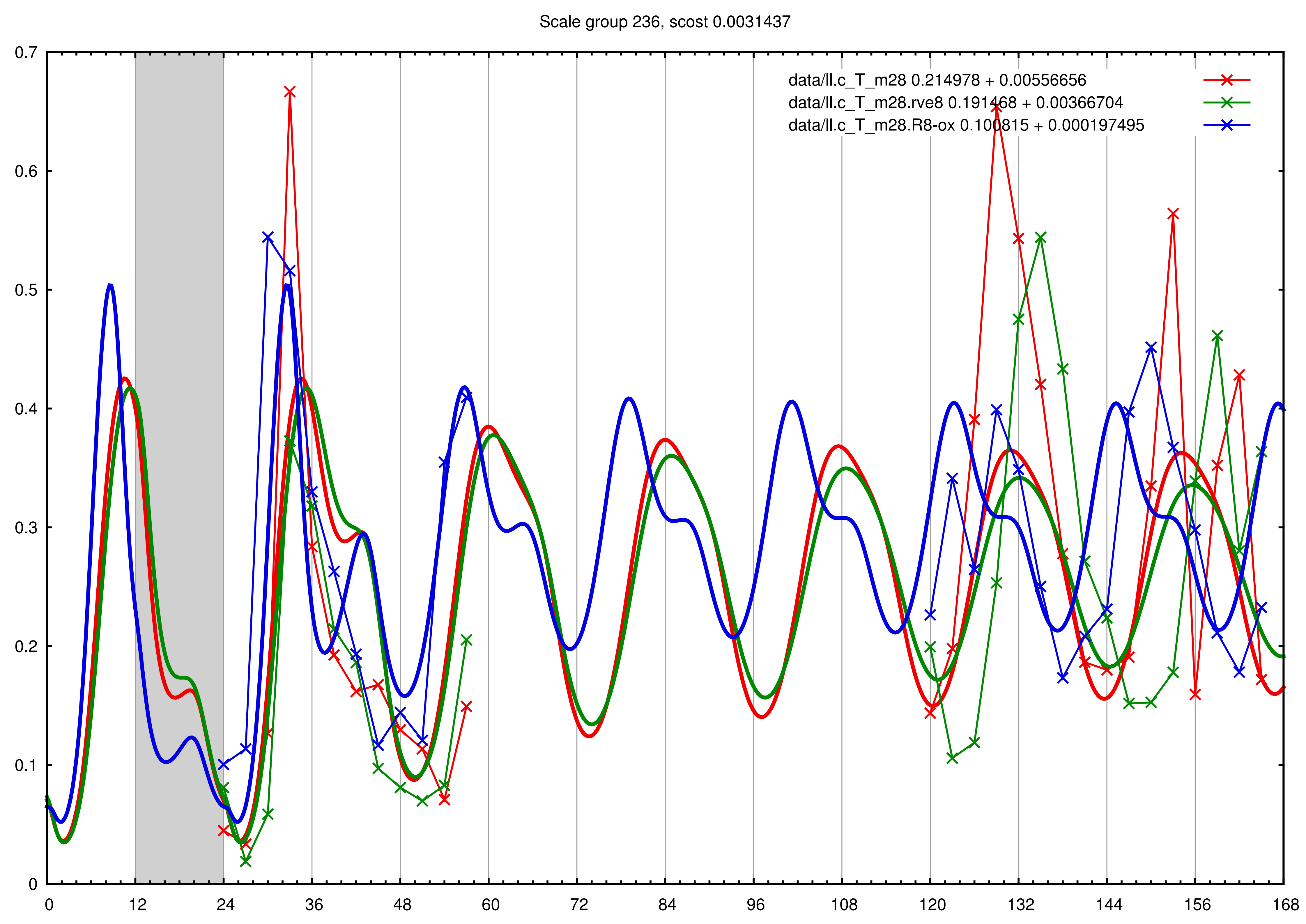

Scale group 236, scost 0.0031437
data/ll.c_T_m28 0.214978 + 0.00556656
data/ll.c_T_m28.rve8 0.191468 + 0.00366704
data/ll.c_T_m28.R8-ox 0.100815 + 0.000197495
0.7
0.6
0.5
0.4
0.3
0.2
0.1
0
0
12
24
36
48
60
72
84
96
108
120
132
144
156
168

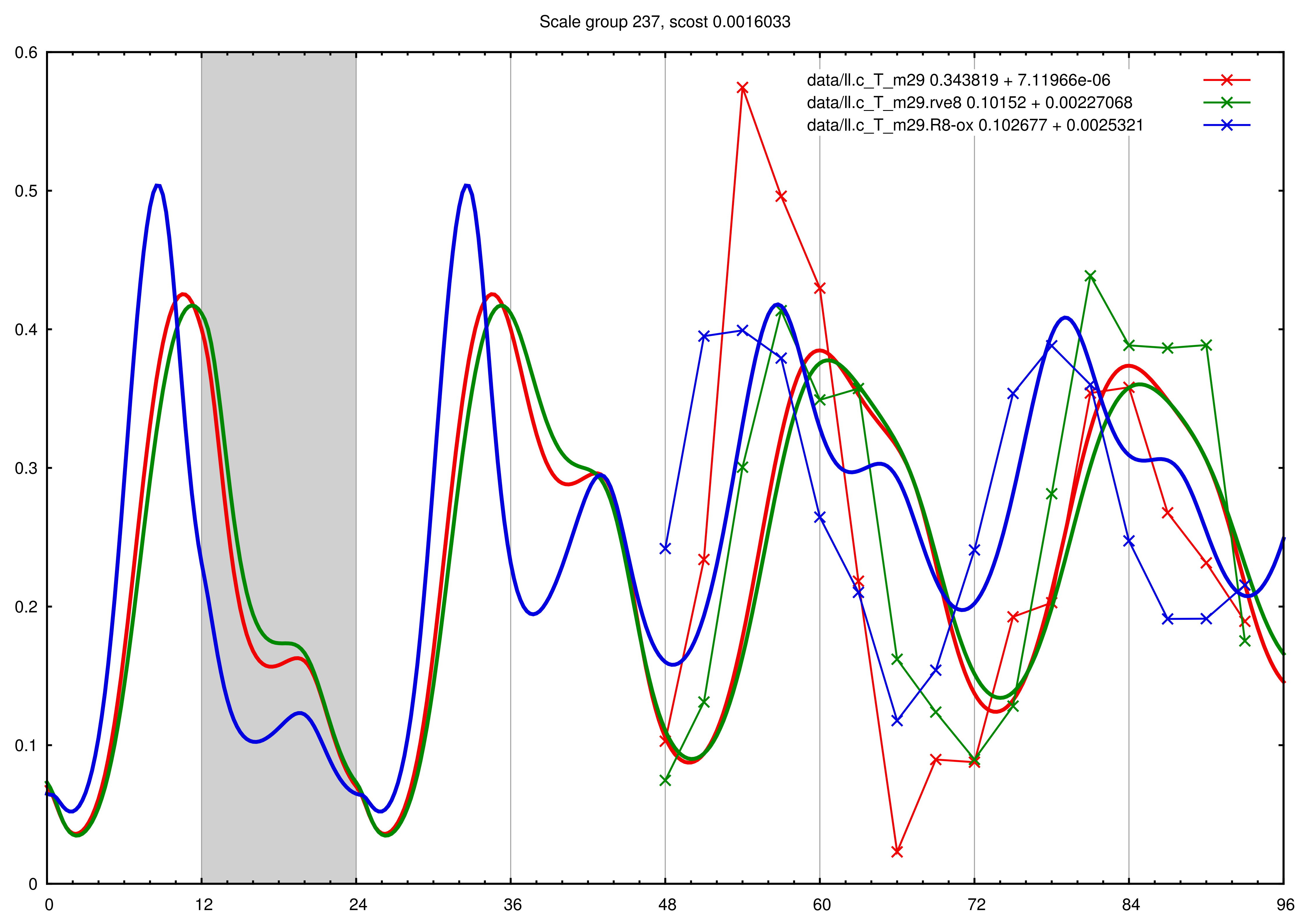
Scale group 237, scost 0.0016033
data/ll.c_T_m29 0.343819 + 7.11966e-06
data/ll.c_T_m29.rve8 0.10152 + 0.00227068
data/ll.c_T_m29.R8-ox 0.102677 + 0.0025321
0.6
0.5
0.4
0.3
0.2
0.1
0
0
12
24
36
48
60
72
84
96

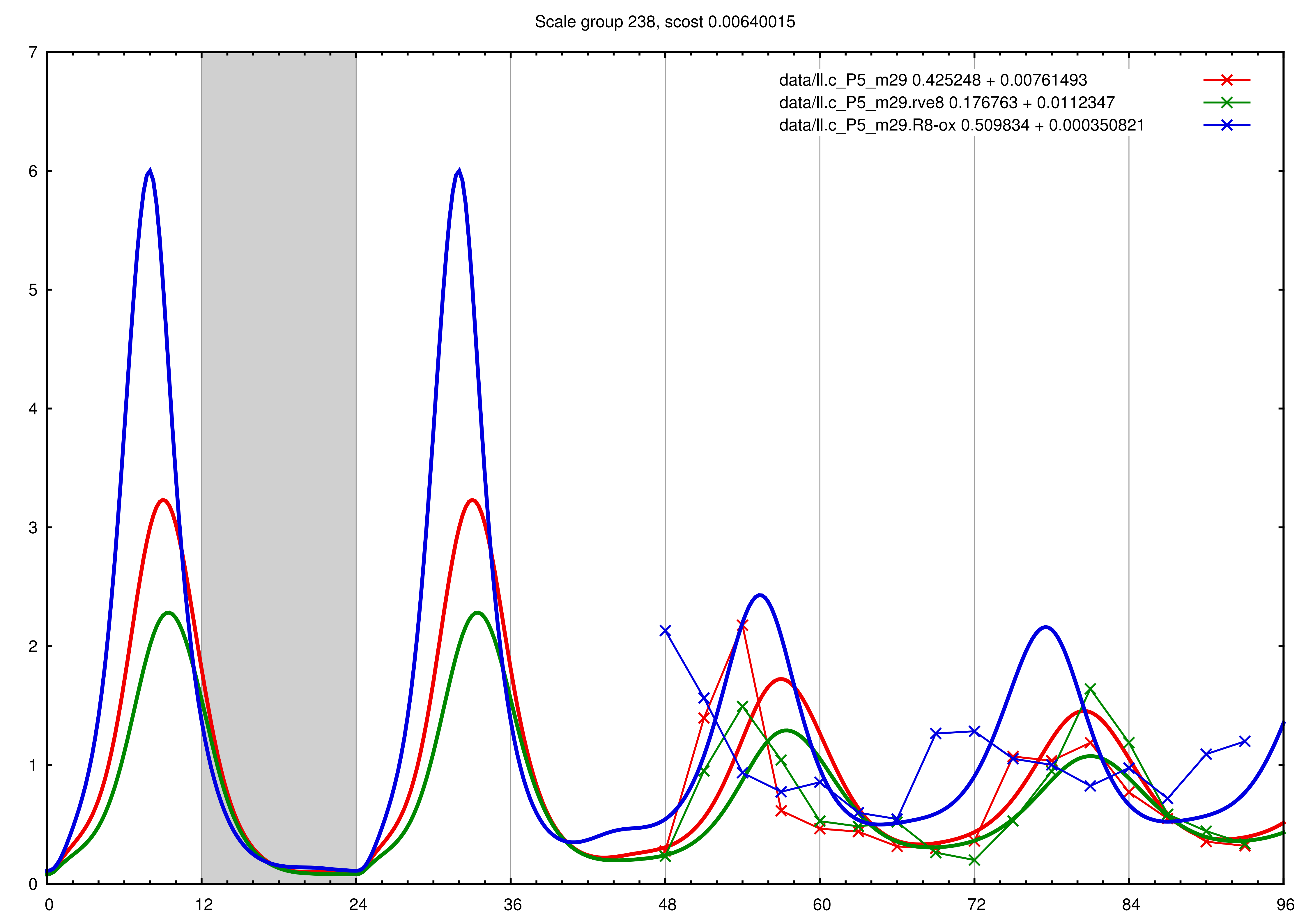

Scale group 238, scost 0.00640015
data/ll.c_P5_m29 0.425248 + 0.00761493
data/ll.c_P5_m29.rve8 0.176763 + 0.0112347
data/ll.c_P5_m29.R8-ox 0.509834 + 0.000350821
0
1
2
3
4
5
6
7
0
12
24
36
48
60
72
84
96

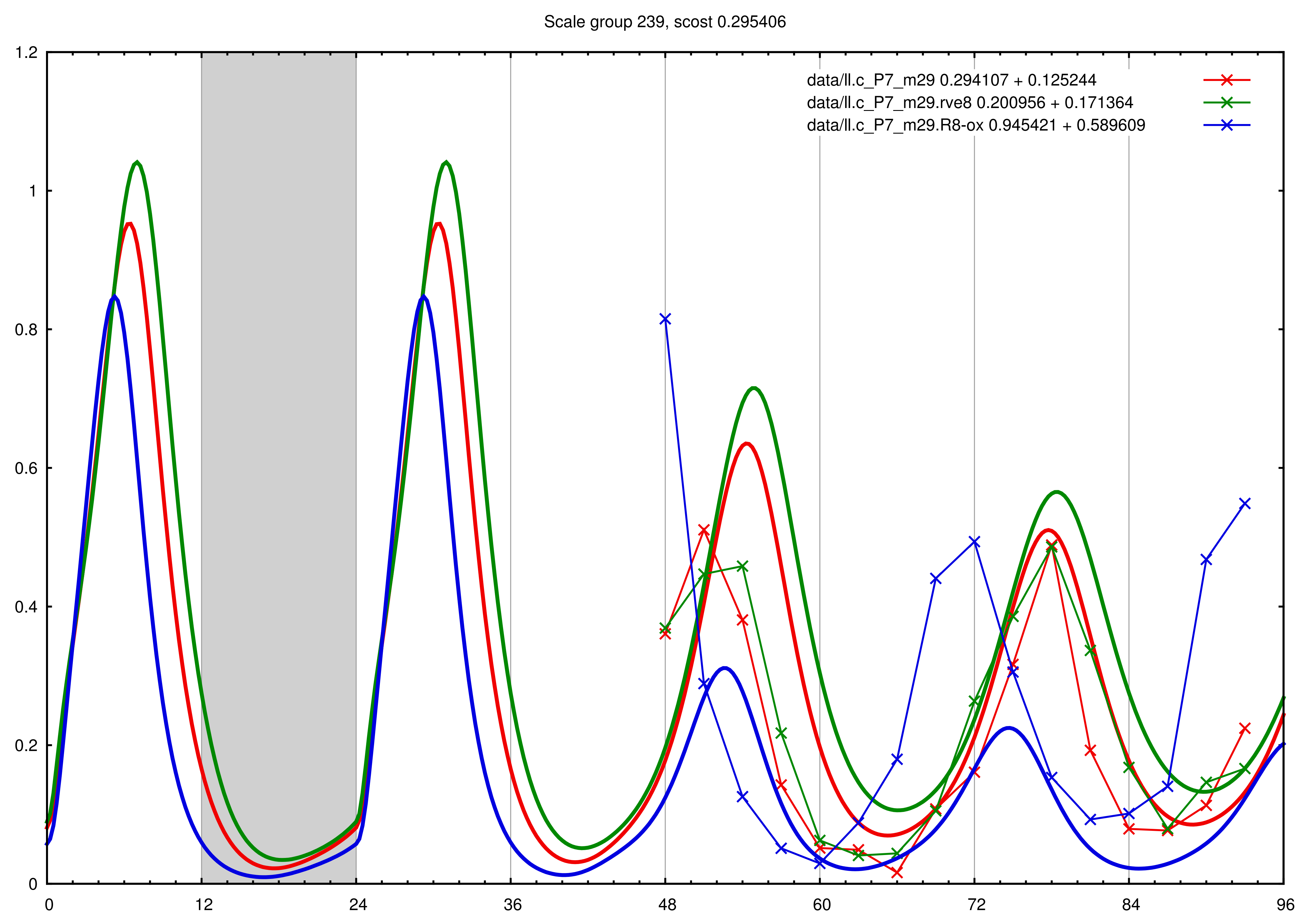
Scale group 239, scost 0.295406
data/ll.c_P7_m29 0.294107 + 0.125244
data/ll.c_P7_m29.rve8 0.200956 + 0.171364
data/ll.c_P7_m29.R8-ox 0.945421 + 0.589609
0
0.2
0.4
0.6
0.8
1
1.2
0
12
24
36
48
60
72
84
96

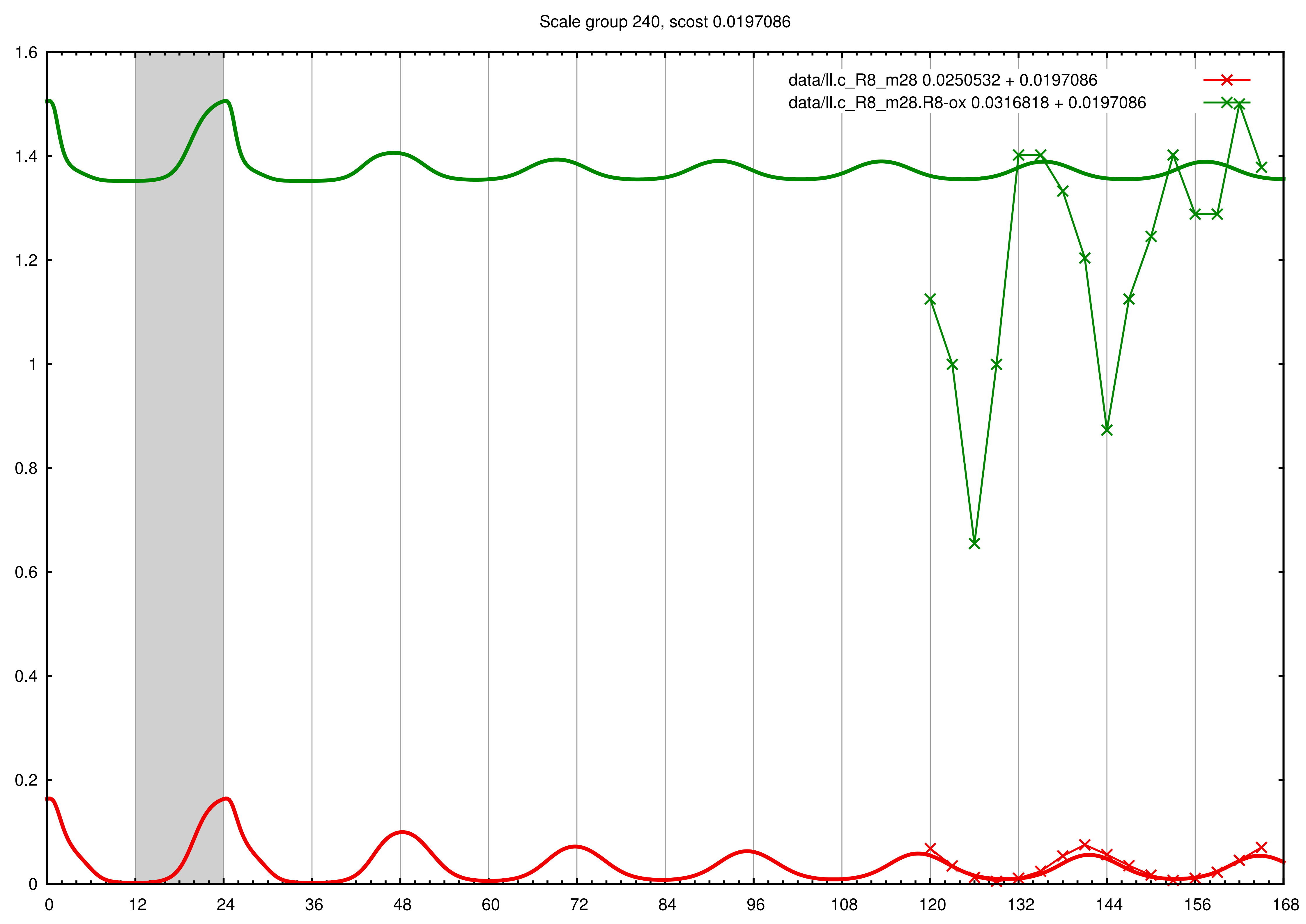
Scale group 240, scost 0.0197086
data/ll.c_R8_m28 0.0250532 + 0.0197086
data/ll.c_R8_m28.R8-ox 0.0316818 + 0.0197086
1.6
1.4
1.2
1
0.8
0.6
0.4
0.2
0
0
12
24
36
48
60
72
84
96
108
120
132
144
156
168

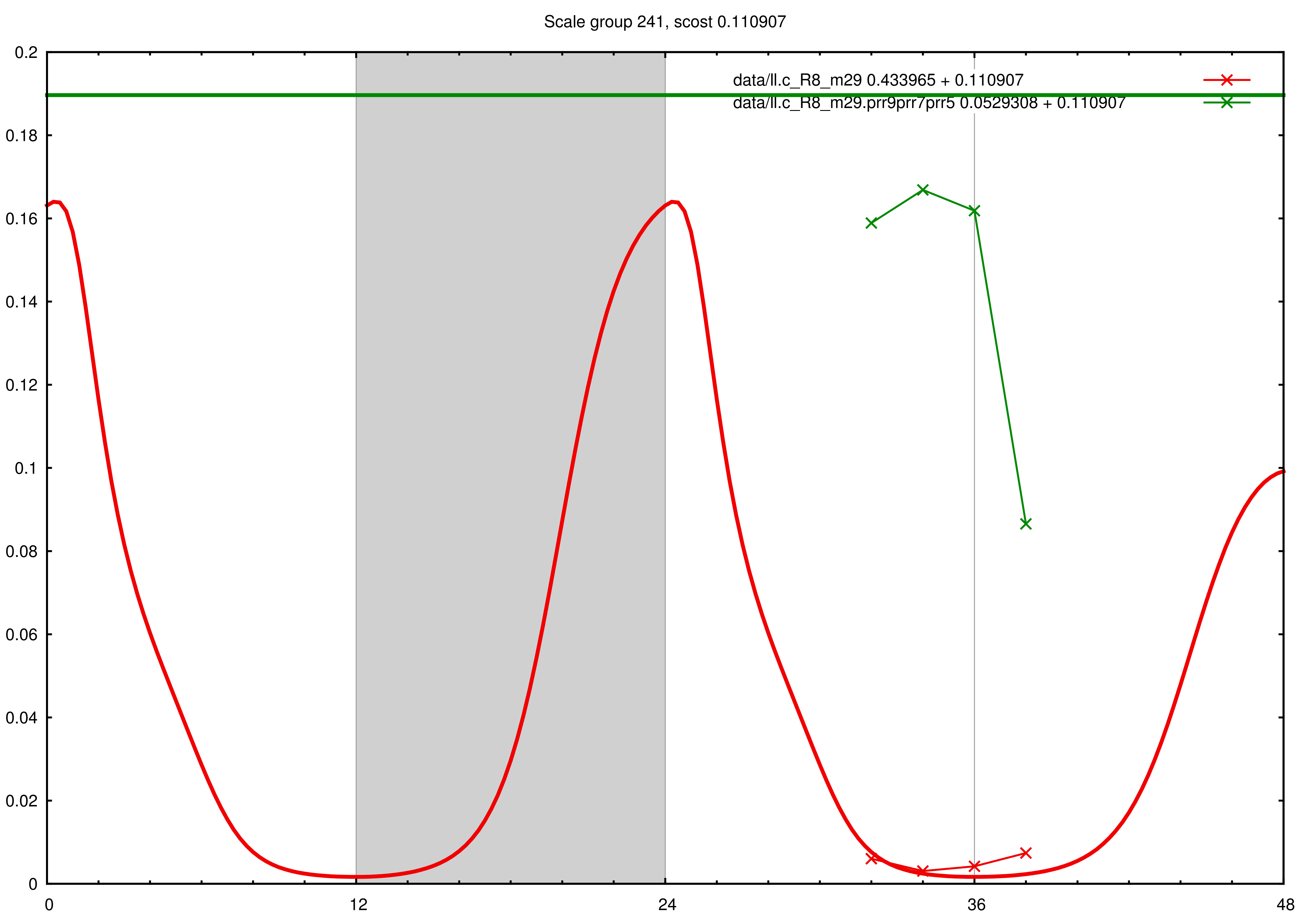

Scale group 241, scost 0.110907
data/ll.c_R8_m29 0.433965 + 0.110907
data/ll.c_R8_m29.prr9prr7prr5 0.0529308 + 0.110907
0.2
0.18
0.16
0.14
0.12
0.1
0.08
0.06
0.04
0.02
0
0
12
24
36
48

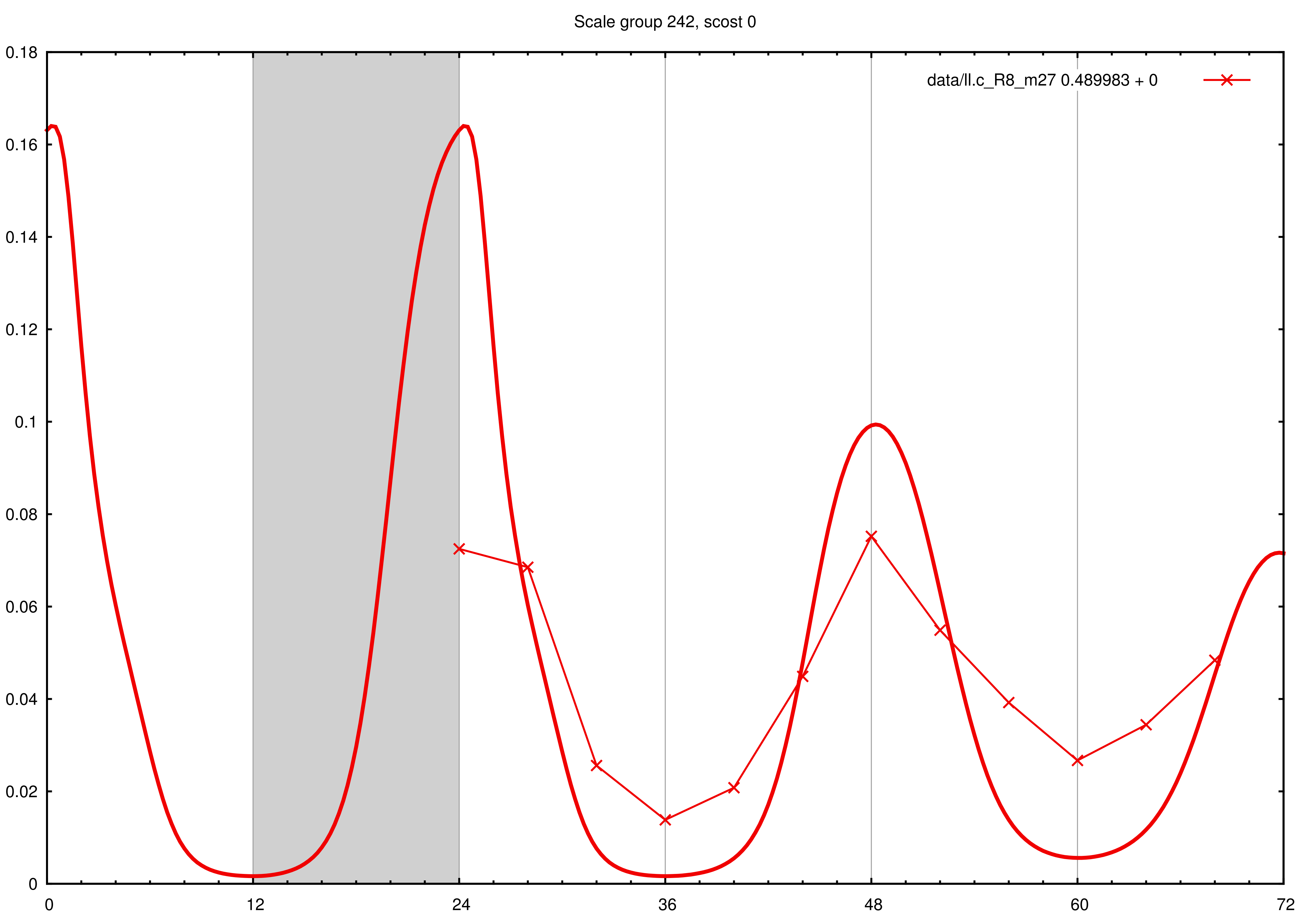

Scale group 242, scost 0
data/ll.c_R8_m27 0.489983 + 0
0.18
0.16
0.14
0.12
0.1
0.08
0.06
0.04
0.02
0
0
12
24
36
48
60
72

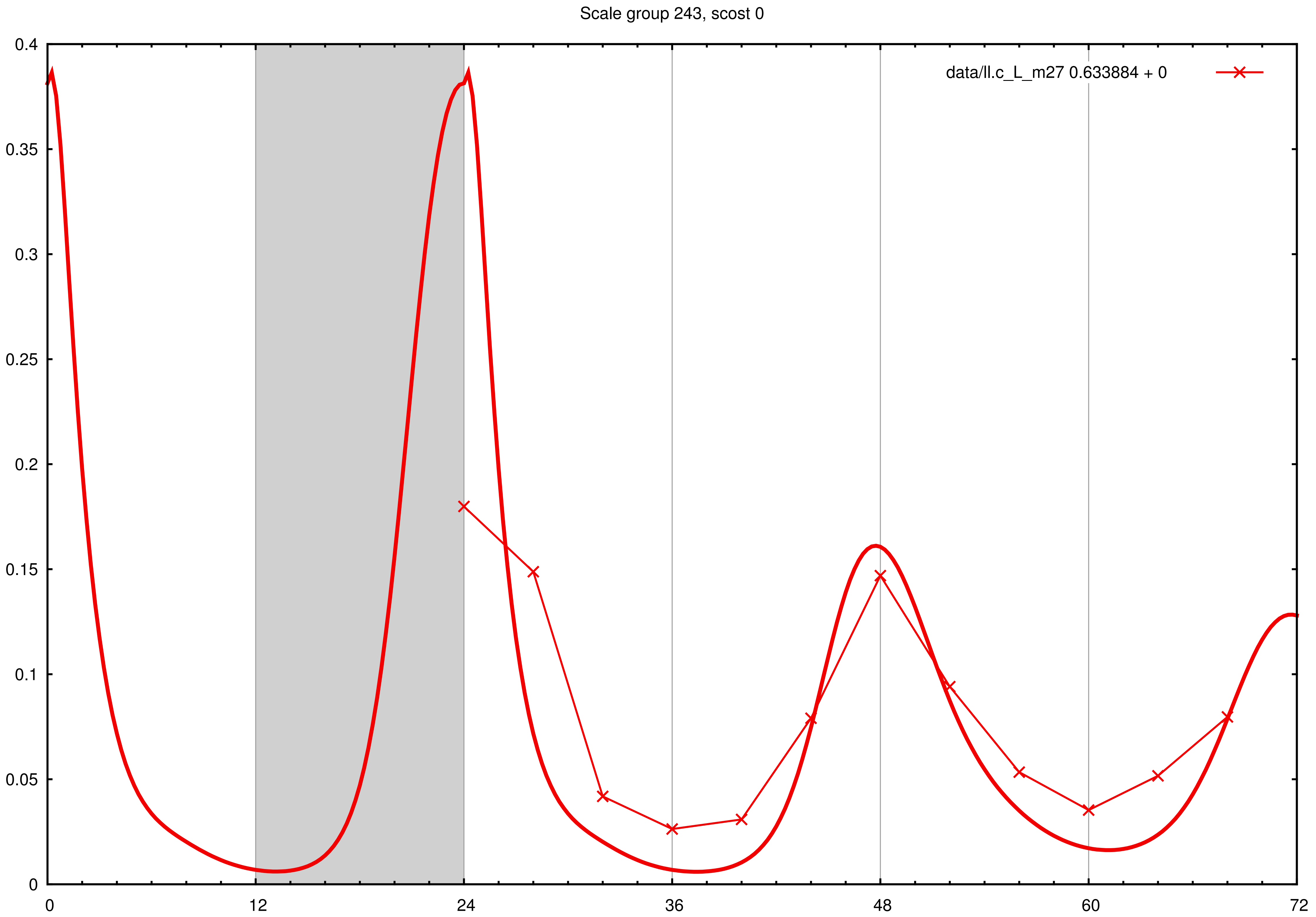

Scale group 243, scost 0
data/ll.c_L_m27 0.633884 + 0
0.4
0.35
0.3
0.25
0.2
0.15
0.1
0.05
0
0
12
24
36
48
60
72

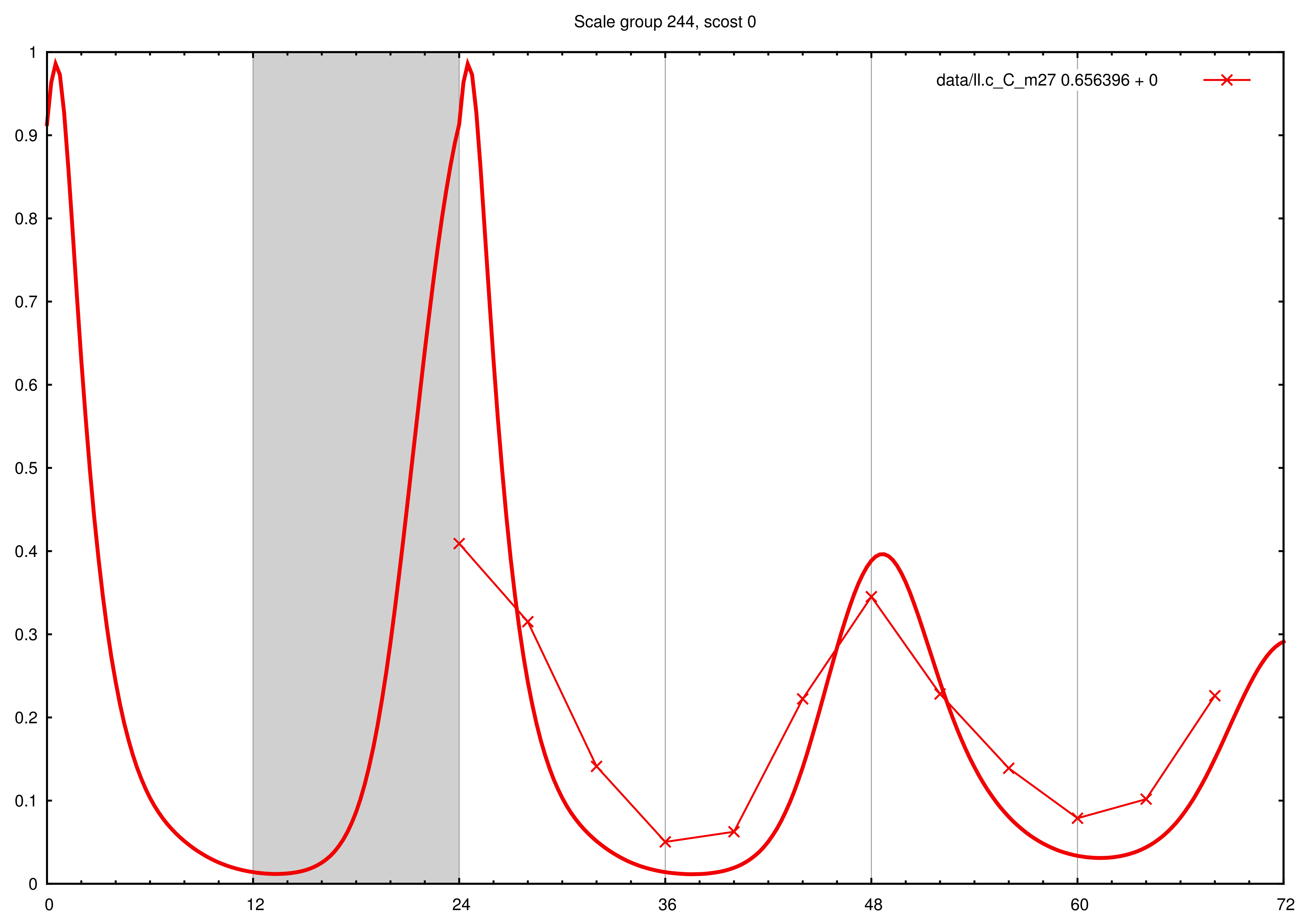

Scale group 244, scost 0
data/ll.c_C_m27 0.656396 + 0
1
0.9
0.8
0.7
0.6
0.5
0.4
0.3
0.2
0.1
0
0
12
24
36
48
60
72

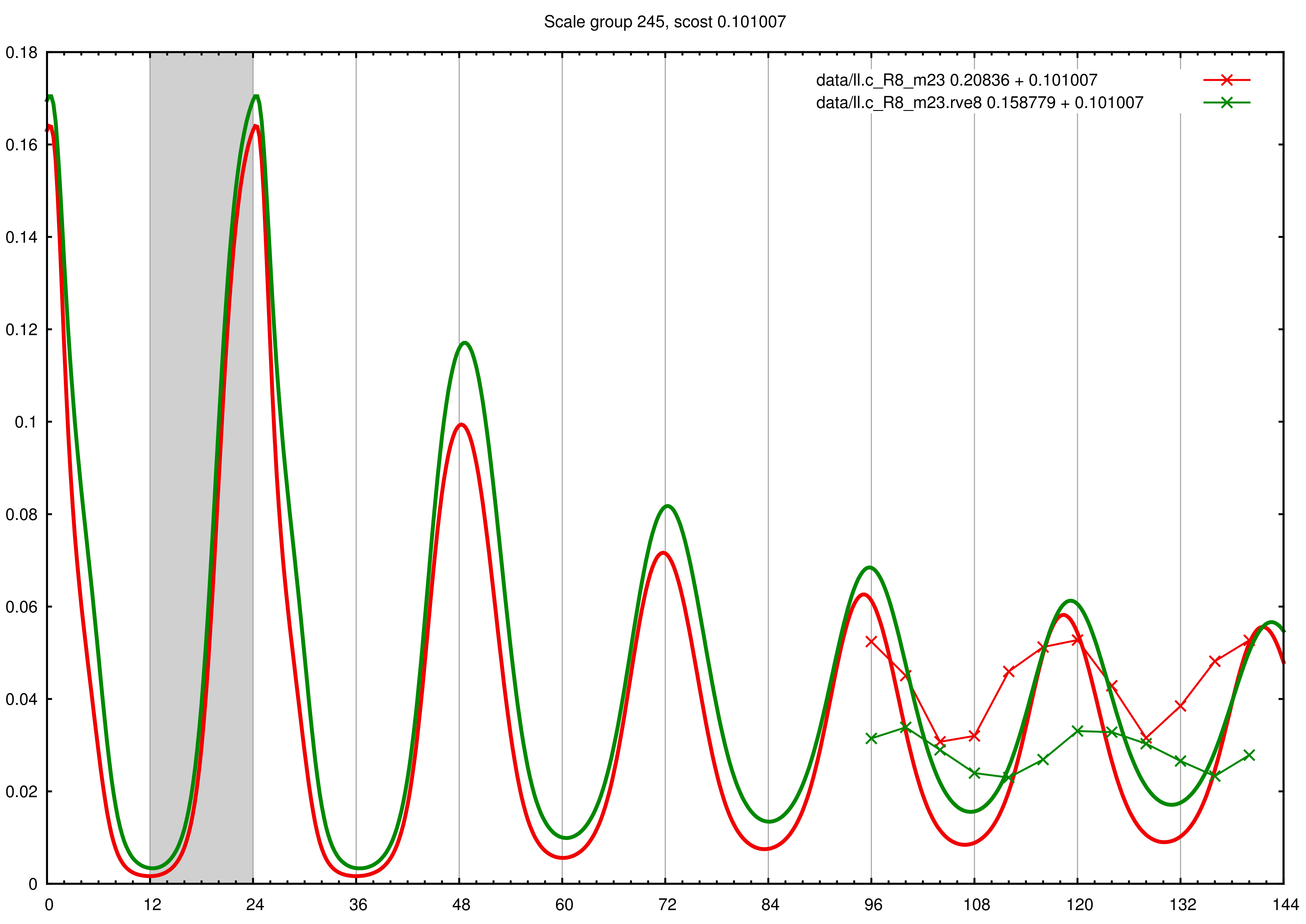

Scale group 245, scost 0.101007
data/ll.c_R8_m23 0.20836 + 0.101007
data/ll.c_R8_m23.rve8 0.158779 + 0.101007
0.18
0.16
0.14
0.12
0.1
0.08
0.06
0.04
0.02
0
0
12
24
36
48
60
72
84
96
108
120
132
144

Scale group 246, scost 1.21987e-05

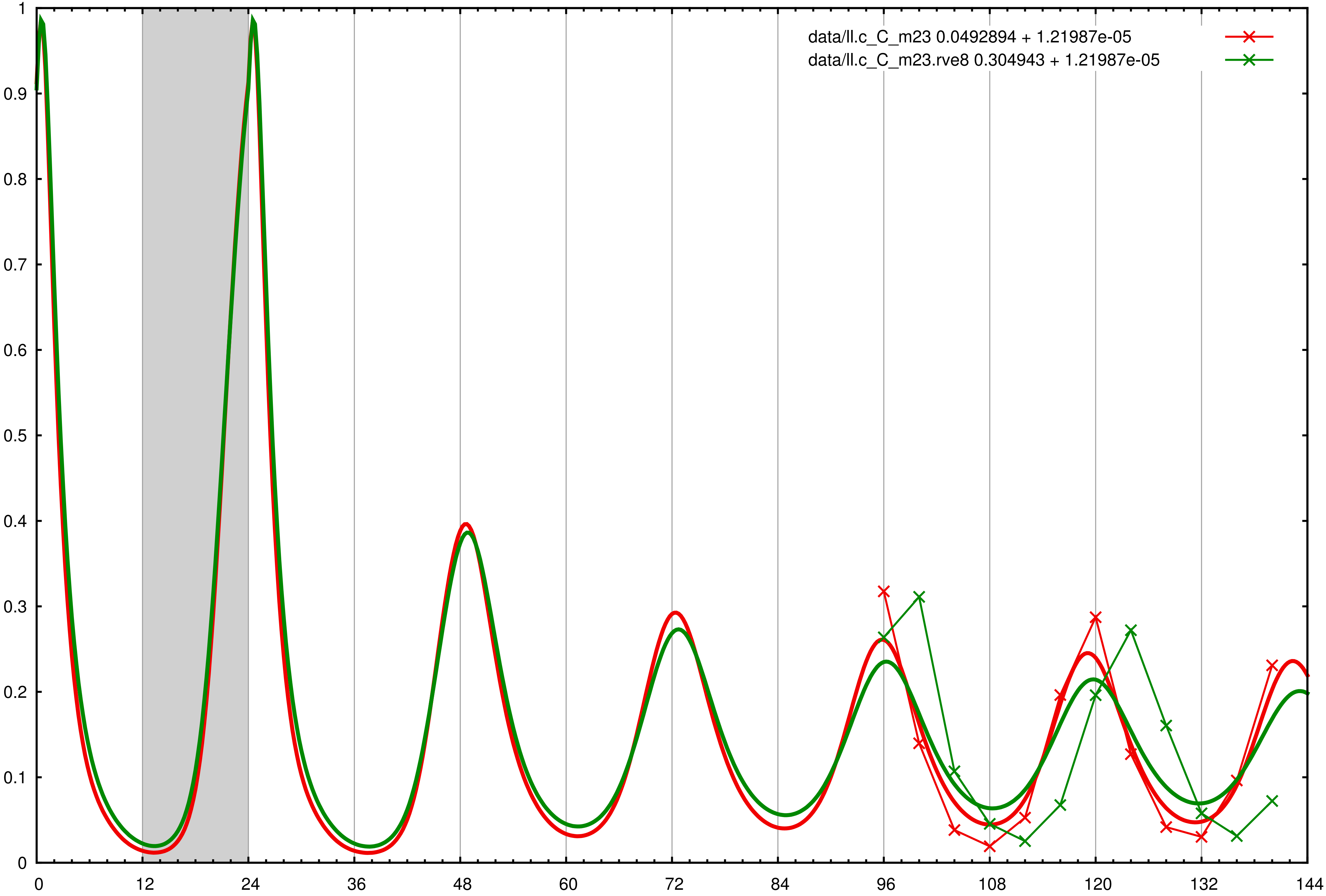

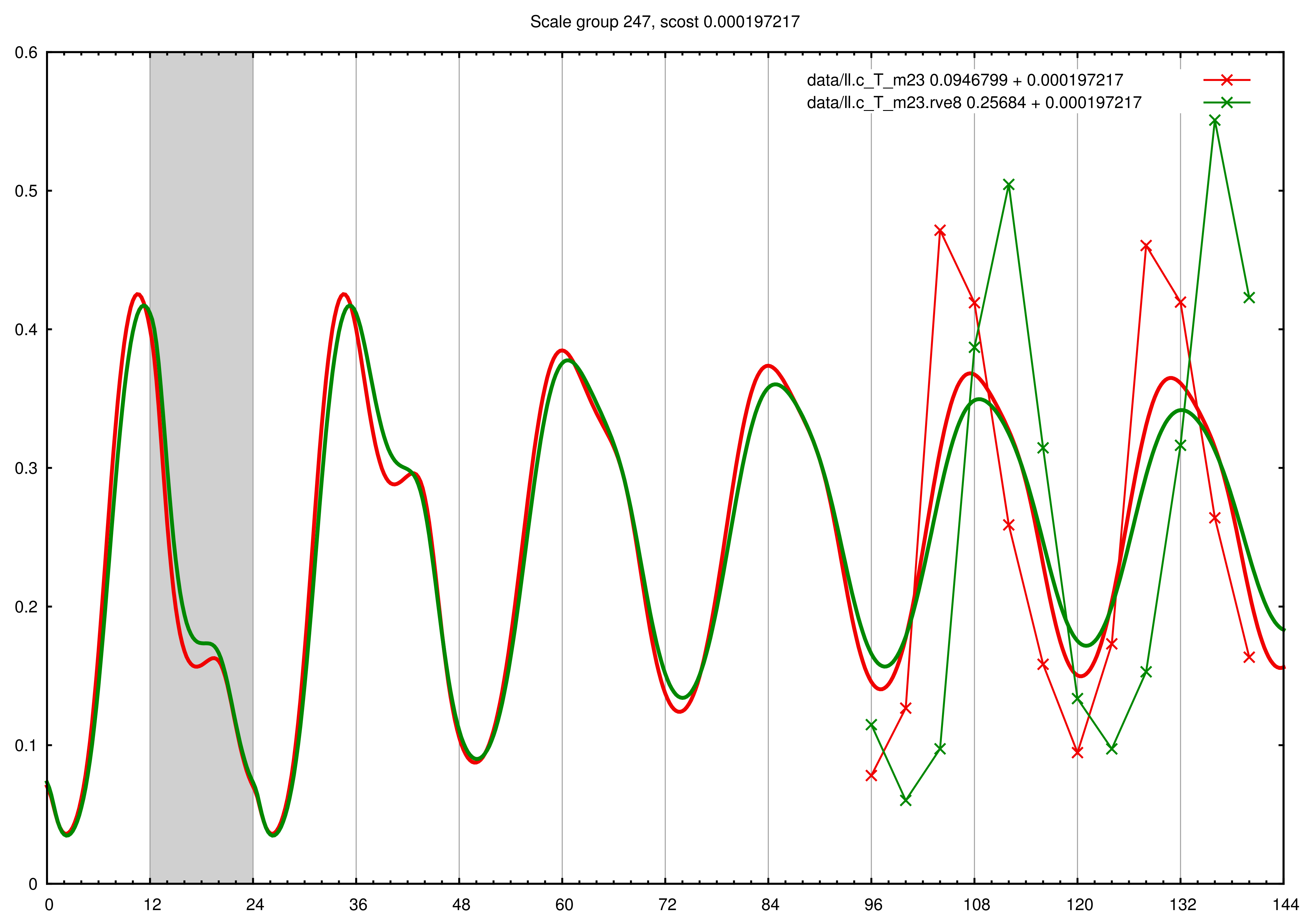

Scale group 247, scost 0.000197217
data/ll.c_T_m23 0.0946799 + 0.000197217
data/ll.c_T_m23.rve8 0.25684 + 0.000197217
0.6
0.5
0.4
0.3
0.2
0.1
0
0
12
24
36
48
60
72
84
96
108
120
132
144

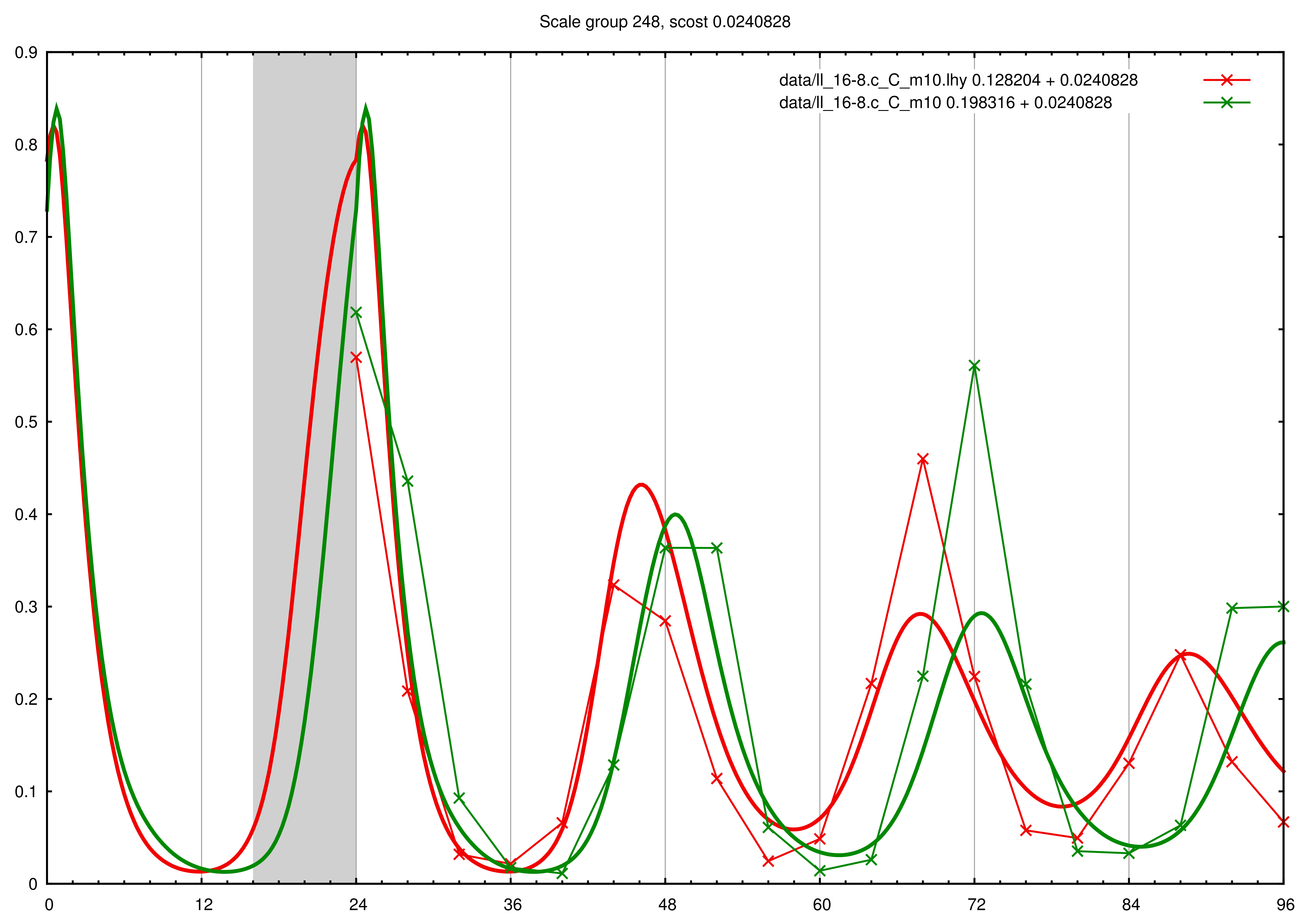

Scale group 248, scost 0.0240828
data/ll_16-8.c_C_m10.lhy 0.128204 + 0.0240828
data/ll_16-8.c_C_m10 0.198316 + 0.0240828
0.9
0.8
0.7
0.6
0.5
0.4
0.3
0.2
0.1
0
0
12
24
36
48
60
72
84
96

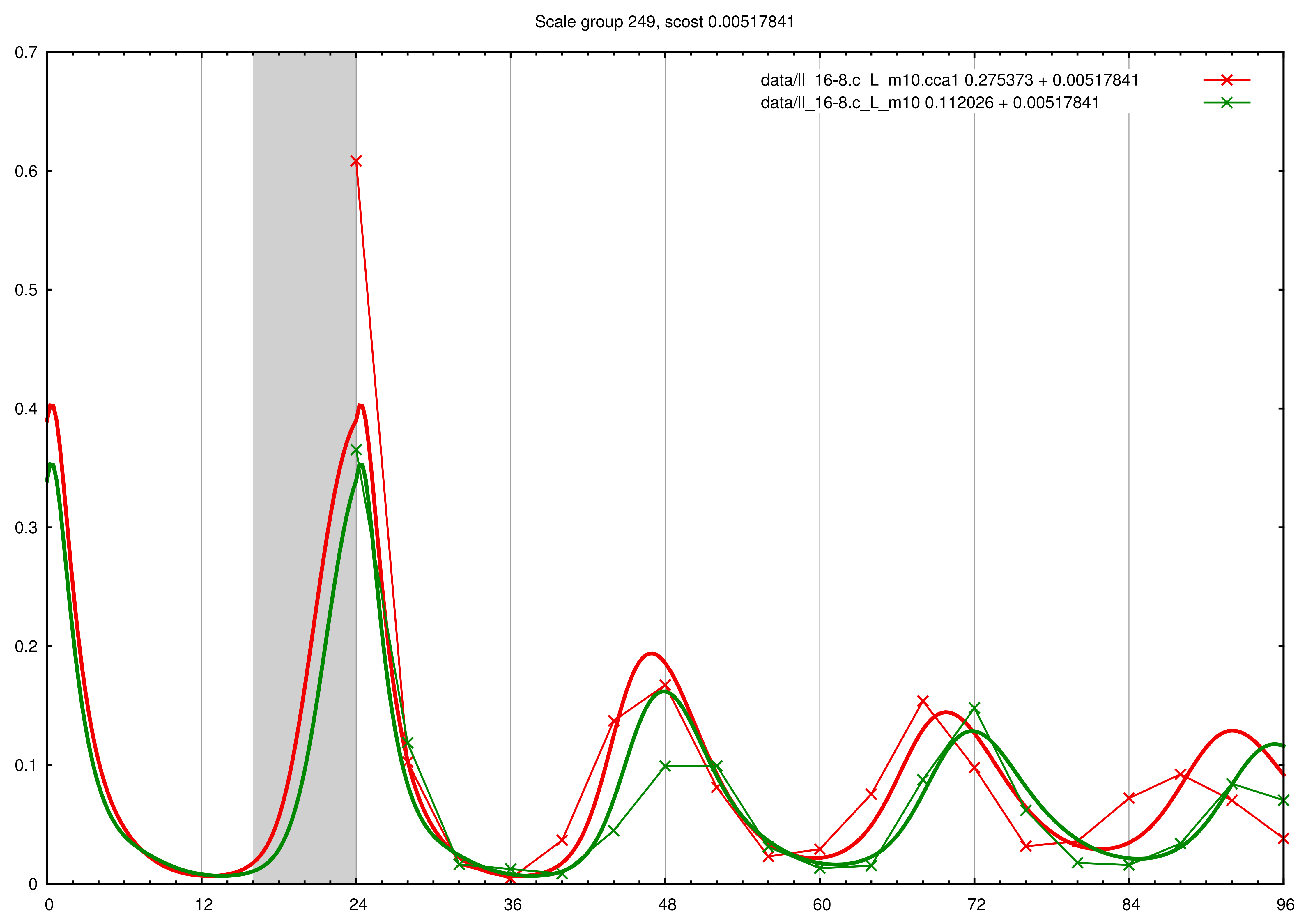

Scale group 249, scost 0.00517841
data/ll_16-8.c_L_m10.cca1 0.275373 + 0.00517841
data/ll_16-8.c_L_m10 0.112026 + 0.00517841
0.7
0.6
0.5
0.4
0.3
0.2
0.1
0
0
12
24
36
48
60
72
84
96

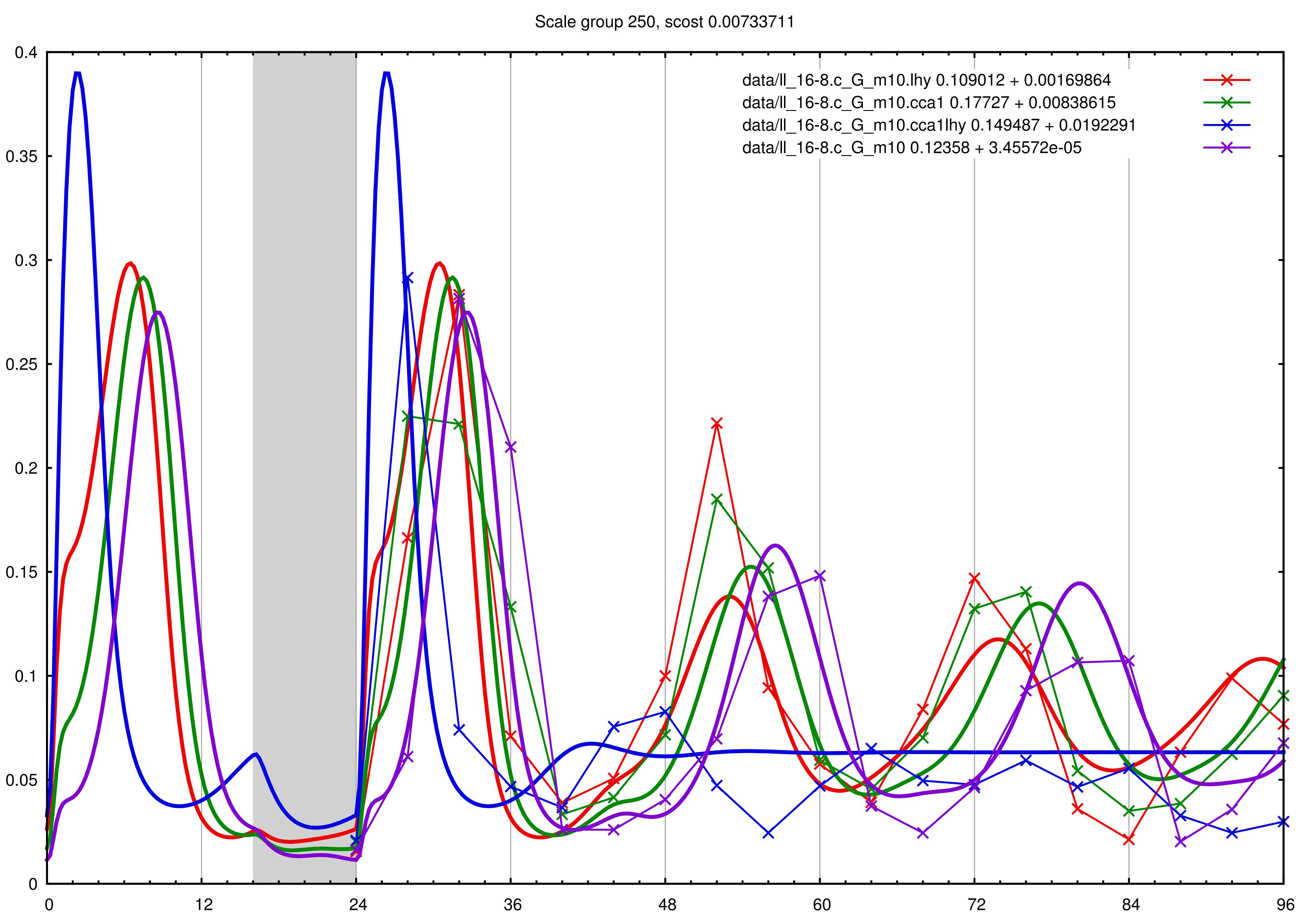
Scale group 250, scost 0.00733711
data/ll_16-8.c_G_m10.lhy 0.109012 + 0.00169864
data/ll_16-8.c_G_m10.cca1 0.17727 + 0.00838615
data/ll_16-8.c_G_m10.cca1lhy 0.149487 + 0.0192291
data/ll_16-8.c_G_m10 0.12358 + 3.45572e-05
0.4
0.35
0.3
0.25
0.2
0.15
0.1
0.05
0
0
12
24
36
48
60
72
84
96

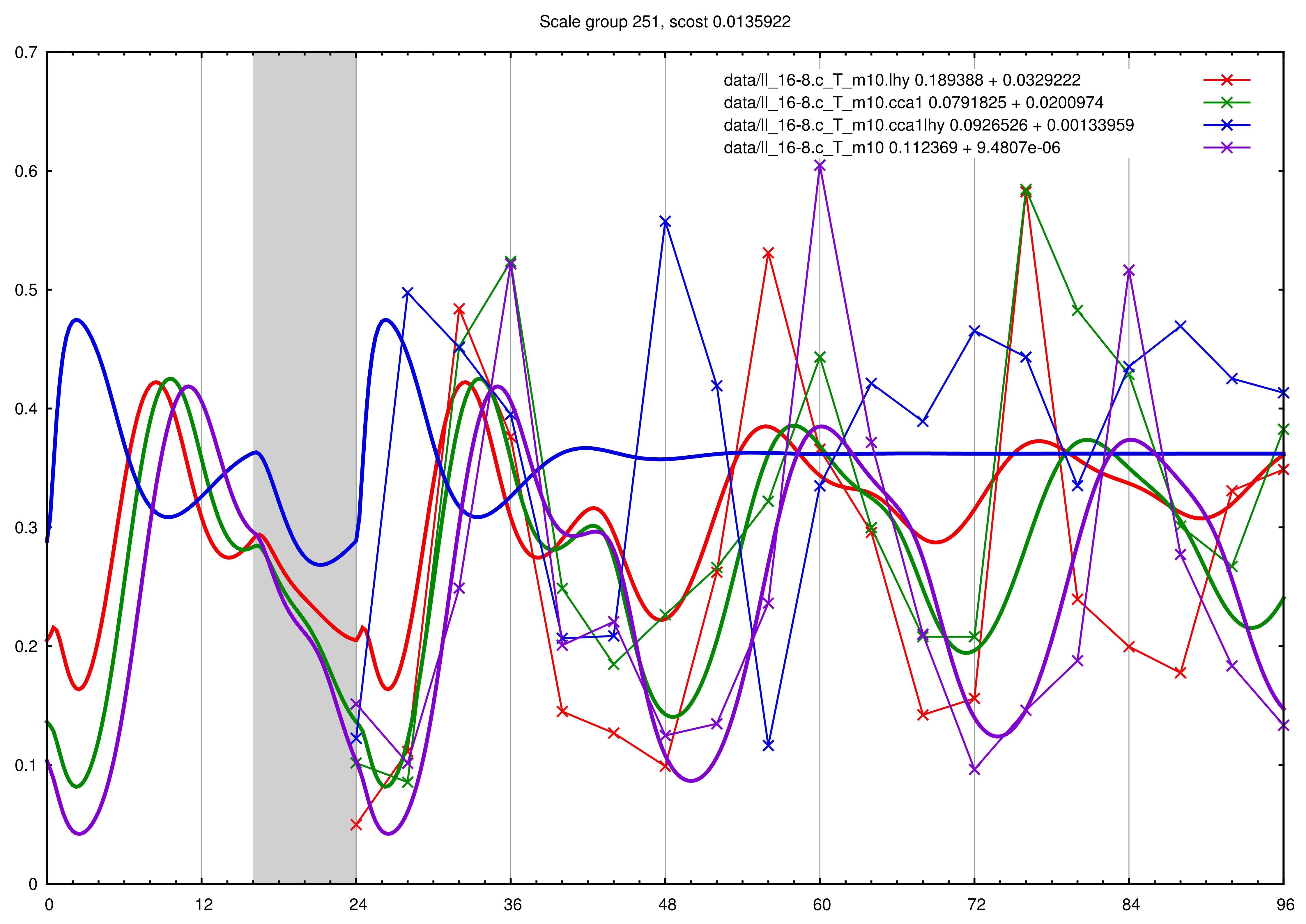

Scale group 251, scost 0.0135922
data/ll_16-8.c_T_m10.lhy 0.189388 + 0.0329222
data/ll_16-8.c_T_m10.cca1 0.0791825 + 0.0200974
data/ll_16-8.c_T_m10.cca1lhy 0.0926526 + 0.00133959
data/ll_16-8.c_T_m10 0.112369 + 9.4807e-06
0.7
0.6
0.5
0.4
0.3
0.2
0.1
0
0
12
24
36
48
60
72
84
96

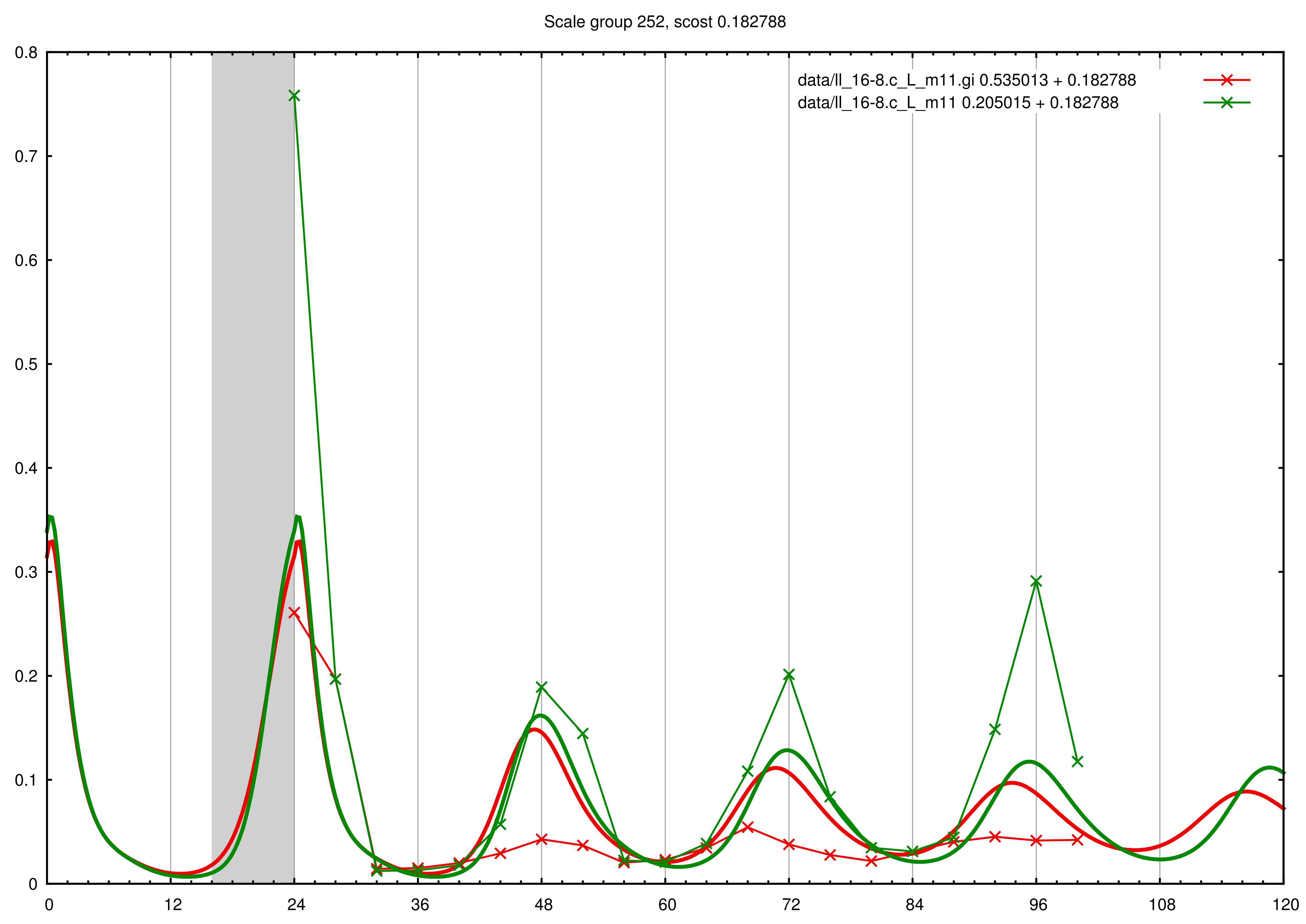
Scale group 252, scost 0.182788
data/ll_16-8.c_L_m11.gi 0.535013 + 0.182788
data/ll_16-8.c_L_m11 0.205015 + 0.182788
0.8
0.7
0.6
0.5
0.4
0.3
0.2
0.1
0
0
12
24
36
48
60
72
84
96
108
120

Scale group 253, scost 0.0668056

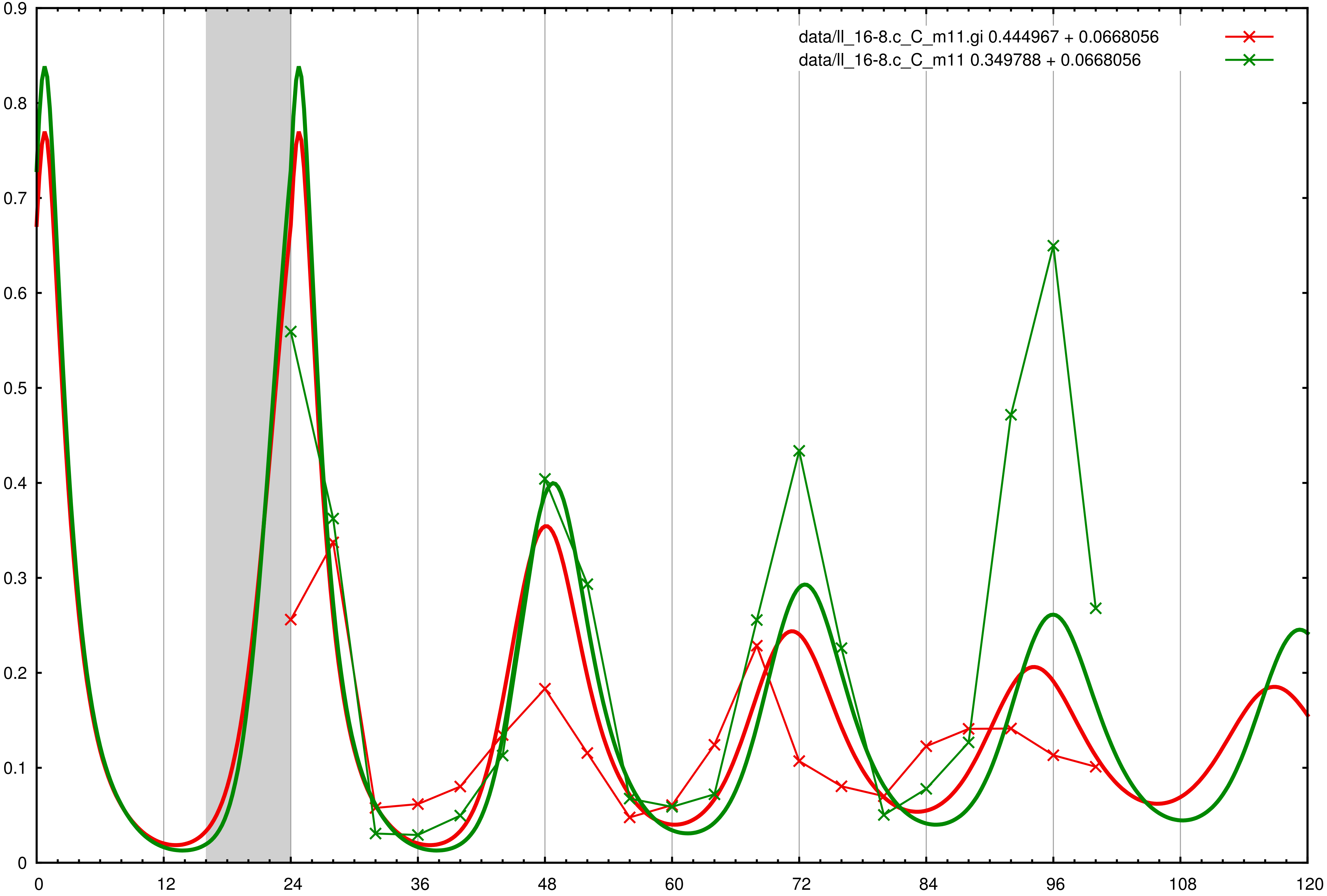

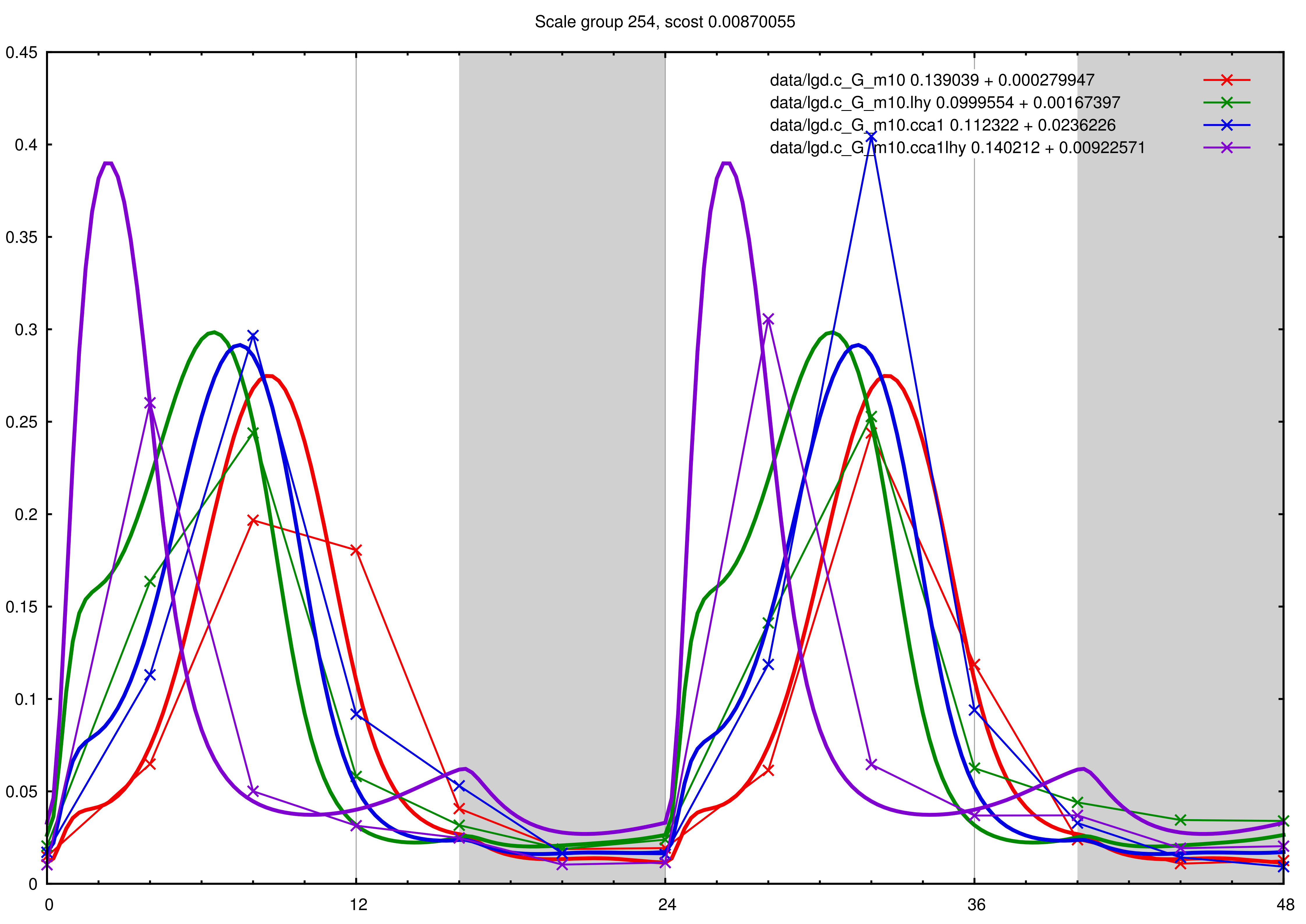

Scale group 254, scost 0.00870055
data/lgd.c_G_m10 0.139039 + 0.000279947
data/lgd.c_G_m10.lhy 0.0999554 + 0.00167397
data/lgd.c_G_m10.cca1 0.112322 + 0.0236226
data/lgd.c_G_m10.cca1lhy 0.140212 + 0.00922571
0.45
0.4
0.35
0.3
0.25
0.2
0.15
0.1
0.05
0
0
12
24
36
48

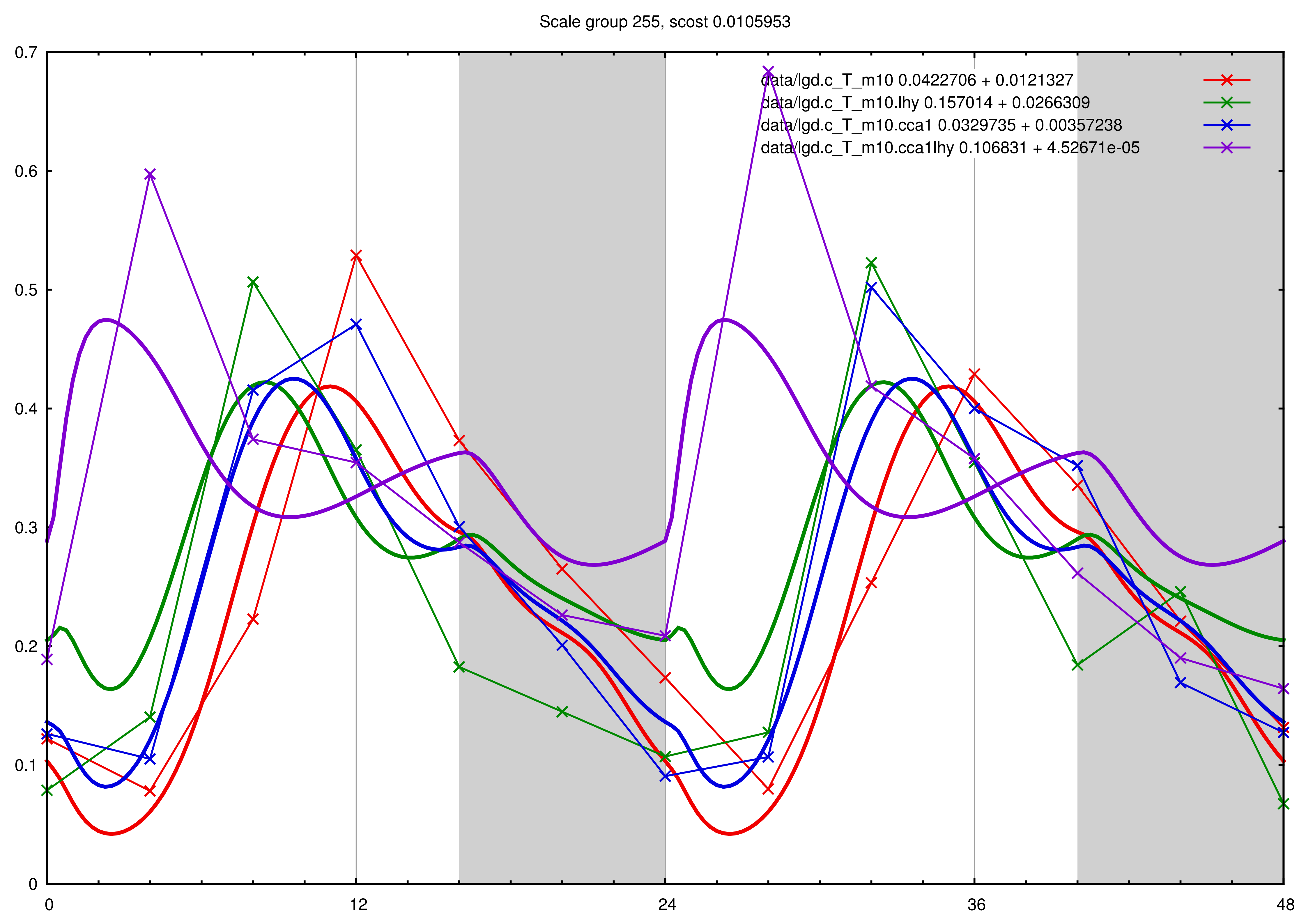
Scale group 255, scost 0.0105953
data/lgd.c_T_m10 0.0422706 + 0.0121327
data/lgd.c_T_m10.lhy 0.157014 + 0.0266309
data/lgd.c_T_m10.cca1 0.0329735 + 0.00357238
data/lgd.c_T_m10.cca1lhy 0.106831 + 4.52671e-05
0.7
0.6
0.5
0.4
0.3
0.2
0.1
0
0
12
24
36
48

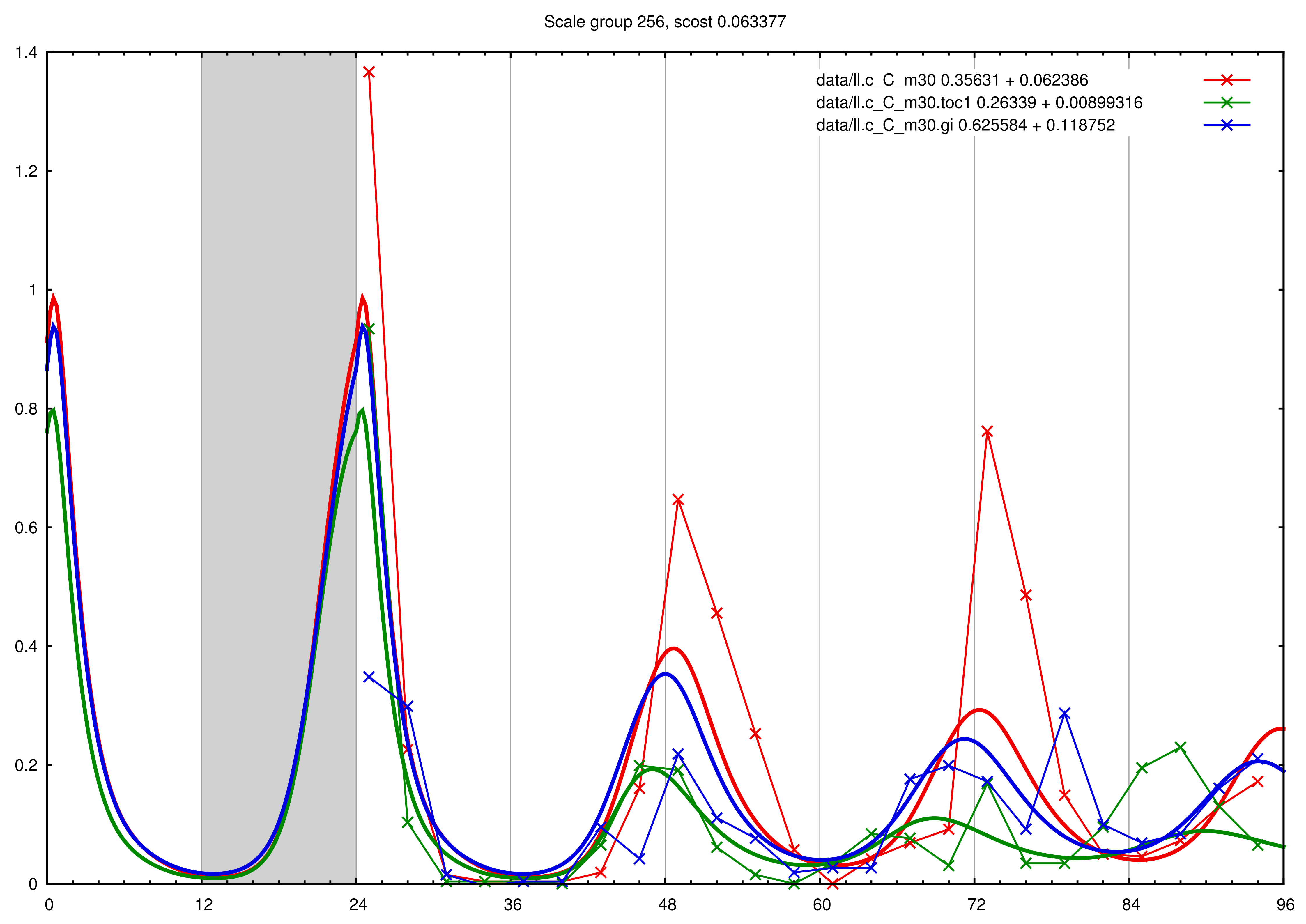

Scale group 256, scost 0.063377
data/ll.c_C_m30 0.35631 + 0.062386
data/ll.c_C_m30.toc1 0.26339 + 0.00899316
data/ll.c_C_m30.gi 0.625584 + 0.118752
1.4
1.2
1
0.8
0.6
0.4
0.2
0
0
12
24
36
48
60
72
84
96

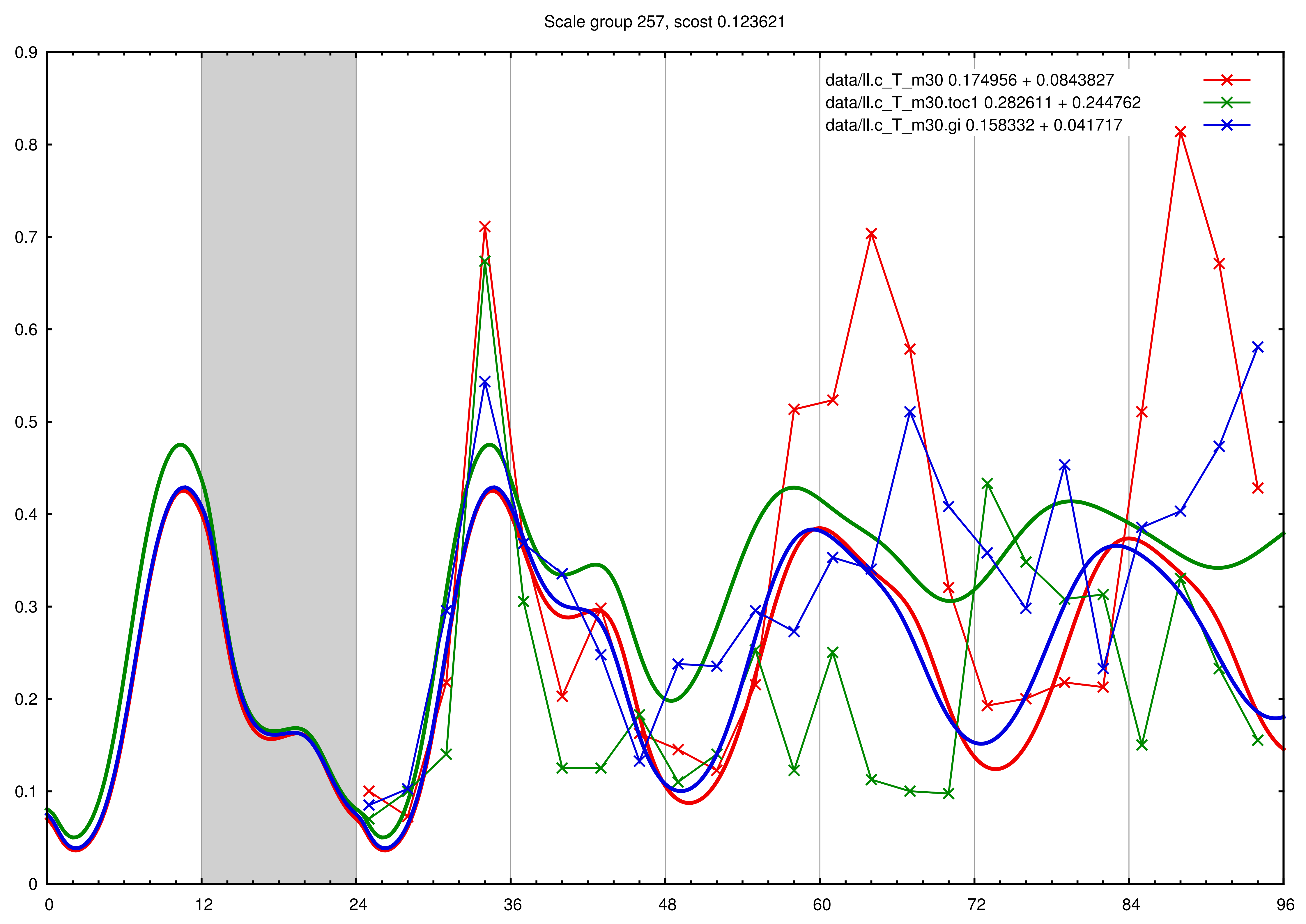
Scale group 257, scost 0.123621
data/ll.c_T_m30 0.174956 + 0.0843827
data/ll.c_T_m30.toc1 0.282611 + 0.244762
data/ll.c_T_m30.gi 0.158332 + 0.041717
0
0.1
0.2
0.3
0.4
0.5
0.6
0.7
0.8
0.9
0
12
24
36
48
60
72
84
96

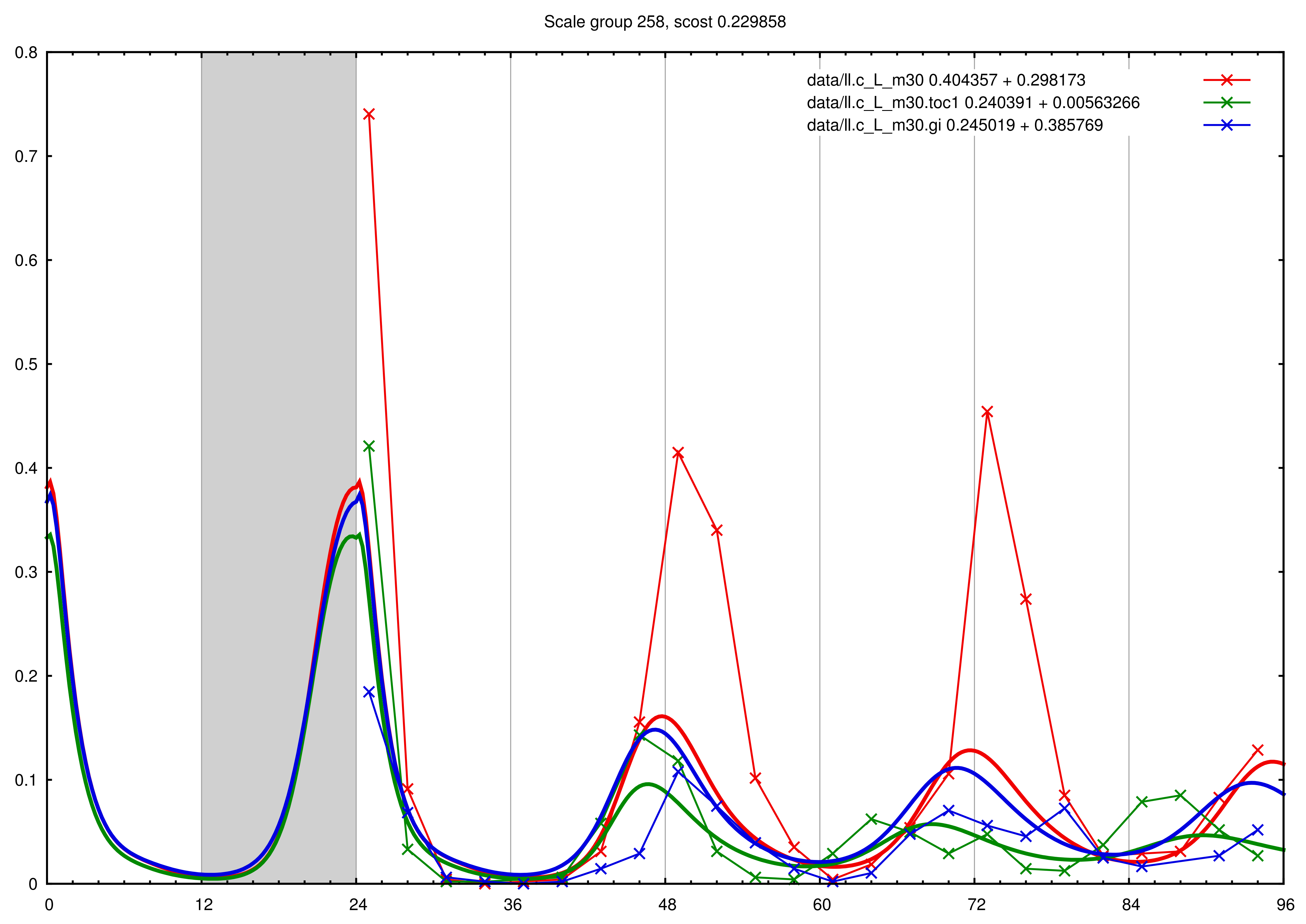

Scale group 258, scost 0.229858
data/ll.c_L_m30 0.404357 + 0.298173
data/ll.c_L_m30.toc1 0.240391 + 0.00563266
data/ll.c_L_m30.gi 0.245019 + 0.385769
0
0.1
0.2
0.3
0.4
0.5
0.6
0.7
0.8
0
12
24
36
48
60
72
84
96

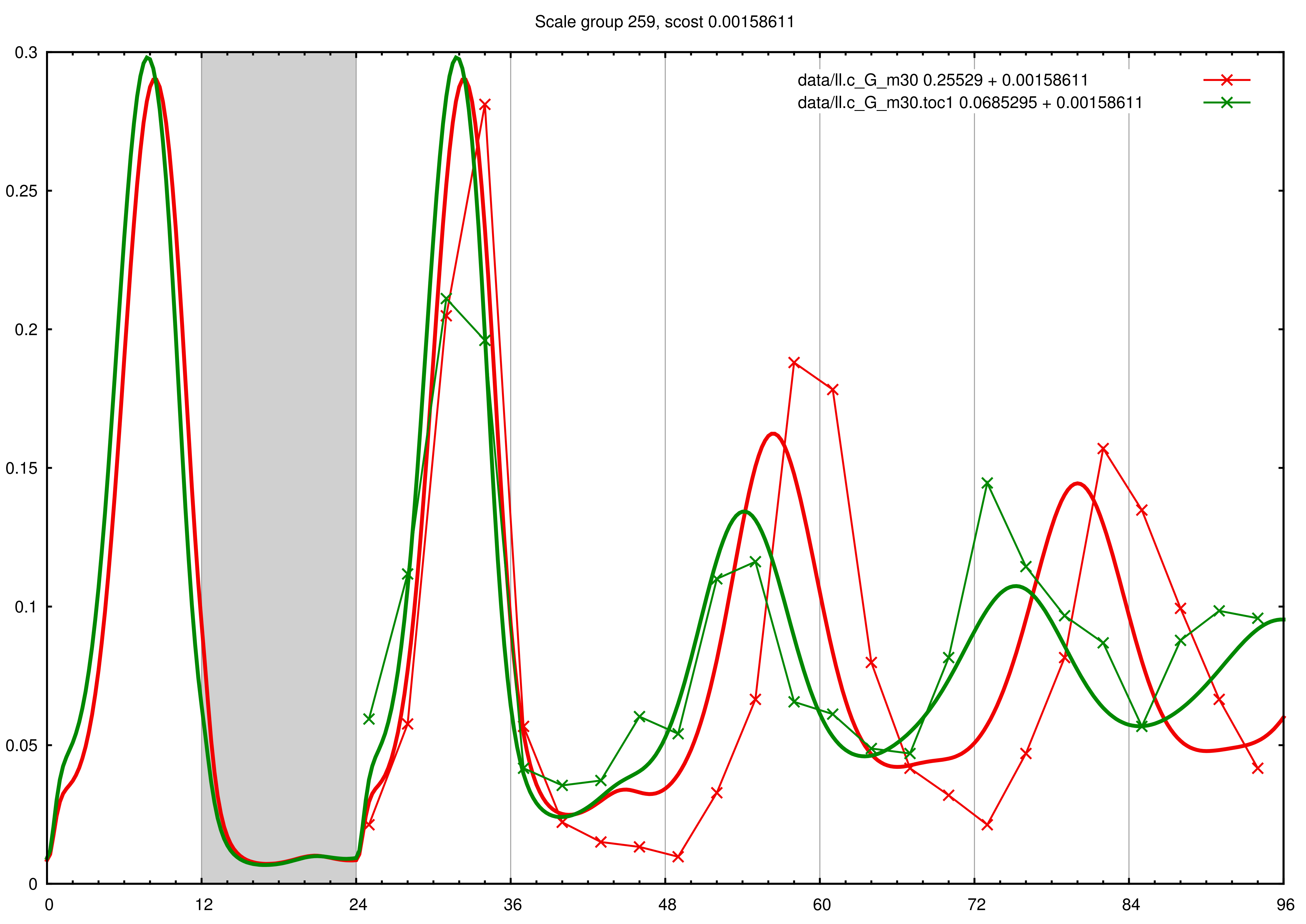
Scale group 259, scost 0.00158611
data/ll.c_G_m30 0.25529 + 0.00158611
data/ll.c_G_m30.toc1 0.0685295 + 0.00158611
0.3
0.25
0.2
0.15
0.1
0.05
0
0
12
24
36
48
60
72
84
96

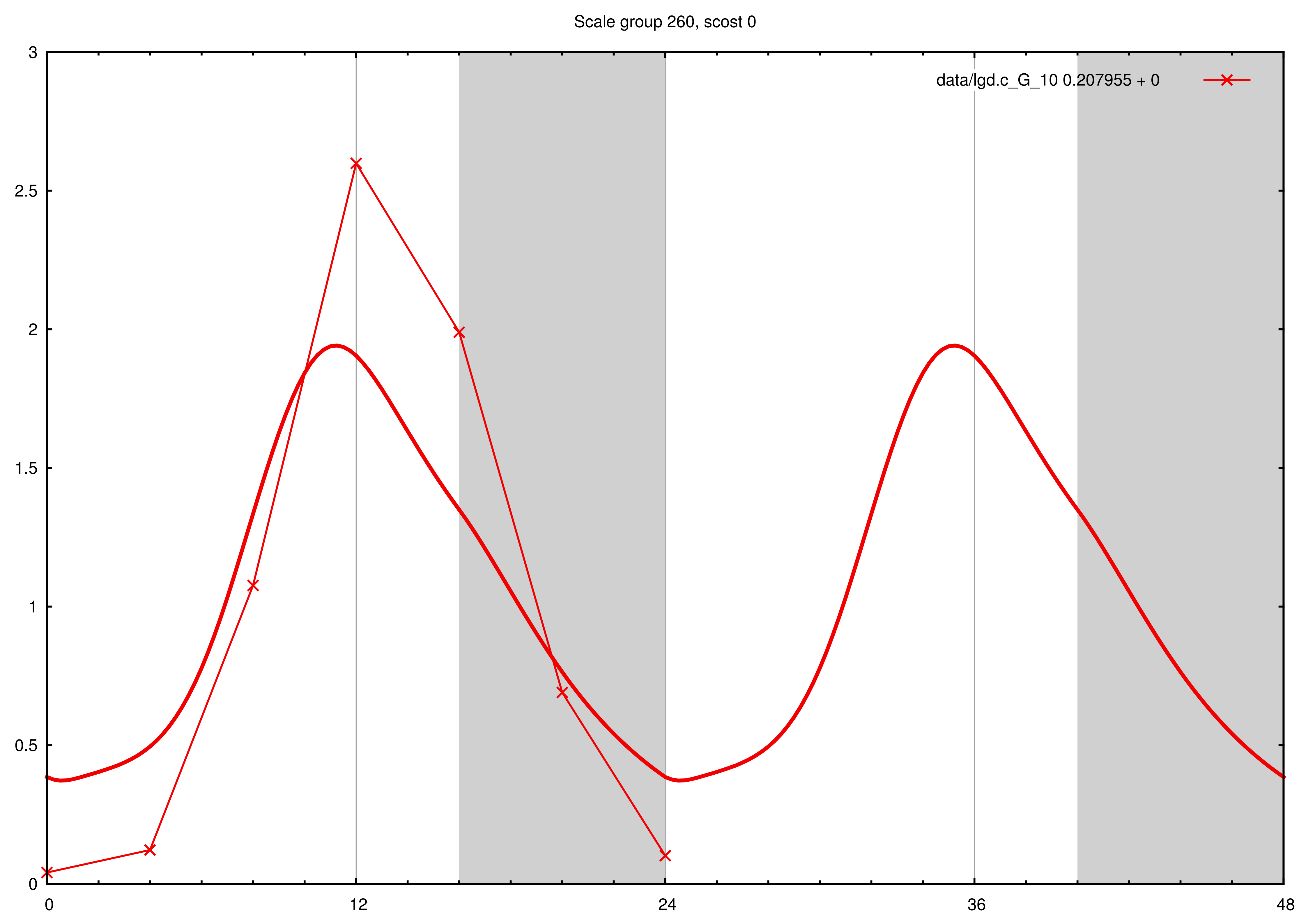
Scale group 260, scost 0
data/lgd.c_G_10 0.207955 + 0
0
0.5
1
1.5
2
2.5
3
0
12
24
36
48

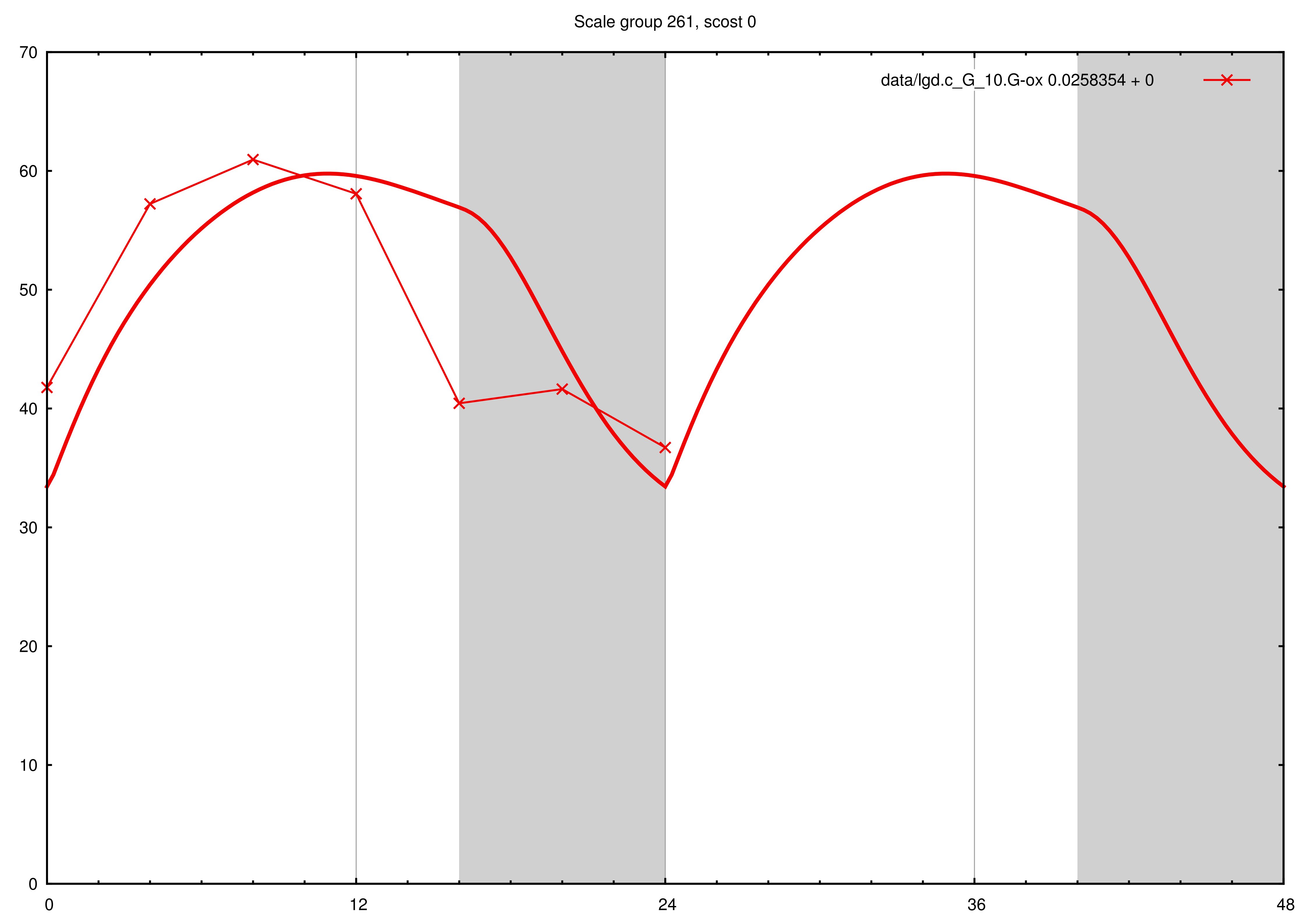

Scale group 261, scost 0
data/lgd.c_G_10.G-ox 0.0258354 + 0
0
10
20
30
40
50
60
70
0
12
24
36
48

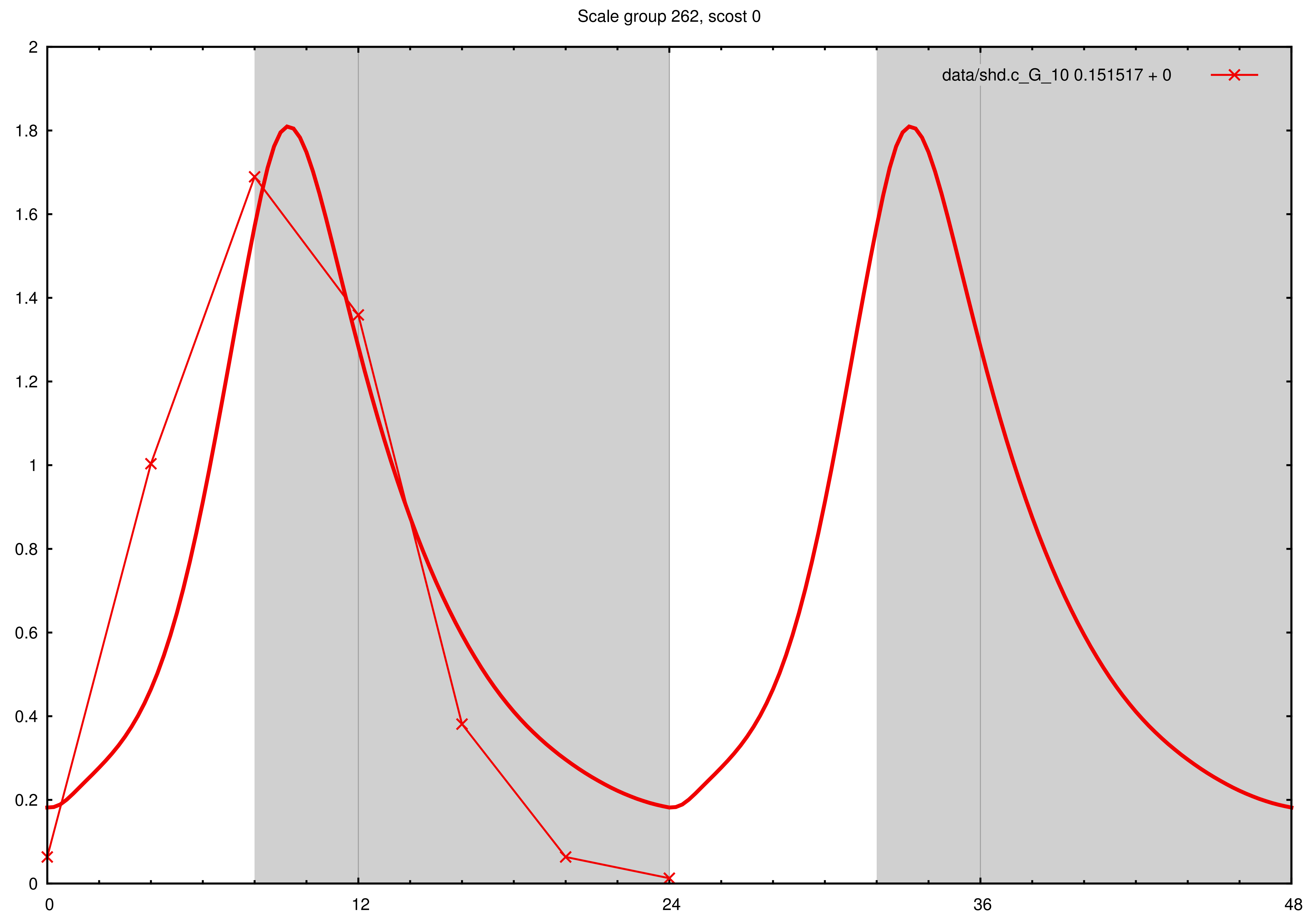

Scale group 262, scost 0
data/shd.c_G_10 0.151517 + 0
0
0.2
0.4
0.6
0.8
1
1.2
1.4
1.6
1.8
2
0
12
24
36
48

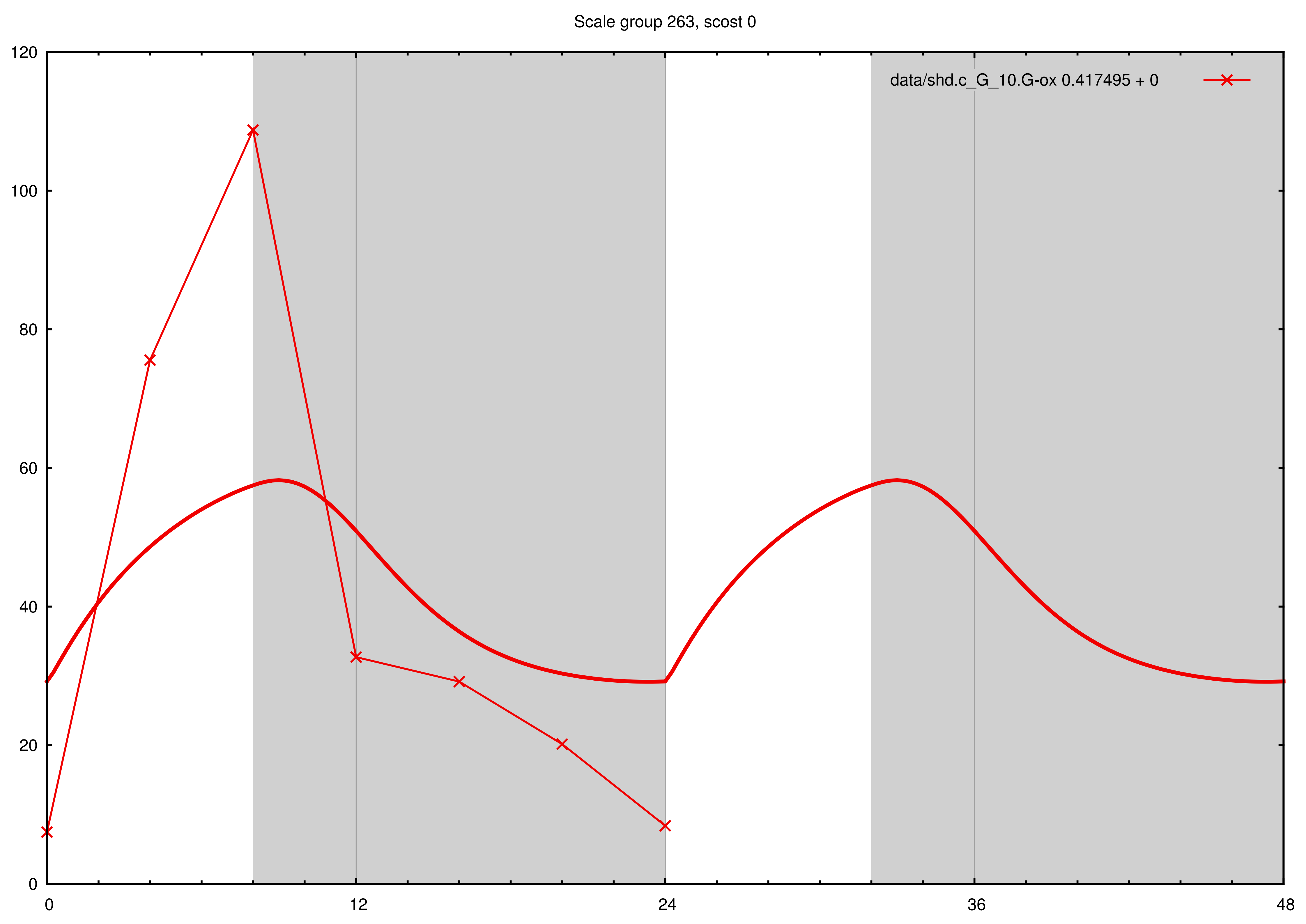
Scale group 263, scost 0
data/shd.c_G_10.G-ox 0.417495 + 0
0
20
40
60
80
100
120
0
12
24
36
48

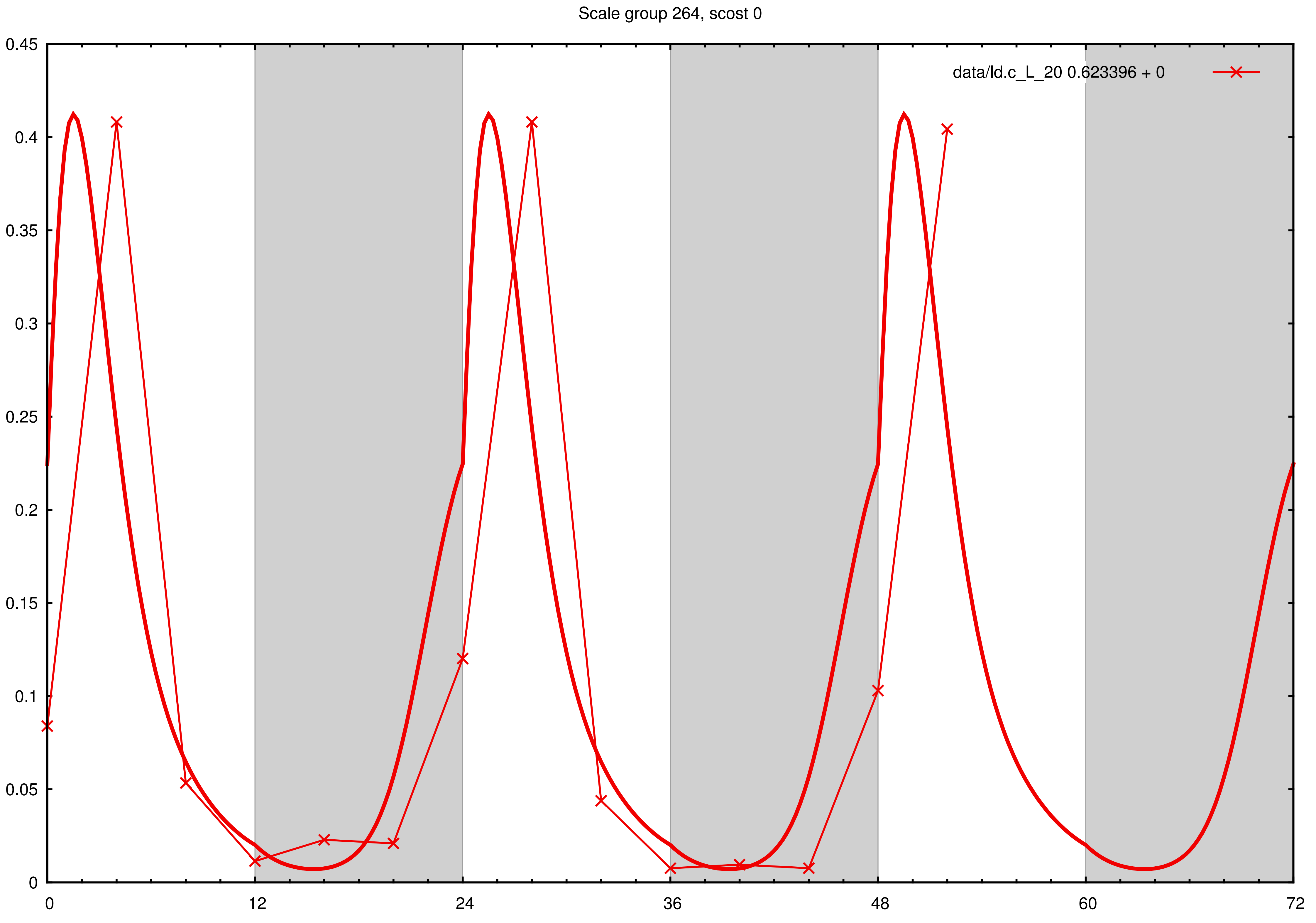
Scale group 264, scost 0
data/ld.c_L_20 0.623396 + 0
0.45
0.4
0.35
0.3
0.25
0.2
0.15
0.1
0.05
0
0
12
24
36
48
60
72

Scale group 265, scost 0

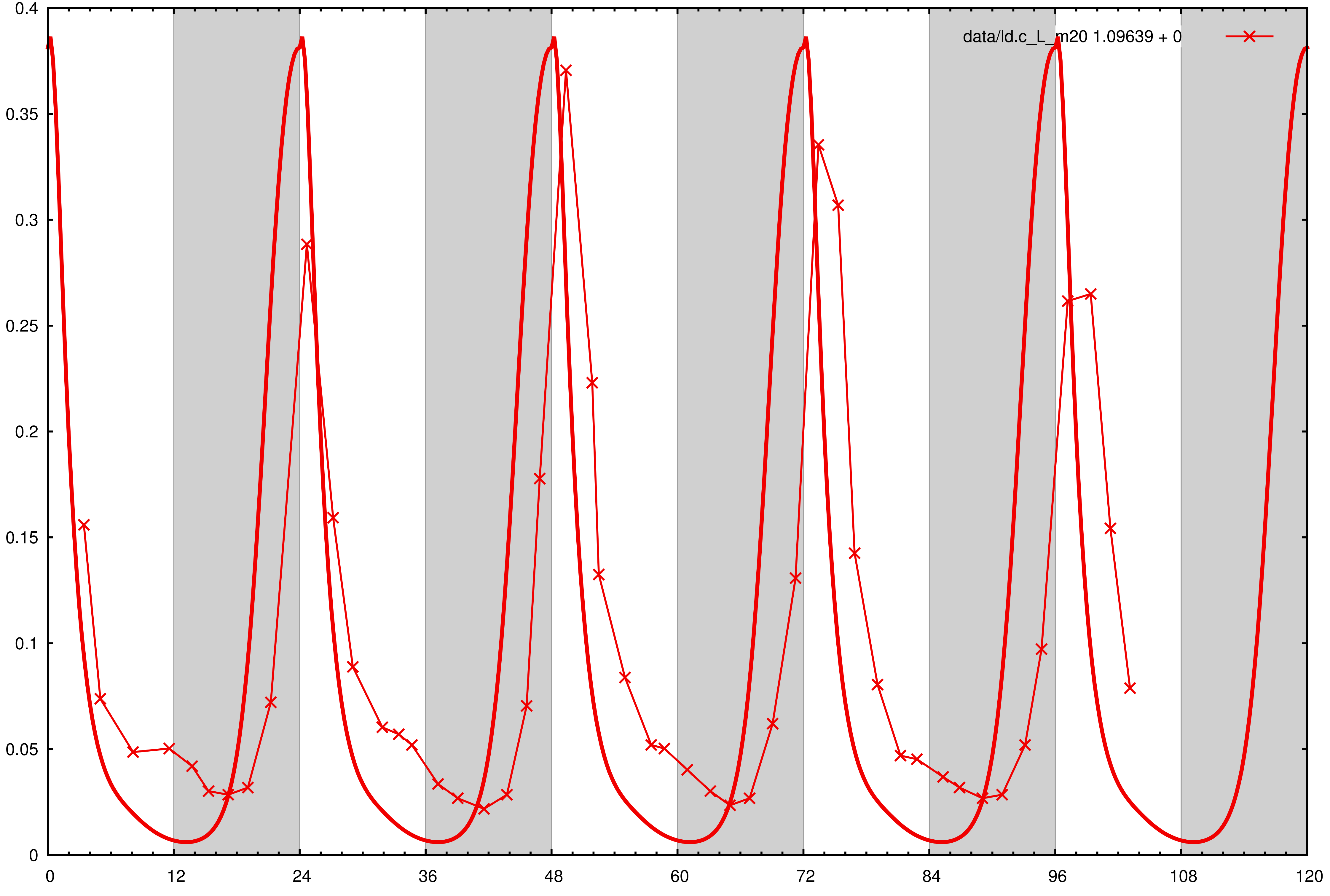

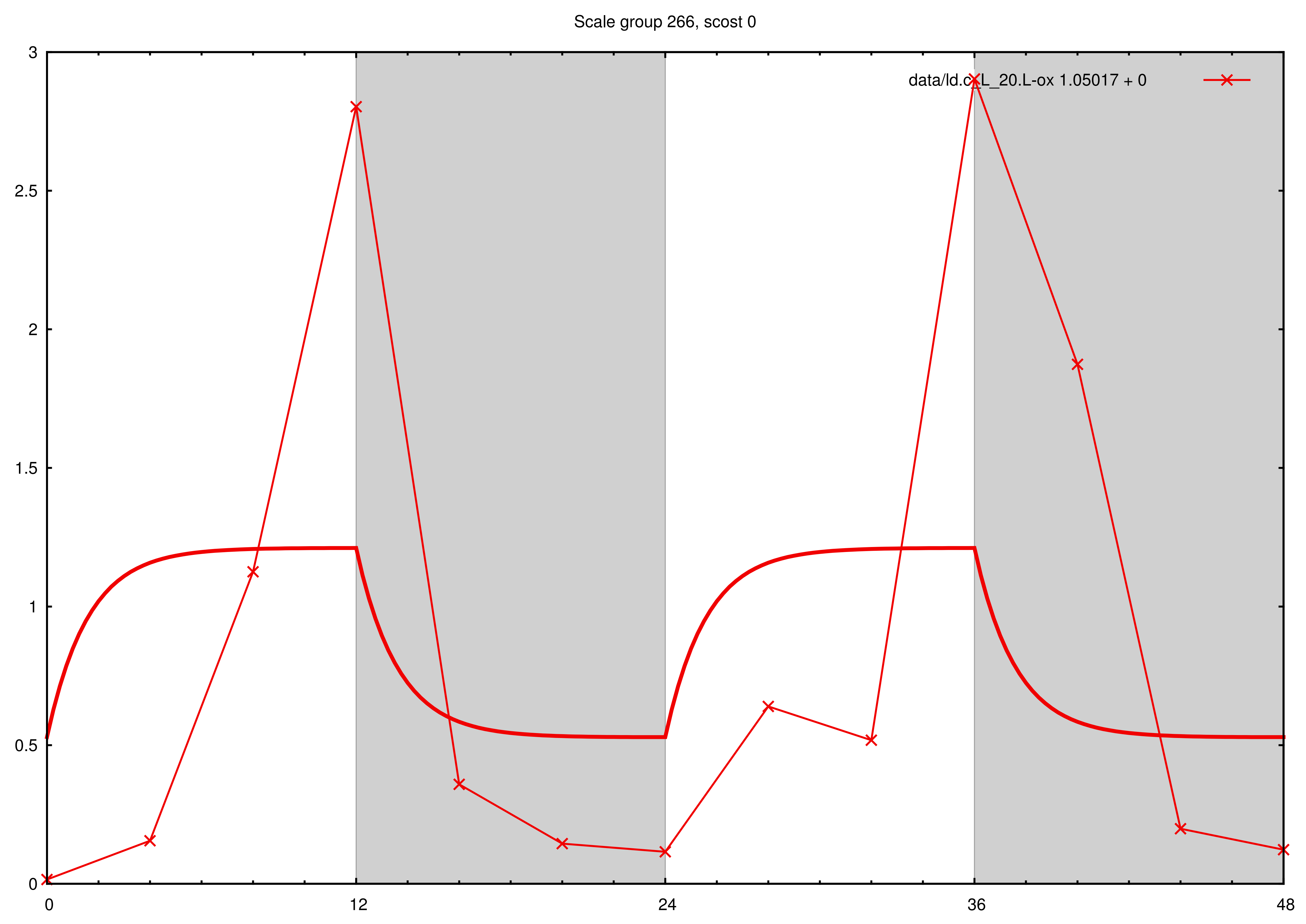

Scale group 266, scost 0
data/ld.c_L_20.L-ox 1.05017 + 0
3
2.5
2
1.5
1
0.5
0
0
12
24
36
48

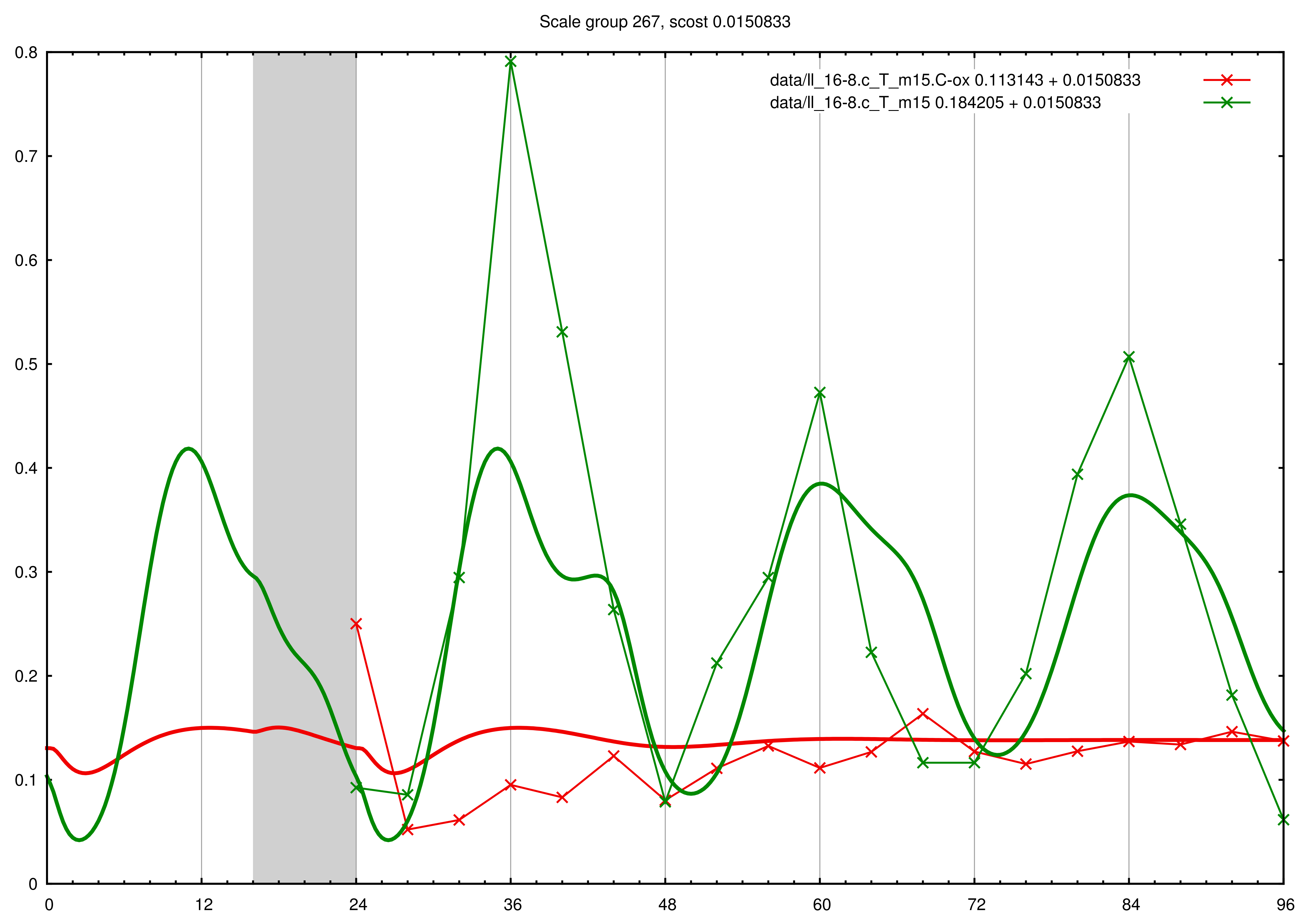

Scale group 267, scost 0.0150833
data/ll_16-8.c_T_m15.C-ox 0.113143 + 0.0150833
data/ll_16-8.c_T_m15 0.184205 + 0.0150833
0.8
0.7
0.6
0.5
0.4
0.3
0.2
0.1
0
0
12
24
36
48
60
72
84
96

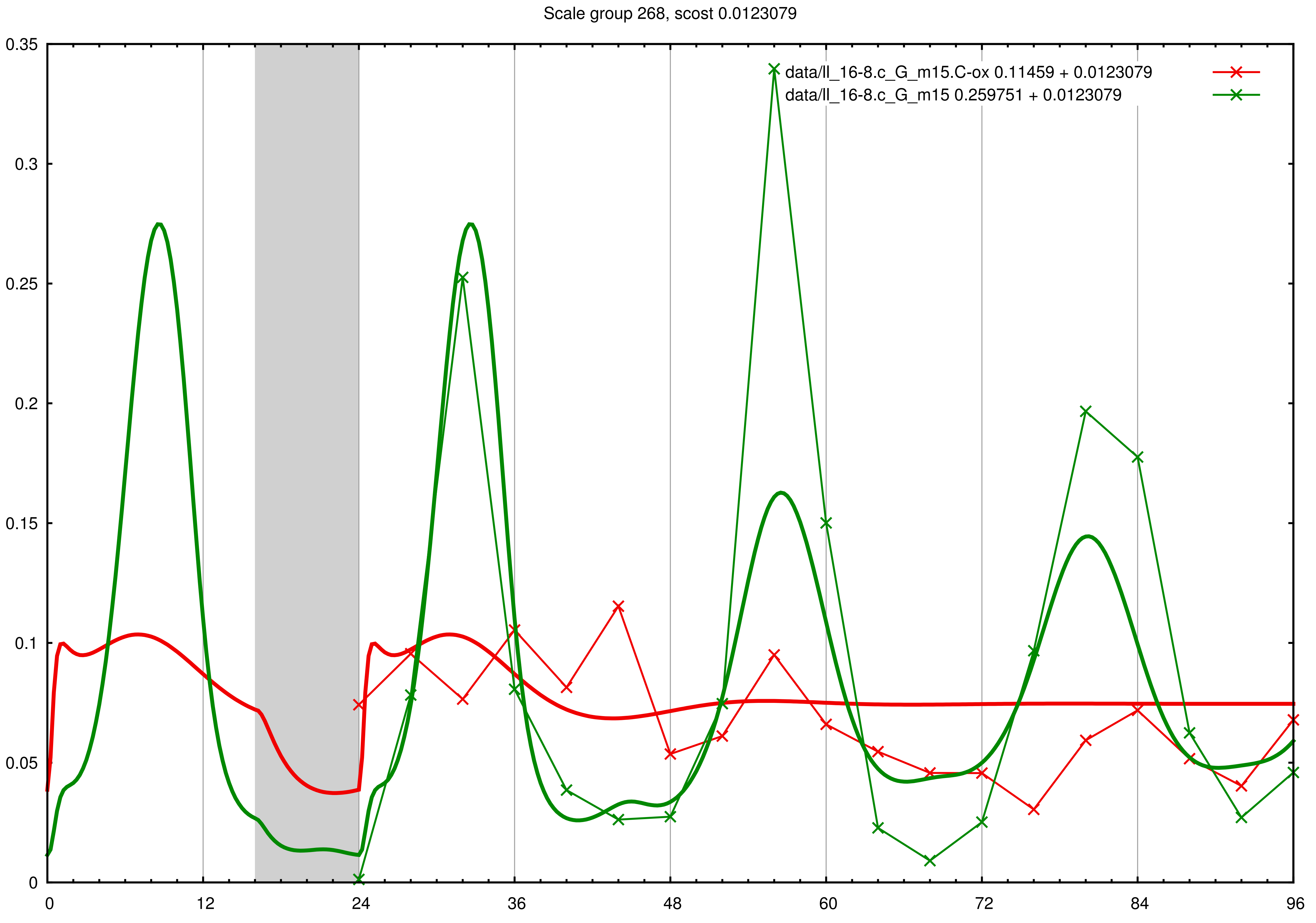

Scale group 268, scost 0.0123079
data/ll_16-8.c_G_m15.C-ox 0.11459 + 0.0123079
data/ll_16-8.c_G_m15 0.259751 + 0.0123079
0.35
0.3
0.25
0.2
0.15
0.1
0.05
0
0
12
24
36
48
60
72
84
96

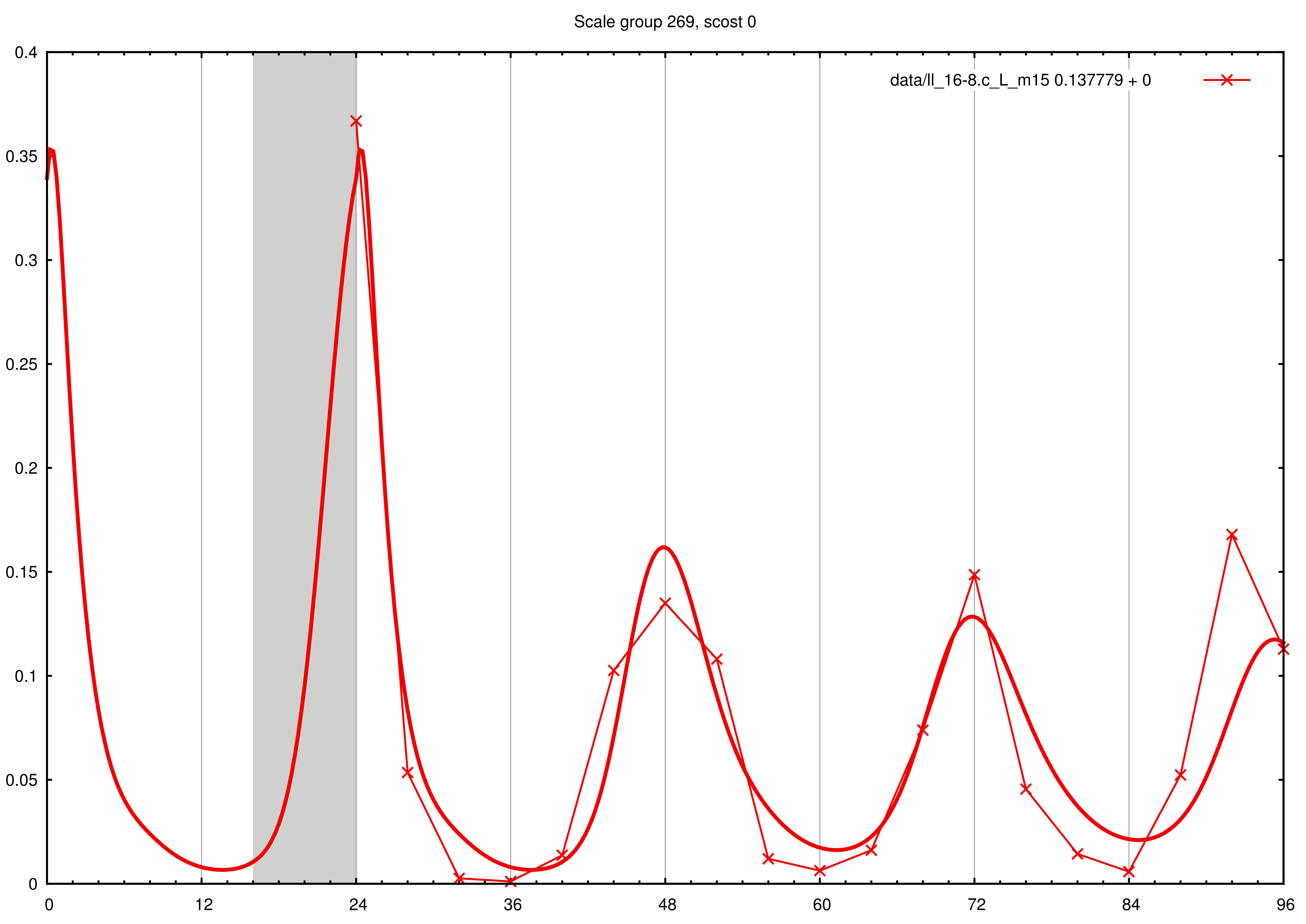
Scale group 269, scost 0
data/ll_16-8.c_L_m15 0.137779 + 0
0.4
0.35
0.3
0.25
0.2
0.15
0.1
0.05
0
0
12
24
36
48
60
72
84
96

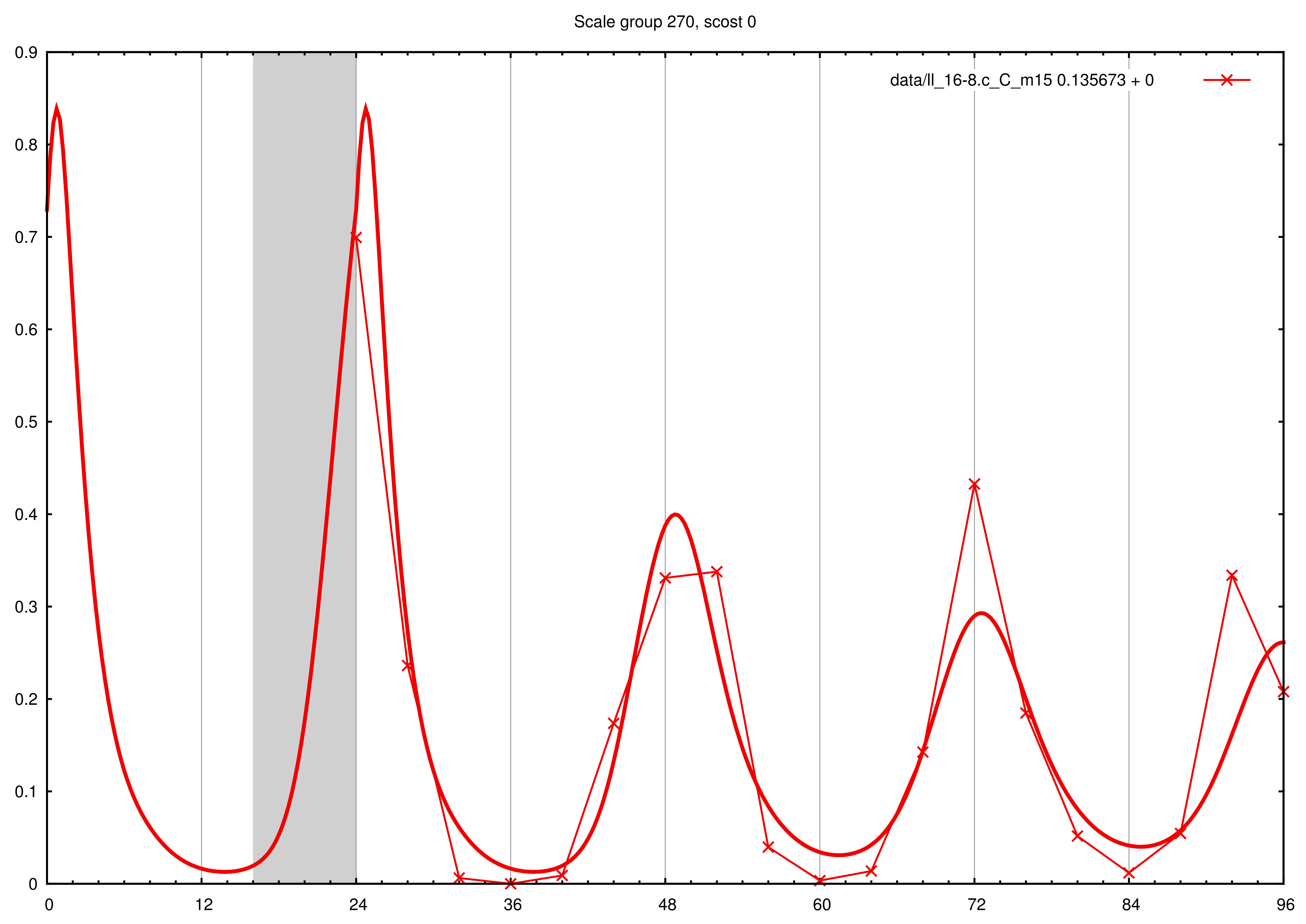

Scale group 270, scost 0
data/ll_16-8.c_C_m15 0.135673 + 0
0
0.1
0.2
0.3
0.4
0.5
0.6
0.7
0.8
0.9
0
12
24
36
48
60
72
84
96

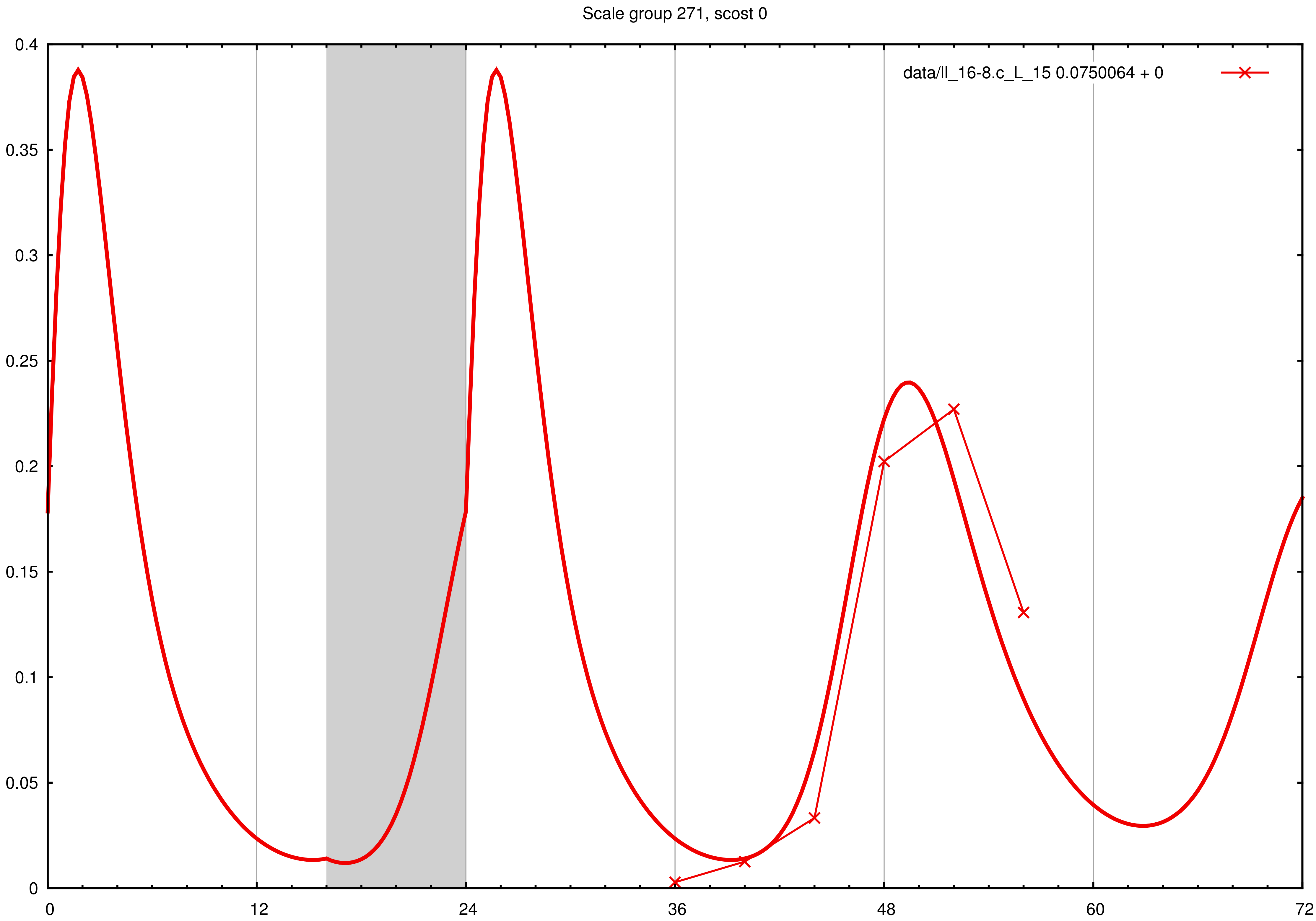
Scale group 271, scost 0
data/ll_16-8.c_L_15 0.0750064 + 0
0.4
0.35
0.3
0.25
0.2
0.15
0.1
0.05
0
0
12
24
36
48
60
72

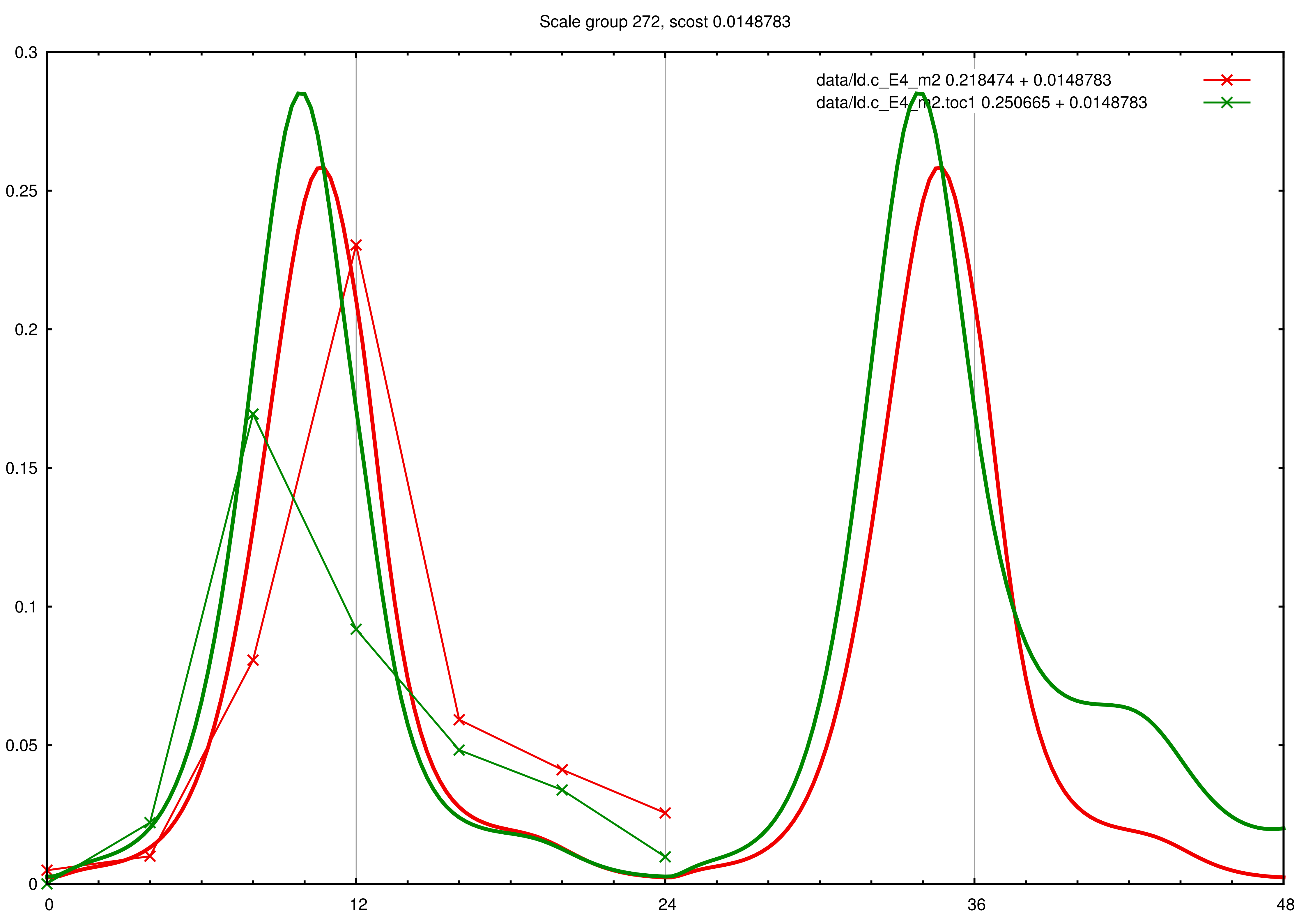
Scale group 272, scost 0.0148783
data/ld.c_E4_m2 0.218474 + 0.0148783
data/ld.c_E4_m2.toc1 0.250665 + 0.0148783
0.3
0.25
0.2
0.15
0.1
0.05
0
0
12
24
36
48

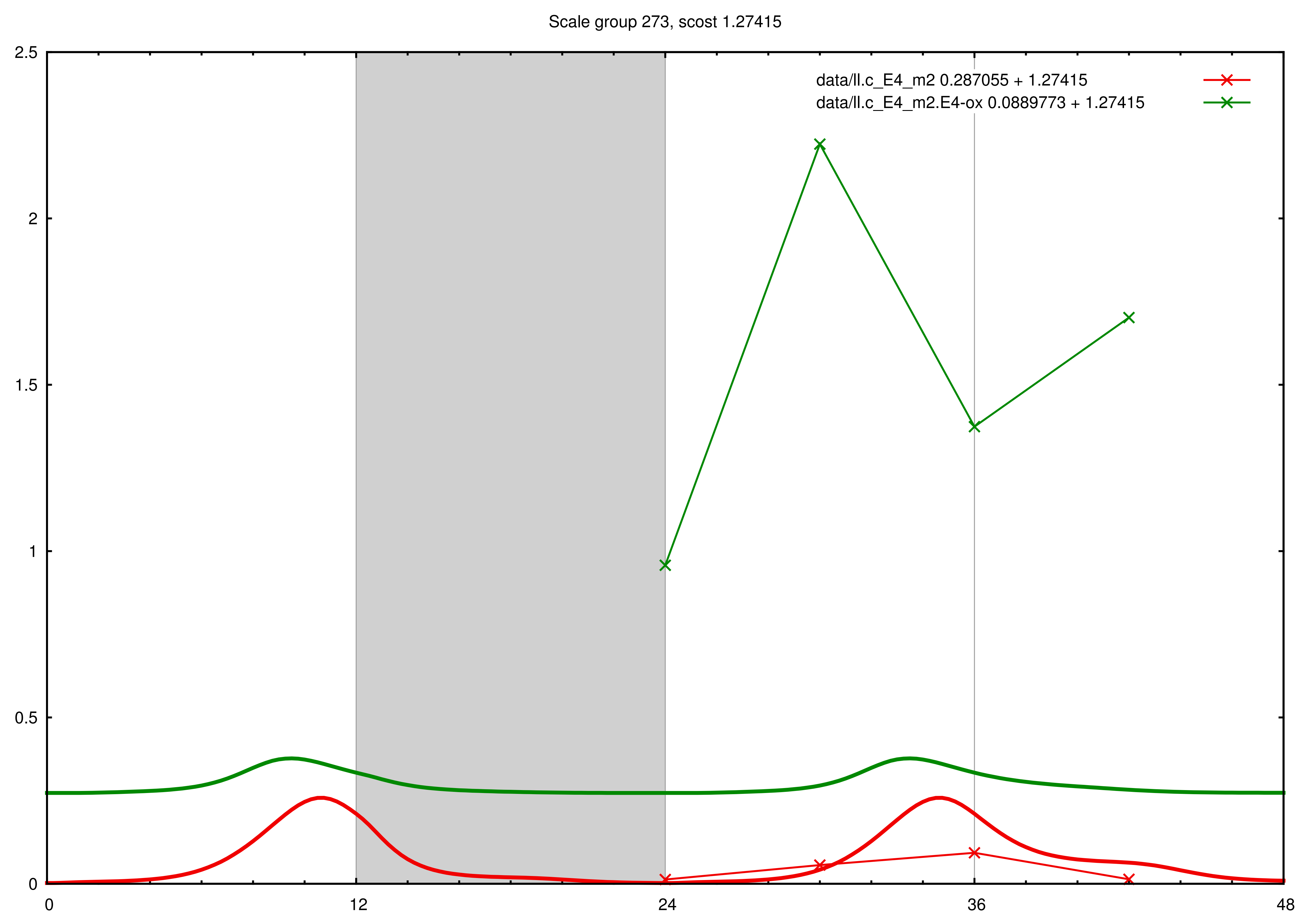

Scale group 273, scost 1.27415
data/ll.c_E4_m2 0.287055 + 1.27415
data/ll.c_E4_m2.E4-ox 0.0889773 + 1.27415
2.5
2
1.5
1
0.5
0
0
12
24
36
48

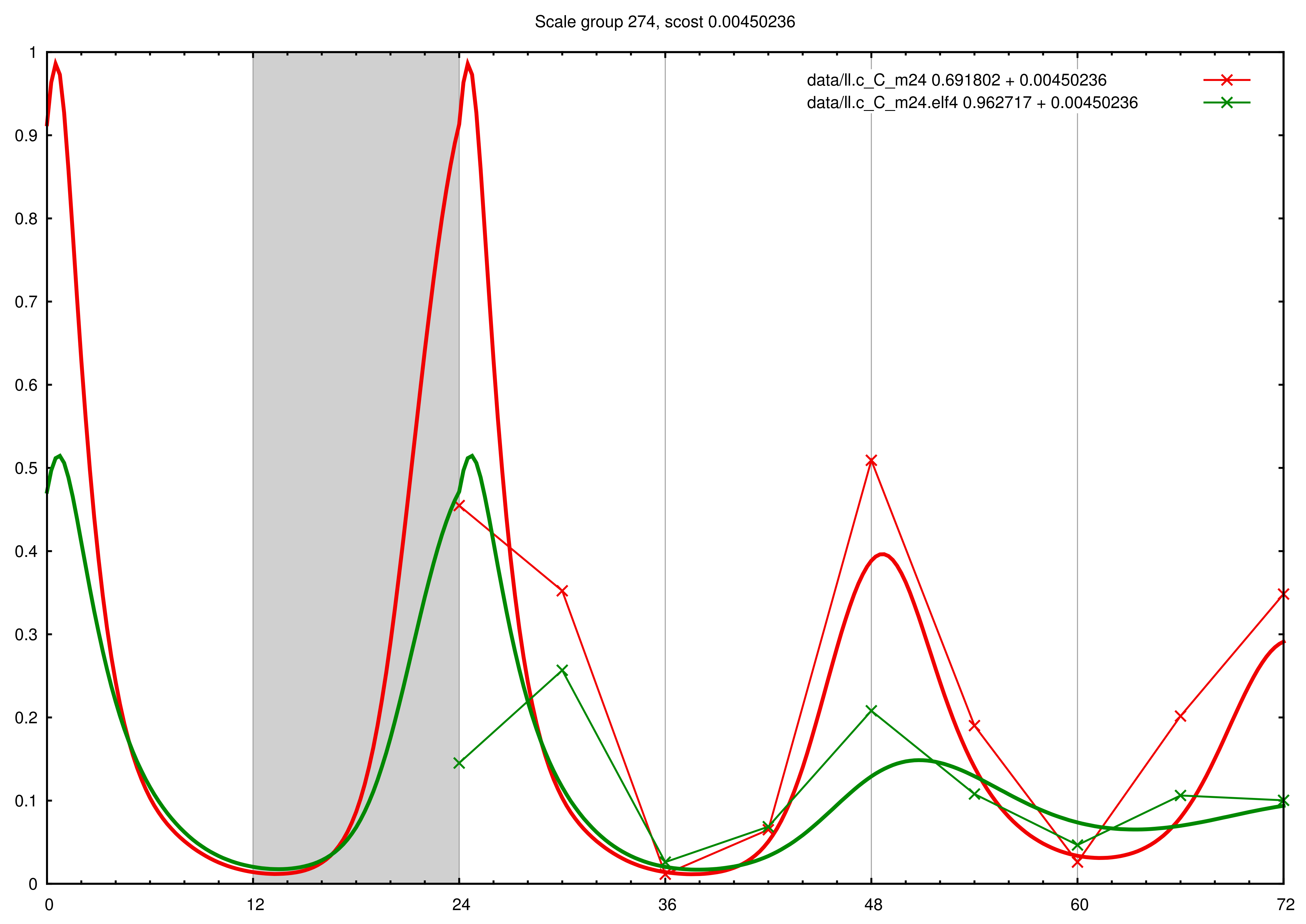
Scale group 274, scost 0.00450236
data/ll.c_C_m24 0.691802 + 0.00450236
data/ll.c_C_m24.elf4 0.962717 + 0.00450236
0
0.1
0.2
0.3
0.4
0.5
0.6
0.7
0.8
0.9
1
0
12
24
36
48
60
72

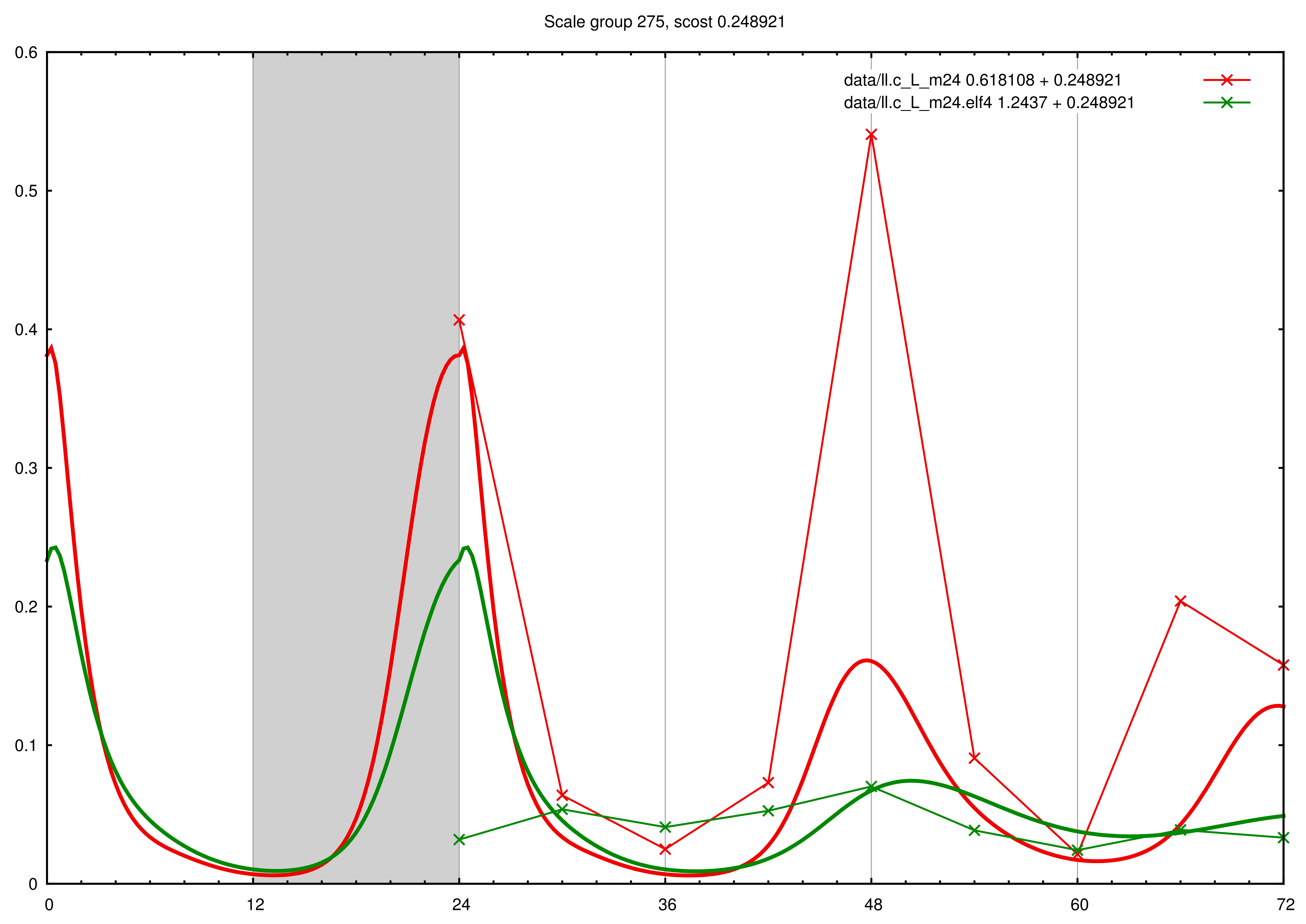
Scale group 275, scost 0.248921
data/ll.c_L_m24 0.618108 + 0.248921
data/ll.c_L_m24.elf4 1.2437 + 0.248921
0.6
0.5
0.4
0.3
0.2
0.1
0
0
12
24
36
48
60
72

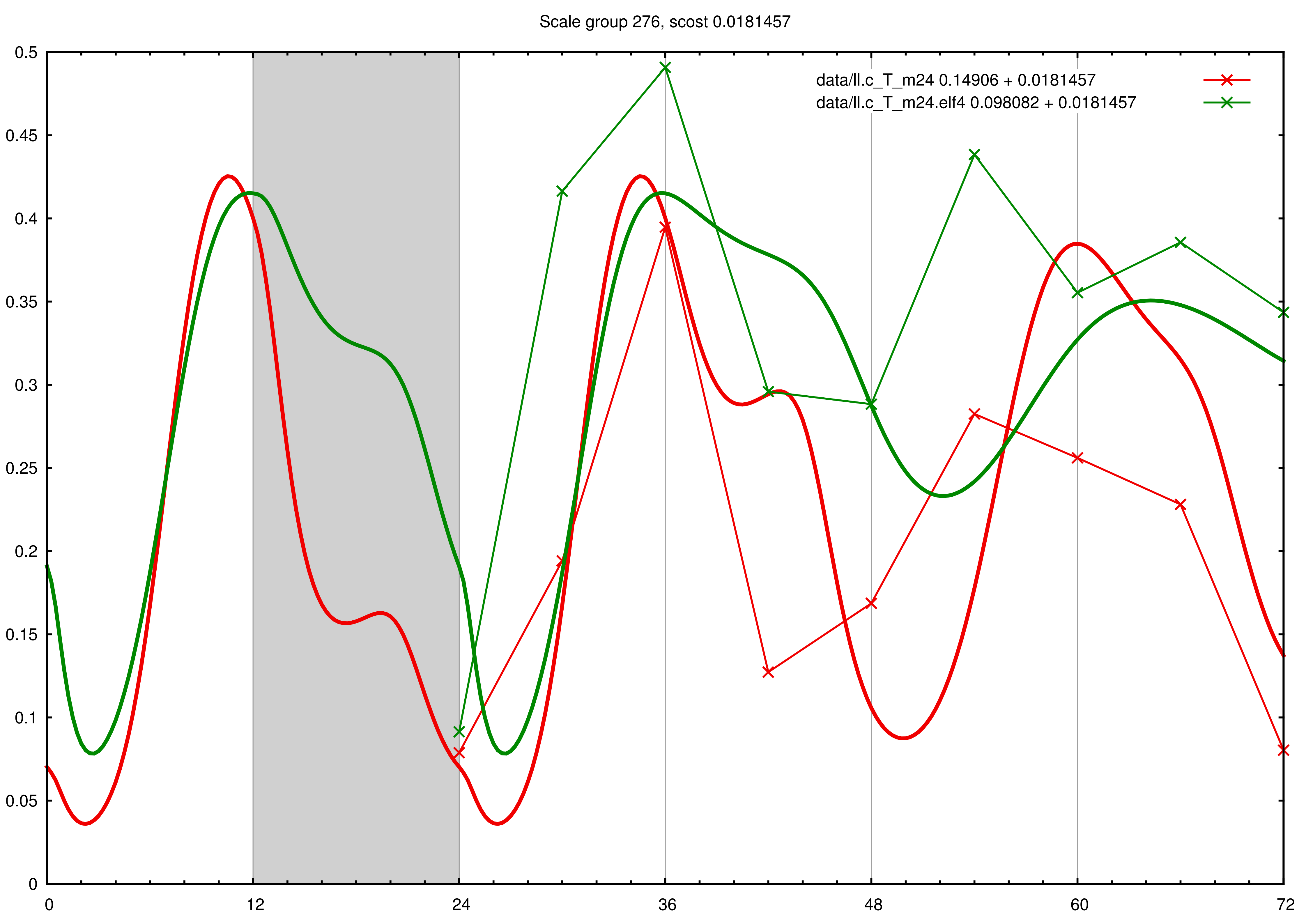
Scale group 276, scost 0.0181457
data/ll.c_T_m24 0.14906 + 0.0181457
data/ll.c_T_m24.elf4 0.098082 + 0.0181457
0
0.05
0.1
0.15
0.2
0.25
0.3
0.35
0.4
0.45
0.5
0
12
24
36
48
60
72

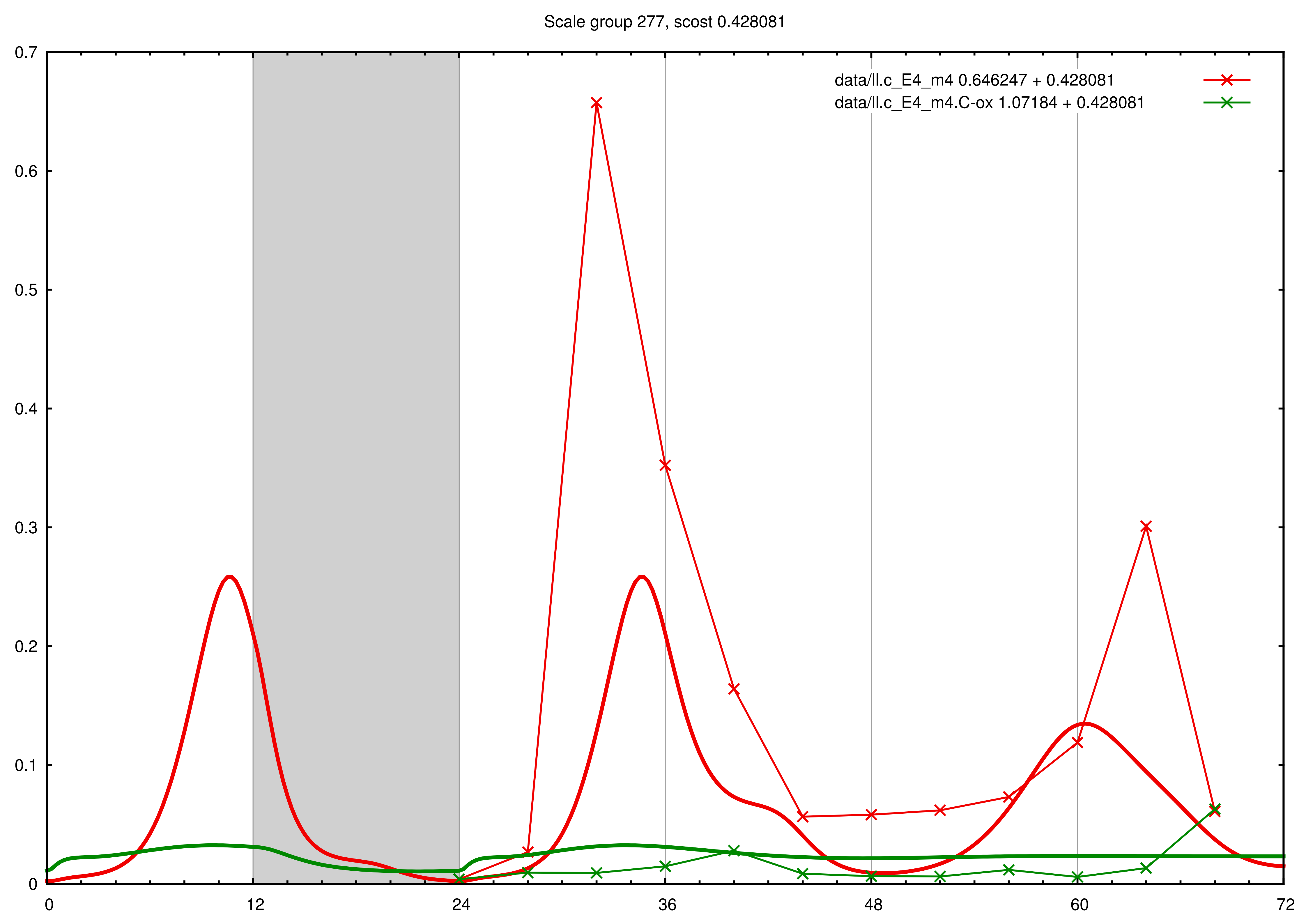
Scale group 277, scost 0.428081
data/ll.c_E4_m4 0.646247 + 0.428081
data/ll.c_E4_m4.C-ox 1.07184 + 0.428081
0
0.1
0.2
0.3
0.4
0.5
0.6
0.7
0
12
24
36
48
60
72

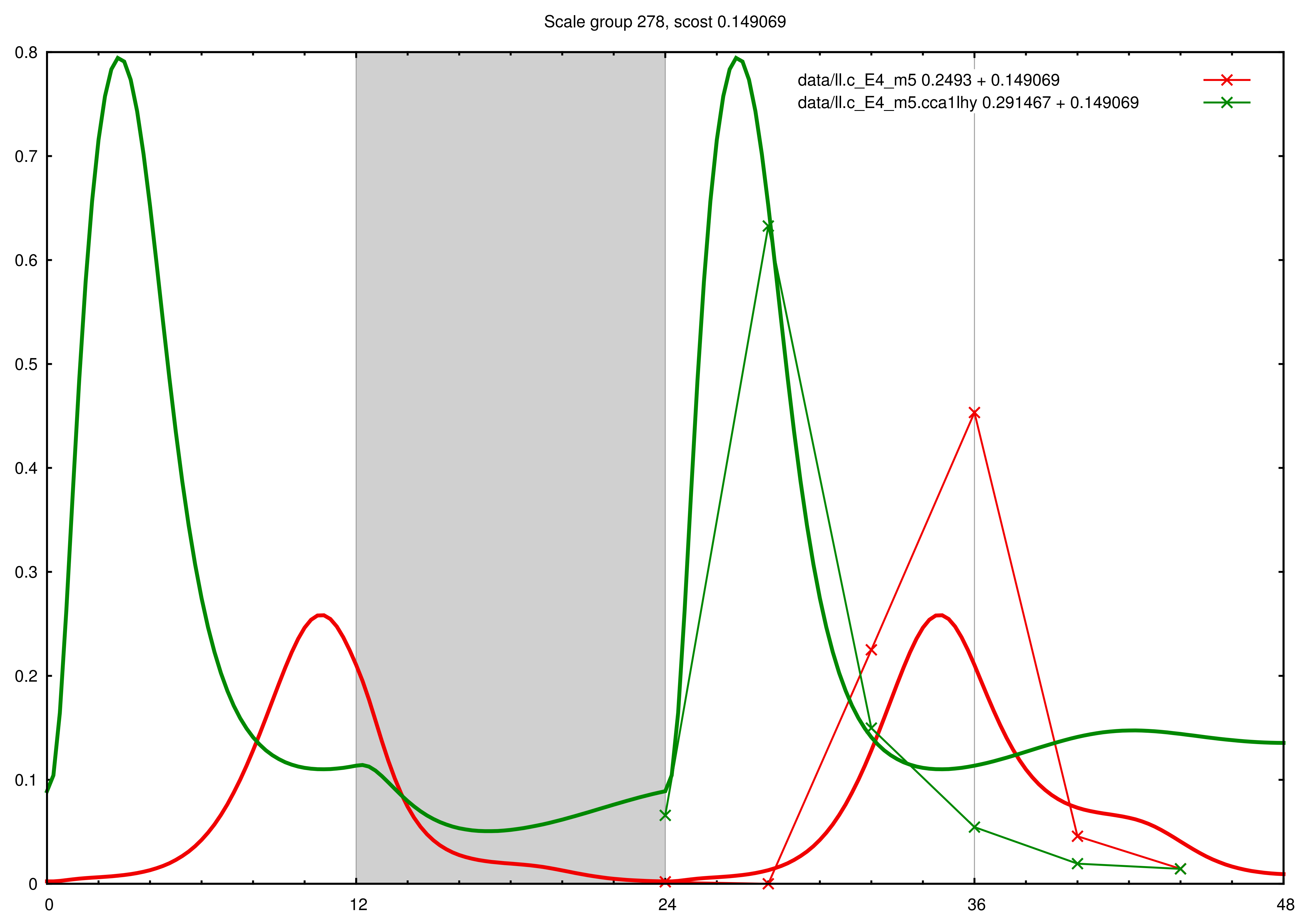
Scale group 278, scost 0.149069
data/ll.c_E4_m5 0.2493 + 0.149069
data/ll.c_E4_m5.cca1lhy 0.291467 + 0.149069
0.8
0.7
0.6
0.5
0.4
0.3
0.2
0.1
0
0
12
24
36
48

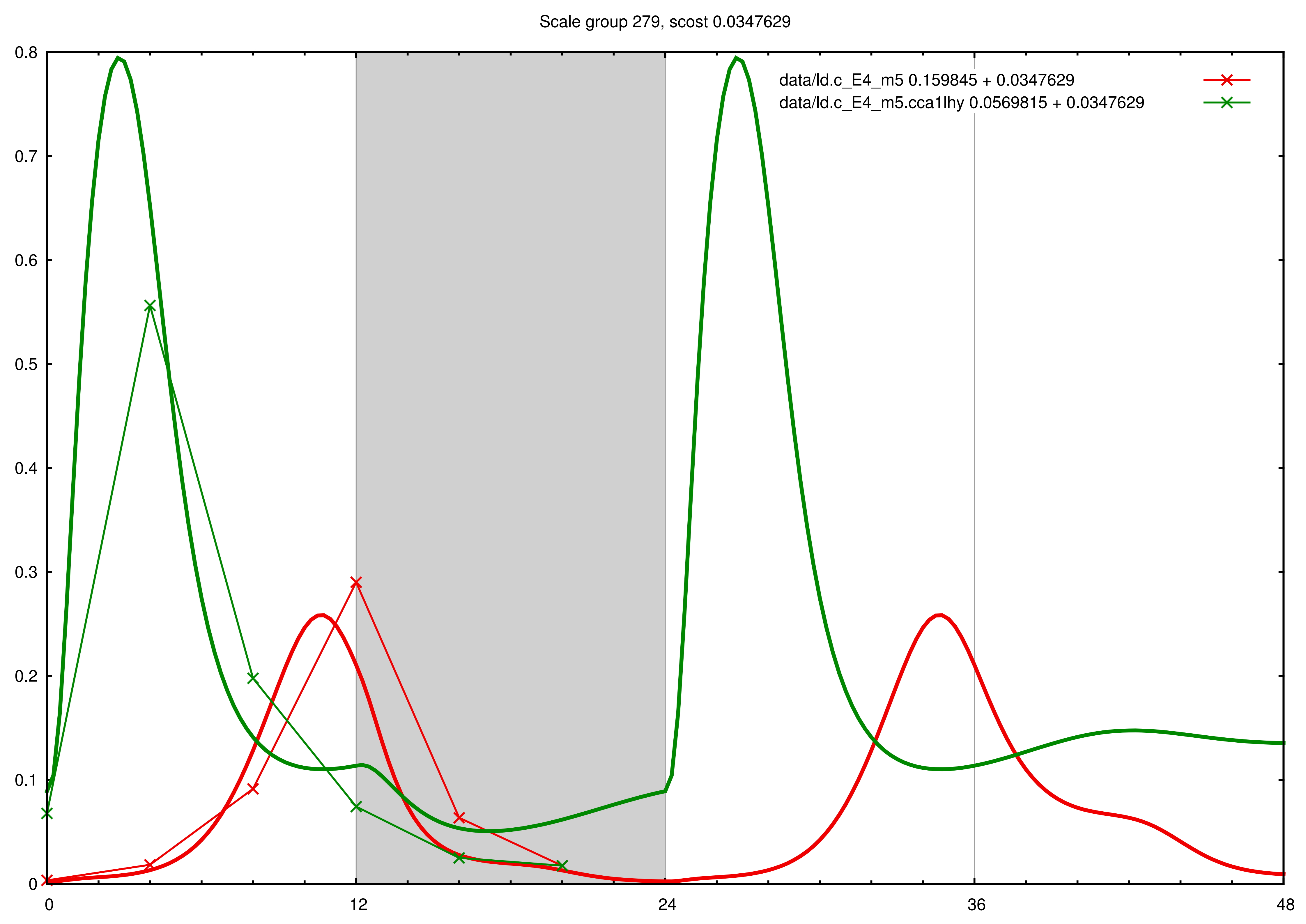

Scale group 279, scost 0.0347629
data/ld.c_E4_m5 0.159845 + 0.0347629
data/ld.c_E4_m5.cca1lhy 0.0569815 + 0.0347629
0.8
0.7
0.6
0.5
0.4
0.3
0.2
0.1
0
0
12
24
36
48

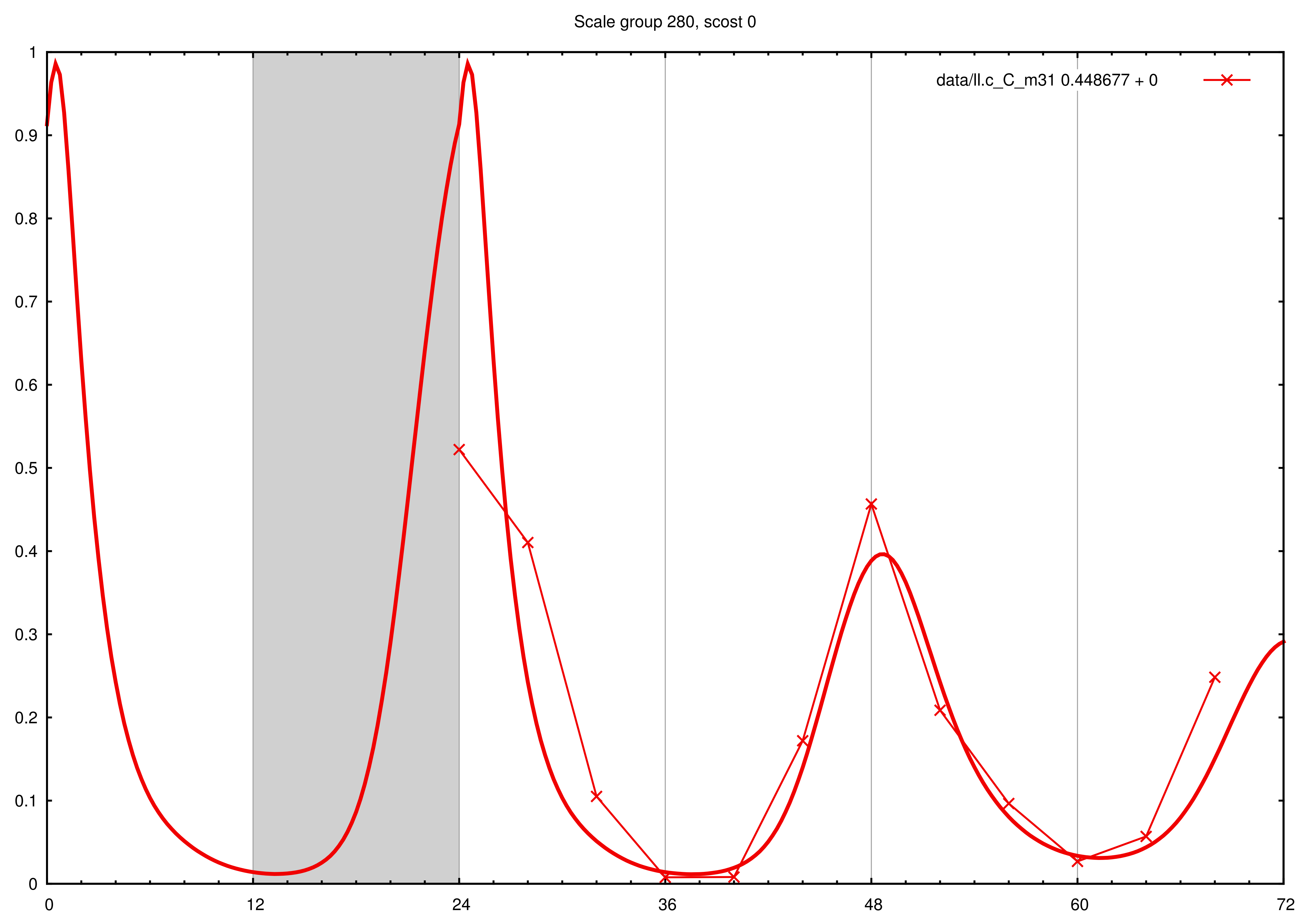

Scale group 280, scost 0
data/ll.c_C_m31 0.448677 + 0
0
0.1
0.2
0.3
0.4
0.5
0.6
0.7
0.8
0.9
1
0
12
24
36
48
60
72

Scale group 281, scost 0

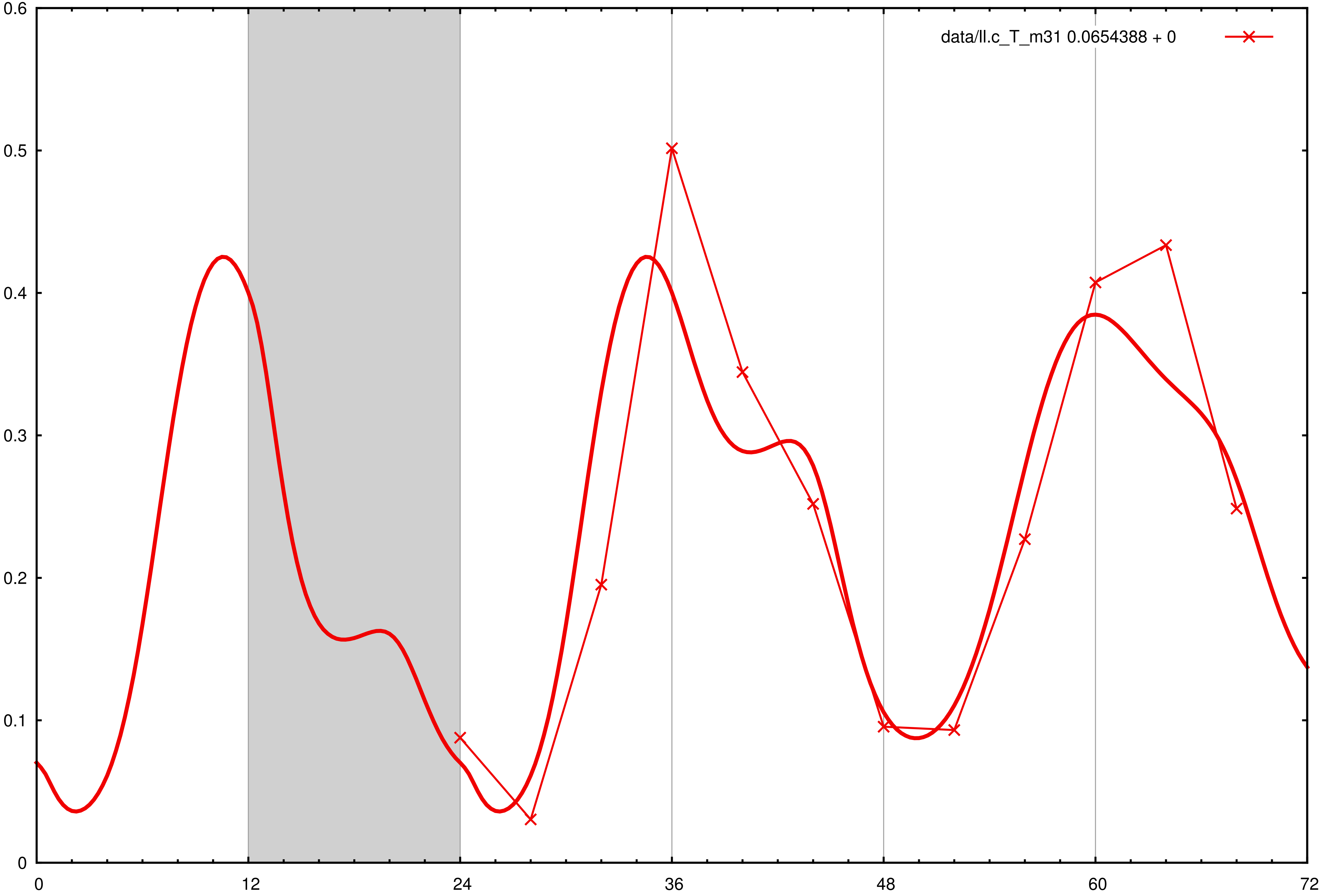

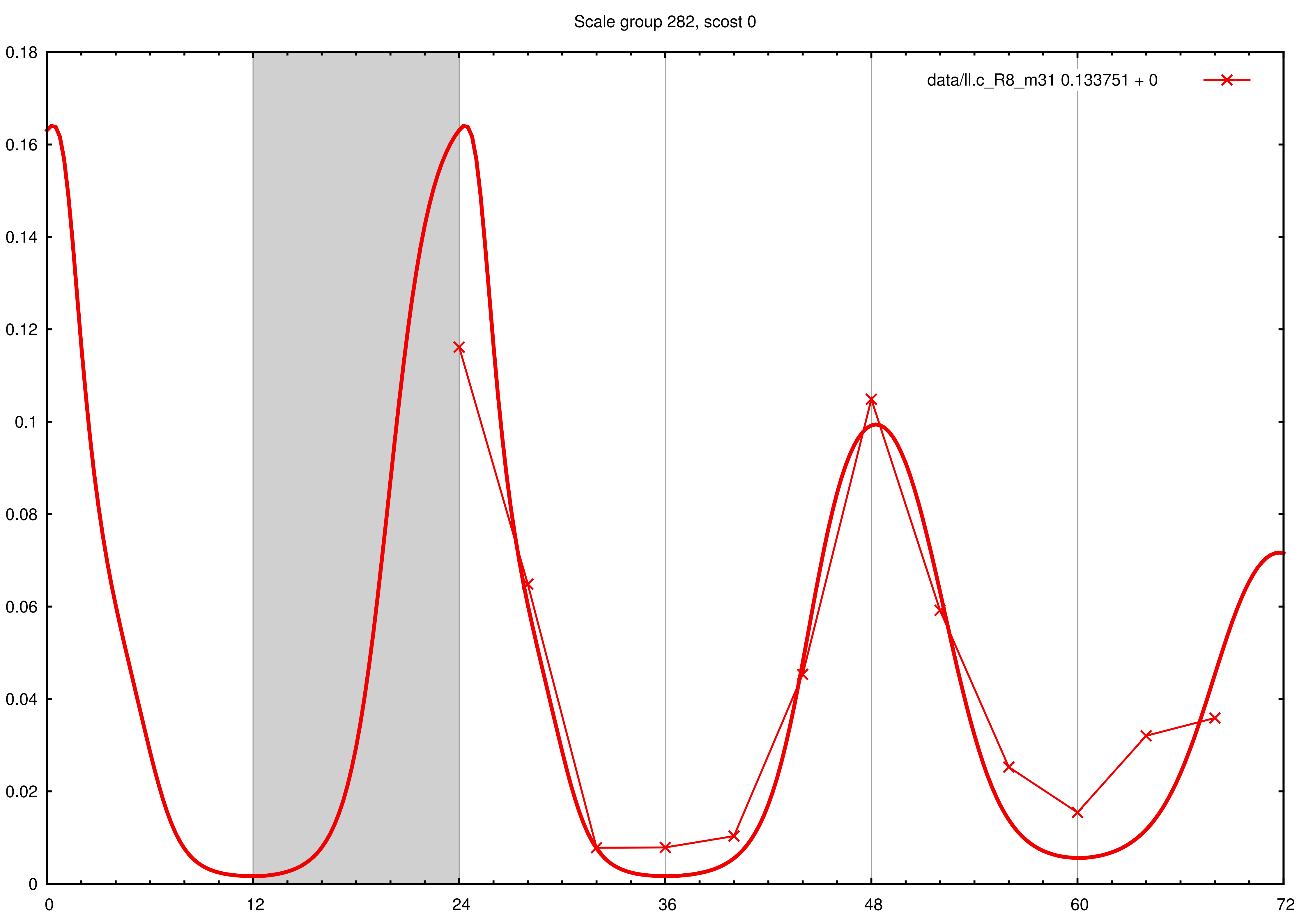

Scale group 282, scost 0
data/ll.c_R8_m31 0.133751 + 0
0.18
0.16
0.14
0.12
0.1
0.08
0.06
0.04
0.02
0
0
12
24
36
48
60
72

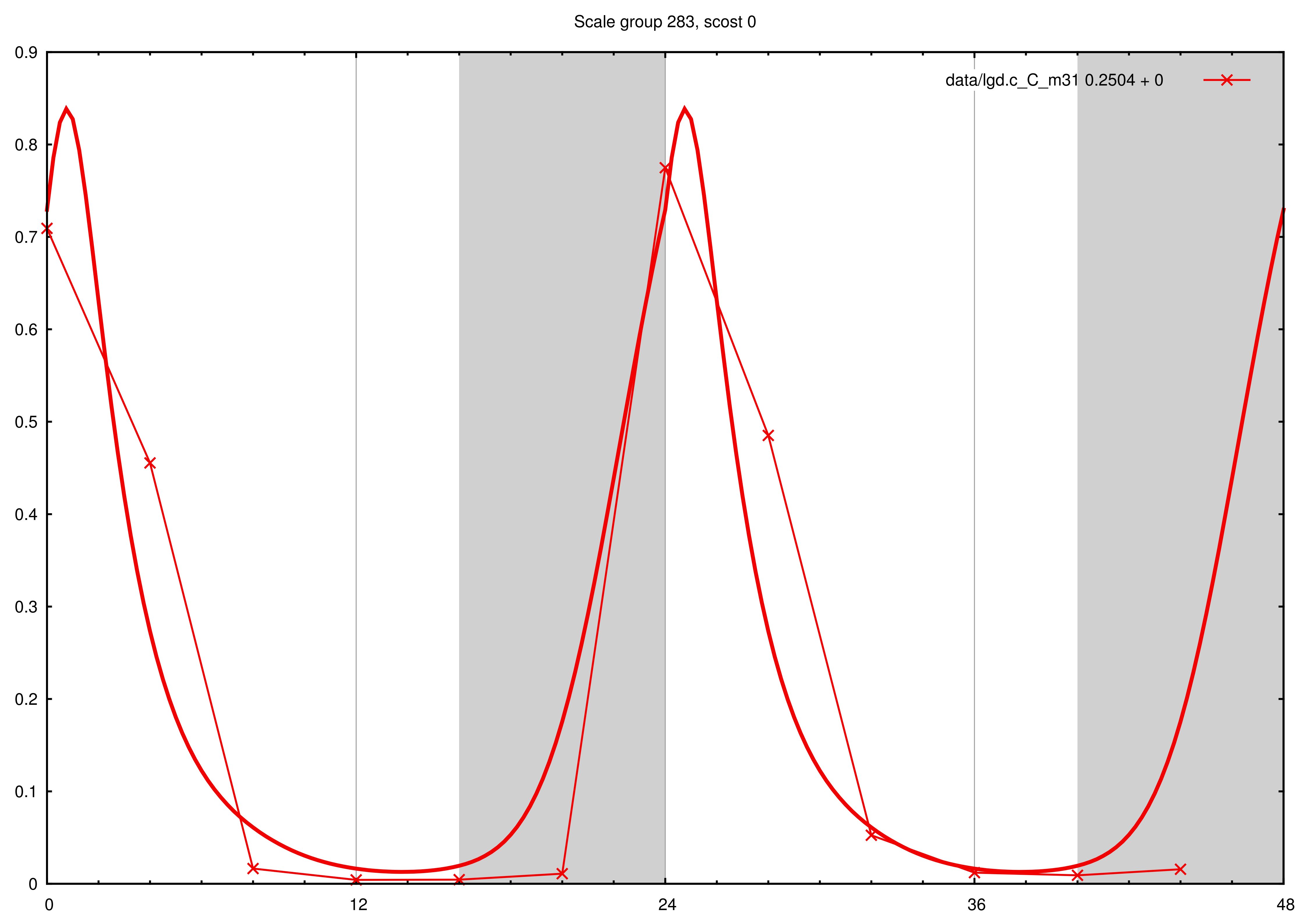
Scale group 283, scost 0
data/lgd.c_C_m31 0.2504 + 0
0.9
0.8
0.7
0.6
0.5
0.4
0.3
0.2
0.1
0
0
12
24
36
48

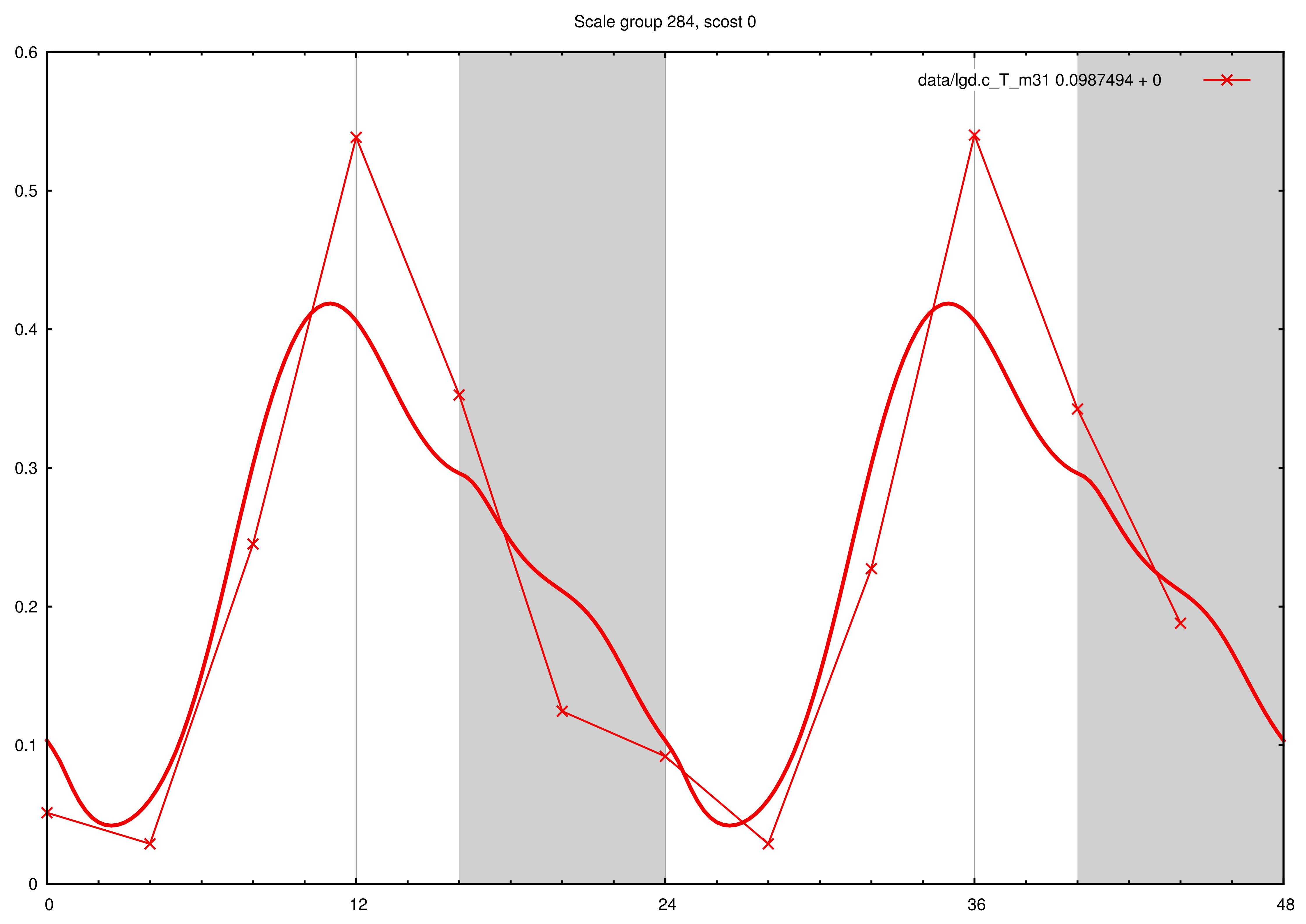

Scale group 284, scost 0
data/lgd.c_T_m31 0.0987494 + 0
0.6
0.5
0.4
0.3
0.2
0.1
0
0
12
24
36
48

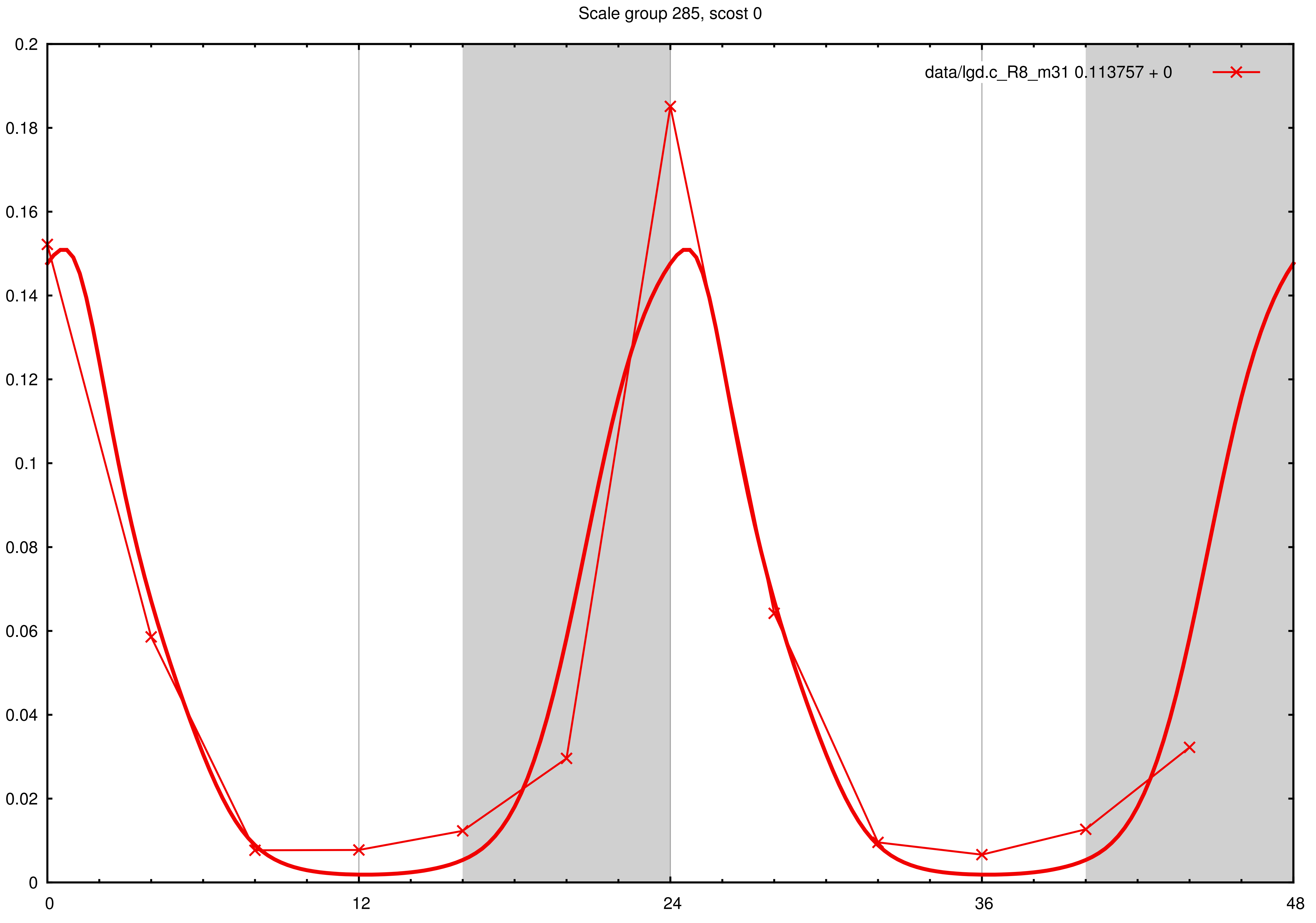
Scale group 285, scost 0
data/lgd.c_R8_m31 0.113757 + 0
0.2
0.18
0.16
0.14
0.12
0.1
0.08
0.06
0.04
0.02
0
0
12
24
36
48

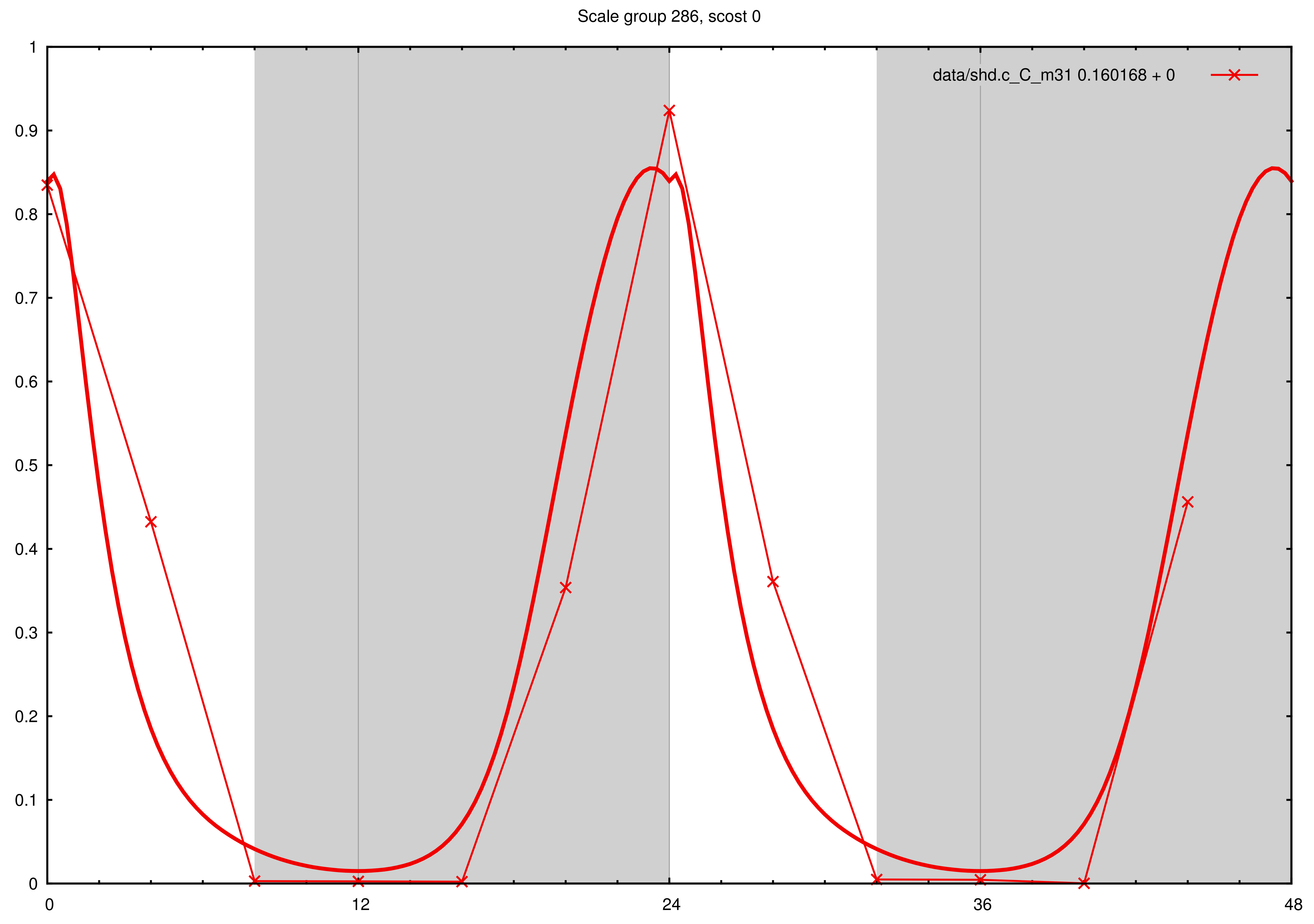

Scale group 286, scost 0
data/shd.c_C_m31 0.160168 + 0
0
0.1
0.2
0.3
0.4
0.5
0.6
0.7
0.8
0.9
1
0
12
24
36
48

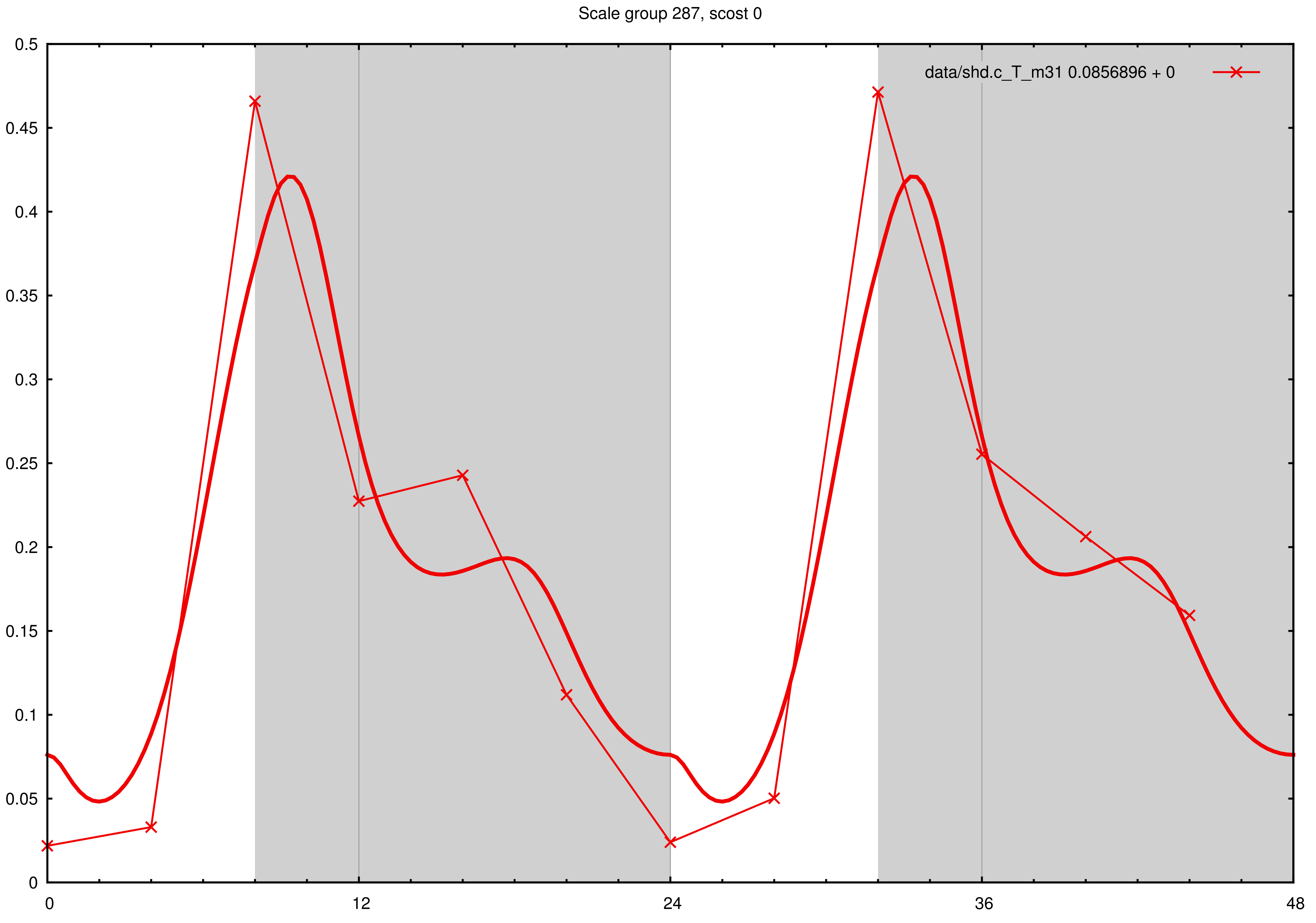
Scale group 287, scost 0
data/shd.c_T_m31 0.0856896 + 0
0.5
0.45
0.4
0.35
0.3
0.25
0.2
0.15
0.1
0.05
0
0
12
24
36
48

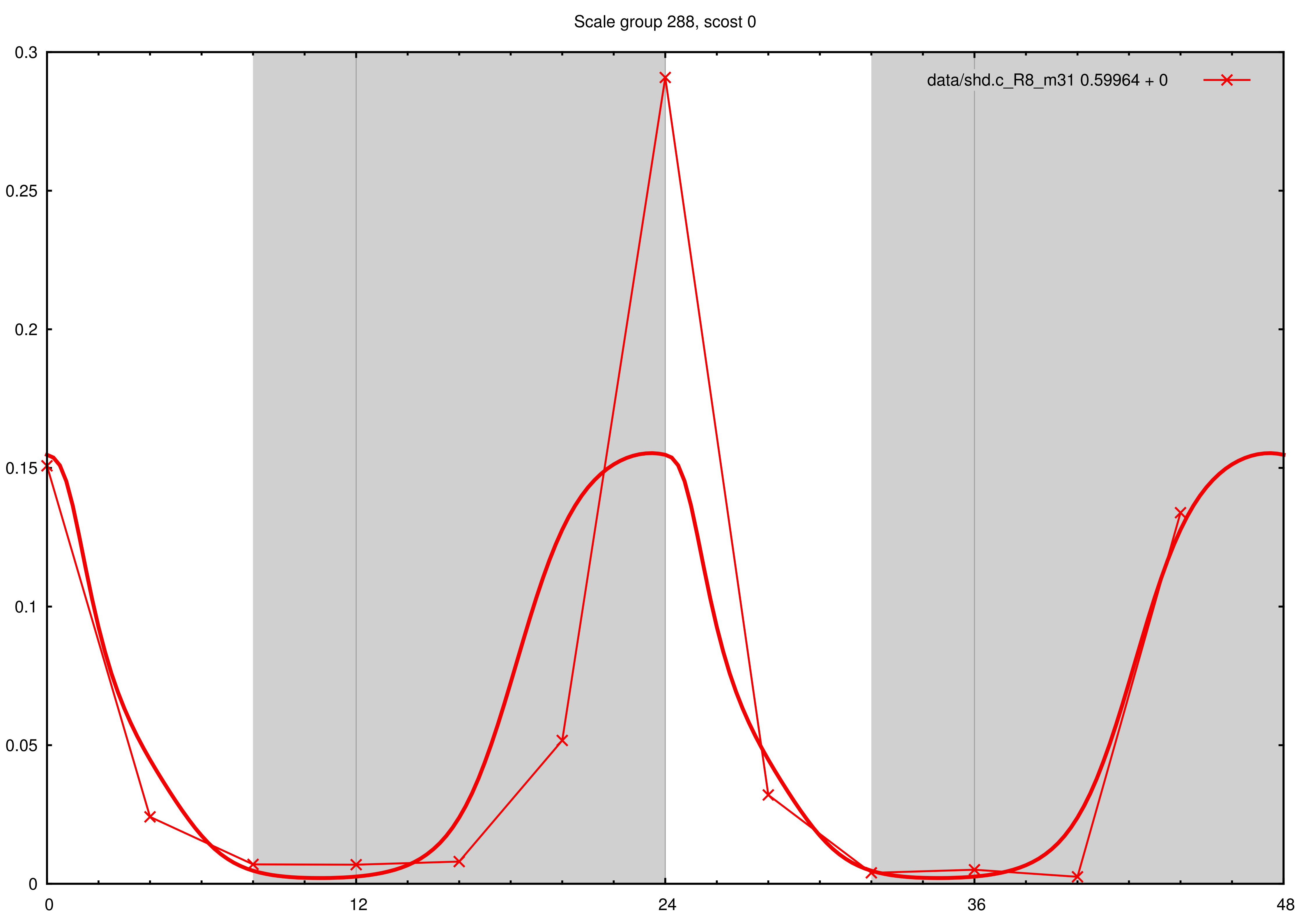
Scale group 288, scost 0
data/shd.c_R8_m31 0.59964 + 0
0.3
0.25
0.2
0.15
0.1
0.05
0
0
12
24
36
48

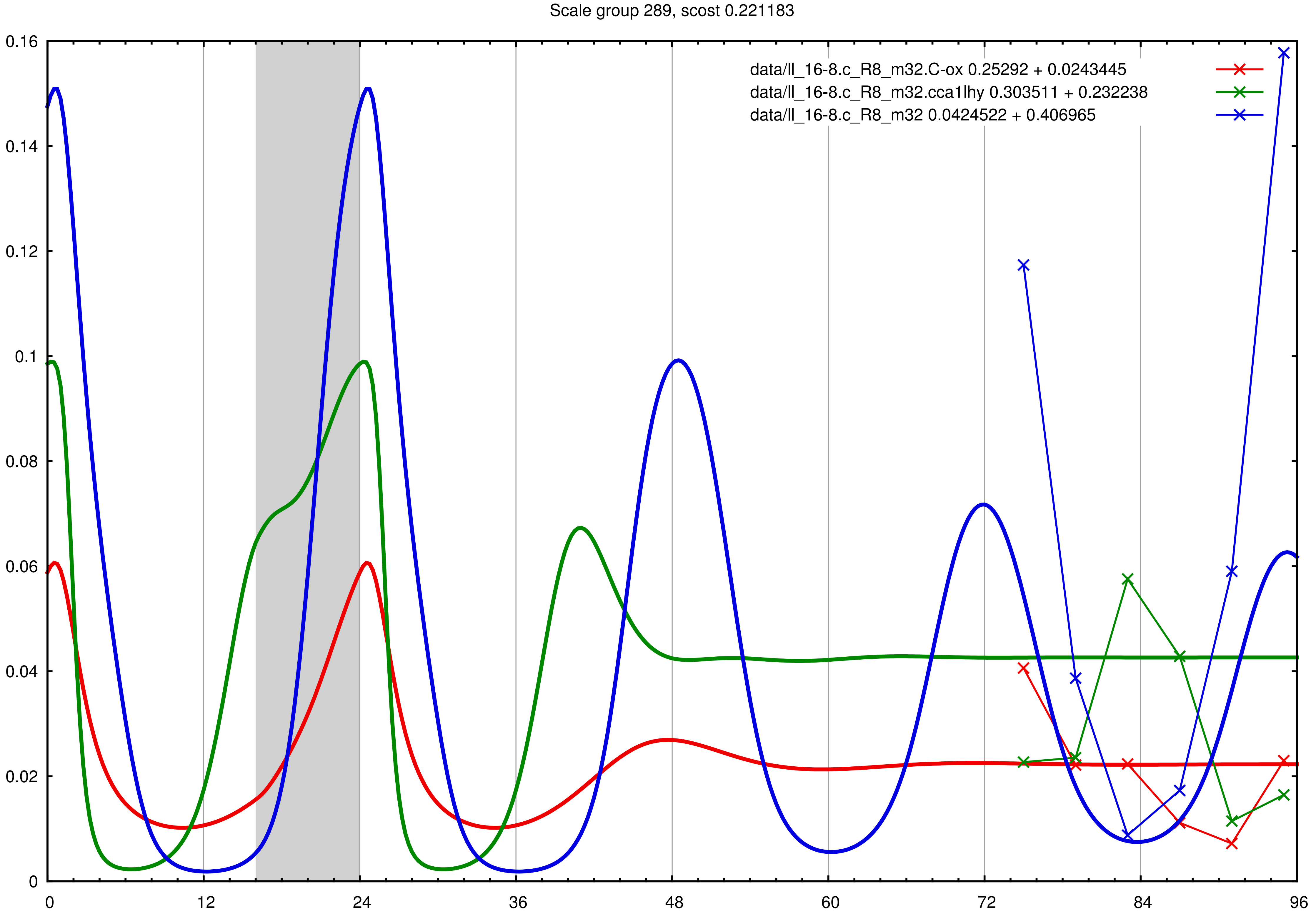

Scale group 289, scost 0.221183
data/ll_16-8.c_R8_m32.C-ox 0.25292 + 0.0243445
data/ll_16-8.c_R8_m32.cca1lhy 0.303511 + 0.232238
data/ll_16-8.c_R8_m32 0.0424522 + 0.406965
0.16
0.14
0.12
0.1
0.08
0.06
0.04
0.02
0
0
12
24
36
48
60
72
84
96

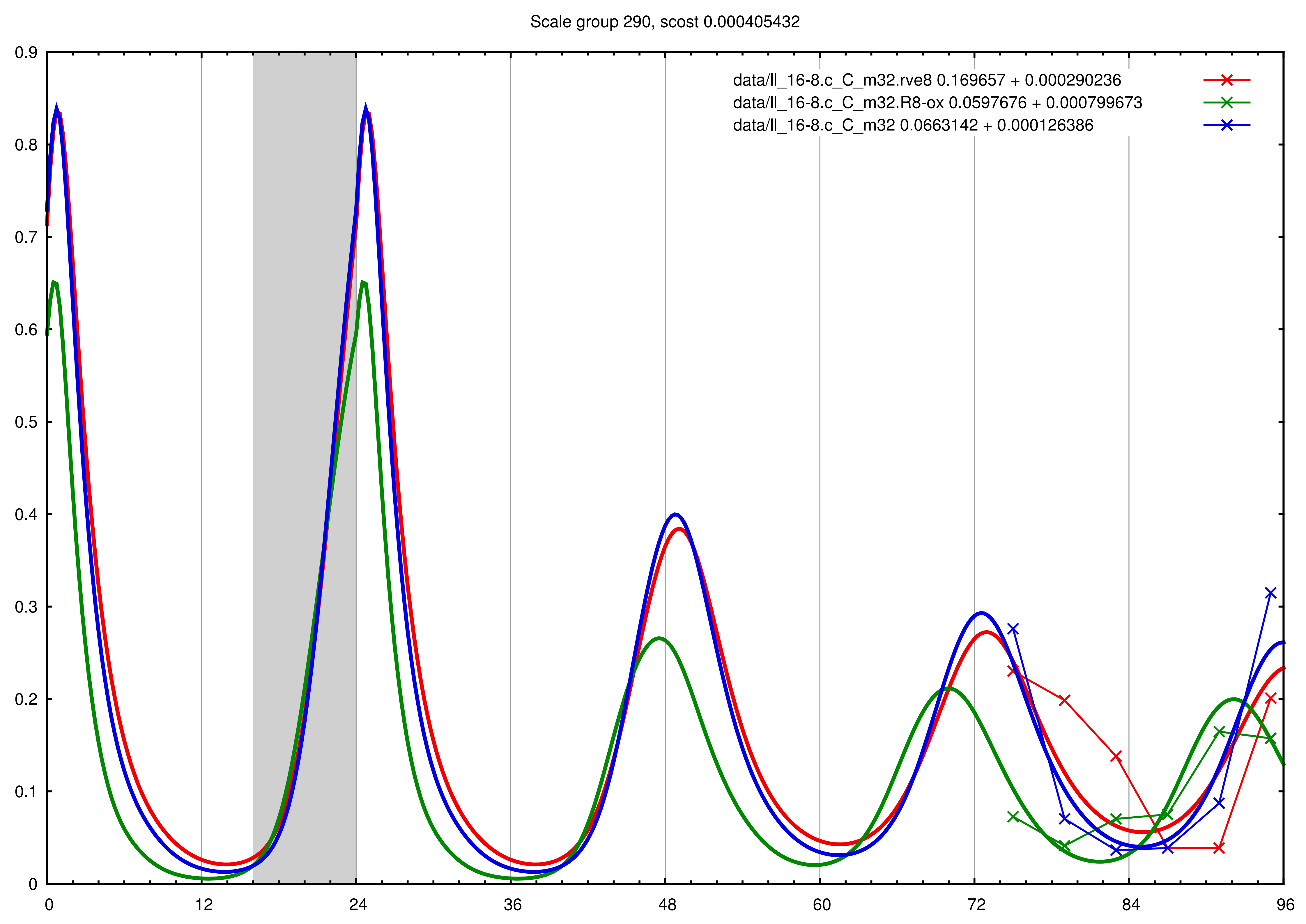

Scale group 290, scost 0.000405432
data/ll_16-8.c_C_m32.rve8 0.169657 + 0.000290236
data/ll_16-8.c_C_m32.R8-ox 0.0597676 + 0.000799673
data/ll_16-8.c_C_m32 0.0663142 + 0.000126386
0.9
0.8
0.7
0.6
0.5
0.4
0.3
0.2
0.1
0
0
12
24
36
48
60
72
84
96

Scale group 291, scost 0.0489845

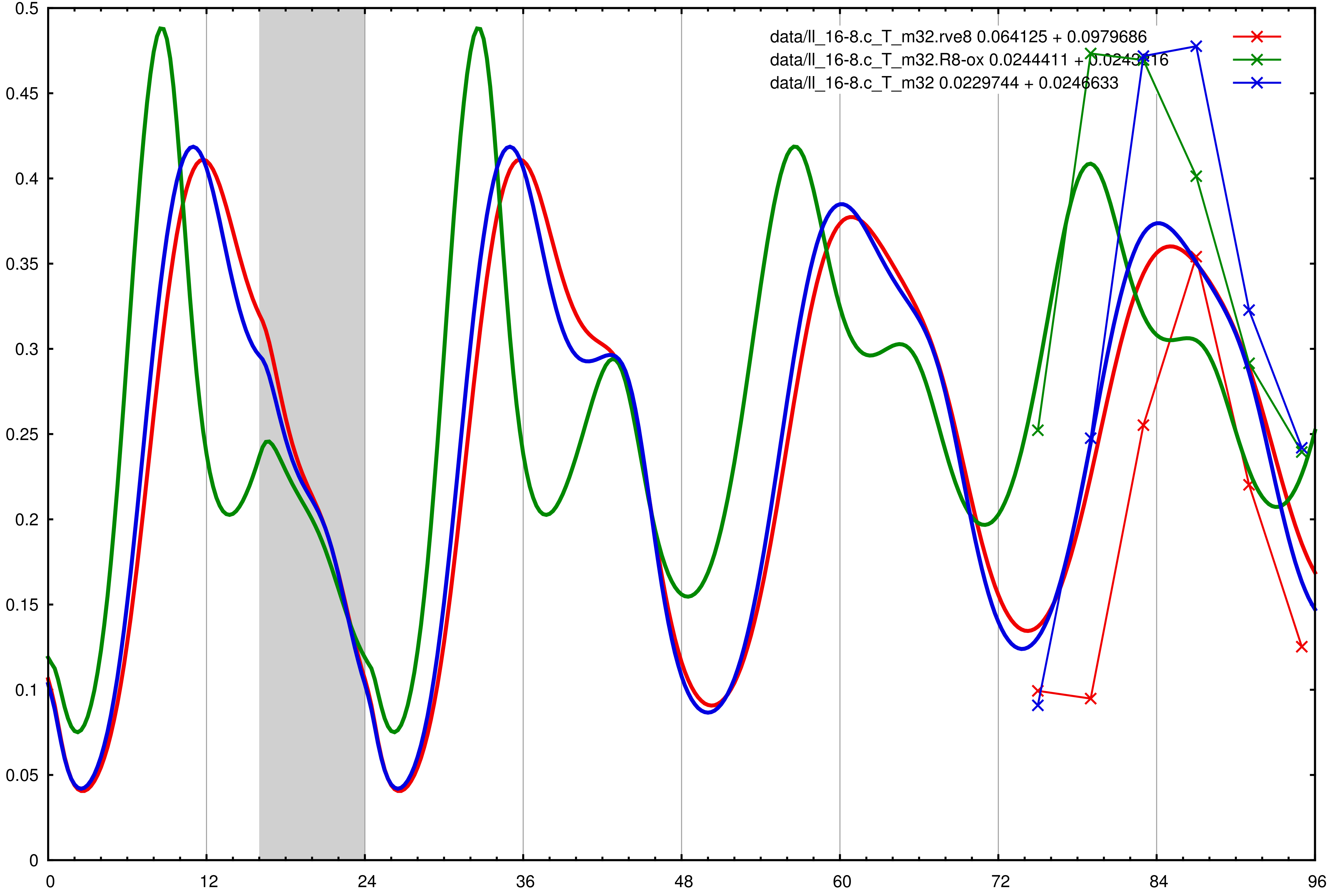

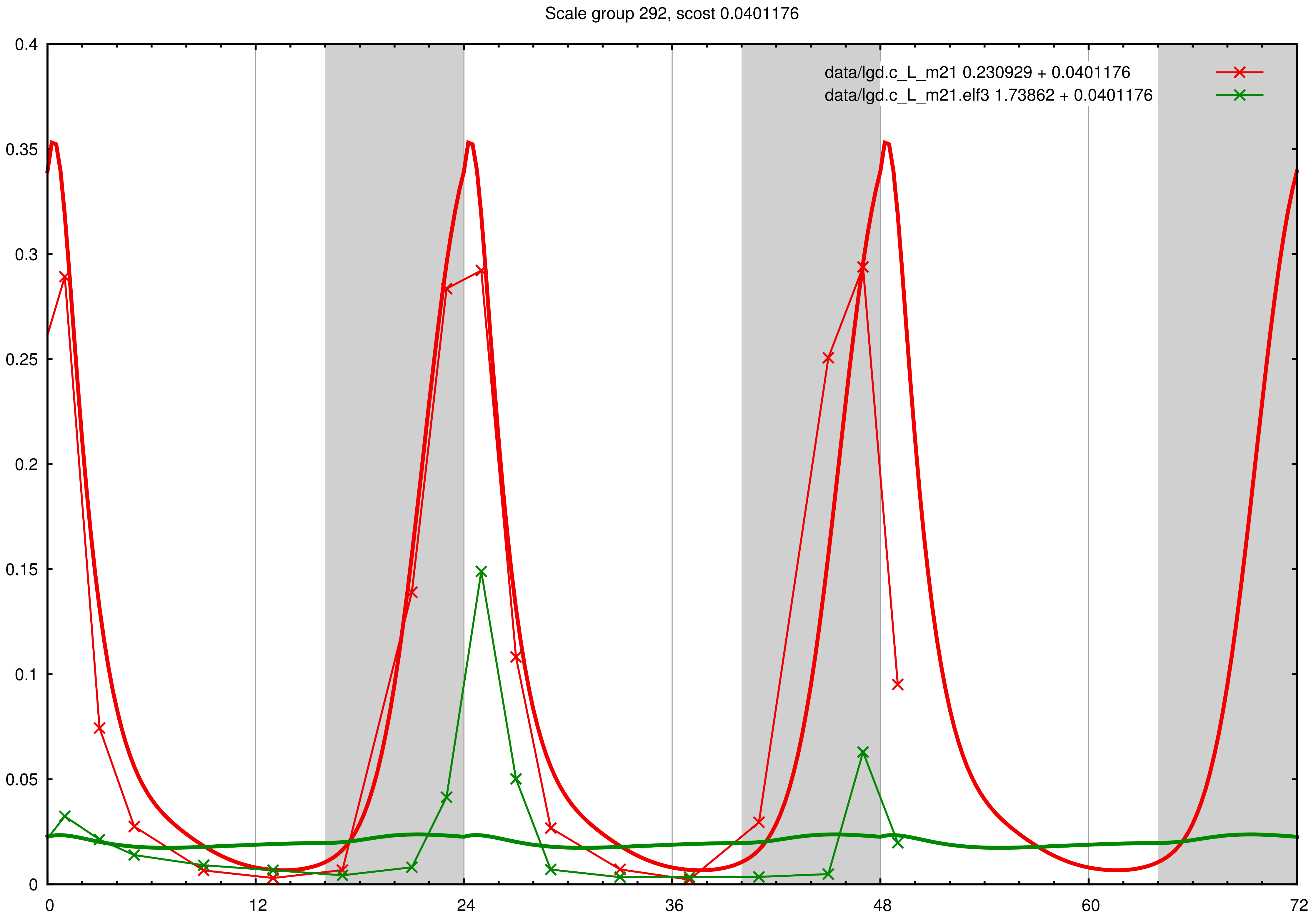

Scale group 292, scost 0.0401176
data/lgd.c_L_m21 0.230929 + 0.0401176
data/lgd.c_L_m21.elf3 1.73862 + 0.0401176
0.4
0.35
0.3
0.25
0.2
0.15
0.1
0.05
0
0
12
24
36
48
60
72

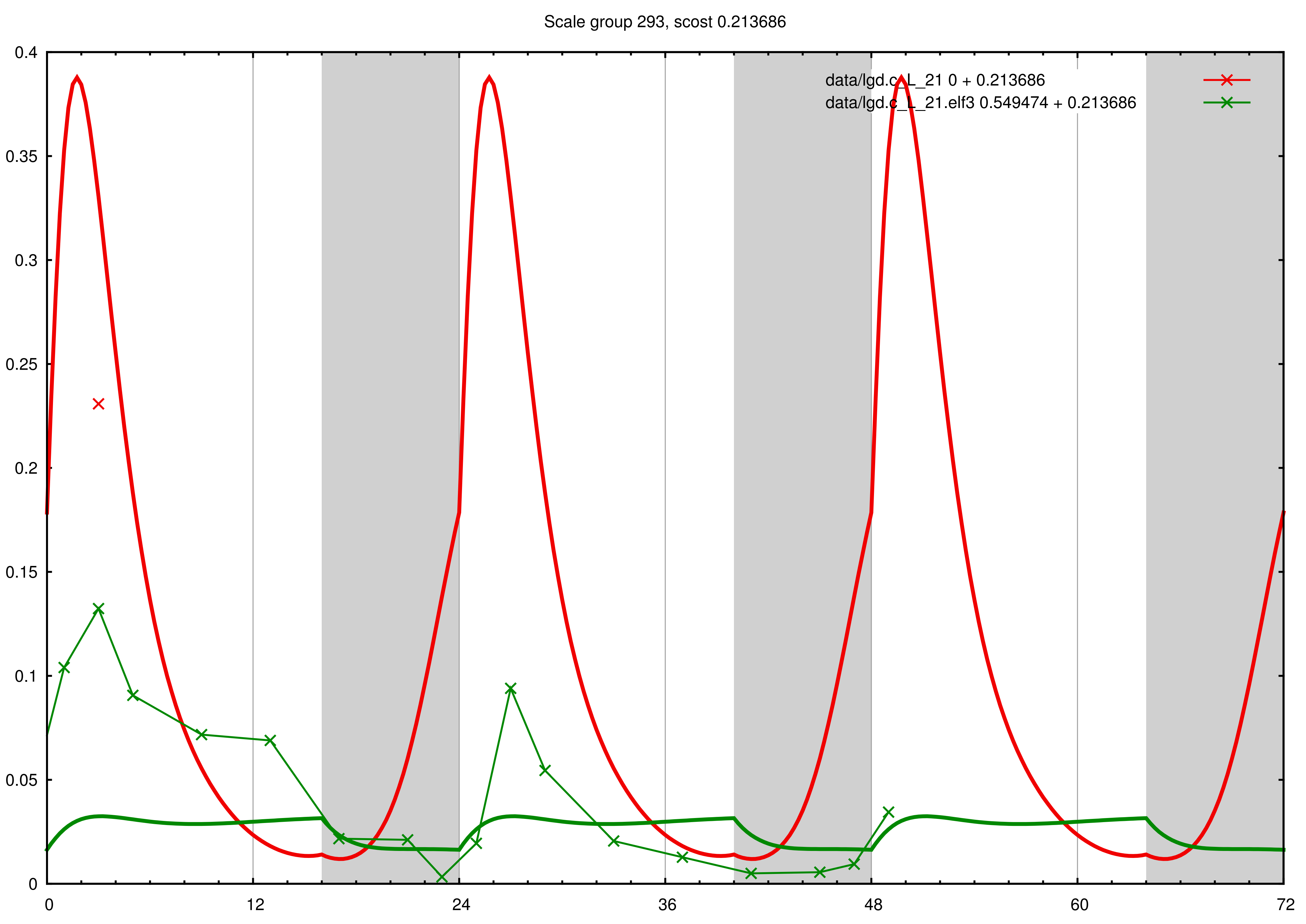

Scale group 293, scost 0.213686
data/lgd.c_L_21 0 + 0.213686
data/lgd.c_L_21.elf3 0.549474 + 0.213686
0.4
0.35
0.3
0.25
0.2
0.15
0.1
0.05
0
0
12
24
36
48
60
72

Scale group 294, scost 0

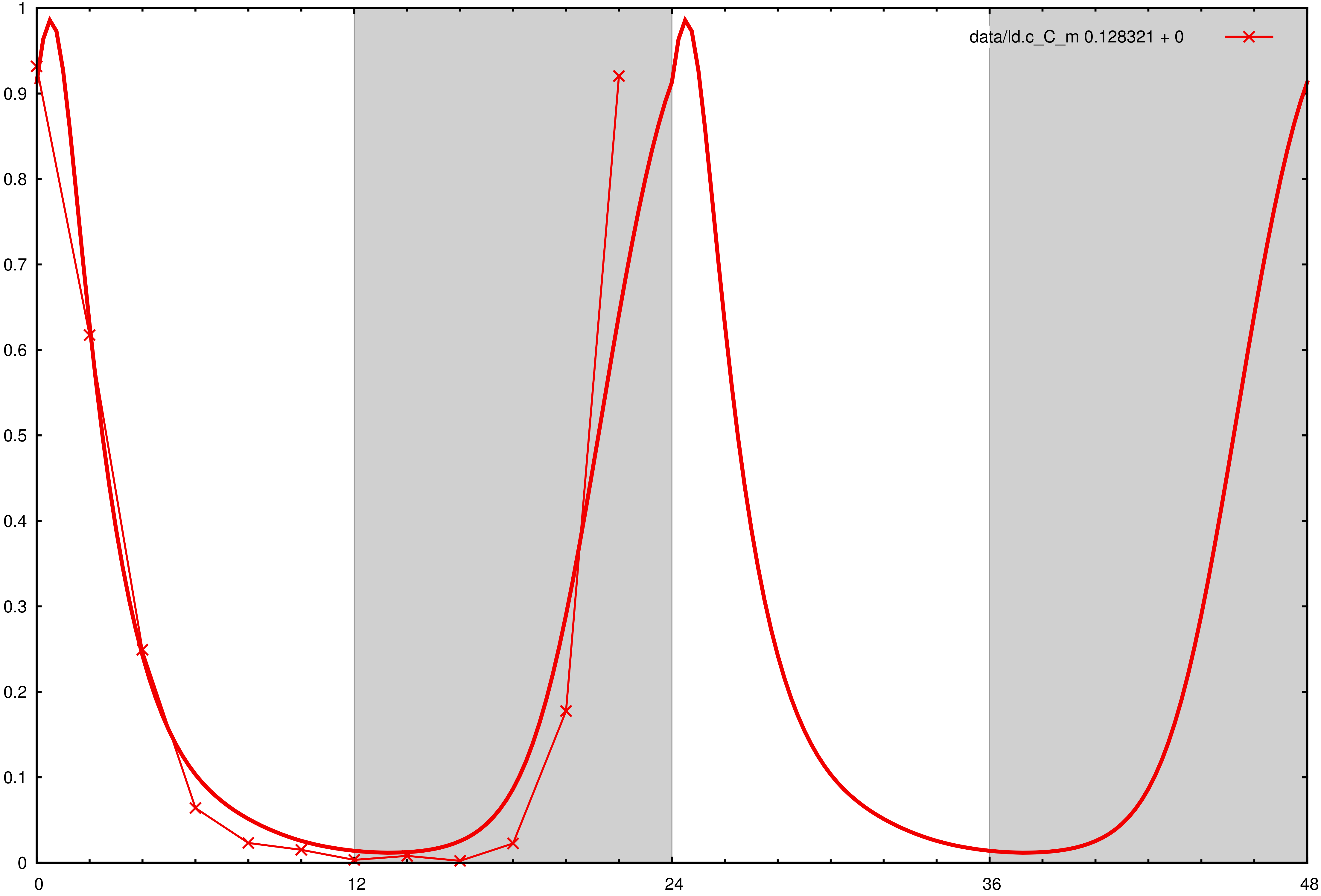

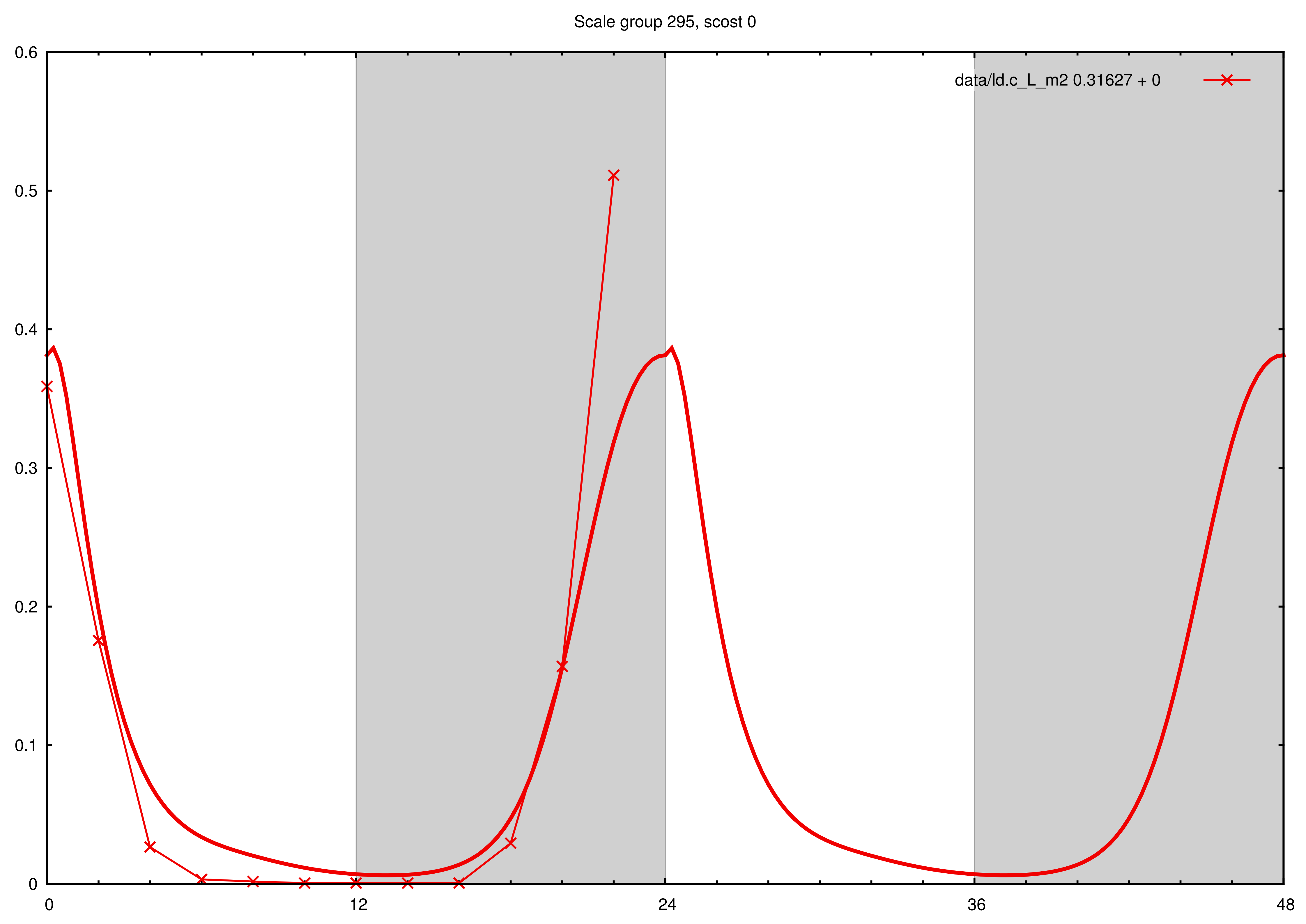
Scale group 295, scost 0
data/ld.c_L_m2 0.31627 + 0
0.6
0.5
0.4
0.3
0.2
0.1
0
0
12
24
36
48

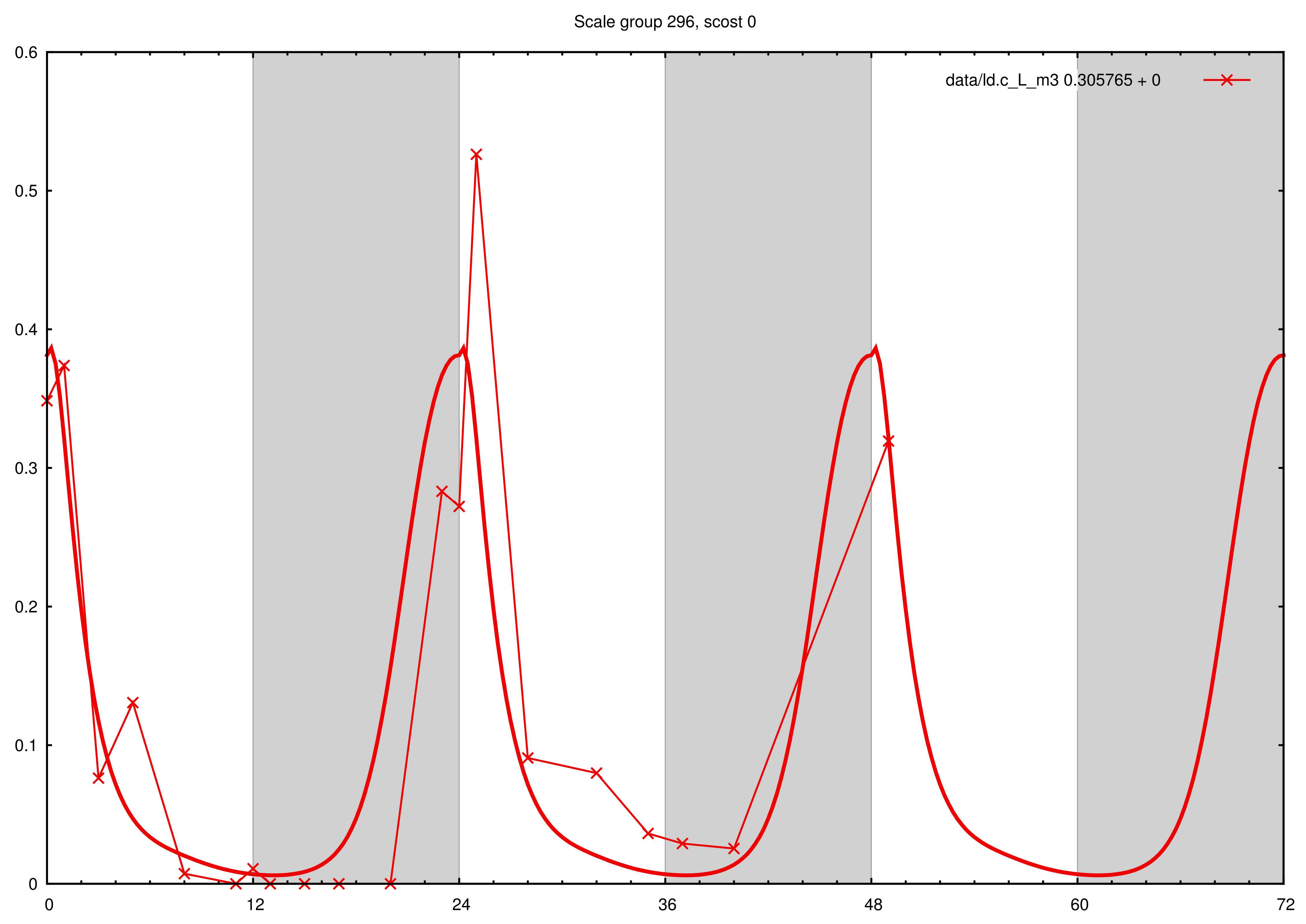

Scale group 296, scost 0
data/ld.c_L_m3 0.305765 + 0
0.6
0.5
0.4
0.3
0.2
0.1
0
0
12
24
36
48
60
72

Scale group 297, scost 0

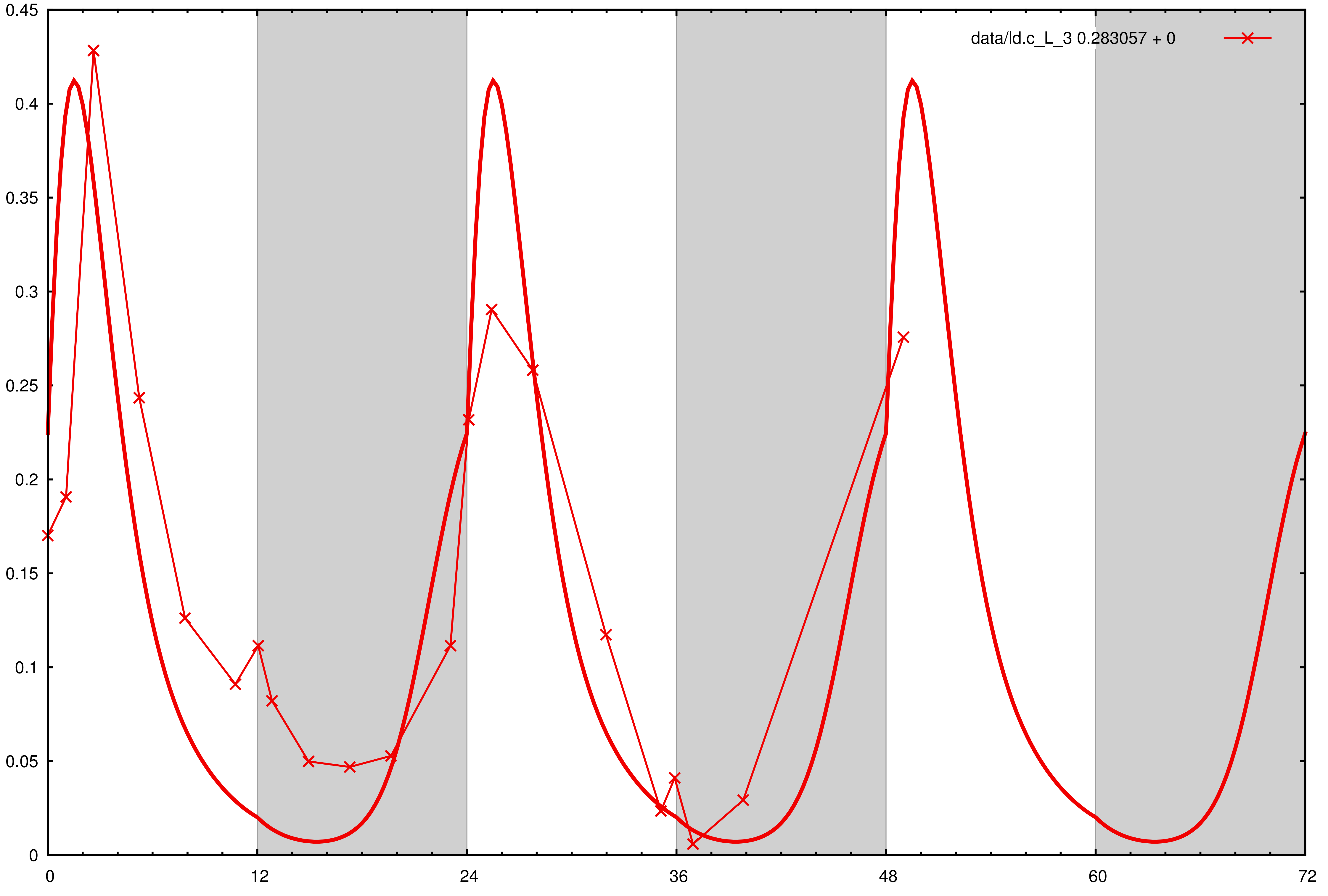

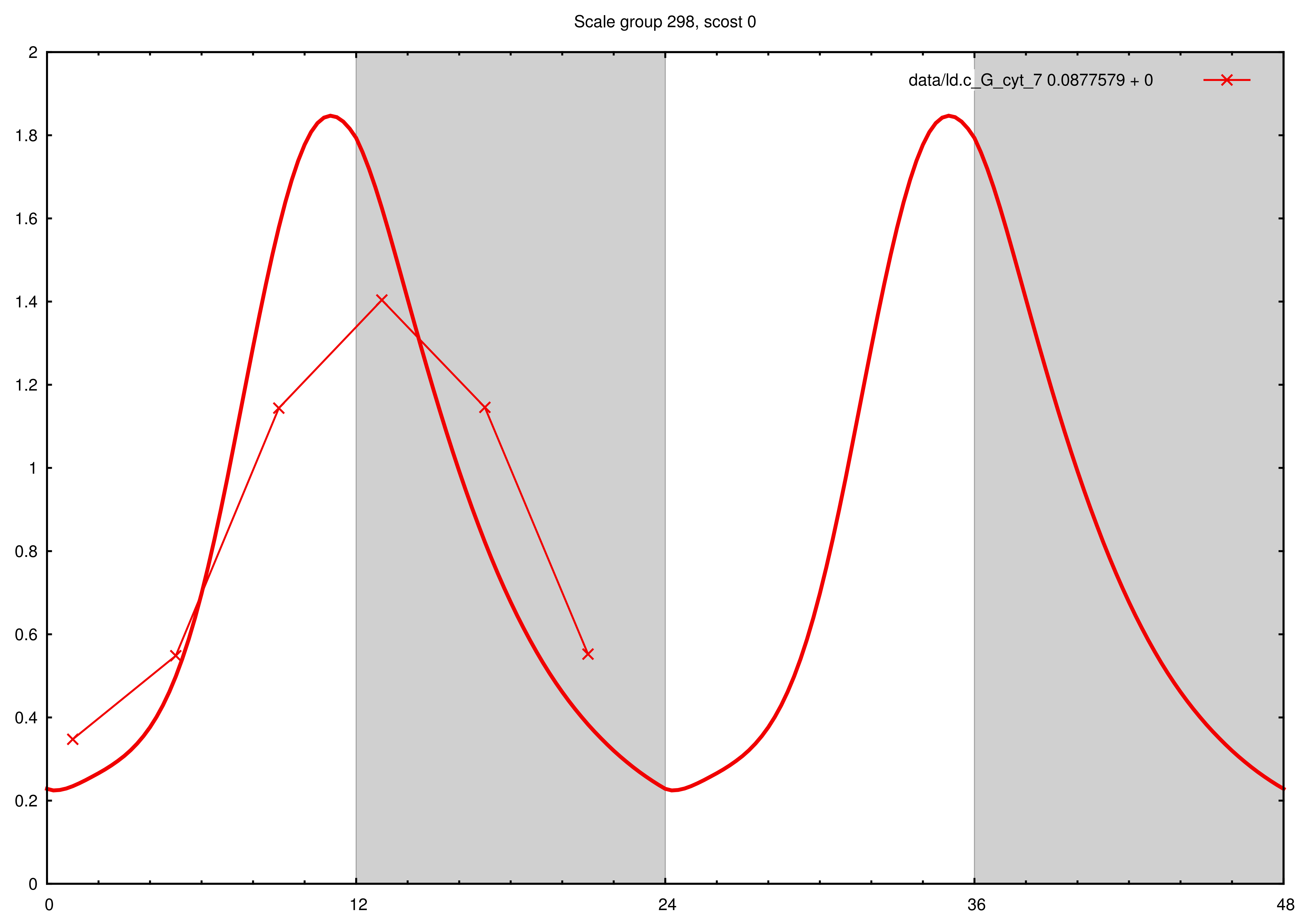

Scale group 298, scost 0
data/ld.c_G_cyt_7 0.0877579 + 0
2
1.8
1.6
1.4
1.2
1
0.8
0.6
0.4
0.2
0
0
12
24
36
48

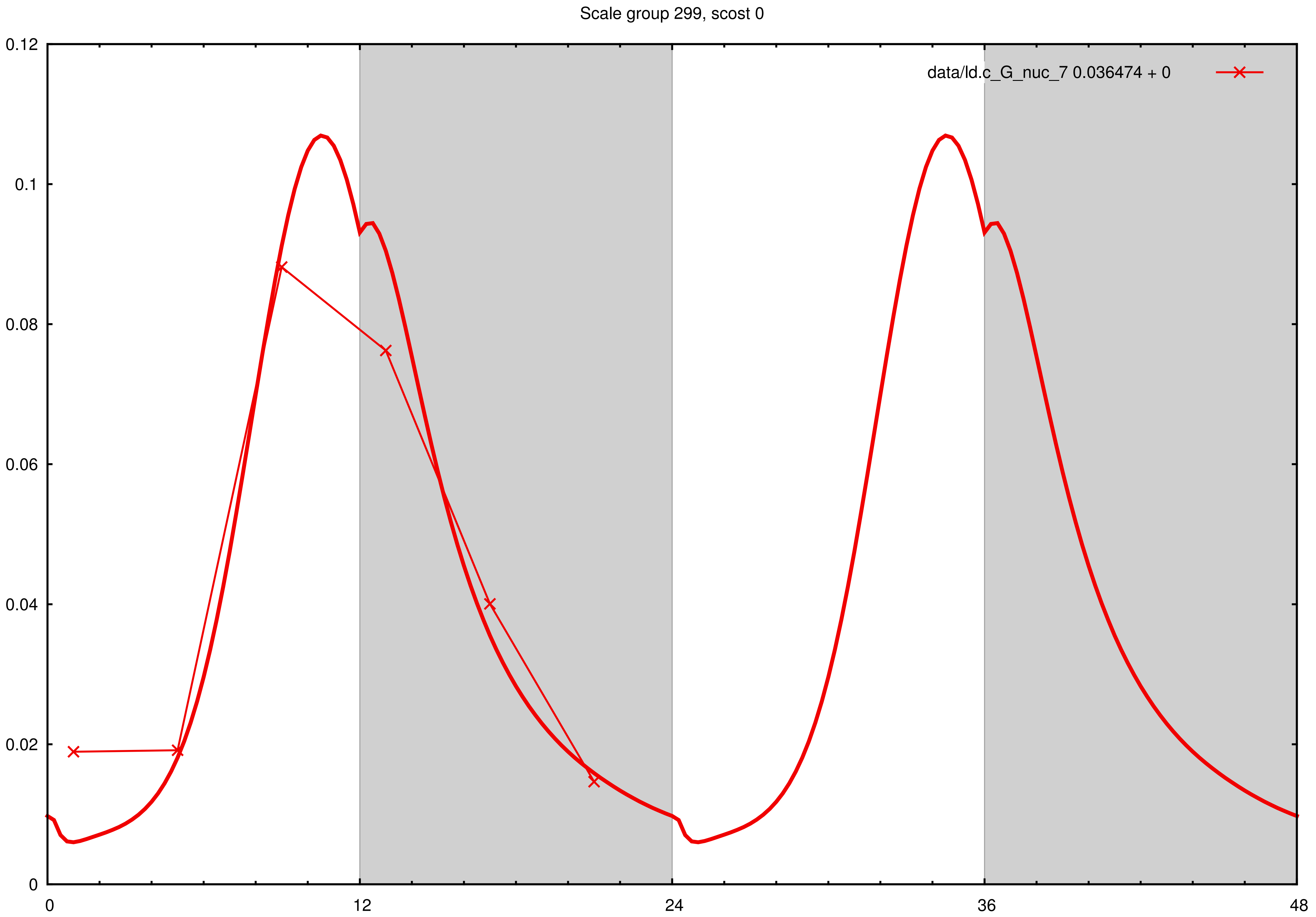

Scale group 299, scost 0
data/ld.c_G_nuc_7 0.036474 + 0
0.12
0.1
0.08
0.06
0.04
0.02
0
0
12
24
36
48

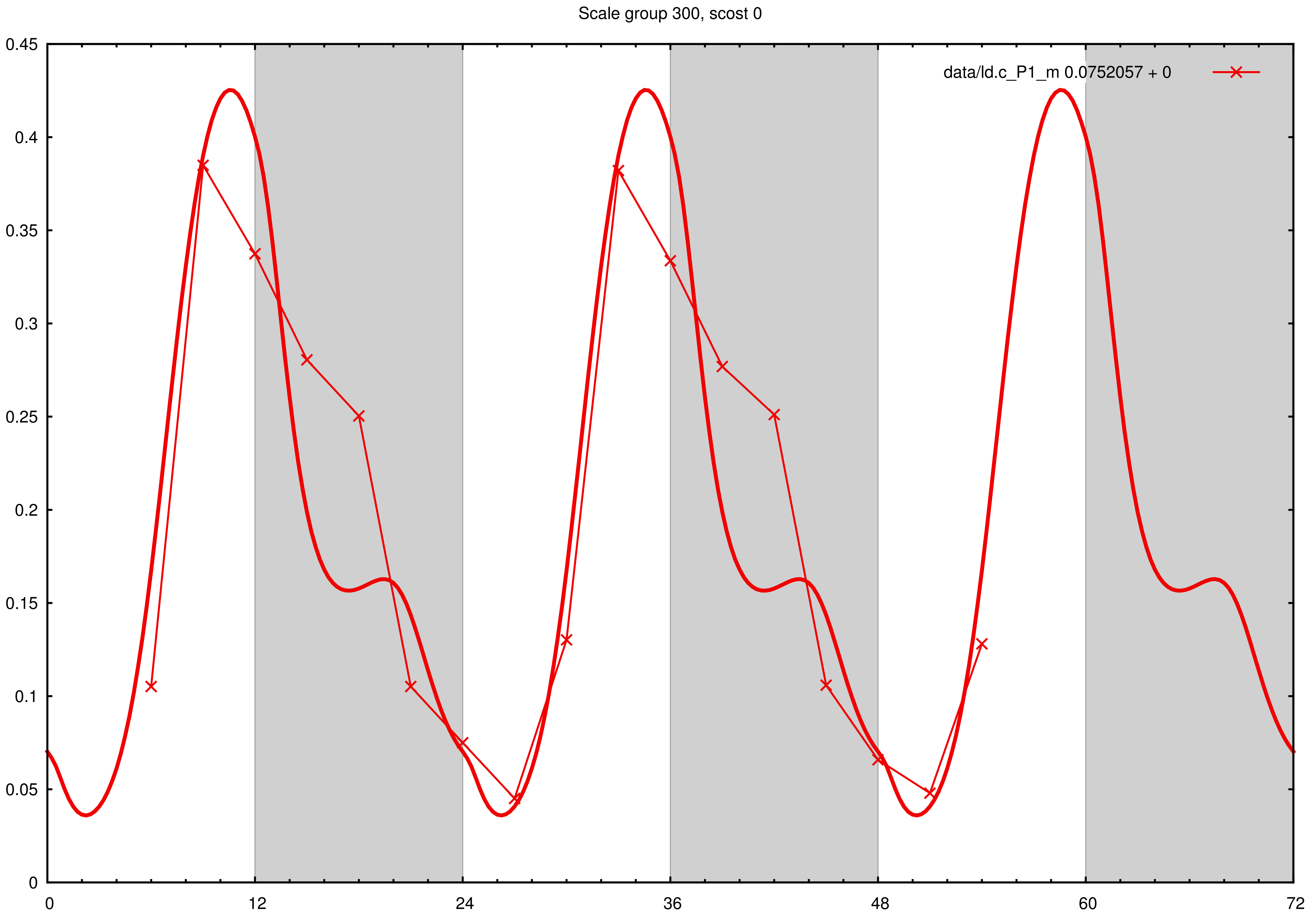
Scale group 300, scost 0
data/ld.c_P1_m 0.0752057 + 0
0
0.05
0.1
0.15
0.2
0.25
0.3
0.35
0.4
0.45
0
12
24
36
48
60
72

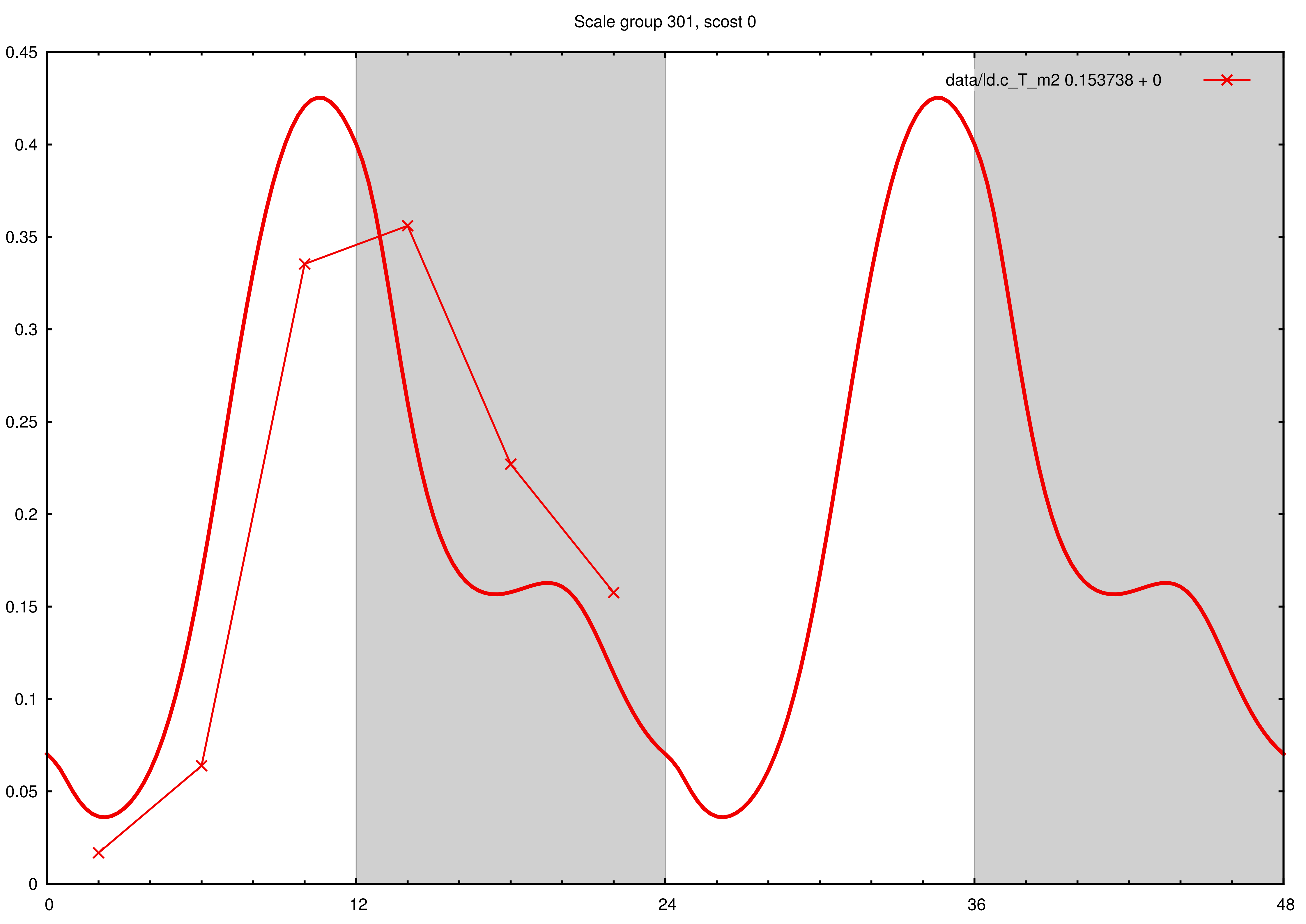

Scale group 301, scost 0
data/ld.c_T_m2 0.153738 + 0
0.45
0.4
0.35
0.3
0.25
0.2
0.15
0.1
0.05
0
0
12
24
36
48

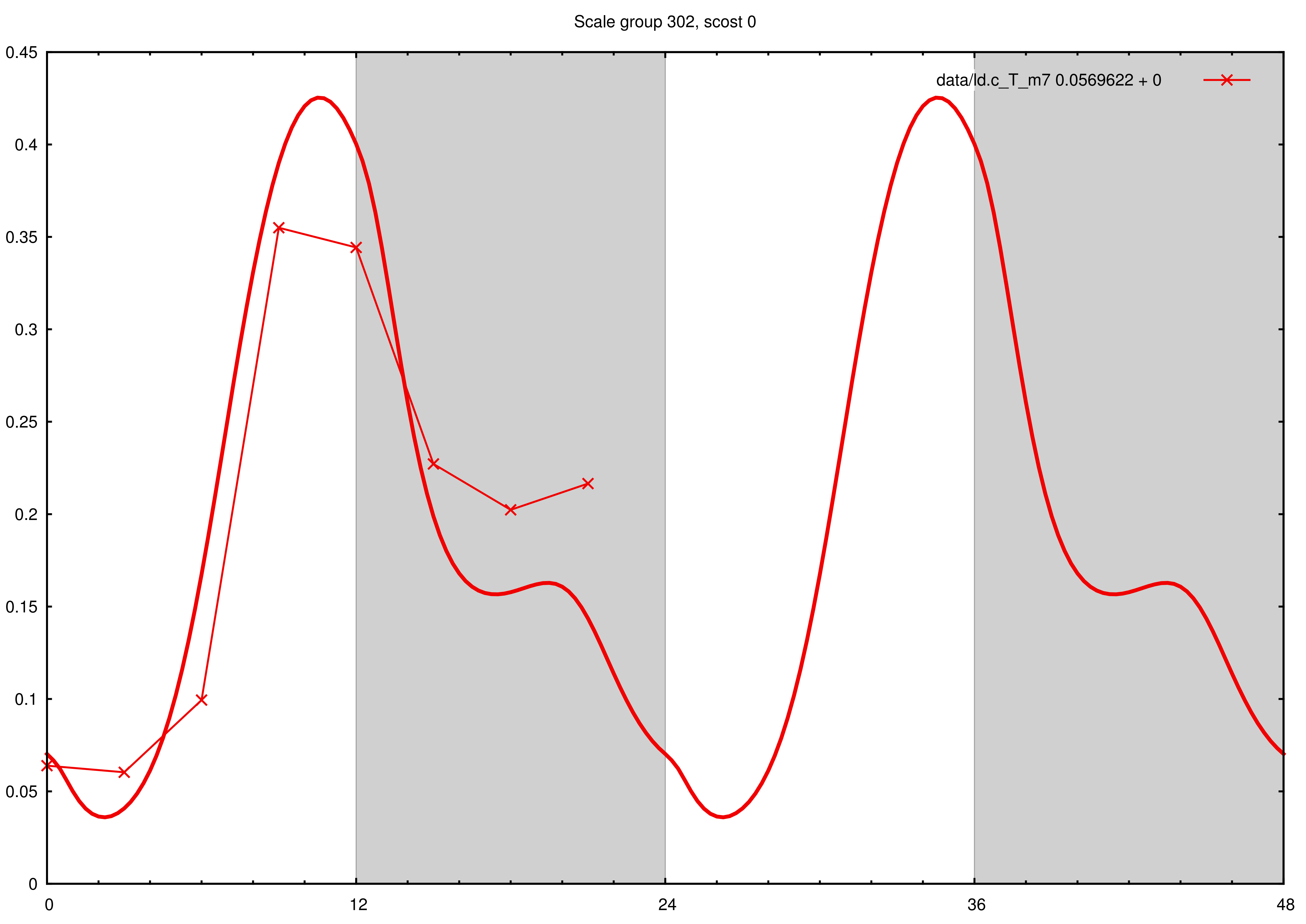
Scale group 302, scost 0
data/ld.c_T_m7 0.0569622 + 0
0.45
0.4
0.35
0.3
0.25
0.2
0.15
0.1
0.05
0
0
12
24
36
48

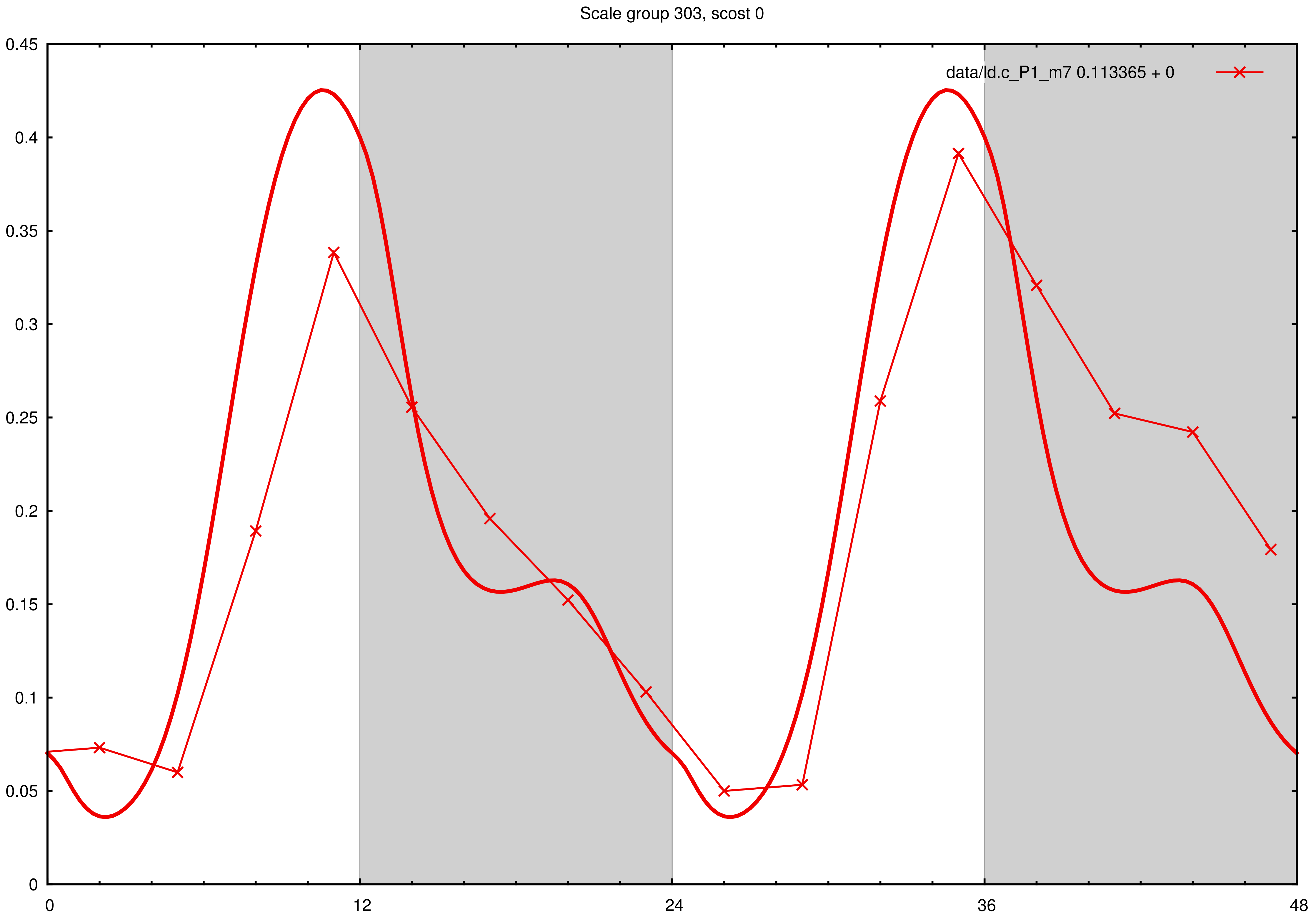
Scale group 303, scost 0
data/ld.c_P1_m7 0.113365 + 0
0.45
0.4
0.35
0.3
0.25
0.2
0.15
0.1
0.05
0
0
12
24
36
48

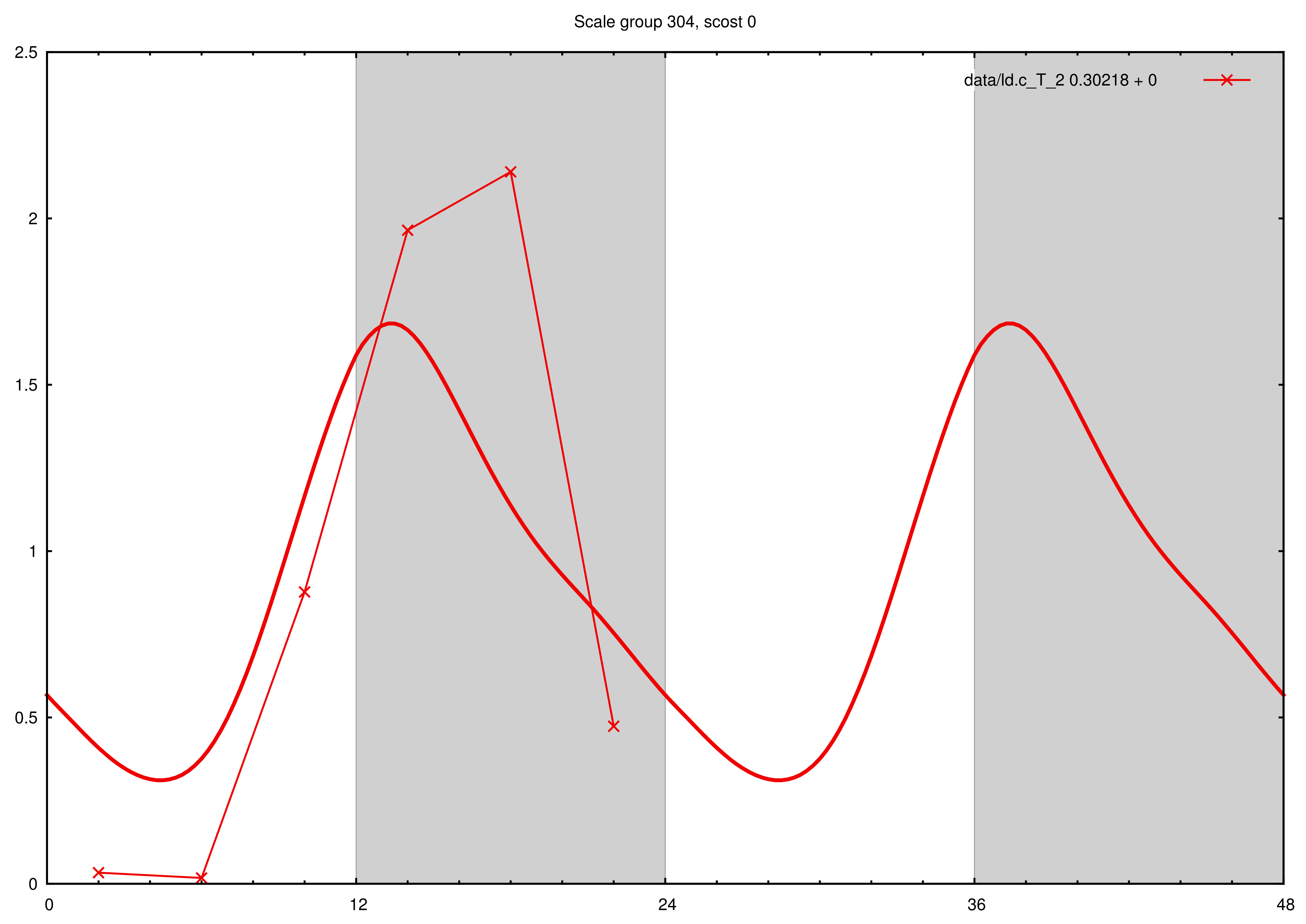
Scale group 304, scost 0
data/ld.c_T_2 0.30218 + 0
2.5
2
1.5
1
0.5
0
0
12
24
36
48

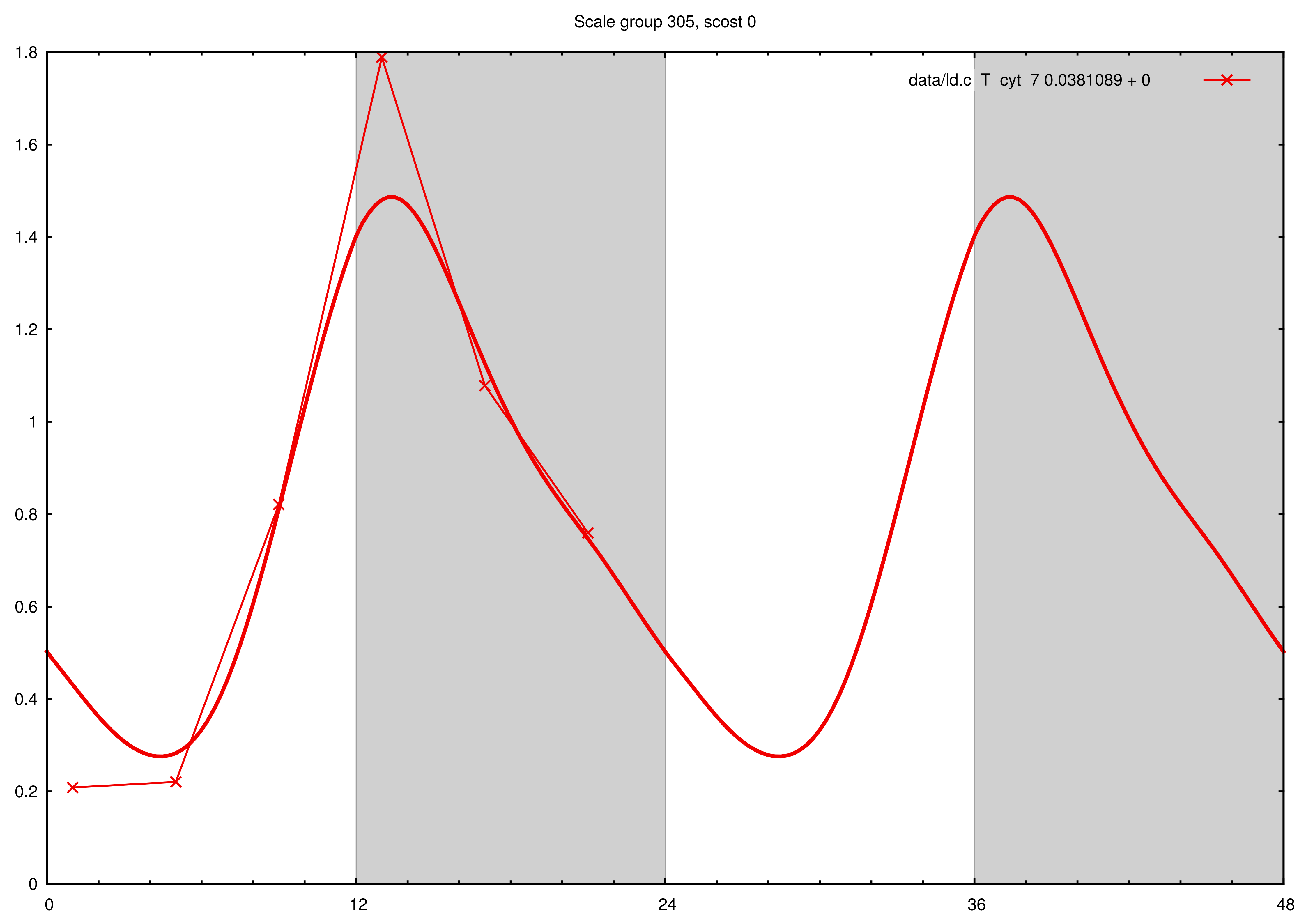

Scale group 305, scost 0
data/ld.c_T_cyt_7 0.0381089 + 0
1.8
1.6
1.4
1.2
1
0.8
0.6
0.4
0.2
0
0
12
24
36
48

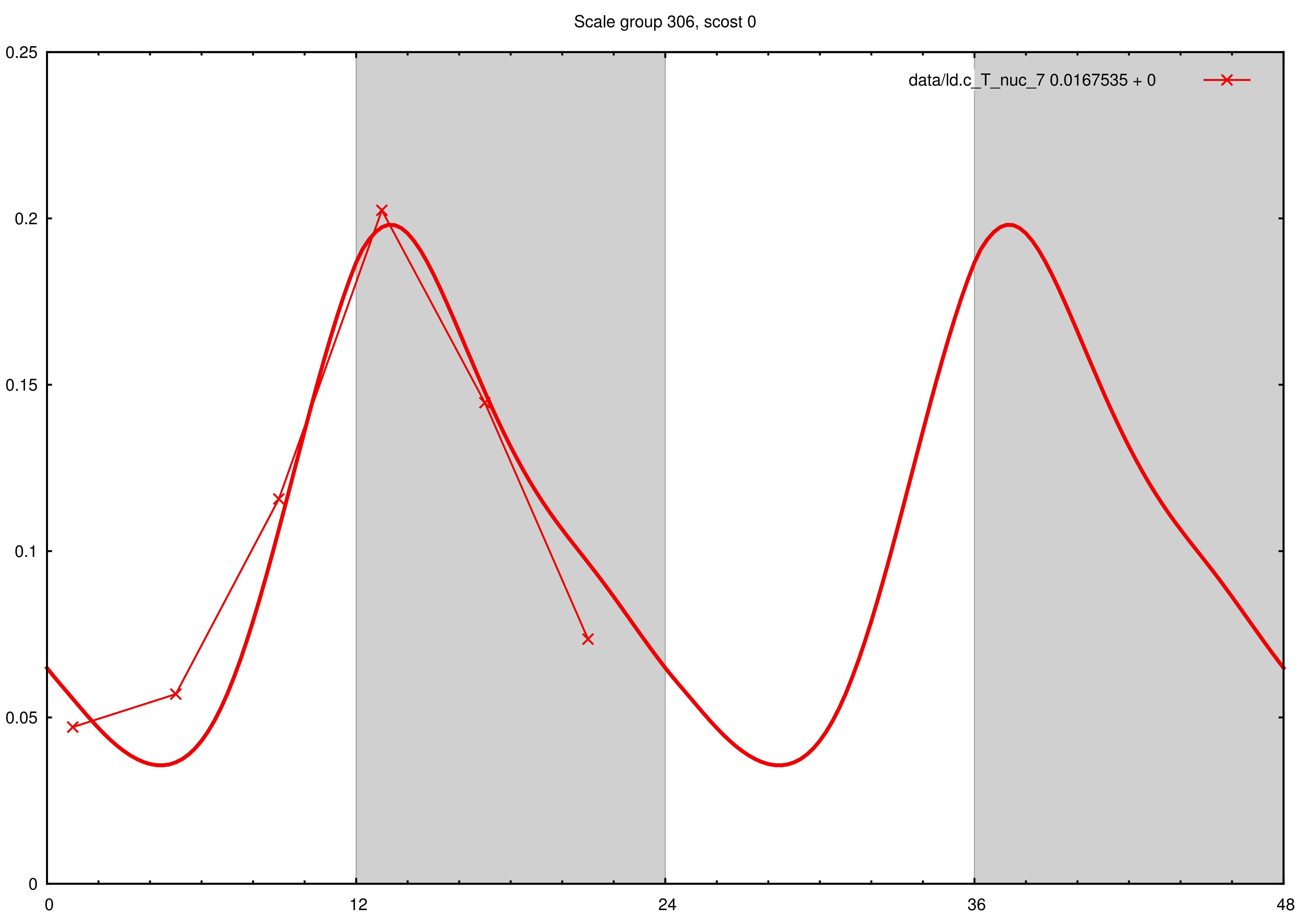
Scale group 306, scost 0
data/ld.c_T_nuc_7 0.0167535 + 0
0.25
0.2
0.15
0.1
0.05
0
0
12
24
36
48

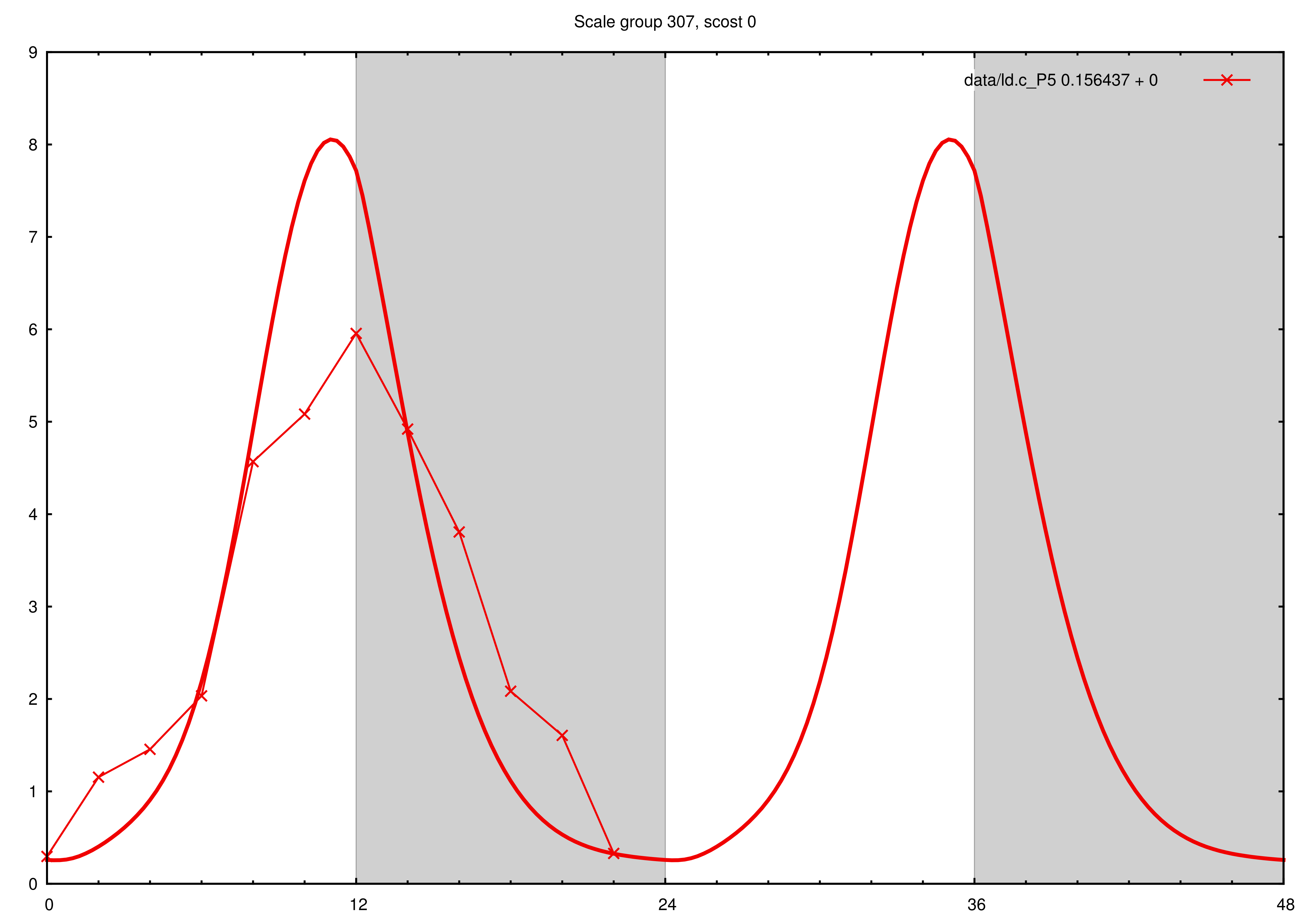
Scale group 307, scost 0
data/ld.c_P5 0.156437 + 0
0
1
2
3
4
5
6
7
8
9
0
12
24
36
48

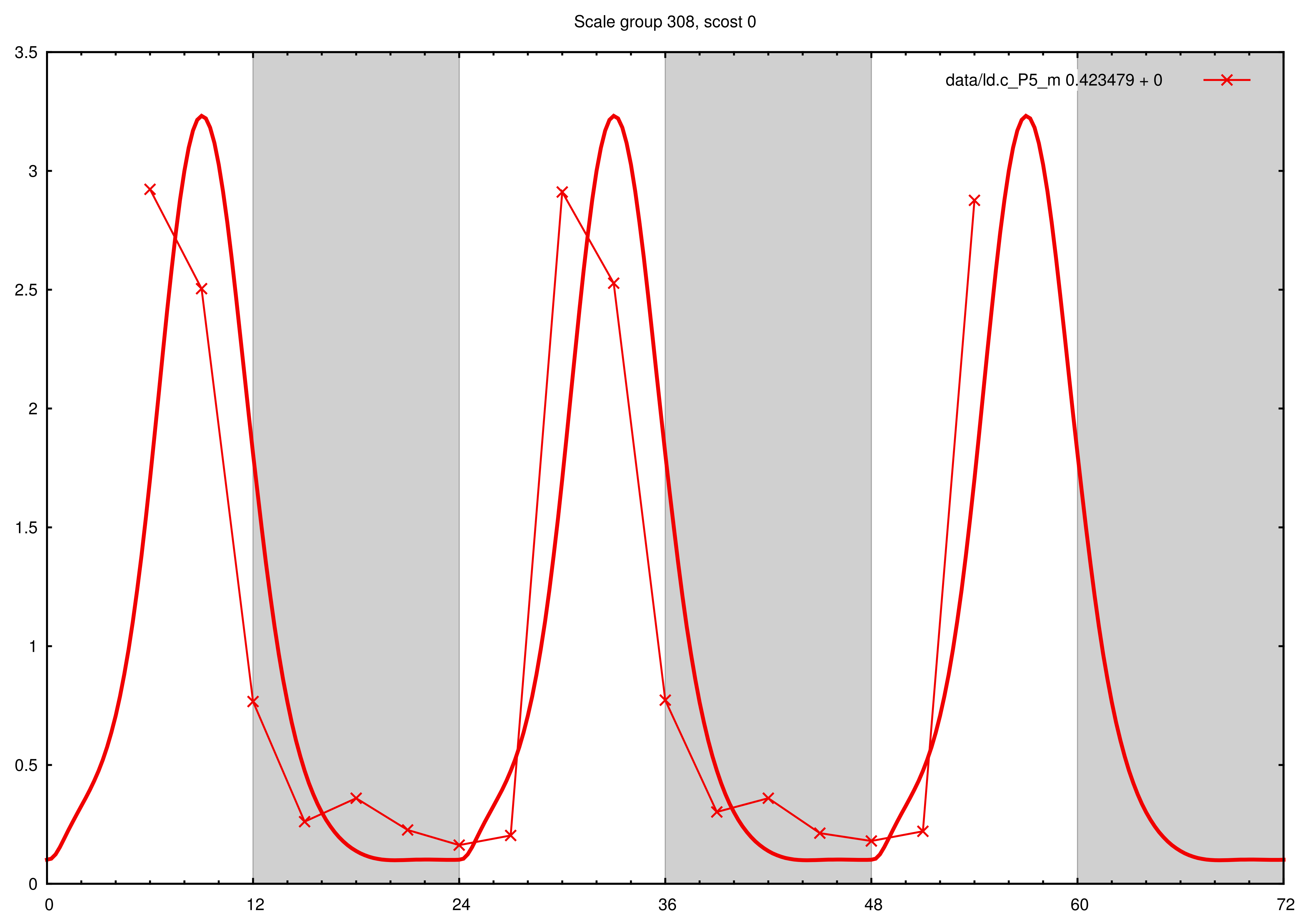

Scale group 308, scost 0
data/ld.c_P5_m 0.423479 + 0
3.5
3
2.5
2
1.5
1
0.5
0
0
12
24
36
48
60
72

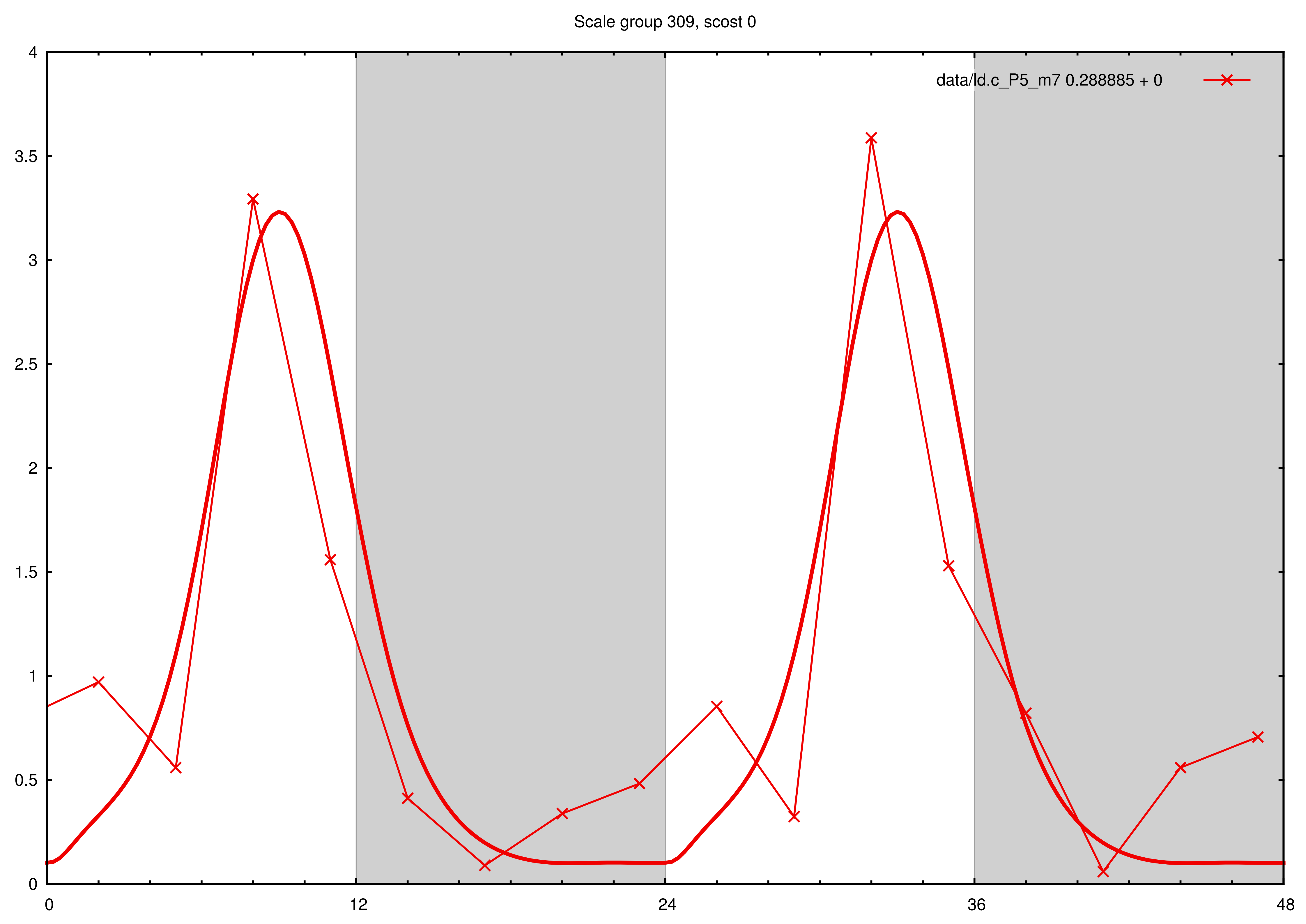

Scale group 309, scost 0
data/ld.c_P5_m7 0.288885 + 0
4
3.5
3
2.5
2
1.5
1
0.5
0
0
12
24
36
48

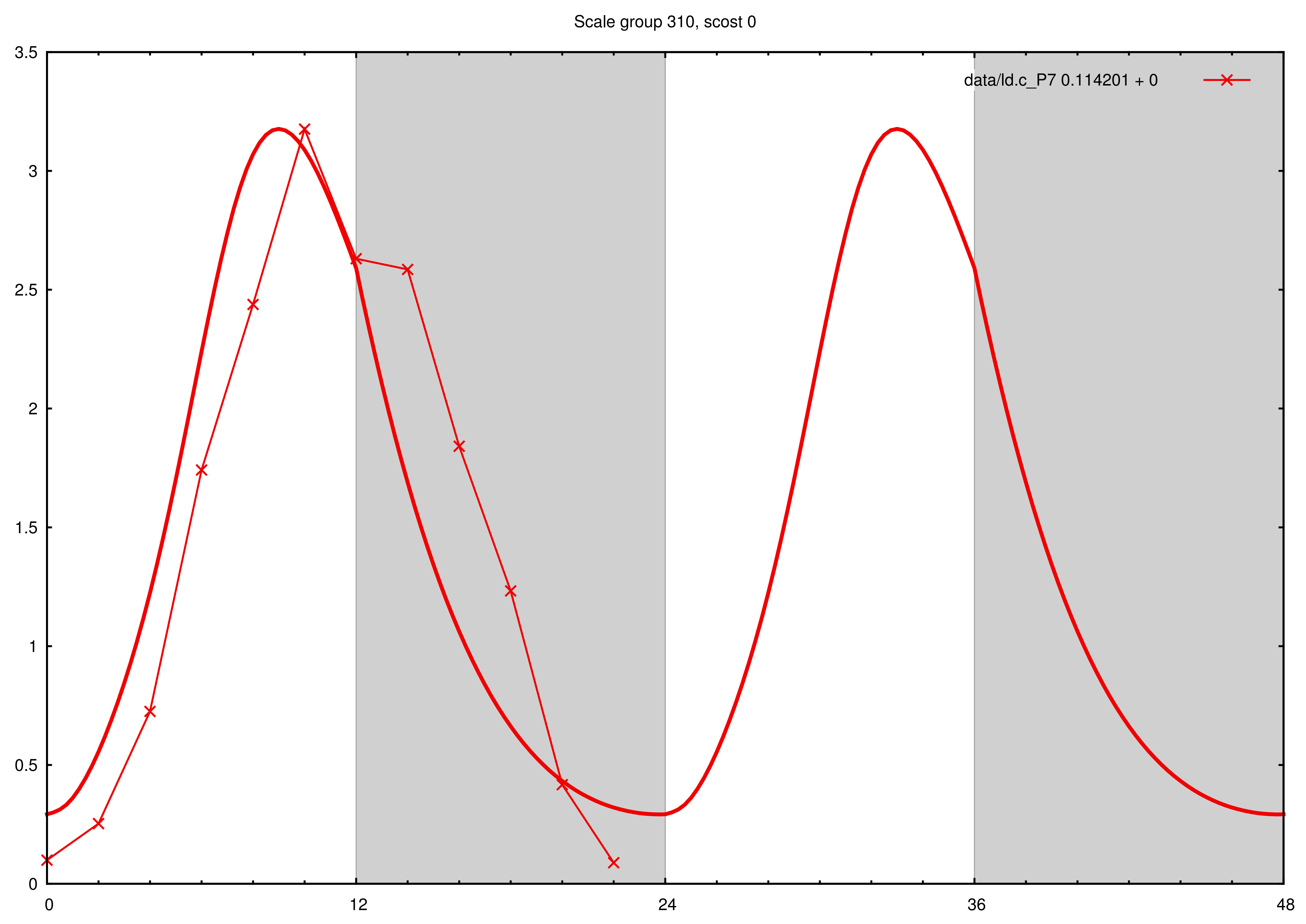
Scale group 310, scost 0
data/ld.c_P7 0.114201 + 0
3.5
3
2.5
2
1.5
1
0.5
0
0
12
24
36
48

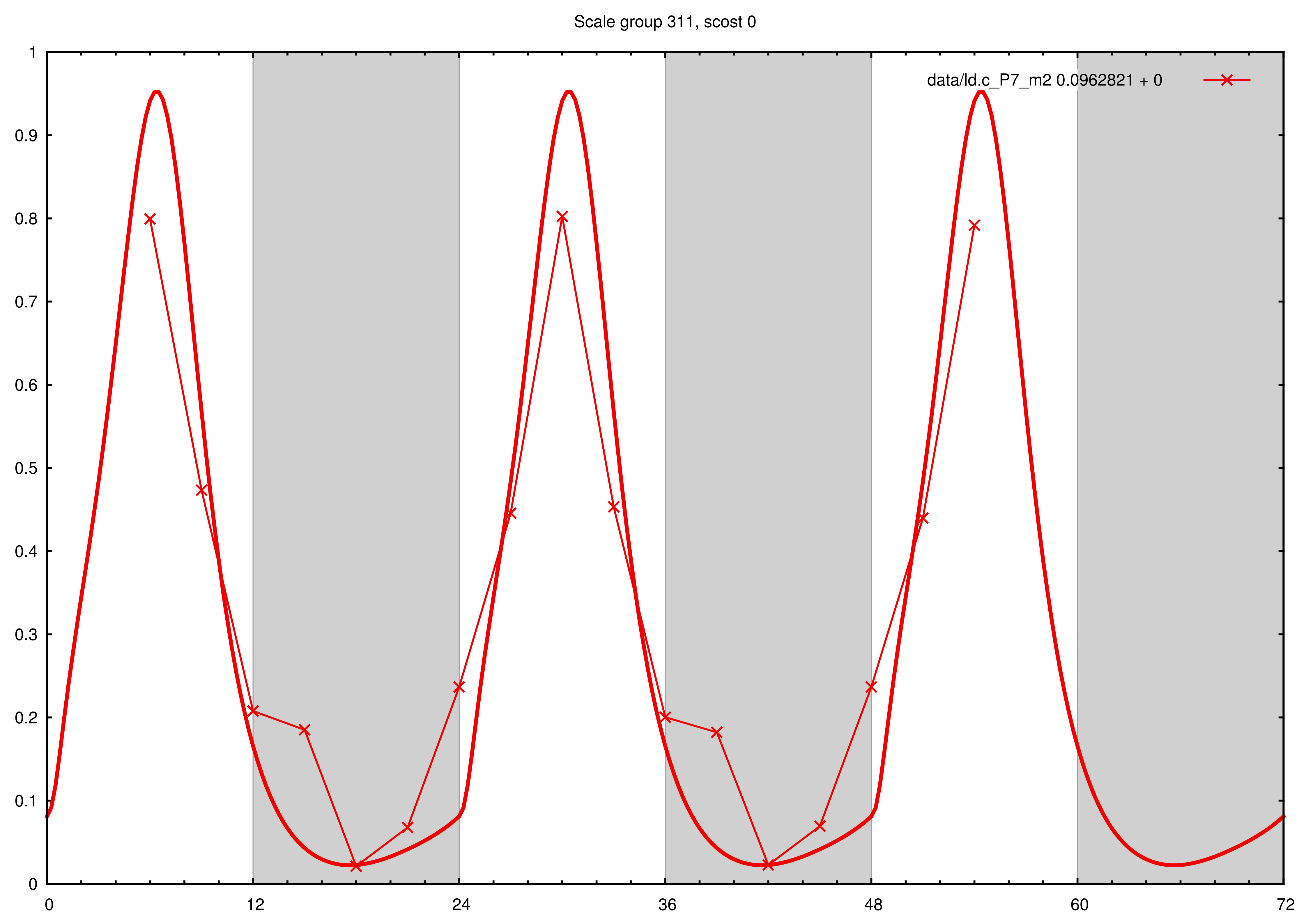

Scale group 311, scost 0
data/ld.c_P7_m2 0.0962821 + 0
0
0.1
0.2
0.3
0.4
0.5
0.6
0.7
0.8
0.9
1
0
12
24
36
48
60
72

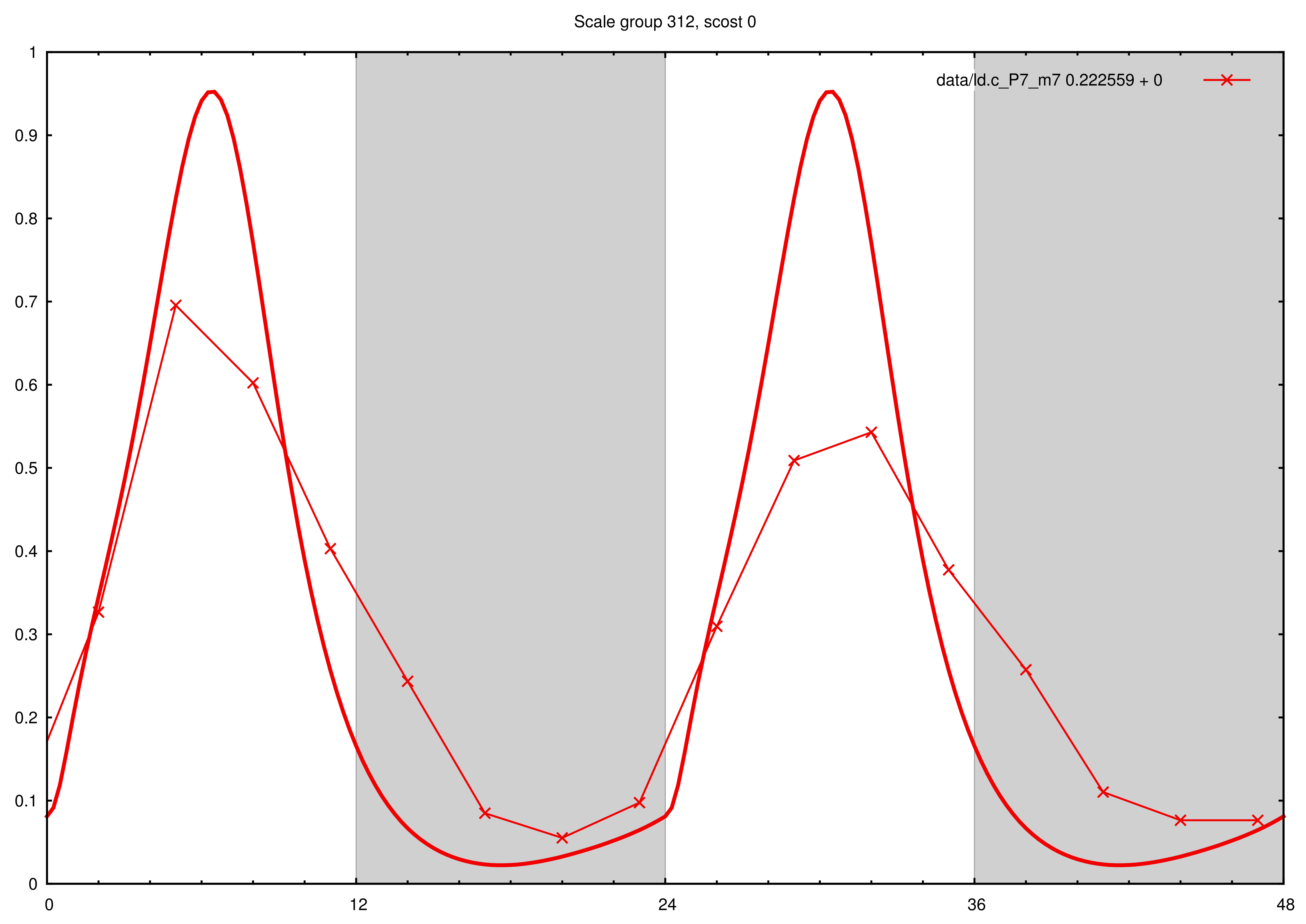

Scale group 312, scost 0
data/ld.c_P7_m7 0.222559 + 0
1
0.9
0.8
0.7
0.6
0.5
0.4
0.3
0.2
0.1
0
0
12
24
36
48

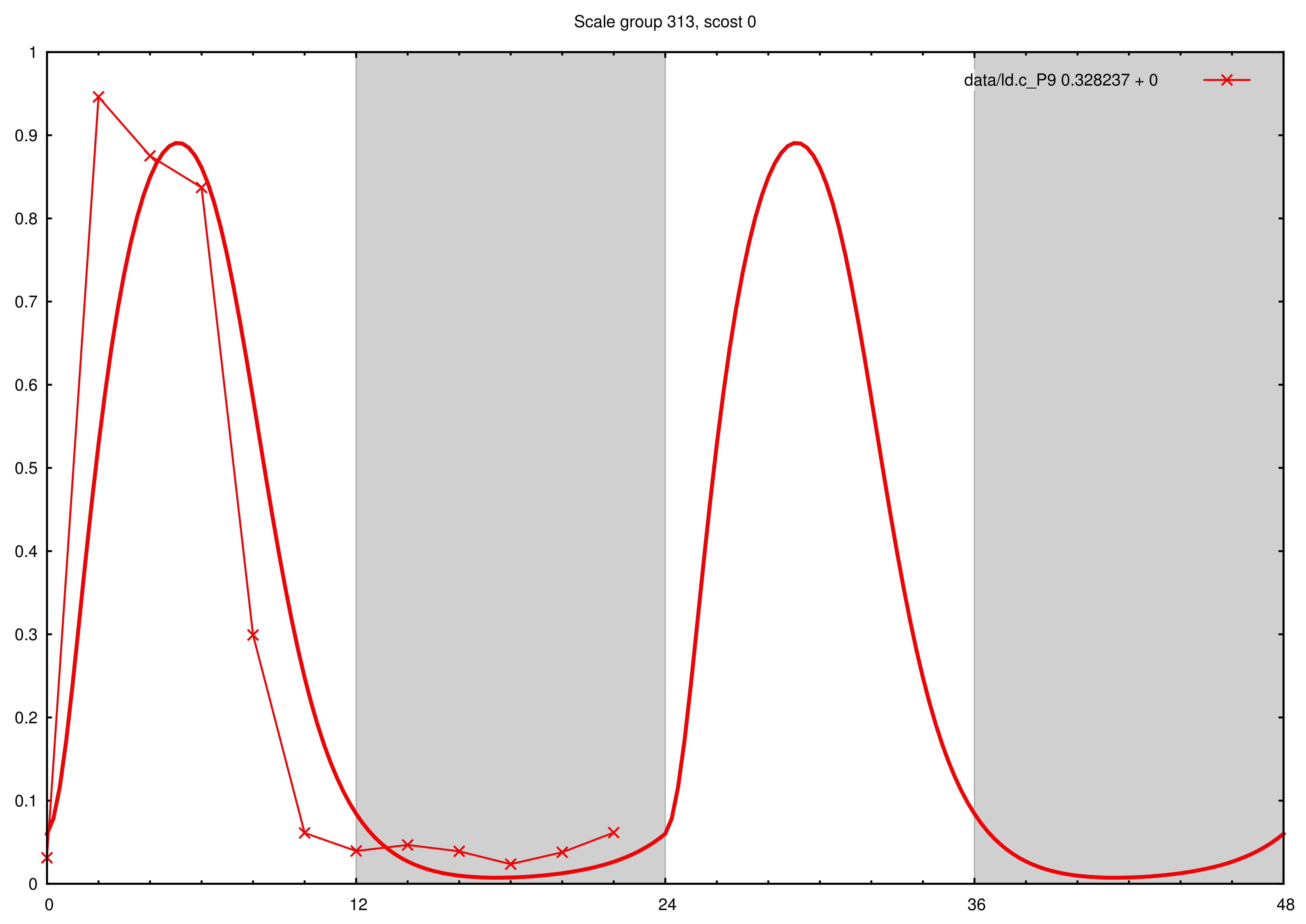
Scale group 313, scost 0
data/ld.c_P9 0.328237 + 0
1
0.9
0.8
0.7
0.6
0.5
0.4
0.3
0.2
0.1
0
0
12
24
36
48

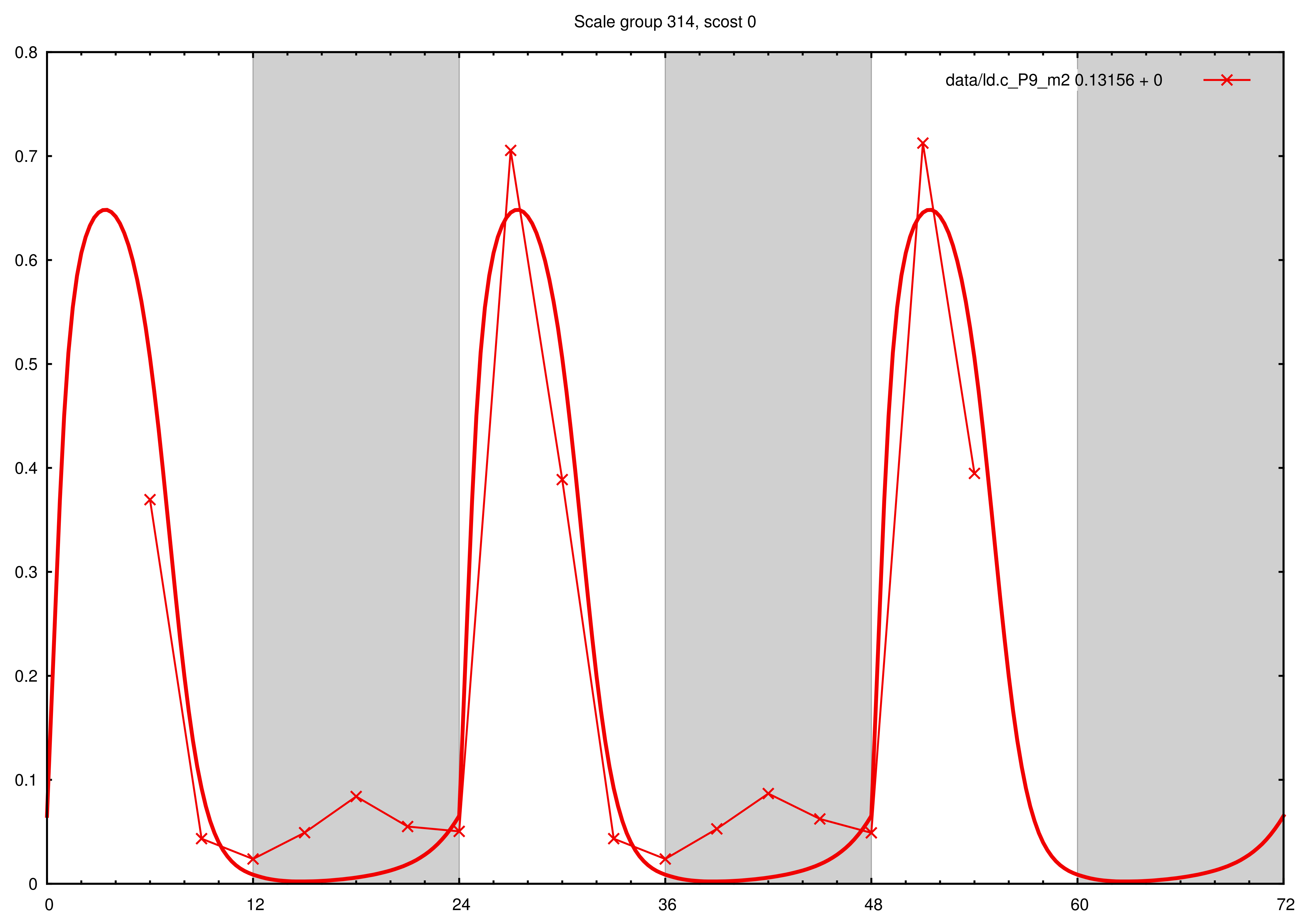

Scale group 314, scost 0
data/ld.c_P9_m2 0.13156 + 0
0.8
0.7
0.6
0.5
0.4
0.3
0.2
0.1
0
0
12
24
36
48
60
72

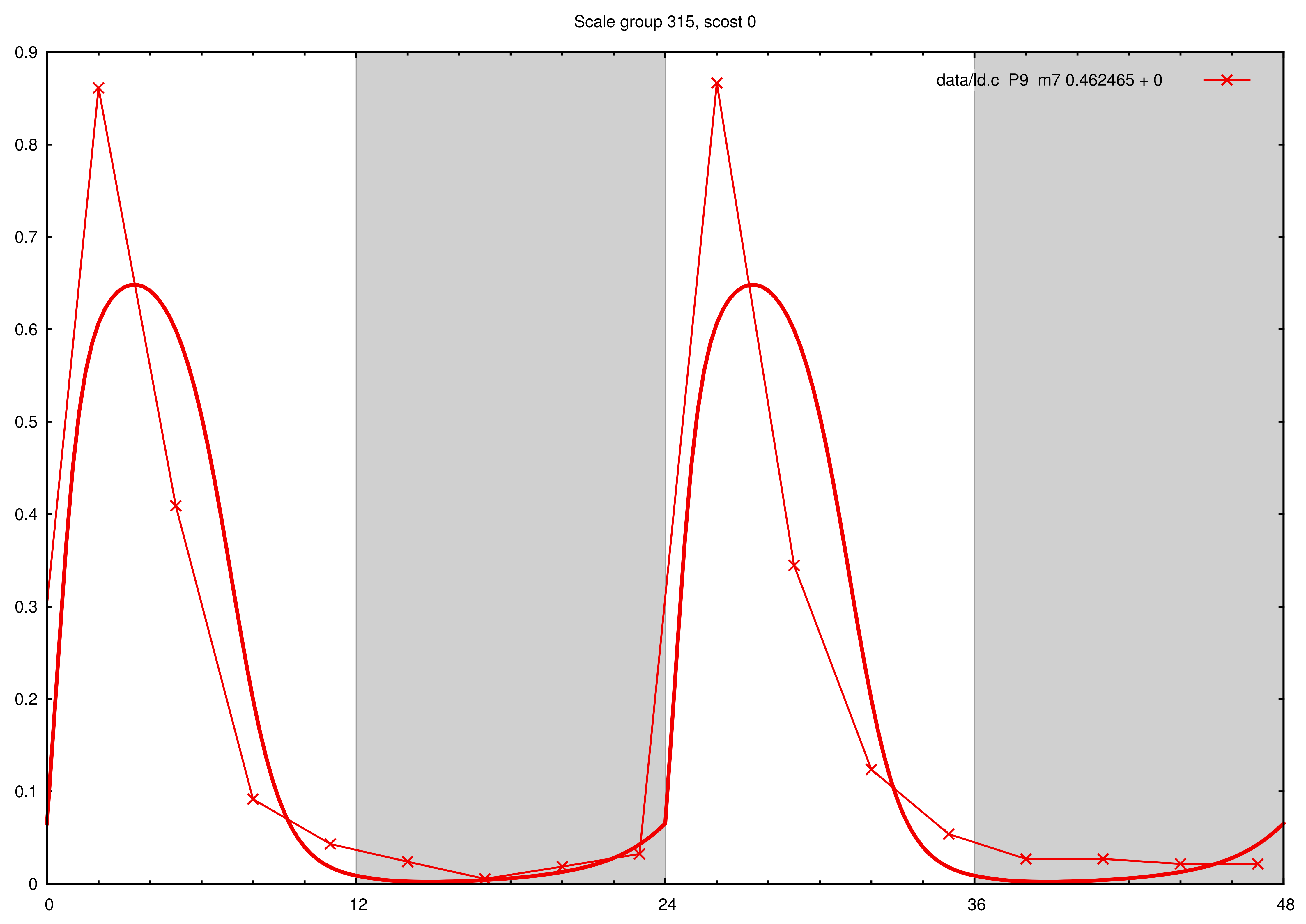

Scale group 315, scost 0
data/ld.c_P9_m7 0.462465 + 0
0.9
0.8
0.7
0.6
0.5
0.4
0.3
0.2
0.1
0
0
12
24
36
48

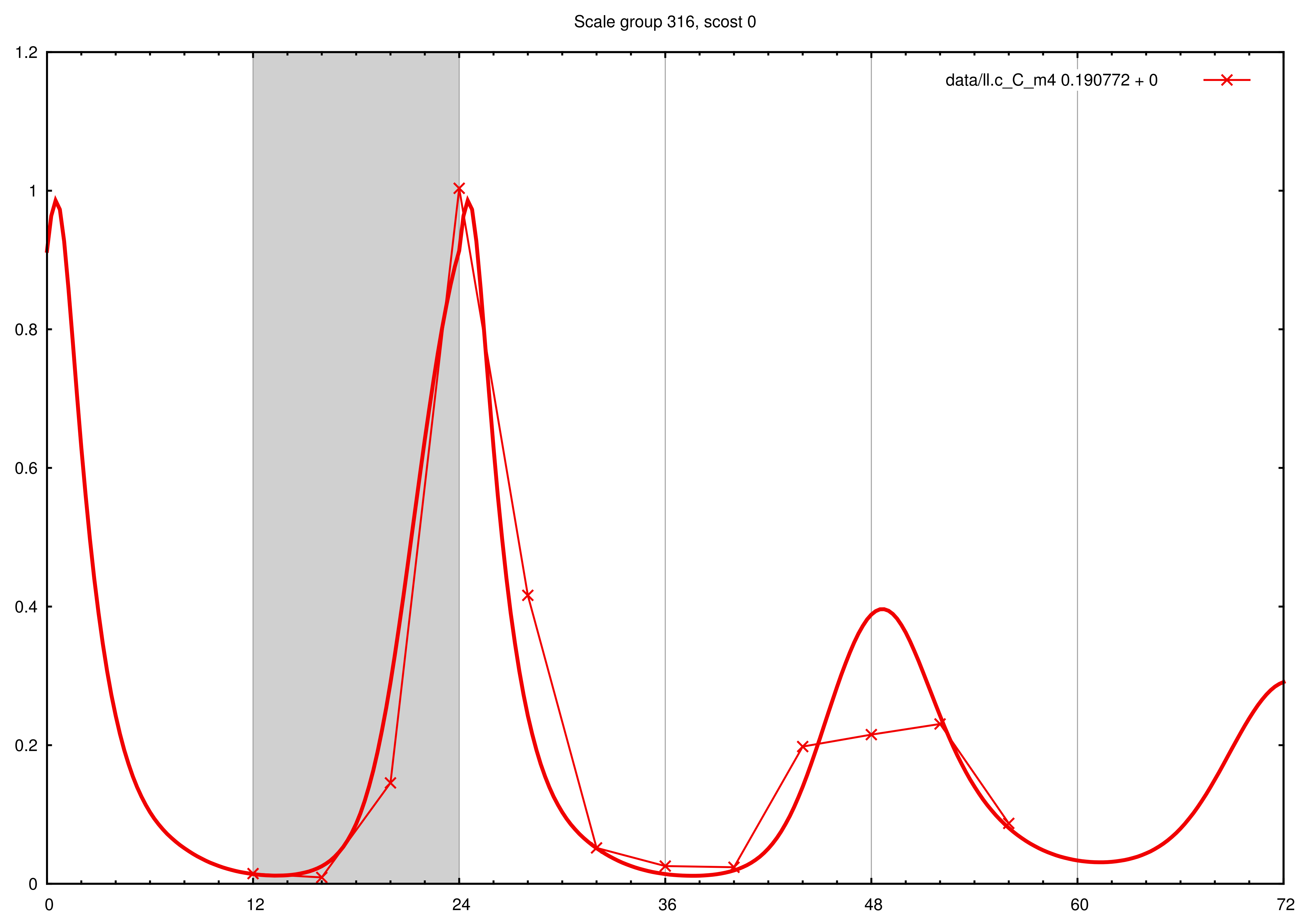

Scale group 316, scost 0
data/ll.c_C_m4 0.190772 + 0
1.2
1
0.8
0.6
0.4
0.2
0
0
12
24
36
48
60
72

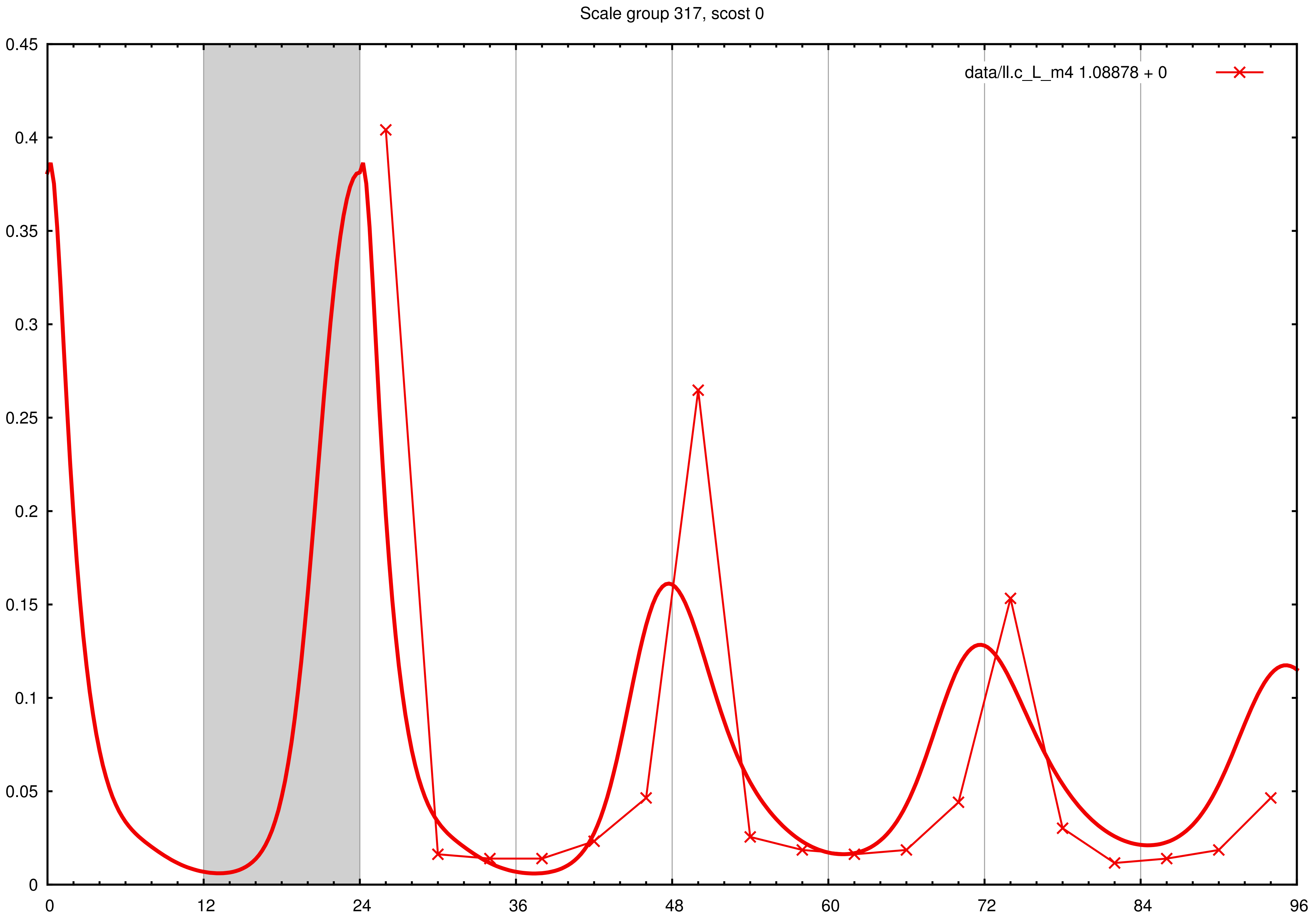

Scale group 317, scost 0
data/ll.c_L_m4 1.08878 + 0
0.45
0.4
0.35
0.3
0.25
0.2
0.15
0.1
0.05
0
0
12
24
36
48
60
72
84
96

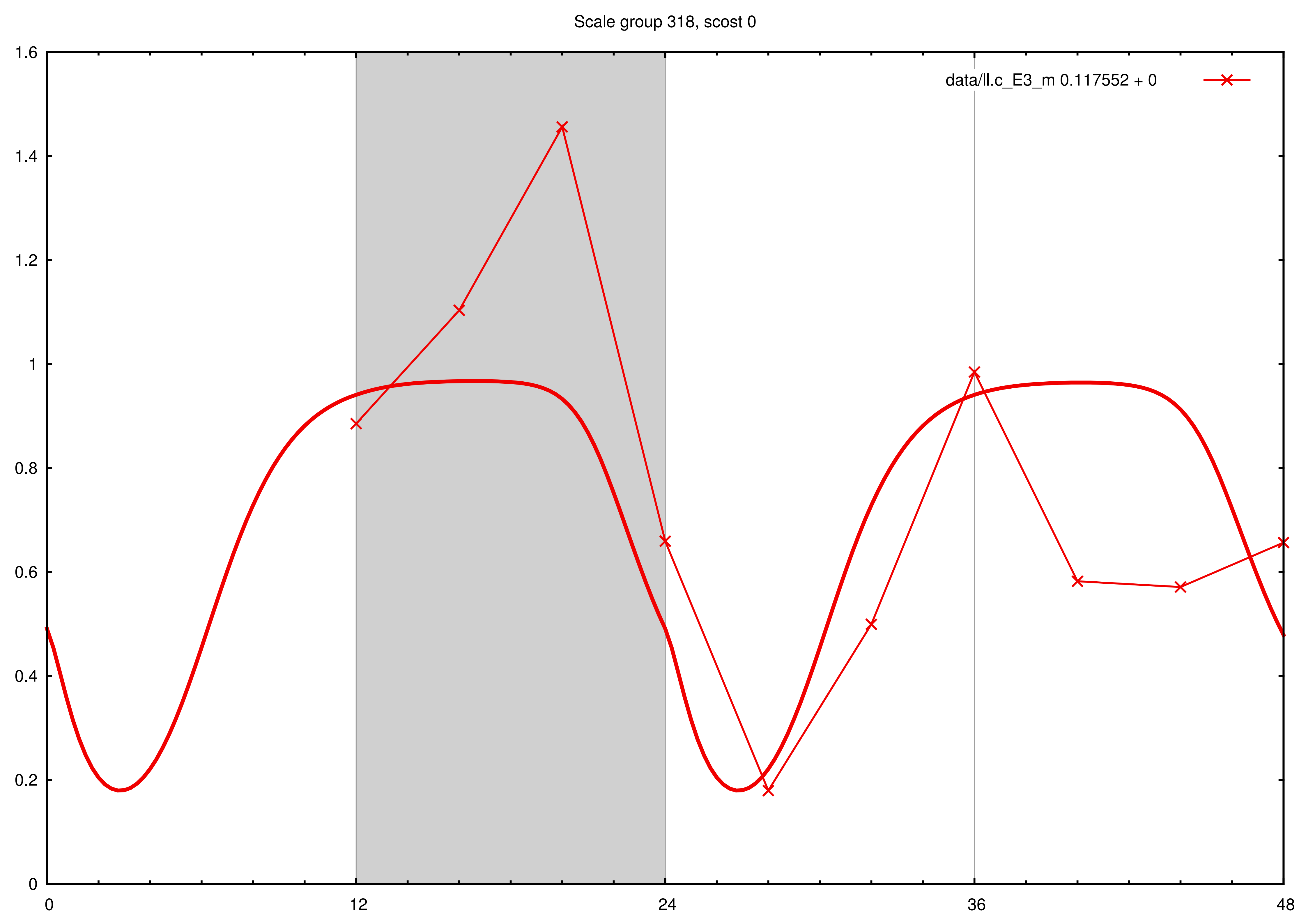
Scale group 318, scost 0
data/ll.c_E3_m 0.117552 + 0
1.6
1.4
1.2
1
0.8
0.6
0.4
0.2
0
0
12
24
36
48

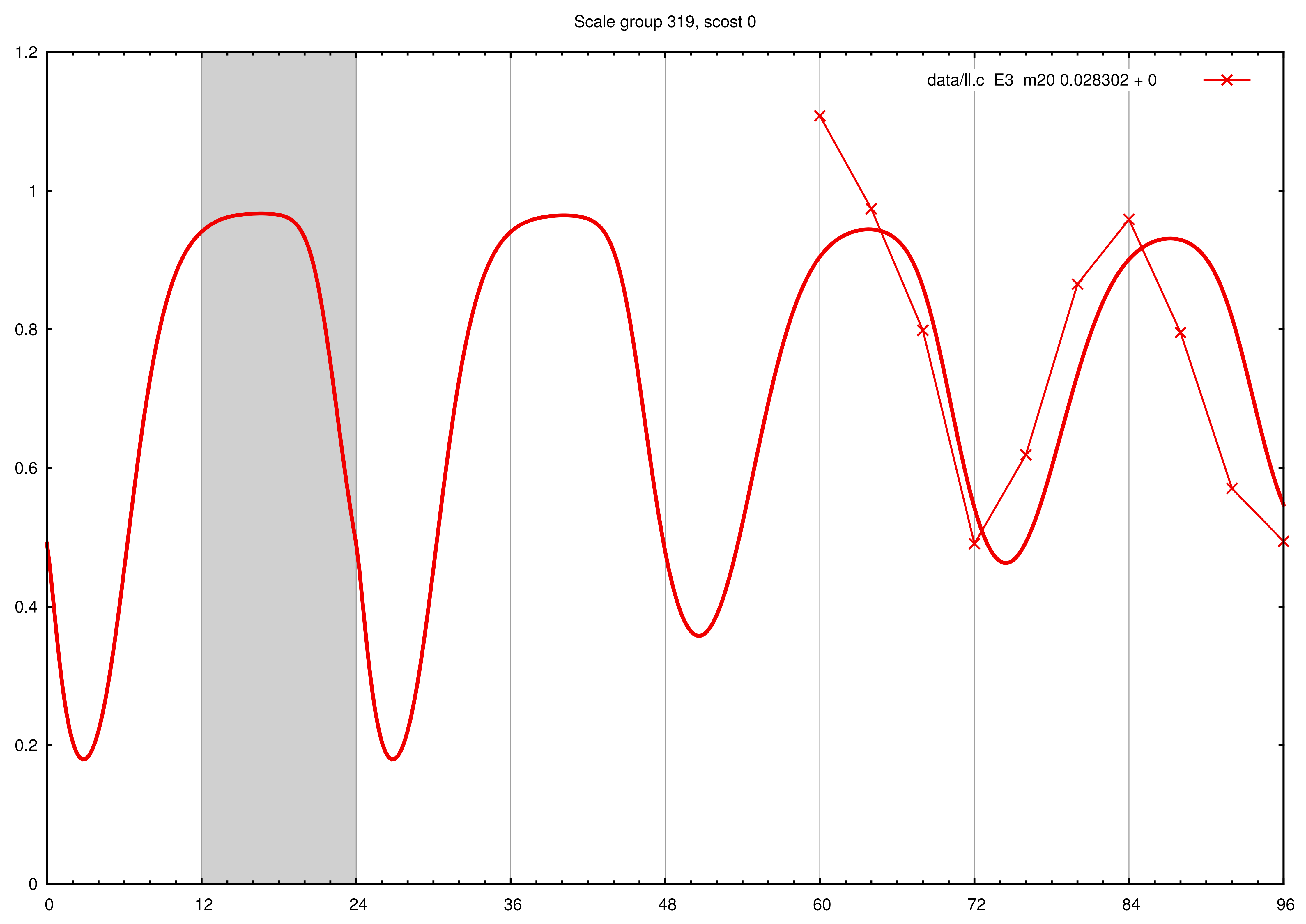

Scale group 319, scost 0
data/ll.c_E3_m20 0.028302 + 0
1.2
1
0.8
0.6
0.4
0.2
0
0
12
24
36
48
60
72
84
96

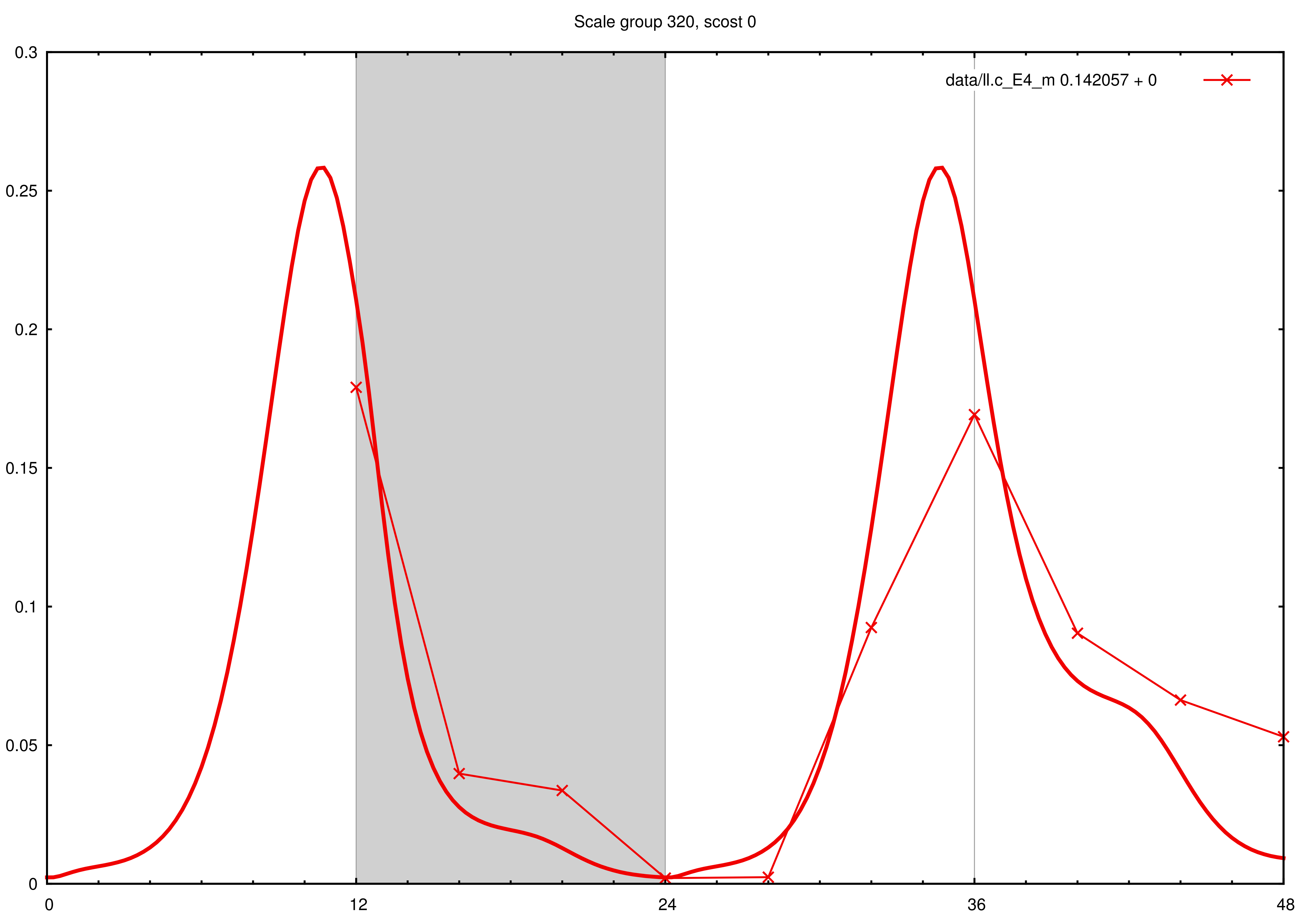
Scale group 320, scost 0
data/ll.c_E4_m 0.142057 + 0
0.3
0.25
0.2
0.15
0.1
0.05
0
0
12
24
36
48

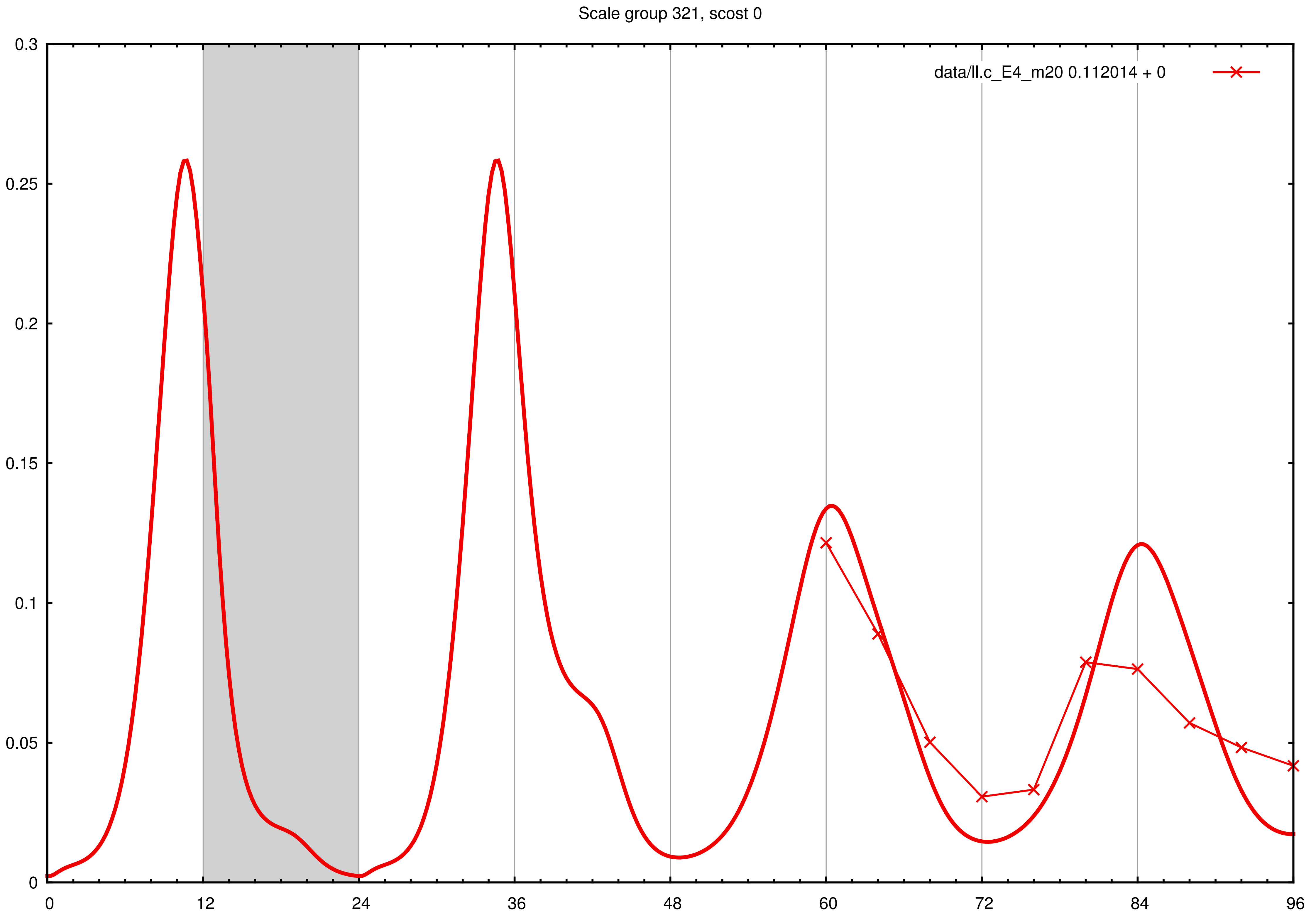

Scale group 321, scost 0
data/ll.c_E4_m20 0.112014 + 0
0.3
0.25
0.2
0.15
0.1
0.05
0
0
12
24
36
48
60
72
84
96

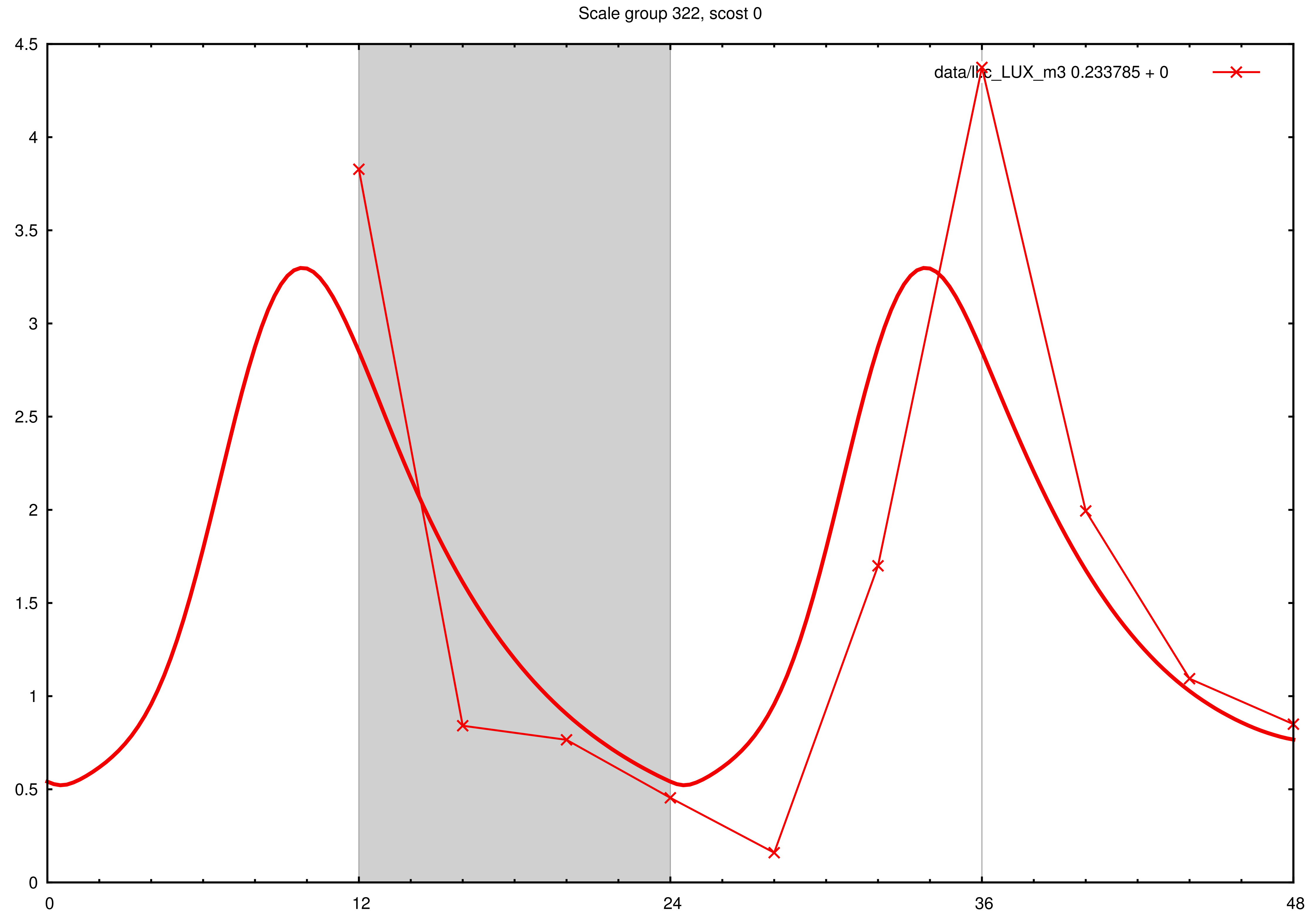

Scale group 322, scost 0
data/lhc_LUX_m3 0.233785 + 0
4.5
4
3.5
3
2.5
2
1.5
1
0.5
0
0
12
24
36
48

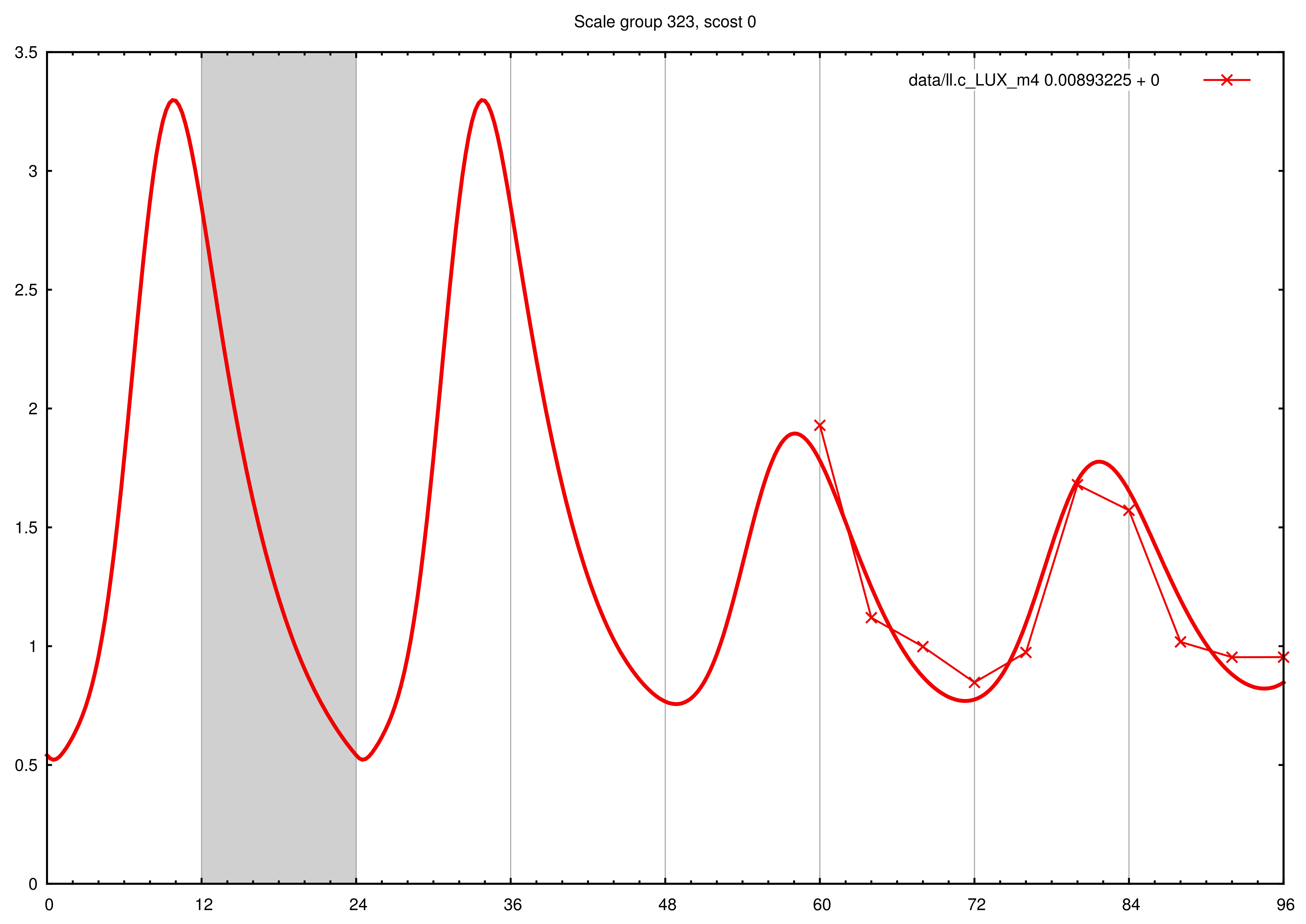

Scale group 323, scost 0
data/ll.c_LUX_m4 0.00893225 + 0
3.5
3
2.5
2
1.5
1
0.5
0
0
12
24
36
48
60
72
84
96

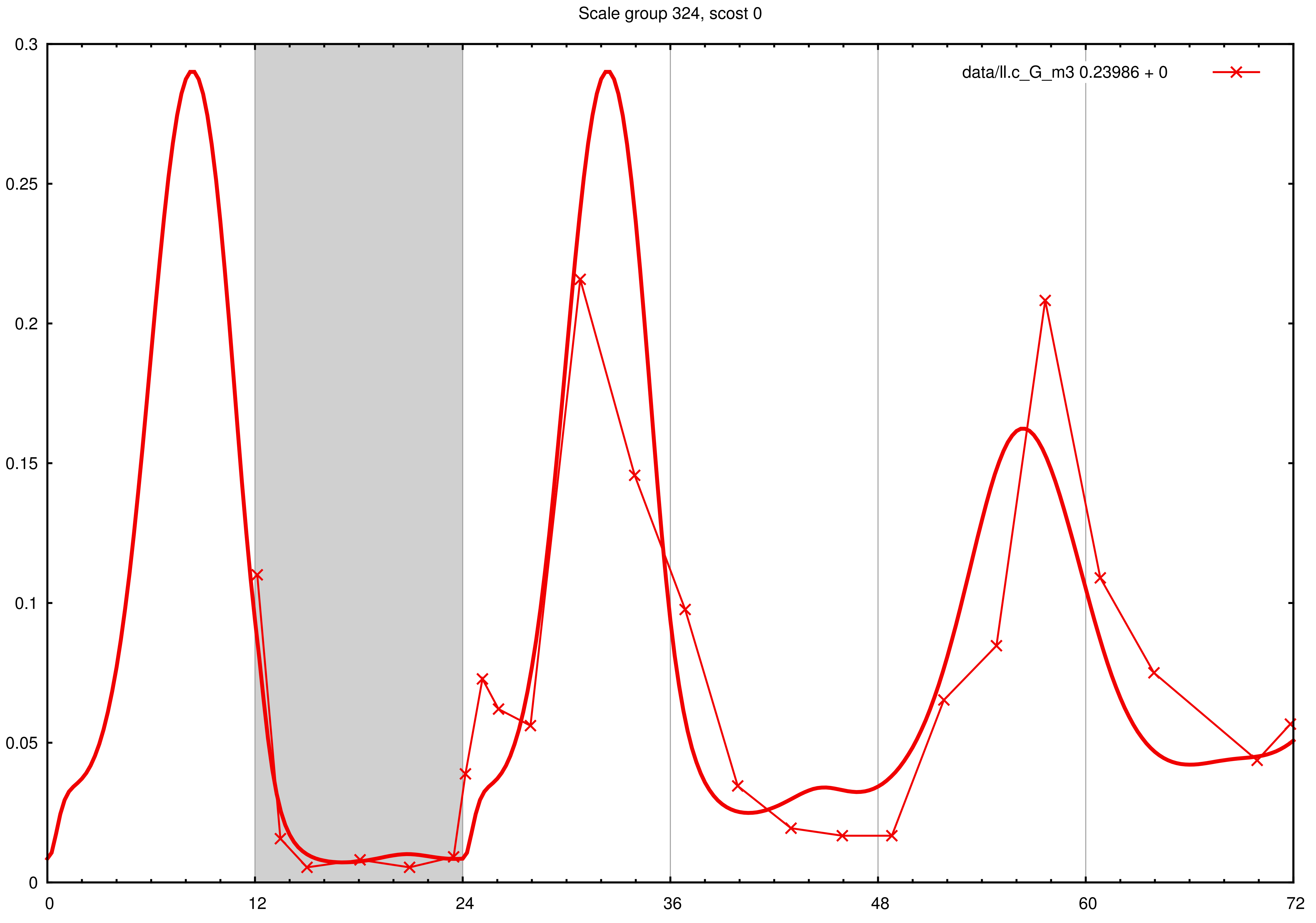

Scale group 324, scost 0
data/ll.c_G_m3 0.23986 + 0
0.3
0.25
0.2
0.15
0.1
0.05
0
0
12
24
36
48
60
72

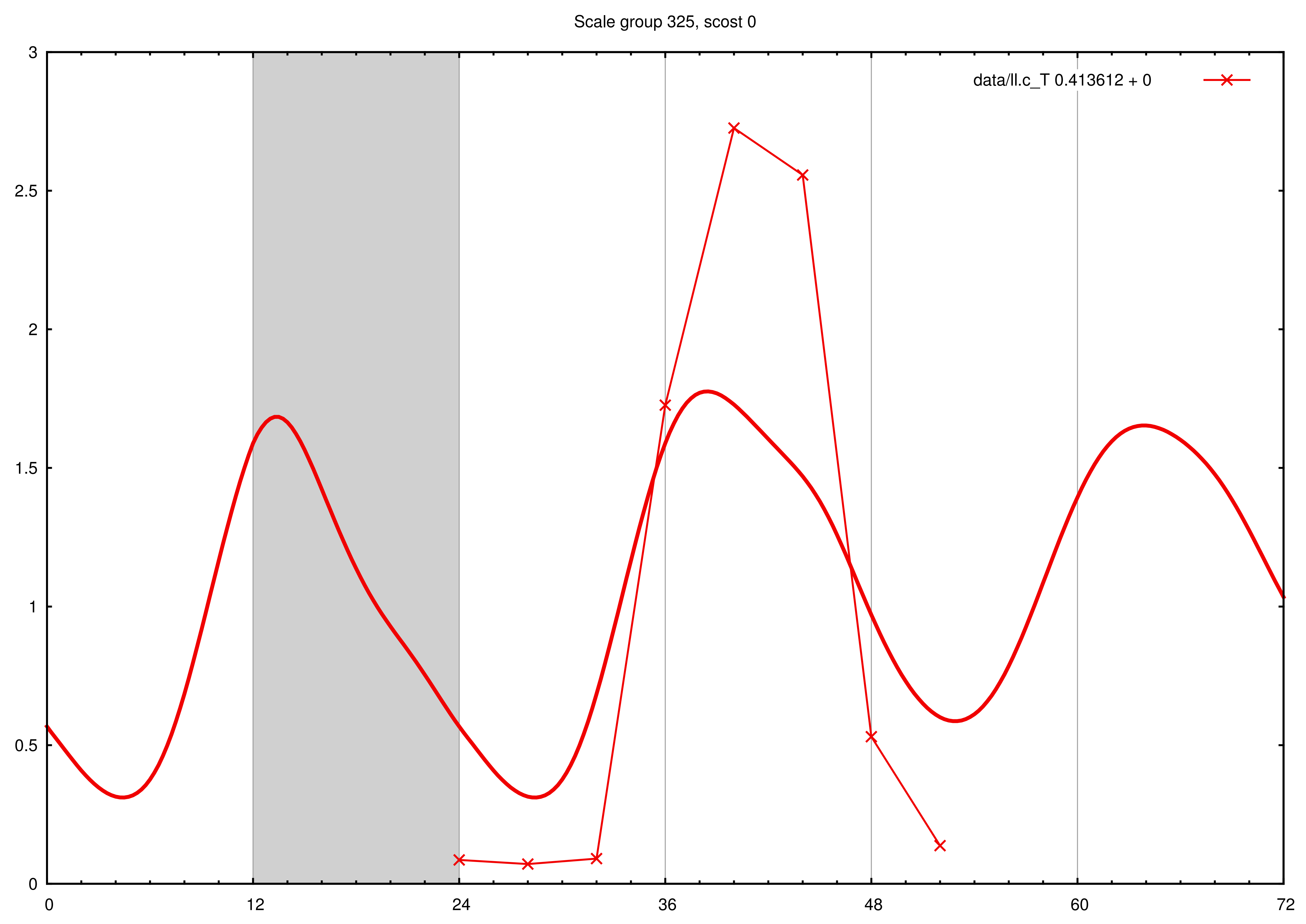

Scale group 325, scost 0
data/ll.c_T 0.413612 + 0
3
2.5
2
1.5
1
0.5
0
0
12
24
36
48
60
72

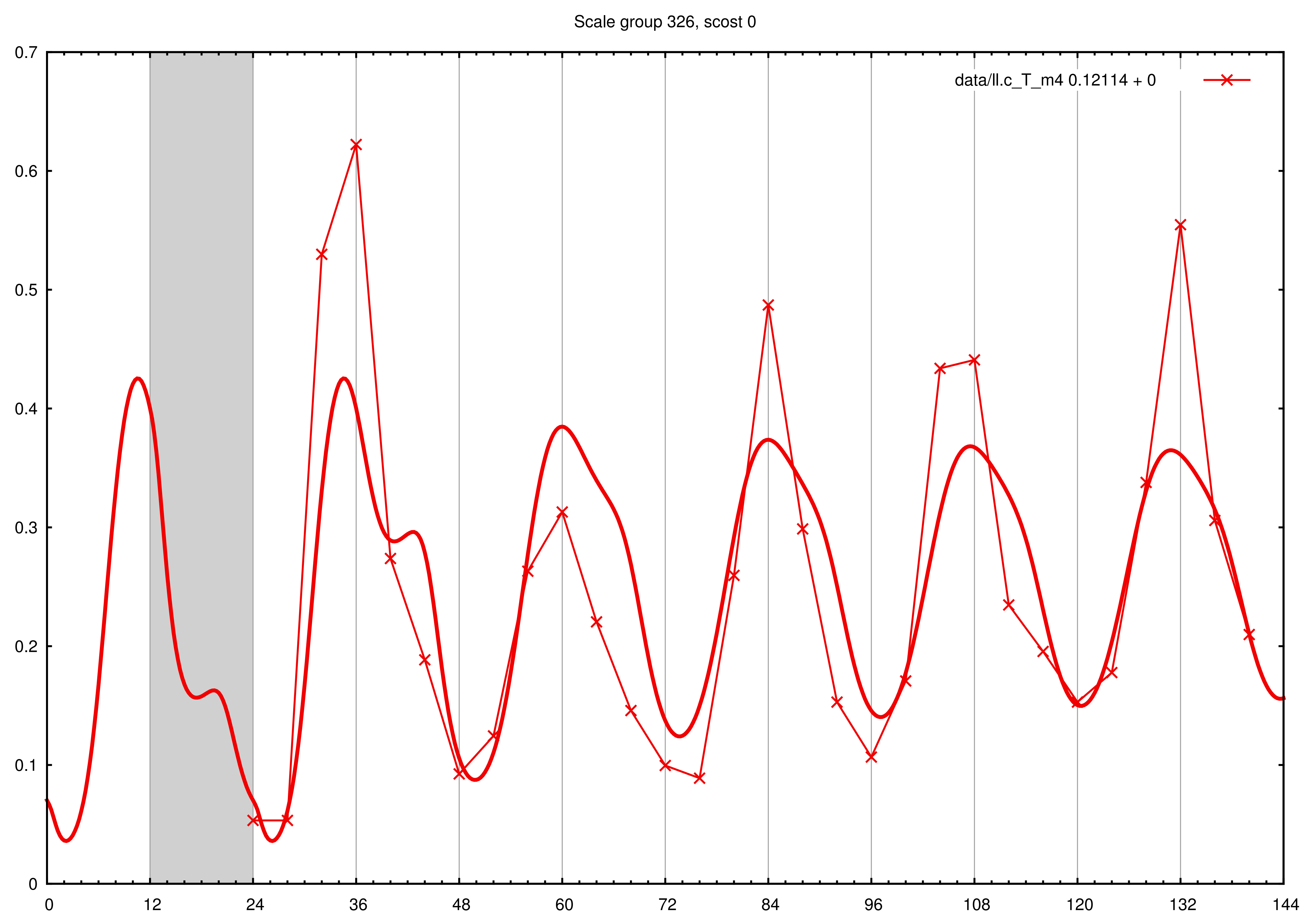

Scale group 326, scost 0
data/ll.c_T_m4 0.12114 + 0
0
0.1
0.2
0.3
0.4
0.5
0.6
0.7
0
12
24
36
48
60
72
84
96
108
120
132
144

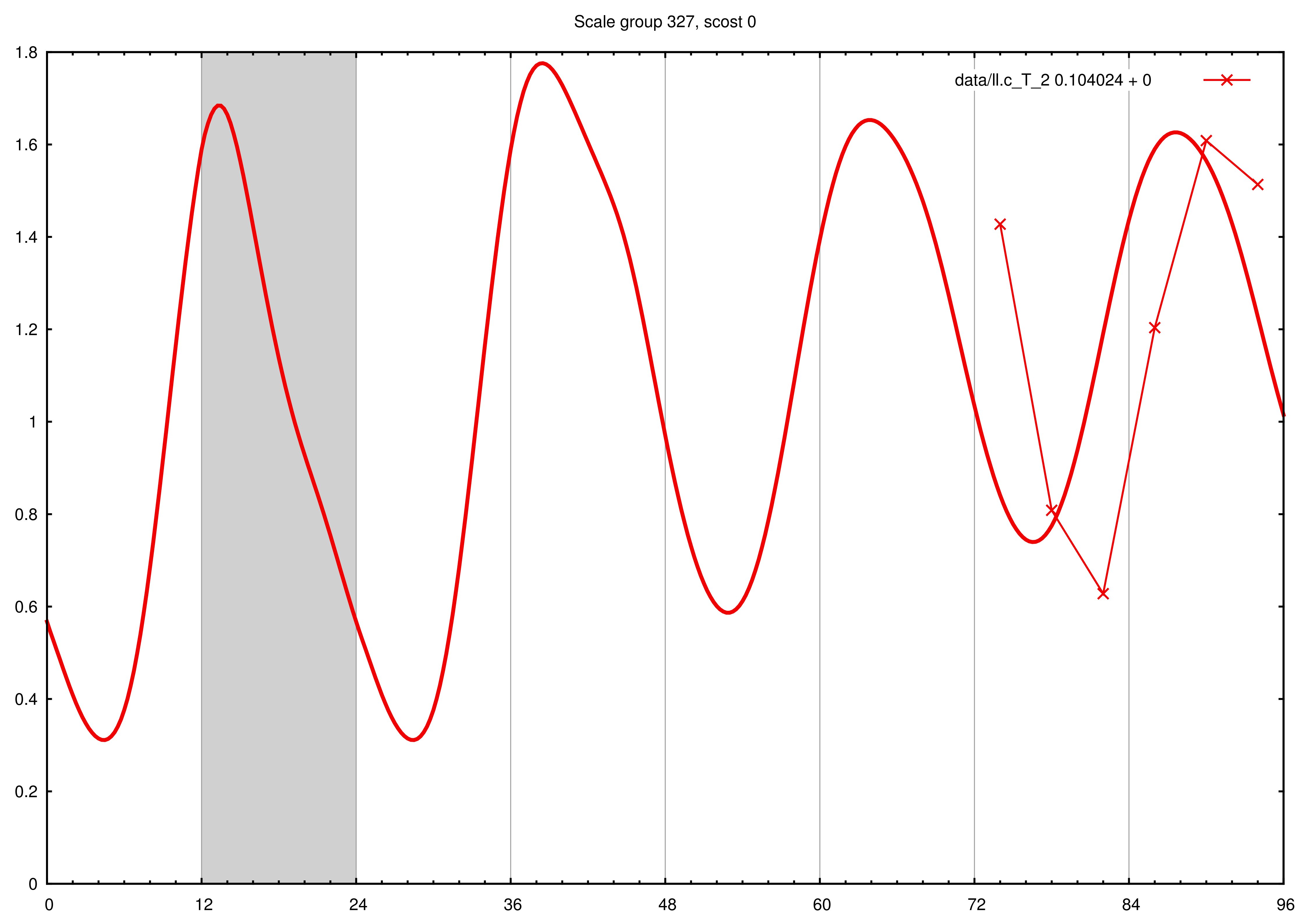
Scale group 327, scost 0
data/ll.c_T_2 0.104024 + 0
1.8
1.6
1.4
1.2
1
0.8
0.6
0.4
0.2
0
0
12
24
36
48
60
72
84
96

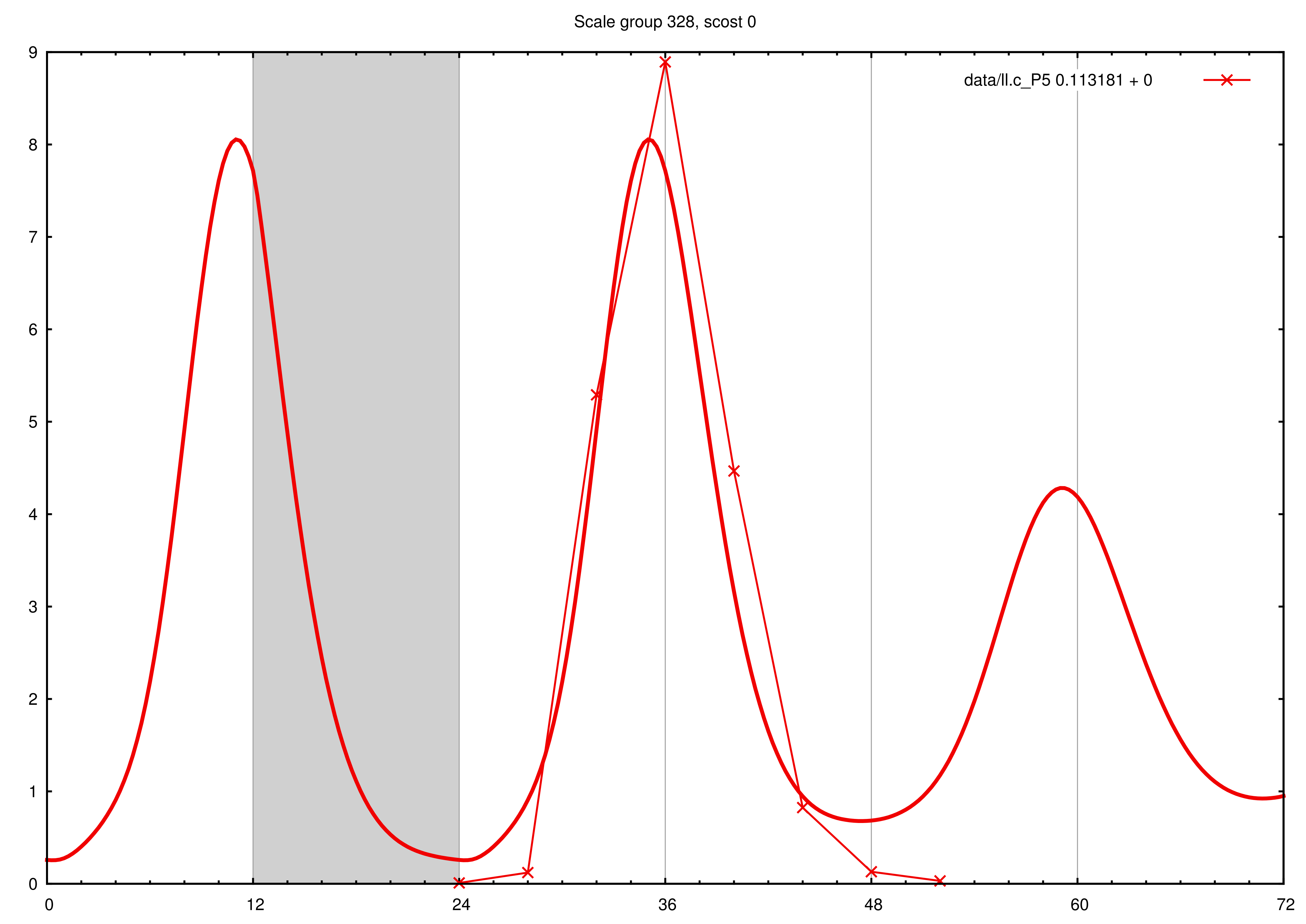

Scale group 328, scost 0
data/ll.c_P5 0.113181 + 0
0
1
2
3
4
5
6
7
8
9
0
12
24
36
48
60
72

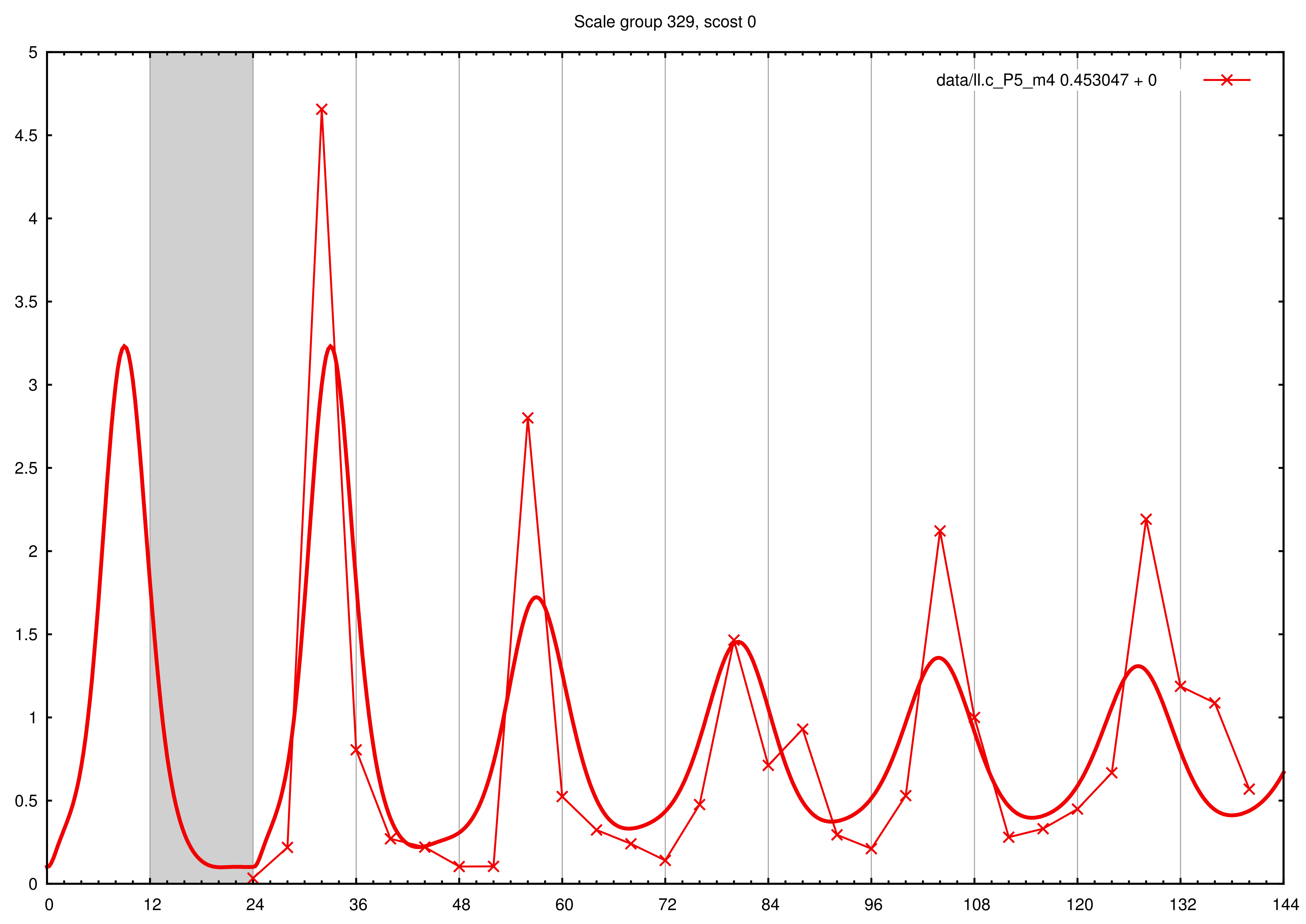

Scale group 329, scost 0
data/ll.c_P5_m4 0.453047 + 0
0
0.5
1
1.5
2
2.5
3
3.5
4
4.5
5
0
12
24
36
48
60
72
84
96
108
120
132
144

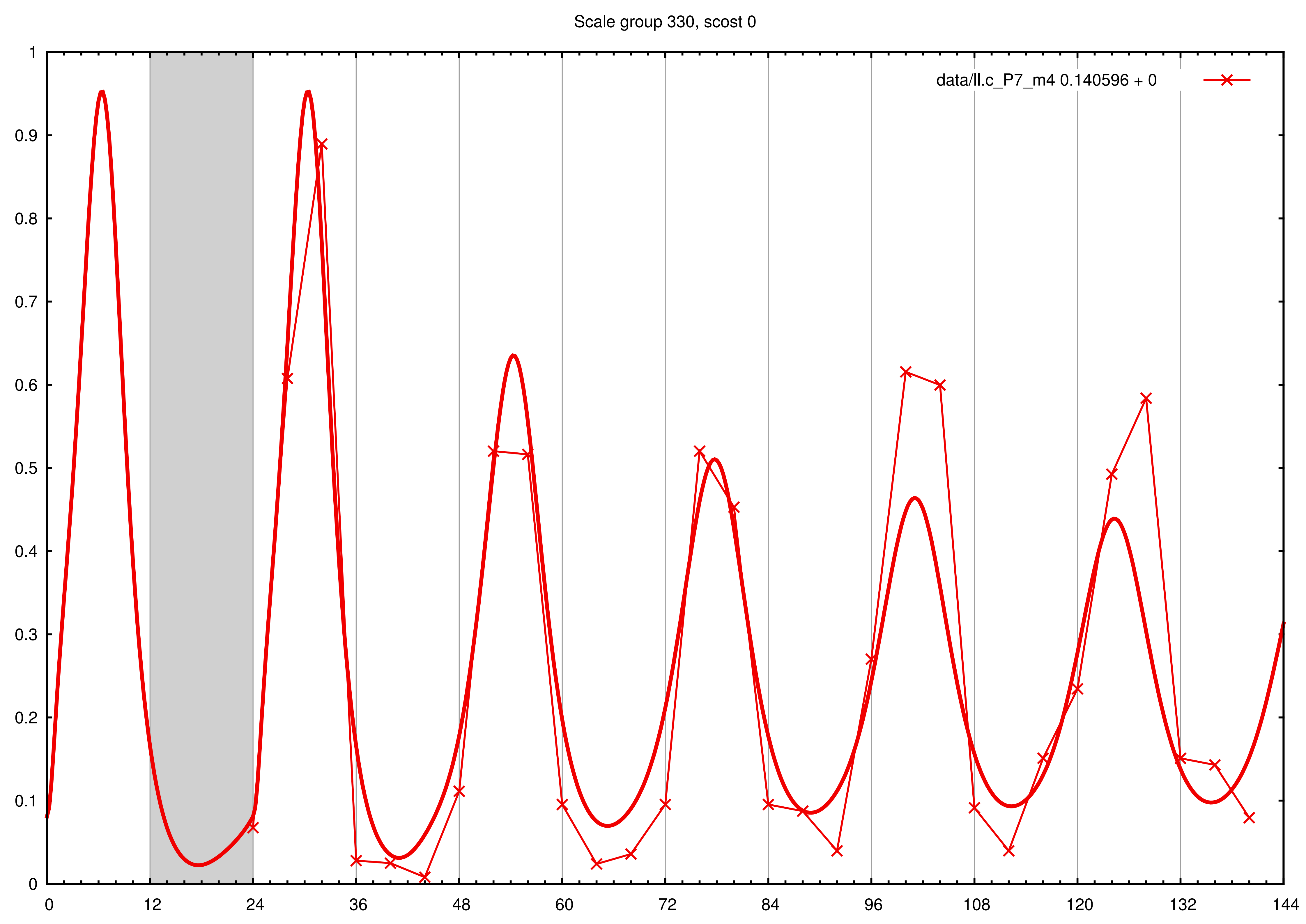

Scale group 330, scost 0
data/ll.c_P7_m4 0.140596 + 0
0
0.1
0.2
0.3
0.4
0.5
0.6
0.7
0.8
0.9
1
0
12
24
36
48
60
72
84
96
108
120
132
144

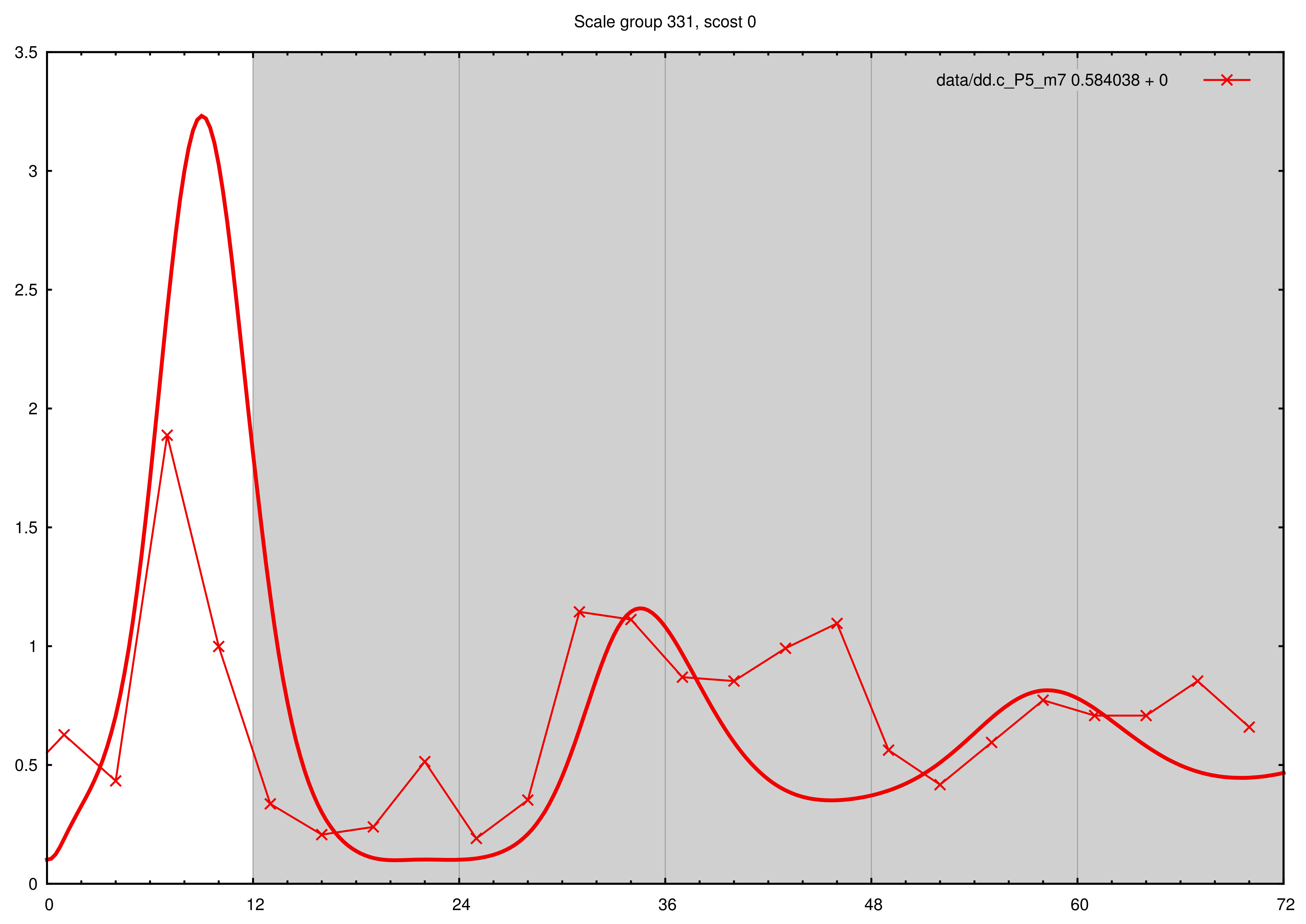

Scale group 331, scost 0
data/dd.c_P5_m7 0.584038 + 0
3.5
3
2.5
2
1.5
1
0.5
0
0
12
24
36
48
60
72

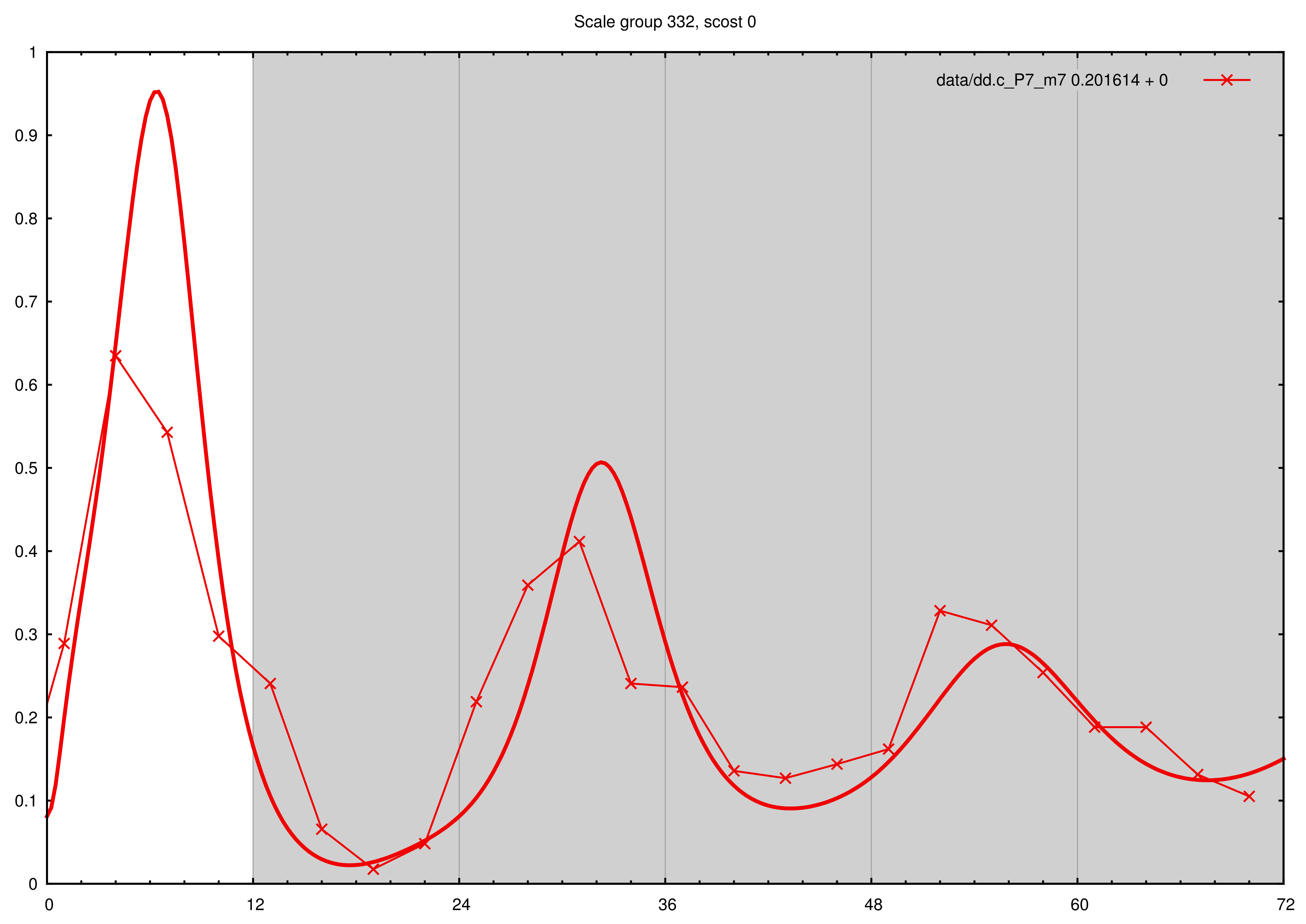

Scale group 332, scost 0
data/dd.c_P7_m7 0.201614 + 0
1
0.9
0.8
0.7
0.6
0.5
0.4
0.3
0.2
0.1
0
0
12
24
36
48
60
72

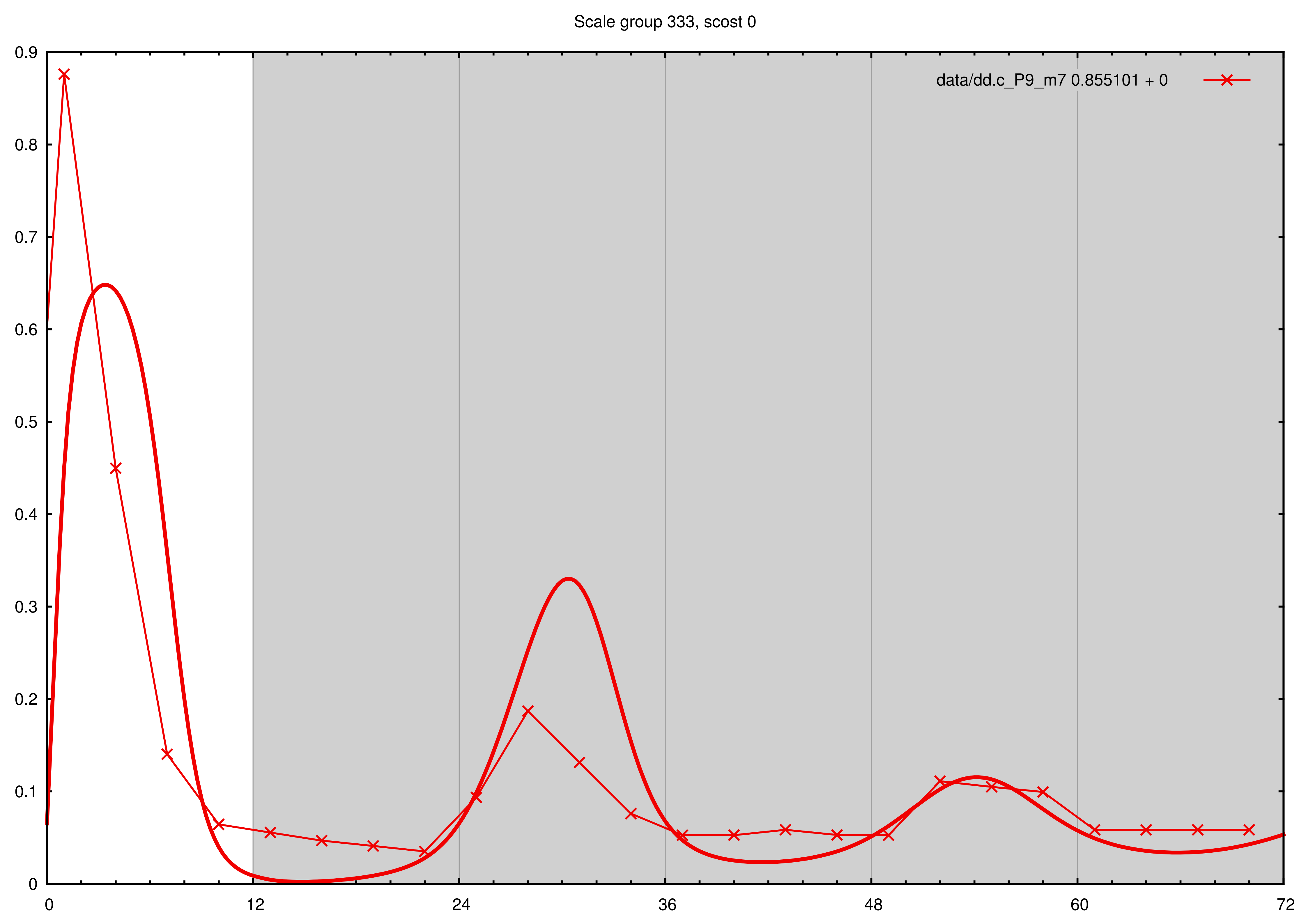
Scale group 333, scost 0
data/dd.c_P9_m7 0.855101 + 0
0
0.1
0.2
0.3
0.4
0.5
0.6
0.7
0.8
0.9
0
12
24
36
48
60
72

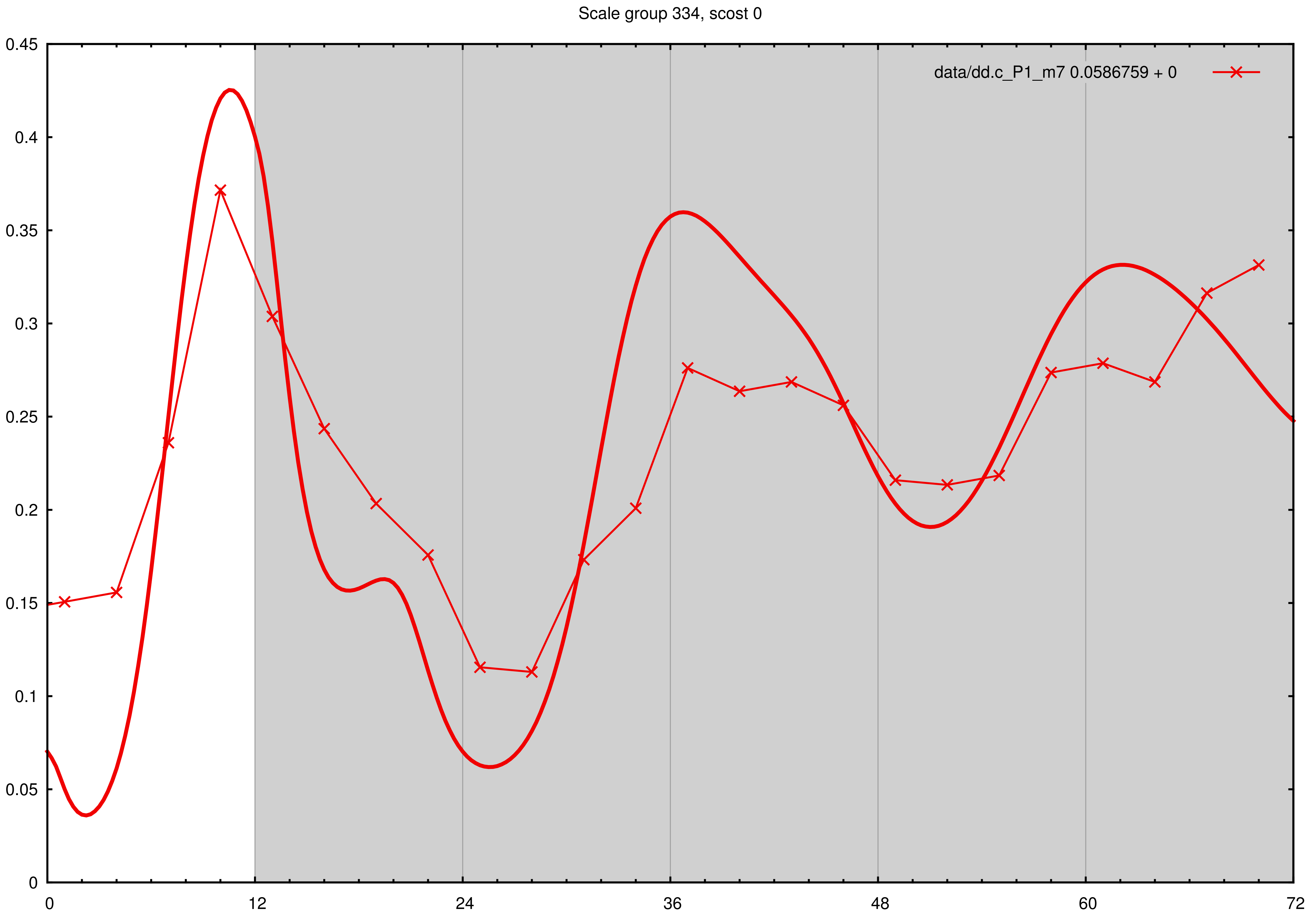

Scale group 334, scost 0
data/dd.c_P1_m7 0.0586759 + 0
0.45
0.4
0.35
0.3
0.25
0.2
0.15
0.1
0.05
0
0
12
24
36
48
60
72

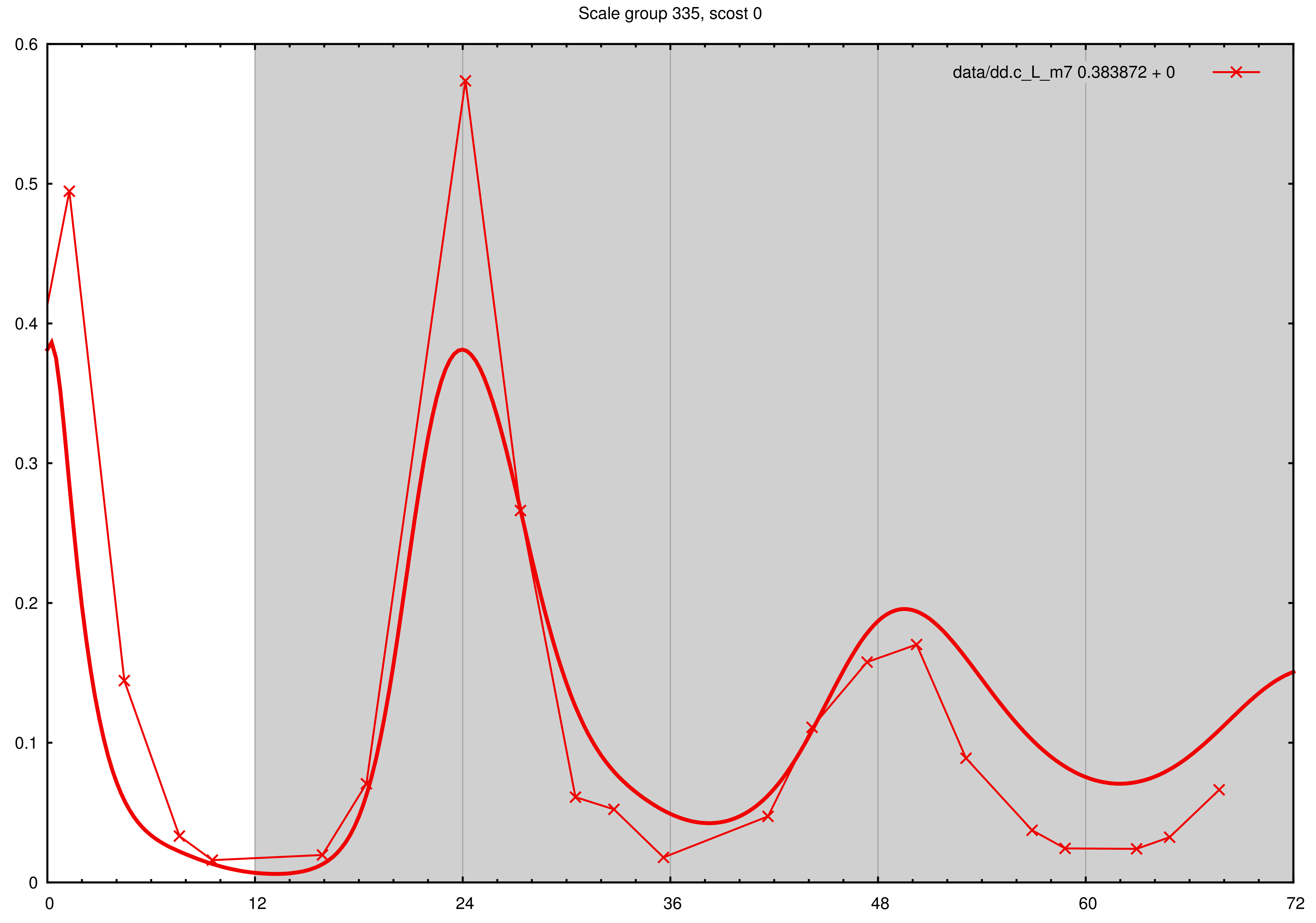

Scale group 335, scost 0
data/dd.c_L_m7 0.383872 + 0
0
0.1
0.2
0.3
0.4
0.5
0.6
0
12
24
36
48
60
72

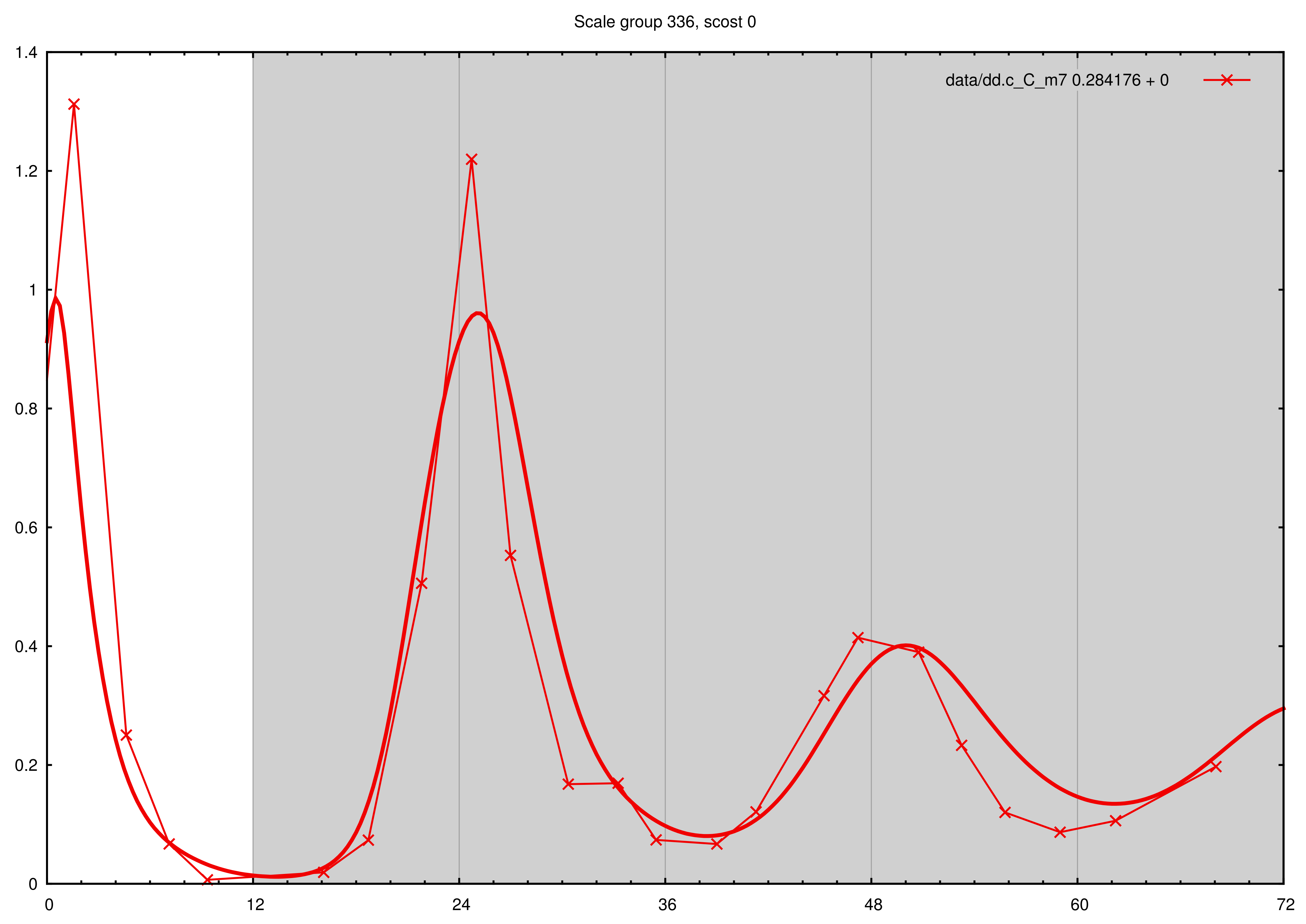

Scale group 336, scost 0
data/dd.c_C_m7 0.284176 + 0
1.4
1.2
1
0.8
0.6
0.4
0.2
0
0
12
24
36
48
60
72

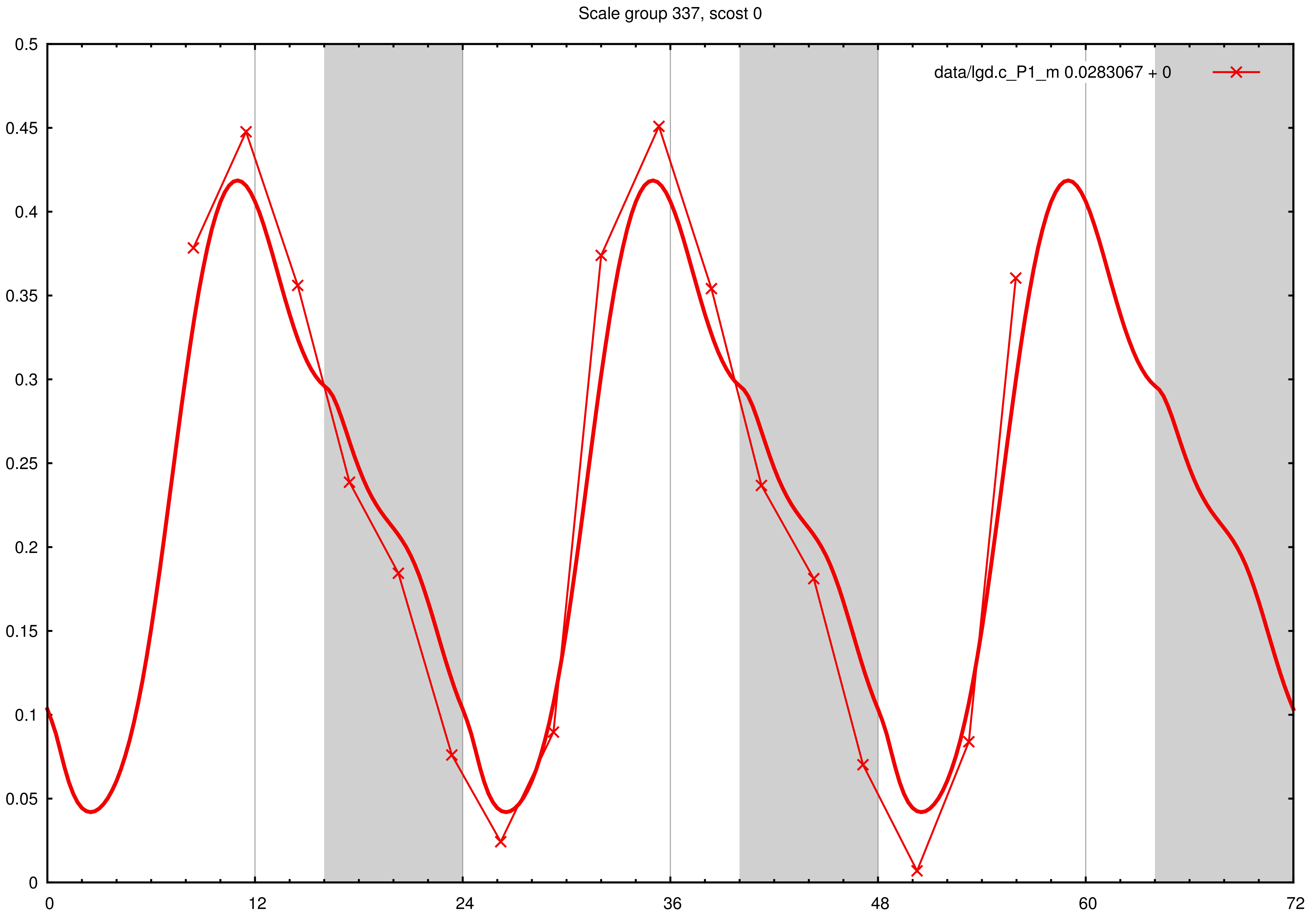

Scale group 337, scost 0
data/lgd.c_P1_m 0.0283067 + 0
0.5
0.45
0.4
0.35
0.3
0.25
0.2
0.15
0.1
0.05
0
0
12
24
36
48
60
72

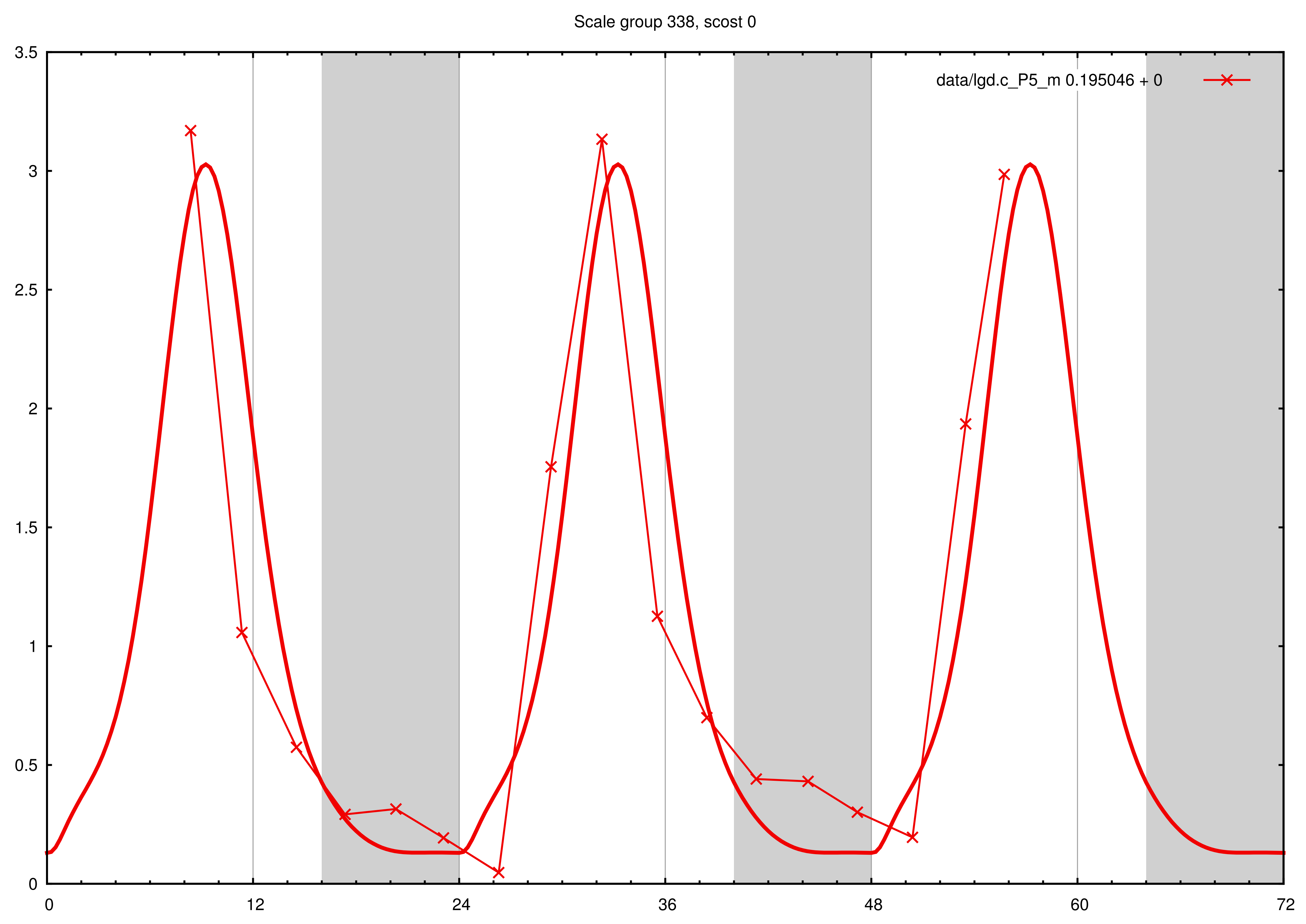
Scale group 338, scost 0
data/lgd.c_P5_m 0.195046 + 0
3.5
3
2.5
2
1.5
1
0.5
0
0
12
24
36
48
60
72

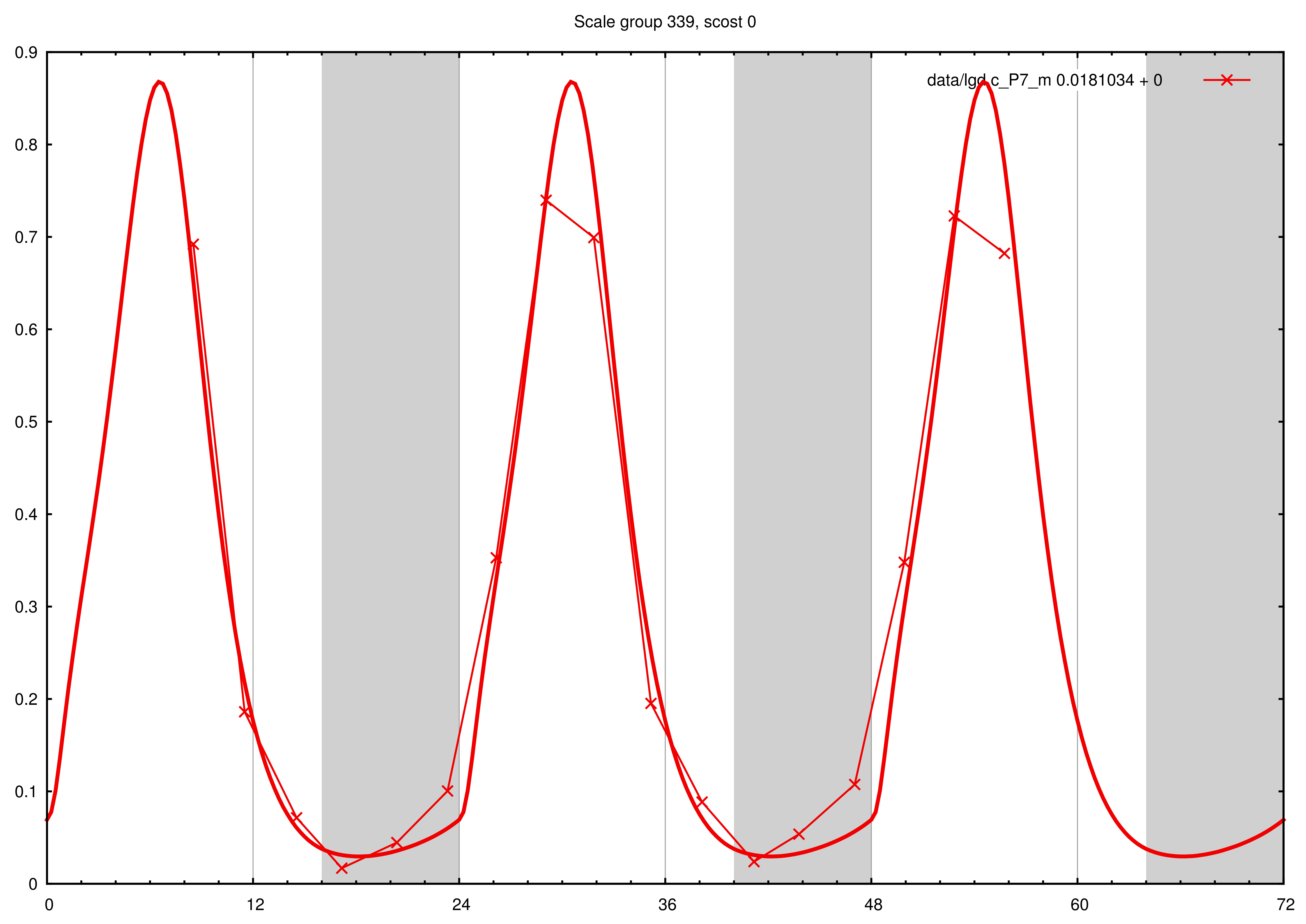
Scale group 339, scost 0
data/lgd.c_P7_m 0.0181034 + 0
0.9
0.8
0.7
0.6
0.5
0.4
0.3
0.2
0.1
0
0
12
24
36
48
60
72

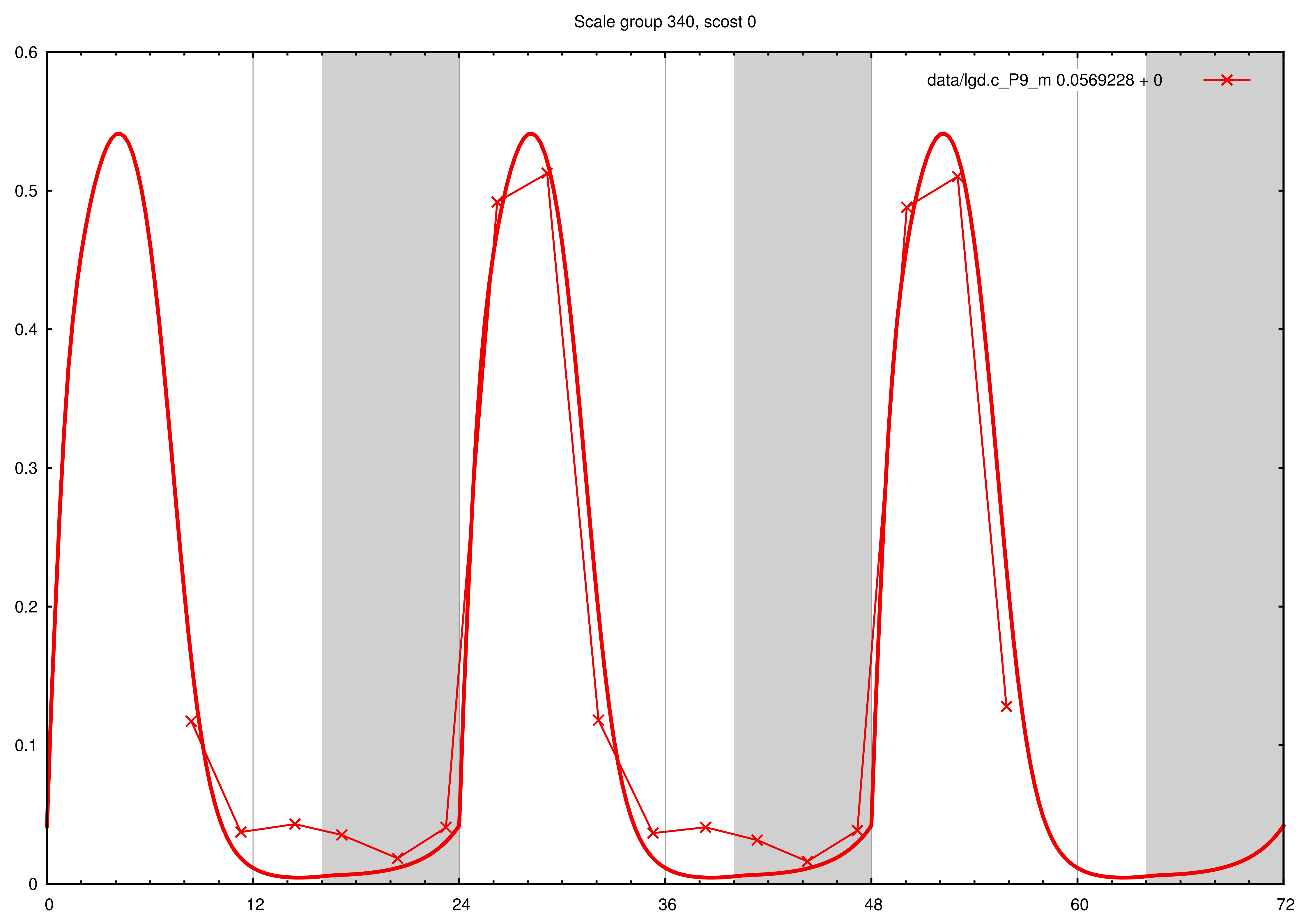
Scale group 340, scost 0
data/lgd.c_P9_m 0.0569228 + 0
0
0.1
0.2
0.3
0.4
0.5
0.6
0
12
24
36
48
60
72

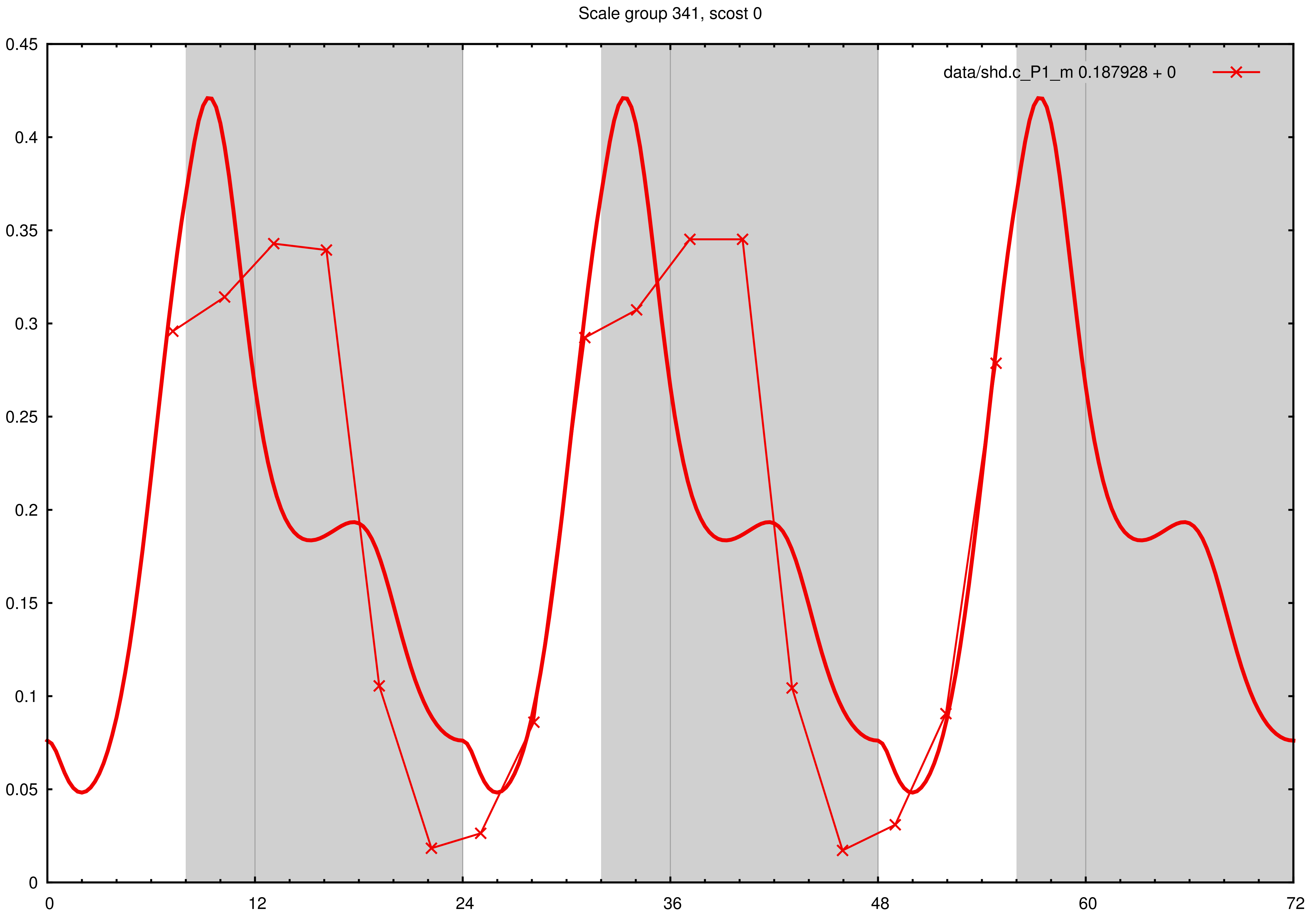

Scale group 341, scost 0
data/shd.c_P1_m 0.187928 + 0
0.45
0.4
0.35
0.3
0.25
0.2
0.15
0.1
0.05
0
0
12
24
36
48
60
72

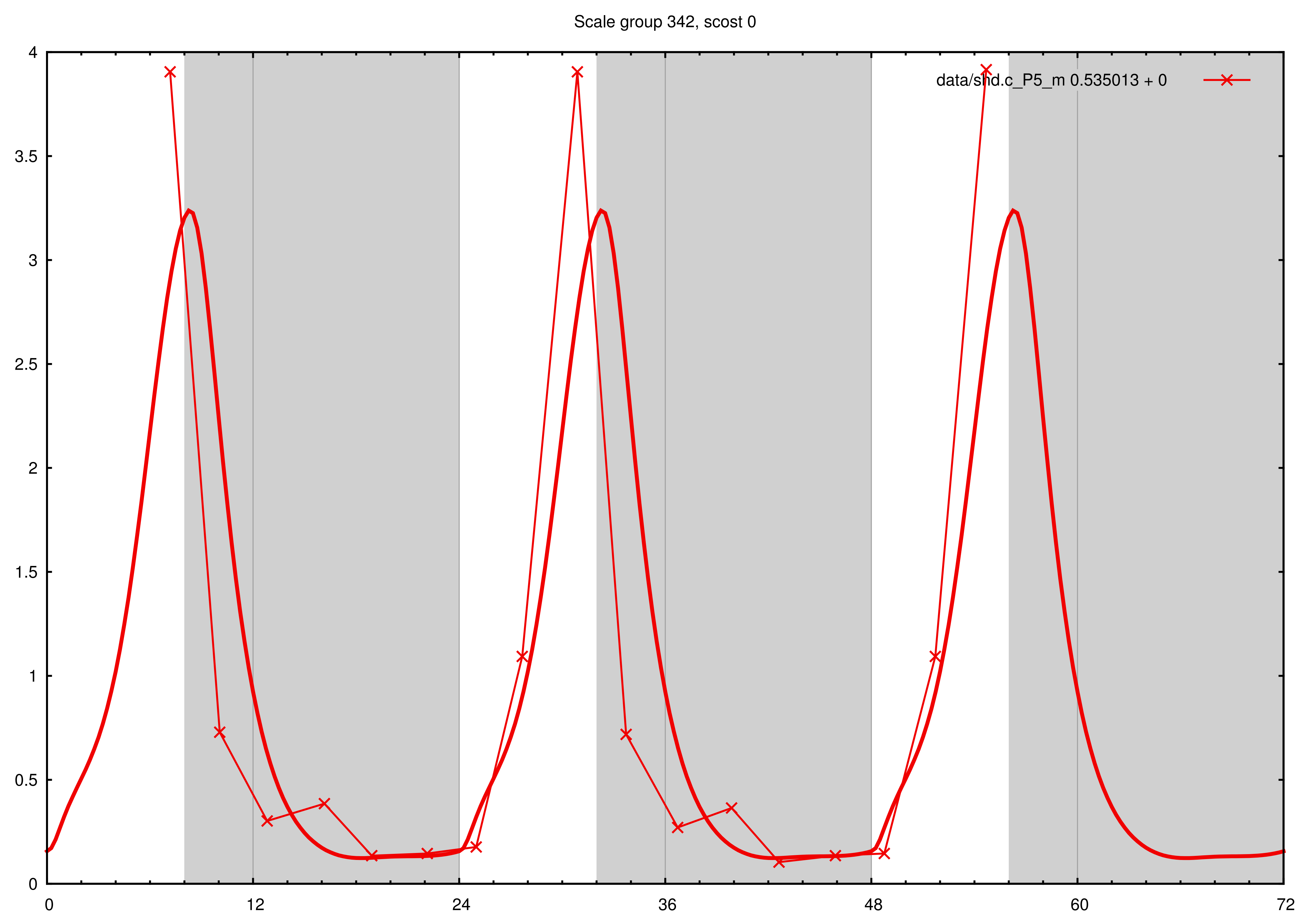

Scale group 342, scost 0
data/shd.c_P5_m 0.535013 + 0
0
0.5
1
1.5
2
2.5
3
3.5
4
0
12
24
36
48
60
72

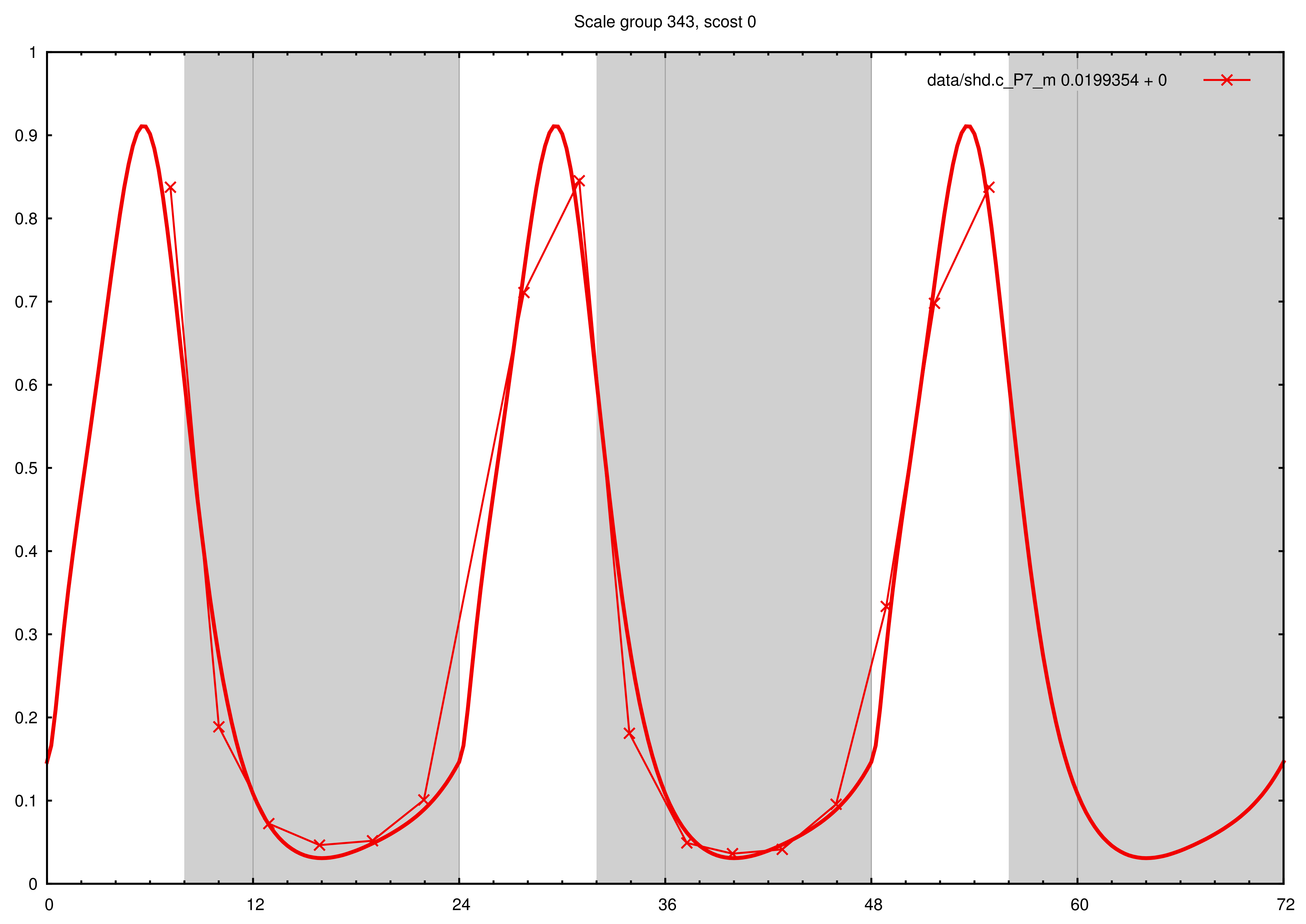

Scale group 343, scost 0
data/shd.c_P7_m 0.0199354 + 0
0
0.1
0.2
0.3
0.4
0.5
0.6
0.7
0.8
0.9
1
0
12
24
36
48
60
72

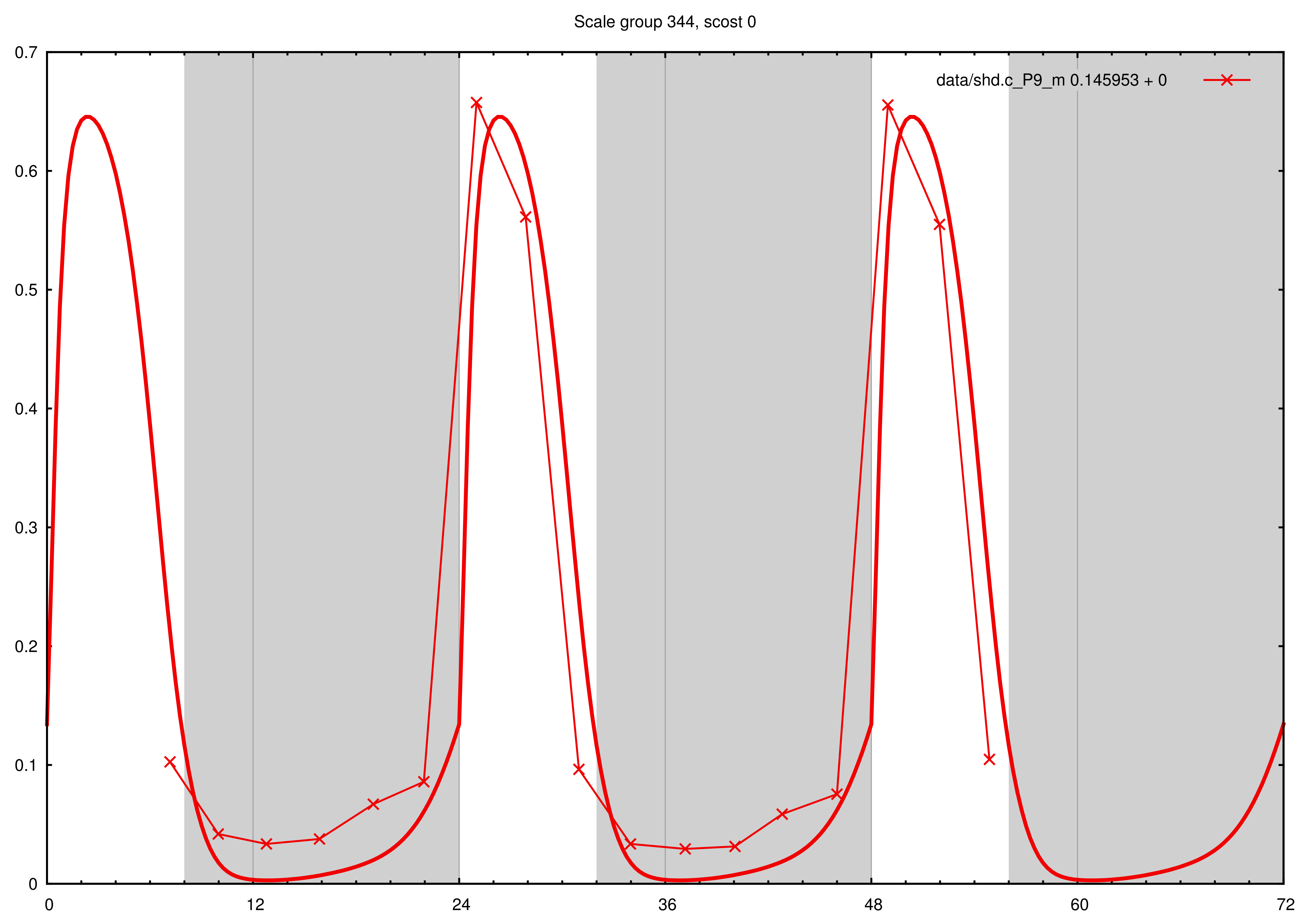

Scale group 344, scost 0
data/shd.c_P9_m 0.145953 + 0
0.7
0.6
0.5
0.4
0.3
0.2
0.1
0
0
12
24
36
48
60
72

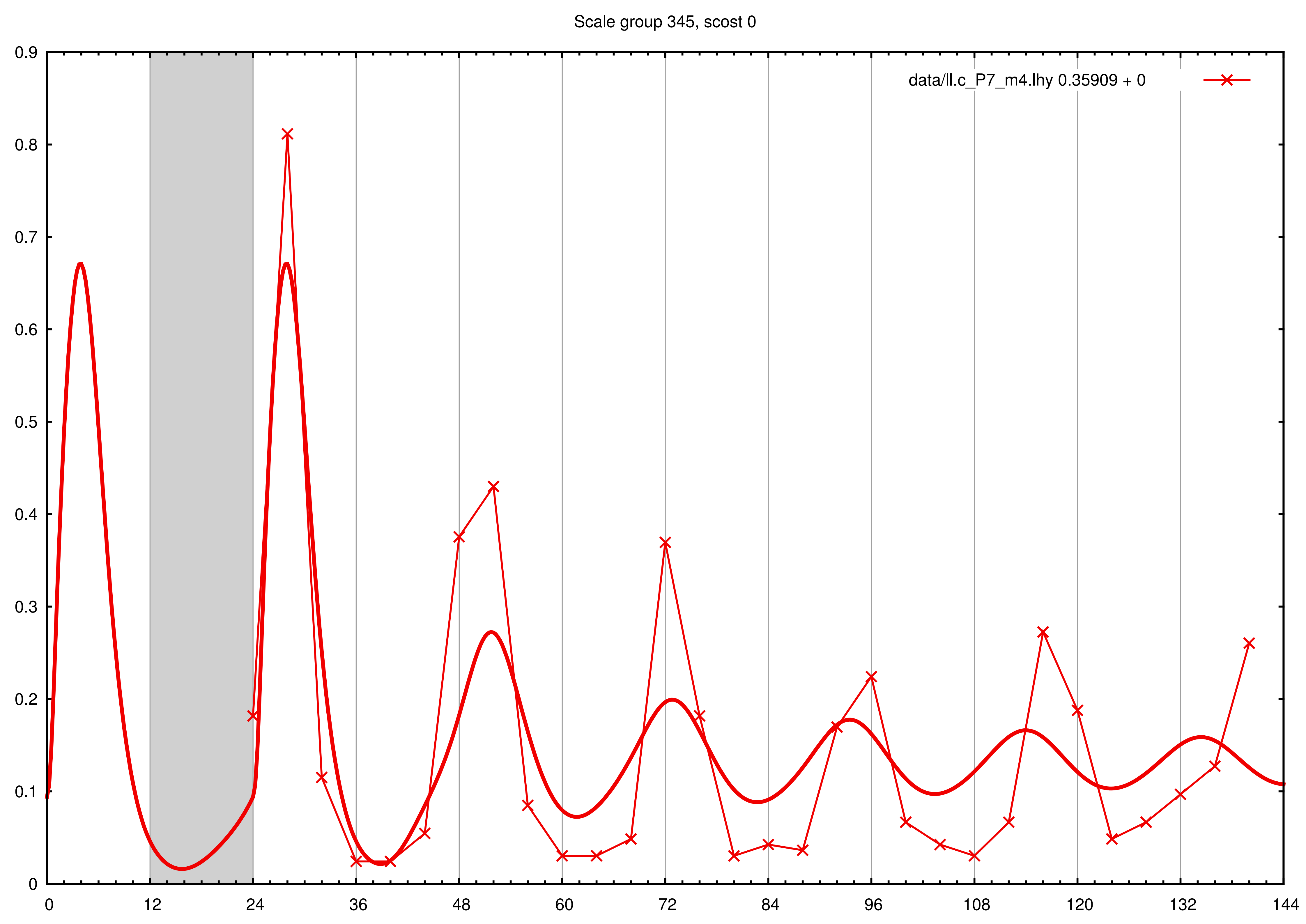

Scale group 345, scost 0
data/ll.c_P7_m4.lhy 0.35909 + 0
0.9
0.8
0.7
0.6
0.5
0.4
0.3
0.2
0.1
0
0
12
24
36
48
60
72
84
96
108
120
132
144

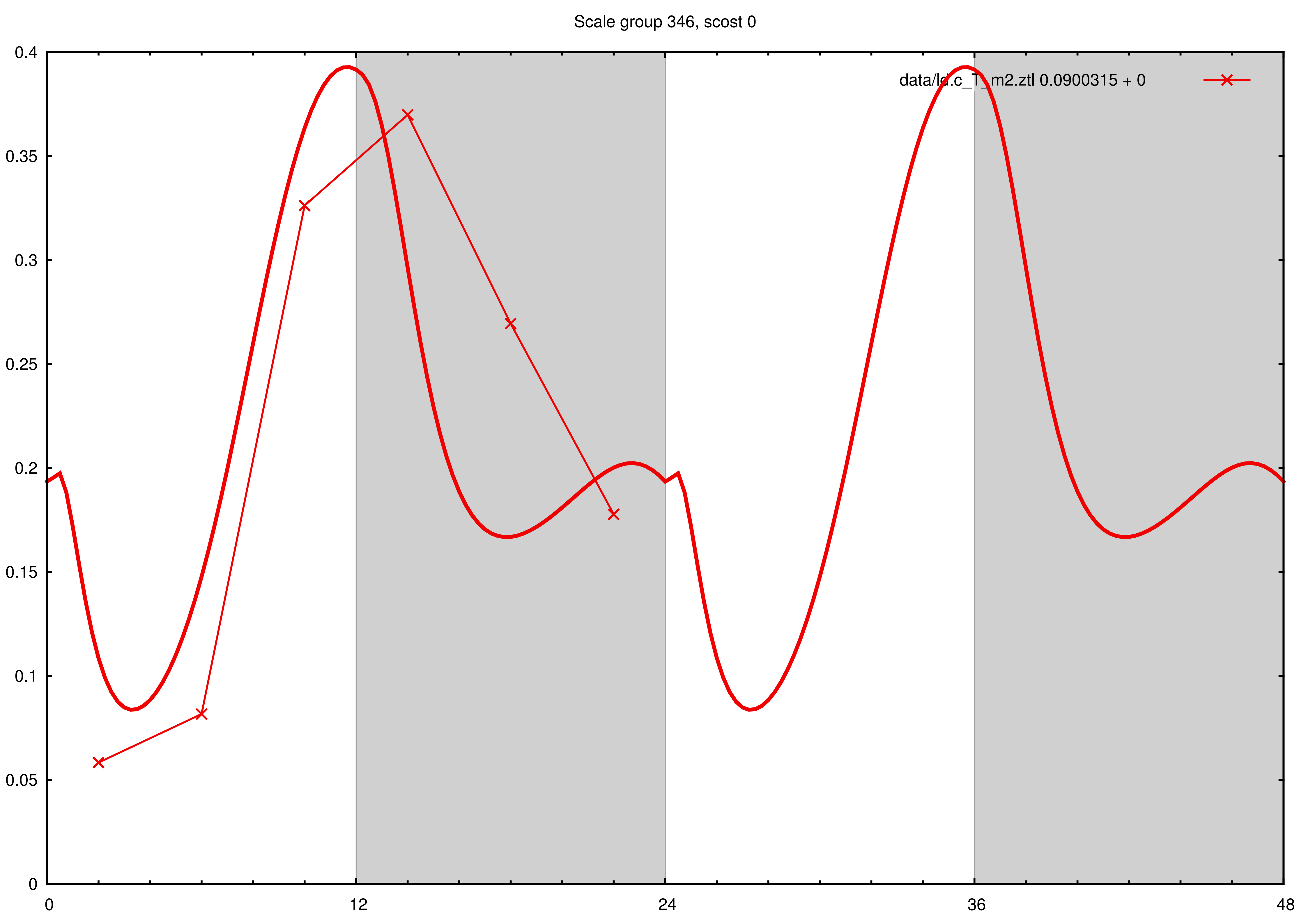
Scale group 346, scost 0
data/ld.c_T_m2.ztl 0.0900315 + 0
0.4
0.35
0.3
0.25
0.2
0.15
0.1
0.05
0
0
12
24
36
48

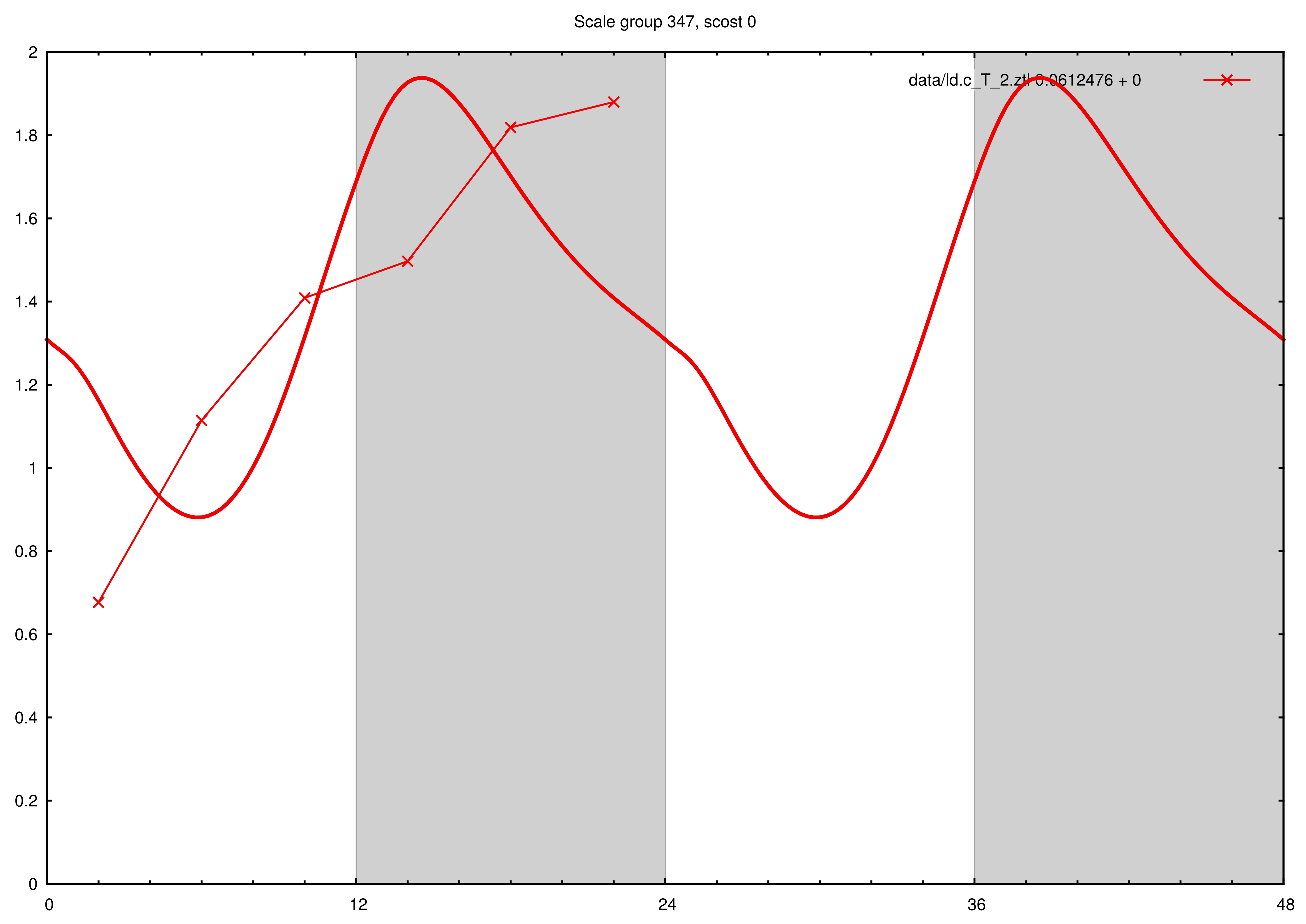
Scale group 347, scost 0
data/ld.c_T_2.ztl 0.0612476 + 0
0
0.2
0.4
0.6
0.8
1
1.2
1.4
1.6
1.8
2
0
12
24
36
48

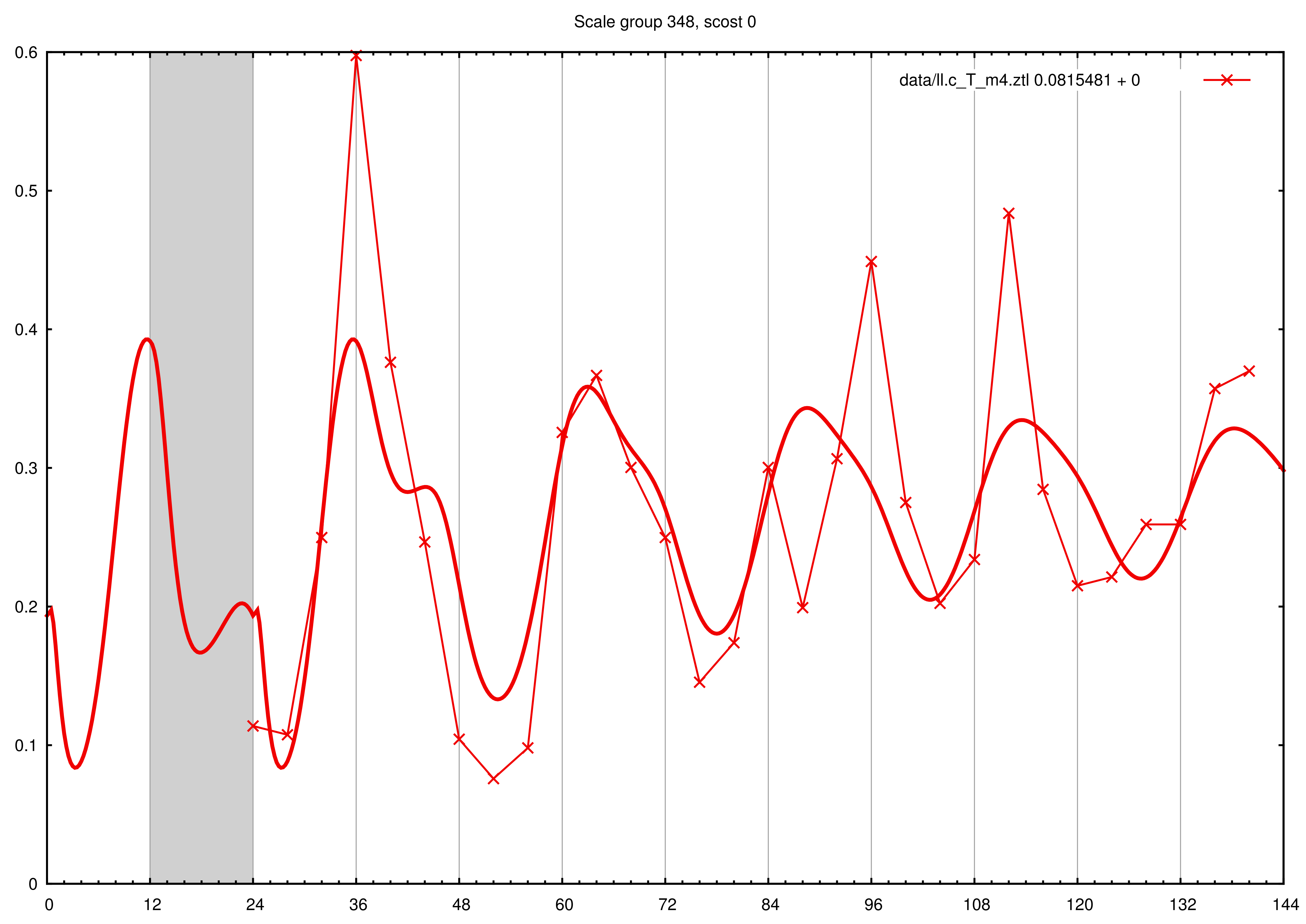

Scale group 348, scost 0
data/ll.c_T_m4.ztl 0.0815481 + 0
0.6
0.5
0.4
0.3
0.2
0.1
0
0
12
24
36
48
60
72
84
96
108
120
132
144

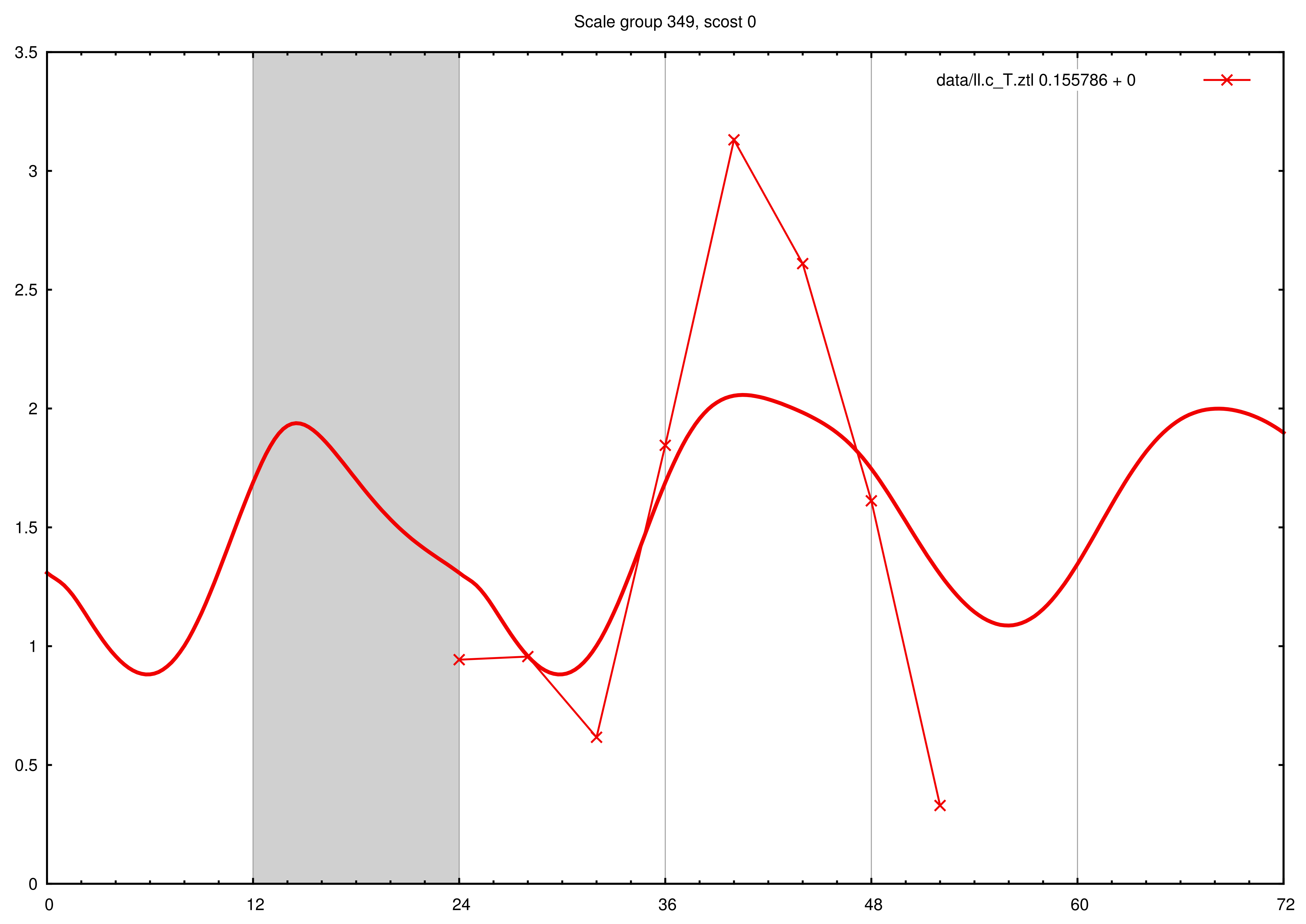

Scale group 349, scost 0
data/ll.c_T.ztl 0.155786 + 0
3.5
3
2.5
2
1.5
1
0.5
0
0
12
24
36
48
60
72

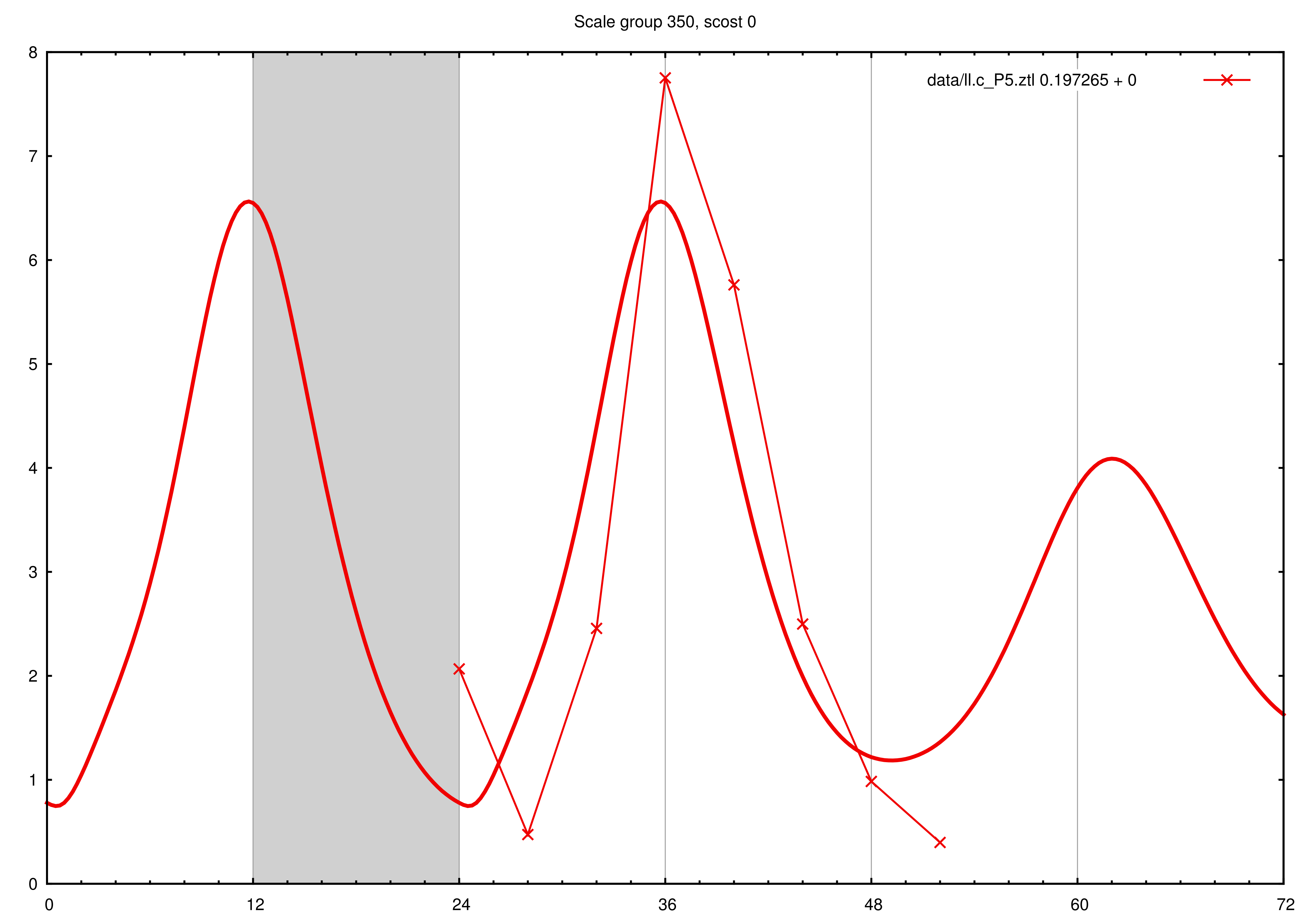
Scale group 350, scost 0
data/ll.c_P5.ztl 0.197265 + 0
0
1
2
3
4
5
6
7
8
0
12
24
36
48
60
72

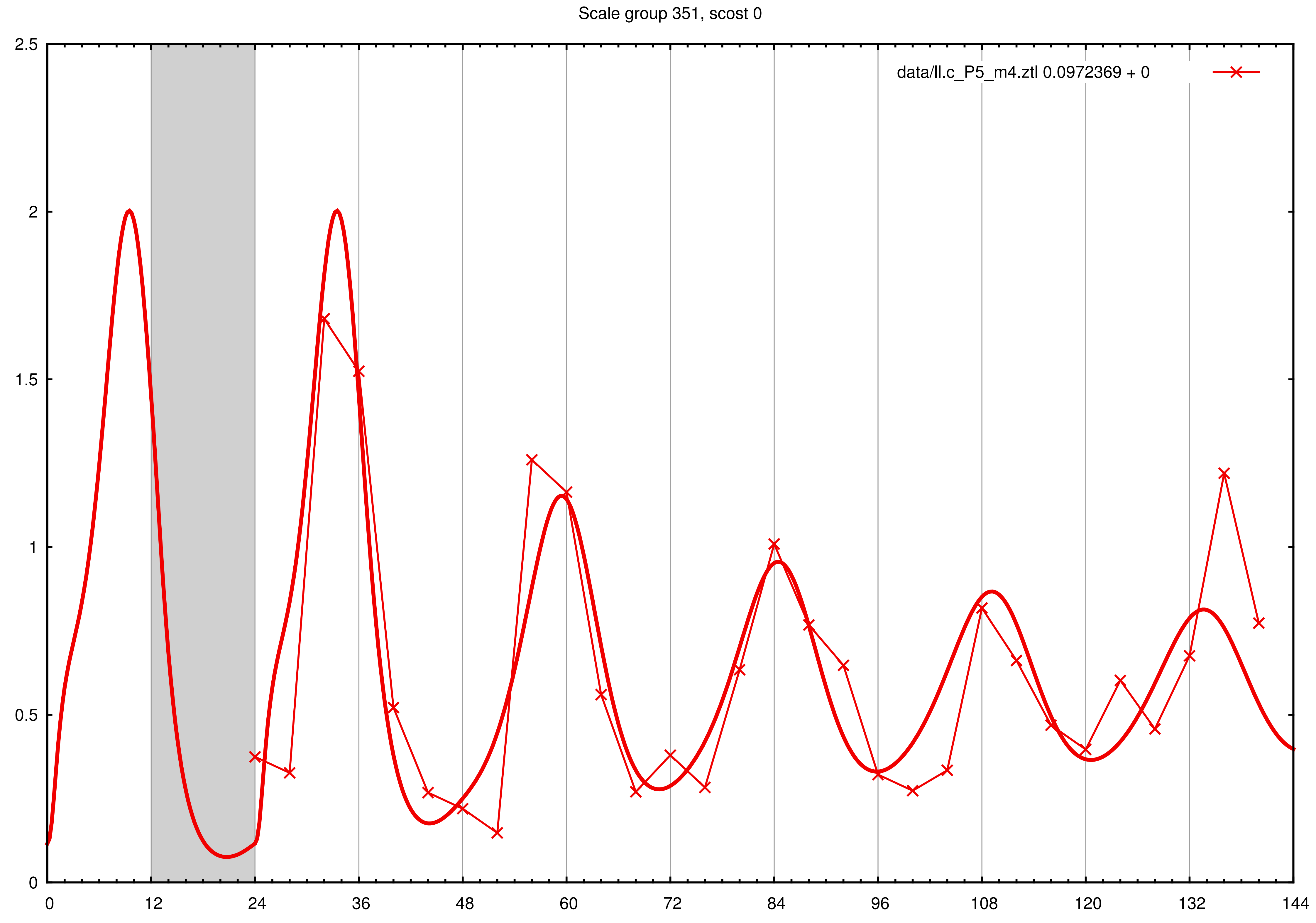

Scale group 351, scost 0
data/ll.c_P5_m4.ztl 0.0972369 + 0
0
0.5
1
1.5
2
2.5
0
12
24
36
48
60
72
84
96
108
120
132
144

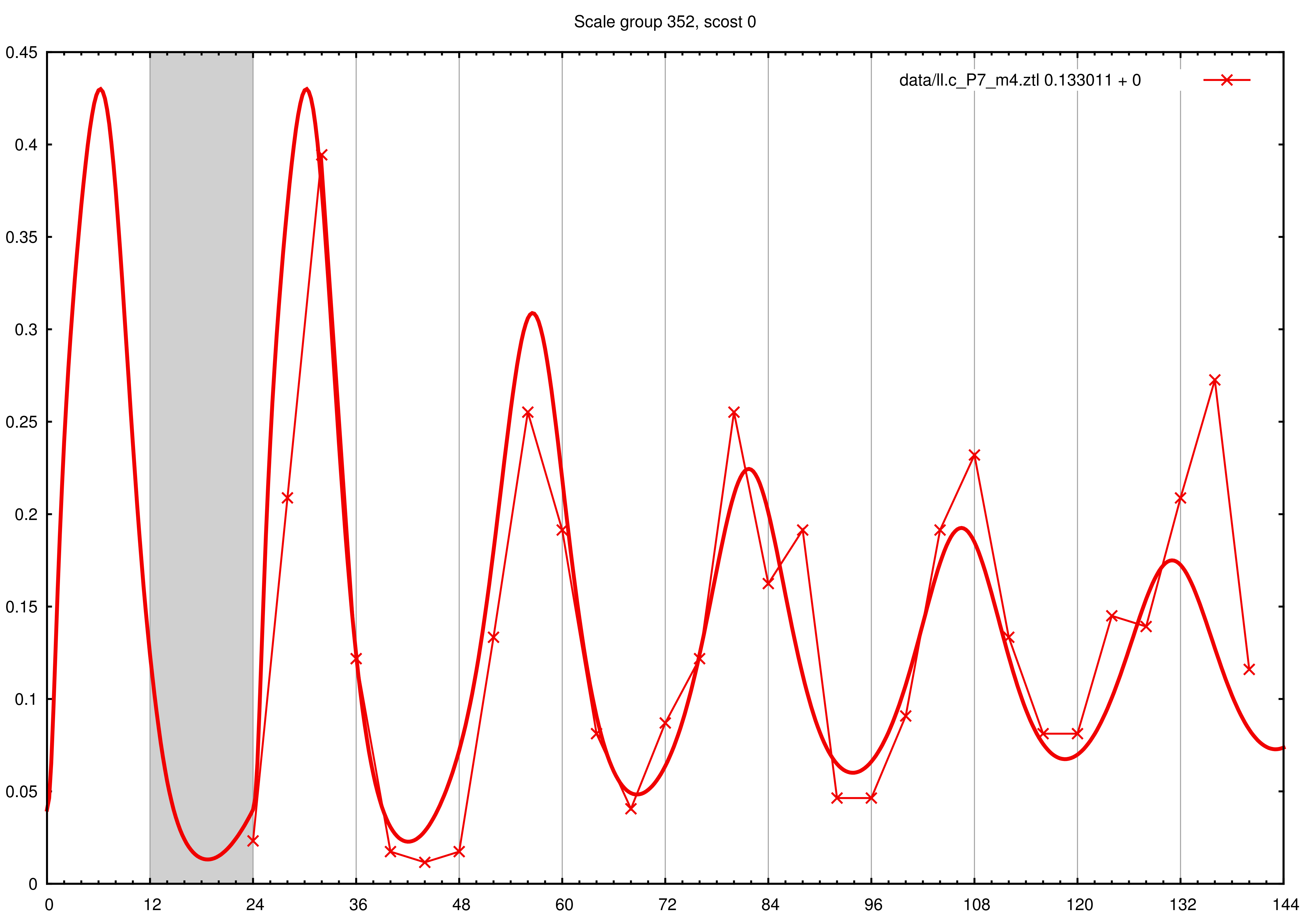
Scale group 352, scost 0
data/ll.c_P7_m4.ztl 0.133011 + 0
0.45
0.4
0.35
0.3
0.25
0.2
0.15
0.1
0.05
0
0
12
24
36
48
60
72
84
96
108
120
132
144

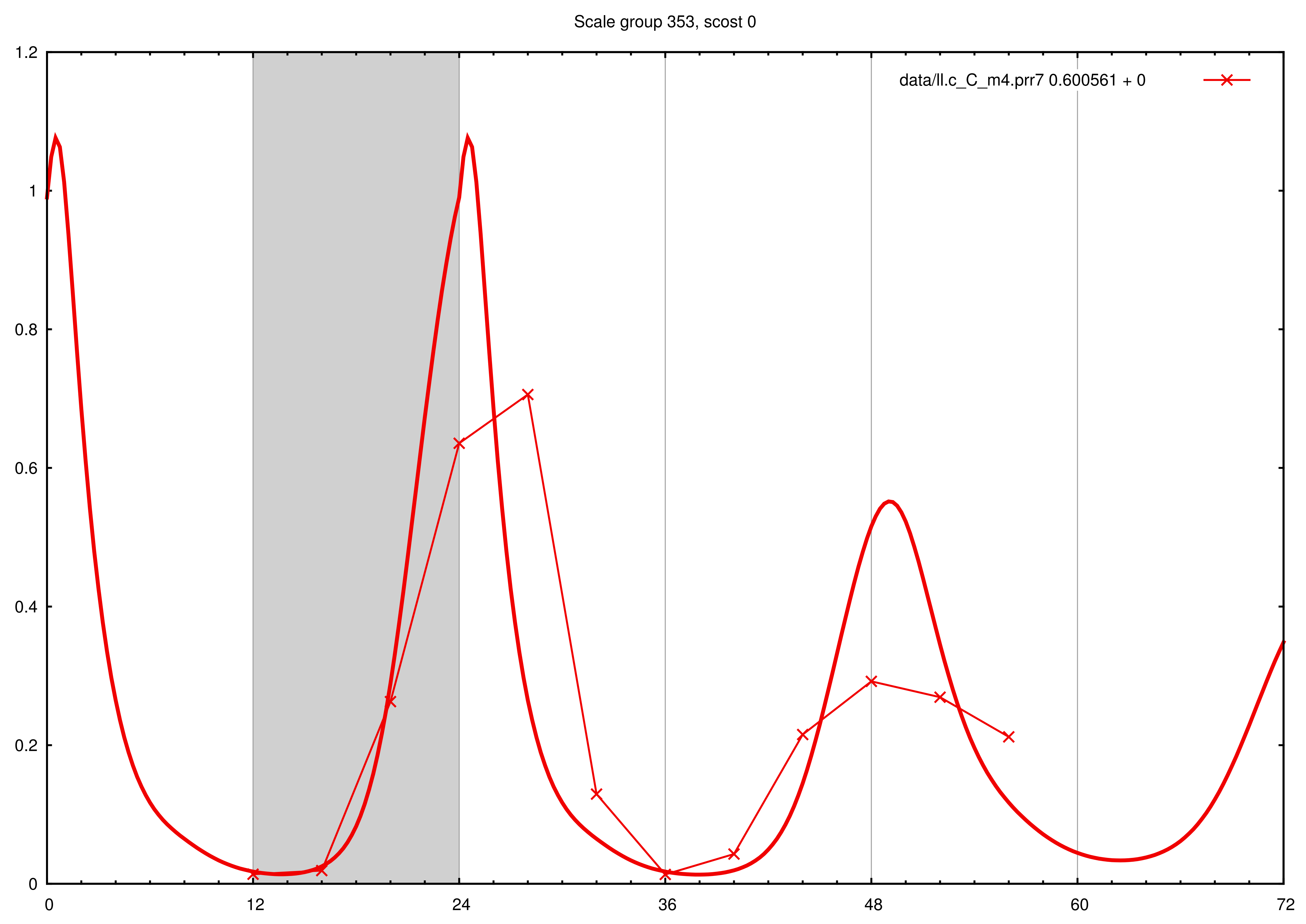

Scale group 353, scost 0
data/ll.c_C_m4.prr7 0.600561 + 0
1.2
1
0.8
0.6
0.4
0.2
0
0
12
24
36
48
60
72

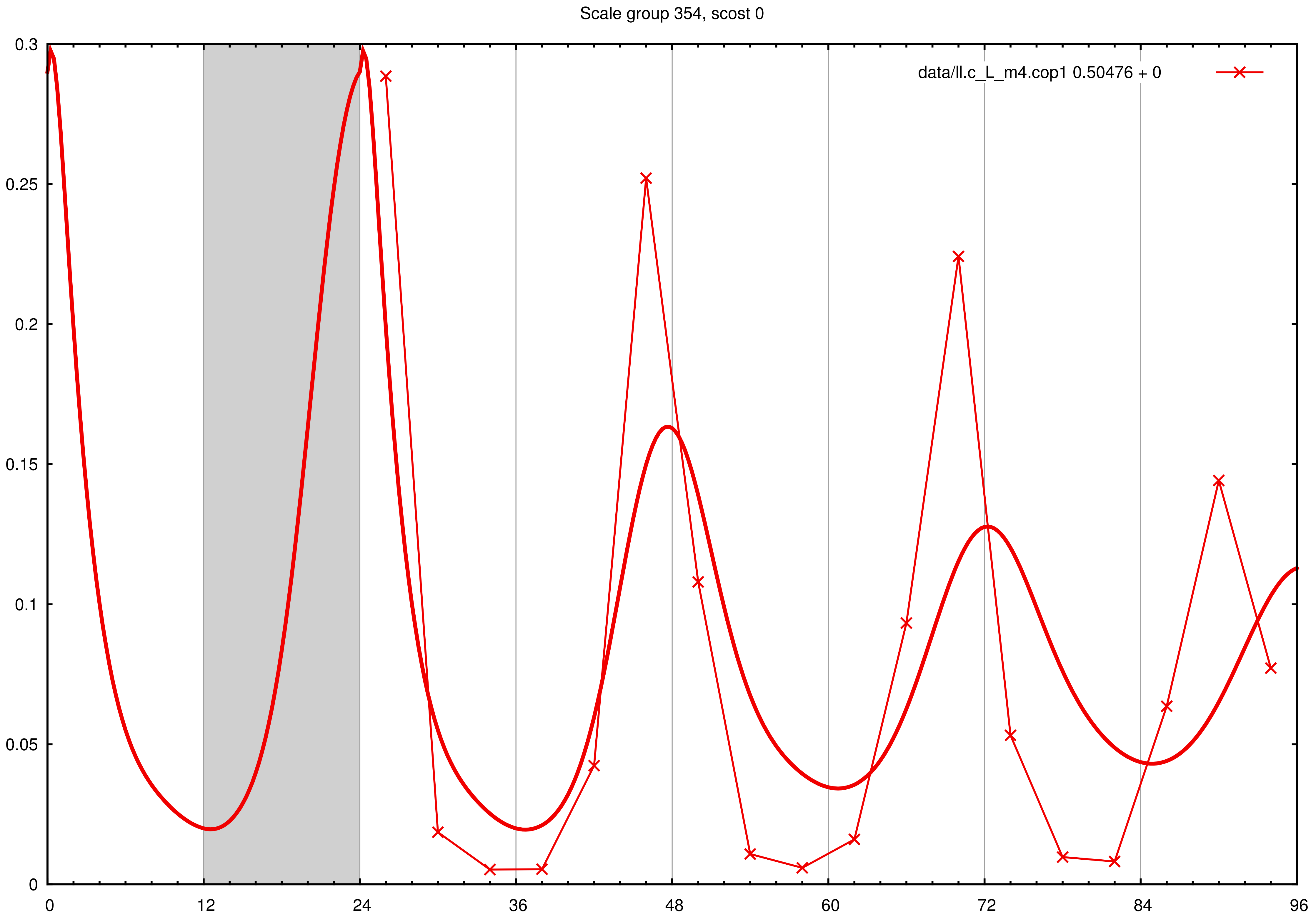

Scale group 354, scost 0
data/ll.c_L_m4.cop1 0.50476 + 0
0.3
0.25
0.2
0.15
0.1
0.05
0
0
12
24
36
48
60
72
84
96

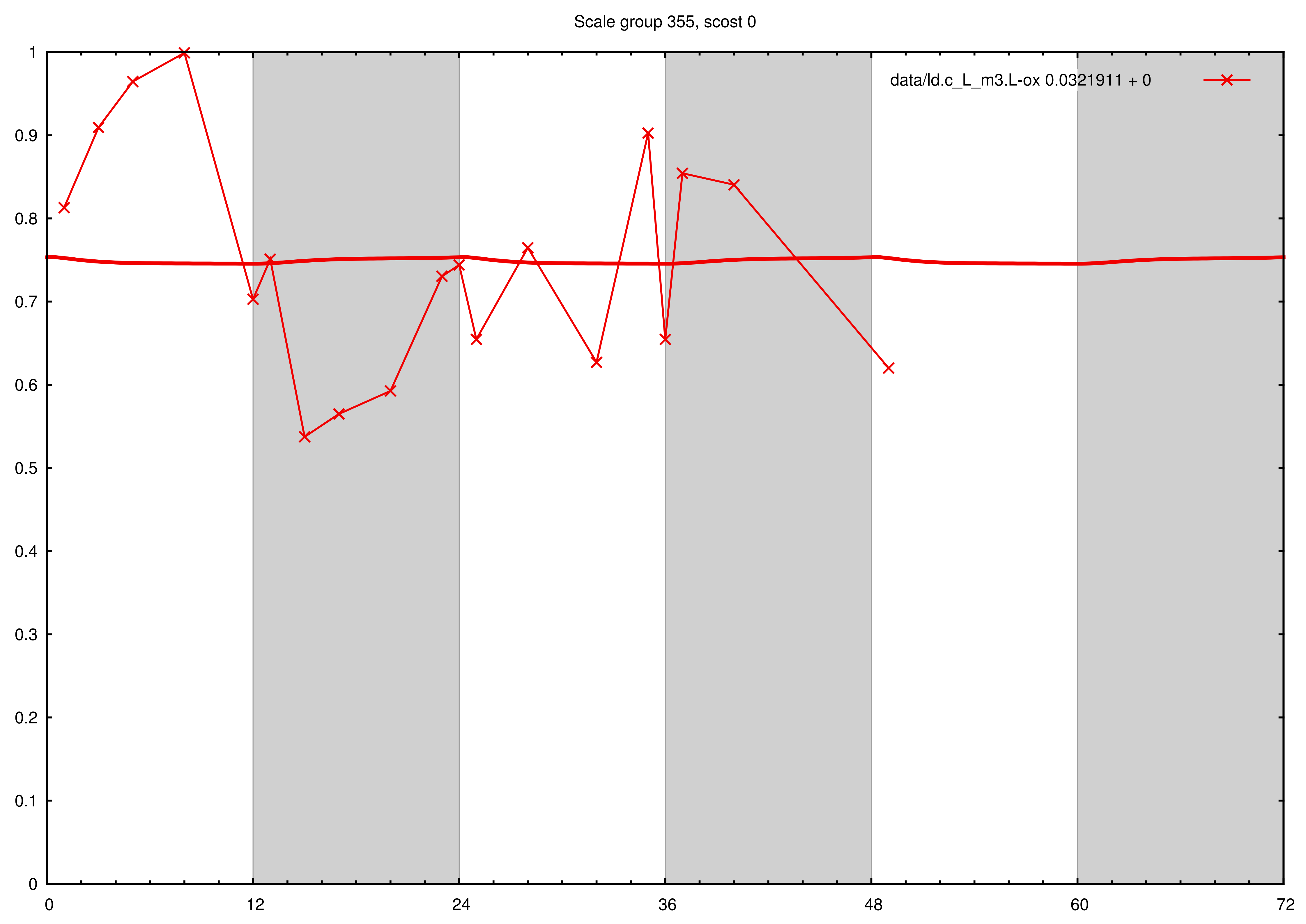

Scale group 355, scost 0
data/ld.c_L_m3.L-ox 0.0321911 + 0
0
0.1
0.2
0.3
0.4
0.5
0.6
0.7
0.8
0.9
1
0
12
24
36
48
60
72

Scale group 356, scost 0

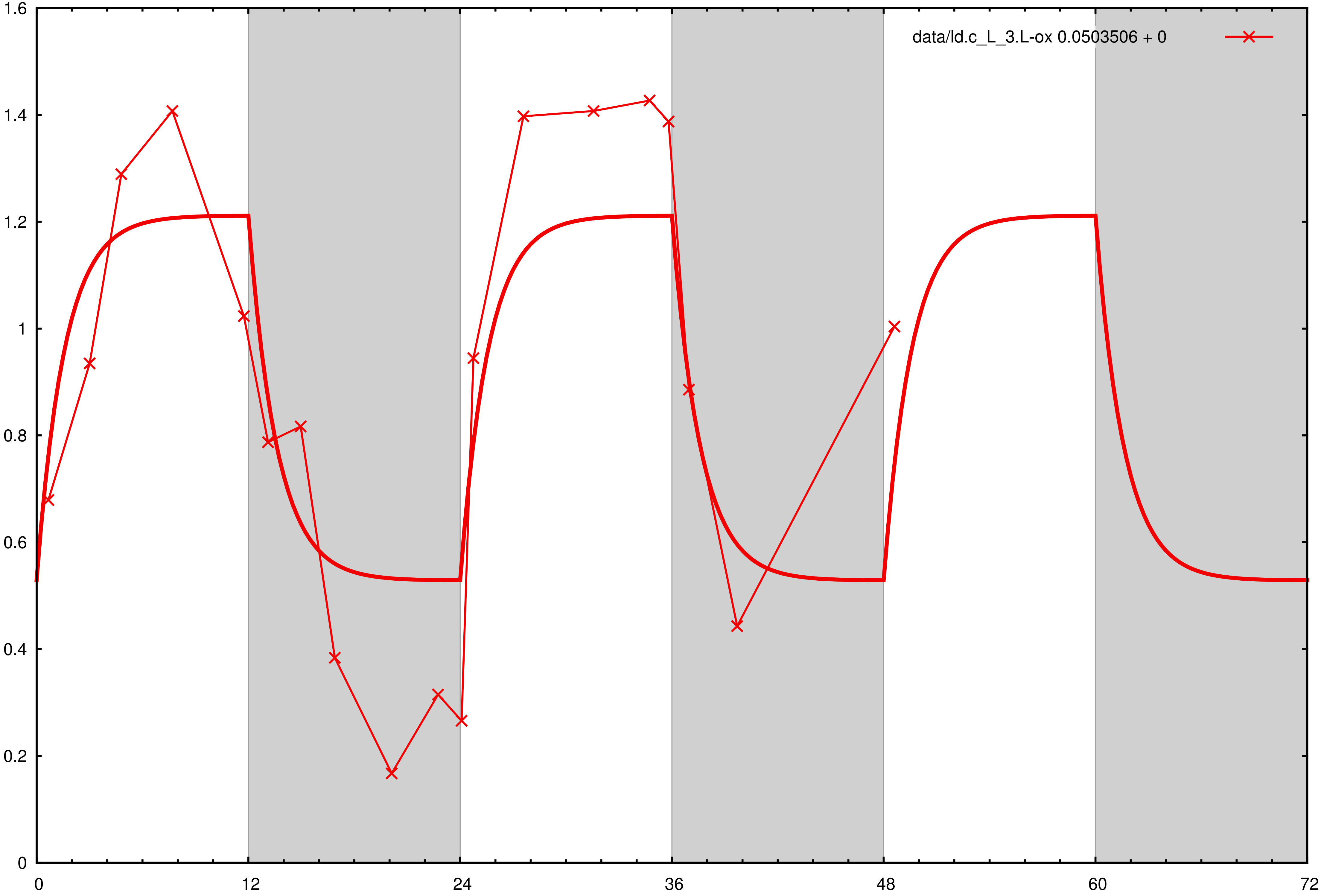

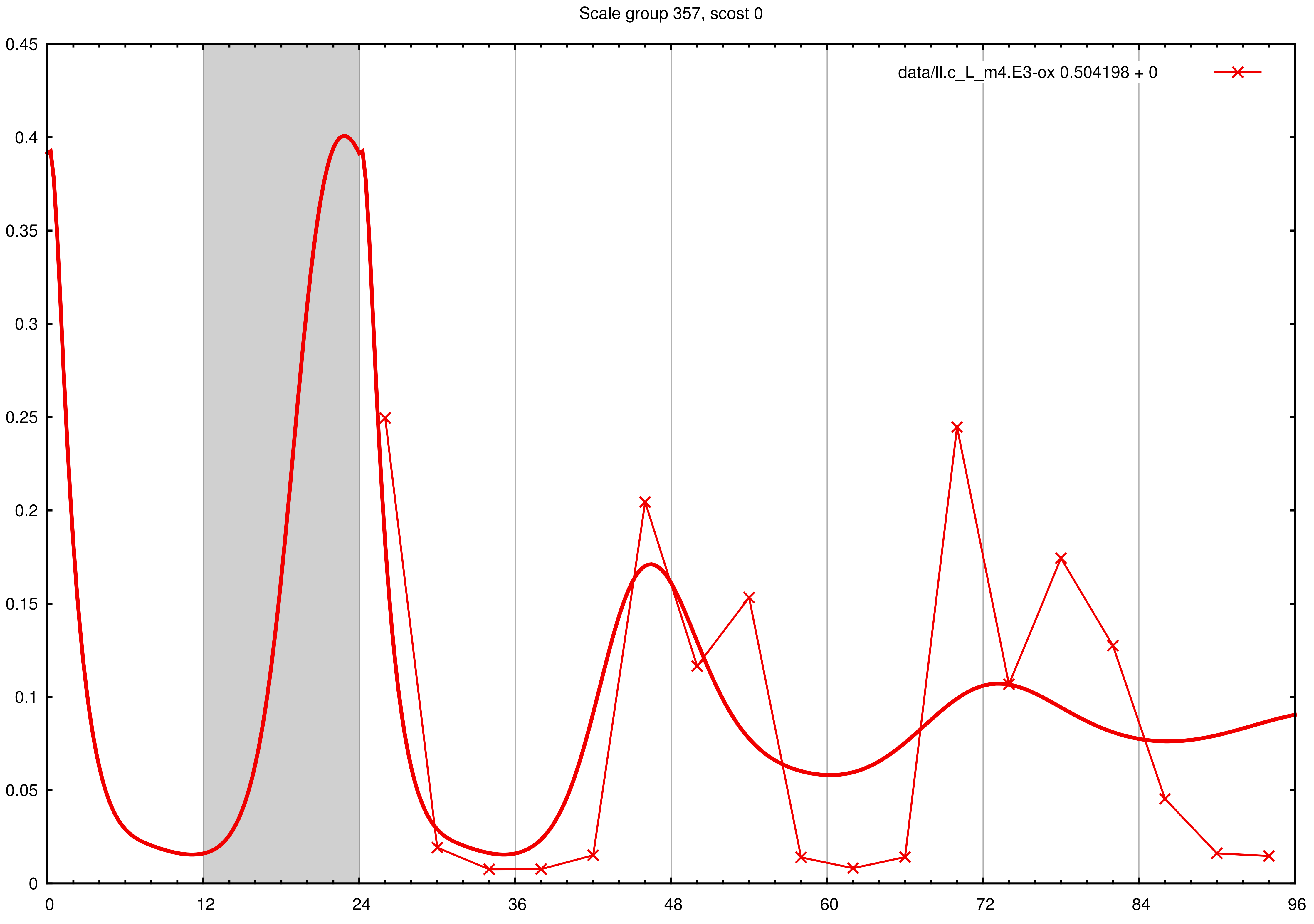

Scale group 357, scost 0
data/ll.c_L_m4.E3-ox 0.504198 + 0
0.45
0.4
0.35
0.3
0.25
0.2
0.15
0.1
0.05
0
0
12
24
36
48
60
72
84
96

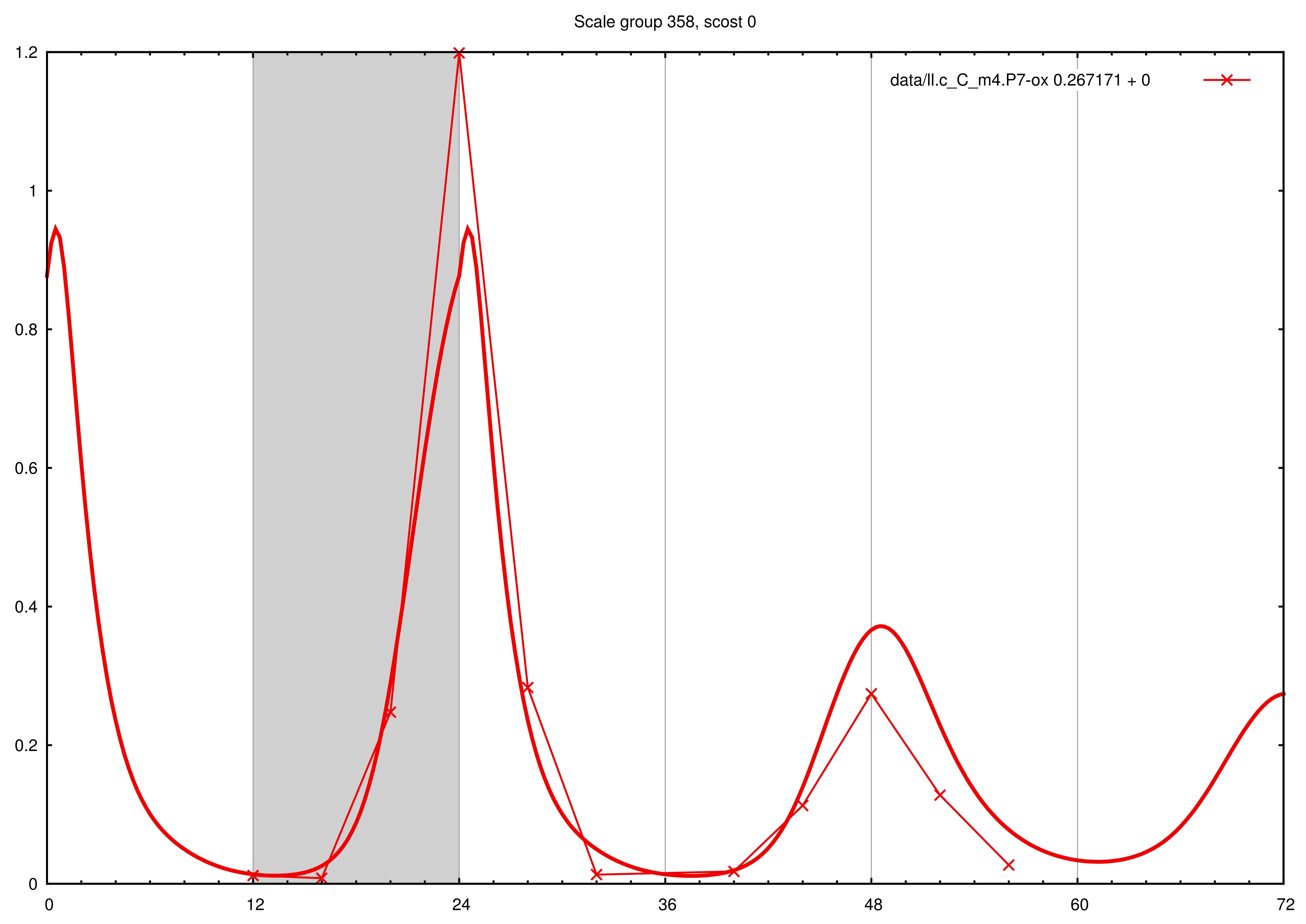

Scale group 358, scost 0
data/ll.c_C_m4.P7-ox 0.267171 + 0
1.2
1
0.8
0.6
0.4
0.2
0
0
12
24
36
48
60
72

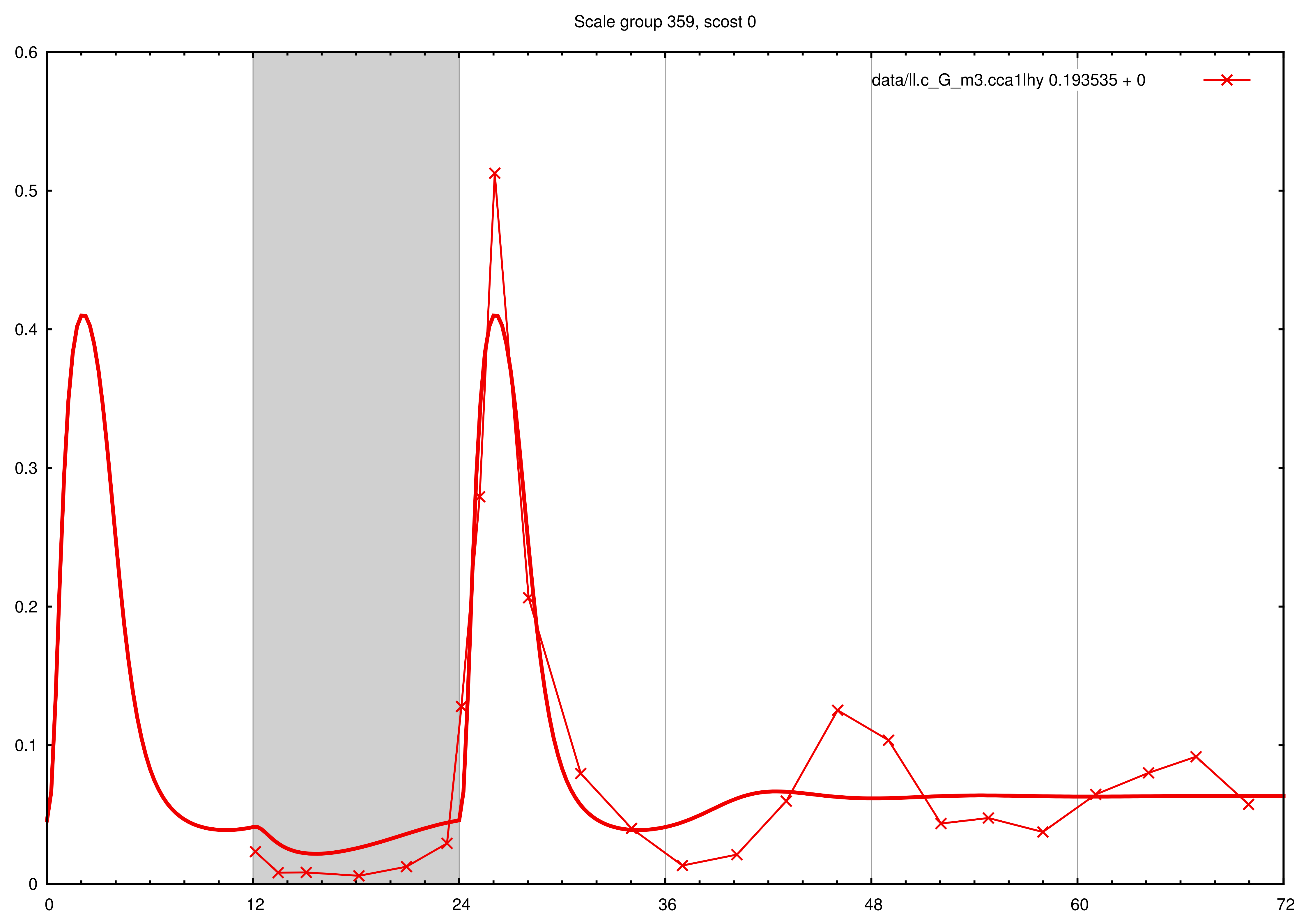

Scale group 359, scost 0
data/ll.c_G_m3.cca1lhy 0.193535 + 0
0.6
0.5
0.4
0.3
0.2
0.1
0
0
12
24
36
48
60
72

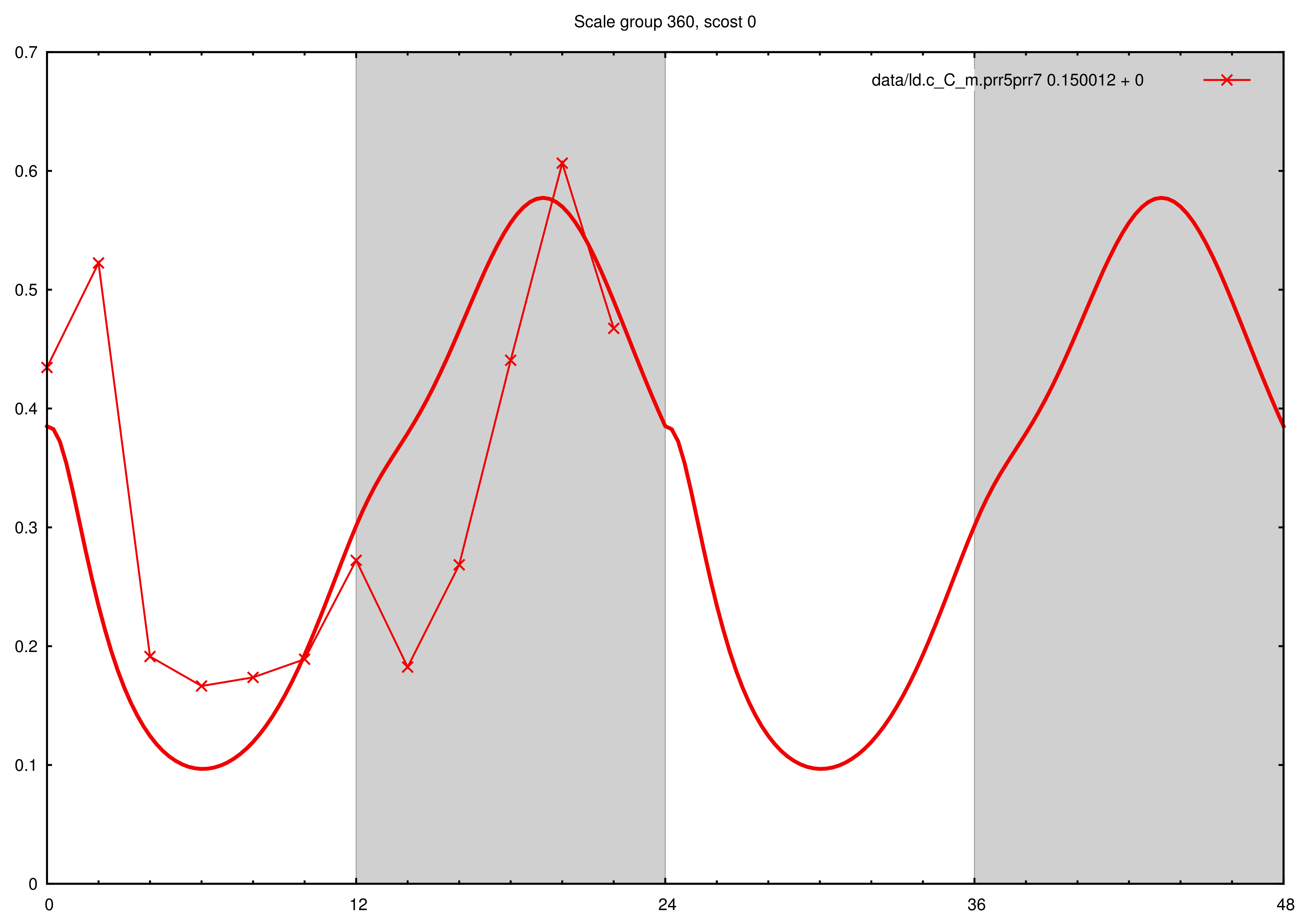

Scale group 360, scost 0
data/ld.c_C_m.prr5prr7 0.150012 + 0
0.7
0.6
0.5
0.4
0.3
0.2
0.1
0
0
12
24
36
48

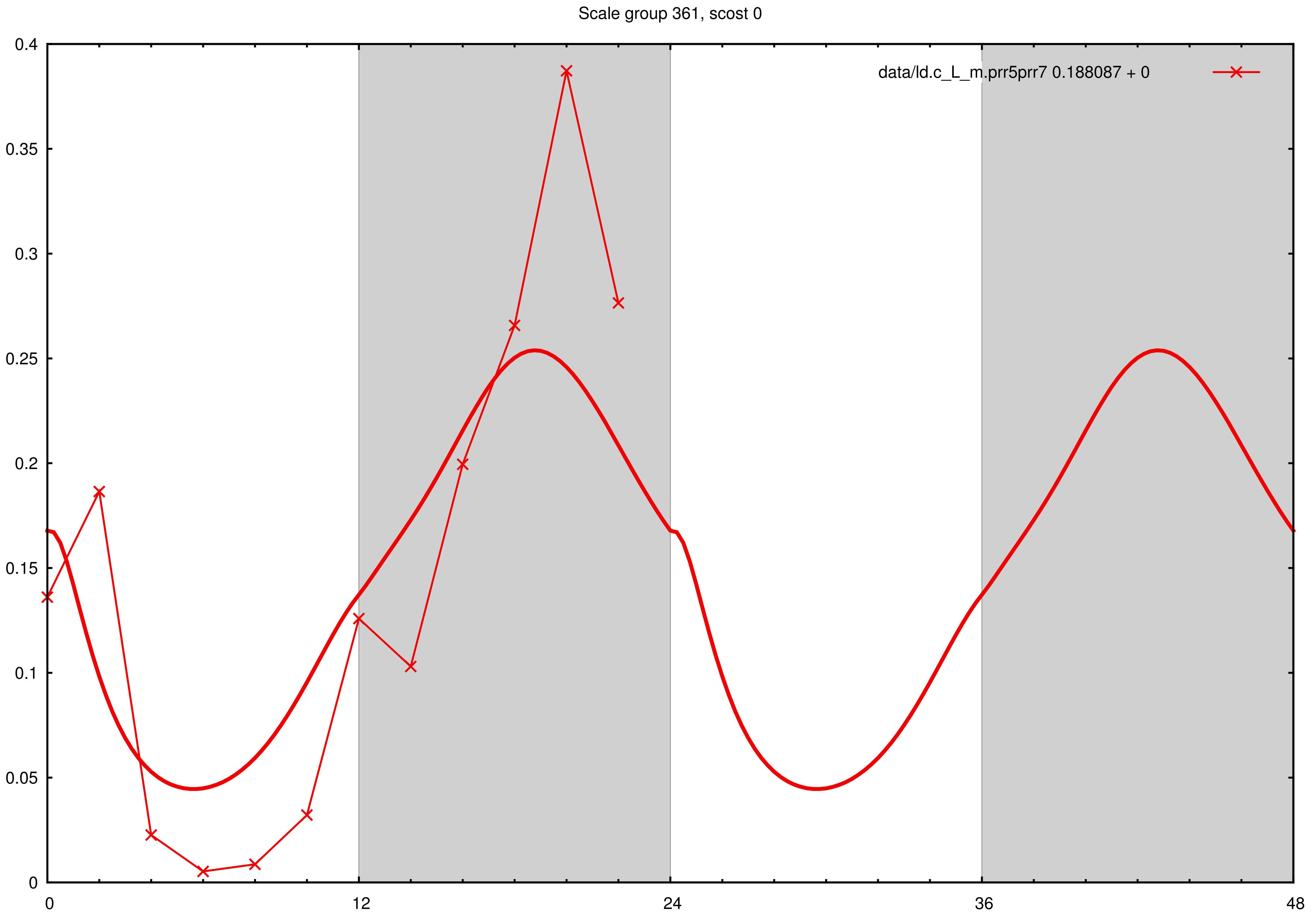

Scale group 361, scost 0
data/ld.c_L_m.prr5prr7 0.188087 + 0
0.4
0.35
0.3
0.25
0.2
0.15
0.1
0.05
0
0
12
24
36
48

Scale group 362, scost 0

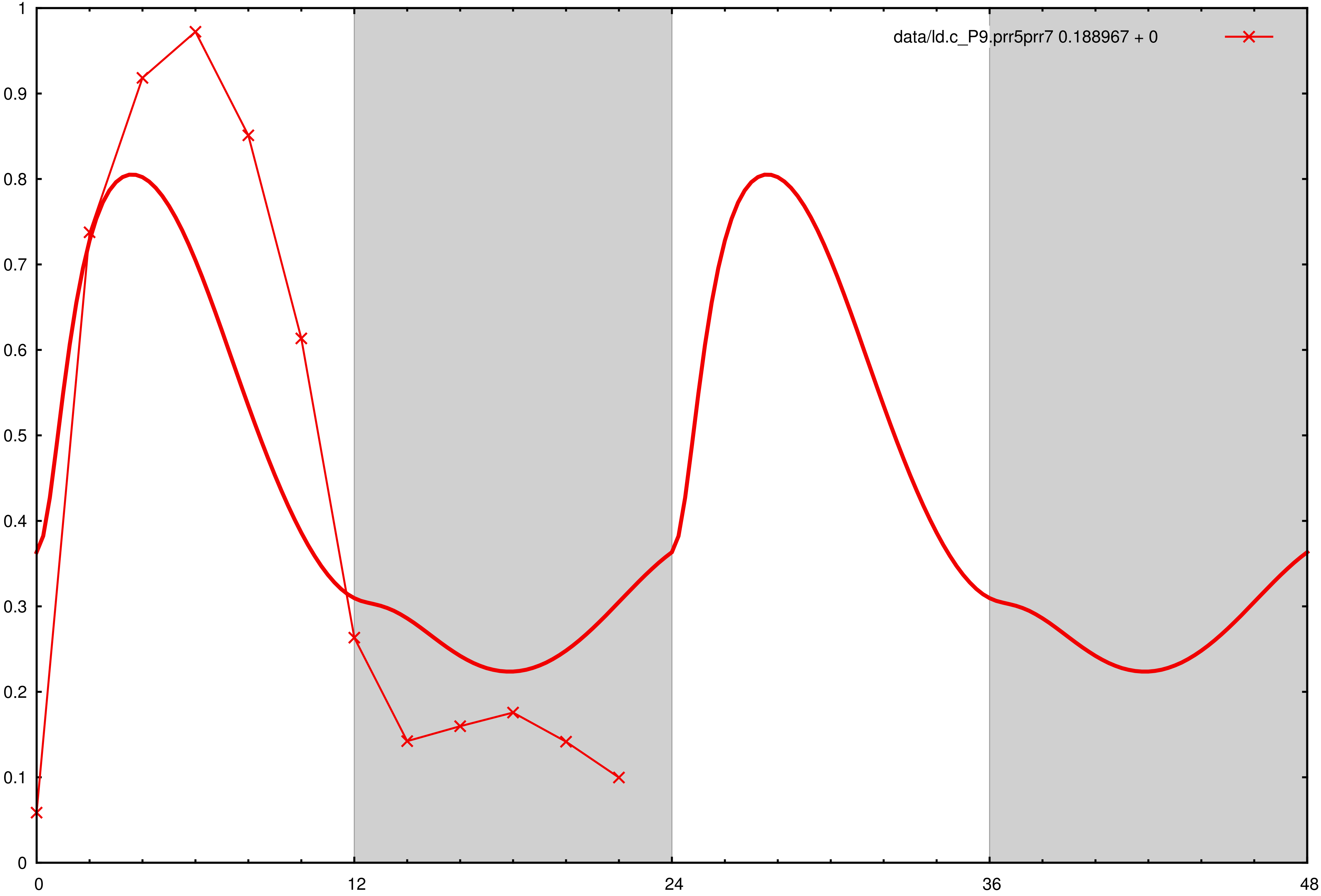

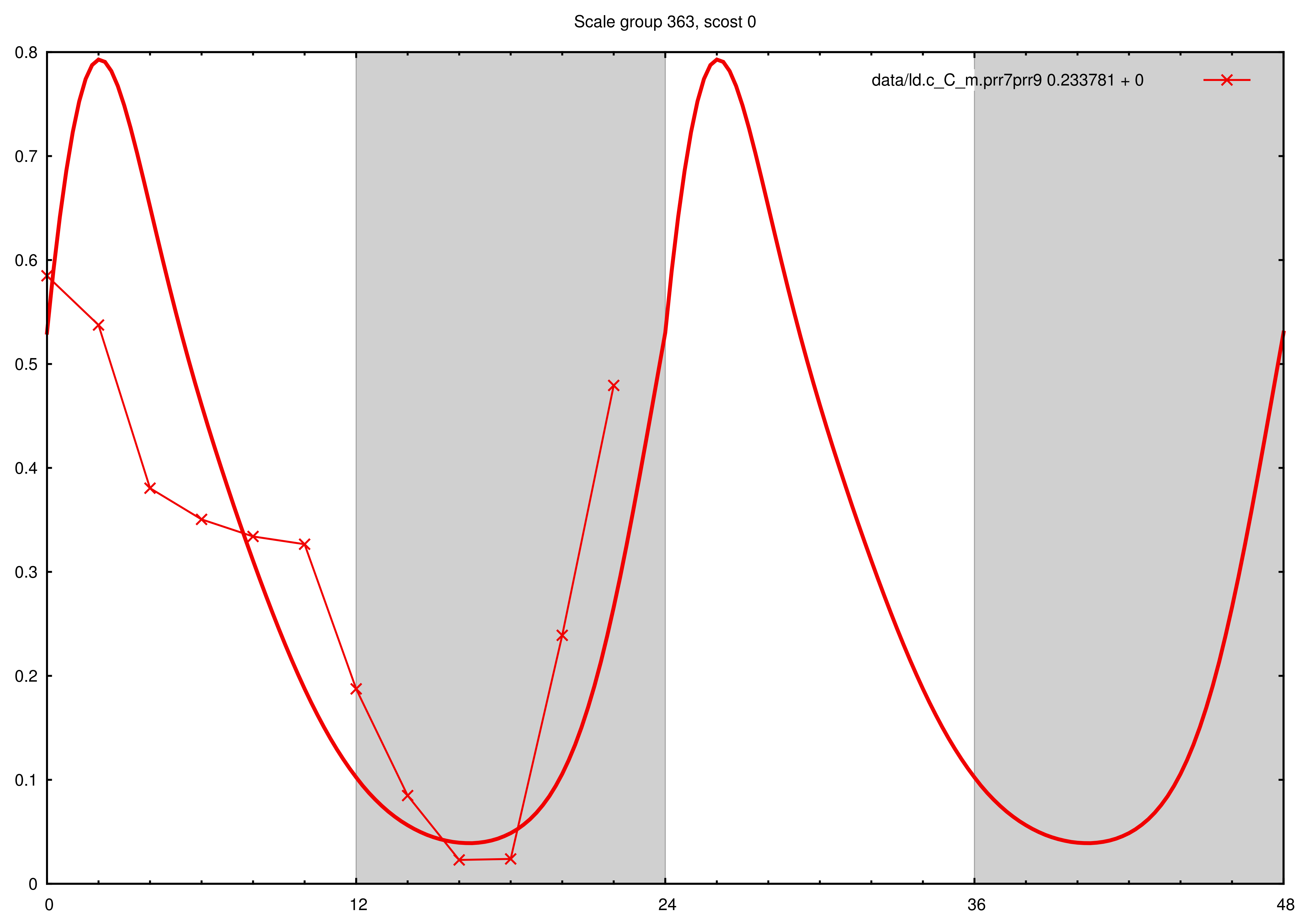
Scale group 363, scost 0
data/ld.c_C_m.prr7prr9 0.233781 + 0
0.8
0.7
0.6
0.5
0.4
0.3
0.2
0.1
0
0
12
24
36
48

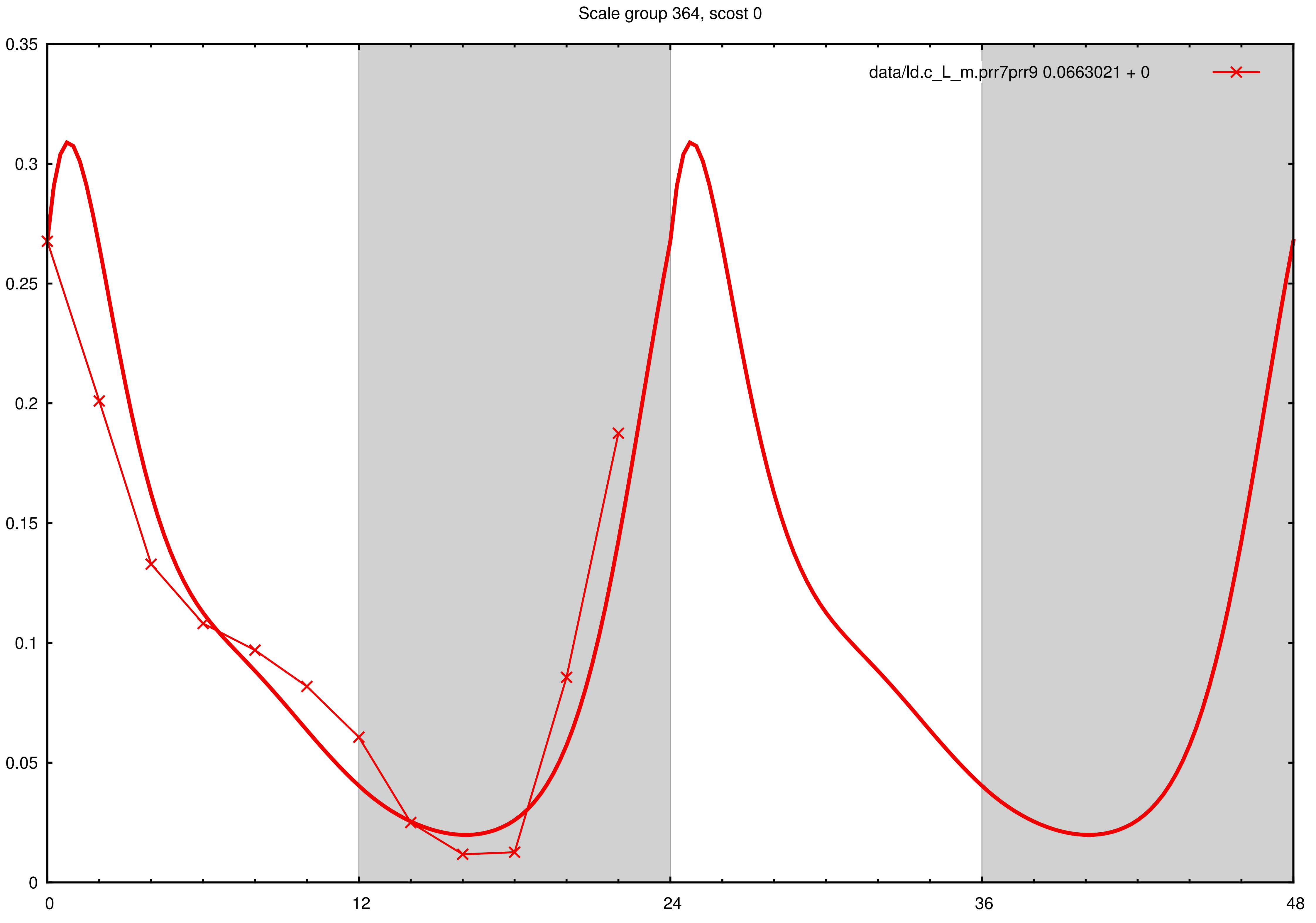

Scale group 364, scost 0
data/ld.c_L_m.prr7prr9 0.0663021 + 0
0.35
0.3
0.25
0.2
0.15
0.1
0.05
0
0
12
24
36
48

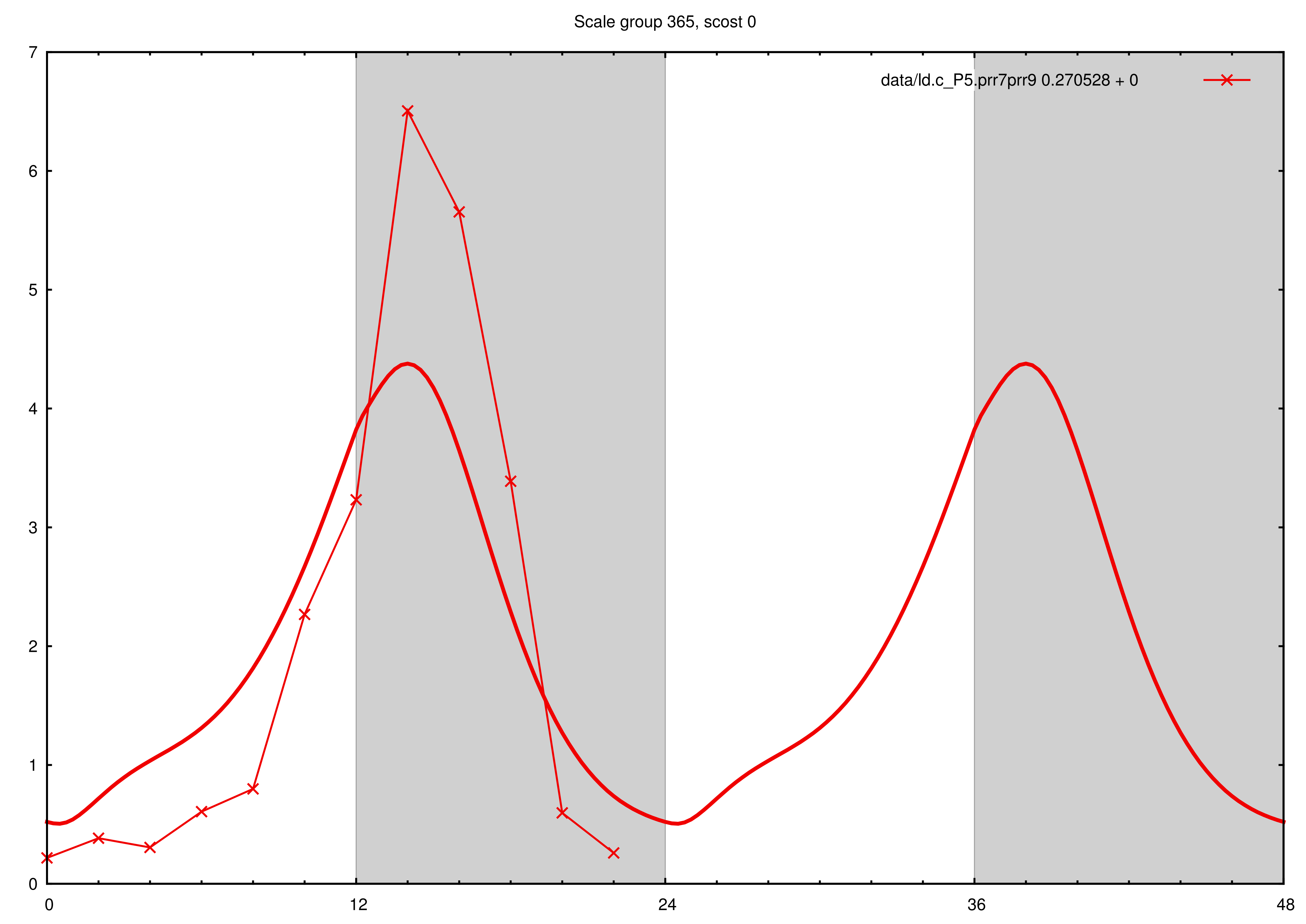

Scale group 365, scost 0
data/ld.c_P5.prr7prr9 0.270528 + 0
0
1
2
3
4
5
6
7
0
12
24
36
48

Scale group 366, scost 0

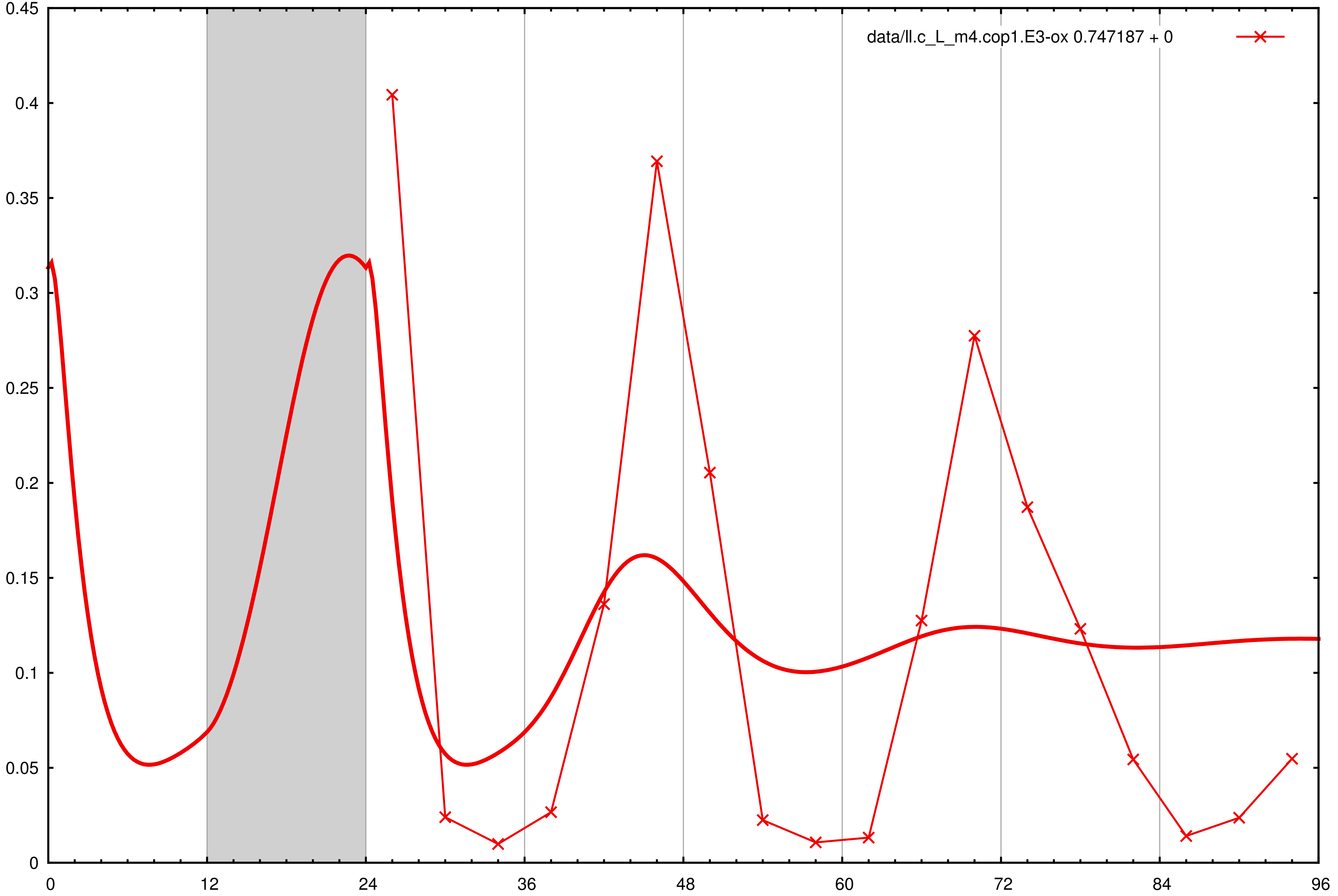

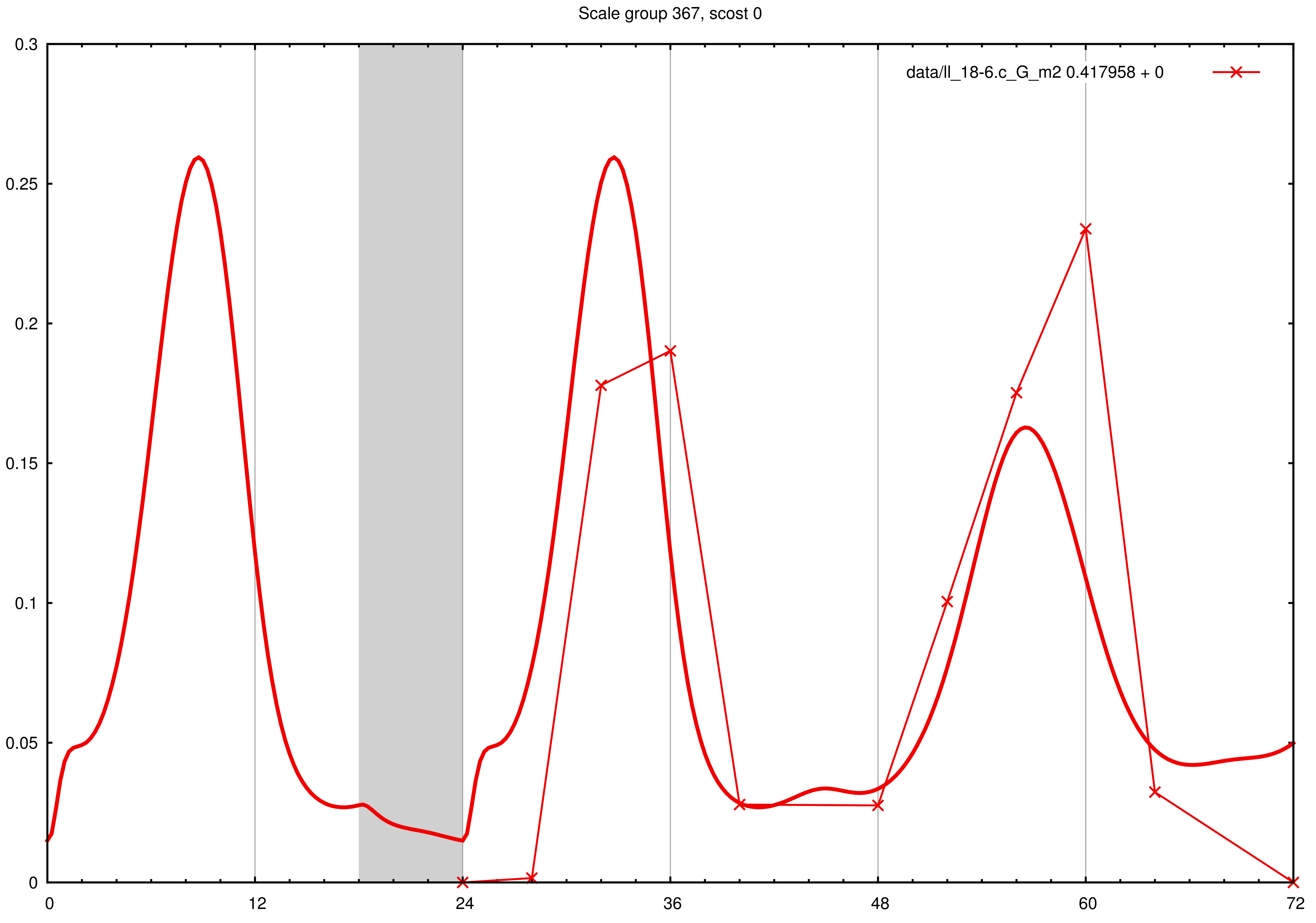

Scale group 367, scost 0
data/ll_18-6.c_G_m2 0.417958 + 0
0.3
0.25
0.2
0.15
0.1
0.05
0
0
12
24
36
48
60
72

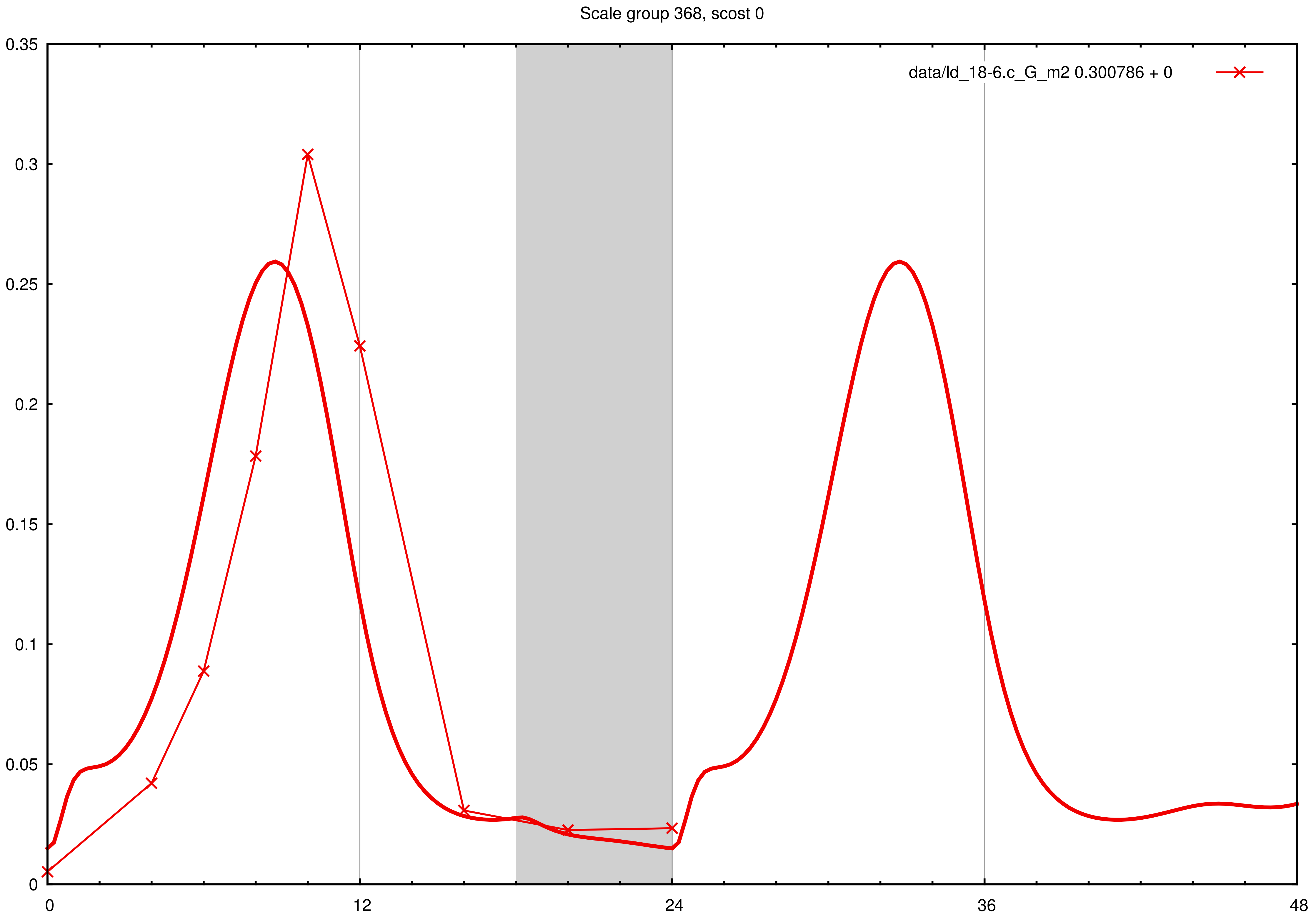
Scale group 368, scost 0
data/ld_18-6.c_G_m2 0.300786 + 0
0.35
0.3
0.25
0.2
0.15
0.1
0.05
0
0
12
24
36
48

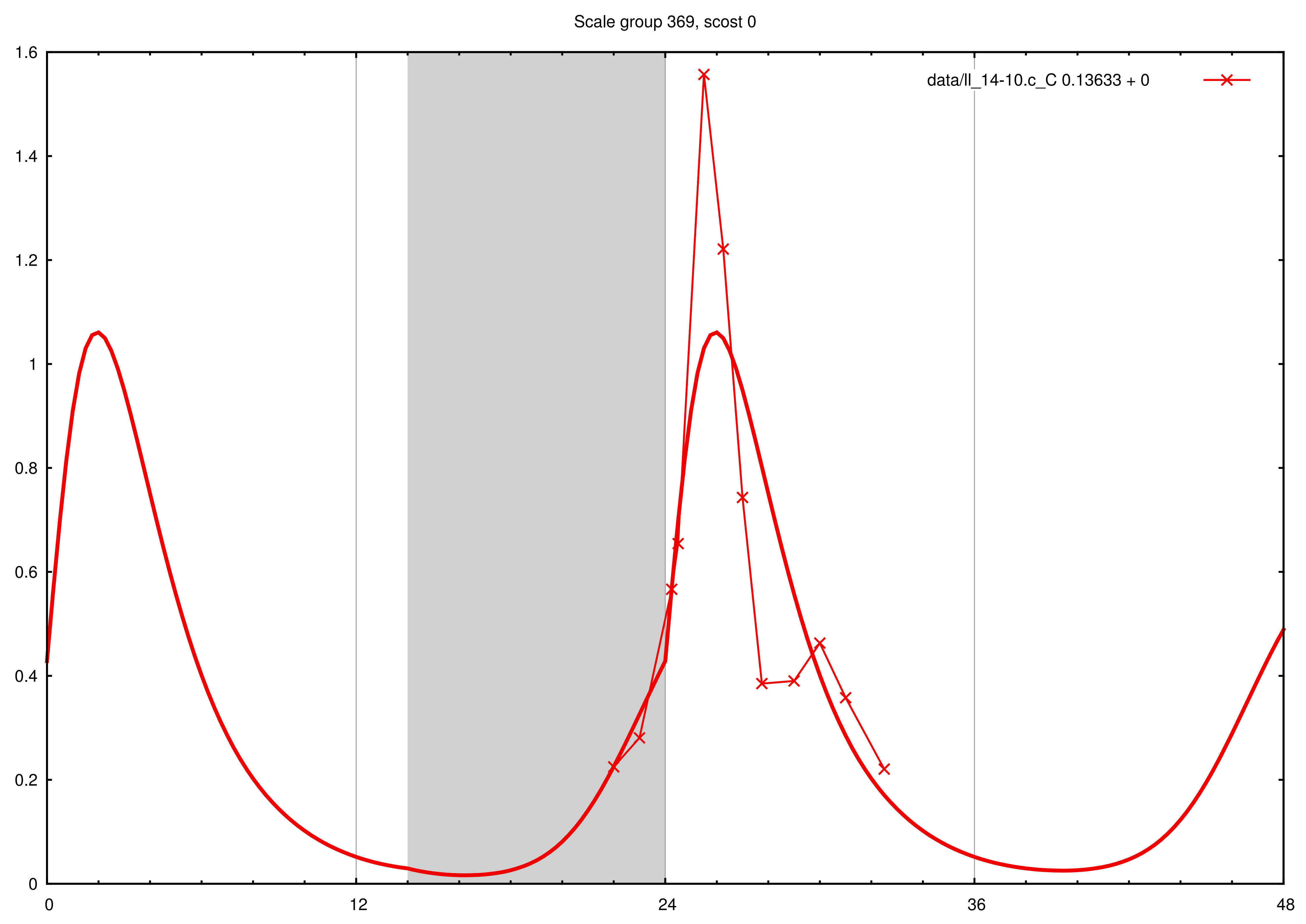
Scale group 369, scost 0
data/ll_14-10.c_C 0.13633 + 0
1.6
1.4
1.2
1
0.8
0.6
0.4
0.2
0
0
12
24
36
48

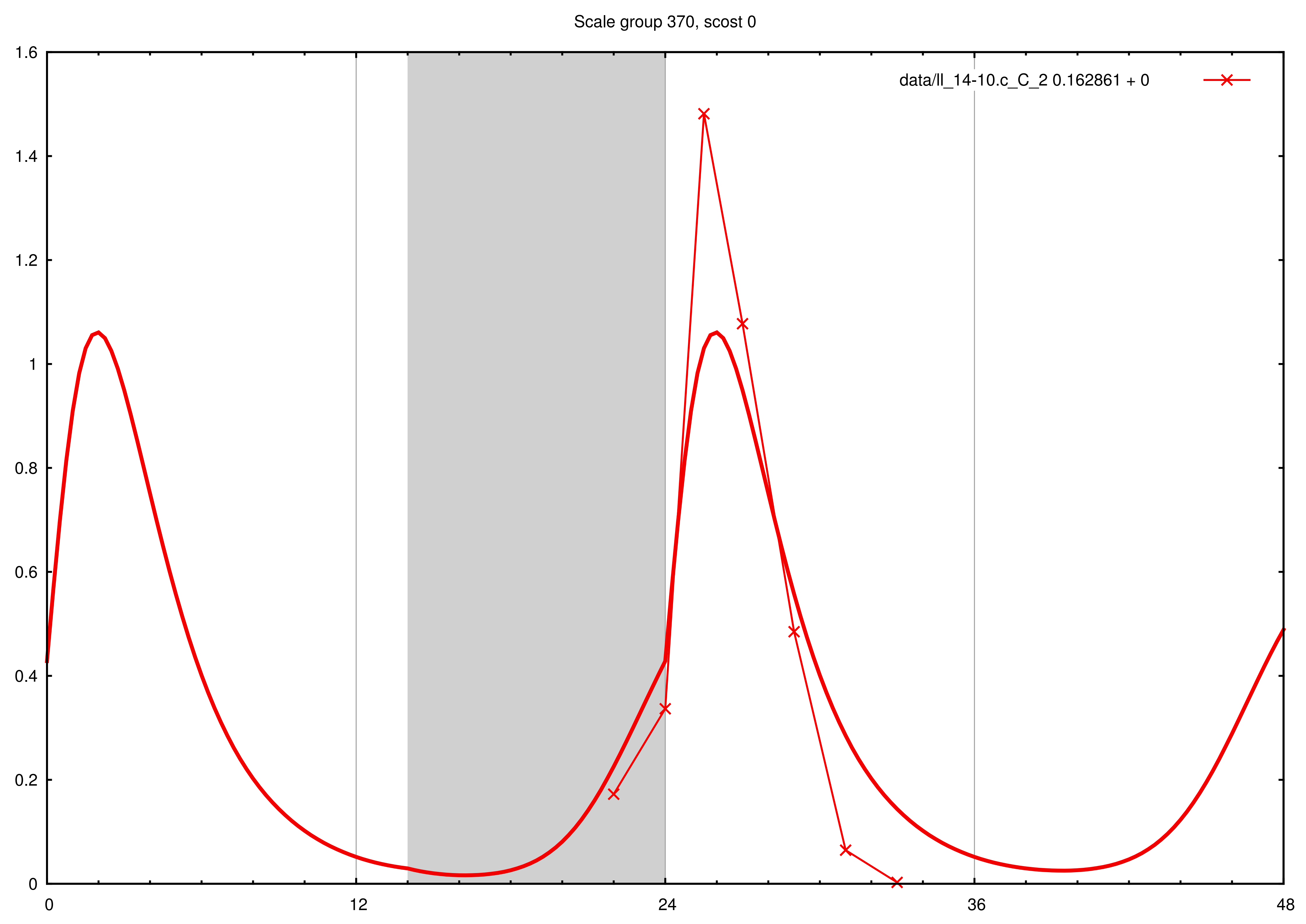
Scale group 370, scost 0
data/ll_14-10.c_C_2 0.162861 + 0
1.6
1.4
1.2
1
0.8
0.6
0.4
0.2
0
0
12
24
36
48
