# Supplementary material for: Rethinking Transcriptional Activation in the Arabidopsis Circadian Clock
Source: PLoS Comput Biol. 2014 Jul 17;10(7):e1003705. doi: 10.1371/journal.pcbi.1003705 (PMC4102396; doi:10.1371/journal.pcbi.1003705)
Supplement: Text S2 — Overview of the compiled time course data. A list of the roughly 800 experimental data sets that were compiled and used for fitting the model. (PDF) [file pcbi.1003705.s010.pdf]

Ref.	Fig.	Reporter	Light	Mutant / Comment	Weight
[1] Alabadi, 2001	1A	*TOC1* mRNA	LL 12:12	wt, Ler	
[1] Alabadi, 2001	1A	*TOC1* mRNA	LL 12:12	lhy (lhy^{TN104})	
[1] Alabadi, 2001	1B	*TOC1* mRNA	LL 12:12	CCA1-ox	
[1] Alabadi, 2001	1B	*TOC1* mRNA	LL 12:12	wt, Col	
[1] Alabadi, 2001	1C	*TOC1* mRNA	LL 12:12	elf3-1	
[1] Alabadi, 2001	1C	*TOC1* mRNA	LL 12:12	wt, Col	
[2] Baudry, 2010	3D	*CCA1* mRNA	LL 12:12	ztl-4	
[2] Baudry, 2010	3D	*CCA1* mRNA	LL 12:12	wt	
[2] Baudry, 2010	3E	*LHY* mRNA	LL 12:12	ztl-4	
[2] Baudry, 2010	3E	*LHY* mRNA	LL 12:12	wt	
[2] Baudry, 2010	3F	*PRR9* mRNA	LL 12:12	ztl-4	
[2] Baudry, 2010	3F	*PRR9* mRNA	LL 12:12	wt	
[2] Baudry, 2010	S1A	ZTL pr	LL 12:12	wt	
[2] Baudry, 2010	S2	*PRR5* mRNA	LL 12:12	wt	
[2] Baudry, 2010	S2	*PRR5* mRNA	LL 12:12	ztl-4	
[2] Baudry, 2010	S2A	*PRR7* mRNA	LL 12:12	ztl-4	
[2] Baudry, 2010	S2A	*PRR7* mRNA	LL 12:12	wt	
[2] Baudry, 2010	S2A	*PRR7* mRNA	LL 12:12	lhy-20	
[2] Baudry, 2010	S5	TOC1 pr	LL 12:12	ztl-4;fkf1;lkp2	
[2] Baudry, 2010	S5	TOC1 pr	LL 12:12	ztl-4	
[2] Baudry, 2010	S5	TOC1 pr	LL 12:12	wt	
[2] Baudry, 2010	S5	*TOC1* mRNA	LL 12:12	ztl-4	
[2] Baudry, 2010	S5	*TOC1* mRNA	LL 12:12	wt	
[2] Baudry, 2010	S5	PRR5 pr	LL 12:12	ztl-4;fkf1;lkp2	
[2] Baudry, 2010	S5	PRR5 pr	LL 12:12	ztl-4	
[2] Baudry, 2010	S5	PRR5 pr	LL 12:12	wt	
[3] Dai, 2011	1	*NOX* mRNA	LL 12:12	wt, Col-0	
[3] Dai, 2011	1	*NOX* mRNA	LL 12:12	35S:BOA-8	
[3] Dai, 2011	5C	*NOX* mRNA	LL 12:12	wt, Ws	
[3] Dai, 2011	5C	*NOX* mRNA	LL 12:12	cca1-11	
[3] Dai, 2011	5C	*NOX* mRNA	LL 12:12	lhy-21	
[3] Dai, 2011	5C	*NOX* mRNA	LL 12:12	lhy-21;cca1-11	
[3] Dai, 2011	6E	*CCA1* mRNA	LL 12:12	wt, Col-0	
[3] Dai, 2011	6E	*CCA1* mRNA	LL 12:12	35S:BOA-8	
[3] Dai, 2011	7A	*LHY* mRNA	LL 12:12	wt, Col-0	
[3] Dai, 2011	7A	*LHY* mRNA	LL 12:12	35S:BOA-8	
[3] Dai, 2011	7B	*GI* mRNA	LL 12:12	wt, Col-0	
[3] Dai, 2011	7B	*GI* mRNA	LL 12:12	35S:BOA-8	
[3] Dai, 2011	7C	*TOC1* mRNA	LL 12:12	wt, Col-0	
[3] Dai, 2011	7C	*TOC1* mRNA	LL 12:12	35S:BOA-8	
[3] Dai, 2011	8A	*NOX* mRNA	LL 12:12	wt, C24	
[3] Dai, 2011	8A	*NOX* mRNA	LL 12:12	toc1-1	
[3] Dai, 2011	8B	*CCA1* mRNA	LL 12:12	wt, C24	
[3] Dai, 2011	8B	*CCA1* mRNA	LL 12:12	toc1-1	5
[3] Dai, 2011	8C	*NOX* mRNA	LL 12:12	wt, Ler-0	
[3] Dai, 2011	8C	*NOX* mRNA	LL 12:12	gi-3	
[3] Dai, 2011	8C	*NOX* mRNA	LL 12:12	gi-4	
[3] Dai, 2011	S1	*CCA1* mRNA	LL 12:12	wt, Col-0	
[3] Dai, 2011	S1	*CCA1* mRNA	LL 12:12	boa-1	
[3] Dai, 2011	S1	*NOX* mRNA	LL 12:12	wt, Col-0	
[3] Dai, 2011	S1	*NOX* mRNA	LL 12:12	boa-1	
[3] Dai, 2011	S3	*NOX* mRNA	LL 12:12	wt, Col-0	
[3] Dai, 2011	S3	*NOX* mRNA	LL 12:12	CCA1-OX38	
[3] Dai, 2011	S6	*CCA1* mRNA	DD 12:12	wt, Col-0	

Ref.	Fig.	Reporter	Light	Mutant / Comment	Weight
[3] Dai, 2011	S6	*CCA1* mRNA	DD 12:12	35S:BOA-8	
[4] David, 2006	1C	GI pr	LD 16:8	HA-GI protein	
[4] David, 2006	1C	GI pr	LD 8:16	HA-GI protein	
[4] David, 2006	5C	GI pr	LD 16:8	wt	
[4] David, 2006	5C	GI pr	LD 8:16	wt	
[5] Ding, 2007	2A	*GI* mRNA	LL 12:12	cca1-11;lhy-21;toc1-21	
[5] Ding, 2007	2A	*GI* mRNA	LL 12:12	wt	
[5] Ding, 2007	2B	*LUX* mRNA	LL 12:12	cca1-11;lhy-21;toc1-21	
[5] Ding, 2007	2B	*LUX* mRNA	LL 12:12	wt	
[5] Ding, 2007	2C	*PRR9* mRNA	LL 12:12	cca1-11;lhy-21;toc1-21	
[5] Ding, 2007	2C	*PRR9* mRNA	LL 12:12	wt	
[5] Ding, 2007	5A	*PRR9* mRNA	LD 8:16	lhy-21	
[5] Ding, 2007	5A	*PRR9* mRNA	LD 8:16	cca1-11;toc1-21	
[5] Ding, 2007	5A	*PRR9* mRNA	LD 8:16	cca1-11;lhy-21;toc1-21	
[5] Ding, 2007	5A	*PRR9* mRNA	LD 8:16	cca1-11;lhy-21	
[5] Ding, 2007	5A	*PRR9* mRNA	LD 8:16	cca1-11	
[5] Ding, 2007	5A	*PRR9* mRNA	LD 8:16	wt	
[5] Ding, 2007	5C	*PRR9* mRNA	LD 8:16	toc1-21	
[5] Ding, 2007	5C	*PRR7* mRNA	LD 8:16	lhy-21;toc1-21	
[5] Ding, 2007	5C	*PRR7* mRNA	LD 8:16	lhy-21	
[5] Ding, 2007	5C	*PRR7* mRNA	LD 8:16	cca1-11;toc1-21	
[5] Ding, 2007	5C	*PRR7* mRNA	LD 8:16	cca1-11;lhy-21;toc1-21	
[5] Ding, 2007	5C	*PRR7* mRNA	LD 8:16	cca1-11;lhy-21	
[5] Ding, 2007	5C	*PRR7* mRNA	LD 8:16	cca1-11	
[5] Ding, 2007	5C	*PRR7* mRNA	LD 8:16	wt	
[6] Dixon, 2011	S3	*TOC1* mRNA	LD 12:12	elf3-4	3
[6] Dixon, 2011	S3	*TOC1* mRNA	LD 12:12	cca1-11;lhy-21;elf3-4	3
[6] Dixon, 2011	S3	*TOC1* mRNA	LD 12:12	cca1-11;lhy-21	3
[6] Dixon, 2011	S3, 2	*TOC1* mRNA	LD 12:12	wt, Ws	
[6] Dixon, 2011	S3	*PRR9* mRNA	LD 12:12	elf3-4	3
[6] Dixon, 2011	S3	*PRR9* mRNA	LD 12:12	cca1;lhy-21;elf3-4	3
[6] Dixon, 2011	S3	*PRR9* mRNA	LD 12:12	cca1-11;lhy-21	3
[6] Dixon, 2011	S3	*PRR9* mRNA	LD 12:12	wt, Ws	
[6] Dixon, 2011	S3	*PRR7* mRNA	LD 12:12	elf3-4	3
[6] Dixon, 2011	S3	*PRR7* mRNA	LD 12:12	cca1;lhy-21;elf3-4	3
[6] Dixon, 2011	S3	*PRR7* mRNA	LD 12:12	cca1-11;lhy-21	3
[6] Dixon, 2011	S3	*PRR7* mRNA	LD 12:12	wt, Ws	
[6] Dixon, 2011	S3	*GI* mRNA	LD 12:12	elf3-4	3
[6] Dixon, 2011	S3	*GI* mRNA	LD 12:12	cca1;lhy-21;elf3-4	3
[6] Dixon, 2011	S3	*GI* mRNA	LD 12:12	cca1-11;lhy-21	3
[6] Dixon, 2011	S3	*GI* mRNA	LD 12:12	wt, Ws	
[7] Edwards, 2010	2A	*CCA1* mRNA	LL 9:15	wt	
[7] Edwards, 2010	2A	*CCA1* mRNA	LL 6:18	wt	
[7] Edwards, 2010	2A	*CCA1* mRNA	LL 3:21	wt	
[7] Edwards, 2010	2A	*CCA1* mRNA	LL 18:6	wt	
[7] Edwards, 2010	2A	*CCA1* mRNA	LL 12:12	wt	
[7] Edwards, 2010	2C	*GI* mRNA	LL 9:15	wt	
[7] Edwards, 2010	2C	*GI* mRNA	LL 6:18	wt	
[7] Edwards, 2010	2C	*GI* mRNA	LL 3:21	wt	
[7] Edwards, 2010	2C	*GI* mRNA	LL 18:6	wt	
[7] Edwards, 2010	2C	*GI* mRNA	LL 12:12	wt	
[7] Edwards, 2010	2E	*TOC1* mRNA	LL 9:15	wt	
[7] Edwards, 2010	2E	*TOC1* mRNA	LL 6:18	wt	
[7] Edwards, 2010	2E	*TOC1* mRNA	LL 3:21	wt	

Ref.	Fig.	Reporter	Light	Mutant / Comment	Weight
[7] Edwards, 2010	2E	*TOC1* mRNA	LL 18:6	wt	
[7] Edwards, 2010	2E	*TOC1* mRNA	LL 12:12	wt	
[7] Edwards, 2010	2B	*CCA1* mRNA	DD 12:12	wt	
[7] Edwards, 2010	2B	*CCA1* mRNA	DD 18:6	wt	
[7] Edwards, 2010	2B	*CCA1* mRNA	DD 6:18	wt	
[7] Edwards, 2010	2D	*GI* mRNA	DD 12:12	wt	
[7] Edwards, 2010	2D	*GI* mRNA	DD 18:6	wt	
[7] Edwards, 2010	2D	*GI* mRNA	DD 6:18	wt	
[7] Edwards, 2010	2E	*TOC1* mRNA	DD 18:6	wt	
[7] Edwards, 2010	2F	*TOC1* mRNA	DD 12:12	wt	
[7] Edwards, 2010	2F	*TOC1* mRNA	DD 6:18	wt	
[7] Edwards, 2010	S3	*CCA1* mRNA	DD 12:12	wt	
[7] Edwards, 2010	S3	*GI* mRNA	DD 12:12	wt	
[7] Edwards, 2010	S3	*TOC1* mRNA	DD 12:12	wt	
[7] Edwards, 2010	S3	*CCA1* mRNA	DD 3:21	wt	
[7] Edwards, 2010	S3	*GI* mRNA	DD 3:21	wt	
[7] Edwards, 2010	S3	*TOC1* mRNA	DD 3:21	wt	
[7] Edwards, 2010	S3	*CCA1* mRNA	DD 9:15	wt	
[7] Edwards, 2010	S3	*GI* mRNA	DD 9:15	wt	
[7] Edwards, 2010	S3	*TOC1* mRNA	DD 9:15	wt	
[8] Farinas, 2011	1A	*CCA1* mRNA	LL 12:12	wt	
[8] Farinas, 2011	1B	*CCA1* mRNA	LL 8:16	wt	
[8] Farinas, 2011	1C	*CCA1* mRNA	LL 16:8	wt	
[8] Farinas, 2011	1A	*RVE8* mRNA	LL 12:12	wt	
[8] Farinas, 2011	1B	*RVE8* mRNA	LL 8:16	wt	
[8] Farinas, 2011	1C	*RVE8* mRNA	LL 16:8	wt	
[8] Farinas, 2011	1D	*TOC1* mRNA	LL 12:12	wt	
[8] Farinas, 2011	1F	*TOC1* mRNA	LL 16:8	wt	
[8] Farinas, 2011	1E	*TOC1* mRNA	LL 8:16	wt	
[8] Farinas, 2011	2C	*RVE8* mRNA	LL 16:8	wt	
[8] Farinas, 2011	2C	*RVE8* mRNA	LL 16:8	CCA1-ox	
[8] Farinas, 2011	2F	*RVE8* mRNA	LL 16:8	cca1;lhy	
[8] Farinas, 2011	3C	*CCA1* mRNA	LL 16:8	wt	
[8] Farinas, 2011	3C	*CCA1* mRNA	LL 16:8	RVE8-ox	
[8] Farinas, 2011	3F	*CCA1* mRNA	LL 16:8	rve8	
[8] Farinas, 2011	4C	*TOC1* mRNA	LL 16:8	wt	
[8] Farinas, 2011	4C	*TOC1* mRNA	LL 16:8	RVE8-ox	
[8] Farinas, 2011	4F	*TOC1* mRNA	LL 16:8	rve8	
[9] Farré, 2005	3F	*TOC1* mRNA	LL 12:12	prr7-3;prr9-1	3
[9] Farré, 2005	3E	*TOC1* mRNA	LL 12:12	prr9-1	
[9] Farré, 2005	3E	*TOC1* mRNA	LL 12:12	prr7-3	
[9] Farré, 2005	3E	*TOC1* mRNA	LL 12:12	wt, Col	
[9] Farré, 2005	3D	*LHY* mRNA	LL 12:12	prr7-3;prr9-1	3
[9] Farré, 2005	3C	*LHY* mRNA	LL 12:12	prr9-1	
[9] Farré, 2005	3C	*LHY* mRNA	LL 12:12	prr7-3	
[9] Farré, 2005	3C	*LHY* mRNA	LL 12:12	wt, Col	
[9] Farré, 2005	3B	*CCA1* mRNA	LL 12:12	prr7-3;prr9-1	3
[9] Farré, 2005	3A	*CCA1* mRNA	LL 12:12	prr9-1	
[9] Farré, 2005	3A	*CCA1* mRNA	LL 12:12	prr7-3	
[9] Farré, 2005	3A	*CCA1* mRNA	LL 12:12	wt, Col	
[9] Farré, 2005	4A	*PRR9* mRNA	LD 12:12	wt, Ws	
[9] Farré, 2005	4A	*PRR9* mRNA	LD 12:12	cca1-1	
[9] Farré, 2005	4A	*PRR9* mRNA	LD 12:12	cca1-1;lhy-R	
[9] Farré, 2005	4B	*PRR9* mRNA	LD 12:12	cca1-ox	

Ref.	Fig.	Reporter	Light	Mutant / Comment	Weight
[9] Farré, 2005	4B	*PRR9* mRNA	LD 12:12	wt, Col	
[9] Farré, 2005	4C	*PRR9* mRNA	LD 12:12	lhy-1 (-ox)	
[9] Farré, 2005	4C	*PRR9* mRNA	LD 12:12	wt, Ler	
[9] Farré, 2005	4D	*PRR7* mRNA	LD 12:12	wt, Ws	
[9] Farré, 2005	4D	*PRR7* mRNA	LD 12:12	cca1-1, Ws	
[9] Farré, 2005	4D	*PRR7* mRNA	LD 12:12	cca1-1;lhy-R	
[9] Farré, 2005	4E	*PRR7* mRNA	LD 12:12	cca1-ox	
[9] Farré, 2005	4E	*PRR7* mRNA	LD 12:12	wt, Col	
[9] Farré, 2005	4F	*PRR7* mRNA	LD 12:12	lhy-1 (-ox)	
[9] Farré, 2005	4F	*PRR7* mRNA	LD 12:12	wt, Ler	
[10] Farré, 2007	3G	*TOC1* mRNA	LL 12:12	P7-ox	3
[10] Farré, 2007	3G	*TOC1* mRNA	LL 12:12	wt	
[10] Farré, 2007	3J	*PRR7* mRNA	LL 12:12	P7-ox	3
[10] Farré, 2007	3J	*PRR7* mRNA	LL 12:12	wt	
[10] Farré, 2007	3D	*LHY* mRNA	LL 12:12	P7-ox	3
[10] Farré, 2007	3D	*LHY* mRNA	LL 12:12	wt	
[10] Farré, 2007	3A	*CCA1* mRNA	LL 12:12	P7-ox	3
[10] Farré, 2007	3A	*CCA1* mRNA	LL 12:12	wt	
[10] Farré, 2007	4B	*CCA1* mRNA	LL 12:12	P7-ox	3
[10] Farré, 2007	4B	*CCA1* mRNA	LL 12:12	wt	
[10] Farré, 2007	4B	*CCA1* mRNA	LL 12:12	prr7-3	
[10] Farré, 2007	5A	PRR7 pr	LL 12:12	wt	
[10] Farré, 2007	5B	PRR7 pr	DD 12:12	wt	
[10] Farré, 2007	5C	PRR7 pr	LL 12:12	P7-ox	
[10] Farré, 2007	5D	PRR7 pr	DD 12:12	P7-ox	
[11] Fowler, 1999	3A	*GI* mRNA	DD 18:6	wt	
[11] Fowler, 1999	3A	*GI* mRNA	LD 18:6	wt	
[11] Fowler, 1999	3A	*GI* mRNA	LL 18:6	wt	
[11] Fowler, 1999	3B	*GI* mRNA	LD 10:14	wt, shifted 18:6 to 10:14	
[11] Fowler, 1999	3B	*GI* mRNA	LD 18:6	wt	
[11] Fowler, 1999	3B	*GI* mRNA	LD 10:14	wt	
[11] Fowler, 1999	3B	*GI* mRNA	LD 18:6	wt	
[11] Fowler, 1999	4A	*GI* mRNA	LD 18:6	wt, measured in LDL 18:5:1	
[11] Fowler, 1999	4A	*GI* mRNA	LD 18:6	elf3, measured in LDL 18:5:1	
[11] Fowler, 1999	4C	*GI* mRNA	LL 18:6	elf3	
[11] Fowler, 1999	4C	*GI* mRNA	LL 18:6	wt	
[11] Fowler, 1999	5A	*GI* mRNA	LL 18:6	C-ox	
[11] Fowler, 1999	5A	*GI* mRNA	LL 18:6	wt	
[11] Fowler, 1999	5B	*GI* mRNA	LD 18:6	lhy-ox	
[11] Fowler, 1999	5B	*GI* mRNA	LD 18:6	wt	
[11] Fowler, 1999	5C	*CCA1* mRNA	LD 18:6	lhy-ox	
[11] Fowler, 1999	5C	*CCA1* mRNA	LD 18:6	wt	
[11] Fowler, 1999	6A	*LHY* mRNA	LD 18:6	wt, measured in LDL 18:5:1	
[11] Fowler, 1999	6A	*LHY* mRNA	LD 18:6	gi-3, measured in LDL 18:5:1	
[11] Fowler, 1999	6B	*CCA1* mRNA	LD 18:6	wt, measured in LDL 18:5:1	
[11] Fowler, 1999	6B	*CCA1* mRNA	LD 18:6	gi-3, measured in LDL 18:5:1	
[12] Fujiwara, 2008	1A	*PRR9* mRNA	LL 12:12	wt	
[12] Fujiwara, 2008	1B	*PRR7* mRNA	LL 12:12	wt	
[12] Fujiwara, 2008	1C	*PRR5* mRNA	LL 12:12	wt	
[12] Fujiwara, 2008	1E	*TOC1* mRNA	LL 12:12	wt	
[12] Fujiwara, 2008	3A	*PRR9* mRNA	LL 12:12	ztl-1	
[13] Hazen, 2005	4A	*CCA1* mRNA	LL 12:12	wt	
[13] Hazen, 2005	4A	*CCA1* mRNA	LL 12:12	lux-1	
[13] Hazen, 2005	4A	*CCA1* mRNA	LL 12:12	lux-2	

Ref.	Fig.	Reporter	Light	Mutant / Comment	Weight
[13] Hazen, 2005	4B	*LHY* mRNA	LL 12:12	wt	
[13] Hazen, 2005	4B	*LHY* mRNA	LL 12:12	lux-1	
[13] Hazen, 2005	4B	*LHY* mRNA	LL 12:12	lux-2	
[13] Hazen, 2005	4C	*TOC1* mRNA	LL 12:12	wt	
[13] Hazen, 2005	4C	*TOC1* mRNA	LL 12:12	lux-1	
[13] Hazen, 2005	4C	*TOC1* mRNA	LL 12:12	lux-2	
[14] Helfer, 2011	3A	*LUX* mRNA	LD 12:12	wt	
[14] Helfer, 2011	3A	*PRR9* mRNA	LD 12:12	wt	
[14] Helfer, 2011	3B	*LUX* mRNA	LL 12:12	wt	
[14] Helfer, 2011	3B	*PRR9* mRNA	LL 12:12	wt	
[14] Helfer, 2011	3C	*PRR9* mRNA	LL 12:12	wt	
[14] Helfer, 2011	3C	*PRR9* mRNA	LL 12:12	lux-4	
[14] Helfer, 2011	S1B	*LUX* mRNA	LD 12:12	wt	
[14] Helfer, 2011	S1B	*NOX* mRNA	LD 12:12	wt	
[14] Helfer, 2011	S1C	*LUX* mRNA	LL 12:12	wt	
[14] Helfer, 2011	S1C	*NOX* mRNA	LL 12:12	wt	
[15] Herrero, 2011	3.11	*PRR9* mRNA	LD 8:16	E4-ox	
[15] Herrero, 2011	3.11	*PRR9* mRNA	LD 8:16	E3-ox	
[15] Herrero, 2011	3.11	*PRR9* mRNA	LD 8:16	wt	
[15] Herrero, 2011	3.11	*PRR7* mRNA	LD 8:16	E4-ox	
[15] Herrero, 2011	3.11	*PRR7* mRNA	LD 8:16	E3-ox	
[15] Herrero, 2011	3.11	*PRR7* mRNA	LD 8:16	wt	
[15] Herrero, 2011	3.11	*PRR9* mRNA	LL 12:12	E4-ox	
[15] Herrero, 2011	3.11	*PRR9* mRNA	LL 12:12	E3-ox	
[15] Herrero, 2011	3.11	*PRR9* mRNA	LL 12:12	wt	
[15] Herrero, 2011	3.11	*PRR7* mRNA	LL 12:12	E4-ox	
[15] Herrero, 2011	3.11	*PRR7* mRNA	LL 12:12	E3-ox	
[15] Herrero, 2011	3.11	*PRR7* mRNA	LL 12:12	wt	
[15] Herrero, 2011	3.2A	*LHY* mRNA	LL 12:12	elf3-4, LHY::LUC	
[15] Herrero, 2011	3.2A	*LHY* mRNA	LL 12:12	E4-ox, LHY::LUC	
[15] Herrero, 2011	3.2B	*LHY* mRNA	LL 12:12	elf4-1, LHY::LUC	
[15] Herrero, 2011	3.2B	*LHY* mRNA	LL 12:12	E3-ox, LHY::LUC	
[15] Herrero, 2011	3.2B	*LHY* mRNA	LL 12:12	wt, LHY::LUC	
[16] Hicks, 2001	4B	*ELF3* mRNA	LD 12:12	wt	
[16] Hicks, 2001	4D	*ELF3* mRNA	LD 12:12	elf3-1	
[16] Hicks, 2001	4D	*ELF3* mRNA	LD 12:12	elf3-2	
[16] Hicks, 2001	4E	*ELF3* mRNA	LD 18:6	wt	
[16] Hicks, 2001	4E	*ELF3* mRNA	LD 9:15	wt	
[16] Hicks, 2001	6A	*ELF3* mRNA	LL 12:12	wt, Ler	
[16] Hicks, 2001	6A	*ELF3* mRNA	LL 12:12	lhy	
[17] Hsu, 2012	2H	*CCA1* mRNA	LL 12:12	Col	
[17] Hsu, 2012	2H	*CCA1* mRNA	LL 12:12	rve8-1	
[17] Hsu, 2012	1C	*RVE8* mRNA	LL 12:12	Col	
[17] Hsu, 2012	1C	*RVE8* mRNA	LL 12:12	rve8	
[17] Hsu, 2012	2G	*CCA1* mRNA	LL 12:12	Col	
[17] Hsu, 2012	2G	*CCA1* mRNA	LL 12:12	rve8-1	
[18] Hsu, 2013	5A	*PRR5* mRNA	LD 12:12	wt, Col	
[18] Hsu, 2013	5A	*PRR5* mRNA	LD 12:12	rve4,rve6,rve8	
[18] Hsu, 2013	5B	*TOC1* mRNA	LD 12:12	wt, Col	
[18] Hsu, 2013	5B	*TOC1* mRNA	LD 12:12	rve4,rve6,rve8	
[18] Hsu, 2013	5C	*CCA1* mRNA	LD 12:12	wt, Col	
[18] Hsu, 2013	5C	*CCA1* mRNA	LD 12:12	rve4,rve6,rve8	
[18] Hsu, 2013	5D	*LHY* mRNA	LD 12:12	wt, Col	
[18] Hsu, 2013	5D	*LHY* mRNA	LD 12:12	rve4,rve6,rve8	

Ref.	Fig.	Reporter	Light	Mutant / Comment	Weight
[18] Hsu, 2013	5I	*PRR5* mRNA	LL 12:12	wt Col	
[18] Hsu, 2013	5I	*PRR5* mRNA	LL 12:12	rve4,rve6,rve8	
[18] Hsu, 2013	5J	*TOC1* mRNA	LL 12:12	wt Col	
[18] Hsu, 2013	5J	*TOC1* mRNA	LL 12:12	rve4,rve6,rve8	
[18] Hsu, 2013	5K	*CCA1* mRNA	LL 12:12	wt Col	
[18] Hsu, 2013	5K	*CCA1* mRNA	LL 12:12	rve4,rve6,rve8	
[18] Hsu, 2013	5L	*LHY* mRNA	LL 12:12	wt Col	
[18] Hsu, 2013	5L	*LHY* mRNA	LL 12:12	rve4,rve6,rve8	
[18] Hsu, 2013	5M	*TOC1* mRNA	LL 12:12	wt Col	
[18] Hsu, 2013	5M	*TOC1* mRNA	LL 12:12	rve4,rve6,rve8	
[18] Hsu, 2013	5O	*LHY* mRNA	LL 12:12	wt Col	
[18] Hsu, 2013	5O	*LHY* mRNA	LL 12:12	rve4,rve6,rve8	
[18] Hsu, 2013	6A	*RVE8* mRNA	LD 12:12	wt Col	
[18] Hsu, 2013	6A	*RVE8* mRNA	LD 12:12	toc1-4	
[18] Hsu, 2013	6A	*RVE8* mRNA	LD 12:12	lux-1	
[18] Hsu, 2013	6A	*RVE8* mRNA	LD 12:12	CCA1-ox	
[18] Hsu, 2013	6B	*PRR5* mRNA	LD 12:12	wt Col, one point	
[18] Hsu, 2013	6B	*PRR5* mRNA	LD 12:12	toc1-4, one point	
[18] Hsu, 2013	6B	*PRR5* mRNA	LD 12:12	lux-1, one point	
[18] Hsu, 2013	6B	*PRR5* mRNA	LD 12:12	CCA1-ox, one point	
[18] Hsu, 2013	6B	*PRR7* mRNA	LD 12:12	wt Col, one point	
[18] Hsu, 2013	6B	*PRR7* mRNA	LD 12:12	toc1-4, one point	
[18] Hsu, 2013	6B	*PRR7* mRNA	LD 12:12	lux-1, one point	
[18] Hsu, 2013	6B	*PRR7* mRNA	LD 12:12	CCA1-ox, one point	
[18] Hsu, 2013	6B	*PRR9* mRNA	LD 12:12	wt Col, one point	
[18] Hsu, 2013	6B	*PRR9* mRNA	LD 12:12	toc1-4, one point	
[18] Hsu, 2013	6B	*PRR9* mRNA	LD 12:12	lux-1, one point	
[18] Hsu, 2013	6B	*PRR9* mRNA	LD 12:12	CCA1-ox, one point	
[19] Huang, 2012	2L	*ELF4* mRNA	LD 12:12	wt	
[19] Huang, 2012	2L	*ELF4* mRNA	LD 12:12	TOC1-ox	
[19] Huang, 2012	2K	*GI* mRNA	LD 12:12	wt	
[19] Huang, 2012	2K	*GI* mRNA	LD 12:12	TOC1-ox	
[19] Huang, 2012	2I	*PRR9* mRNA	LD 12:12	TOC1-ox	
[19] Huang, 2012	2I	*PRR9* mRNA	LD 12:12	wt	
[19] Huang, 2012	2J	*PRR7* mRNA	LD 12:12	TOC1-ox	
[19] Huang, 2012	2J	*PRR7* mRNA	LD 12:12	wt	
[19] Huang, 2012	4A	*LHY* mRNA	LL 12:12	wt	
[19] Huang, 2012	4A	*LHY* mRNA	LL 12:12	TOCRNAi	
[19] Huang, 2012	4B	*PRR7* mRNA	LL 12:12	wt	
[19] Huang, 2012	4B	*PRR7* mRNA	LL 12:12	TOCRNAi	
[19] Huang, 2012	4C	*PRR9* mRNA	LL 12:12	wt	
[19] Huang, 2012	4C	*PRR9* mRNA	LL 12:12	TOCRNAi	
[19] Huang, 2012	4D	*GI* mRNA	LL 12:12	wt	
[19] Huang, 2012	4D	*GI* mRNA	LL 12:12	TOCRNAi	
[19] Huang, 2012	S10	*PRR7* mRNA	LD 12:12	wt	
[19] Huang, 2012	S10	*LHY* mRNA	LD 12:12	wt	
[19] Huang, 2012	S10	*LHY* mRNA	LD 12:12	toc1-2	
[19] Huang, 2012	S10	*PRR7* mRNA	LD 12:12	toc1-2	
[19] Huang, 2012	S7	*PRR9* mRNA	LL 12:12	TOC1-ox	
[19] Huang, 2012	S7	*PRR9* mRNA	LL 12:12	wt	
[19] Huang, 2012	S7	*PRR7* mRNA	LL 12:12	TOC1-ox	
[19] Huang, 2012	S7	*PRR7* mRNA	LL 12:12	wt	
[19] Huang, 2012	S7	*LHY* mRNA	LL 12:12	TOC1-ox	
[19] Huang, 2012	S7	*LHY* mRNA	LL 12:12	wt	

Ref.	Fig.	Reporter	Light	Mutant / Comment	Weight
[19] Huang, 2012	S7	*CCA1* mRNA	LL 12:12	TOC1-ox	
[19] Huang, 2012	S7	*CCA1* mRNA	LL 12:12	wt	
[19] Huang, 2012	S8B	*LUX* mRNA	LD 12:12	wt, LUX::LUC	
[19] Huang, 2012	S8C	*LUX* mRNA	LL 12:12	wt, LUX::LUC	
[19] Huang, 2012	S8D	*LUX* mRNA	LD 12:12	TOC1-ox, LUX::LUC	
[19] Huang, 2012	S8D	*LUX* mRNA	LD 12:12	wt, LUX::LUC	
[20] Ito, 2008	2C	*GI* mRNA	LL 12:12	toc1-2;prr5-11	
[20] Ito, 2008	2C	*GI* mRNA	LL 12:12	toc1-2	
[20] Ito, 2008	2C	*GI* mRNA	LL 12:12	wt	
[20] Ito, 2008	2C	*CCA1* mRNA	LL 12:12	toc1-2;prr5-11	
[20] Ito, 2008	2C	*CCA1* mRNA	LL 12:12	toc1-2	
[20] Ito, 2008	2C	*CCA1* mRNA	LL 12:12	wt	
[20] Ito, 2008	4	*GI* mRNA	LD 16:8	toc1-2;prr5-11	
[20] Ito, 2008	4	*GI* mRNA	LD 16:8	toc1-2	
[20] Ito, 2008	4	*GI* mRNA	LD 16:8	prr5-11	
[20] Ito, 2008	4	*GI* mRNA	LD 16:8	wt	
[20] Ito, 2008	4G	*LHY* mRNA	LD 16:8	prr5-11	
[20] Ito, 2008	4G	*LHY* mRNA	LD 16:8	wt	
[20] Ito, 2008	4H	*LHY* mRNA	LD 16:8	toc1-2;prr5-11	
[20] Ito, 2008	4H	*LHY* mRNA	LD 16:8	toc1-2	
[21] Kaczorowski, 2003	6A	*CCA1* mRNA	LL 12:12	wt, Red light entr	
[21] Kaczorowski, 2003	6A	*CCA1* mRNA	LL 12:12	prr7-1, Red light entr	
[21] Kaczorowski, 2003	6B	*LHY* mRNA	LL 12:12	wt, Red light entr	
[21] Kaczorowski, 2003	6B	*LHY* mRNA	LL 12:12	prr7-1, Red light entr	
[21] Kaczorowski, 2003	6C	*TOC1* mRNA	LL 12:12	wt, Red light entr	
[21] Kaczorowski, 2003	6C	*TOC1* mRNA	LL 12:12	prr7-1, Red light entr	
[22] Kiba, 2007	1B	*PRR5* mRNA	LD 12:12	wt	
[22] Kiba, 2007	1B	PRR5 pr	LD 12:12	wt	
[22] Kiba, 2007	1C	*PRR5* mRNA	LL 12:12	wt	
[22] Kiba, 2007	1C	PRR5 pr	LL 12:12	wt	
[22] Kiba, 2007	1D	PRR5 pr	DD 12:12	wt	
[22] Kiba, 2007	1D	*PRR5* mRNA	DD 12:12	wt	
[22] Kiba, 2007	1E	*PRR5* mRNA	LD 16:8	wt	
[22] Kiba, 2007	1E	PRR5 pr	LD 16:8	wt	
[22] Kiba, 2007	1F	*PRR5* mRNA	LD 8:16	wt	
[22] Kiba, 2007	1F	PRR5 pr	LD 8:16	wt	
[23] Kikis, 2005	3A	*TOC1* mRNA	RR 12:12	wt, Ws (DD to RR)	
[23] Kikis, 2005	3A	*TOC1* mRNA	RR 12:12	cca1-1;lhy-12 (DD to RR)	
[23] Kikis, 2005	4A	*CCA1* mRNA	RR 12:12	wt, Col (DD to RR)	
[23] Kikis, 2005	4A	*CCA1* mRNA	RR 12:12	toc-101 (DD to RR)	
[23] Kikis, 2005	4C	*LHY* mRNA	RR 12:12	wt, Col (DD to RR)	
[23] Kikis, 2005	4C	*LHY* mRNA	RR 12:12	toc-101 (DD to RR)	
[23] Kikis, 2005	5A	*CCA1* mRNA	RR 12:12	wt, Col (DD to RR)	
[23] Kikis, 2005	5A	*CCA1* mRNA	RR 12:12	elf4-101 (DD to RR)	
[23] Kikis, 2005	5C	*LHY* mRNA	RR 12:12	wt, Col (DD to RR)	
[23] Kikis, 2005	5C	*LHY* mRNA	RR 12:12	elf4-101 (DD to RR)	
[23] Kikis, 2005	5E	*TOC1* mRNA	RR 12:12	wt, Col (DD to RR)	
[23] Kikis, 2005	5E	*TOC1* mRNA	RR 12:12	elf4-101 (DD to RR)	
[23] Kikis, 2005	6A	*CCA1* mRNA	RR 12:12	wt, Col (DD to RR)	
[23] Kikis, 2005	6A	*CCA1* mRNA	RR 12:12	elf3-1 (DD to RR)	
[23] Kikis, 2005	6C	*LHY* mRNA	RR 12:12	wt, Col (DD to RR)	
[23] Kikis, 2005	6C	*LHY* mRNA	RR 12:12	elf3-1 (DD to RR)	
[23] Kikis, 2005	6E	*TOC1* mRNA	RR 12:12	wt, Col (DD to RR)	
[23] Kikis, 2005	6E	*TOC1* mRNA	RR 12:12	elf3-1 (DD to RR)	

Ref.	Fig.	Reporter	Light	Mutant / Comment	Weight
[23] Kikis, 2005	7A	*ELF4* mRNA	RR 12:12	wt, Col (DD to RR)	
[23] Kikis, 2005	7A	*ELF4* mRNA	RR 12:12	elf3-1 (DD to RR)	
[23] Kikis, 2005	7C	*ELF3* mRNA	RR 12:12	wt, Col (DD to RR)	
[23] Kikis, 2005	7C	*ELF3* mRNA	RR 12:12	elf4-101, (DD to RR)	
[23] Kikis, 2005	8A	*ELF4* mRNA	RR 12:12	wt, Ws (DD to RR)	
[23] Kikis, 2005	8A	*ELF4* mRNA	RR 12:12	cca1-1;lhy-12 (DD to RR)	
[24] Kim, J-Y, 2003	3C	*LHY* mRNA	LD 12:12	LHY-ox	
[24] Kim, J-Y, 2003	3C	*LHY* mRNA	LD 12:12	wt	
[24] Kim, J-Y, 2003	3C	LHY pr	LD 12:12	LHY-ox	
[24] Kim, J-Y, 2003	3C	LHY pr	LD 12:12	wt	
[25] Kim, W-Y, 2005	5A	*GI* mRNA	LD 16:8	wt, Col	
[25] Kim, W-Y, 2005	5A	*GI* mRNA	LD 16:8	elf3-1	
[25] Kim, W-Y, 2005	5A	*GI* mRNA	LD 16:8	E3-ox	
[25] Kim, W-Y, 2005	5A	*GI* mRNA	LD 16:8	elf3-1 ZTL-ox	
[25] Kim, W-Y, 2005	5A	*GI* mRNA	LD 16:8	elf3-1;ztl-3	
[25] Kim, W-Y, 2005	5A	*GI* mRNA	LD 16:8	ztl-3	
[25] Kim, W-Y, 2005	5A	*GI* mRNA	LD 16:8	ZTL-ox	
[26] Kim, W-Y, 2007	1B	ZTL pr	LD 12:12	GI-ox	10
[26] Kim, W-Y, 2007	1B	ZTL pr	LD 12:12	gi-1	10
[26] Kim, W-Y, 2007	1B	ZTL pr	LD 12:12	wt	10
[26] Kim, W-Y, 2007	1C	GI pr	LD 12:12	wt	
[26] Kim, W-Y, 2007	1C	GI pr	LD 12:12	ztl-103	
[26] Kim, W-Y, 2007	1C	GI pr	LD 12:12	wt, cyt	
[26] Kim, W-Y, 2007	1C	GI pr	LD 12:12	ztl-103	
[26] Kim, W-Y, 2007	S5	TOC1 pr	LD 12:12	wt, nuc	
[26] Kim, W-Y, 2007	S5	TOc1 pr	LD 12:12	wt, cyt	
[26] Kim, W-Y, 2007	S5	GI pr	LD 12:12	wt, nuc	
[26] Kim, W-Y, 2007	2F	ZTL pr	LD 12:12	ztl-21	10
[26] Kim, W-Y, 2007	2F	ZTL pr	LD 12:12	wt	10
[26] Kim, W-Y, 2007	4A	TOC1 pr	LD 12:12	gi-2	
[26] Kim, W-Y, 2007	4A	TOC1 pr	LD 12:12	wt	
[26] Kim, W-Y, 2007	4B	*TOC1* mRNA	LD 12:12	gi-2	
[26] Kim, W-Y, 2007	4B	*TOC1* mRNA	LD 12:12	wt	
[27] Kim, Y. 2013	2A	*LHY* mRNA	LD 16:8	wt	
[27] Kim, Y. 2013	2A	*LHY* mRNA	LD 16:8	gi-2	
[27] Kim, Y. 2013	2A	*LHY* mRNA	LD 16:8	GI-NLS	
[27] Kim, Y. 2013	2A	*LHY* mRNA	LD 16:8	GI-NES	
[27] Kim, Y. 2013	2B	*LHY* mRNA	LL 12:12	wt	
[27] Kim, Y. 2013	2B	*LHY* mRNA	LL 12:12	gi-2	
[27] Kim, Y. 2013	2B	*LHY* mRNA	LL 12:12	GI-NLS	
[27] Kim, Y. 2013	2B	*LHY* mRNA	LL 12:12	GI-NES	
[27] Kim, Y. 2013	4F	GI pr (N)	LD 16:8	wt	
[27] Kim, Y. 2013	4F	GI pr (C)	LD 16:8	wt	
[27] Kim, Y. 2013	4F	GI pr (N)	LD 16:8	GI-constitutive	
[27] Kim, Y. 2013	4F	GI pr (C)	LD 16:8	GI-constitutive	
[27] Kim, Y. 2013	4G	*LHY* mRNA	LD 16:8	GI-constitutive	
[27] Kim, Y. 2013	S1B	*GI* mRNA	LD 16:8	wt	
[27] Kim, Y. 2013	S1B	*GI* mRNA	LD 16:8	GI-NES (GI in cyt)	
[27] Kim, Y. 2013	S1B	*GI* mRNA	LD 16:8	GI-NLS (GI in nuc)	
[27] Kim, Y. 2013	S1C	GI pr	LD 16:8	GI-NLS	
[27] Kim, Y. 2013	S1C	GI pr	LD 16:8	GI-NES	
[27] Kim, Y. 2013	S2A	*TOC1* mRNA	LD 16:8	wt	
[27] Kim, Y. 2013	S2A	*TOC1* mRNA	LD 16:8	gi-2	
[27] Kim, Y. 2013	S2A	*TOC1* mRNA	LD 16:8	GI-NLS	

Ref.	Fig.	Reporter	Light	Mutant / Comment	Weight
[27] Kim, Y. 2013	S2A	*TOC1* mRNA	LD 16:8	GI-NES	
[27] Kim, Y. 2013	S6	*LHY* mRNA	LD 8:16	wt Ler	
[27] Kim, Y. 2013	S6	*LHY* mRNA	LD 8:16	lhy-mutant	
[28] Kolmos, 2009	4A	*CCA1* mRNA	LD 12:12	wt	
[28] Kolmos, 2009	4A	*CCA1* mRNA	LD 12:12	elf4-207	
[28] Kolmos, 2009	4A	*CCA1* mRNA	DD 12:12	wt, Col-0	0.1
[28] Kolmos, 2009	4A	*CCA1* mRNA	DD 12:12	elf4-207	0.1
[28] Kolmos, 2009	4A	*CCA1* mRNA	LL 12:12	elf4-207	
[28] Kolmos, 2009	4A	*CCA1* mRNA	LL 12:12	Col-0, wt	
[28] Kolmos, 2009	4B	*LHY* mRNA	LD 12:12	elf4-207	
[28] Kolmos, 2009	4B	*LHY* mRNA	LD 12:12	Col-0, wt	
[28] Kolmos, 2009	4B	*LHY* mRNA	DD 12:12	wt, Col-0	0.1
[28] Kolmos, 2009	4B	*LHY* mRNA	DD 12:12	elf4-207	0.1
[28] Kolmos, 2009	4B	*LHY* mRNA	LL 12:12	elf4-207	
[28] Kolmos, 2009	4B	*LHY* mRNA	LL 12:12	Col-0, wt	
[28] Kolmos, 2009	4C	*PRR9* mRNA	LD 12:12	elf4-207	
[28] Kolmos, 2009	4C	*PRR9* mRNA	LD 12:12	Col-0, wt	
[28] Kolmos, 2009	4C	*PRR9* mRNA	DD 12:12	wt	0.1
[28] Kolmos, 2009	4C	*PRR9* mRNA	DD 12:12	elf4-207	0.1
[28] Kolmos, 2009	4C	*PRR9* mRNA	LL 12:12	Col-0, wt	
[28] Kolmos, 2009	4C	*PRR9* mRNA	LL 12:12	elf4-207	
[28] Kolmos, 2009	4D	*PRR7* mRNA	LD 12:12	elf4-207	
[28] Kolmos, 2009	4D	*PRR7* mRNA	LD 12:12	Col-0, wt	
[28] Kolmos, 2009	4D	*PRR7* mRNA	DD 12:12	wt, Col-0	0.1
[28] Kolmos, 2009	4D	*PRR7* mRNA	DD 12:12	elf4-207	0.1
[28] Kolmos, 2009	4D	*PRR7* mRNA	LL 12:12	elf4-207	
[28] Kolmos, 2009	4D	*PRR7* mRNA	LL 12:12	Col-0, wt	
[28] Kolmos, 2009	4E	*GI* mRNA	LD 12:12	elf4-207	
[28] Kolmos, 2009	4E	*GI* mRNA	LD 12:12	Col-0, wt	
[28] Kolmos, 2009	4E	*GI* mRNA	DD 12:12	wt, Col-0	0.1
[28] Kolmos, 2009	4E	*GI* mRNA	DD 12:12	elf4-207	0.1
[28] Kolmos, 2009	4E	*GI* mRNA	LL 12:12	elf4-207	
[28] Kolmos, 2009	4E	*GI* mRNA	LL 12:12	Col-0, wt	
[28] Kolmos, 2009	4F	*TOC1* mRNA	LD 12:12	elf4-207	
[28] Kolmos, 2009	4F	*TOC1* mRNA	LD 12:12	Col-0, wt	
[28] Kolmos, 2009	4F	*TOC1* mRNA	DD 12:12	wt	0.1
[28] Kolmos, 2009	4F	*TOC1* mRNA	DD 12:12	elf4-207	0.1
[28] Kolmos, 2009	4F	*TOC1* mRNA	LL 12:12	elf4-207	
[28] Kolmos, 2009	4F	*TOC1* mRNA	LL 12:12	Col-0, wt	
[28] Kolmos, 2009	4G	*LUX* mRNA	LD 12:12	elf4-207	
[28] Kolmos, 2009	4G	*LUX* mRNA	LD 12:12	Col-0, wt	
[28] Kolmos, 2009	4G	*LUX* mRNA	DD 12:12	wt	0.1
[28] Kolmos, 2009	4G	*LUX* mRNA	DD 12:12	elf4-207	0.1
[28] Kolmos, 2009	4G	*LUX* mRNA	LL 12:12	elf4-207	
[28] Kolmos, 2009	4G	*LUX* mRNA	LL 12:12	Col-0, wt	
[29] Lau, 2011	3C	*TOC1* mRNA	LL 16:8	Col	
[29] Lau, 2011	3C	*TOC1* mRNA	LL 16:8	det1-1	
[29] Lau, 2011	4A	*TOC1* mRNA	LL 16:8	CCA1-ox	
[29] Lau, 2011	4A	*TOC1* mRNA	LL 16:8	det1-1;CCA1-ox	
[29] Lau, 2011	3D	*GI* mRNA	LL 16:8	Col	
[29] Lau, 2011	3D	*GI* mRNA	LL 16:8	det1-1	
[29] Lau, 2011	4B	*GI* mRNA	LL 16:8	CCA1-ox	
[29] Lau, 2011	4B	*GI* mRNA	LL 16:8	det1-1;CCA1-ox	
[29] Lau, 2011	S2B	LHY pr	LL 16:8	Col wt	

Ref.	Fig.	Reporter	Light	Mutant / Comment	Weight
[29] Lau, 2011	S2B	LHY pr	LL 16:8	det1-1	
[29] Lau, 2011	S2C	*LHY* mRNA	LL 16:8	Col wt	
[29] Lau, 2011	S2C	*LHY* mRNA	LL 16:8	det1-1	
[29] Lau, 2011	S2D	*CCA1* mRNA	LL 16:8	Col wt	
[29] Lau, 2011	S2D	*CCA1* mRNA	LL 16:8	det1-1	
[30] Li, 2011	3D	*ELF4* mRNA	LL 12:12	Col-0, wt	
[30] Li, 2011	3D	*ELF4* mRNA	LL 12:12	CCA1-ox	
[30] Li, 2011	5E	*ELF4* mRNA	LD 12:12	Ws	
[30] Li, 2011	5E	*ELF4* mRNA	LD 12:12	cca1;lhy	
[30] Li, 2011	5E	*ELF4* mRNA	LL 12:12	Ws	
[30] Li, 2011	5E	*ELF4* mRNA	LL 12:12	cca1;lhy	
[31] Liu, 2001	2C	ELF3 pr	LL 12:12	first plot	
[31] Liu, 2001	2C	ELF3 pr	LL 12:12	second plot	
[32] Locke, 2005	6	*GI* mRNA	LL 12:12	wt	
[32] Locke, 2005	6	*GI* mRNA	LL 12:12	cca1;lhy	
[33] Lu, 2012	1	*ELF3* mRNA	LL 12:12	CCA1-ox	
[33] Lu, 2012	1	*ELF3* mRNA	LL 12:12	cca1-1	
[33] Lu, 2012	1	*ELF3* mRNA	LL 12:12	wt	
[33] Lu, 2012	1	*CCA1* mRNA	LL 12:12	ELF3-ox	
[33] Lu, 2012	1	*CCA1* mRNA	LL 12:12	elf3-1	
[33] Lu, 2012	1	*CCA1* mRNA	LL 12:12	wt	
[33] Lu, 2012	6	*GI* mRNA	LD 16:8	wt	
[33] Lu, 2012	6	*GI* mRNA	LD 16:8	elf3-1;C-ox	
[33] Lu, 2012	6	*GI* mRNA	LD 16:8	elf3-1	
[33] Lu, 2012	6	*GI* mRNA	LD 16:8	C-ox	
[33] Lu, 2012	7	*GI* mRNA	LD 8:16	elf3;C-ox	
[33] Lu, 2012	7	*GI* mRNA	LD 8:16	elf3	
[33] Lu, 2012	7	*GI* mRNA	LD 8:16	C-ox	
[33] Lu, 2012	7	*GI* mRNA	LD 8:16	wt	
[34] Martin-Tryon, 2007	5A	*CCA1* mRNA	LL 12:12	Col	
[34] Martin-Tryon, 2007	5A	*CCA1* mRNA	LL 12:12	gi-201	
[34] Martin-Tryon, 2007	5A	*CCA1* mRNA	LL 12:12	toc1-2	
[34] Martin-Tryon, 2007	5B	*LHY* mRNA	LL 12:12	Col	
[34] Martin-Tryon, 2007	5B	*LHY* mRNA	LL 12:12	gi-201	
[34] Martin-Tryon, 2007	5B	*LHY* mRNA	LL 12:12	toc1-2	
[34] Martin-Tryon, 2007	5C	*TOC1* mRNA	LL 12:12	Col	
[34] Martin-Tryon, 2007	5C	*TOC1* mRNA	LL 12:12	gi-201	
[34] Martin-Tryon, 2007	5C	*TOC1* mRNA	LL 12:12	toc1-2	
[34] Martin-Tryon, 2007	5D	*GI* mRNA	LL 12:12	Col	
[34] Martin-Tryon, 2007	5D	*GI* mRNA	LL 12:12	gi-201	
[34] Martin-Tryon, 2007	5D	*GI* mRNA	LL 12:12	toc1-2	
[35] Mas, 2003	2A	TOC1 pr	LD 12:12	TMG	
[35] Mas, 2003	2B	*TOC1* mRNA	LD 12:12	TMG	
[35] Mas, 2003	2C	TOC1 pr	LD 12:12	ztl-1 TMG	
[35] Mas, 2003	2D	*TOC1* mRNA	LD 12:12	ztl-1 TMG	
[35] Mas, 2003	3A	TOC1 pr	LL 12:12	wt, TMG	
[35] Mas, 2003	3B	*TOC1* mRNA	LL 12:12	wt, TMG	
[36] Matsushika, 2000	7A	*PRR9* mRNA	LD 16:8	wt	
[36] Matsushika, 2000	7A	*PRR7* mRNA	LD 16:8	wt	
[36] Matsushika, 2000	7A	*PRR5* mRNA	LD 16:8	wt	
[36] Matsushika, 2000	7A	*PRR3* mRNA	LD 16:8	wt	
[36] Matsushika, 2000	7A	*PRR1* mRNA	LD 16:8	wt	
[36] Matsushika, 2000	7B	*PRR9* mRNA	LD 12:12	wt	
[36] Matsushika, 2000	7B	*PRR7* mRNA	LD 12:12	wt	

Ref.	Fig.	Reporter	Light	Mutant / Comment	Weight
[36] Matsushika, 2000	7B	*PRR5* mRNA	LD 12:12	wt	
[36] Matsushika, 2000	7B	*PRR3* mRNA	LD 12:12	wt	
[36] Matsushika, 2000	7B	*PRR1* mRNA	LD 12:12	wt	
[36] Matsushika, 2000	7C	*PRR9* mRNA	LD 8:16	wt	
[36] Matsushika, 2000	7C	*PRR7* mRNA	LD 8:16	wt	
[36] Matsushika, 2000	7C	*PRR5* mRNA	LD 8:16	wt	
[36] Matsushika, 2000	7C	*PRR3* mRNA	LD 8:16	wt	
[36] Matsushika, 2000	7C	*PRR1* mRNA	LD 8:16	wt	
[37] Matsushika, 2002	3B	*PRR9* mRNA	LL 12:12	P9-ox	3
[37] Matsushika, 2002	3B	*PRR9* mRNA	LL 12:12	wt, Col	
[37] Matsushika, 2002	3B	*PRR7* mRNA	LL 12:12	P9-ox	3
[37] Matsushika, 2002	3B	*PRR7* mRNA	LL 12:12	wt, Col	
[37] Matsushika, 2002	3B	*PRR5* mRNA	LL 12:12	P9-ox	3
[37] Matsushika, 2002	3B	*PRR5* mRNA	LL 12:12	wt, Col	
[37] Matsushika, 2002	3B	*PRR1* mRNA	LL 12:12	P9-ox	3
[37] Matsushika, 2002	3B	*PRR1* mRNA	LL 12:12	wt, Col	
[37] Matsushika, 2002	4A	*LHY* mRNA	LL 12:12	P9-ox	3
[37] Matsushika, 2002	4A	*LHY* mRNA	LL 12:12	wt, Col	
[37] Matsushika, 2002	4B	*ELF3* mRNA	LL 12:12	P9-ox	3
[37] Matsushika, 2002	4B	*ELF3* mRNA	LL 12:12	wt, Col	
[37] Matsushika, 2002	4A	*CCA1* mRNA	LL 12:12	P9-ox	3
[37] Matsushika, 2002	4A	*CCA1* mRNA	LL 12:12	wt, Col	
[38] McWatters, 2007	6G	*ELF4* mRNA	LD 12:12	wt C24	
[38] McWatters, 2007	6G	*ELF4* mRNA	LD 12:12	toc1-1	
[38] McWatters, 2007	S2A	*CCA1* mRNA	LL 12:12	Ws	
[38] McWatters, 2007	S2A	*CCA1* mRNA	LL 12:12	elf4-1	
[38] McWatters, 2007	S2B	*LHY* mRNA	LL 12:12	Ws	
[38] McWatters, 2007	S2B	*LHY* mRNA	LL 12:12	elf4-1	
[38] McWatters, 2007	S2D	*TOC1* mRNA	LL 12:12	Ws	
[38] McWatters, 2007	S2D	*TOC1* mRNA	LL 12:12	elf4-1	
[38] McWatters, 2007	S1A	*ELF4* mRNA	LL 12:12	Ws	
[38] McWatters, 2007	S1A	*ELF4* mRNA	LL 12:12	ELF4-ox-11	
[39] Mizoguchi, 2002	7C	*GI* mRNA	LD 16:8	Ler	
[39] Mizoguchi, 2002	7C	*GI* mRNA	LD 16:8	lhy-12	
[39] Mizoguchi, 2002	7C	*GI* mRNA	LD 16:8	cca1-1	
[39] Mizoguchi, 2002	7D	*GI* mRNA	LD 16:8	cca1-1;lhy-12	
[39] Mizoguchi, 2002	7E	*TOC1* mRNA	LD 16:8	Ler	
[39] Mizoguchi, 2002	7E	*TOC1* mRNA	LD 16:8	lhy-12	
[39] Mizoguchi, 2002	7E	*TOC1* mRNA	LD 16:8	cca1-1	
[39] Mizoguchi, 2002	7F	*TOC1* mRNA	LD 16:8	cca1-1;lhy-12	
[39] Mizoguchi, 2002	7K	*LHY* mRNA	LL 16:8	Ler	
[39] Mizoguchi, 2002	7K	*LHY* mRNA	LL 16:8	gi-3	
[39] Mizoguchi, 2002	7L	*CCA1* mRNA	LL 16:8	Ler	
[39] Mizoguchi, 2002	7L	*CCA1* mRNA	LL 16:8	gi-3	
[39] Mizoguchi, 2002	6C	*GI* mRNA	LL 16:8	Ler	
[39] Mizoguchi, 2002	6C	*GI* mRNA	LL 16:8	lhy-12	
[39] Mizoguchi, 2002	6C	*GI* mRNA	LL 16:8	cca1-1	
[39] Mizoguchi, 2002	6G	*TOC1* mRNA	LL 16:8	Ler	
[39] Mizoguchi, 2002	6G	*TOC1* mRNA	LL 16:8	lhy-12	
[39] Mizoguchi, 2002	6G	*TOC1* mRNA	LL 16:8	cca1-1	
[39] Mizoguchi, 2002	6D	*GI* mRNA	LL 16:8	lhy-12;cca1-1	
[39] Mizoguchi, 2002	6H	*TOC1* mRNA	LL 16:8	lhy-12;cca1-1	
[39] Mizoguchi, 2002	2C	*CCA1* mRNA	LL 16:8	wt	
[39] Mizoguchi, 2002	2C	*CCA1* mRNA	LL 16:8	lhy-12	

Ref.	Fig.	Reporter	Light	Mutant / Comment	Weight
[39] Mizoguchi, 2002	2D	*LHY* mRNA	LL 16:8	wt	
[39] Mizoguchi, 2002	2D	*LHY* mRNA	LL 16:8	cca1-1	
[40] Nakamichi, 2003	1	*PRR9* mRNA	LD 12:12	wt	
[40] Nakamichi, 2003	1	*PRR7* mRNA	LD 12:12	wt	
[40] Nakamichi, 2003	1	*PRR5* mRNA	LD 12:12	wt	
[40] Nakamichi, 2003	1	*PRR1* mRNA	LD 12:12	wt	
[40] Nakamichi, 2003	3	*PRR1* mRNA	DD 12:12	wt	
[40] Nakamichi, 2003	3	*PRR5* mRNA	DD 12:12	wt	
[40] Nakamichi, 2003	3	*PRR7* mRNA	DD 12:12	wt	
[40] Nakamichi, 2003	3	*PRR7* mRNA	DD 12:12	wt	
[40] Nakamichi, 2003	3	*PRR9* mRNA	DD 12:12	wt	
[40] Nakamichi, 2003	4	*CCA1* mRNA	DD 12:!2	wt	
[40] Nakamichi, 2003	4	*LHY* mRNA	DD 12:12	wt	
[41] Nakamichi, 2005	4A	*CCA1* mRNA	LL 12:12	wt, Col	
[41] Nakamichi, 2005	4A	*CCA1* mRNA	LL 12:12	prr5-11;prr7-11	
[41] Nakamichi, 2005	4B	*LHY* mRNA	LL 12:12	wt, Col	
[41] Nakamichi, 2005	4B	*LHY* mRNA	LL 12:12	prr5-11;prr7-11	
[41] Nakamichi, 2005	4C	*TOC1* mRNA	LL 12:12	wt, Col	
[41] Nakamichi, 2005	4C	*TOC1* mRNA	LL 12:12	prr5-11;prr7-11	
[41] Nakamichi, 2005	5A	*CCA1* mRNA	DD 12:12	wt, Col	
[41] Nakamichi, 2005	5A	*CCA1* mRNA	DD 12:12	prr5-11;prr7-11	
[41] Nakamichi, 2005	5B	*LHY* mRNA	DD 12:12	wt, Col	
[41] Nakamichi, 2005	5B	*LHY* mRNA	DD 12:12	prr5-11;prr7-11	
[41] Nakamichi, 2005	6A	*CCA1* mRNA	LD 12:12	wt, Col	
[41] Nakamichi, 2005	6A	*CCA1* mRNA	LD 12:12	prr5-11;prr7-11	
[41] Nakamichi, 2005	6B	*LHY* mRNA	LD 12:12	wt, Col	
[41] Nakamichi, 2005	6B	*LHY* mRNA	LD 12:12	prr5-11;prr7-11	
[41] Nakamichi, 2005	6C	*TOC1* mRNA	LD 12:12	wt, Col	
[41] Nakamichi, 2005	6C	*TOC1* mRNA	LD 12:12	prr5-11;prr7-11	
[41] Nakamichi, 2005	6E	*GI* mRNA	LD 12:12	wt, Col	
[41] Nakamichi, 2005	6E	*GI* mRNA	LD 12:12	prr5-11;prr7-11	
[42] Nakamichi, 2005b	5A	*CCA1* mRNA	LL 12:12	wt, Col	
[42] Nakamichi, 2005b	5A	*CCA1* mRNA	LL 12:12	prr9-10;prr7-11;prr5-11	
[42] Nakamichi, 2005b	5B	*TOC1* mRNA	LL 12:12	wt, Col	
[42] Nakamichi, 2005b	5B	*TOC1* mRNA	LL 12:12	prr9-10;prr7-11;prr5-11	
[42] Nakamichi, 2005b	5C	*GI* mRNA	LL 12:12	wt, Col	
[42] Nakamichi, 2005b	5C	*GI* mRNA	LL 12:12	prr9-10;prr7-11;prr5-11	
[42] Nakamichi, 2005b	6C	*TOC1* mRNA	LD 12:12	wt	
[43] Nakamichi, 2010	6A	*CCA1* mRNA	LD 12:12	wt	
[43] Nakamichi, 2010	6B	PRR9 pr	LD 12:12	prr5;prr7	
[43] Nakamichi, 2010	6A	PRR9 pr	LD 12:12	wt	
[43] Nakamichi, 2010	6A	PRR7 pr	LD 12:12	wt	
[43] Nakamichi, 2010	6C	PRR5 pr	LD 12:12	prr7;prr9	
[43] Nakamichi, 2010	6A	PRR5 pr	LD 12:12	wt	
[43] Nakamichi, 2010	6C	*LHY* mRNA	LD 12:12	prr7;prr9	
[43] Nakamichi, 2010	6B	*LHY* mRNA	LD 12:12	prr5;prr7	
[43] Nakamichi, 2010	6A	*LHY* mRNA	LD 12:12	wt	
[43] Nakamichi, 2010	6B	*CCA1* mRNA	LD 12:12	prr5;prr7	
[43] Nakamichi, 2010	6C	*CCA1* mRNA	LD 12:12	prr7;prr9	
[44] Nakamichi, 2012	S4B	*LHY* mRNA	LL 12:12	wt	
[44] Nakamichi, 2012	S4B	*LHY* mRNA	LL 12:12	P5-ox	3
[44] Nakamichi, 2012	S4B	*PRR9* mRNA	LL 12:12	wt	
[44] Nakamichi, 2012	S4B	*PRR7* mRNA	LL 12:12	wt	
[45] Niwa, 2007	6L	*GI* mRNA	LD 10:14	wt	

Ref.	Fig.	Reporter	Light	Mutant / Comment	Weight
[45] Niwa, 2007	6L	*GI* mRNA	LD 10:14	toc1-2;cca1-1	
[45] Niwa, 2007	6R	*GI* mRNA	LD 10:14	wt	
[45] Niwa, 2007	6R	*GI* mRNA	LD 10:14	toc1-2;cca1-1	
[45] Niwa, 2007	8	*GI* mRNA	LD 10:14	toc1-2;cca1-1;lhy-11	
[45] Niwa, 2007	8	*GI* mRNA	LD 10:14	cca1-1;lhy-11	
[45] Niwa, 2007	8	*GI* mRNA	LD 10:14	wt	
[46] Nusinow, 2011	1	*ELF3* mRNA	LL 12:12	wt	
[46] Nusinow, 2011	1	*ELF4* mRNA	LL 12:12	wt	
[46] Nusinow, 2011	1	*LUX* mRNA	LL 12:12	wt	
[46] Nusinow, 2011	S11	*ELF4* mRNA	LD 8:16	wt	
[46] Nusinow, 2011	S11	*ELF4* mRNA	LD 8:16	lhy-1 (-ox)	
[46] Nusinow, 2011	S11	*ELF3* mRNA	LD 8:16	wt	
[46] Nusinow, 2011	S11	*ELF3* mRNA	LD 8:16	lhy-1 (-ox)	
[46] Nusinow, 2011	S11	*LUX* mRNA	LD 8:16	wt	
[46] Nusinow, 2011	S11	*LUX* mRNA	LD 8:16	lhy-1 (-ox)	
[47] Onai, 2005	1D	*GI* mRNA	LL 12:12	wt	
[47] Onai, 2005	1D	*GI* mRNA	LL 12:12	lux (pcl1-1)	
[47] Onai, 2005	1F	*TOC1* mRNA	LL 12:12	wt	
[47] Onai, 2005	1F	*TOC1* mRNA	LL 12:12	lux (pcl1-1)	
[47] Onai, 2005	1G	*ELF4* mRNA	LL 12:12	wt	
[47] Onai, 2005	1G	*ELF4* mRNA	LL 12:12	lux (pcl1-1)	
[47] Onai, 2005	1H	*CCA1* mRNA	LL 12:12	wt	
[47] Onai, 2005	1H	*CCA1* mRNA	LL 12:12	lux (pcl1-1)	
[47] Onai, 2005	1I	*LHY* mRNA	LL 12:12	wt	
[47] Onai, 2005	1I	*LHY* mRNA	LL 12:12	lux (pcl1-1)	
[47] Onai, 2005	6A	*LUX* mRNA	LL 12:12	wt	
[47] Onai, 2005	6A	*LUX* mRNA	LL 12:12	lux (pcl1-1)	
[47] Onai, 2005	6G	*LUX* mRNA	LL 12:12	wt	
[47] Onai, 2005	6G	*LUX* mRNA	LL 12:12	lux (pcl1-1)	
[47] Onai, 2005	6G	*LUX* mRNA	LL 12:12	LUX-ox	
[47] Onai, 2005	7A	*LUX* mRNA	LL 12:12	wt	
[47] Onai, 2005	7A	*LUX* mRNA	LL 12:12	lux (pcl1-1)	
[47] Onai, 2005	7A	*LUX* mRNA	LL 12:12	LUX-ox	
[47] Onai, 2005	7B	*GI* mRNA	LL 12:12	wt	
[47] Onai, 2005	7B	*GI* mRNA	LL 12:12	lux (pcl1-1)	
[47] Onai, 2005	7B	*GI* mRNA	LL 12:12	LUX-ox	
[47] Onai, 2005	7C	*TOC1* mRNA	LL 12:12	wt	
[47] Onai, 2005	7C	*TOC1* mRNA	LL 12:12	lux (pcl1-1)	
[47] Onai, 2005	7C	*TOC1* mRNA	LL 12:12	LUX-ox	
[47] Onai, 2005	7D	*ELF4* mRNA	LL 12:12	wt	
[47] Onai, 2005	7D	*ELF4* mRNA	LL 12:12	lux (pcl1-1)	
[47] Onai, 2005	7D	*ELF4* mRNA	LL 12:12	LUX-ox	
[47] Onai, 2005	7E	*CCA1* mRNA	LL 12:12	wt	
[47] Onai, 2005	7E	*CCA1* mRNA	LL 12:12	lux (pcl1-1)	
[47] Onai, 2005	7E	*CCA1* mRNA	LL 12:12	LUX-ox	
[47] Onai, 2005	7F	*LHY* mRNA	LL 12:12	wt	
[47] Onai, 2005	7F	*LHY* mRNA	LL 12:12	lux (pcl1-1)	
[47] Onai, 2005	7F	*LHY* mRNA	LL 12:12	LUX-ox	
[48] Park, 1999	3A	*GI* mRNA	LL 12:12	wt, Col	
[48] Park, 1999	3A	*GI* mRNA	LL 12:12	gi-1	
[48] Park, 1999	3A	*GI* mRNA	LL 12:12	gi-2	
[48] Park, 1999	3A	*CCA1* mRNA	LL 12:12	wt, Col	
[48] Park, 1999	3A	*CCA1* mRNA	LL 12:12	gi-1	
[48] Park, 1999	3A	*CCA1* mRNA	LL 12:12	gi-2	

Ref.	Fig.	Reporter	Light	Mutant / Comment	Weight
[48] Park, 1999	3A	*LHY* mRNA	LL 12:12	wt, Col	
[48] Park, 1999	3A	*LHY* mRNA	LL 12:12	gi-1	
[48] Park, 1999	3A	*LHY* mRNA	LL 12:12	gi-2	
[48] Park, 1999	3B	*LHY* mRNA	DD 12:12	wt, Col	
[48] Park, 1999	3B	*LHY* mRNA	DD 12:12	gi-1	
[48] Park, 1999	3B	*LHY* mRNA	DD 12:12	gi-2	
[48] Park, 1999	3B	*GI* mRNA	DD 12:12	wt, Col	
[48] Park, 1999	3B	*GI* mRNA	DD 12:12	gi-1	
[48] Park, 1999	3B	*GI* mRNA	DD 12:12	gi-2	
[49] Pokhilko, 2012	2A	*TOC1* mRNA	LD 12:12	wt	
[49] Pokhilko, 2012	2A	*TOC1* mRNA	LD 12:12	cca1;lhy	
[49] Pokhilko, 2012	2B	*LUX* mRNA	LD 12:12	wt	
[49] Pokhilko, 2012	2B	*LUX* mRNA	LD 12:12	cca1;lhy	
[49] Pokhilko, 2012	3C	*TOC1* mRNA	LD 12:12	cca1;lhy;gi	
[49] Pokhilko, 2012	3C	*TOC1* mRNA	LD 12:12	cca1;lhy	
[49] Pokhilko, 2012	3D	*LUX* mRNA	LD 12:12	cca1;lhy;gi	
[49] Pokhilko, 2012	3D	*LUX* mRNA	LD 12:12	cca1;lhy	
[49] Pokhilko, 2012	5A	*CCA1* mRNA	LD 12:12	wt	
[49] Pokhilko, 2012	5A	*CCA1* mRNA	LD 12:12	toc1	
[49] Pokhilko, 2012	5A	*CCA1* mRNA	LD 12:12	T-ox	
[49] Pokhilko, 2012	5B	*LHY* mRNA	LD 12:12	wt	
[49] Pokhilko, 2012	5B	*LHY* mRNA	LD 12:12	toc1	
[49] Pokhilko, 2012	5B	*LHY* mRNA	LD 12:12	T-ox	
[50] Rawat, 2011	4B	RVE8 pr	LL 12:12	wt	
[50] Rawat, 2011	6A	*CCA1* mRNA	LL 12:12	wt Col	
[50] Rawat, 2011	6A	*CCA1* mRNA	LL 12:12	rve8-1	
[50] Rawat, 2011	6A	*CCA1* mRNA	LL 12:12	RVE8-ox	
[50] Rawat, 2011	6B	*LHY* mRNA	LL 12:12	wt Col	
[50] Rawat, 2011	6B	*LHY* mRNA	LL 12:12	rve8-1	
[50] Rawat, 2011	6B	*LHY* mRNA	LL 12:12	RVE8-ox	
[50] Rawat, 2011	6C	*TOC1* mRNA	LL 12:12	wt Col	
[50] Rawat, 2011	6C	*TOC1* mRNA	LL 12:12	rve8-1	
[50] Rawat, 2011	6C	*TOC1* mRNA	LL 12:12	RVE8-ox	
[50] Rawat, 2011	6D	*CCA1* mRNA	LL 12:12	rve8-1;RVE8-ox	
[50] Rawat, 2011	6E	*LHY* mRNA	LL 12:12	rve8-1;RVE8-ox	
[50] Rawat, 2011	6F	*TOC1* mRNA	LL 12:12	rve8-1;RVE8-ox	
[50] Rawat, 2011	8C	*PRR5* mRNA	LL 12:12	wt Col	
[50] Rawat, 2011	8C	*PRR5* mRNA	LL 12:12	rve8-1	
[50] Rawat, 2011	8C	*PRR5* mRNA	LL 12:12	RVE8-ox	
[50] Rawat, 2011	8D	*TOC1* mRNA	LL 12:12	wt Col	
[50] Rawat, 2011	8D	*TOC1* mRNA	LL 12:12	rve8-1	
[50] Rawat, 2011	8D	*TOC1* mRNA	LL 12:12	RVE8-ox	
[50] Rawat, 2011	8G	*RVE8* mRNA	LL 12:12?	Col	
[50] Rawat, 2011	8G	*RVE8* mRNA	LL 12:12?	prr9;prr7;prr5	
[50] Rawat, 2011	8E	*PRR7* mRNA	LL 12:12	wt Col	
[50] Rawat, 2011	8E	*PRR7* mRNA	LL 12:12	rve8-1	
[50] Rawat, 2011	8E	*PRR7* mRNA	LL 12:12	RVE8-ox	
[50] Rawat, 2011	S1A	*RVE8* mRNA	LL 12:12	Col	
[50] Rawat, 2011	S1A	*RVE8* mRNA	LL 12:12	RVE8-ox	
[50] Rawat, 2011	S1B	*RVE8* mRNA	LL 12:12	rve8-1	
[51] Sato, 2002	2B	*GI* mRNA	LL 12:12	wt, Col	
[51] Sato, 2002	2B	*GI* mRNA	LL 12:12	P5-ox	3
[51] Sato, 2002	3	*CCA1* mRNA	LL 12:12	wt, Col	
[51] Sato, 2002	3	*CCA1* mRNA	LL 12:12	P5-ox	3

Ref.	Fig.	Reporter	Light	Mutant / Comment	Weight
[51] Sato, 2002	3	*LHY* mRNA	LL 12:12	wt, Col	
[51] Sato, 2002	3	*LHY* mRNA	LL 12:12	P5-ox	3
[51] Sato, 2002	4	*PRR9* mRNA	LL 12:12	wt, Col	
[51] Sato, 2002	4	*PRR9* mRNA	LL 12:12	P5-ox	3
[51] Sato, 2002	4	*PRR7* mRNA	LL 12:12	wt, Col	
[51] Sato, 2002	4	*PRR7* mRNA	LL 12:12	P5-ox	3
[51] Sato, 2002	4	*PRR5* mRNA	LL 12:12	wt, Col	
[51] Sato, 2002	4	*PRR5* mRNA	LL 12:12	P5-ox	3
[51] Sato, 2002	4	*PRR1* mRNA	LL 12:12	wt, Col	
[51] Sato, 2002	4	*PRR1* mRNA	LL 12:12	P5-ox	3
[52] Somers, 2004	9A	*CCA1* mRNA	LD 12:12	wt	
[52] Somers, 2004	9A	*CCA1* mRNA	LD 12:12	Z-ox	
[52] Somers, 2004	9A	*CCA1* mRNA	LD 12:12	ztl-1	
[52] Somers, 2004	9A	*CCA1* mRNA	LD 12:12	ztl-2	
[52] Somers, 2004	9A	*CCA1* mRNA	LD 12:12	ztl-3	
[52] Somers, 2004	9A	*CCA1* mRNA	LD 12:12	wt	
[52] Somers, 2004	9A	*CCA1* mRNA	LL 12:12	wt, Col	
[52] Somers, 2004	9A	*CCA1* mRNA	LL 12:12	wt, C24	
[52] Somers, 2004	9A	*CCA1* mRNA	LL 12:12	ztl-1, C24	
[52] Somers, 2004	9A	*CCA1* mRNA	LL 12:12	ztl-2, C24	
[52] Somers, 2004	9A	*CCA1* mRNA	LL 12:12	ztl-3, Col	
[52] Somers, 2004	9A	*CCA1* mRNA	LL 12:12	Z-ox	
[52] Somers, 2004	9B	*TOC1* mRNA	LL 12:12	wt, C24	
[52] Somers, 2004	9B	*TOC1* mRNA	LL 12:12	wt, Col	
[52] Somers, 2004	9B	*TOC1* mRNA	LL 12:12	ztl-1, C24	
[52] Somers, 2004	9B	*TOC1* mRNA	LL 12:12	ztl-2, C24	
[52] Somers, 2004	9B	*TOC1* mRNA	LL 12:12	ztl-3, Col	
[52] Somers, 2004	9B	*TOC1* mRNA	LL 12:12	Z-ox	
[52] Somers, 2004	9C	*ELF3* mRNA	LD 12:12	wt,	
[52] Somers, 2004	9C	*ELF3* mRNA	LD 12:12	Z-ox	
[52] Somers, 2004	9C	*ELF3* mRNA	LD 12:12	ztl-1	
[52] Somers, 2004	9C	*ELF3* mRNA	LD 12:12	ztl-2	
[52] Somers, 2004	9C	*ELF3* mRNA	LD 12:12	ztl-3	
[52] Somers, 2004	9C	*ELF3* mRNA	LL 12:12	wt, Col	
[52] Somers, 2004	9C	*ELF3* mRNA	LL 12:12	wt, C24	
[52] Somers, 2004	9C	*ELF3* mRNA	LL 12:12	ztl-1, C24	
[52] Somers, 2004	9C	*ELF3* mRNA	LL 12:12	ztl-2, C24	
[52] Somers, 2004	9C	*ELF3* mRNA	LL 12:12	ztl-3, Col	
[52] Somers, 2004	9C	*ELF3* mRNA	LL 12:12	Z-ox	
[53] Song, 2005	1B	*LHY* mRNA	LD 12:12	wt LHY:luc	
[53] Song, 2005	1B	*LHY* mRNA	LD 12:12	det1-1 LHY:luc	
[53] Song, 2005	1D	LHY pr	LD 12:12	wt	
[53] Song, 2005	1D	LHY pr	LD 12:12	det1-1	
[53] Song, 2005	4B	LHY pr	LD 12:12	lhy-1	
[53] Song, 2005	4B	LHY pr	LD 12:12	lhy-1;det1-1	
[54] Song, 2012	4C	*LHY* mRNA	LD 16:8	Col	
[54] Song, 2012	4C	*LHY* mRNA	LD 16:8	elf3-1	
[54] Song, 2012	6C	LHY pr	LD 16:8	elf3-1	
[54] Song, 2012	6D	LHY pr	LD 16:8	wt	
[55] Wang, 1998	6	*CCA1* mRNA	LL 12:12	wt	
[55] Wang, 1998	6	*CCA1* mRNA	LL 12:12	C-ox	
[55] Wang, 1998	6	*LHY* mRNA	LL 12:12	wt	
[55] Wang, 1998	6	*LHY* mRNA	LL 12:12	C-ox	
[56] Wang, 2010	3B	TOC1 pr	LD 12:12	prr5-1	

Ref.	Fig.	Reporter	Light	Mutant / Comment	Weight
[56] Wang, 2010	3B	TOC1 pr	LD 12:12	wt	
[56] Wang, 2010	3D	*TOC1* mRNA	LD 12:12	prr5-1	
[56] Wang, 2010	3D	*TOC1* mRNA	LD 12:12	wt, TMG	
[57] Yakir, 2009	1C	CCA1 pr	LL 14:10	(quantified in GIMP)	
[57] Yakir, 2009	S2	CCA1 pr	LL 14:10		
[57] Yakir, 2009	2E	CCA1 pr	LL 14:10?	CCA1::CCA1-HA-YFP cca1-1	
[57] Yakir, 2009	2E	CCA1 pr	DD 14:10?	CCA1::CCA1-HA-YFP cca1-1	
[58] Yamashino, 2008	4C	*CCA1* mRNA	LD 12:12	wt, Col	
[58] Yamashino, 2008	4C	*CCA1* mRNA	LD 12:12	prr9;prr7;prr5	
[58] Yamashino, 2008	4D	*GI* mRNA	LD 12:12	wt, Col	
[58] Yamashino, 2008	4D	*GI* mRNA	LD 12:12	cca1;lhy;toc1	
[58] Yamashino, 2008	4D	*GI* mRNA	LD 12:12	prr9;prr7;prr5	
[58] Yamashino, 2008	5B	*CCA1* mRNA	LD 12:12	wt, Col	
[58] Yamashino, 2008	5B	*CCA1* mRNA	LD 12:12	prr9;prr7;prr5;toc1	
[58] Yamashino, 2008	5C	*GI* mRNA	LD 12:12	wt, Col	
[58] Yamashino, 2008	5C	*GI* mRNA	LD 12:12	cca1;lhy;toc1	
[58] Yamashino, 2008	5C	*GI* mRNA	LD 12:12	prr9;prr7;prr5;toc1	
[59] Yu, 2008	3A	*ELF3* mRNA	LD 8:16	cop1-4	
[59] Yu, 2008	3A	*ELF3* mRNA	LD 8:16	wt	
[59] Yu, 2008	3A	*ELF3* mRNA	LD 16:8	cop1-4	
[59] Yu, 2008	3A	*ELF3* mRNA	LD 16:8	wt	
[59] Yu, 2008	3B	*GI* mRNA	LD 8:16	cop1-4	
[59] Yu, 2008	3B	*GI* mRNA	LD 8:16	wt	
[59] Yu, 2008	3B	*GI* mRNA	LD 16:8	cop1-4	
[59] Yu, 2008	3B	*GI* mRNA	LD 16:8	wt	
[59] Yu, 2008	5C	E3 pr	LD 8:16	cop1-4, Col, nuc	
[59] Yu, 2008	5C	E3 pr	LD 8:16	wt, Col, nuc	
[59] Yu, 2008	5C	E3 pr	LD 16:8	cop1-4, Col, nuc	
[59] Yu, 2008	5C	E3 pr	LD 16:8	wt, Col, nuc	
[59] Yu, 2008	5F	*LHY* mRNA	LD 12:12	wt,	
[59] Yu, 2008	5F	*LHY* mRNA	LD 12:12	cop1-4	
[59] Yu, 2008	5F	*LHY* mRNA	LD 12:12	E3-ox	
[59] Yu, 2008	5F	*LHY* mRNA	LD 12:12	cop1-4;E3-ox	
[59] Yu, 2008	6E	GI pr	LD 8:16	cop1-4	
[59] Yu, 2008	6E	GI pr	LD 8:16	elf3-8	
[59] Yu, 2008	6E	GI pr	LD 8:16	wt, left panel	
[59] Yu, 2008	6E	GI pr	LD 8:16	wt, right panel	

Table 1. Data used for model fitting. Each row corresponds to a time course of either mRNA or protein concentration in some light condition, extracted from the listed publications. All time courses were given a weight $w_i = 1$ unless otherwise specified here. Increased weights were used where important results were otherwise found to be difficult to reproduce: *CCA1* oscillations in *toc1* [3], ZTL protein level in wt and mutants [26], interaction between *elf3* and *cca1;lhy* [6], period lengthening in *prr7;prr9* [9], small effects of PRR7-ox [10], small period shortening in PRR9-ox [37], and level changes and period preservation in PRR5-ox [44, 51]. Decreased weights were used for some DD data where the system became arrhythmic in wt [28]. Experimental data was obtained in many different light conditions, which were simulated in the model. LD, LL and DD refers to light/dark, constant light and constant dark, respectively. Numbers such as 12:12 refer to hours of light and dark per period during entrainment, and for LD also during measurements. RR is constant red light, which was simulated as LL. Data were not manipulated in any way (e.g. stitched, joined or normalized) before entering the costfunction described in Methods in the main text. All raw data are available from the website mentioned in main text.

References

[1] Alabadı D, Oyama T, Yanovsky MJ, Harmon FG, Más P, et al. (2001) Reciprocal regulation between TOC1 and LHY/CCA1 within the *Arabidopsis* circadian clock. Science 293: 880–883.

[2] Baudry A, Ito S, Song YH, Strait AA, Kiba T, et al. (2010) F-box proteins FKF1 and LKP2 act in concert with ZEITLUPE to control *Arabidopsis* clock progression. Plant Cell 22: 606–622.

[3] Dai S, Wei X, Pei L, Thompson RL, Liu Y, et al. (2011) BROTHER OF LUX ARRHYTHMO is a component of the *Arabidopsis* circadian clock. Plant Cell 23: 961–972.

[4] David KM, Armbruster U, Tama N, Putterill J (2006) *Arabidopsis* GIGANTEA protein is post-transcriptionally regulated by light and dark. FEBS lett 580: 1193–1197.

[5] Ding Z, Doyle MR, Amasino RM, Davis SJ (2007) A complex genetic interaction between *Arabidopsis thaliana* TOC1 and CCA1/LHY in driving the circadian clock and in output regulation. Genetics 176: 1501–1510.

[6] Dixon LE, Knox K, Kozma-Bognar L, Southern MM, Pokhilko A, et al. (2011) Temporal repression of core circadian genes is mediated through EARLY FLOWERING 3 in *Arabidopsis*. Curr Biol 21: 120–125.

[7] Edwards KD, Akman OE, Knox K, Lumsden PJ, Thomson AW, et al. (2010) Quantitative analysis of regulatory flexibility under changing environmental conditions. Mol Syst Biol 6: 424.

[8] Farinas B, Mas P (2011) Functional implication of the MYB transcription factor RVE8/LCL5 in the circadian control of histone acetylation. Plant J 66: 318–329.

[9] Farré EM, Harmer SL, Harmon FG, Yanovsky MJ, Kay SA (2005) Overlapping and distinct roles of PRR7 and PRR9 in the *Arabidopsis* circadian clock. Curr Biol 15: 47–54.

[10] Farré EM, Kay SA (2007) PRR7 protein levels are regulated by light and the circadian clock in *Arabidopsis*. Plant J 52: 548–560.

[11] Fowler S, Lee K, Onouchi H, Samach A, Richardson K, et al. (1999) GIGANTEA: a circadian clock-controlled gene that regulates photoperiodic flowering in *Arabidopsis* and encodes a protein with several possible membrane-spanning domains. EMBO J 18: 4679–4688.

[12] Fujiwara S, Wang L, Han L, Suh SS, Salomé PA, et al. (2008) Post-translational regulation of the *Arabidopsis* circadian clock through selective proteolysis and phosphorylation of pseudo-response regulator proteins. J Biol Chem 283: 23073–23083.

[13] Hazen SP, Schultz TF, Pruneda-Paz JL, Borevitz JO, Ecker JR, et al. (2005) LUX ARRHYTHMO encodes a MYB domain protein essential for circadian rhythms. Proc Natl Acad Sci USA 102: 10387–10392.

[14] Helfer A, Nusinow DA, Chow BY, Gehrke AR, Bulyk ML, et al. (2011) LUX ARRHYTHMO encodes a nighttime repressor of circadian gene expression in the *Arabidopsis* core clock. Curr Biol 21: 126–133.

[15] Herrero Serrano E (2011) A molecular basis of ELF3 action in the *Arabidopsis* circadian clock. Ph.D. thesis, Universität zu Köln.

[16] Hicks KA, Albertson TM, Wagner DR (2001) EARLY FLOWERING 3 encodes a novel protein that regulates circadian clock function and flowering in *Arabidopsis.* Plant Cell 13: 1281–1292.

[17] Hsu PY, Harmer SL (2012) Circadian phase has profound effects on differential expression analysis. PLoS One 7: e49853.

[18] Hsu PY, Devisetty UK, Harmer SL (2013) Accurate timekeeping is controlled by a cycling activator in *Arabidopsis.* eLife 2: e00473.

[19] Huang W, Pérez-García P, Pokhilko A, Millar A, Antoshechkin I, et al. (2012) Mapping the core of the *Arabidopsis* circadian clock defines the network structure of the oscillator. Science 336: 75–79.

[20] Ito S, Niwa Y, Nakamichi N, Kawamura H, Yamashino T, et al. (2008) Insight into missing genetic links between two evening-expressed pseudo-response regulator genes TOC1 and PRR5 in the circadian clock-controlled circuitry in *Arabidopsis thaliana.* Plant Cell Physiol 49: 201–213.

[21] Kaczorowski KA, Quail PH (2003) *Arabidopsis* PSEUDO-RESPONSE REGULATOR 7 is a signaling intermediate in phytochrome-regulated seedling deetiolation and phasing of the circadian clock. Plant Cell 15: 2654–2665.

[22] Kiba T, Henriques R, Sakakibara H, Chua NH (2007) Targeted degradation of PSEUDO-RESPONSE REGULATOR 5 by an SCFZTL complex regulates clock function and photomorphogenesis in *Arabidopsis thaliana.* Plant Cell 19: 2516–2530.

[23] Kikis EA, Khanna R, Quail PH (2005) ELF4 is a phytochrome-regulated component of a negative-feedback loop involving the central oscillator components CCA1 and LHY. Plant J 44: 300–313.

[24] Kim JY, Song HR, Taylor BL, Carré IA (2003) Light-regulated translation mediates gated induction of the *Arabidopsis* clock protein LHY. EMBO J 22: 935–944.

[25] Kim WY, Hicks KA, Somers DE (2005) Independent roles for EARLY FLOWERING 3 and ZEITLUPE in the control of circadian timing, hypocotyl length, and flowering time. Plant Physiol 139: 1557–1569.

[26] Kim WY, Fujiwara S, Suh SS, Kim J, Kim Y, et al. (2007) ZEITLUPE is a circadian photoreceptor stabilized by GIGANTEA in blue light. Nature 449: 356–360.

[27] Kim Y, Han S, Yeom M, Kim H, Lim J, et al. (2013) Balanced nucleocytosolic partitioning defines a spatial network to coordinate circadian physiology in plants. Dev Cell 26: 73 - 85.

[28] Kolmos E, Nowak M, Werner M, Fischer K, Schwarz G, et al. (2009) Integrating ELF4 into the circadian system through combined structural and functional studies. HFSP J 3: 350–366.

[29] Lau OS, Huang X, Charron JB, Lee JH, Li G, et al. (2011) Interaction of *Arabidopsis* DET1 with CCA1 and LHY in mediating transcriptional repression in the plant circadian clock. Mol Cell 43: 703–712.

[30] Li G, Siddiqui H, Teng Y, Lin R, Wan Xy, et al. (2011) Coordinated transcriptional regulation underlying the circadian clock in *Arabidopsis.* Nat Cell Biol 13: 616–622.

[31] Liu XL, Covington MF, Fankhauser C, Chory J, Wagner DR (2001) ELF3 encodes a circadian clock–regulated nuclear protein that functions in an *Arabidopsis* PHYB signal transduction pathway. Plant Cell 13: 1293–1304.

[32] Locke JC, Southern MM, Kozma-Bognár L, Hibberd V, Brown PE, et al. (2005) Extension of a genetic network model by iterative experimentation and mathematical analysis. Mol Syst Biol 1: 2005.0013.

[33] Lu SX, Webb CJ, Knowles SM, Kim SH, Wang Z, et al. (2012) CCA1 and ELF3 interact in the control of hypocotyl length and flowering time in *Arabidopsis.* Plant Physiol 158: 1079–1088.

[34] Martin-Tryon EL, Kreps JA, Harmer SL (2007) GIGANTEA acts in blue light signaling and has biochemically separable roles in circadian clock and flowering time regulation. Plant Physiol 143: 473–486.

[35] Más P, Kim WY, Somers DE, Kay SA, et al. (2003) Targeted degradation of TOC1 by ZTL modulates circadian function in *Arabidopsis thaliana.* Nature 426: 567–570.

[36] Matsushika A, Makino S, Kojima M, Mizuno T (2000) Circadian waves of expression of the APRR1/TOC1 family of pseudo-response regulators in *Arabidopsis thaliana*: insight into the plant circadian clock. Plant Cell Physiol 41: 1002–1012.

[37] Matsushika A, Imamura A, Yamashino T, Mizuno T (2002) Aberrant expression of the light-inducible and circadian-regulated APRR9 gene belonging to the circadian-associated APRR1/TOC1 quintet results in the phenotype of early flowering in *Arabidopsis thaliana.* Plant Cell Physiol 43: 833–843.

[38] McWatters HG, Kolmos E, Hall A, Doyle MR, Amasino RM, et al. (2007) ELF4 is required for oscillatory properties of the circadian clock. Plant Physiol 144: 391–401.

[39] Mizoguchi T, Wheatley K, Hanzawa Y, Wright L, Mizoguchi M, et al. (2002) LHY and CCA1 are partially redundant genes required to maintain circadian rhythms in *Arabidopsis.* Dev Cell 2: 629–641.

[40] Nakamichi N, Matsushika A, Yamashino T, Mizuno T (2003) Cell autonomous circadian waves of the APRR1/TOC1 quintet in an established cell line of *Arabidopsis thaliana.* Plant Cell Physiol 44: 360–365.

[41] Nakamichi N, Kita M, Ito S, Sato E, Yamashino T, et al. (2005) The *Arabidopsis* pseudo-response regulators, PRR5 and PRR7, coordinately play essential roles for circadian clock function. Plant Cell Physiol 46: 609–619.

[42] Nakamichi N, Kita M, Ito S, Yamashino T, Mizuno T (2005) PSEUDO-RESPONSE REGULATORS, PRR9, PRR7 and PRR5, together play essential roles close to the circadian clock of *Arabidopsis thaliana.* Plant Cell Physiol 46: 686–698.

[43] Nakamichi N, Kiba T, Henriques R, Mizuno T, Chua NH, et al. (2010) PSEUDO-RESPONSE REGULATORS 9, 7, and 5 are transcriptional repressors in the *Arabidopsis* circadian clock. Plant Cell 22: 594–605.

[44] Nakamichi N, Kiba T, Kamioka M, Suzuki T, Yamashino T, et al. (2012) Transcriptional repressor PRR5 directly regulates clock-output pathways. Proc Natl Acad Sci USA 109: 17123–17128.

[45] Niwa Y, Ito S, Nakamichi N, Mizoguchi T, Niinuma K, et al. (2007) Genetic linkages of the circadian clock-associated genes, TOC1, CCA1 and LHY, in the photoperiodic control of flowering time in *Arabidopsis thaliana.* Plant Cell Physiol 48: 925–937.

[46] Nusinow DA, Helfer A, Hamilton EE, King JJ, Imaizumi T, et al. (2011) The ELF4-ELF3-LUX complex links the circadian clock to diurnal control of hypocotyl growth. Nature 475: 398–402.

[47] Onai K, Ishiura M (2005) PHYTOCLOCK 1 encoding a novel GARP protein essential for the *Arabidopsis* circadian clock. Genes Cells 10: 963–972.

[48] Park DH, Somers DE, Kim YS, Choy YH, Lim HK, et al. (1999) Control of circadian rhythms and photoperiodic flowering by the *Arabidopsis* GIGANTEA gene. Science 285: 1579–1582.

[49] Pokhilko A, Fernández AP, Edwards KD, Southern MM, Halliday KJ, et al. (2012) The clock gene circuit in *Arabidopsis* includes a repressilator with additional feedback loops. Mol Syst Biol 8: 574.

[50] Rawat R, Takahashi N, Hsu PY, Jones MA, Schwartz J, et al. (2011) REVEILLE 8 and PSEUDO-REPONSE REGULATOR 5 form a negative feedback loop within the *Arabidopsis* circadian clock. PLoS Genet 7: e1001350.

[51] Sato E, Nakamichi N, Yamashino T, Mizuno T (2002) Aberrant expression of the *Arabidopsis* circadian-regulated APRR5 gene belonging to the APRR1/TOC1 quintet results in early flowering and hypersensitiveness to light in early photomorphogenesis. Plant Cell Physiol 43: 1374–1385.

[52] Somers DE, Kim WY, Geng R (2004) The F-box protein ZEITLUPE confers dosage-dependent control on the circadian clock, photomorphogenesis, and flowering time. Plant Cell 16: 769–782.

[53] Song HR, Carré IA (2005) DET1 regulates the proteasomal degradation of LHY, a component of the *Arabidopsis* circadian clock. Plant Mol Biol 57: 761–771.

[54] Song HR (2012) Interaction between the late elongated hypocotyl (LHY) and early flowering 3 (ELF3) genes in the *Arabidopsis* circadian clock. Genes Genomics 34: 329–337.

[55] Wang ZY, Tobin EM, et al. (1998) Constitutive expression of the CIRCADIAN CLOCK ASSOCIATED 1 (CCA1) gene disrupts circadian rhythms and suppresses its own expression. Cell 93: 1207–1218.

[56] Wang L, Fujiwara S, Somers DE (2010) PRR5 regulates phosphorylation, nuclear import and subnuclear localization of TOC1 in the *Arabidopsis* circadian clock. EMBO J 29: 1903–1915.

[57] Yakir E, Hilman D, Kron I, Hassidim M, Melamed-Book N, et al. (2009) Posttranslational regulation of CIRCADIAN CLOCK ASSOCIATED 1 in the circadian oscillator of *Arabidopsis.* Plant Physiol 150: 844–857.

[58] Yamashino T, Ito S, Niwa Y, Kunihiro A, Nakamichi N, et al. (2008) Involvement of *Arabidopsis* clock-associated pseudo-response regulators in diurnal oscillations of gene expression in the presence of environmental time cues. Plant Cell Physiol 49: 1839–1850.

[59] Yu JW, Rubio V, Lee NY, Bai S, Lee SY, et al. (2008) COP1 and ELF3 control circadian function and photoperiodic flowering by regulating GI stability. Mol Cell 32: 617–630.
