# Supplementary material for: Rethinking Transcriptional Activation in the Arabidopsis Circadian Clock
Source: PLoS Comput Biol. 2014 Jul 17;10(7):e1003705. doi: 10.1371/journal.pcbi.1003705 (PMC4102396; doi:10.1371/journal.pcbi.1003705)
Supplement: Text S1 — Additional results and equations. Further information about the modelling, covering details about the evening complex, the regulation of NOX, the splitting of CCA1 and LHY into two variables, the localization of TOC1 and PRR5, and the removal of the ABA circuit, and some light inputs. This text also includes the differential equations of the model, a table of periods comparing model to experiments, and the parameter values of the eight best fitted parameter sets. The equations are presented in their wild type forms, which do not include modifications used when simulating the many different mutants. (PDF) [file pcbi.1003705.s009.pdf]

Text S1: Additional results and equations

Additional Results

Evening complex modelling details

The equations describing EC are practically identical between P2011 and P2012. A minor difference is that several parameters for ELF3 degradation by COP1 are merged in the latter model. In the P2011–P2012 equations, EC is formed in two steps: ELF3 and ELF4 form a complex, which then binds with LUX to form EC. The models also describe the formation of a complex between GI and ELF3, whereby GI facilitates the degradation of ELF3 through COP1, based on observed interactions between the three proteins [1].

To reformulate the EC equations in order to take into account the possible redundancy between NOX and LUX and the ability of ELF3-ox to rescue EC function in *elf4*, as described in the main text, we had to simplify the description of EC formation to avoid a combinatorial explosion of reaction paths between sub-complexes.

In our model, the formation of EC begins with the homodimerization of ELF4 [2]. This homodimer, which is given its own variable, is in turn bound in the ELF3-ELF4 complex [3]. We assumed that delays caused by e.g. the time needed for LUX to bind to ELF3 (with or without ELF4) can at least partly be transferred to other steps in the model. Thus we modelled EC activity directly as a function of the levels of ELF3, ELF3-ELF4, LUX and NOX.

The equations for COP1, which regulates degradation of ELF3 and GI, were left unchanged from the P2012 model, but the action of COP1 was by necessity adapted to the altered EC. ELF3 is strongly localized to the nucleus [4] and unlike P2012 our model only considers its nuclear fraction. In our model, COP1 acts on ELF3 through its nuclear "day" and "night" forms, and in addition ELF3 degradation is directly increased by nuclear GI; the ELF3-GI complex of P2012 has been removed. Cytosolic and nuclear GI are no longer handled as being in a quasi-steady state, but instead are given their own variables. The degradation of GI by COP1 is mediated by ELF3 [1]. This is reflected in the equations, where nuclear GI is degraded by the two forms of COP1 only when ELF3 is present. This is qualitatively similar to the structure of the P2012 model, even though the equations are different and may allow different dynamics.

In simulations of the *elf3-4* knockout mutant, the clock loses rhythmicity in LL, and the expression levels of *PRR9*, *PRR7*, *GI* and *TOC1* peak at the wrong time in LD compared with experiments (Figure 1). This is true for F2014 as well as P2011–P2012, even though the predicted expression profiles are different. Simulating the mutants as having ELF3 function at 20% of its normal value led to expression profiles in better agreement with the data for *GI* and *TOC1* and possibly also *PRR9*. However, the *elf3-4* mutant has an early stop codon which is expected to lead to a total loss of function [5].

One way to resolve this conflict could be to assume that LUX and NOX retain some function in the absence of ELF3. However, when we allowed LUX and NOX to act as EC in the absence of ELF3, with or without ELF4, the resulting expression of GI was low and out of phase with experiments. When instead we assumed that ELF4 on its own is able to interact with LUX and NOX, the level of EC became constant rather than oscillating. We conclude that if, as expected, the *elf3-4* mutant leads to a total loss of function, EC function may be rescued by some other clock component which is partly redundant with ELF3.

Furthermore, strong rhythmicity was seen in *ELF3* mRNA in LD in the loss-of-function

elf3-1 and *elf3-2* mutants [5], even though *CCA1* and *LHY* are repressed and only weakly oscillating in *elf3-1* [6]. Even simulations with ELF3 at 20% function showed only weak *ELF3* rhythms, which suggests that *ELF3* transcription is regulated by a clock component other than CCA1 and LHY, probably one with daytime expression and preserved rhythms in *elf3*. The best candidate represented in the model is PRR9. Making PRR9 a repressor of *ELF3* transcription did not work well in the current model, possibly because the predicted timing of the weakly constrained PRR9 protein was incorrect, but we think this predicted connection is worth exploring in future work.

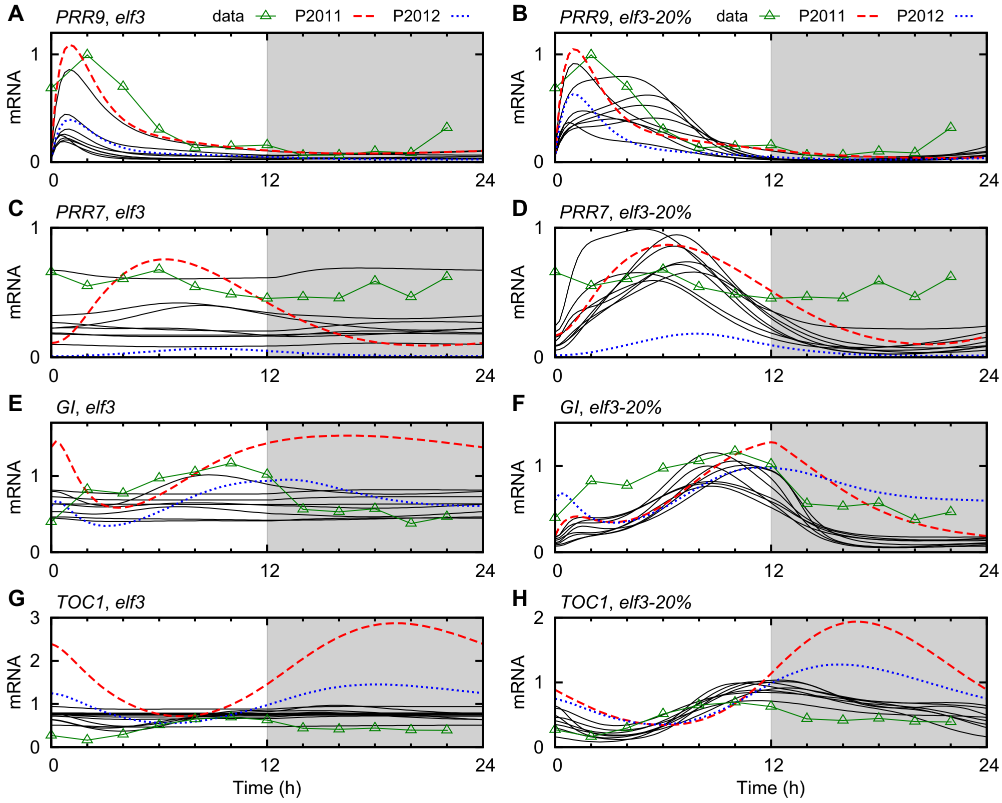

Figure 1. Retained ELF3 function in *elf3* mutants. Comparison between modelling the *elf3-4* mutant as a complete loss of function (left panels) and as lowering ELF3 production to 20% of its normal value (right panels). The F2014 model (solid black lines) is compared with data from Dixon *et al.* [7] (green triangles) and the models P2011 (dashed red lines) and P2012 (dotted blue lines) in LD. (A-B) *PRR9* expression, (C-D) *PRR7* expression, (E-F) *GI* expression, and (G-H) *TOC1* expression. Levels were normalized to a peak value of 1 in wt.

Additional input into NOX

Having only CCA1 and LHY as inputs to *NOX* was not sufficient to reproduce all *NOX* expression data; *NOX* is rhythmic in constant light in the *cca1-11;lhy-21* double mutant [8]. Our interpretation is that *NOX* should have at least one more repressor in the model. Among the clock components in our model, the expression profiles of PRR7 or PRR9 in LD provided

the closest match to what we expected of an additional repressor. For computational reasons, we did not fully explore the difference between using PRR7 or PRR9 as the repressor of NOX transcription in the equations, but our initial attempts suggested that PRR7 may lead to a better fit. Hence, we included PRR7 as a transcriptional repressor in the equation for NOX. Figure **??**C, in the main text, shows the resulting expression profile, where the input from PRR7 is seen to modulate the shape and peak phase of *NOX* expression by reducing transcription around ZT 10.

CCA1 and LHY are modelled separately

Although CCA1 and LHY are closely related, highly coexpressed, and have some overlap in function, they are not redundant [9–11]. There are noticeable differences in their regulation, as only the *CCA1* promoter interacts with CHE (which is not represented in the current model due to a lack of experimental data) [12], which may also be true for NOX [8]. Furthermore, CCA1 is more important than LHY at lower temperatures for regulating the period, and vice versa at higher temperatures [13].

These facts, in conjunction with access to significant amounts of separate data for CCA1 and LHY, and for their mutants, led us to split the LHY/CCA1 module of previous models (L2005 to P2012) into two separate parts. Both parts contribute to the repression of the targets of the previous LHY/CCA1 in P2012. The difference between the two parts in our model lies only in the transcriptional regulation of *CCA1* and *LHY* themselves, not in their binding targets. In contrast to the *CCA1* promoter, the *LHY* promoter contains two predicted specific CCA1 binding elements [14, 15]. For this reason, we modelled only *LHY* as repressed by CCA1 and LHY. However, we cannot rule out that the interaction is activating, but the model agrees with experimental data that *CCA1* expression is lower in *lhy* whereas *LHY* expression is lower in *cca1* [16].

By modelling CCA1 and LHY separately we were able to include the interaction between the two proteins in the model. CCA1 and LHY are single MYB-domain transcription factors [17] and form both homodimers and heterodimers *in vivo* in order to bind DNA, which most likely requires two MYB-domains [18, 19]. In the model, we assumed that CCA1 and LHY may differ in their overall binding affinities, but not in any target-specific way. The heterodimer could be more or less active than the homodimers, but parameter fitting indicated that this freedom was not needed; hence, we removed it from the equations.

With the separation of *CCA1* and *LHY*, we hope to set forth a process of better distinguishing what the differences between them actually are. It is usually taken for granted that one trait of LHY must probably also be true for CCA1. Only on rare occasions is it explicitly said that it is not so, as in the case of CHE and NOX in relation to CCA1 and LHY. Another example relates to *CCA1* and *LHY* mRNA stability in dark/light, where results by Yakir *et al.* [20] and Kim *et al.* [21] are in direct contradiction.

Localization of TOC1 and PRR5

PRR5 plays a major part in translocating TOC1 to the nucleus, in addition to its role as a transcriptional repressor. In the absence of functional PRR5, the level of TOC1 is lower in the nucleus and higher in the cytosol than in the wild type. The total TOC1 protein level is lower, even though the *TOC1* mRNA level is unchanged, suggesting that PRR5 both localizes and stabilizes TOC1 [22].

Like TOC1, PRR5 is targeted for degradation by ZTL [23, 24], which is localized only to the cytosol [25]. Thus the model must include cycling of both TOC1 and PRR5 between the cytosol and the nucleus.

Due to the small amount of data at the protein level, we modelled this part of the system in a relatively simple way: TOC1 diffuses freely into the nucleus, but diffusion back into the cytosol is inhibited by nuclear PRR5. The PRR5 protein may diffuse between nucleus and cytosol, unaffected by TOC1. The model encourages stabilization of TOC1 by also allowing PRR5 to inhibit nuclear degradation of TOC1. However, if TOC1 is more stable in the nucleus than in the cytosol, such a mechanism may be unnecessary.

For some parameter sets, the model reproduces the qualitative level changes in *prr5* compatered with wt, but there is great variation between the parameter sets and the fit to data for total TOC1 protein is bad. (Figure 2) Likewise, neither the nuclear degradation rate nor the diffusion rate of TOC1 shows any clear pattern between the parameter sets. This difficulty in fitting the model was likely due to both the relatively small amount of data relevant to TOC1 localization and the large discrepancies in TOC1 peak timing between different data sets.

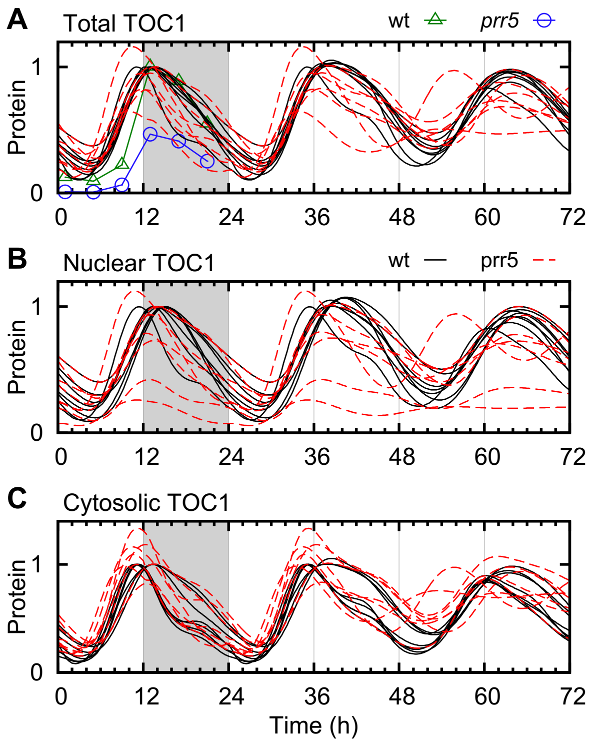

Figure 2. Localization of TOC1 protein. TOC1 protein simulated in wt (solid black lines) and *prr5* (dashed red lines), compared with data [22] for wt (green triangles) and *prr5* (blue circles). (A) Total TOC1 protein. (B) Nuclear TOC1 protein. (C) Cytosolic TOC1 protein. In each panel, the curves were normalized to a peak level of 1 in wt.

Removal of light inputs and components

We discarded several experimentally unmotivated or computationally unnecessary components and interactions compared with the P2012 model. This includes the removal of several

light inputs for which we could find no convincing evidence. Specifically, we removed the direct light dependence in the degradation rates of *CCA1* and *LHY* mRNA and of the PRR9, PRR5 and TOC1 proteins. In the case of PRR5, the light input was replaced by ZTL-dependent degradation [23,24]. The direct transcriptional light response of *GI* was also removed, since the degradation of EC by COP1 was sufficient to explain the experimentally observed rise in *GI* transcription in the morning.

We removed the hypothetical modified form of LHY/CCA1, LHY_{mod}. Its purpose in P2010–P2012 was to give a delayed positive input into PRR5, which proved to be redundant in our model where the rise in PRR5 in the afternoon is instead due to ceasing repression by CCA1 and LHY (see Figure **??**A and E in the main text).

An additional difference between our model and P2012 is our exclusion of equations related to ABA. The primary purpose of the ABA circuit was to introduce an output from the clock, and although this circuit feeds back into *TOC1*, is has very little impact on the dynamics of the clock when the ABA input level is kept at its normal value (Figure 3).

The removal of unmotivated parameters and addition of new clock components balanced out. In spite of the inclusion of *NOX* and *RVE8* and the separation of *CCA1* and *LHY*, our model reduces the number of parameters compared with P2012, as shown in Table **??** in the main text.

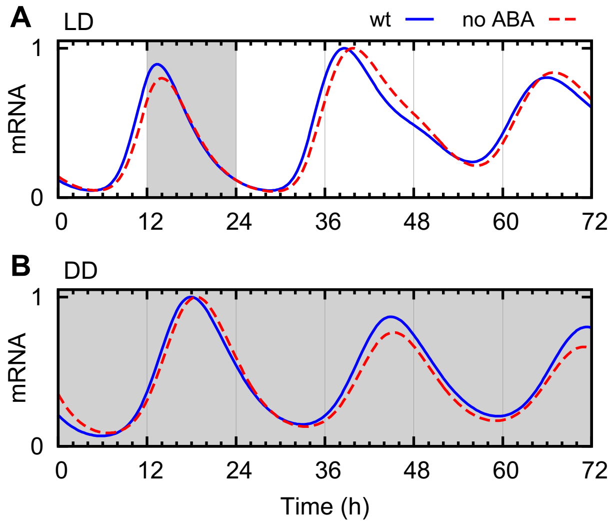

Figure 3. Limited feedback from ABA circuit to the clock in P2012. Normalized *TOC1* transcription in the P2012 model, with (solid blue lines) and without (dashed red lines) the ABA circuit connected to *TOC1* transcription. (A) in the transition from LD 12:12 to LL, and (B) in DD.

Model equations

We here describe the system of ordinary differential equations of the F2014 model. The dimensionless concentration levels of mRNA and protein of clock component X are denoted $c_X^{(m)}$ and c_X, respectively, where X is an abbreviated component name explained in Table 1. Non-subscript L and D denote light and darkness, respectively, where one is 0 when the other is 1. When localization of a protein X is included in the model, it is either nuclear, Xn, or cytosolic, Xc. However, with the nomenclature inherited from P2012, *COP1n* and *COP1d* both denote nuclear COP1 protein, in its day and night forms. For ELF4, d indicates a dimer.

Short	Component
P	dark accumulator
R	RVE8
C	CCA1
L	LHY
P9	PRR9
P7	PRR7
P5	PRR5
T	TOC1
E3	ELF3
E4	ELF4
E4d	ELF4 dimer
E34	ELF3-ELF4 complex
LUX	LUX
NOX	NOX
ZTL	ZTL
G	GI
ZG	ZTL-GI complex
COP1	COP1

Table 1. Symbols used in the equation system.

In order to simplify the equations, eqs. (1), (2), (3), (14), (17), (23), (26), (34) and (39) define some recurring expressions. LC is a weighted sum of CCA1 and LHY concentrations, used where both CCA1 and LHY repress transcription. LC_{common} is the common term in the regulation of *CCA1* and *LHY* transcription. $P5_{trans}$, T_{trans} and G_{trans} describe the cytosolic/nuclear translocation of their respective proteins. $E34_{prod}$ and ZG_{prod} are complex formation rates, and $E3_{deg}$ is the *E3* degradation rate that also applies to *E34*.

Parameters are named according to function. Parameters that govern transcriptional activation and repression are denoted by a and r, respectively. The symbol q is used for light-activated transcription, t for protein transport rates, m for degradation rates (protein and mRNA), and n for protein production (for COP1 only). Weights between components that play similar roles (in EC and LC) are denoted by f, and p is used for various parameters from P2012 for protein production, transport, degradation and complex formation.

The mRNA production terms are all based on the same general assumptions about how repressors and activators bind to DNA to regulate transcription. CCA1 and LHY are assumed to share binding sites, as are the PRR proteins, but otherwise the binding sites for different proteins are assumed to be independent. As in P2012, all repression terms are squared in the denominators to represent the unknown degree of cooperativity. Activators

have a corresponding term in the numerators, with a parameter describing the maximum level of activation relative to the unactivated state. The degradation rates of mRNAs always follow mass-action kinetics.

With few exceptions, the levels of mRNAs and proteins are arbitrary in the model as a change in the production rate could equally well be described as an opposite change in all binding affinities of the protein. The exceptions are those proteins that are involved in complexes, where the model has parameters to set the relative production rates. For other proteins, the maximum levels are determined by degradation and the regulation of production.

The expression for EC is designed such that it is limited by ELF3 and ELF3-ELF4 when LUX and NOX are high, and vice versa. What "high" means is defined by f_3 and f_4. It is assumed that LUX and ELF3-ELF4 are the most important players in the complex, and f_1, f_2 and f_6 allow NOX and ELF3 to also participate. For the difference between NOX and LUX, we separate the activity (numerator, f_6) from the saturation (denominator, f_2) to allow for the possibility that EC with NOX is a weaker repressor than EC with LUX. In contrast, the same expression with f_1 is used in both numerator and denominator because ELF3 is supposed to act like more dilute ELF3-ELF4.

The equations and parameter values are also available for download from `http://cbbp.thep.lu.se/activities/clocksim/`.

$$LC = (c_L + f_5 c_C) \tag{1}$$

$$LC_{common} = \frac{q_1 L c_P + 1}{1 + (r_1 c_{P9})^2 + (r_2 c_{P7})^2 + (r_3 c_{P5n})^2 + (r_4 c_{Tn})^2} \tag{2}$$

$$EC = \frac{(c_{LUX} + f_6 c_{NOX})(c_{E34} + f_1 c_{E3})}{1 + f_3(c_{LUX} + f_2 c_{NOX}) + f_4(c_{E34} + f_1 c_{E3})} \tag{3}$$

$$\frac{dc_L^{(m)}}{dt} = \frac{LC_{common}}{(1 + (r_{11} LC)^2)} - m_1 c_L^{(m)} \tag{4}$$

$$\frac{dc_L}{dt} = (L + m_4 D) c_L^{(m)} - m_3 c_L \tag{5}$$

$$\frac{dc_C^{(m)}}{dt} = LC_{common} - m_1 c_C^{(m)} \tag{6}$$

$$\frac{dc_C}{dt} = (L + m_4 D) c_C^{(m)} - m_3 c_C \tag{7}$$

$$\frac{dc_P}{dt} = p_7 D(1 - c_P) - m_{11} c_P L \tag{8}$$

$$\begin{aligned}\frac{dc_{P9}^{(m)}}{dt} &= q_3 c_P L - m_{12} c_{P9}^{(m)} \\ &+ \frac{1 + a_3 r_{33} c_R}{(1 + r_{33} c_R)(1 + (r_5 LC)^2)(1 + (r_6 EC)^2)(1 + (r_7 c_{Tn})^2)(1 + (r_{40} c_{P5n})^2)}\end{aligned} \tag{9}$$

$$\frac{dc_{P9}}{dt} = c_{P9}^{(m)} - m_{13} c_{P9} \tag{10}$$

$$\frac{dc_{P7}^{(m)}}{dt} = \frac{1}{(1 + (r_8 LC)^2)(1 + (r_9 EC)^2)(1 + (r_{10} c_{Tn})^2)(1 + (r_{40} c_{P5n})^2)} - m_{14} c_{P7}^{(m)} \tag{11}$$

$$\frac{dc_{P7}}{dt} = c_{P7}^{(m)} - (m_{15} + m_{23}D)c_{P7} \tag{12}$$

$$\frac{dc_{P5}^{(m)}}{dt} = \frac{1 + a_4 r_{34} c_R}{(1 + r_{34}c_R)(1 + (r_{12}LC)^2)(1 + (r_{13}EC)^2)(1 + (r_{14}c_{Tn})^2)} - m_{16}c_{P5}^{(m)} \tag{13}$$

$$P5_{trans} = t_5 c_{P5c} - t_6 c_{P5n} \tag{14}$$

$$\frac{dc_{P5c}}{dt} = c_{P5}^{(m)} - (m_{17} + m_{24}c_{ZTL})c_{P5c} - P5_{trans} \tag{15}$$

$$\frac{dc_{P5n}}{dt} = P5_{trans} - m_{42}c_{P5n} \tag{16}$$

$$T_{trans} = t_7 c_{Tc} - \frac{t_8}{1 + m_{37}c_{P5n}} c_{Tn} \tag{17}$$

$$\frac{dc_{T}^{(m)}}{dt} = \frac{1 + a_5 r_{35} c_R}{(1 + r_{35}c_R)(1 + (r_{15}LC)^2)(1 + (r_{16}EC)^2)(1 + (r_{17}c_T n)^2)} - m_5 c_{T}^{(m)} \tag{18}$$

$$\frac{dc_{Tn}}{dt} = T_{trans} - \frac{m_{43}}{1 + m_{38}c_{P5n}} c_{Tn} \tag{19}$$

$$\frac{dc_{Tc}}{dt} = c_{T}^{(m)} - (m_8 + m_6 c_{ZTL})c_{Tc} - T_{trans} \tag{20}$$

$$\frac{dc_{E4}^{(m)}}{dt} = \frac{1 + a_6 r_{36} c_R}{(1 + r_{36}c_R)(1 + (r_{18}EC)^2)(1 + (r_{19}LC)^2)(1 + (r_{20}c_{Tn})^2)} - m_7 c_{E4}^{(m)} \tag{21}$$

$$\frac{dc_{E4}}{dt} = p_{23}c_{E4}^{(m)} - m_{35}c_{E4} - {c_{E4}}^2 \tag{22}$$

$$E34_{prod} = p_{25}c_{E3}c_{E4d} \tag{23}$$

$$\frac{dc_{E4d}}{dt} = {c_{E4}}^2 - m_{36}c_{E4d} - E34_{prod} \tag{24}$$

$$\frac{dc_{E3}^{(m)}}{dt} = \frac{1}{1 + (r_{21}LC)^2} - m_{26}c_{E3}^{(m)} \tag{25}$$

$$E3_{deg} = (m_{30}c_{COP1d} + m_{29}c_{COP1n} + m_9 + m_{10}c_{Gn}) \tag{26}$$

$$\frac{dc_{E3}}{dt} = p_{16}c_{E3}^{(m)} - E34_{prod} - E3_{deg}c_{E3} \tag{27}$$

$$\frac{dc_{E34}}{dt} = E34_{prod} - m_{22}c_{E34}E3_{deg} \tag{28}$$

$$\frac{dc_{LUX}^{(m)}}{dt} = \frac{1 + a_7 r_{37} c_R}{(1 + r_{37}c_R)(1 + (r_{22}EC)^2)(1 + (r_{23}LC)^2)(1 + (r_{24}c_{Tn})^2)} - m_{34}c_{LUX}^{(m)} \tag{29}$$

$$\frac{dc_{LUX}}{dt} = c_{LUX}^{(m)} - m_{39}c_{LUX} \tag{30}$$

$$\frac{dc_{COP1c}}{dt} = n_5 - p_6 c_{COP1c} - m_{27}c_{COP1c}(1 + p_{15}L) \tag{31}$$

$$\frac{dc_{COP1n}}{dt} = p_6 c_{COP1c} - (n_{14} + n_6 L c_P)c_{COP1n} - m_{27}c_{COP1n}(1 + p_{15}L) \tag{32}$$

$$\frac{dc_{COP1d}}{dt} = (n_{14} + n_6 L c_P) c_{COP1n} - m_{31}(1 + m_{33} D) c_{COP1d} \tag{33}$$

$$ZG_{prod} = p_{12} c_{ZTL} c_{Gc} - (p_{13} D + p_{10} L) c_{ZG} \tag{34}$$

$$\frac{dc_{ZTL}}{dt} = p_{14} - ZG_{prod} - m_{20} c_{ZTL} \tag{35}$$

$$\frac{dc_{ZG}}{dt} = ZG_{prod} - m_{21} c_{ZG} \tag{36}$$

$$\frac{dc_G^{(m)}}{dt} = \frac{1 + a_8 r_{38} c_R}{(1 + r_{38} c_R)(1 + (r_{25} EC)^2)(1 + (r_{26} LC)^2)(1 + (r_{27} c_{Tn})^2)} - m_{18} c_G^{(m)} \tag{37}$$

$$c_{E3tot} = c_{E3} + c_{E34} \tag{38}$$

$$G_{trans} = p_{28} c_{Gc} - \frac{p_{29}}{1 + t_9 c_{E3tot}} c_{Gn} \tag{39}$$

$$\frac{dc_{Gc}}{dt} = p_{11} c_G^{(m)} - ZG_{prod} - G_{trans} - m_{19} c_{Gc} \tag{40}$$

$$\frac{dc_{Gn}}{dt} = G_{trans} - m_{19} c_{Gn} - m_{25} c_{E3tot}(1 + m_{28} c_{COP1d} + m_{32} c_{COP1n}) c_{Gn} \tag{41}$$

$$\frac{dc_{NOX}^{(m)}}{dt} = \frac{1}{(1 + (r_{28} LC)^2)(1 + (r_{29} c_{P7})^2)} - m_{44} c_{NOX}^{(m)} \tag{42}$$

$$\frac{dc_{NOX}}{dt} = c_{NOX}^{(m)} - m_{45} c_{NOX} \tag{43}$$

$$\frac{dc_R^{(m)}}{dt} = \frac{1}{1 + (r_{30} c_{P9})^2 + (r_{31} c_{P7})^2 + (r_{32} c_{P5n})^2} - m_{46} c_R^{(m)} \tag{44}$$

$$\frac{dc_R}{dt} = c_R^{(m)} - m_{47} c_R \tag{45}$$

Model variants

In the model without RVE8, $c_R^{(m)}$ and c_R were set to 0, and all data for RVE8 and the *rve* mutants were removed from the cost function. For testing NOX as an activator of *CCA1* and *LHY*, an activation term, $a_1 c_{NOX}/(1+r_{39} c_{NOX})$, was added to the numerator of eq. (2). Similarly, the activation of *PRR9* transcription by CCA1 and LHY was implemented by the addition of $a_2(r_5 LC)^2$ to the numerator in eq. (9).

Parameter sensitivity analysis

The sensitivity of the cost function to perturbations in the parameter values are presented in Figure **??**, which shows that the parameter sets generally agree on which parameters are sensitive to perturbations. However, parameters with high sensitivity are not necessarily constant between parameter sets.

Figure 4 shows that there is only a very weak correlation between the variability of a parameter between parameter sets and the robustness of the model to changes in that parameter. Thus, parameter sensitivity cannot be used to estimate how widely a parameter can vary between alternative parameter sets. Even though we have removed any obviously redundant parameters from the equations, the model is likely to be constraining many

nonlinear functions of several parameters rather than the individual parameters. That is, the parameter values are often meaningful only in the context of their respective parameter sets.

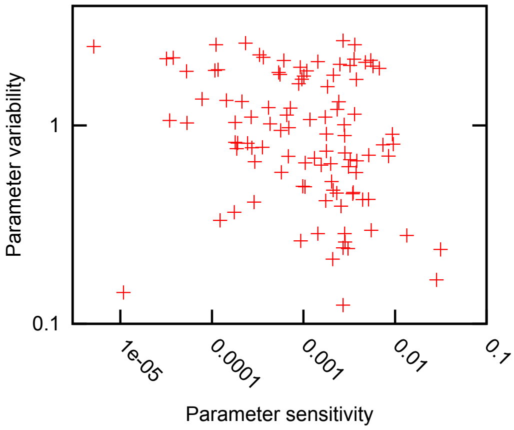

Figure 4. Parameter sensitivity and variability. For each parameter, the variability between parameter sets is plotted against the sensitivity of the parameter. Variability is defined as the standard deviation of the logarithm of the parameter value across the eight parameter sets. Sensitivity is defined as the mean relative change in the cost function when the parameter is increased and decreased by 10%, averaged over the parameter sets.

Model period predictions

To independently verify the output of the model, we compared with experimental data for the relative period change between wild type and mutants.

Experiment	Light cond	wt (exp)	Mutant (exp)	Change (exp)	Change (sim)	Source
toc1 RNAi	LL	24.27	20.51	-3.72	-1.50	[26]
toc1-1	LL	24.5	21	-3.43	-1.50	[27]
toc1-1	LL	24.82	22.46	-2.28	-1.50	[8]
cca1-1	LL	26.41	24.77	-1.49	-0.76	[18]
cca1-1	LL	25.31	23.82	-1.41	-0.76	[11]
cca1-11	LL	26.02	23.25	-2.55	-0.76	[8]
cca1-11;lhy-21	RR	24.5	18.2	-6.17	-5.90	[28]
cca1-11;lhy-21	LL	26.02	17.4	-7.95	-5.90	[8]
cca1-11;lhy-21	LL	26.41	19.73	-6.07	-5.90	[18]
cca1-1;lhy-R	LL	23.99	arr		-1.82	[11]
lhy	LL	22.71	23.64	0.98	-1.07	[9]
lhy$^{\text{TN104}}$	LL	22.71	24.67	2.07	-1.07	[9]
prr7-3	LL	24.3	25.0	0.69	1.27	[29]
prr7-3;prr9-1	LL	24.3	36.2	11.75	0.30	[29]
prr9-1	LL	24.3	24.8	0.49	1.26	[29]
PRR5-ox	LL	23.41	22.66	-0.77	-0.30	[30]
NOX-ox	LL	25.15	29.95	4.58	2.16	[8]

Experiment	Light cond	wt (exp)	Mutant (exp)	Change (exp)	Change (sim)	Source
nox-1	LL	24.47	23.23	-1.22	-0.05	[8]
lux	LL		arr		1.93	[31]
elf3-1	LL		arr		0.90	[5]
elf4	LL	23.8	22.3	-1.51	2.42	[32]
ELF4-ox	LL	27.308	30.89	3.15	-0.63	[33]
ELF4-ox	LL	25.1	28.75	3.49	-0.63	[34]
ELF3-ox	LL	26.25	26.85	0.55	1.25	[34]
ELF3-ox	LL	24.28	26.41	2.11	1.25	[35]
elf4-1;ELF3-ox	LL	26.25	27.05	0.73	3.23	[34]
ztl-22	LL	26.1	33.0	6.34	2.59	[36]
ztl-21	LL	26.3	27.7	1.28	2.59	[36]
ztl-21	RR	24.5	27.1	2.55	2.59	[28]
ztl-1	BB	24.9	28.95	3.90	2.59	[37]
ztl-3	RR	24.9	29.4	4.34	2.59	[37]
ztl-1 (Bx4)	LL	27.3	32.0	4.13	2.59	[38]
ztl-2 (Bx1)	LL	27.3	32.8	4.84	2.59	[38]
RVE8-ox	LL	24	22.16	-1.84	-1.07	[39]
rve8	LL	24	25.68	1.68	0.15	[39]
gi-11	LL	24.4	23.4	-0.98	-0.83	[13]
gi-201	LL	25.12	24.44	-0.65	-0.83	[40]
toc1-1	DD	27.5	22.3	-4.54	0.54	[27]
gi-201	DD	27.48	arr		-0.29	[40]
elf3-1	DD	25.07	25.41	0.33	1.46	[35]
elf4-1;ELF3-ox	DD	28.0	30.3	1.97	1.12	[34]
elf4	DD	26.4	27.1	0.64	0.88	[32]
ELF3-ox	DD	28.0	29.5	1.29	1.06	[34]
ELF3-ox	DD	25.07	25.10	0.03	1.06	[35]
ztl-22	DD	27.05	33.56	5.78	3.82	[36]
ztl-27	DD	27.05	36.43	8.32	3.82	[36]
prr7-3	DD	25.7	25.8	0.09	-0.16	[29]
NOX-ox	DD	24.95	26.85	1.83	0.01	[8]

Table 2. Period change in mutants, compared between experiments and the F2014 model. The change in period between mutant, x, and wild type, y, is computed as $(x-y)\frac{24}{y}$. Experimental data were averaged where replicates were available within a publication (e.g. *toc1* RNAi [26], *ztl-1/3* [37], ELF4-ox [33], ELF3-ox [34], NOX-ox [8], RVE8-ox, *rve8* [39], and *cca1-1* [11]). The periods from the model were taken as the mean, across the eight parameters sets, of the median of the period of *TOC1*, *CCA1* and *PRR5* mRNA. Simulations were run in LD 12:12 and then transferred to constant light (LL) or darkness (DD) for four days. Some experiments were performed in constant red (RR), or blue (BB) light; these were simulated as LL. The experimental periods were largely based on luciferase data which were not used to fit the model. Mutants marked with "arr" were found to be arrhythmic.

The eight best fitted parameter sets

Parameter	1	2	3	4	5	6	7	8
a_1	5.314	1.022	6.962	4.835	9.946	9.707	9.971	9.964
a_4	7.175	9.866	4.687	9.776	8.412	7.882	7.139	1.155
a_5	1.96	5.353	1	5.004	4.883	9.988	2.672	1.112
a_6	8.874	2.278	2.459	1.371	2.25	1.821	1.04	1
a_7	1.528	5.917	1	9.246	1.73	1.005	9.33	8.058
a_8	3.014	4.365	6.927	9.413	7.008	9.914	2.72	9.866
r_1	5.673	3.747	11.45	1.182	3.794	3.458	1.028	1.24
r_2	1.395	2.384	2.638	5.219	3.087	2.826	4.889	3.528
r_3	8.556	4.747	1.171	7.636	9.789	3.979	0.01852	9.399
r_4	3.422	16.3	5.842	94.55	0.1007	0.1002	0.1057	0.1001
r_5	0.1532	0.1	0.1	0.1183	0.1001	0.1001	0.1002	0.1001
r_6	25.77	0.4728	96.91	275.3	41.25	31.46	1.286	176.2
r_7	12.27	35.94	23.2	231.6	2.785	0.4538	13.76	12.07
r_8	1.753	2.225	1.867	1.574	1.401	1.785	0.3183	1.026
r_9	30.75	0.5885	153.7	363.5	47.7	39.29	2.544	396.5
r_{10}	9.981	21.8	11.88	81.95	3.601	0.01082	26.78	11.29
r_{11}	1.941	2.086	1.59	1.066	1.232	2.428	1.6	1.102
r_{12}	4.987	6.033	3.409	3.455	4.937	10.59	15.17	1.303
r_{13}	44.99	1.053	142.6	443.7	58.63	35.93	4.415	326.5
r_{14}	3.904	12.66	8.865	0.1209	1.807	0.3359	94.19	17.22
r_{15}	5.344	6.743	18.56	4.354	5.036	10.07	6.786	11.3
r_{16}	9.74	0.1519	52.48	82.46	13.34	8.783	0.9119	84.15
r_{17}	2.25	5.199	41.75	0.3816	1.775	0.8467	61.33	14.62
r_{18}	41.94	1.205	291.2	493.8	63.51	22.02	3.123	385.8
r_{19}	15.93	16.24	26.48	12.24	12.13	11.21	7.592	29.8
r_{20}	0.109	0.1465	0.1458	0.4281	0.6299	0.01341	0.2518	12.3
r_{21}	3.336	5.127	3.068	2.549	1.704	13.25	2.484	4.045
r_{22}	56.5	1.971	182.5	150.7	33.31	93.73	6.552	725.3
r_{23}	3.28	7.1	4.992	4.912	2.576	4.767	4.378	3.856
r_{24}	4.315	16.33	10.8	54.31	4.513	0.106	18.25	0.101
r_{25}	41.19	1.027	156.9	365.3	43.43	39	2.794	368.7
r_{26}	4.774	5.466	4.54	3.558	4.259	6.963	3.439	4.691
r_{27}	1.34	6.864	4.343	1.108	2.083	0.4267	17.04	7.185
r_{28}	5.91	8.392	8.667	4.178	3.885	11.58	4.722	7.319
r_{29}	0.2745	0.1423	0.1	1.732	0.7494	0.1001	0.1002	0.1
r_{30}	1.411	2.714	6.728	0.5166	0.5525	3.9	0.2139	0.1194
r_{31}	0.03975	0.01041	0.2149	0.1999	1.944	0.3978	2.477	2.029
r_{32}	6.967	4.775	1.069	7.782	10.91	3.516	0.1142	0.04042
r_{33}	0.7146	0.9026	0.03532	0.5893	0.06372	0.1207	0.1105	0.8943
r_{34}	2.874	0.05704	1.302	0.1891	1.021	1.025	9.955	6.847
r_{35}	0.06041	0.02929	0.2607	0.1446	0.03939	0.02616	9.917	0.02871
r_{36}	0.08923	0.49	0.5084	2.492	8.706	1.344	0.02061	0.01041
r_{37}	9.958	0.554	0.01093	0.5246	0.1416	0.04229	0.1703	0.08021
r_{38}	3.887	0.05062	0.3151	0.1267	0.1986	0.6058	0.9035	0.3665
r_{40}	1.422	1.051	0.2785	1.016	2.263	1.208	1.681	5.28
r_{41}	1.856	0.3341	0.3861	0.6126	2.295	1.034	9.883	2.965
f_1	0.06021	0.409	0.1273	0.4442	0.4195	0.09476	0.3549	0.04486
f_2	0.02732	2.021	0.01001	0.01015	0.02322	0.07358	7.587	2.726
f_3	0.2654	0.03313	4.774	48.57	1.886	6.904	0.01097	3.993
f_4	0.2687	0.1	0.1848	5.627	0.1	0.187	0.6628	1.854
f_5	0.2899	0.3853	0.2802	0.4432	0.353	0.2547	0.2058	0.4459
f_6	0.08977	0.2525	0.03518	0.08095	1.744	0.0169	0.09316	0.2516
t_5	0.2518	1.103	1.484	0.6334	3.107	6.618	0.5109	0.1847
t_6	0.112	0.5891	0.6062	1.099	2.985	1.76	0.2073	0.9535
t_7	3.772	0.2317	13.31	0.1221	0.2228	0.3602	0.6104	3.268
t_8	28.34	0.1472	1.198	3.449	0.1286	0.5261	0.1402	2.619
t_9	2.543	0.8543	1.947	0.9492	0.1006	1.171	3.011	3.302
m_1	0.6127	0.996	0.7966	0.6529	0.4437	0.6407	0.5032	0.7201
m_3	0.6154	0.5889	0.472	0.6059	0.5981	0.5305	0.3759	0.4505
m_4	0.4322	0.3761	0.4737	0.5013	0.5489	0.5424	0.4513	0.5409
m_5	1.869	2.3	0.5026	0.9276	0.8059	0.6966	0.5001	0.961
m_6	0.451	0.0133	0.1935	0.05858	2.764	0.03575	0.01039	0.01
m_7	1.277	0.6487	0.6006	0.6056	0.6031	0.7777	0.7167	0.6031
m_8	0.1	5.437	3.417	5.527	2.369	2.336	4.77	0.5255
m_9	0.1886	0.1225	0.3288	0.2881	0.07189	0.1356	0.01001	0.03257
m_{10}	0.01001	0.01001	0.01	0.01087	0.2276	0.01072	3.854	0.2368
m_{11}	0.7611	0.6771	0.6066	0.7547	1.042	1.191	0.5066	0.8838
m_{12}	2.57	1.988	2.987	2.085	1.146	2.72	1.726	1.597
m_{13}	0.6654	0.376	0.6748	0.2103	0.3006	0.6107	0.2658	0.2214
m_{14}	0.5015	4.916	0.5044	3.482	0.5081	0.5002	0.6408	0.5017

Parameter	1	2	3	4	5	6	7	8
m_{15}	0.1791	0.09303	0.1774	0.2039	0.241	0.2177	0.2261	0.1897
m_{16}	0.5383	0.5828	0.4082	0.6494	2.579	2.917	1.366	0.2781
m_{17}	0.07499	0.04744	0.108	0.1645	0.1993	0.1999	0.07084	0.1023
m_{18}	4.505	2.426	1.549	2.13	2.219	1.41	1.211	1.192
m_{22}	0.3007	0.3012	0.3003	0.3006	0.3002	2.409	0.3835	0.4269
m_{23}	0.08544	0.1764	0.09547	0.1197	0.0651	0.03943	0.08041	0.07392
m_{24}	1.5	2.848	4.21	1.874	2.577	5.882	1.152	7.667
m_{25}	0.6526	0.4176	0.2553	0.4625	0.08625	0.3877	0.1015	0.0686
m_{26}	1.032	1.57	0.7937	0.8424	1.04	0.3001	0.7549	0.6028
m_{28}	0.0382	0.02757	0.02349	0.4466	7.57	8.143	9.986	1.737
m_{29}	0.01001	0.01	0.01	0.05984	0.168	0.01032	0.01	0.03227
m_{30}	4.949	5.491	3.84	5.204	7.242	1.795	9.875	3.856
m_{32}	9.998	5.681	6.275	8.609	6.993	3.588	9.996	5.083
m_{34}	0.1561	0.1115	0.2087	0.8513	0.2252	0.1086	0.1061	0.1566
m_{35}	0.9413	0.9188	1.184	6.928	6.751	1.56	0.7253	5.421
m_{36}	0.506	0.5711	0.5108	0.5072	0.502	9.773	0.5003	0.5017
m_{37}	0.01001	0.9391	0.03945	0.01014	0.6989	2.776	0.03128	0.01007
m_{38}	1.774	7.975	0.07585	25.38	9.964	44.21	0.2389	0.1916
m_{39}	0.2001	0.216	0.2012	0.2854	0.2021	1.619	0.202	0.447
m_{42}	0.9229	0.3759	0.2279	0.456	0.1023	0.09626	0.3508	0.3184
m_{43}	1.139	0.5214	0.03266	0.05359	0.07754	0.06792	0.02179	0.001505
m_{44}	0.6769	0.3577	0.4285	1.443	4.363	0.3769	0.4726	0.4493
m_{45}	0.8039	0.7944	9.998	1.633	5.051	0.8805	6.154	8.512
m_{46}	5.273	0.7541	0.9665	0.8438	0.7331	0.5034	0.7004	2.599
m_{47}	0.2466	0.1293	0.1668	0.1838	0.2269	0.1458	0.2496	0.2389
p_{11}	1.912	1.78	0.6623	0.684	3.208	0.4549	1.356	1.939
p_{16}	0.1211	0.4024	0.1647	0.2261	0.1254	0.3022	0.1949	0.1181
p_{23}	1.011	1.461	3.951	14.66	5.237	29.92	11.17	29.93
p_{25}	1.003	1.111	1.004	1.213	4.415	4.268	4.768	1.017
p_{28}	1.061	2.13	1.187	1.622	8.092	1.104	1.66	3.725
p_{29}	10.18	25.2	5.827	23.91	3.122	19.15	3.93	5.9
q_1	0.2607	0.1217	0.5518	0.3	0.9744	1.543	1.358	0.5445
q_3	0.4659	0.2873	0.312	1.13	1.225	1.666	9.682	2.453

Table 3. The eight best parameter sets. The values of the parameters after optimization with parallel tempering from random initial starting points in parameter space, as described in Methods.

Parameter	Value
m_{19}	0.2
m_{20}	1.8
m_{21}	0.1
m_{27}	0.1
m_{31}	0.3
m_{33}	13
n_5	0.23
n_6	20
n_{14}	0.1
p_6	0.6
p_7	0.3
p_{10}	0.2
p_{12}	8
p_{13}	0.7
p_{14}	0.3
p_{15}	3

Table 4. Constant parameters. These parameters control c_P, COP1, ZTL and the ZTL-GI complex, and were not included in the optimization process. Instead, they were taken from P2012 (c_P and COP1) or fitted manually (ZTL and ZTL-GI).

References

[1] Yu JW, Rubio V, Lee NY, Bai S, Lee SY, et al. (2008) COP1 and ELF3 control circadian function and photoperiodic flowering by regulating GI stability. Mol Cell 32: 617–630.

[2] Kolmos E, Nowak M, Werner M, Fischer K, Schwarz G, et al. (2009) Integrating ELF4 into the circadian system through combined structural and functional studies. HFSP J 3: 350–366.

[3] Chow BY, Helfer A, Nusinow DA, Kay SA (2012) ELF3 recruitment to the PRR9 promoter requires other evening complex members in the *Arabidopsis* circadian clock. Plant Signal Behav 7: 170–173.

[4] Liu XL, Covington MF, Fankhauser C, Chory J, Wagner DR (2001) ELF3 encodes a circadian clock–regulated nuclear protein that functions in an *Arabidopsis* PHYB signal transduction pathway. Plant Cell 13: 1293–1304.

[5] Hicks KA, Albertson TM, Wagner DR (2001) EARLY FLOWERING 3 encodes a novel protein that regulates circadian clock function and flowering in *Arabidopsis.* Plant Cell 13: 1281–1292.

[6] Lu SX, Webb CJ, Knowles SM, Kim SH, Wang Z, et al. (2012) CCA1 and ELF3 interact in the control of hypocotyl length and flowering time in *Arabidopsis.* Plant Physiol 158: 1079–1088.

[7] Dixon LE, Knox K, Kozma-Bognar L, Southern MM, Pokhilko A, et al. (2011) Temporal repression of core circadian genes is mediated through EARLY FLOWERING 3 in *Arabidopsis.* Curr Biol 21: 120–125.

[8] Dai S, Wei X, Pei L, Thompson RL, Liu Y, et al. (2011) BROTHER OF LUX ARRHYTHMO is a component of the *Arabidopsis* circadian clock. Plant Cell 23: 961–972.

[9] Schaffer R, Ramsay N, Samach A, Corden S, Putterill J, et al. (1998) The late elongated hypocotyl mutation of *Arabidopsis* disrupts circadian rhythms and the photoperiodic control of flowering. Cell 93: 1219–1229.

[10] Wang ZY, Tobin EM, et al. (1998) Constitutive expression of the CIRCADIAN CLOCK ASSOCIATED 1 (CCA1) gene disrupts circadian rhythms and suppresses its own expression. Cell 93: 1207–1218.

[11] Alabadı D, Yanovsky MJ, Más P, Harmer SL, Kay SA (2002) Critical role for CCA1 and LHY in maintaining circadian rhythmicity in *Arabidopsis.* Curr Biol 12: 757–761.

[12] Pruneda-Paz JL, Breton G, Para A, Kay SA (2009) A functional genomics approach reveals CHE as a component of the *Arabidopsis* circadian clock. Science 323: 1481–1485.

[13] Gould PD, Locke JC, Larue C, Southern MM, Davis SJ, et al. (2006) The molecular basis of temperature compensation in the *Arabidopsis* circadian clock. Plant Cell 18: 1177–1187.

[14] Higo K, Ugawa Y, Iwamoto M, Korenaga T (1999) Plant cis-acting regulatory DNA elements (PLACE) database: 1999. Nucleic Acids Res 27: 297–300.

[15] Prestridge DS (1991) SIGNAL SCAN: a computer program that scans DNA sequences for eukaryotic transcriptional elements. Computer applications in the biosciences: CABIOS 7: 203–206.

[16] Mizoguchi T, Wheatley K, Hanzawa Y, Wright L, Mizoguchi M, et al. (2002) LHY and CCA1 are partially redundant genes required to maintain circadian rhythms in *Arabidopsis*. Dev Cell 2: 629–641.

[17] Alabadı D, Oyama T, Yanovsky MJ, Harmon FG, Más P, et al. (2001) Reciprocal regulation between TOC1 and LHY/CCA1 within the *Arabidopsis* circadian clock. Science 293: 880–883.

[18] Lu SX, Knowles SM, Andronis C, Ong MS, Tobin EM (2009) CIRCADIAN CLOCK ASSOCIATED 1 and LATE ELONGATED HYPOCOTYL function synergistically in the circadian clock of *Arabidopsis*. Plant Physiol 150: 834–843.

[19] Yakir E, Hilman D, Kron I, Hassidim M, Melamed-Book N, et al. (2009) Posttranslational regulation of CIRCADIAN CLOCK ASSOCIATED 1 in the circadian oscillator of *Arabidopsis*. Plant Physiol 150: 844–857.

[20] Yakir E, Hilman D, Hassidim M, Green RM (2007) CIRCADIAN CLOCK ASSOCIATED 1 transcript stability and the entrainment of the circadian clock in *Arabidopsis*. Plant Physiol 145: 925–932.

[21] Kim JY, Song HR, Taylor BL, Carré IA (2003) Light-regulated translation mediates gated induction of the *Arabidopsis* clock protein LHY. EMBO J 22: 935–944.

[22] Wang L, Fujiwara S, Somers DE (2010) PRR5 regulates phosphorylation, nuclear import and subnuclear localization of TOC1 in the *Arabidopsis* circadian clock. EMBO J 29: 1903–1915.

[23] Kiba T, Henriques R, Sakakibara H, Chua NH (2007) Targeted degradation of PSEUDO-RESPONSE REGULATOR 5 by an SCFZTL complex regulates clock function and photomorphogenesis in *Arabidopsis thaliana*. Plant Cell 19: 2516–2530.

[24] Fujiwara S, Wang L, Han L, Suh SS, Salomé PA, et al. (2008) Post-translational regulation of the *Arabidopsis* circadian clock through selective proteolysis and phosphorylation of pseudo-response regulator proteins. J Biol Chem 283: 23073–23083.

[25] Kim WY, Fujiwara S, Suh SS, Kim J, Kim Y, et al. (2007) ZEITLUPE is a circadian photoreceptor stabilized by GIGANTEA in blue light. Nature 449: 356–360.

[26] Más P, Alabadí D, Yanovsky MJ, Oyama T, Kay SA (2003) Dual role of TOC1 in the control of circadian and photomorphogenic responses in *Arabidopsis*. Plant Cell 15: 223–236.

[27] Strayer C, Oyama T, Schultz TF, Raman R, Somers DE, et al. (2000) Cloning of the *Arabidopsis* clock gene TOC1, an autoregulatory response regulator homolog. Science 289: 768–771.

[28] Johansson M, McWatters HG, Bakó L, Takata N, Gyula P, et al. (2011) Partners in time: EARLY BIRD associates with ZEITLUPE and regulates the speed of the *Arabidopsis* clock. Plant Physiol 155: 2108–2122.

[29] Farré EM, Harmer SL, Harmon FG, Yanovsky MJ, Kay SA (2005) Overlapping and distinct roles of PRR7 and PRR9 in the *Arabidopsis* circadian clock. Curr Biol 15: 47–54.

[30] Baudry A, Ito S, Song YH, Strait AA, Kiba T, et al. (2010) F-box proteins FKF1 and LKP2 act in concert with ZEITLUPE to control *Arabidopsis* clock progression. Plant Cell 22: 606–622.

[31] Onai K, Ishiura M (2005) PHYTOCLOCK 1 encoding a novel GARP protein essential for the *Arabidopsis* circadian clock. Genes Cells 10: 963–972.

[32] Doyle MR, Davis SJ, Bastow RM, McWatters HG, Kozma-Bognár L, et al. (2002) The ELF4 gene controls circadian rhythms and flowering time in *Arabidopsis thaliana*. Nature 419: 74–77.

[33] McWatters HG, Kolmos E, Hall A, Doyle MR, Amasino RM, et al. (2007) ELF4 is required for oscillatory properties of the circadian clock. Plant Physiol 144: 391–401.

[34] Herrero E, Kolmos E, Bujdoso N, Yuan Y, Wang M, et al. (2012) EARLY FLOWERING 4 recruitment of EARLY FLOWERING 3 in the nucleus sustains the *Arabidopsis* circadian clock. Plant Cell 24: 428–443.

[35] Covington MF, Panda S, Liu XL, Strayer CA, Wagner DR, et al. (2001) ELF3 modulates resetting of the circadian clock in *Arabidopsis*. Plant Cell 13: 1305–1316.

[36] Kevei E, Gyula P, Hall A, Kozma-Bognár L, Kim WY, et al. (2006) Forward genetic analysis of the circadian clock separates the multiple functions of ZEITLUPE. Plant Physiol 140: 933–945.

[37] Somers DE, Kim WY, Geng R (2004) The F-box protein ZEITLUPE confers dosage-dependent control on the circadian clock, photomorphogenesis, and flowering time. Plant Cell 16: 769–782.

[38] Somers DE, Schultz TF, Milnamow M, Kay SA (2000) ZEITLUPE encodes a novel clock-associated pas protein from *Arabidopsis*. Cell 101: 319–329.

[39] Farinas B, Mas P (2011) Functional implication of the MYB transcription factor RVE8/LCL5 in the circadian control of histone acetylation. Plant J 66: 318–329.

[40] Martin-Tryon EL, Kreps JA, Harmer SL (2007) GIGANTEA acts in blue light signaling and has biochemically separable roles in circadian clock and flowering time regulation. Plant Physiol 143: 473–486.
